# Imidazole as a Pendant Reactivation Ligand Increases Efficacy Scope for Reactivation and Resurrection of Organophosphorus-Inhibited/Aged Cholinesterases by Quinone Methide Precursors

**DOI:** 10.1021/acschemneuro.5c00631

**Published:** 2026-02-19

**Authors:** Alex R. Lovins, Kevin A. Miller, Rose K. Homoelle, Hayden J. Hoover, Craig A. McElroy, Christopher S. Callam, Christopher M. Hadad

**Affiliations:** † Department of Chemistry and Biochemistry, 2647 Ohio State University, Columbus, Ohio 43210, United States; ‡ InfinixBio, 1507 Chambers Road, Columbus, Ohio 43212, United States

**Keywords:** acetylcholinesterase, butyrylcholinesterase, organophosphorus, aging, resurrection, reactivation

## Abstract

Organophosphorus (OP) inhibition of acetylcholinesterase (AChE) continues to pose a deadly risk to human health. Despite decades of research on oximes, improved therapeutics are still needed as most oximes fail to cross the blood-brain barrier, none exhibit efficacy against the OP-aged form of AChE, and reactivation by oximes results in a toxic byproduct. However, despite efforts to replace oxime therapeutics, many of the new candidates fall short in broad-scope activity or sufficient efficacy relative to the oxime therapeutics. Previously, researchers have used imidazole or Mannich phenol moieties as a basic therapeutic handle for reactivation of OP-inhibited forms of AChE. Herein, we report a novel strategy of utilizing Mannich phenol quinone methide precursors (QMPs), which are capable of reactivation and resurrection of OP-inhibited and OP-aged AChE, linked to a pendant *N*-heterocyclic ring reactivator moiety we hypothesize to act in both the mechanism for reactivation of OP-inhibited AChE and resurrection of OP-aged AChE. We tested our hypothesis *via* the synthesis and *in vitro* evaluation of 24 novel QMP therapeutics across 20 frameworks containing various *N*-heterocyclic ring linkages. These QMP therapeutics were tested against eight OP-inhibited forms of AChE, seven OP-inhibited forms of BChE, and four OP-aged forms of AChE to determine broad-scope efficacy. We identify **5** as an impressive lead therapeutic with efficacy against all tested OP-inhibited/aged forms of AChE alongside **3**, **9** and **11** as lead reactivators due to their impressive efficacy against all OP-inhibited forms of AChE. For reactivation against the tested OP compounds (250 μM, 1 h), **5** and **9** demonstrate >20% recovery of six of the eight OP-inhibited forms of AChE while **11** demonstrates >20% recovery of seven of the eight OP-inhibited forms of AChE. For resurrection against the tested OP compounds (250 μM, 24 h), **5** demonstrates >20% recovery of two of the four OP-aged forms of AChE. Indeed, **5**, **9**, and **11** demonstrate superior efficacy to the oxime controls.

## Graphical abstract

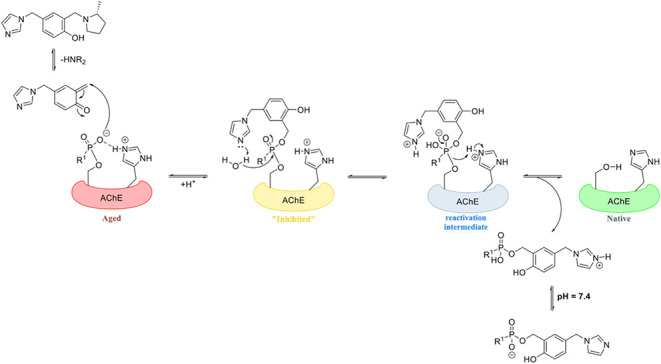

## Introduction

Organophosphorus (OP) compounds are systemic toxicants which exert their toxicity through covalent inhibition of the catalytic serine residue of acetylcholinesterase (AChE). This results in the inability for the enzyme to hydrolyze the neurotransmitter acetylcholine (ACh).^1,2^ Due to ACh having a wide variety of functions in the central and peripheral nervous systems (including muscle contractions), buildup of the neurotransmitter can result in respiratory failure.^3^ Such OP compounds have been used in terrorism, but they are also ubiquitous as pesticides, especially in developing countries.^4,5^

Commonly, oximes have been used to treat the OP-inhibited form of AChE. These compounds elicit their therapeutic effect *via* a direct nucleophilic displacement of the phosphorus from the phosphylated serine of the OP-inhibited state, thus restoring the native serine residue and enzymatic function.^6,7^ The recovery of this inhibited form to the native enzyme is termed “reactivation”. However, each OP compound results in a different phosphylated serine structure (Table 1), and depending on the groups attached for the OP compound, these differences can cause substantial difficulty for FDA-approved oxime therapeutics. Thus, no oxime therapeutic is capable of reactivation of every OP-inhibited form of AChE.^8^

**Table 1:**
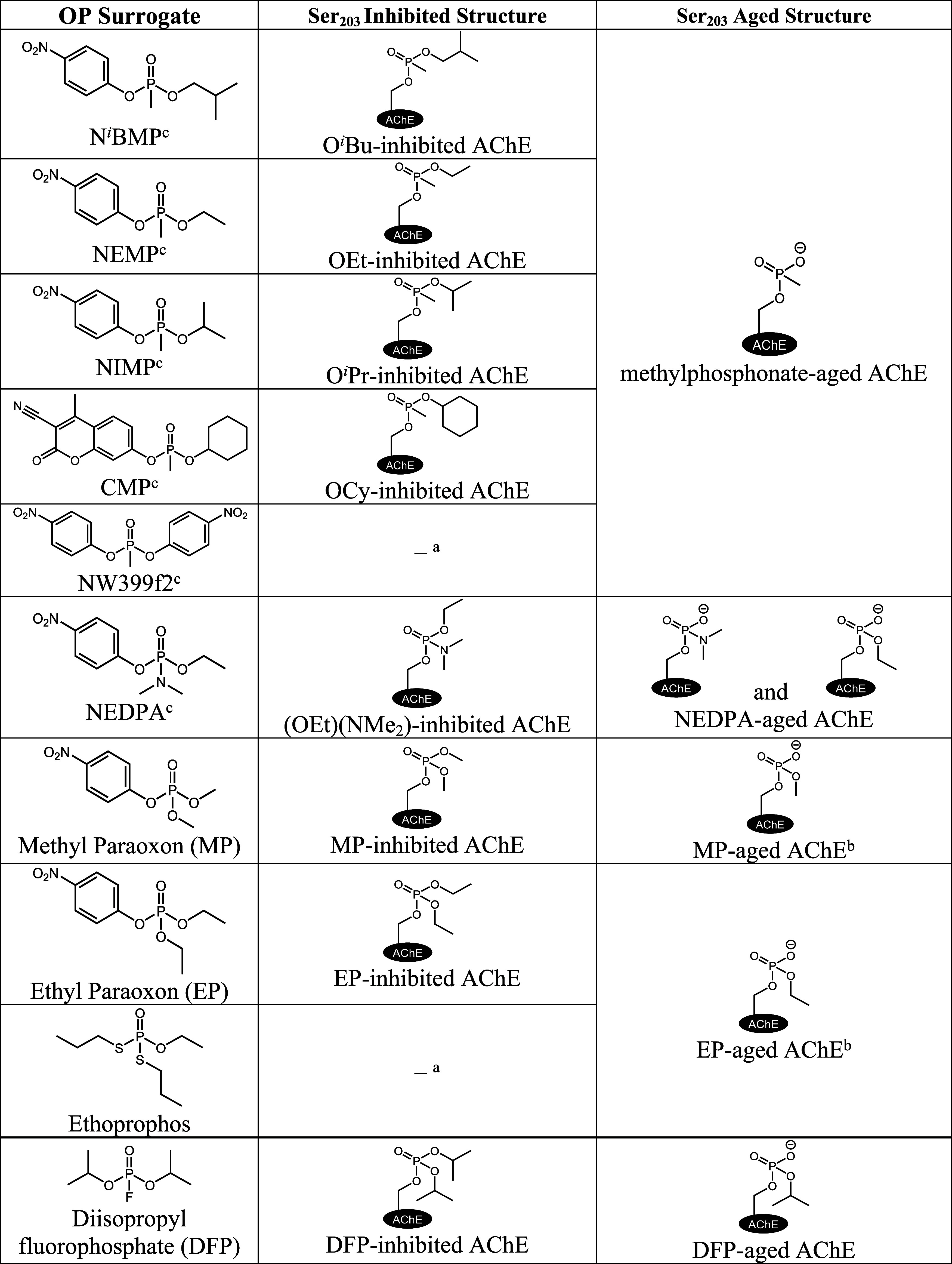
Structures of Organophosphorus (OP) Surrogates and Pesticides alongside Their Respective OP-Inhibited and OP-Aged forms of AChE with Analogous Structures Being Formed for BChE

a The OP-inhibited form is short-lived, and aging is near instantaneous.

b Recent studies identify alkylation of His447 as an alteration in the active site during aging to the OP-aged adduct; however, ethoprophos does not exhibit this alkylation.^20^

c The OP compounds indicated were synthesized following previously published procedures^21–23^ or obtained from generous collaborators.

Moreover, if left untreated, the inhibited structure can undergo a spontaneous *O*-dealkylation event, resulting in a phosphylated serine with a pendant oxyanion that is recalcitrant to reactivation by oximes.^9–11^ Depending on the OP-inhibited structure, aging half-lives span from days to mere minutes, leaving little window for oxime efficacy.^12^ In the same manner as inhibition, these OP-aged forms also vary in their structures, increasing the scope of exposures that must be treated (Table 1). And, many oxime therapeutics contain a permanent positive charge, reducing their efficacy in crossing the blood-brain barrier (BBB).^13–15^ Consequently, the variety of OP-inhibited and OP-aged structures of AChE creates a lethal biochemical challenge that oximes alone cannot effectively address.

Furthermore, butyrylcholinesterase (BChE) is another serine hydrolase enzyme present at high concentrations in the blood and brain but has no known essential biochemical function. However, due to its structural similarities to AChE, BChE can be inhibited by OP compounds in the same manner. As such, BChE acts as a stoichiometric scavenger of an OP exposure.^16^ Moreover, BChE can hydrolyze ACh as a natural substrate and can alleviate symptoms of OP exposure when AChE is impaired or not present.^17,18^ By performing reactivation and/or resurrection of OP-inhibited/aged BChE, the enzyme would be free to scavenge additional OP compounds, essentially creating a pseudocatalytic bioscavenger. Thus, a therapeutic that is capable of restoring the native enzyme from OP-inhibited/aged AChE and BChE may allow for the best chance of survival when exposed to OP compounds.^19^

In 2018, our team was the first to demonstrate *in vitro* recovery of methylphosphonate-aged AChE using a novel quinone methide precursor (QMP) approach, utilized previously for realkylation of peptides and phosphodiesters.^24–28^ The recovery of the aged enzyme to the native form has been termed “resurrection” by Quinn.^7,29^ We hypothesize the mechanism of resurrection occurs *via* the formation of a quinone methide (QM) which acts as an electrophile to the nucleophilic oxyanion.^24^ Upon realkylation, the phosphylated serine is in a state more similar to an inhibited form of the enzyme, upon which the phenol (or its conjugate base or an external base) is hypothesized to act as the reactivator and return the enzyme to its native form (Figure 1). However, this process is slow and difficult as demonstrated in our previous work in which many QMP compounds fail to recover over 10% of the methylphosphonate-aged enzyme in 24 h with micromolar concentrations.^22,30^ Indeed, this process requires multiple steps to recover the enzyme, and it is unknown which step is rate-limiting for the net recovery.

**Figure 1:**
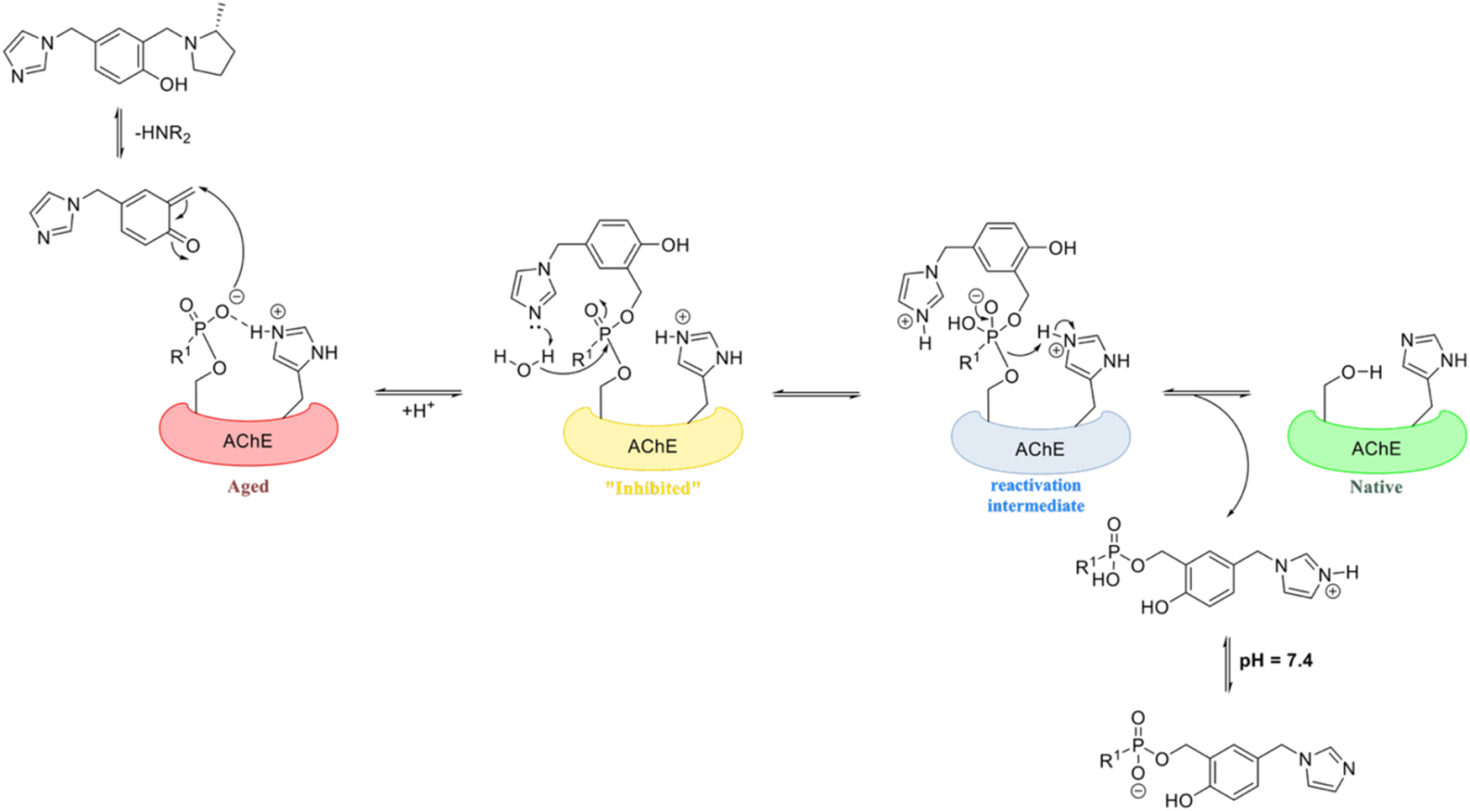
Hypothesized role of imidazole in the resurrection mechanism of OP-aged AChE by quinone methide precursors (QMPs).

Previously, our team also demonstrated the first dual-function therapeutics capable of performing both reactivation and resurrection of OP-inhibited/aged AChE.^22^ However, many of these compounds fail to demonstrate impressive reactivation and resurrection of all OP-inhibited/aged forms, indicating the need for optimization of the reactivator/resurrector moiety. In 2015, Katz et al. demonstrated that reactivation of OP-inhibited AChE was not limited to oximes and Mannich phenol moieties, but also could be performed using imidazole as a basic functionality (*via* the lead compound SP134).^31^ Furthermore, the imidazole was hypothesized to reactivate not through a direct nucleophilic addition, but rather by activation of a water molecule in accomplishing the overall reactivation step. Indeed, this water-mediated reactivation is seen as preferred over nucleophilic attack as the resulting hydrogen phosphate or phosphonate is acidic, thus forming an innocuous phosphate byproduct at physiological pH. Conversely, it is well documented that pyridinium oxime reactivators can create a toxic byproduct in the process of reactivation.^32,33^ The role of byproduct formation in an *in vivo* setting is unclear, but is highly dependent on the byproduct leaving the active site of the enzyme and other potential biological targets.^34^ Despite this, imidazole reactivators have seen very little development over time, whereas most research is still focused on charged oxime reactivators, despite their deficiencies for byproduct formation and poor penetrability into the central nervous system.^7,8,13^ Indeed, other researchers have attempted to develop classes of oximes that do not contain a charge to increase the BBB penetrability, these oximes often are less efficient than their charged counterparts.^35^

Thus, we hypothesized that linking an imidazole to a QMP framework will allow the imidazole to act as a pendant reactivator moiety for both the reactivation of OP-inhibited cholinesterases (Figure 2) as well as to act as a reactivator in the mechanism of QMP-mediated resurrection of OP-aged AChE (Figure 1). Herein, we demonstrate the synthesis and *in vitro* biochemical evaluation of 24 novel QMP therapeutics (Figure 3) with each containing an *N*-heterocyclic group (imidazole, benzimidazole, or pyrazole). We posit that the *N*-heterocyclic group may act both as a binding ligand in the active site as well as a reactivation moiety, greatly increasing the scope and efficacy of our dual-function QMP therapeutics. We sought to evaluate these hypotheses *via* *in vitro* evaluations of these QMP therapeutics, which are shown in Figure 3.

**Figure 2:**
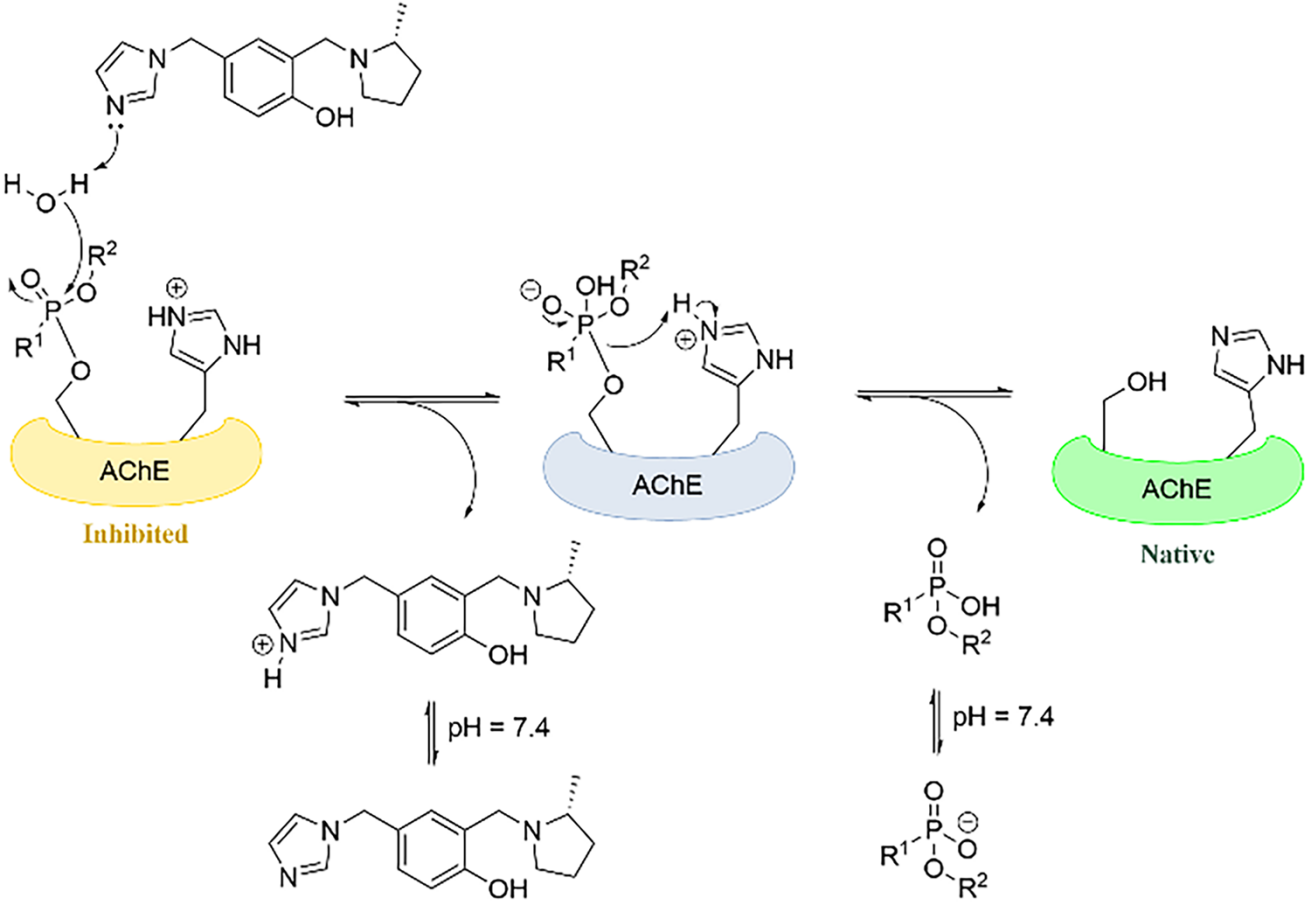
Hypothesized role of imidazole in the reactivation of OP-inhibited AChE.

**Figure 3:**
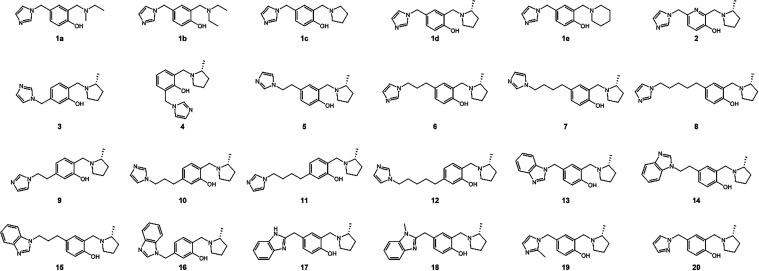
Chemical structures of synthesized *N*-heterocyclic-linked QMP compounds evaluated by biochemical *in vitro* screenings.

## Results and Discussion

### Synthesis of *N*-Heterocyclic QMP Compounds

Due to the number of different frameworks, the syntheses of the 24 QMPs were performed through a wide variety of synthetic routes. To begin, the imidazole linked in the *para* position to the hydroxyl as seen in framework **1a**–**e** was synthesized using a substitution, mediated through a transient quinone methide—guided by previously reported studies.^36,37^ Indeed, this reaction was of great use throughout this work as it was repurposed and used as the primary substitution reaction to generate frameworks in the *ortho* or *para* positions due to its robust nature, oftentimes reaching near 90% yield. This substitution was followed by a Mannich reaction to install the amine groups in these frameworks (Scheme 1). Compounds **1a**–**e** were determined to be an appropriate test of amine selectivity given the short and simple synthesis. However, given the previously observed QMP preference for the (*R*)-2-methylpyrrolidine as the leaving group,^22,30^ only framework **1** was used as a base framework to determine the differences between amine groups, while all other frameworks only featured the (*R*)-2-methylpyrrolidine.

**Scheme 1:**
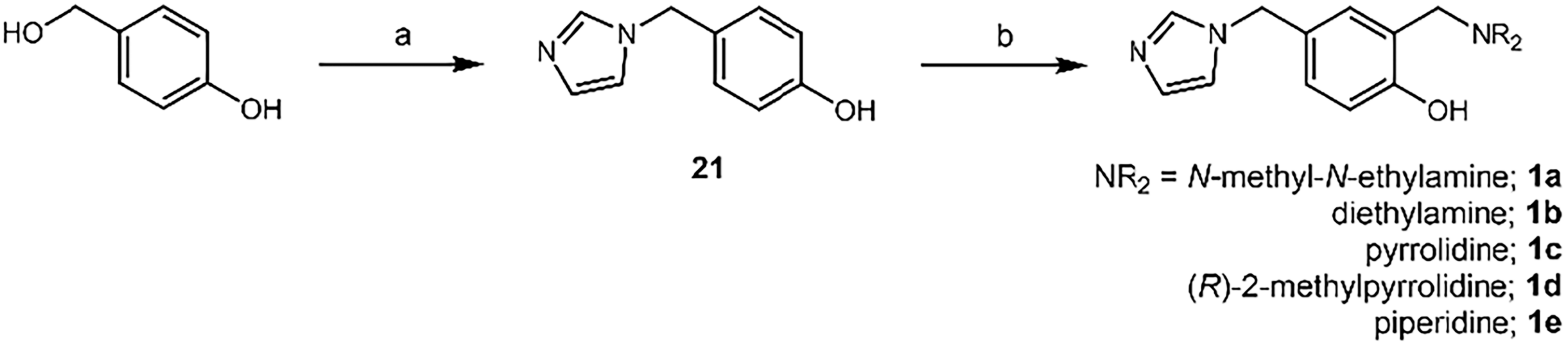
Synthesis of **1a–1e** ^a^

a (a) Imidazole, neat, 90 °C, 89%; (b) amine (1.5 equiv), paraformaldehyde (1.5 equiv), toluene, EtOH, 60–120 °C, 15–65%.

Next, we tried to synthesize the matched molecular pair of **1d** *via* synthesis of the pyridine-based QMP core (Scheme 2). An *m*-CPBA oxidation of 6-methylpyridin-3-ol generated the pyridine *N*-oxide.^38^ A Boekelheide rearrangement was used to prepare **23**,^38^ which, upon deprotection of the esters, allowed for the QM-mediated substitution as was used in framework **1** to prepare the imidazole **25**. Finally, a Mannich reaction using (*R*)-2-methylpyrrolidine was performed to prepare QMP **2**.

**Scheme 2:**
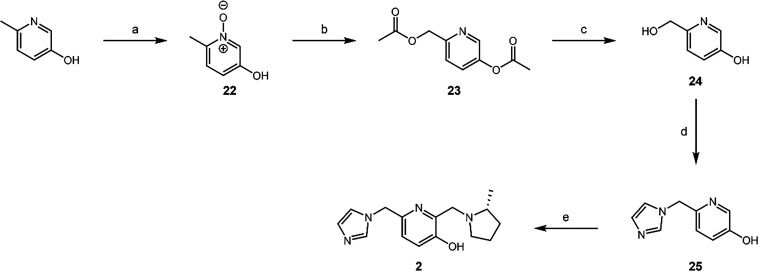
Synthesis of **2** ^a^

a (a) *m*-CPBA, chloroform, rt, 80%; (b) acetic anhydride, reflux, 76%; (c) LiOH, MeOH, H_2_O; (d) imidazole, neat, 120 °C; 18% yield for steps (c, d); (e) (*R*)-2-methylpyrrolidine (2 equiv), paraformaldehyde (2 equiv), toluene, EtOH, 120 °C, 77%.

Following the synthesis of **2**, the differences in efficacy from the regiochemistry of the imidazole was evaluated through synthesis of QMP therapeutics **3** and **4** (Schemes 3 and 4). Since the 5-position is unable to undergo the QM-mediated substitution, 3-methoxybenzyl chloride was used as the starting material. An S_N_2 reaction using imidazole was performed to add the imidazole ring, followed by a HBr-mediated demethylation to prepare the free phenol. A Mannich reaction then provided QMP therapeutic **3** in moderate yield. It should be noted that in **3**, the Mannich reaction led to only one isolable product (as shown in Scheme 3), and the possible *bis*-*ortho* QMP products from the sterically congested *ortho* position to both the *N*-heterocyclic ring and phenol moieties were not observed.

**Scheme 3:**
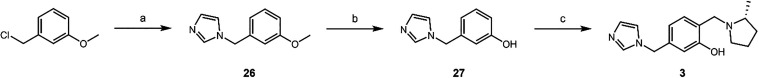
Synthesis of **3** ^a^

a (a) imidazole, Cs_2_CO_3_, MeCN, 70 °C, 67%; (b) HBr, neat, reflux, 44%; (c) (*R*)-2-methylpyrrolidine (3 equiv), paraformaldehyde (3 equiv), toluene, EtOH, 120 °C, 49%.

**Scheme 4:**
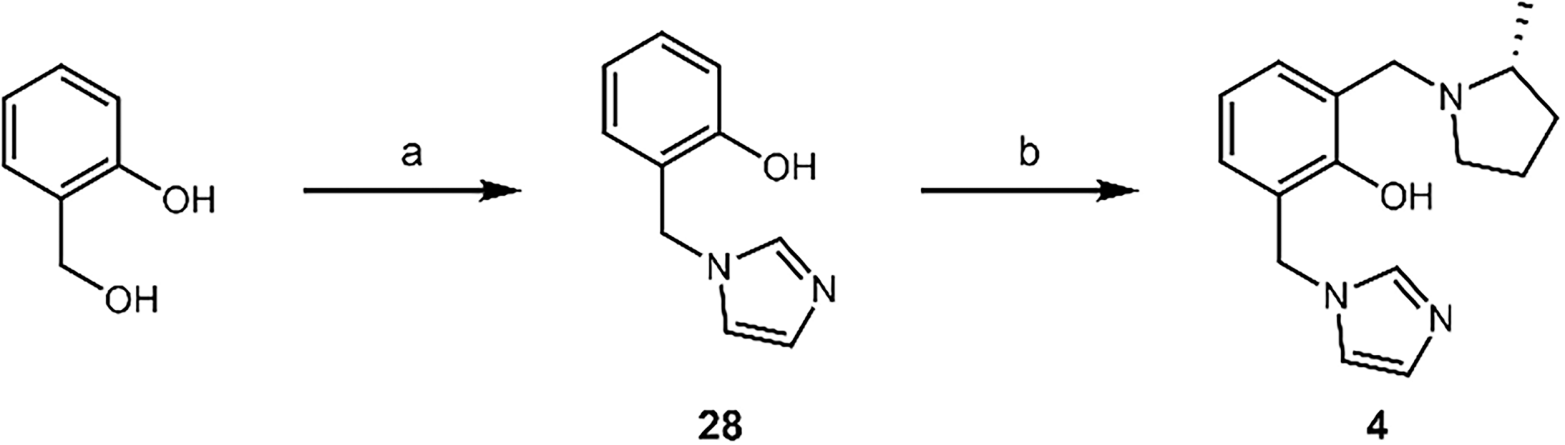
Synthesis of **4** ^a^

a (a) Imidazole, neat, 90 °C; (b) (*R*)-2-methylpyrrolidine (3 equiv), paraformaldehyde (3 equiv), toluene, EtOH, 120 °C; 28% 2-step yield for (a, b).

Installation of the imidazole in the 6-position was trivial (Scheme 4). Similar to **1**, the QM-mediated substitution was performed on 2-hydroxybenzyl alcohol, followed by a Mannich reaction, resulting in QMP therapeutic **4** in moderate yield.

Next, using SP134 as an inspiration,^31^ it was hypothesized that extending the distance between the QMP ring and the imidazole moiety could allow for alternate binding poses which place the imidazole in the active site with the QMP ring as a peripheral binding element. To extend the chain between the QMP ring and the imidazole, an alternative synthesis route was performed starting from 4-methoxyphenylacetic acid and its extended chain counterparts (Scheme 5). Starting from the acid, a boron trifluoride-assisted reduction resulted in the subsequent alcohols in excellent yields. The alcohol was then tosylated to install a leaving group which was then displaced in an S_N_2 reaction by imidazole. The *N*-heterocyclic product was then subjected to a HBr-mediated demethylation, resulting in the free phenol, which was then subjected to a Mannich reaction to yield the QMP therapeutics **5**–**7** (Scheme 5).

**Scheme 5:**
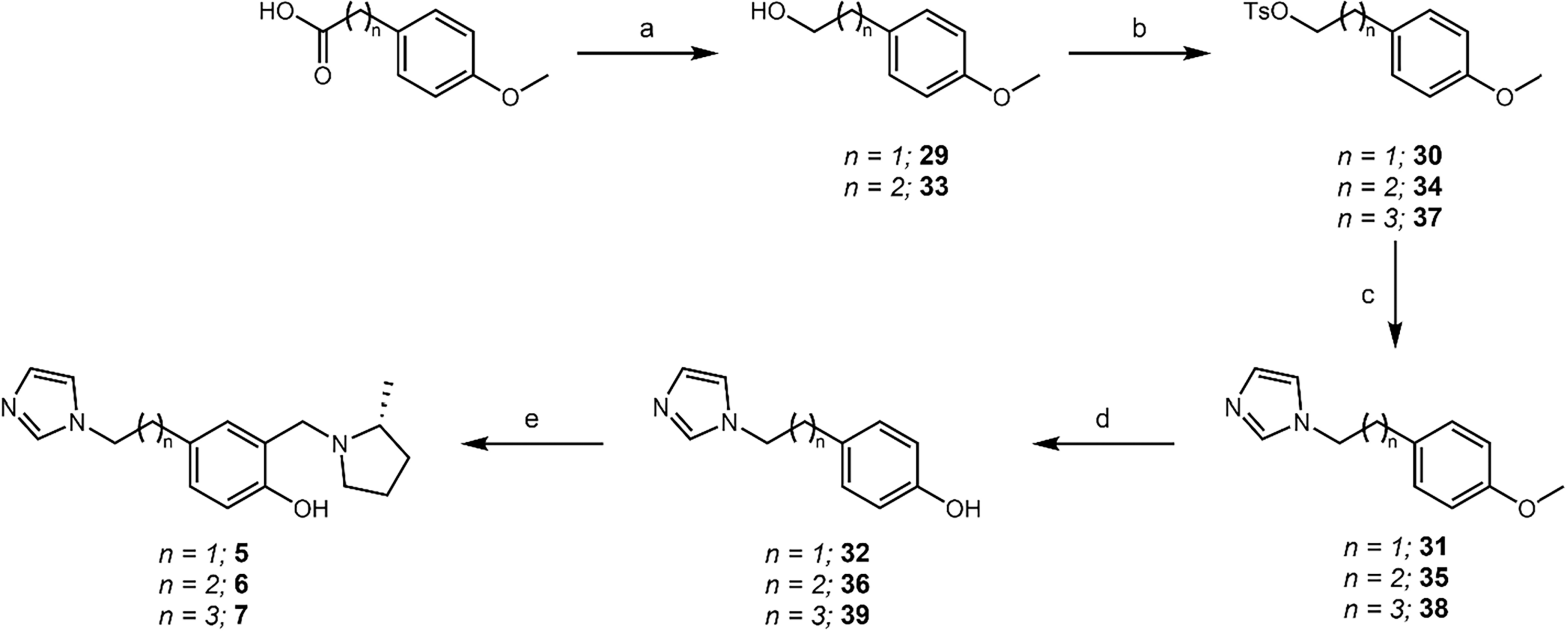
Synthesis of **5**–**7** ^a^

a (a) BF_3_·Et_2_O, NaBH_4_, THF, rt, 75–94%; (b) TsCl, NEt_3_, DCM, rt, 67–76%; (c) imidazole, Cs_2_CO_3_, MeCN, 70 °C, 35–51%; (e) HBr, neat, reflux, 50%-quantitative; (f) (*R*)-2-methylpyrrolidine (3 equiv), paraformaldehyde (3 equiv), toluene, EtOH, 120 °C, 30–52%.

Due to the high cost of the analogous carboxylic acid and alcohol starting material, the 5-carbon extended chain between the imidazole and QMP ring in the *para* position (**8**) was synthesized *via* a different route (Scheme 6). Benzyl protection of 4-iodophenol was accomplished using benzyl bromide in acetone and in high yield. The aryl iodide was then subjected to a Sonogashira cross coupling reaction^39^ to 4-pentyn-1-ol. The alcohol was then converted to a bromo derivative through an Appel reaction,^40^ followed by an S_N_2 reaction to install the peripheral imidazole ring. The alkyne was reduced, and the benzyl protecting group was removed concomitantly to generate **44**. A Mannich reaction provided the QMP therapeutic **8** (Scheme 6). Indeed, this route is useful for the longer chains but is poor yielding (6% overall); thus, this approach was only used when necessary.

**Scheme 6:**
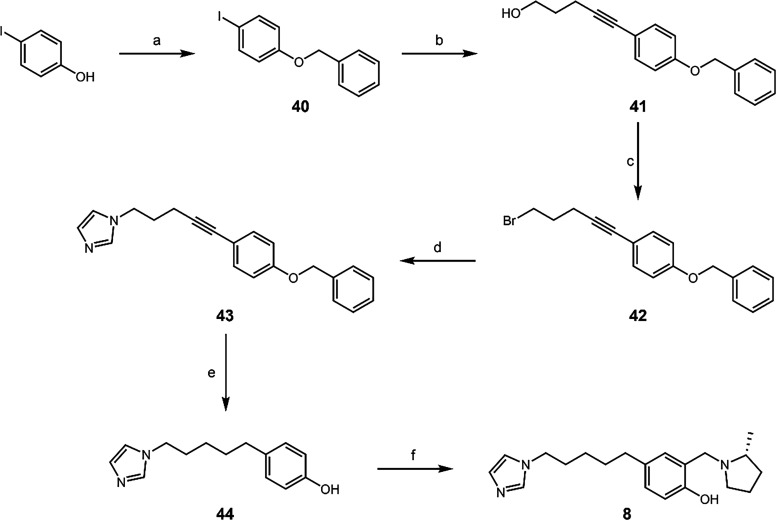
Synthesis of **8** ^a^

a (a) BnBr, K_2_CO_3_, acetone, reflux, 88%; (b) 4-pentyn-1-ol, Pd­(PPh_3_)_4_, CuI, NEt_3_, DCM, 40 °C, 75%; (c) CBr_4_, PPh_3_, DCM, rt, 69%; (d) imidazole, Cs_2_CO_3_, TBAI, MeCN, 80 °C, 85%; (e) H_2_, Pd­(OH)_2_, MeOH, EtOAc, 50 °C, 71%; (f) (*R*)-2-methylpyrrolidine (1.5 equiv), paraformaldehyde (1.5 equiv), toluene, EtOH, 120 °C, 22%.

Analogous to the extensions in the *para* position, the same reaction scheme was used for the synthesis of the extensions for the *meta* position regioisomer. The synthetic route started from 3-methoxyphenylacetic acid, and the use of analogous reactions for the extended chain counterparts resulted in QMP therapeutics **9** and **10** (Scheme 7).

**Scheme 7:**
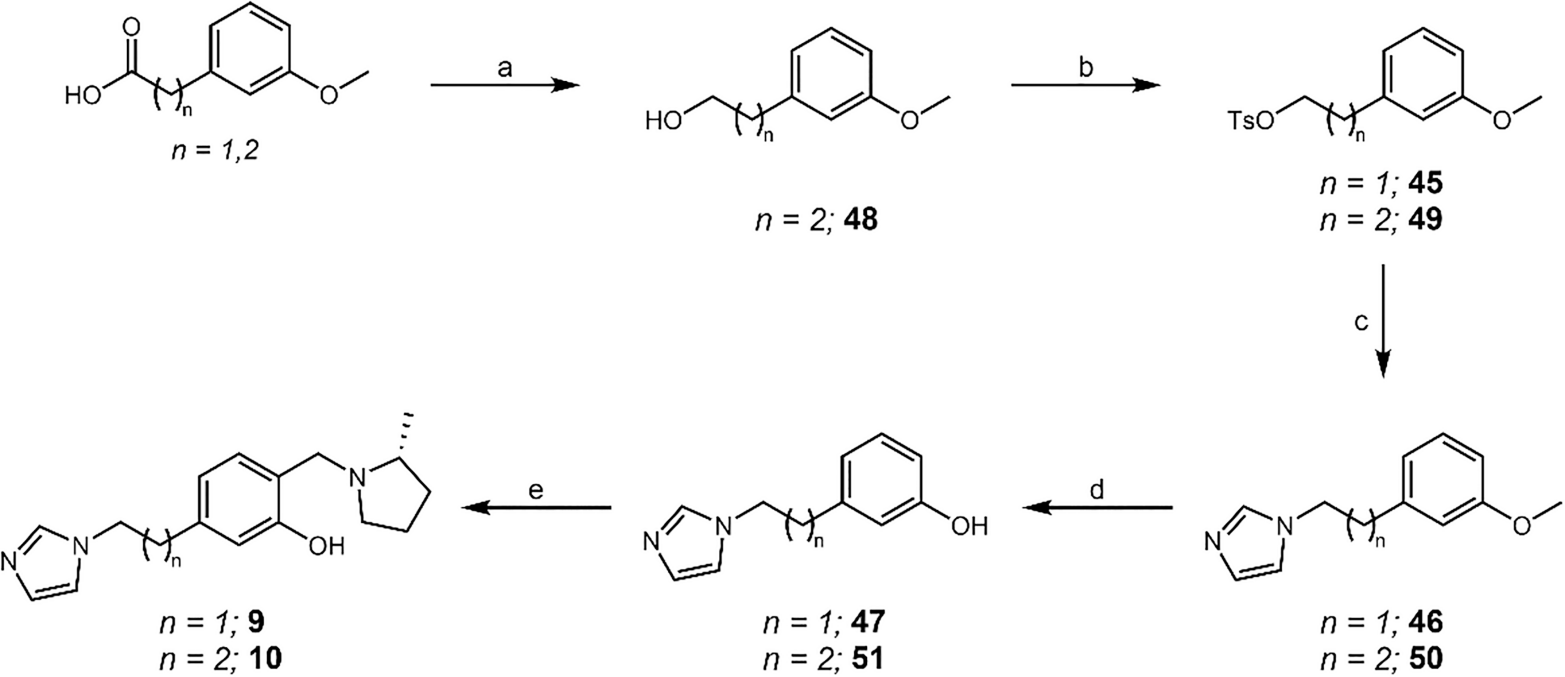
Synthesis of **9** and **10** ^a^

a (a) BF_3_·Et_2_O, NaBH_4_, THF, rt, quantitative; (b) TsCl, NEt_3_, DCM, rt, 50–77%; (c) imidazole, Cs_2_CO_3_, TBAI, MeCN, 80 °C, 40–76%; (d) HBr, neat, reflux, 85–86%; (e) (*R*)-2-methylpyrrolidine (1.5 equiv), paraformaldehyde (1.5 equiv), toluene, EtOH, 120 °C, 38–51%.

Once again, the route shown in Scheme 6 was utilized to synthesize QMP therapeutics **11** and **12** (Scheme 8), as these carboxylic acid and alcohol starting materials were also far more expensive than the equivalent 3-iodophenol. Further extensions past five carbon atoms in the chain between the QMP and imidazole were deemed unnecessary as there was a vast decrease in efficacy at the longer lengths, as noted below.

**Scheme 8:**
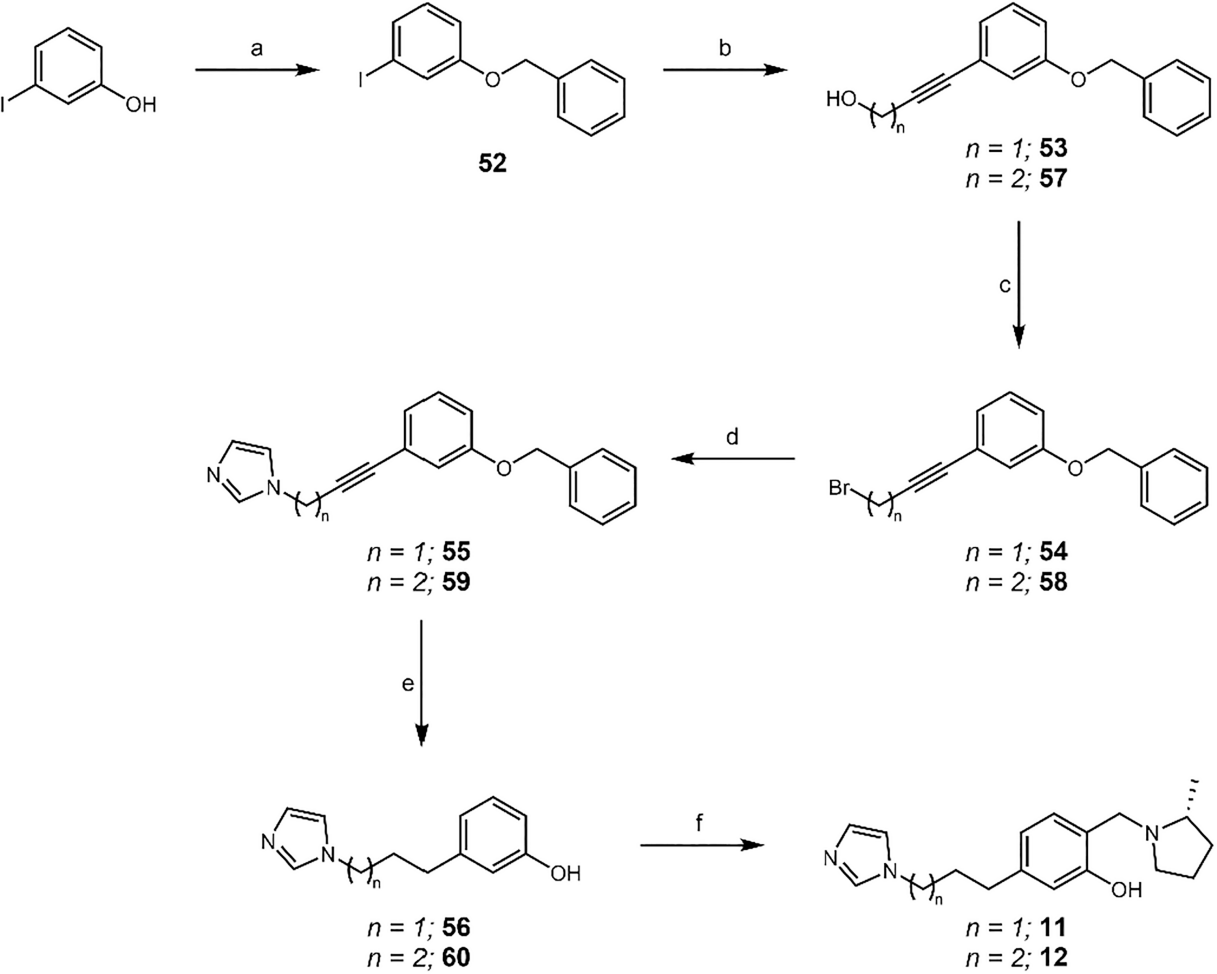
Synthesis of **11** and **12** ^a^

a (a) BnBr, K_2_CO_3_, acetone, reflux, quantitative; (b) *n* = 1:3-butyn-1-ol and *n* = 2:4-pentyn-1-ol; Pd­(PPh_3_)_4_, CuI, NEt_3_, DCM, 40 °C, 40–73%; (c) CBr_4_, PPh_3_, DCM, rt, 50–62%; (d) imidazole, Cs_2_CO_3_, TBAI, MeCN, 80 °C, 22–83%; (e) H_2_, Pd­(OH)_2_, MeOH, EtOAc, 50 °C, 91–94%; (f) (*R*)-2-methylpyrrolidine (1.5 equiv), paraformaldehyde (1.5 equiv), toluene, EtOH, 120 °C, 37–57%.

While imidazole was the primary target, we also wanted to determine if binding interactions and steric effects influenced the biochemical efficacy. Thus, we envisioned using benzimidazole to potentially increase the binding affinity in the enzyme due to the higher density of π-π stacking interactions that could occur for **13**.^41^ Despite great utility, the QM-mediated substitution used to synthesize **1**, **2**, **4** and others failed to work when the nucleophile was benzimidazole or 2-methylimidazole. Instead, these nucleophiles were installed in a similar manner to **3** from Scheme 3, wherein a simple substitution was performed on 4-methoxybenzyl chloride (Scheme 9). Indeed, this substitution could also be used on the previously synthesized tosylates **30** and **34** to yield the corresponding carbon extensions of the benzimidazole-linked QMP therapeutics analogous to QMP therapeutics **5** and **6**. Upon HBr-mediated demethylation and a Mannich reaction, the syntheses of QMP therapeutics **13–15** were achieved in moderate yields.

**Scheme 9:**
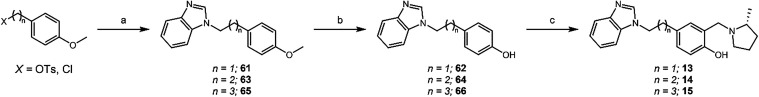
Synthesis of **13–15** ^a^

a (a) Benzimidazole, Cs_2_CO_3_, MeCN, 70 °C, 60–63%; (b) HBr, neat, reflux, 64%-quantitative; (c) (*R*)-2-methylpyrrolidine (3 equiv), paraformaldehyde (3 equiv), toluene, EtOH, 120 °C, 35–52%.

The route utilized for framework **3** (Scheme 3) was also used to synthesize QMP therapeutic **16**, by exchanging the imidazole for benzimidazole to alter the peripheral *N*-heterocyclic ring. Though both routes resulted in moderate yields of the product, the demethylation is much more efficient for benzimidazole as the product crystallizes upon neutralization (Scheme 10).

**Scheme 10:**
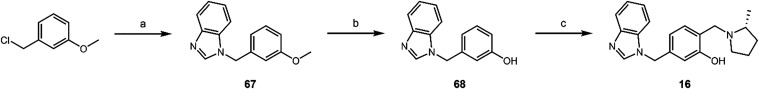
Synthesis of **16** ^a^

a (a) Benzimidazole, Cs_2_CO_3_, TBAI, MeCN, 70 °C, 89%; (b) HBr, neat, reflux, 81%; (c) (*R*)-2-methylpyrrolidine (3 equiv), paraformaldehyde (3 equiv), toluene, EtOH, 120 °C, 10%.

To further our understanding of the orientation of the *N*-heterocyclic group, the benzimidazole was linked *via* the 2-carbon position to the phenol ring of the QMP ring (**17** and **18**, Scheme 10) and the *N*-center was methylated. This was achieved through a cyclization onto 4-methoxyphenylacetic acid using phenylenediamine. From this material, the nitrogen was altered by retaining the N–H for hydrogen-bond donation, as well as by masking the nitrogen *via* methylation using methyl iodide. These separate frameworks were then subjected to a HBr-mediated demethylation and then a Mannich reaction, thus resulting in QMP therapeutics **17** and **18** in moderate yields (Scheme 11).

**Scheme 11:**
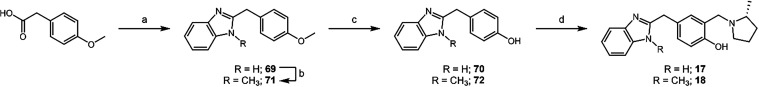
Synthesis of **17** and **18** ^a^

a (a) Phenylenediamine, HCl, reflux, 49%; (b) NaH, MeI, THF, rt, 84%; (c) HBr, neat, reflux, 98%-quantitative; (d) (*R*)-2-methylpyrrolidine (3 equiv), paraformaldehyde (3 equiv), toluene, EtOH, 120 °C, 15–33%.

Given the small steric size of imidazole, installation of 2-methylimidazole (**19**) may provide a steric handle on the imidazole, potentially revealing if the surroundings of the group are affected by steric bulk. Thus, utilizing a similar synthesis to Scheme 9, 4-methoxybenzyl chloride was substituted with 2-methylimidazole, which was then subjected to a HBr-mediated demethylation and then a Mannich reaction, resulting in QMP therapeutic **19** in moderate yield (Scheme 12).

**Scheme 12:**
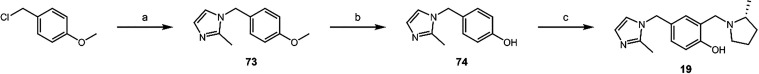
Synthesis of **19** ^a^

a (a) 2-Methylimidazole, Cs_2_CO_3_, MeCN, 70 °C, 52%; (b) HBr, neat, 120 °C, 73%; (c) (*R*)-2-methylpyrrolidine (3 equiv), paraformaldehyde (3 equiv), toluene, EtOH, 120 °C, 51%.

Despite being a different nitrogen-containing heterocycle, pyrazole was also of interest in the structure–activity relationship due to its reduced basicity relative to imidazole as well as its slight change in nitrogen orientation. Synthesis of **20** was performed in the same manner as **1a**–**1e**, only exchanging the imidazole for pyrazole in the QM-mediated substitution (Scheme 13).

**Scheme 13:**
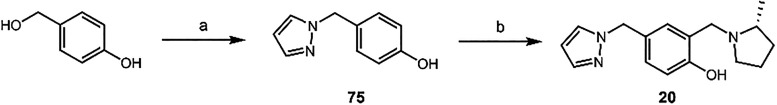
Synthesis of **20** ^a^

a (a) Pyrazole, neat 110 °C, 51%; (b) (*R*)-2-methylpyrrolidine (1.5 equiv), paraformaldehyde (1.5 equiv), toluene, EtOH, 120 °C, 30%.

Overall, a total of 24 novel QMP therapeutics with 20 unique frameworks were synthesized and evaluated by *in vitro* biochemical studies.

### Biochemical *In Vitro* Evaluations of *N*-Heterocyclic QMP Compounds (**1**–**20**)

To test our hypothesis of *in vitro* reactivation *via* *N*-heterocycle-linked QMP compounds, we began our biochemical evaluations by testing the QMP therapeutics against OEt-inhibited AChE (pH 7.5, 37 °C) as shown in Figure 4. Impressively, at 250 μM and 1 h of incubation, multiple QMP therapeutics reached nearly complete recovery of the inhibited form of the enzyme, improving on the oximes’ therapeutic efficacy.

**Figure 4:**
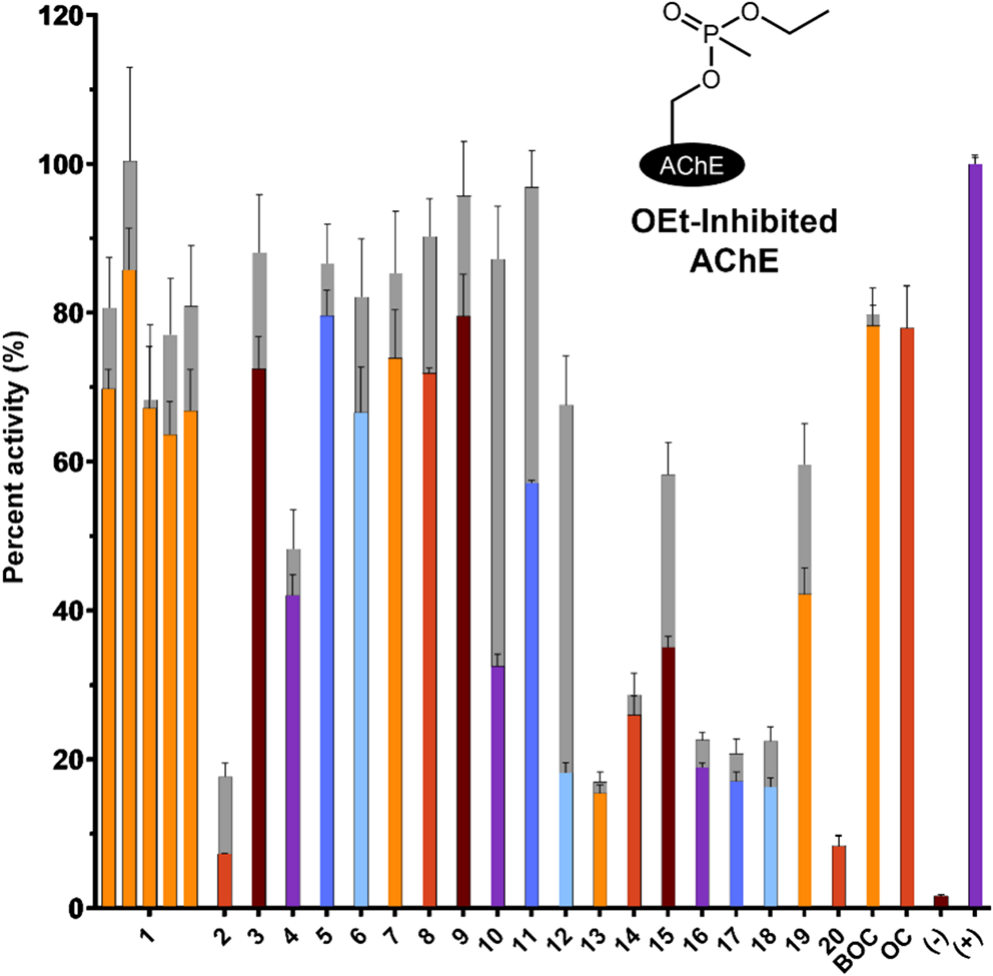
Biochemical *in vitro* recovery (pH 7.5, 37 °C) by *N*-heterocycle-linked QMP compounds of OEt-inhibited AChE. Numbered bars represent frameworks listed in Figure 3, with left to right bars in the same chain representing different amine leaving groups, along with oxime controls (OC and BOC). The graph shows the results for a concentration of 250 μM and after 1 h of incubation, followed by Ellman’s assay (with 100× dilution) to evaluate the activity of the reactivated, native AChE. Colored bars represent a direct comparison of the QMP recovery relative to a water positive control while the gray bars represent the recovery relative to an equivalent concentration of that specific compound in the positive control, which was never exposed to the OP compound, thereby accounting for any native inhibition of AChE. The negative control represents OEt-inhibited AChE that was not exposed to any subsequent therapeutic. Each measurement is shown as an average of four replicate measurements, along with an error bar that depicts one standard deviation of those replicate measurements.

Our broad-scope biochemical *in vitro* evaluations began by determining the effect of the imidazole regiochemistry and distance to the QMP ring in the reactivation of OP-inhibited AChE (250 μM, 1 h, 37 °C) (Figure 5, black cells). Due to the large number of OP-inhibited/aged forms tested against, these data were combined into heat maps which represent the percent recovery of AChE in colored cells rather than a histogram plot, as shown in Figure 4. For the data, first, unlike our previous studies, surprisingly (*R*)-2-methylpyrrolidine (**1d**) was not the most efficacious amine leaving group. Rather, **1b**, which contains diethylamine as a leaving group consistently outperforms **1d**, indicating the likelihood of binding in the active site by the amine as not being the only contributor to reactivation. Perhaps, the imidazole group is performing a role as an external base for reactivation, either as the primary reactivator, or as a second ancillary reactivator. Interestingly, QMP compounds (**1b** and **1d**) demonstrate a wider range of reactivation than we have seen before. While some previous QMPs have shown some difficulty in reactivating some specific OP-inhibited forms, such as OCy-, EP- and DFP-inhibited forms of AChE, these imidazolyl QMPs demonstrate recovery of these forms. Impressively, there is little difference between oxime therapeutics (indicated by BOC and OC) and these QMPs for reactivation of OP-inhibited AChE at this 250 μM concentration and 1 h of incubation.

**Figure 5:**
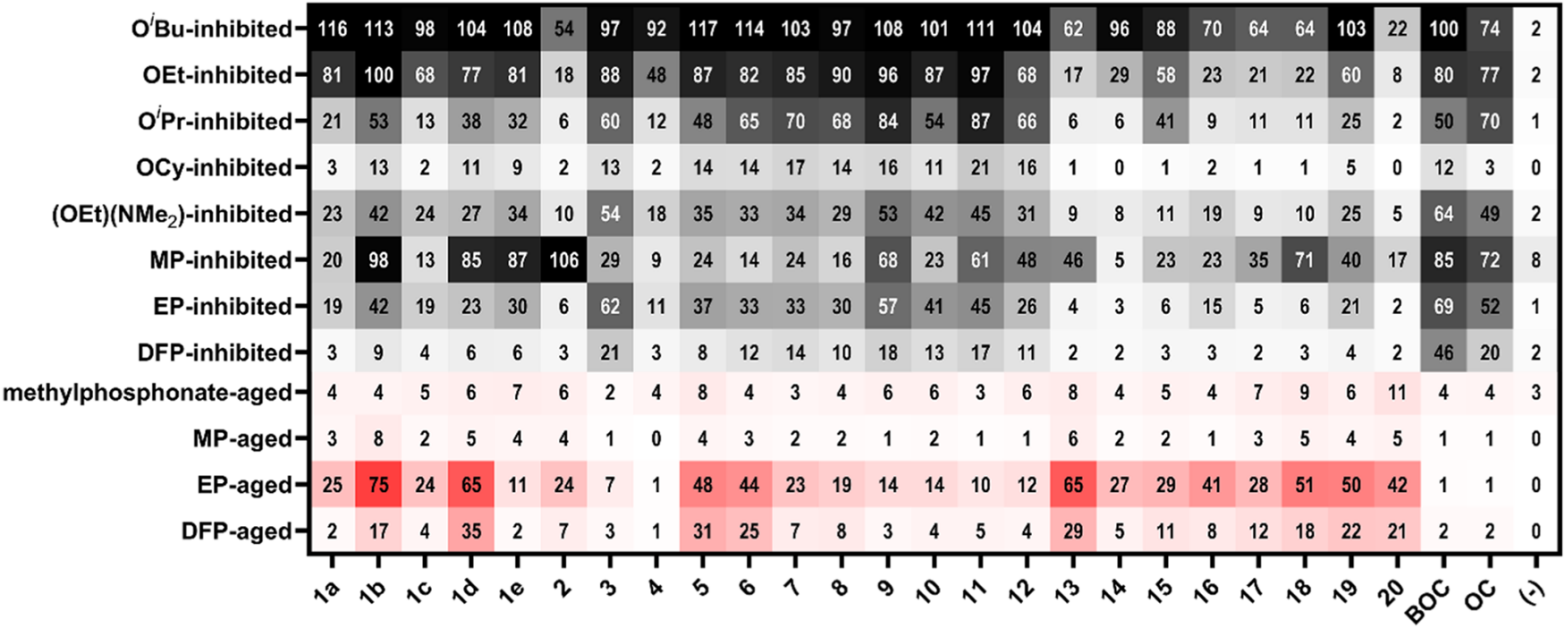
Biochemical *in vitro* recovery (pH 7.5, 37 °C) by *N*-heterocycle-linked QMP compounds for (black) reactivation of OP-inhibited AChE after incubation for 1 h at a concentration of 250 μM, and (red) resurrection of OP-aged AChE after incubation for 24 h at a concentration of 250 μM of OP-aged AChE. Each cell of the heat map represents the percent reactivation (black) or resurrection (red) of a QMP (identified by column labels based on compounds listed in Figure 3) for a given OP-inhibited or aged form of AChE (identified by row labels listed in Table 1) relative to an equivalent concentration of that specific compound in the positive control which was never exposed to the OP compound, thereby accounting for any native inhibition of AChE. Cell color is more saturated as percent recovery increases, with 100% recovery being the darkest hue. The final Ellman’s assay was completed after a 100× dilution, thus the final Ellman’s concentration was 2.5 μM. Each measurement is shown as an average of four replicate measurements, and all graphs with error bars are provided in Figures S2–S12.

Furthermore, to ensure this is the case, we chose to test **21** (Scheme 1) for reactivation efficacy as this compound was formed prior to the Mannich and thus only contained the imidazole group as a reactivator and not the Mannich phenol as well. Indeed, **21** had far less reactivation efficacy (likely due to poorer binding), but still performed some reactivation, indicating the imidazole group plays a role in reactivation (Figure S1). Oddly, exchanging the phenol in **1d** for a pyridine (**2**) results in much poorer efficacy for all OP-inhibited forms except MP-inhibited AChE, wherein **2** is the most efficacious QMP.

Interestingly, when the imidazole ring is linked *via* the 5-position (*meta*) of the phenol, there is a vast difference in reactivation. QMP therapeutic **3** demonstrates worse reactivation of MP-inhibited AChE but boasts much higher reactivation of (OEt)­(NMe_2_)-, EP- and DFP-inhibited AChE. Furthermore, the phosphoramidate- and phosphate-inhibited forms of AChE (Table 1) are very difficult to reactivate, making the observed efficacy more impressive. Moreover, extension of this chain in the *meta* position results in what seems to be the best reactivation found—with high recovery for therapeutics **9** and **11**. Indeed, **11** is the only therapeutic (including oximes) that performs significant reactivation (>20%) of all but one of the OP-inhibited forms of AChE, wherein reactivation of DFP-inhibited AChE reaches 17%. However, given **3** performs sufficient reactivation of DFP-inhibited AChE (21%), and diethylamine as the amine leaving group yielded higher reactivation efficacy (often near 1.5 times better), it is likely further optimization can result in a framework that can perform significant reactivation in all OP-inhibited forms of AChE—aspects that will be evaluated in due course.

Despite the extensions in the *meta*-position performing far better than the *para*-position, it seems extensions in the *para*-position provide little additional benefit. Therapeutics **5**, **6**, **7** and **8** show less reactivation than **1d** against (OEt)­(NMe_2_)-, MP- and EP-inhibited AChE, but show better activity against O^*i*^Pr-, OCy- and DFP-inhibited AChE. However, it is of note that the extended *para*-substituted therapeutic **7**, which is analogous to **11**, outperforms the other chain length extensions in the 4-position (*para*). Indeed, this 2- or 4-carbon atom linkage seems important for the reactivation as these analogues yield the best reactivation efficacy. Furthermore, investigation into the benzimidazole linkers provides no significant therapeutics for the reactivation of OP-inhibited AChE when compared to **1d**, **9** and **11**. On the other hand, substituting the imidazole group for a 2-methylimidazole group (**19**) allows the therapeutic to retain some activity, although much activity is lost for OCy- and MP-inhibited AChE. Indeed, this shows the opportunity to functionalize the imidazole ring in the future, potentially resulting in greater efficacy of the therapeutics. Lastly, it is of note that the pyrazole group in (**20**), despite its similarities to imidazole, retains little to no OP-inhibited AChE reactivation efficacy and is the least effective compound that was tested for reactivation.

Pleasantly surprised by the results for the reactivation of OP-inhibited AChE, we investigated the effect of the imidazole therapeutics on resurrection of OP-aged AChE (250 μM, 24 h, 37 °C) (Figure 5, red cells). Amazingly, at a modest concentration of 250 μM, **1b** and **1d** perform resurrection of methylphosphonate- (5%), MP- (8%), EP- (>65%), and DFP-aged (>15%) AChE. In conjunction with the reactivation results, these observations suggest that **1b** and **1d** are very promising candidates for further exploration and optimization. However, methylphosphonate-aged AChE still poses an extreme challenge as many therapeutics are very slow at recovering this form of the enzyme. Along with this, MP-aged AChE upon aging at pH 7.5 is also a challenge.

Interestingly, despite **3**, **9** and **11** performing well for reactivation, these therapeutics offer little recovery against the OP-aged forms of AChE. Instead, the opposite trend of reactivation is observed wherein the benzimidazole-containing frameworks **13** through **18** provide strong recovery of the OP-aged forms of the enzyme, with **13** and **18** having some of the highest recoveries (∼10% at 250 μM) of methylphosphonate-aged AChE. It is also of note that comparing **17** and **18** against all of the screenings shows a clear preference for the lack of the N–H hydrogen-bond donation, instead the data point toward retaining the *N*-methyl group on the nitrogen for higher efficacy. Furthermore, despite **20** being the least effective reactivator of OP-inhibited AChE, as a resurrector, **20** boasts an impressive 11% recovery of methylphosphonate-aged AChE, outperforming our previous work as the highest recorded recovery of this aged form for this concentration and time.^30^ Thus, although a poor reactivator, the pyrazole group in **20** shows promise in resurrection, and as such will be explored in the future. Furthermore, a detailed comparison of Figure 5 suggests that **5** and **6** as well as **19** are of interest as these QMP therapeutics show recovery of various OP-inhibited forms of AChE as well as good resurrection of OP-aged AChE. Again, **19** shows much room for improvement as the imidazole ring can be easily functionalized in the 2-position to alter electronic and steric effects for optimized therapeutics.

These data indicate an interesting trend in which the 4-position (*para*) shows overall better broad-scope efficacy in both reactivation of OP-inhibited and resurrection of OP-aged AChE while the 5-position shows overall better reactivation, while not being able to perform resurrection. We posit this to be an effect of the reactive distance from the imidazole to the phosphylated serine residue. When in the 5-position (*meta*), the imidazole group is further from the amine leaving group; hence, upon realkylation in the hypothesized resurrection mechanism (Figure 1), such *meta* therapeutics would be less able to assist in the further reactivation step, while the imidazole linked in the 4-position would provide a much closer reactive distance. Moreover, this aligns with **20** wherein the pyrazole group should perform better resurrection of methylphosphonate-aged AChE as the reactive nitrogen would be one atom closer to the aminomethyl carbon. However, despite this, it is unclear why the benzimidazole framework performs such better resurrection of OP-aged AChE in comparison to their weak reactivation efficacy.

To further our understanding of the broad-scope nature of these compounds, we performed *in vitro* reactivation studies of OP-inhibited BChE (250 μM, 1 h, 37 °C), as shown in Figure 6. Once again, **1b** is the best performing reactivator of OP-inhibited BChE, showing reactivation against all inhibited forms that were tested. Oddly, **1b** shows a near opposite trend to that of the oxime reactivators. Specifically, the OP-inhibited structures for which the oximes performed best were much less effective for **1b**, but the OP-inhibited BChE structures for which **1b** performed well were then less effective for the oximes. Of importance is O^*i*^Pr-, OCy- and EP-inhibited BChE as **1b** shows reasonable reactivation efficacy, a trait we have not seen from our previous reactivators. However, although **1b** is the best broad-scope reactivator, its efficacy against many OP-inhibited BChE structures is not as good as other therapeutics shown. For example, **18** demonstrates the highest efficacy against OCy-inhibited BChE, reaching 37% native enzyme recovery, much greater than that of the oximes at 9% recovery. Indeed, the benzimidazole containing groups demonstrate much greater reactivation efficacy in OP-inhibited BChE, likely due to the larger active site size allowing for the size of the benzimidazole aryl group. This is demonstrated well with **16**, which shows the highest reactivation efficacy against O^*i*^Pr-inhibited BChE, despite not being as impressive as the oximes. Another important reactivator is **11**, which performed the best for OP-inhibited AChE, but also has reasonable reactivation efficacy in OP-inhibited BChE. Overall, these data show that these imidazole containing compounds may require more optimization of the benzimidazole periphery to allow for more impressive BChE reactivation.

**Figure 6:**
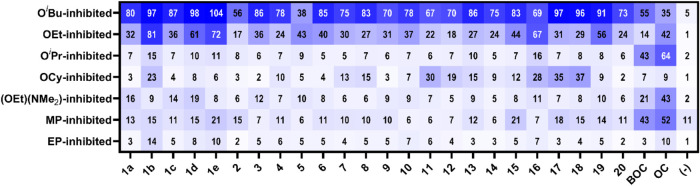
*In vitro* reactivation by imidazolyl QMP compounds after incubation for 1 h at a concentration of 250 μM (pH 7.5, 37 °C) with OP-inhibited BChE. Each cell of the heat map represents the percent reactivation of a QMP (identified by column labels based on compounds listed in Figure 3) for a given OP-inhibited form of BChE (identified by row labels listed in Table 1) relative to an equivalent concentration of that specific compound in the positive control which was never exposed to the OP compound, thereby accounting for any native inhibition of BChE. Cell color is more saturated as percent recovery increases, with 100% recovery being the darkest hue. Each measurement is shown as an average of four replicate measurements, and all graphs with error bars are shown in Figures S13–S22.

When these compounds were tested against OP-aged BChE (250 μM, 24 h, 37 °C), no detectable resurrection was observed (Figures S20–S22). Sadly, resurrection of OP-aged BChE is still elusive and far more difficult than that of OP-aged AChE.

## Conclusions

The attachment of imidazole as a pendant reactivation moiety to our QMP dual function therapeutics was successful in improving the scope of reactivation and resurrection of OP-inhibited/aged cholinesterases. Indeed, for every OP-inhibited and OP-aged AChE structure, at least one of the synthesized QMP compounds performed sufficient reactivation or resurrection. Of importance was the regiochemistry in which the imidazole was installed, as regiochemistry had a substantial effect on therapeutic efficacy. The 4-position (**1d**) indicated a balance of reactivation and resurrection efficacy while the 5-position (**3**) showed excellent reactivation efficacy while lacking resurrection efficacy. Meanwhile, the 6-position (**4**) shows little to no efficacy in both processes. Interestingly, extensions in the 4- and 5-position (**5**–**12**) showed increases to some efficacies and decreases to others. Exchanging the imidazole moiety for benzimidazole (**13**–**18**) often resulted in decreased reactivation efficacy, despite increasing resurrection efficacy. Furthermore, the exchange of the imidazole group for a pyrazole group (**20**) resulted in complete loss of reactivation efficacy, but surprisingly increased resurrection of methylphosphonate-aged AChE to levels not seen previously at this concentration. Indeed, the ease of access to **1b** and **20** cannot be understated as these therapeutics make fantastic control compounds for identifying reactivation or resurrection of OP-inhibited/aged AChE.

Of course, these therapeutics also require optimization in BChE as well, as these fail to perform well in many of the OP-inhibited forms of the enzyme and yield no resurrection of OP-aged BChE. Overall, this study shows imidazole groups may be the key for further optimization of future therapeutics for OP exposure. The inclusion of an imidazole group increases the scope of efficacy for reactivation and resurrection of OP-inhibited/aged cholinesterases, alongside providing an additional handle for reactivation, which further increases efficacy for reactivation of OP-inhibited cholinesterases.

Of the 20 compounds in this *N*-heterocyclic chemical library, specific QMP derivatives, such as **1b**, **1d**, **5** and **6**, provide broad-scope reactivation of multiple OP-inhibited AChE/BChE as well as resurrection of OP-aged AChE—a feature that is extremely important when medical professionals who are treating an OP exposure may not know exactly which specific OP toxicant was involved. Conversely, other QMP compounds (**1b**, **1d**, **3**, **9**, **11** and **20**) from this modest library, though less effective as a broad-scope therapeutic, provide excellent conversion in one category or another.

## Methods

### Chemistry

Proton and carbon nuclear magnetic resonance (NMR) spectra were recorded in CDCl_3_ or DMSO-*d*_6_ using a Bruker Avance 400 MHz (5 mm BBFO probe, temperature: 300 K) instrument or a Bruker Avance Neo 400 MHz (prodigy BBO cryoprobe, temperature: 298 K). ^1^H NMR spectra were recorded at 400 MHz, and chemical shifts are referenced to CDCl_3_ (7.26 ppm) or (CD_3_)_2_SO (2.50 ppm). ^13^C NMR spectra were recorded at 100 MHz, and the ^13^C chemical shifts are referenced to CDCl_3_ (77.00 ppm) or (CD_3_)_2_SO (39.50 ppm). High-resolution mass spectrometric studies with electrospray ionization (ESI-HRMS) were completed on a Bruker Impact II qTOF instrument or an Orbitrap Exploris MX-ESI instrument with Vanquish Flex UHPLC equipped with a C18 guard column *via* direct infusion. The samples were dissolved in methanol. High-performance liquid chromatography (HPLC) was carried out on an Agilent 1200 HPLC to confirm purity (>95%). All compounds were detected by UV–vis absorption at 254 and/or 280 nm. Thin-layer chromatography was carried out using 0.25 mm glass-supported silica gel coated 60 F_254_ plates (Silicycle). All starting materials were >95% purity and obtained from Sigma-Aldrich, Fisher, Oakwood Chemical or Ambeed.

### Biochemical Evaluations

#### AChE and BChE Reactivation and Resurrection

Reactivation and resurrection of recombinant huAChE^42^ expressed from a HEK293 cell line provided by Zoran Radic (University of California, San Diego) and huBChE was performed in the same manner as our previous works.^22^ However, to prepare the OP-aged form that is expected from the pesticide ethyl paraoxon, ethoprophos was used to prepare the OEt-phosphylated oxyanion aged form (EP-aged) in AChE, as shown in Table 1. In addition, for this study, the QMP replicates were added with a final concentration of 250 μM, and the plate was placed in an incubator at 37 °C for 1 h for reactivation or 24 h for resurrection. An oxime control (OC), 2-[(hydroxyimino)­methyl]-1-methylpyridin-1-ium chloride, and *bis*-oxime control (BOC), 1,1′-(oxidimethylene)*bis*(pyridinium-4-carbaldoxime) dichloride, were included for comparison to previous oxime literature reports. Further information can be found in the Supporting Information.

## Supplementary Material



## Notes

Synthetic procedures, associated ^1^H NMR, ^13^C NMR, HPLC and HRMS spectra, and complete results from biochemical *in vitro* screenings (PDF)
