# Supplementary material for: Imidazole as a Pendant Reactivation Ligand Increases Efficacy Scope for Reactivation and Resurrection of Organophosphorus-Inhibited/Aged Cholinesterases by Quinone Methide Precursors
Source: ACS Chem Neurosci. 2026 Feb 19;17(5):962–75. doi: 10.1021/acschemneuro.5c00631 (PMC12964352; doi:10.1021/acschemneuro.5c00631)

Imidazole as a Pendant Reactivation Ligand Increases Efficacy Scope for Reactivation and Resurrection of Organophosphorus-Inhibited/Aged Cholinesterases by Quinone Methide Precursors

Alex R. Lovins[†], Kevin A. Miller[†], Rose K. Homoelle[†], Hayden J. Hoover[†], Craig A. McElroy[‡], Christopher S. Callam[†], Christopher M. Hadad[†*]

[†] Department of Chemistry and Biochemistry, College of Arts and Sciences, Ohio State University, Columbus, OH 43210

[‡] InfinixBio, 1507 Chambers Road, Columbus, OH 43212

Contents

General Information..............................1

Reactivation and Resurrection Screens..............................6

Synthesis and Characterization of Final Therapeutics..............................28

Synthesis and Characterization of Intermediate Materials..............................51

Associated NMR and HPLC Spectra..............................91

We acknowledge financial support from the Joint Science and Technology Office (CB10791 MCDC1905-006).

Distribution Statement A. Cleared for public release: Distribution is unlimited.

General Information

Chemistry

Proton and carbon nuclear magnetic resonance (NMR) spectra were recorded in $CDCl_3$ or DMSO-d_6 using a Bruker Avance 400 MHz (5 mm BBFO probe, temperature: 300K) instrument or a Bruker Avance Neo 400 MHz (prodigy BBO cryoprobe, temperature: 298K). ^{1}H NMR spectra were recorded at 400 MHz, and chemical shifts are referenced to $CDCl_3$ (7.26 ppm) or $(CD_3)_2SO$ (2.50 ppm). ^{13}C NMR spectra were recorded at 100 MHz, and the ^{13}C chemical shifts are referenced to $CDCl_3$ (77.00 ppm) or $(CD_3)_2SO$ (39.50 ppm). High-resolution mass

spectrometric studies with electrospray ionization (ESI-HRMS) were completed on a Bruker Impact II qTOF instrument or an Orbitrap Exploris MX-ESI instrument with Vanquish Flex UHPLC equipped with a C18 guard column via direct infusion. The samples were dissolved in methanol. High-performance liquid chromatography (HPLC) was carried out on an Agilent 1200 HPLC to confirm purity (>95%). All compounds were detected by UV-vis absorption at 254 and/or 280 nm. Thin-layer chromatography was carried out using 0.25 mm glass-supported silica gel coated 60 F_{254} plates (Silicycle). All starting materials were >95% purity and obtained from Sigma-Aldrich, Fisher, Oakwood Chemical or Ambeed. Human AChE used for the biochemical evaluations was purified from recombinant expression in a HEK293 cell line provided by Dr. Zoran Radic (University of California, San Diego). BChE used for the biochemical evaluations was obtained from human plasma; specifically, human plasma-derived BChE, as kindly provided by Dr. Oksana Lockridge (University of Nebraska), was used for all *in vitro* BChE screenings. A BioTek Synergy H1 plate reader was used for all absorbance measurements to analyze enzyme activity. All reagents for the biochemical *in vitro* assays, such as BSA, DTNB, ATC and BTC, were obtained from Fisher.

AChE Reactivation and Resurrection

Recombinant human AChE[1] was expressed from a HEK293 cell line generously provided by Dr. Zoran Radic (University of California, San Diego), and this *in vitro* source was used for all AChE biochemical evaluations. For reactivation, AChE at an approximate concentration of 200 U/mL in phosphate buffer at pH 7.5 with 1% BSA was inhibited by the addition of an excess of the desired OP compound (in acetonitrile), as shown in **Table 1**, to a final concentration of 200

μM. A positive control sample was prepared in tandem but was exposed to an equivalent amount of acetonitrile rather than the OP compound in acetonitrile. After 15 minutes of inhibition, excess OP and any byproducts were removed by filtration using 0.5 mL Amicon 30 kD cutoff spin filters. AChE was diluted 188-fold into 200 mM phosphate buffer containing 1% BSA at pH 7.5. AChE was then transferred to a 384-well plate before adding QMP compounds in replicates of 4 to a final concentration of 100 μM. An oxime control (OC), 2-[(hydroxyimino)methyl]-1-methylpyridin-1-ium chloride, and a bisoxime control (BOC), 1,1′-(Oxydimethylene)bis(pyridinium-4-carbaldoxime) dichloride, were also included. Negative and positive controls received water instead of QMP. The plate was placed in a shaking incubator at 37 ˚C for 1 hr. Following incubation, 1 μL of each sample was transferred to 94 μL of a modified Ellman's assay solution containing 80 μL of 40 mM phosphate buffer, 9 μL of 40 mM phosphate buffer with 1% BSA, and 5 μL of 20 mM 5,5'-dithiobis(nitrobenzoic acid) (DTNB, Ellman's reagent) – thus, a 100x dilution of QMP concentration prior to evaluation by Ellman's assay resulting in an approximate final AChE concentration of 0.01 U/mL. To initiate the activity test, 5 μL of 10 mM acetylthiocholine (ATC) was dispensed into the plate using a Biotek Synergy H1 plate reader. The plate was removed, covered, and physically shaken before immediately placing the plate back in the plate reader, in which the absorbance was then measured at 412 nm every 20 seconds for 5 minutes. The relative activity of each QMP well was compared to the wells of the positive control (set at 100%) to determine the amount of recovered relative activity.

For resurrection, the same process was performed except ethoprophos (**Table 1**) was used to age AChE to the same form as would be derived from ethyl paraoxon, but significantly faster. For

complete aging to occur, a fresh 10 mM stock of ethoprophos was prepared in acetonitrile and added in 1 μL increments every 30 seconds for a total of 3 μL to 122 μL of AChE at approximately 40 U/mL. The enzyme was left to incubate for 30 minutes before repeating the same process with a freshly prepared stock of ethoprophos. After an additional 30 minutes of incubation, filtration of excess OP and any byproducts was performed, and then QMP incubation was performed in the same manner as reactivation except QMP compounds were allowed to incubate with EP-aged AChE for 24 hours before testing the recovered activity of native AChE by Ellman's assay. The recovered (relative) activity of each QMP compound was then compared to the positive control (at 100%) that was never exposed to the OP compound.

BChE Reactivation and Resurrection

Human plasma-derived BChE was kindly provided by Dr. Oksana Lockridge (University of Nebraska). For reactivation studies with BChE, a sample of BChE, at approximately 1.13 mg/mL, was first inhibited with 200 μM of the given OP surrogate (**Table 1**). After 15 minutes, any excess OP was filtered away, and the solution was prepared for incubation with QMPs in the same manner as AChE reactivation or resurrection (as described above). Following incubation for the appropriate time, 1 μL of each sample was transferred to 94 μL of a modified Ellman's assay solution containing 80 μL of 40 mM phosphate buffer, 9 μL of 40 mM phosphate buffer with 1% BSA, and 5 μL of 20 mM DTNB. To initiate the activity test, 5 μL of 100 mM butyrylthiocholine (BTC) was dispensed into the plate using a Biotek Synergy H1 plate reader. Each plate was well mixed using a multichannel pipet. The absorbance was then measured at 412 nm for 5 minutes.

The activity of each QMP well was compared to the wells of the positive control (as 100%) to determine the recovered relative activity of native BChE.

Reactivation and Resurrection Screens

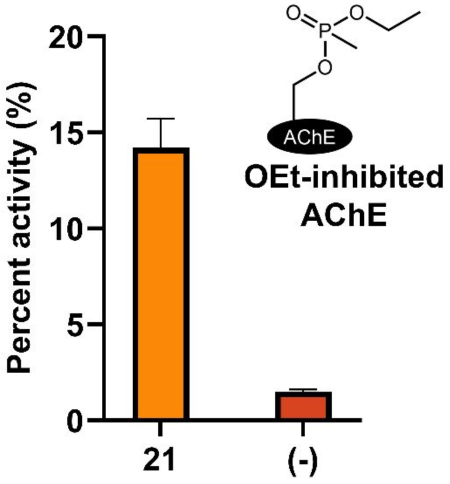

Figure S1. Biochemical *in vitro* recovery (pH 7.5, 37 °C) by compound **21** of OEt-inhibited AChE. The graph shows the results for a concentration of 5 mM and after 1 hour of incubation, followed by Ellman's assay (with 100x dilution) to evaluate the activity of the reactivated, native AChE relative to a positive control which was never exposed to the OP compound. The negative control represents OEt-inhibited AChE that was not exposed to any subsequent therapeutic. Each measurement is shown as an average of four replicate measurements, along with an error bar that depicts one standard deviation of those replicate measurements.

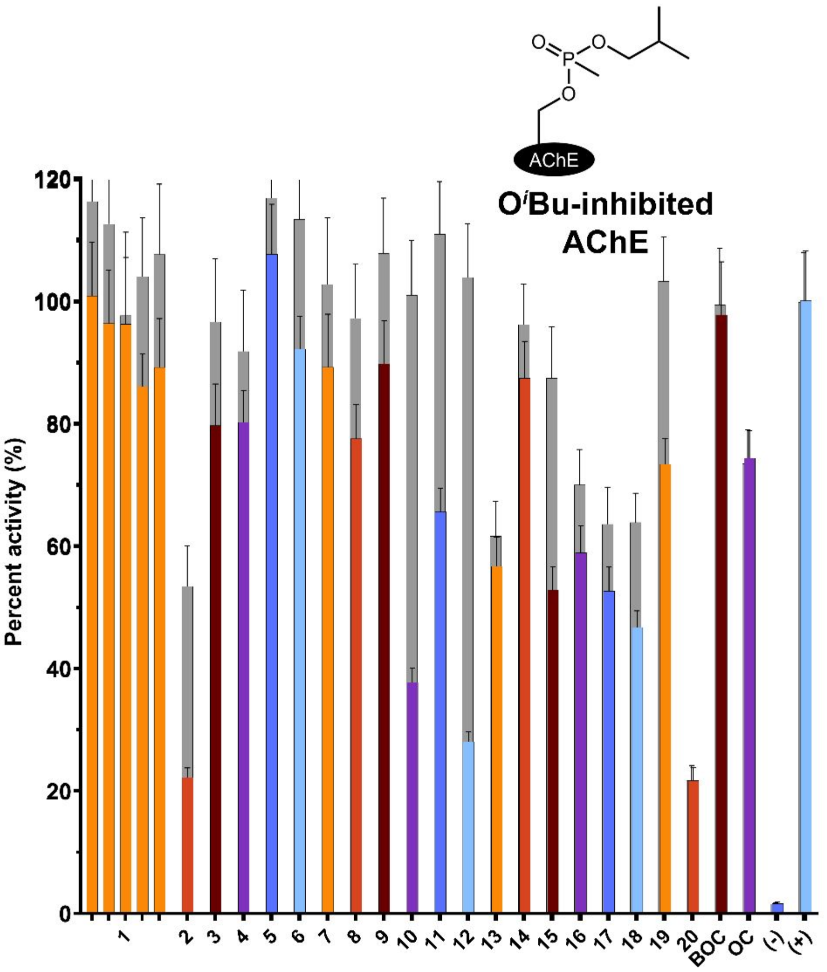

Figure S2. Biochemical *in vitro* recovery (pH 7.5, 37 °C) by *N*-heterocycle-linked QMP compounds of O^{i}Bu-inhibited AChE. Numbered bars represent frameworks listed in **Figure 3**, with left to right bars in the same chain representing different amine leaving groups, along with oxime controls (OC and BOC). The graph shows the results for a concentration of 250 μM and after 1 hour of incubation, followed by Ellman's assay (with 100x dilution) to evaluate the activity of the reactivated, native AChE. Colored bars represent a direct comparison of the QMP recovery relative to a water positive control whilst the gray bars represent the recovery relative to an equivalent concentration of that specific compound in the positive control which was never exposed to the OP compound, thereby accounting for any native inhibition of AChE. The negative control represents O^{i}Bu-inhibited AChE that was not exposed to any subsequent therapeutic. Each measurement is shown as an average of four replicate measurements, along with an error bar that depicts one standard deviation of those replicate measurements.

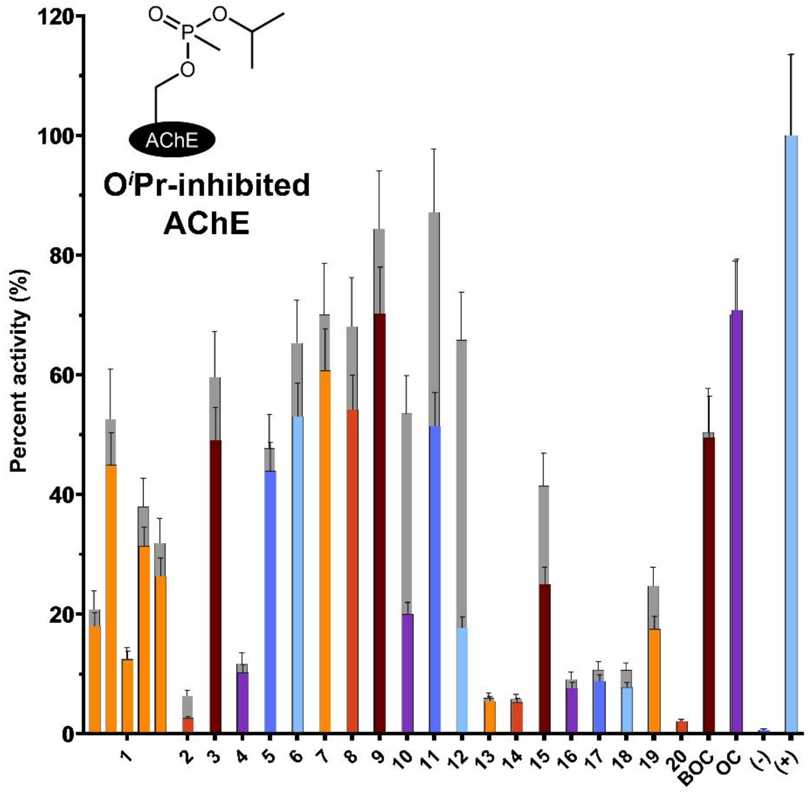

Figure S3. Biochemical *in vitro* recovery (pH 7.5, 37 °C) by *N*-heterocycle-linked QMP compounds of O^{i}Pr-inhibited AChE. Numbered bars represent frameworks listed in **Figure 3**, with left to right bars in the same chain representing different amine leaving groups, along with oxime controls (OC and BOC). The graph shows the results for a concentration of 250 μM and after 1 hour of incubation, followed by Ellman's assay (with 100x dilution) to evaluate the activity of the reactivated, native AChE. See **Figure S1** for more information.

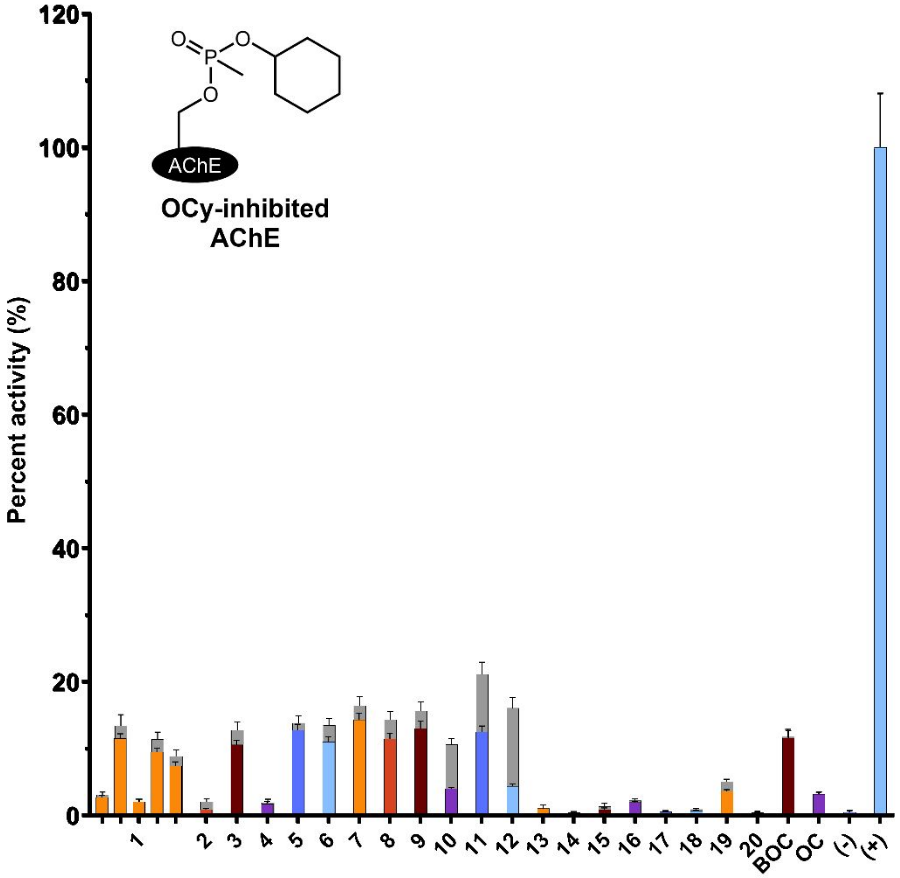

Figure S4. Biochemical *in vitro* recovery (pH 7.5, 37 °C) by *N*-heterocycle-linked QMP compounds of OCy-inhibited AChE. Numbered bars represent frameworks listed in **Figure 3**, with left to right bars in the same chain representing different amine leaving groups, along with oxime controls (OC and BOC). The graph shows the results for a concentration of 250 μM and after 1 hour of incubation, followed by Ellman's assay (with 100x dilution) to evaluate the activity of the reactivated, native AChE. See **Figure S1** for more information.

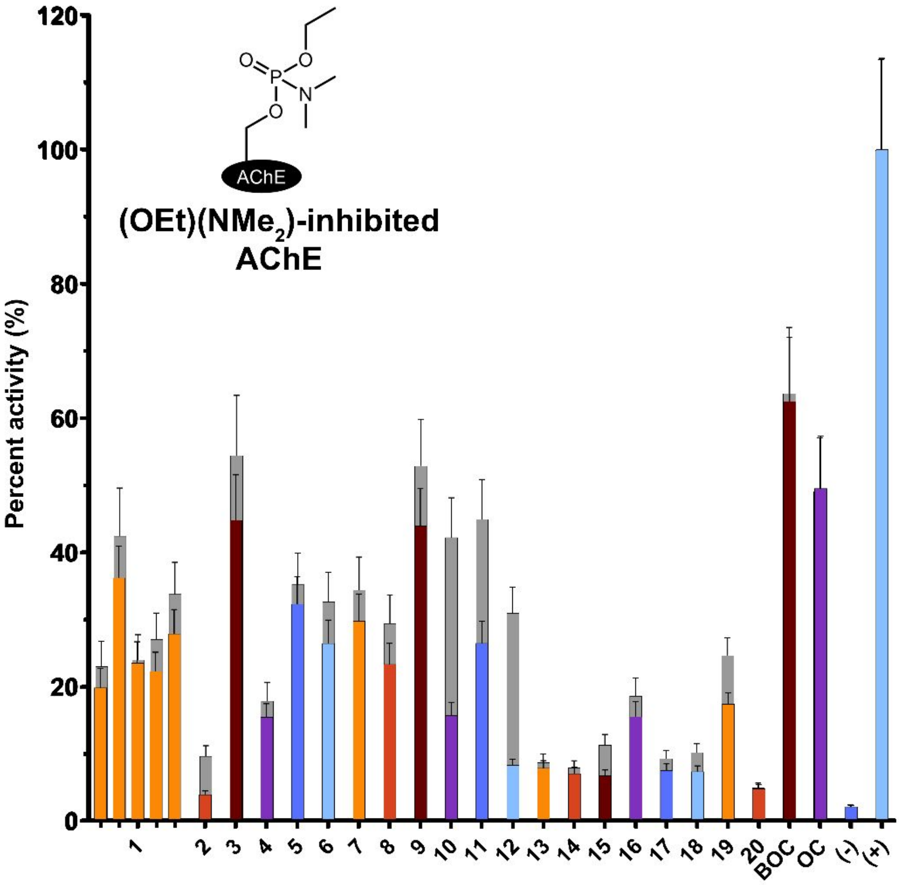

Figure S5. Biochemical *in vitro* recovery (pH 7.5, 37 °C) by *N*-heterocycle-linked QMP compounds of (OEt)(NMe_2)-inhibited AChE. Numbered bars represent frameworks listed in **Figure 3**, with left to right bars in the same chain representing different amine leaving groups, along with oxime controls (OC and BOC). The graph shows the results for a concentration of 250 µM and after 1 hour of incubation, followed by Ellman's assay (with 100x dilution) to evaluate the activity of the reactivated, native AChE. See **Figure S1** for more information.

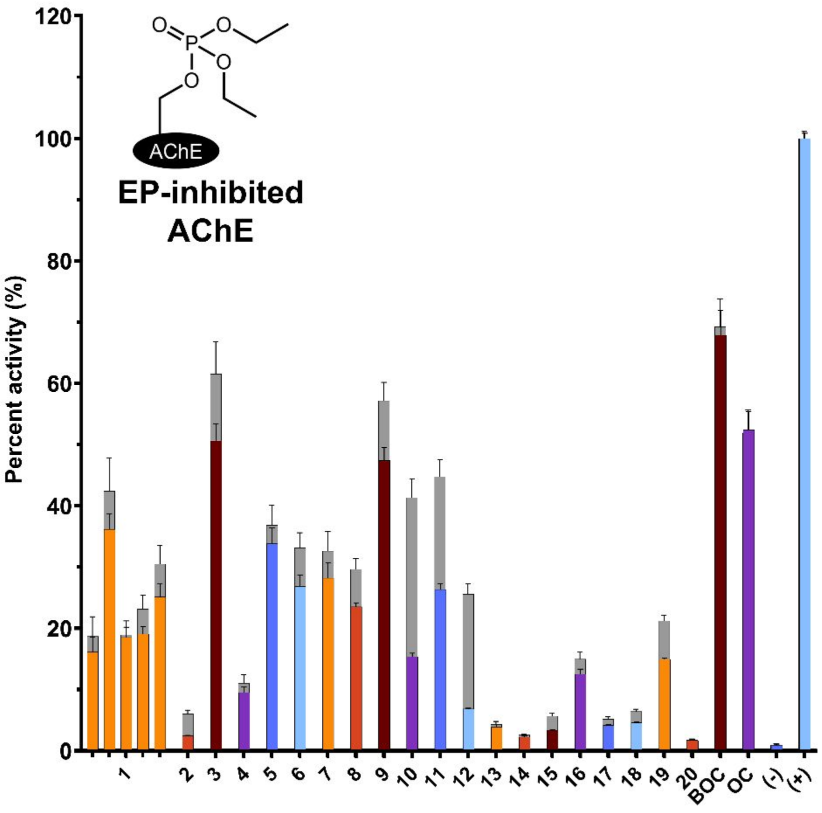

Figure S6. Biochemical *in vitro* recovery (pH 7.5, 37 °C) by *N*-heterocycle-linked QMP compounds of EP-inhibited AChE. Numbered bars represent frameworks listed in **Figure 3**, with left to right bars in the same chain representing different amine leaving groups, along with oxime controls (OC and BOC). The graph shows the results for a concentration of 250 µM and after 1 hour of incubation, followed by Ellman's assay (with 100x dilution) to evaluate the activity of the reactivated, native AChE. See **Figure S1** for more information.

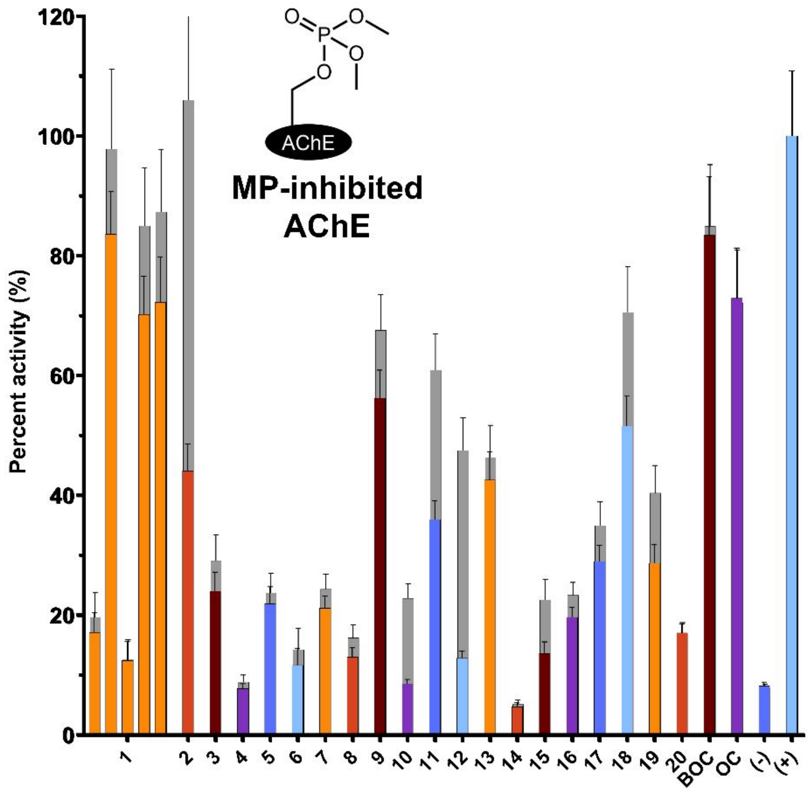

Figure S7. Biochemical *in vitro* recovery (pH 7.5, 37 °C) by *N*-heterocycle-linked QMP compounds of MP-inhibited AChE. Numbered bars represent frameworks listed in **Figure 3**, with left to right bars in the same chain representing different amine leaving groups, along with oxime controls (OC and BOC). The graph shows the results for a concentration of 250 μM and after 1 hour of incubation, followed by Ellman's assay (with 100x dilution) to evaluate the activity of the reactivated, native AChE. See **Figure S1** for more information.

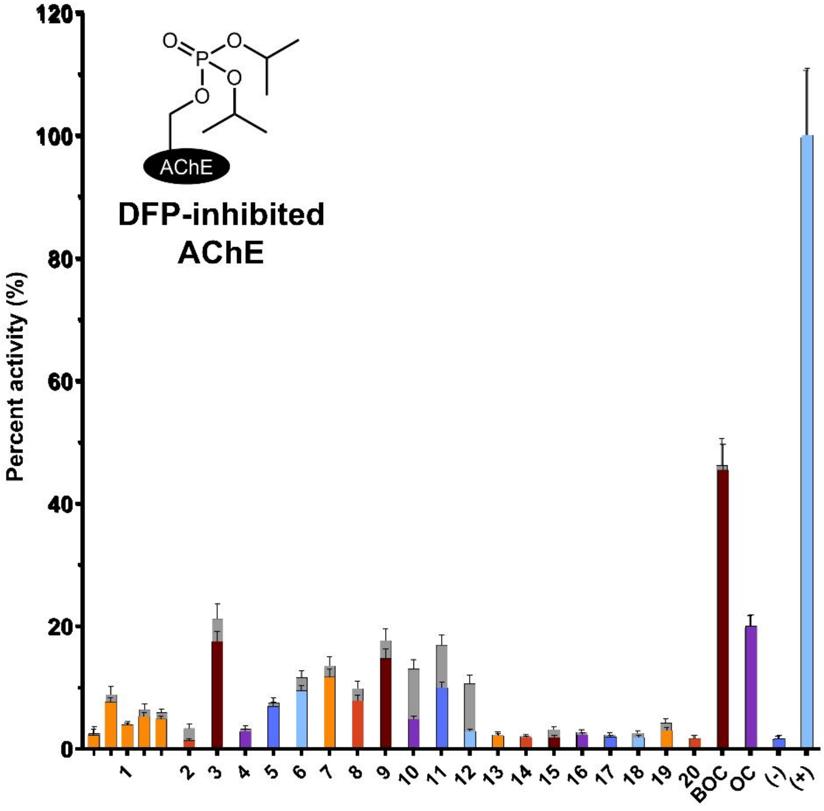

Figure S8. Biochemical *in vitro* recovery (pH 7.5, 37 °C) by *N*-heterocycle-linked QMP compounds of DFP-inhibited AChE. Numbered bars represent frameworks listed in **Figure 3**, with left to right bars in the same chain representing different amine leaving groups, along with oxime controls (OC and BOC). The graph shows the results for a concentration of 250 μM and after 1 hour of incubation, followed by Ellman's assay (with 100x dilution) to evaluate the activity of the reactivated, native AChE. See **Figure S1** for more information.

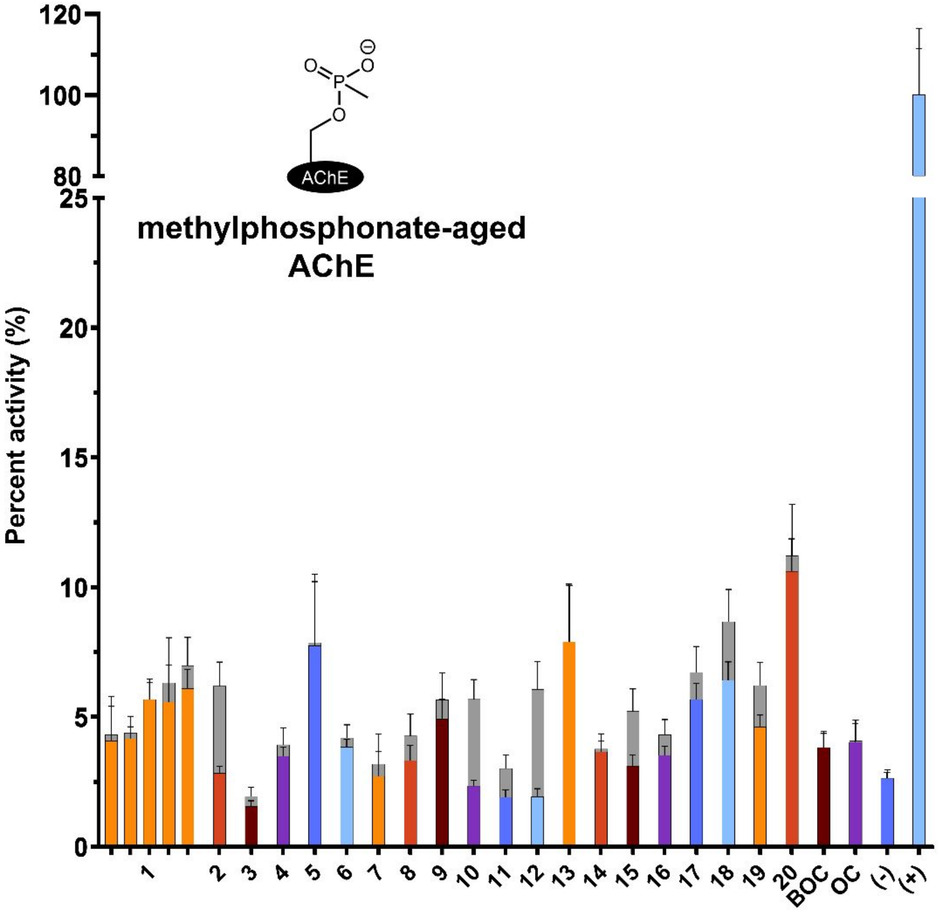

Figure S9. Biochemical *in vitro* recovery (pH 7.5, 37 °C) by *N*-heterocycle-linked QMP compounds of methylphosphonate-aged AChE. Numbered bars represent frameworks listed in **Figure 3**, with left to right bars in the same chain representing different amine leaving groups, along with oxime controls (OC and BOC). The graph shows the results for a concentration of 250 µM and after 24 hours of incubation, followed by Ellman's assay (with 100x dilution) to evaluate the activity of the resurrected, native AChE. See **Figure S1** for more information.

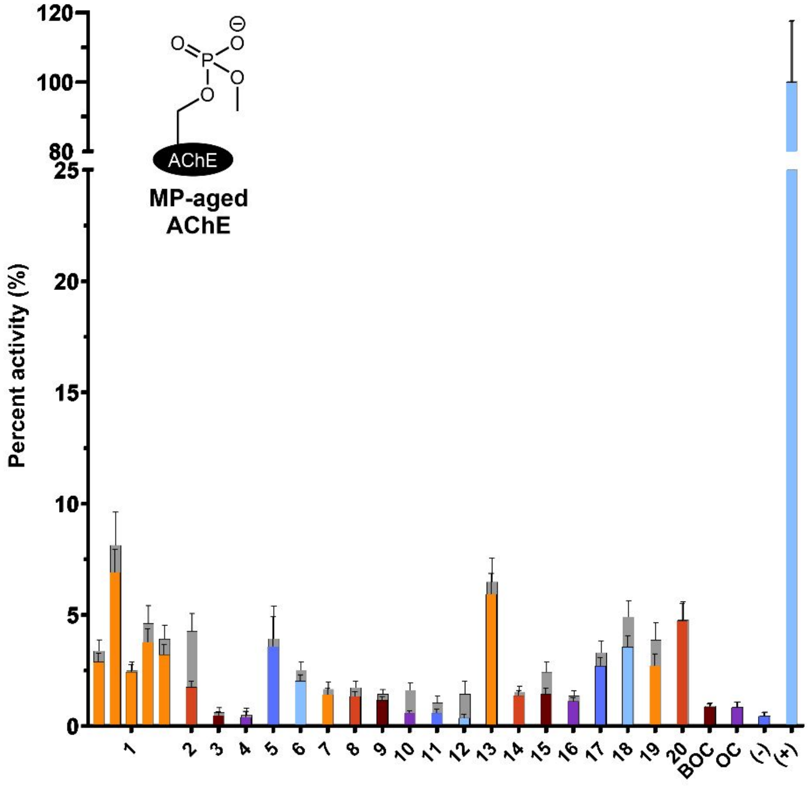

Figure S10. Biochemical *in vitro* recovery (pH 7.5, 37 °C) by *N*-heterocycle-linked QMP compounds of MP-aged AChE. Numbered bars represent frameworks listed in **Figure 3**, with left to right bars in the same chain representing different amine leaving groups, along with oxime controls (OC and BOC). The graph shows the results for a concentration of 250 μM and after 24 hours of incubation, followed by Ellman's assay (with 100x dilution) to evaluate the activity of the resurrected, native AChE. See **Figure S1** for more information.

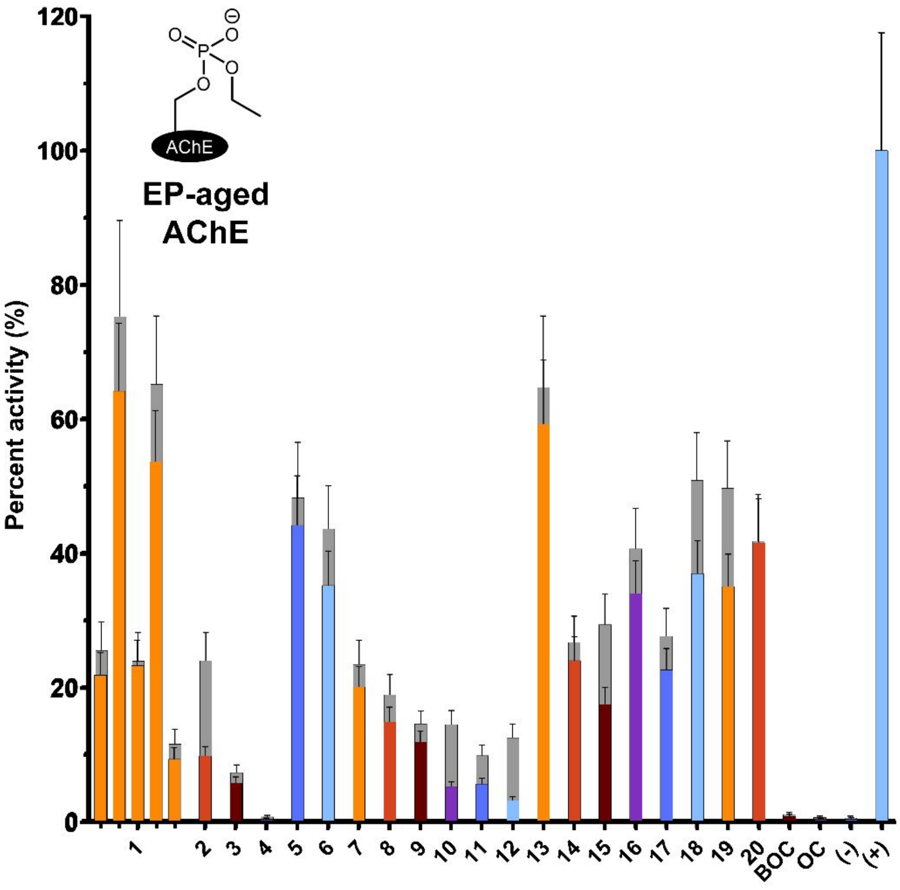

Figure S11. Biochemical *in vitro* recovery (pH 7.5, 37 °C) by *N*-heterocycle-linked QMP compounds of EP-aged AChE, achieved via aging of ethoprophos. Numbered bars represent frameworks listed in **Figure 3**, with left to right bars in the same chain representing different amine leaving groups, along with oxime controls (OC and BOC). The graph shows the results for a concentration of 250 µM and after 24 hours of incubation, followed by Ellman's assay (with 100x dilution) to evaluate the activity of the resurrected, native AChE. See **Figure S1** for more information.

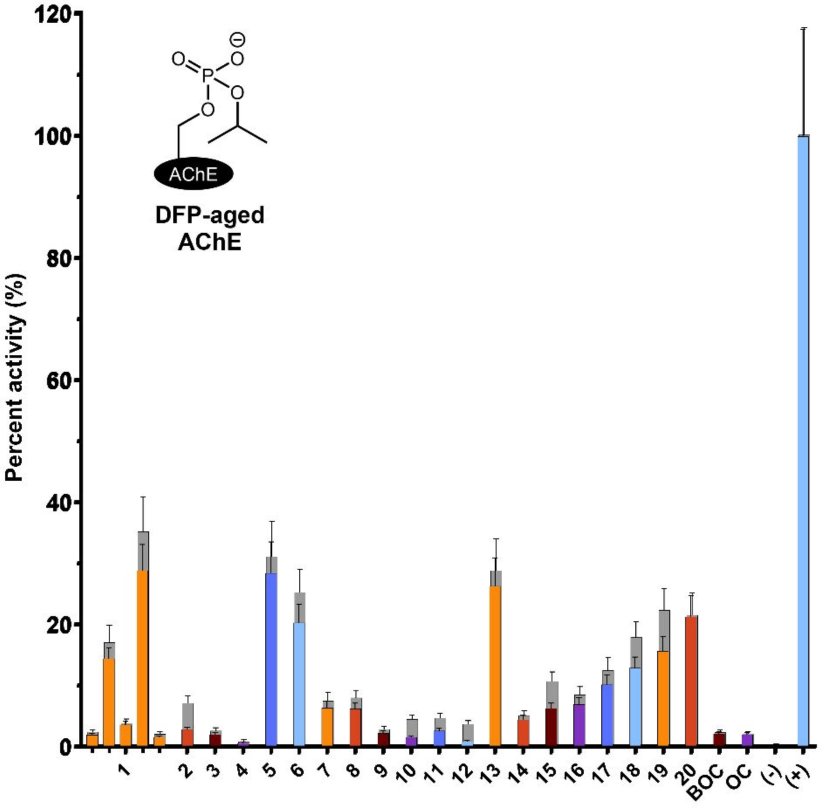

Figure S12. Biochemical *in vitro* recovery (pH 7.5, 37 °C) by *N*-heterocycle-linked QMP compounds of DFP-aged AChE. Numbered bars represent frameworks listed in **Figure 3**, with left to right bars in the same chain representing different amine leaving groups, along with oxime controls (OC and BOC). The graph shows the results for a concentration of 250 µM and after 24 hours of incubation, followed by Ellman's assay (with 100x dilution) to evaluate the activity of the resurrected, native AChE. See **Figure S1** for more information.

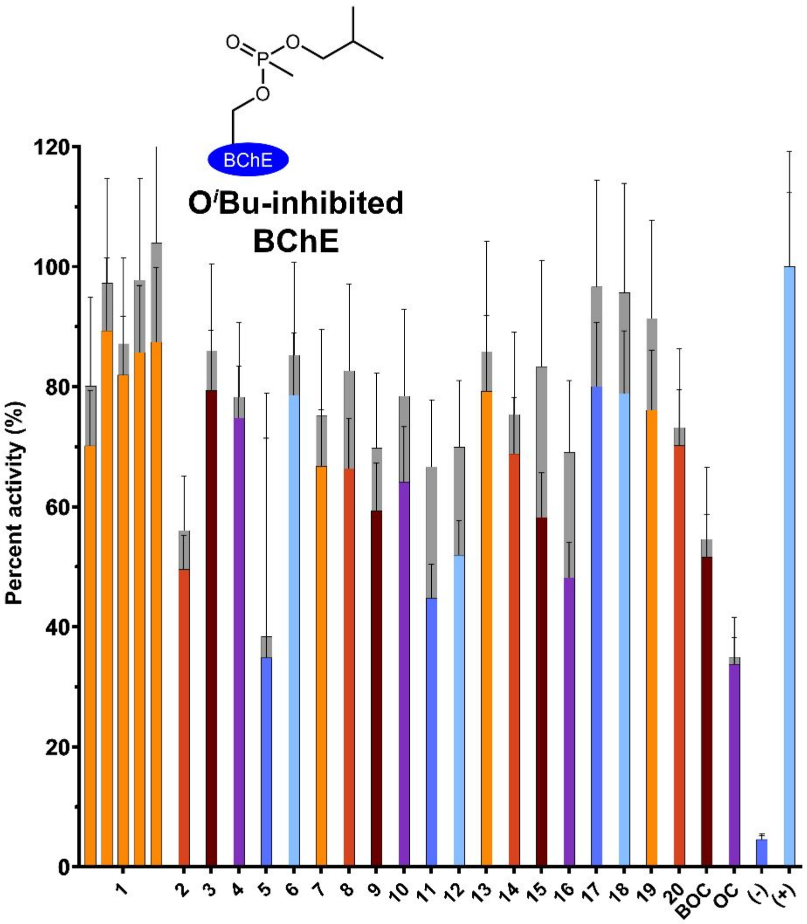

Figure S13. Biochemical *in vitro* recovery (pH 7.5, 37 °C) by *N*-heterocycle-linked QMP compounds of O^{i}Bu-inhibited BChE. Numbered bars represent frameworks listed in **Figure 3**, with left to right bars in the same chain representing different amine leaving groups, along with oxime controls (OC and BOC). The graph shows the results for a concentration of 250 µM and after 15 minutes of incubation, followed by Ellman's assay (with 100x dilution) to evaluate the activity of the reactivated, native BChE. See **Figure S1** for more information.

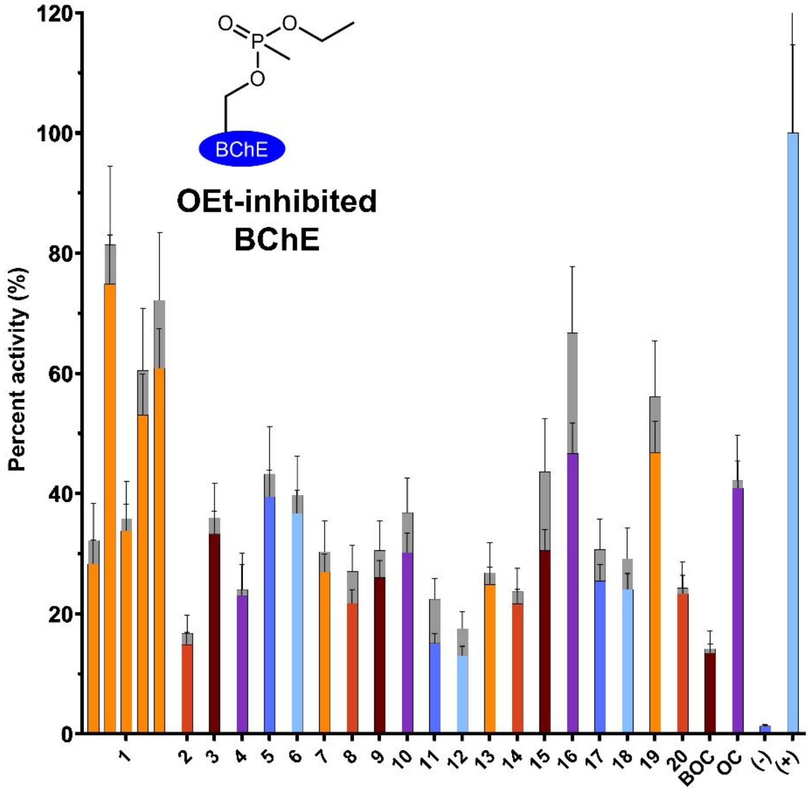

Figure S14. Biochemical *in vitro* recovery (pH 7.5, 37 °C) by *N*-heterocycle-linked QMP compounds of OEt-inhibited BChE. Numbered bars represent frameworks listed in **Figure 3**, with left to right bars in the same chain representing different amine leaving groups, along with oxime controls (OC and BOC). The graph shows the results for a concentration of 250 µM and after 1 hour of incubation, followed by Ellman's assay (with 100x dilution) to evaluate the activity of the reactivated, native BChE. See **Figure S1** for more information.

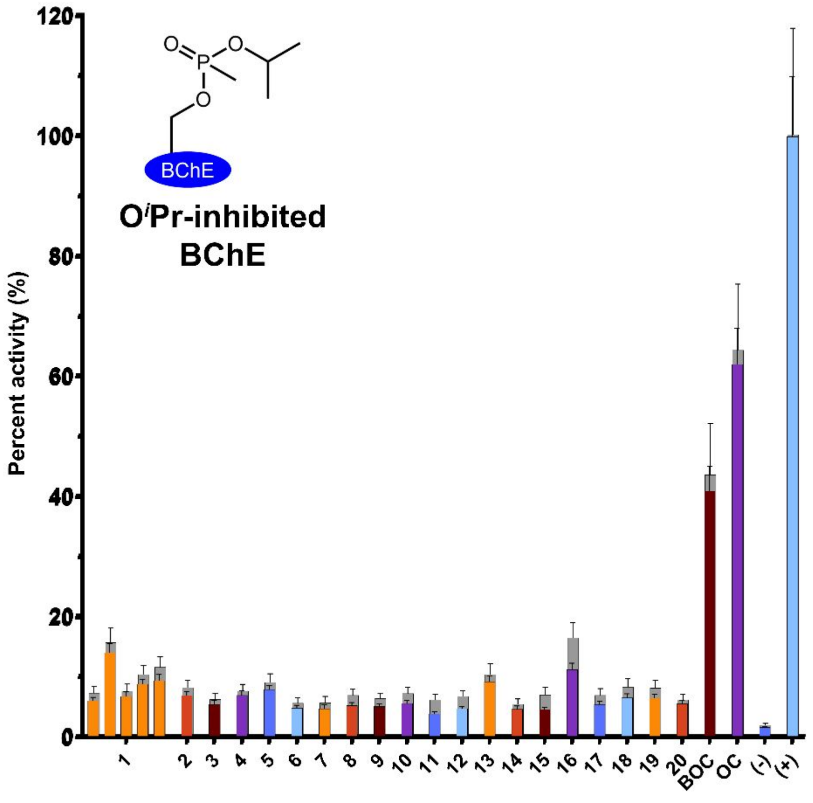

Figure S15. Biochemical *in vitro* recovery (pH 7.5, 37 °C) by *N*-heterocycle-linked QMP compounds of O^{i}Pr-inhibited BChE. Numbered bars represent frameworks listed in **Figure 3**, with left to right bars in the same chain representing different amine leaving groups, along with oxime controls (OC and BOC). The graph shows the results for a concentration of 250 μM and after 1 hour of incubation, followed by Ellman's assay (with 100x dilution) to evaluate the activity of the reactivated, native BChE. See **Figure S1** for more information.

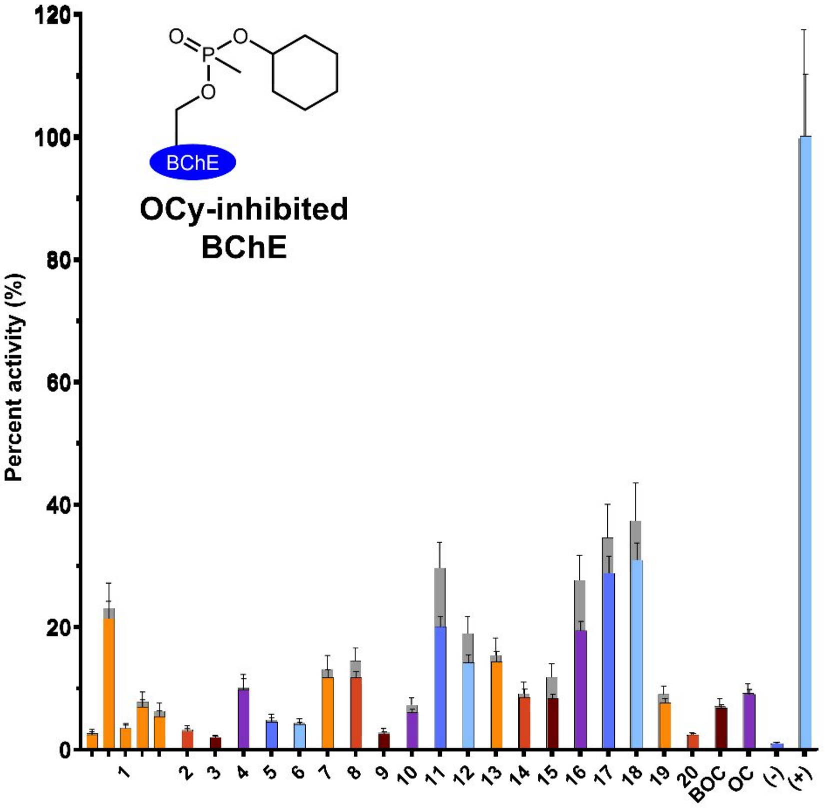

Figure S16. Biochemical *in vitro* recovery (pH 7.5, 37 °C) by *N*-heterocycle-linked QMP compounds of OCy-inhibited BChE. Numbered bars represent frameworks listed in **Figure 3**, with left to right bars in the same chain representing different amine leaving groups, along with oxime controls (OC and BOC). The graph shows the results for a concentration of 250 µM and after 1 hour of incubation, followed by Ellman's assay (with 100x dilution) to evaluate the activity of the reactivated, native BChE. See **Figure S1** for more information.

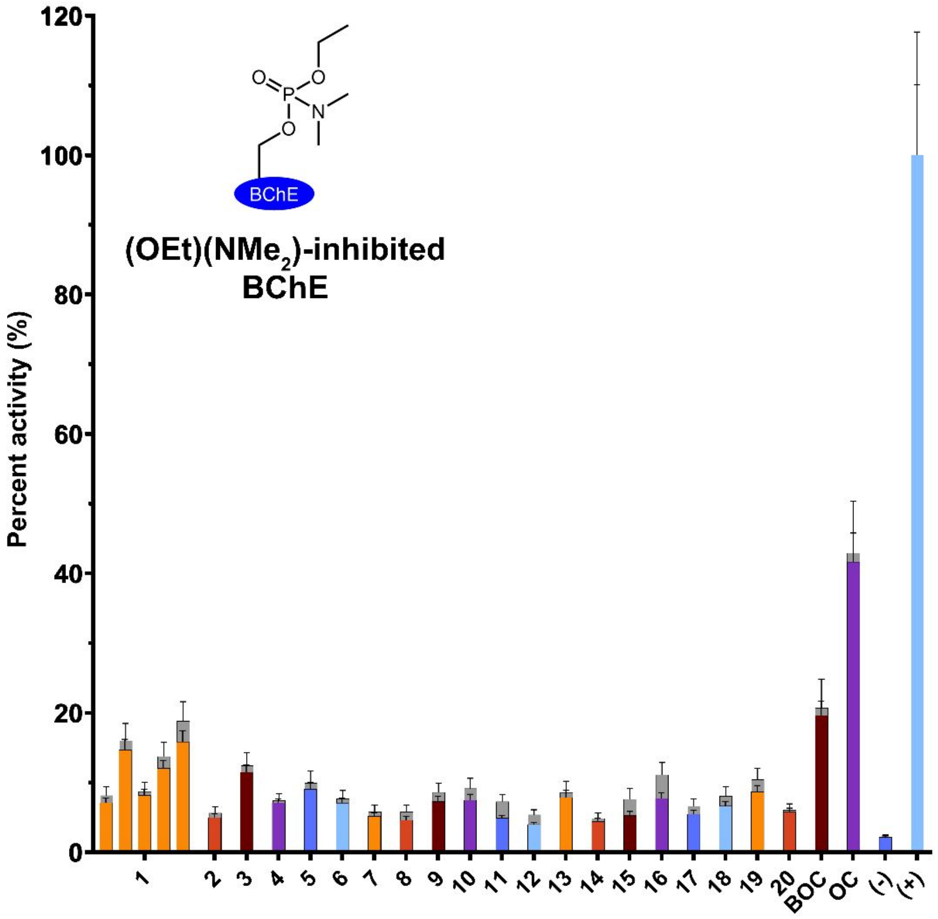

Figure S17. Biochemical *in vitro* recovery (pH 7.5, 37 °C) by *N*-heterocycle-linked QMP compounds of (OEt)(NMe)$_2$-inhibited BChE. Numbered bars represent frameworks listed in **Figure 3**, with left to right bars in the same chain representing different amine leaving groups, along with oxime controls (OC and BOC). The graph shows the results for a concentration of 250 µM and after 1 hour of incubation, followed by Ellman's assay (with 100x dilution) to evaluate the activity of the reactivated, native BChE. See **Figure S1** for more information.

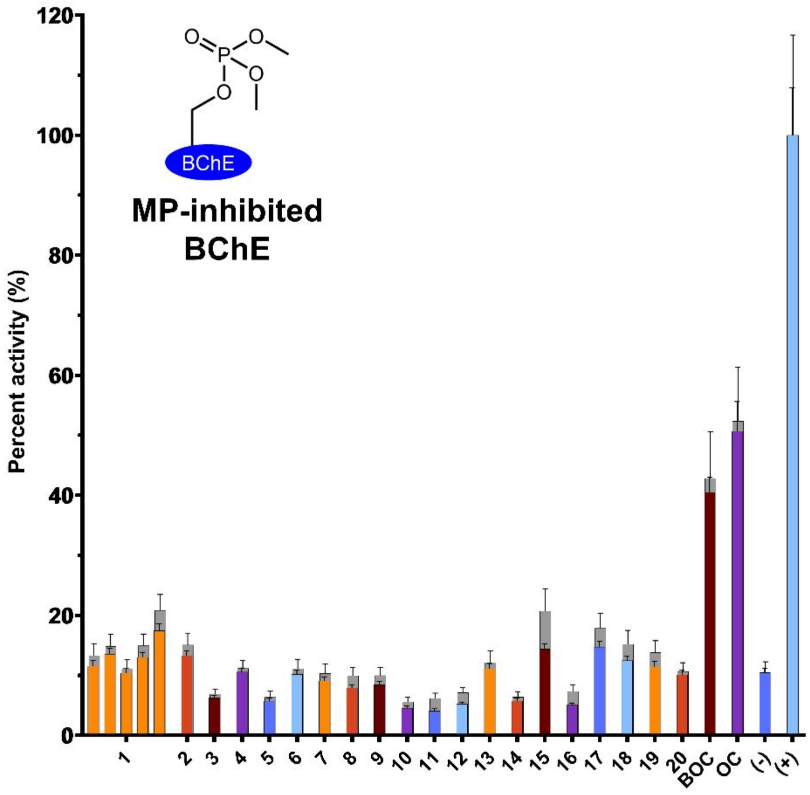

Figure S18. Biochemical *in vitro* recovery (pH 7.5, 37 °C) by *N*-heterocycle-linked QMP compounds of MP-inhibited BChE. Numbered bars represent frameworks listed in **Figure 3**, with left to right bars in the same chain representing different amine leaving groups, along with oxime controls (OC and BOC). The graph shows the results for a concentration of 250 μM and after 1 hour of incubation, followed by Ellman's assay (with 100x dilution) to evaluate the activity of the reactivated, native BChE. See **Figure S1** for more information.

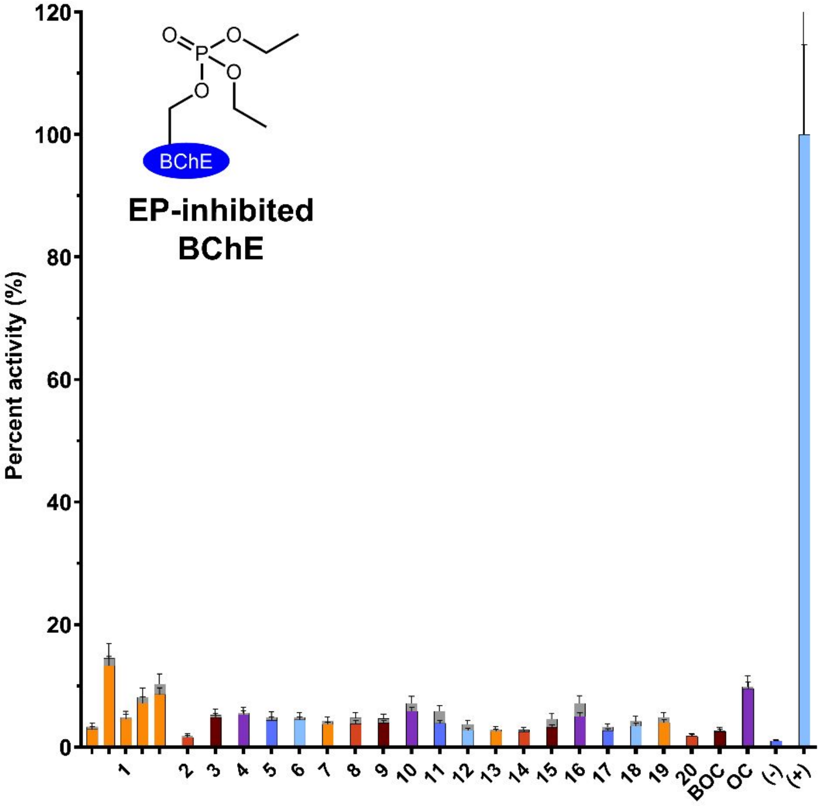

Figure S19. Biochemical *in vitro* recovery (pH 7.5, 37 °C) by *N*-heterocycle-linked QMP compounds of EP-inhibited BChE. Numbered bars represent frameworks listed in **Figure 3**, with left to right bars in the same chain representing different amine leaving groups, along with oxime controls (OC and BOC). The graph shows the results for a concentration of 250 μM and after 1 hour of incubation, followed by Ellman's assay (with 100x dilution) to evaluate the activity of the reactivated, native BChE. See **Figure S1** for more information.

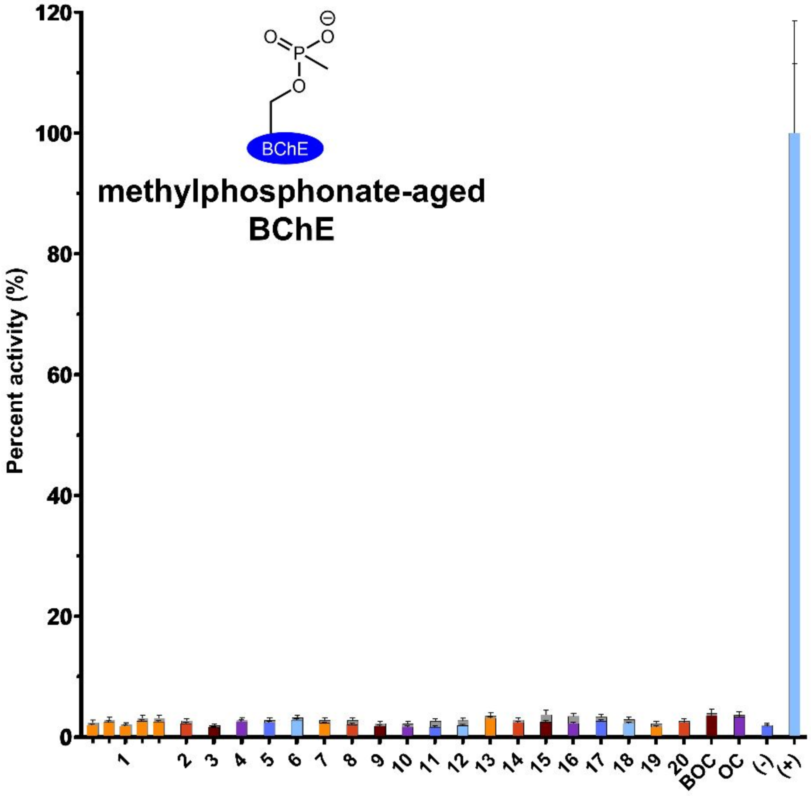

Figure S20. Biochemical *in vitro* recovery (pH 7.5, 37 °C) by *N*-heterocycle-linked QMP compounds of methylphosphonate-aged BChE. Numbered bars represent frameworks listed in **Figure 3**, with left to right bars in the same chain representing different amine leaving groups, along with oxime controls (OC and BOC). The graph shows the results for a concentration of 250 µM and after 24 hours of incubation, followed by Ellman's assay (with 100x dilution) to evaluate the activity of the resurrected, native BChE. See **Figure S1** for more information.

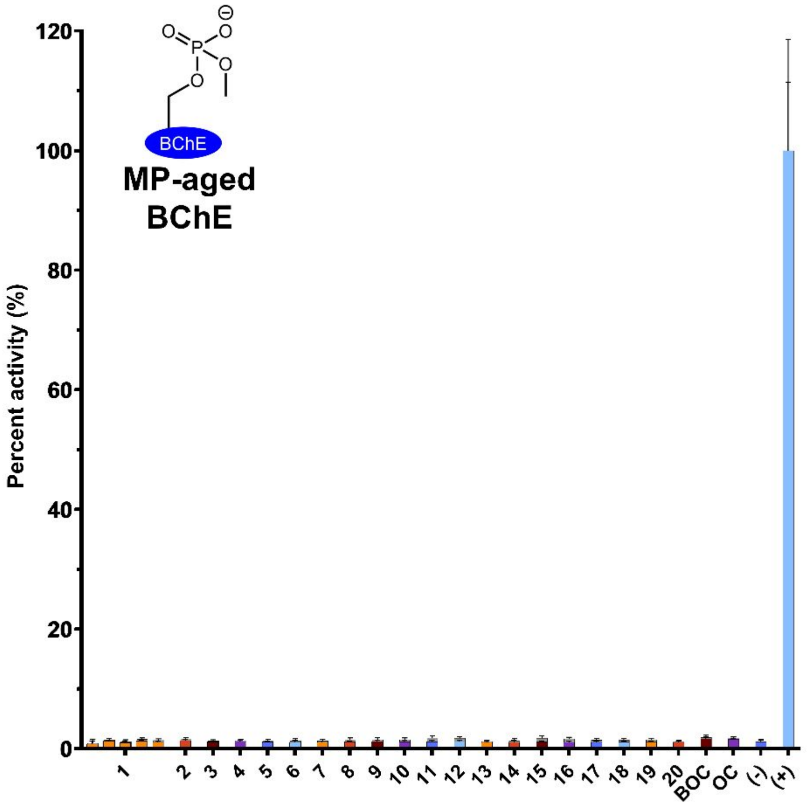

Figure S21. Biochemical *in vitro* recovery (pH 7.5, 37 °C) by *N*-heterocycle-linked QMP compounds of MP-aged BChE. Numbered bars represent frameworks listed in **Figure 3**, with left to right bars in the same chain representing different amine leaving groups, along with oxime controls (OC and BOC). The graph shows the results for a concentration of 250 μM and after 24 hours of incubation, followed by Ellman's assay (with 100x dilution) to evaluate the activity of the resurrected, native BChE. See **Figure S1** for more information.

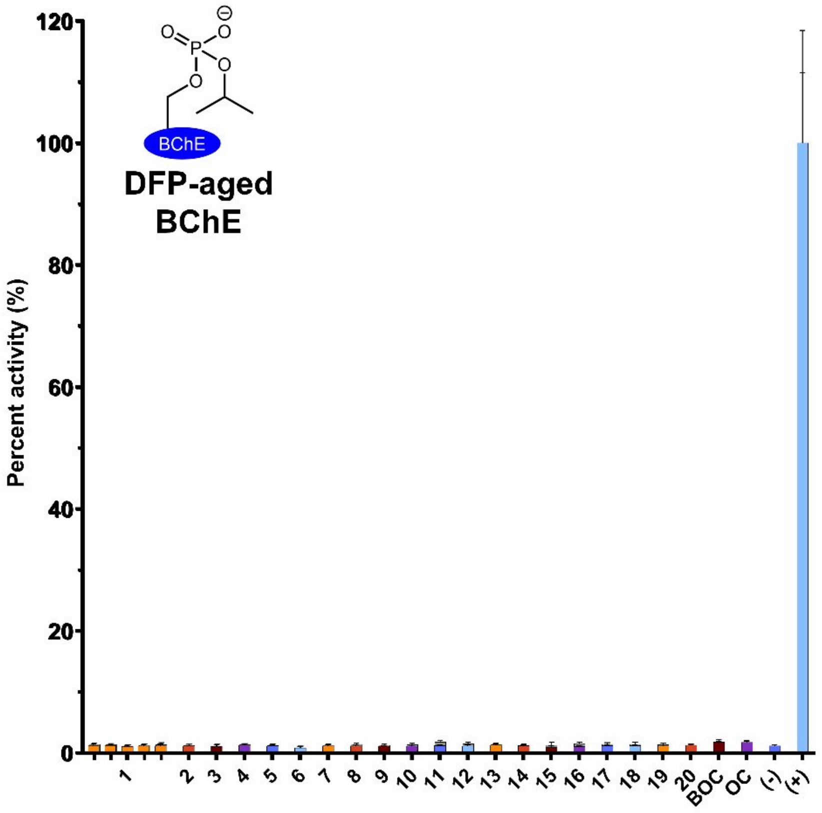

Figure S22. Biochemical *in vitro* recovery (pH 7.5, 37 °C) by *N*-heterocycle-linked QMP compounds of DFP-aged BChE. Numbered bars represent frameworks listed in **Figure 3**, with left to right bars in the same chain representing different amine leaving groups, along with oxime controls (OC and BOC). The graph shows the results for a concentration of 250 µM and after 24 hours of incubation, followed by Ellman's assay (with 100x dilution) to evaluate the activity of the resurrected, native BChE. See **Figure S1** for more information.

Synthesis and Characterization of Final Therapeutics

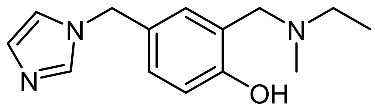

1a

4-((1*H*-imidazol-1-yl)methyl)-2-((ethyl(methyl)amino)methyl)phenol (1a): To a pressure flask equipped with a magnetic stir bar was added paraformaldehyde (51.7 mg, 1.72 mmol), *N*-ethyl-*N*-methylamine (0.15 mL, 1.72 mmol), toluene (5 mL) and ethanol (1 mL). The mixture was then stirred at 60 ℃ for 1 hour, upon which the solution was cooled to room temperature and 4-((1*H*-imidazol-1-yl)methyl)phenol (100 mg, 0.57 mmol) was added. The reaction mixture was then stirred at 120 ℃ for 16 hours. Upon completion, the solution was then concentrated *in vacuo* and the residue was then purified via silica gel chromatography (24 g ReadySep Column, 100% dichloromethane ramp to 9:1 DCM:MeOH) across 10 minutes. The product was eluted in 9:1 DCM:MeOH, resulting in the title compound as a tan oil (75.0 mg, 0.30 mmol, 53%).
^{1}H NMR ($CDCl_3$, 400 MHz) δ_H: 7.49 (s, 1H), 7.05 (s, 1H), 6.99 (dd, *J* = 8.2, 2.2 Hz, 1H), 6.87 (s, 1H), 6.77 (d, *J* = 8.2 Hz, 1H), 6.74 (d, *J* = 2.1 Hz, 1H), 4.97 (s, 2H), 3.64 (s, 2H), 2.52 (q, *J* = 7.2 Hz, 2H), 2.26 (s, 3H), 1.12 (t, *J* = 7.2 Hz, 3H); ^{13}C NMR ($CDCl_3$, 100 MHz) δ_C: 158.4, 137.3, 129.7, 128.1, 127.6, 126.3, 122.5, 119.2, 116.5, 60.7, 50.8, 50.5, 40.8, 12.1; MS-ESI Calculated: 246.1606, Experimental: 246.1603 $(M+H)^+$, Difference: 0.0003 (0.9 ppm); MS-ESI Calculated (para-QM formation): 178.1232, Experimental: 178.1224 $(C_{11}H_{16}NO)^+$, Difference: 0.0008 (2.4 ppm); Purity (HPLC): 100%.

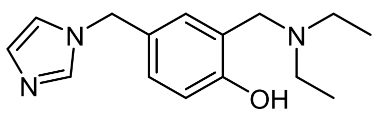

1b

4-((1*H*-imidazol-1-yl)methyl)-2-((diethylamino)methyl)phenol (1b): To a round-bottom flask equipped with a magnetic stir bar was added paraformaldehyde (25 mg, 0.86 mmol), diethylamine (0.09 mL, 0.86 mmol), toluene (5 mL), and ethanol (1 mL). A septum was placed on the flask and the mixture was stirred at 60 ℃ for 2 hours. The reaction mixture was allowed to cool to room temperature and 4-((1*H*-imidazol-1-yl)methyl)phenol (100 mg, 0.57 mmol) was added. A septum was placed on the flask and the reaction mixture was stirred at 80 ℃ overnight. The reaction mixture was then concentrated *in vacuo* and the residue was purified via silica gel chromatography (24 g ReadySep Column, 100% dichloromethane ramp to 9:1 DCM:MeOH) across 10 minutes. The product was eluted in 9:1 DCM:MeOH, resulting in the title compound as a clear oil (23 mg, 0.09 mmol, 15%). ^{1}H NMR ($CDCl_3$, 400 MHz) δ_H: 7.50 (s, 1H), 7.04 (s, 1H), 6.98 (dd, *J* = 8.2, 2.2 Hz, 1H), 6.87 (s, 1H), 6.78 - 6.72 (m, 2H), 4.96 (s, 2H), 3.71 (s, 2H), 2.59 (q, *J* = 7.2 Hz, 4H), 1.08 (t, *J* = 7.2 Hz, 6H); ^{13}C NMR ($CDCl_3$, 100 MHz) δ_C: 158.5, 137.3, 129.5, 128.0, 127.7, 126.2, 122.5, 119.2, 116.5, 56.7, 50.6, 46.4, 11.2; MS-ESI Calculated: 260.1763, Experimental: 260.1759 $(M+H)^+$, Difference: 0.0006 (1.8 ppm); MS-ESI Calculated (para-QM formation): 192.1388, Experimental: 192.1385 $(C_{12}H_{18}NO)^+$, Difference: 0.0003 (0.9 ppm); Purity (HPLC): 100%.

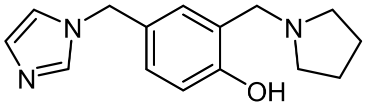

1c

4-((1*H*-imidazol-1-yl)methyl)-2-(pyrrolidin-1-ylmethyl)phenol (1c): To a pressure flask equipped with a magnetic stir bar was added paraformaldehyde (25.8 mg, 0.86 mmol), pyrrolidine (0.07 mL, 0.86 mmol), toluene (5 mL) and ethanol (1 mL). The mixture was then stirred at 60 ℃ for 1 hour, upon which the solution was cooled to room temperature and 4-((1*H*-imidazol-1-yl)methyl)phenol (100 mg, 0.57 mmol) was added. The reaction mixture was then stirred at 120 ℃ for 16 hours. Upon completion, the solution was then concentrated *in vacuo* and the residue was then purified via silica gel chromatography (24 g ReadySep Column, 100% dichloromethane ramp to 9:1 DCM:MeOH) across 6 minutes. The product was eluted in 9:1 DCM:MeOH, resulting in the title compound as a tan oil (96.0 mg, 0.37 mmol, 65%). ^{1}H NMR ($CDCl_3$, 400 MHz) δ_H: 7.50 (s, 1H), 7.05 (t, *J* = 1.1 Hz, 1H), 6.99 (dd, *J* = 8.2, 2.3 Hz, 1H), 6.87 (t, *J* = 1.2 Hz, 1H), 6.77 (d, *J* = 8.2 Hz, 1H), 6.76 (s, 1H), 4.97 (s, 2H), 3.77 (s, 2H), 2.67 - 2.56 (m, 4H), 1.89 - 1.79 (m, 4H); ^{13}C NMR ($CDCl_3$, 100 MHz) δ_C: 158.2, 137.1, 129.4, 127.9, 127.2, 126.1, 122.9, 119.1, 116.2, 58.5, 53.4, 50.4, 23.6; MS-ESI Calculated: 258.1606, Experimental: 258.1601 $(M+H)^+$, Difference: 0.0005 (1.5 ppm); MS-ESI Calculated (para-QM formation): 190.1232, Experimental: 190.1228 $(C_{12}H_{16}NO)^+$, Difference: 0.0004 (1.2 ppm); Purity (HPLC): 100%.

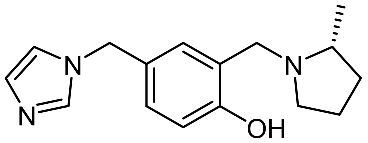

1d

(*R*)-4-((1*H*-imidazol-1-yl)methyl)-2-((2-methylpyrrolidin-1-yl)methyl)phenol (1d): To a pressure flask equipped with a magnetic stir bar was added paraformaldehyde (25.8 mg, 0.86 mmol), (*R*)-2-methylpyrrolidine (0.09 mL, 0.86 mmol), toluene (5 mL) and ethanol (1 mL). The mixture was then stirred at 60 °C for 1 hour, upon which the solution was cooled to room temperature and 4-((1*H*-imidazol-1-yl)methyl)phenol (100 mg, 0.57 mmol) was added. The reaction mixture was then stirred at 120 °C for 16 hours. Upon completion, the solution was then concentrated *in vacuo* and the residue was then purified via silica gel chromatography (40 g ReadySep Column, 100% dichloromethane ramp to 9:1 DCM:MeOH) across 12 minutes. The product was eluted in 92:8 DCM:MeOH, resulting in the title compound as a tan oil (68.0 mg, 0.25 mmol, 44%). ^{1}H NMR ($CDCl_3$, 400 MHz) δ_H: 7.50 (s, 1H), 7.05 (s, 1H), 7.01 - 6.96 (m, 1H), 6.87 (s, 1H), 6.78 - 6.73 (m, 2H), 4.97 (s, 2H), 4.19 (d, *J* = 14.0 Hz, 1H), 3.29 (d, *J* = 14.0 Hz, 1H), 3.05 - 2.96 (m, 1H), 2.60 - 2.50 (m, 1H), 2.22 (q, *J* = 9.7 Hz, 1H), 2.09 - 1.97 (m, 1H), 1.86 - 1.67 (m, 2H), 1.56 - 1.44 (m, 1H), 1.19 (d, *J* = 6.1 Hz, 3H); ^{13}C NMR ($CDCl_3$, 100 MHz) δ_C: 158.2, 137.2, 129.6, 128.0, 127.2, 126.2, 123.3, 119.2, 116.3, 60.1, 56.8, 54.0, 50.6, 32.8, 21.8, 18.9; MS-ESI Calculated: 272.1763, Experimental: 272.1759 $(M+H)^+$, Difference: 0.0004 (1.2 ppm), MS-ESI Calculated (para-QM formation): 204.1388, Experimental: 204.1383 $(C_{13}H_{18}NO)^+$, Difference: 0.0005 (1.5 ppm); Purity (HPLC): 97%.

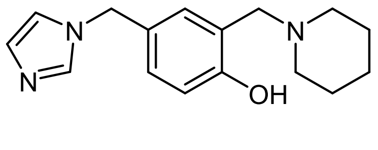

1e

4-((1*H*-imidazol-1-yl)methyl)-2-(piperidin-1-ylmethyl)phenol (1e): To a pressure flask equipped with a magnetic stir bar was added paraformaldehyde (51.7 mg, 1.72 mmol), piperidine (0.15 mL, 1.72 mmol), toluene (5 mL) and ethanol (1 mL). The mixture was then stirred at 60 ℃ for 1 hour, upon which the solution was cooled to room temperature and 4-((1*H*-imidazol-1-yl)methyl)phenol (100 mg, 0.57 mmol) was added. The reaction mixture was then stirred at 120 ℃ for 16 hours. Upon completion, the solution was then concentrated *in vacuo* and the residue was then purified via silica gel chromatography (24 g ReadySep Column, 100% dichloromethane ramp to 9:1 DCM:MeOH) across 8 minutes. The product was eluted in 92:8 DCM:MeOH, resulting in the title compound as a tan oil (50.1 mg, 0.18 mmol, 32%). ^{1}H NMR ($CDCl_3$, 400 MHz) δ_H: 7.45 (s, 1H), 7.00 (s, 1H), 6.94 (dd, J = 8.2, 2.3 Hz, 1H), 6.83 (s, 1H), 6.75 - 6.69 (m, 2H), 4.92 (s, 2H), 3.57 (s, 2H), 2.73 - 2.08 (br m, 4H), 1.64 - 1.33 (m, 6H); ^{13}C NMR ($CDCl_3$, 100 MHz) δ_C: 158.2, 137.1, 129.5, 127.9, 127.7, 126.2, 122.0, 119.0, 116.3, 61.8, 53.8, 50.4, 25.7, 23.8; MS-ESI Calculated: 272.1763, Experimental: 272.1760 $(M+H)^+$, Difference: 0.0003 (0.9 ppm); MS-ESI Calculated (para-QM formation): 204.1388, Experimental: 204.1384 $(C_{13}H_{18}NO)^+$, Difference: 0.0004 (1.2 ppm); Purity (HPLC): 100%.

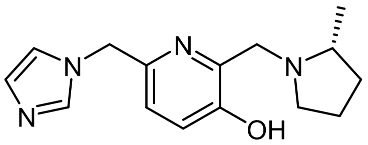

2

(*R*)-6-((1*H*-imidazol-1-yl)methyl)-2-((2-methylpyrrolidin-1-yl)methyl)pyridin-3-ol (2): To a pressure flask equipped with a magnetic stir bar was added paraformaldehyde (43.7 mg, 1.46 mmol), (*R*)-2-methylpyrrolidine (0.15 mL, 1.46 mmol), toluene (5 mL) and ethanol (1 mL). The

mixture was then stirred at 60 °C for 1 hour, upon which the solution was cooled to room temperature and 6-((1*H*-imidazol-1-yl)methyl)pyridin-3-ol (150 mg, 0.73 mmol) was added. The reaction mixture was then stirred at 120 °C for 16 hours. Upon completion, the solution was then concentrated *in vacuo* and the residue was then purified via silica gel chromatography (24 g ReadySep Column, 100% dichloromethane ramp to 8:2 DCM:MeOH) across 8 minutes. The product was eluted in 85:15 DCM:MeOH, resulting in the title compound as a tan oil (152 mg, 0.56 mmol, 77%). ^{1}H NMR ($CDCl_3$, 400 MHz) δ_H: 7.56 (s, 1H), 7.05 (s, 1H), 6.99 (d, J = 8.3 Hz, 1H), 6.94 (t, J = 1.2 Hz, 1H), 6.79 (d, J = 8.3 Hz, 1H), 5.08 (s, 2H), 4.28 (d, J = 15.0 Hz, 1H), 3.66 (d, J = 15.0 Hz, 1H), 3.10 - 3.02 (m, 1H), 2.70 - 2.60 (m, 1H), 2.37 (q, J = 9.8 Hz, 1H), 2.11 - 2.00 (m, 1H), 1.89 - 1.72 (m, 2H), 1.57 - 1.46 (m, 1H), 1.20 (d, J = 6.2 Hz, 3H); ^{13}C NMR ($CDCl_3$, 100 MHz) δ_C: 154.2, 145.2, 143.5, 137.5, 129.7, 123.6, 121.5, 119.4, 60.4, 59.2, 54.4, 52.2, 32.7, 22.0, 18.8; MS-ESI Calculated: 273.1715, Experimental: 273.1713 $(M+H)^+$, Difference: 0.0002 (0.6 ppm); MS-ESI Calculated: 205.1341, Experimental: 205.1337 $(C_{12}H_{17}N_2O)^+$, Difference: 0.0004 (1.2 ppm); Purity (HPLC): 95%.

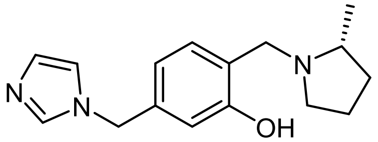

3

(*R*)-5-((1*H*-imidazol-1-yl)methyl)-2-((2-methylpyrrolidin-1-yl)methyl)phenol (3): To a pressure flask equipped with a magnetic stir bar was added paraformaldehyde (51.7 mg, 1.72 mmol), *(R)*-2-methylpyrrolidine (0.17 mL, 1.72 mmol), toluene (5 mL) and ethanol (1 mL). The mixture was then stirred at 60 °C for 1 hour, upon which the solution was cooled to room

temperature and 3-((1*H*-imidazol-1-yl)methyl)phenol (100 mg, 0.57 mmol) was added. The reaction mixture was then stirred at 120 °C for 16 hours. Upon completion, the solution was then concentrated *in vacuo* and the residue was then purified via silica gel chromatography (12 g ReadySep Column, 100% dichloromethane ramp to 95:5 DCM:MeOH) across 7 minutes. The product was eluted in 95:5 DCM:MeOH, resulting in the title compound as a tan oil (76.0 mg, 0.28 mmol, 49%). ^{1}H NMR ($CDCl_3$, 400 MHz) δ_H: 7.48 (s, 1H), 7.02 (s, 1H), 6.90 (d, J = 7.6 Hz, 1H), 6.86 (s, 1H), 6.57 (d, J = 1.6 Hz, 1H), 6.49 (dd, J = 7.6, 1.8 Hz, 1H), 4.97 (s, 2H), 4.17 (d, J = 14.0 Hz, 1H), 3.31 (d, J = 14.1 Hz, 1H), 3.02 - 2.94 (m, 1H), 2.58 - 2.48 (m, 1H), 2.20 (q, J = 9.8 Hz, 1H), 2.05 - 1.95 (m, 1H), 1.82 - 1.64 (m, 2H), 1.52 - 1.40 (m, 1H), 1.17 (d, J = 6.1 Hz, 3H); ^{13}C NMR ($CDCl_3$, 100 MHz) δ_C: 158.4, 137.4, 136.6, 129.5, 128.3, 122.7, 119.3, 117.6, 114.9, 60.0, 56.4, 53.9, 50.6, 32.7, 21.7, 18.8; MS-ESI Calculated: 272.1763, Experimental: 272.1759 $(M+H)^+$, Difference: 0.0004 (1.2 ppm); Purity (HPLC): 100%.

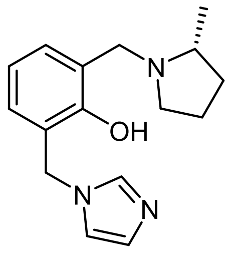

4

(*R*)-2-((1*H*-imidazol-1-yl)methyl)-6-((2-methylpyrrolidin-1-yl)methyl)phenol (4): To a pressure tube equipped with a magnetic stir bar was added paraformaldehyde (52 mg, 1.72 mmol), (*R*)-2-methylpyrrolidine (0.17 mL, 1.72 mmol), toluene (5 mL), and ethanol (1 mL). The tube was then sealed and the reaction was stirred at 60 °C for 2 hours. The reaction mixture was then cooled to room temperature and 2-((1*H*-imidazol-1-yl)methyl)phenol (100 mg, 0.57 mmol)

was added. The tube was then sealed and stirred at 110 °C for 18 hours. The reaction mixture was then concentrated *in vacuo* and purified by silica gel chromatography (12 g ReadySep Column, 99% dichloromethane, 1% triethylamine ramp to 9:1 DCM:MeOH with 1% triethylamine). The product was eluted in 93:7 DCM:MeOH with 1% triethylamine, resulting in the title compound as a clear oil (44 mg, 0.16 mmol, 28%). ^{1}H NMR ($CDCl_3$, 400 MHz) δ_H: 7.56 (s, 1H), 7.01 (s, 1H), 6.96 (s, 1H), 6.94 - 6.89 (m, 1H), 6.88 - 6.83 (m, 1H), 6.68 (t, *J* = 7.5 Hz, 2H), 5.07 (s, 2H), 4.19 (d, *J* = 14.0 Hz, 1H), 3.36 (d, *J* = 14.0 Hz, 1H), 3.03 - 2.93 (m, 1H), 2.62 - 2.50 (m, 1H), 2.24 (q, *J* = 9.8 Hz, 1H), 2.09 - 1.97 (m, 1H), 1.85 - 1.66 (m, 2H), 1.56 - 1.44 (m, 1H), 1.19 (d, *J* = 6.1 Hz, 3H); ^{13}C NMR ($CDCl_3$, 100 MHz) δ_C: 155.9, 137.6, 129.1, 127.99, 127.90, 123.0, 122.8, 119.4, 118.7, 60.0, 56.8, 53.9, 45.6, 32.8, 21.8, 18.8; MS-ESI Calculated: 272.1763, Experimental: 272.1763 $(M+H)^+$, Difference: 0.0000 (0.0 ppm); Purity (HPLC): 100%.

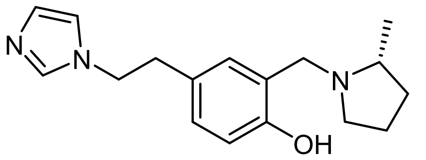

5

(*R*)-4-(2-(1*H*-imidazol-1-yl)ethyl)-2-((2-methylpyrrolidin-1-yl)methyl)phenol (5): To a pressure flask equipped with a magnetic stir bar was added paraformaldehyde (47.8 mg, 1.59 mmol), *(R)*-2-methylpyrrolidine (0.16 mL, 1.59 mmol), toluene (5 mL) and ethanol (1 mL). The mixture was then stirred at 60 °C for 1 hour, upon which the solution was cooled to room temperature and 4-(2-(1*H*-imidazol-1-yl)ethyl)phenol (100 mg, 0.53 mmol) was added. The reaction mixture was then stirred at 120 °C for 16 hours. Upon completion, the solution was then concentrated *in vacuo* and the residue was then purified via silica gel chromatography (12 g

ReadySep Column, 100% dichloromethane ramp to 9:1 DCM:MeOH) across 15 minutes, the product was eluted in 93:7 DCM:MeOH, resulting in the title compound as a tan oil (46.0 mg, 0.16 mmol, 30%). ^{1}H NMR ($CDCl_3$, 400 MHz) δ_H: 7.26 (s, 1H), 6.97 (s, 1H), 6.83 (dd, *J* = 8.2, 2.1 Hz, 1H), 6.76 (s, 1H), 6.69 (d, *J* = 8.2 Hz, 1H), 6.54 (d, *J* = 1.9 Hz, 1H), 4.14 (d, *J* = 14.0 Hz, 1H), 4.10 - 4.03 (m, 2H), 3.23 (d, *J* = 14.0 Hz, 1H), 3.02 - 2.94 (m, 1H), 2.88 (t, *J* = 7.0 Hz, 2H), 2.58 - 2.47 (m, 1H), 2.20 (q, *J* = 9.8 Hz, 1H), 2.06 - 1.95 (m, 1H), 1.82 - 1.64 (m, 2H), 1.53 - 1.42 (m, 1H), 1.17 (d, *J* = 6.1 Hz, 3H); ^{13}C NMR ($CDCl_3$, 100 MHz) δ_C: 156.8, 137.1, 129.2, 128.5, 128.3, 127.6, 122.8, 118.9, 116.1, 60.1, 56.6, 53.9, 48.9, 37.1, 32.8, 21.8, 18.8; MS-ESI Calculated: 286.1919, Experimental: 286.1919 $(M+H)^+$, Difference: 0.0000 (0.0 ppm); Purity (HPLC): 100%.

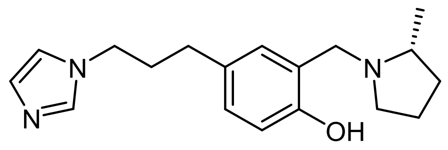

6

(*R*)-4-(3-(1*H*-imidazol-1-yl)propyl)-2-((2-methylpyrrolidin-1-yl)methyl)phenol (6): To a pressure tube equipped with a magnetic stir bar was added paraformaldehyde (44 mg, 1.48 mmol), (*R*)-2-methylpyrrolidine (0.15 mL, 1.48 mmol), toluene (5 mL), and ethanol (1 mL). The tube was then sealed and the reaction was stirred at 60 ℃ for 2 hours. The reaction mixture was then cooled to room temperature and 4-(3-(1*H*-imidazol-1-yl)propyl)phenol (100 mg, 0.49 mmol) was added. The tube was then sealed and stirred at 110 ℃ for 18 hours. The reaction mixture was then concentrated *in vacuo* and purified by silica gel chromatography (24 g ReadySep Column, 99% dichloromethane with 1% triethylamine). The product was eluted in 99% dichloromethane with 1% triethylamine, resulting in the title compound as a clear oil (45

mg, 0.15 mmol, 30%). ^{1}H NMR ($CDCl_3$, 400 MHz) δ_H: 7.41 (s, 1H), 7.03 (s, 1H), 6.92 - 6.86 (m, 2H), 6.73 - 6.68 (m, 2H), 4.19 (d, *J* = 13.9 Hz, 1H), 3.88 (t, *J* = 7.0 Hz, 2H), 3.27 (d, *J* = 13.9 Hz, 1H), 3.05 - 2.97 (m, 1H), 2.58 - 2.48 (m, 1H), 2.47 (t, *J* = 7.5 Hz, 2H), 2.21 (q, *J* = 9.7 Hz, 1H), 2.09 - 1.95 (m, 3H), 1.83 - 1.65 (m, 2H), 1.53 - 1.42 (m, 1H), 1.18 (d, *J* = 6.1 Hz, 3H); ^{13}C NMR ($CDCl_3$, 100 MHz) δ_C: 156.3, 137.1, 130.3, 129.4, 128.1, 127.7, 122.7, 118.7, 115.9, 60.0, 56.8, 53.9, 46.1, 32.8, 32.5, 31.6, 21.8, 18.8; MS-ESI Calculated: 300.2076, Experimental: 300.2076 $(M+H)^+$, Difference: 0.0000 (0.0 ppm); Purity (HPLC): 100%.

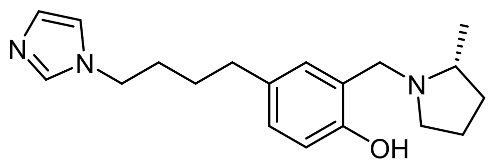

7

(*R*)-4-(4-(1*H*-imidazol-1-yl)butyl)-2-((2-methylpyrrolidin-1-yl)methyl)phenol (7): To a round-bottom flask equipped with a magnetic stir bar was added paraformaldehyde (10 mg, 0.35 mmol), (*R*)-2-methylpyrrolidine (0.03 mL, 0.35 mmol), toluene (5 mL), and ethanol (1 mL). The resulting solution was stirred at 60 °C for 1 hour. Then, 4-(4-(1*H*-imidazol-1-yl)butyl)phenol (50 mg, 0.23 mmol) was added in one portion and then stirred at 120 °C for 16 hours. Upon completion, the reaction mixture was concentrated *in vacuo* and the residue was purified via silica gel chromatography (9:1 DCM:MeOH; 1% NEt_3), resulting in the title compound as a yellow oil (17.0 mg, 0.05 mmol, 23%). ^{1}H NMR ($CDCl_3$, 400MHz) δ_H: 7.43 (s, 1H), 7.03 (s, 1H), 6.90 (dd, *J* = 8.2, 1.9 Hz, 1H), 6.86 (s, 1H), 6.73 - 6.68 (m, 2H), 4.20 (d, *J* = 13.9 Hz, 1H), 3.90 (t, *J* = 7.0 Hz, 2H), 3.27 (d, *J* = 13.9 Hz, 1H), 3.06 - 2.99 (m, 1H), 2.59 - 2.45 (m, 3H), 2.26 - 2.17 (m, 1H), 2.09 - 1.97 (m, 1H), 1.83 - 1.66 (m, 4H), 1.60 - 1.43 (m, 3H), 1.19 (d, *J* = 6.1 Hz,

3H); ^{13}C NMR ($CDCl_3$, 100MHz) δ_C: 156.1, 137.1, 131.7, 129.4, 128.2, 127.7, 122.6, 118.8, 115.8, 60.0, 57.0, 54.0, 47.0, 34.5, 32.9, 30.6, 28.6, 21.8, 18.9; MS-ESI Calculated: 314.2232, Experimental: 314.2227 $(M+H)^+$, 157.6149 (2+), Difference: 0.0005 (1.6 ppm).

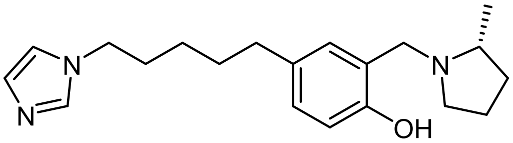

8

(*R*)-4-(5-(1*H*-imidazol-1-yl)pentyl)-2-((2-methylpyrrolidin-1-yl)methyl)phenol (8): To a round-bottom flask equipped with a magnetic stir bar was added paraformaldehyde (10 mg, 0.33 mmol), (*R*)-2-methylpyrrolidine (0.03 mL, 0.33 mmol), toluene (5 mL), and ethanol (1 mL). The resulting solution was stirred at 60 °C for 1 hour. Then, 4-(5-(1*H*-imidazol-1-yl)pentyl)phenol (50 mg, 0.22 mmol) was added in one portion and then stirred at 120 °C for 16 hours. Upon completion, the reaction mixture was concentrated *in vacuo* and the residue was purified via silica gel chromatography (9:1 DCM:MeOH; 1% NEt_3), resulting in the title compound as a yellow oil (16.0 mg, 0.05 mmol, 22%). ^1H NMR ($CDCl_3$, 400MHz) δ_H: 7.43 (s, 1H), 7.03 (s, 1H), 6.91 (dd, J = 8.1, 1.9 Hz, 1H), 6.87 (t, J = 1.2 Hz, 1H), 6.73 (d, J = 2.0 Hz, 1H), 6.70 (d, J = 8.2 Hz, 1H), 4.21 (d, J = 13.9 Hz, 1H), 3.90 (t, J = 7.1 Hz, 2H), 3.28 (d, J = 13.9 Hz, 1H), 3.06 - 2.99 (m, 1H), 2.59 - 2.49 (m, 1H), 2.47 (t, J = 7.7 Hz, 2H), 2.22 (q, J = 9.8 Hz, 1H), 2.08 - 1.97 (m, 1H), 1.83 - 1.66 (m, 4H), 1.62 - 1.45 (m, 3H), 1.36 - 1.23 (m, 3H), 1.20 (d, J = 6.1 Hz, 3H); ^{13}C NMR ($CDCl_3$, 100MHz) δ_C: 155.9, 137.1, 132.3, 129.4, 128.1, 127.7, 122.5, 118.8, 115.7, 60.0, 57.0, 54.0, 47.0, 34.9, 32.9, 31.2, 31.1, 26.2, 21.8, 18.9; MS-ESI Calculated: 328.2389,

Experimental: 328.2381 $(M+H)^+$, 164.6227 (2+), Difference: 0.0008 (2.4 ppm); Purity (HPLC): 100%.

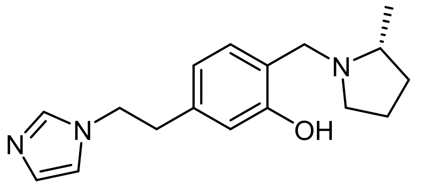

9

(*R*)-5-(2-(1*H*-imidazol-1-yl)ethyl)-2-((2-methylpyrrolidin-1-yl)methyl)phenol (9): To a round-bottom flask was added paraformaldehyde (24 mg, 0.80 mmol), (*R*)-2-methylpyrrolidine (0.08 mL, 0.80 mmol), toluene (5 mL), and ethanol (1 mL). The reaction mixture was then heated to 60 °C and stirred for 1 hour. The reaction mixture was then cooled to room temperature upon which 3-(2-(1*H*-imidazol-1-yl)ethyl)phenol (100 mg, 0.53 mmol) was added in one portion. The reaction was then set to stir at 110 °C for 16 hours. Upon completion, the solution was concentrated *in vacuo,* and the residue was purified via silica gel chromatography (9:1 DCM:MeOH; 1% NEt_3), resulting in the title compound as a yellow oil (77.0 mg, 0.27 mmol, 51%). ^1H NMR ($CDCl_3$, 400MHz) δ_H: 7.27 (s, 1H), 6.97 (s, 1H), 6.84 - 6.79 (m, 2H), 6.55 (d, J = 1.4 Hz, 1H), 6.37 (dd, J = 7.6, 1.5 Hz, 1H), 4.15 (d, J = 13.9 Hz, 1H), 4.09 (t, J = 7.2 Hz, 2H), 3.28 (d, J = 13.9 Hz, 1H), 3.01 - 2.94 (m, 1H), 2.91 (t, J = 7.2 Hz, 2H), 2.56 - 2.46 (m, 1H), 2.19 (q, J = 9.7 Hz, 1H), 2.04 - 1.94 (m, 1H), 1.81 - 1.63 (m, 2H), 1.52 - 1.40 (m, 1H), 1.16 (d, J = 6.1 Hz, 3H); ^{13}C NMR ($CDCl_3$, 100MHz) δ_C: 158.1, 137.8, 137.0, 129.2, 128.0, 121.4, 119.0, 118.7, 115.8, 59.9, 56.5, 53.9, 48.3, 37.5, 32.8, 21.7, 18.8; MS-ESI Calculated: 286.1919,

Experimental: 286.1914 $(M+H)^+$, 143.5993 (2+), Difference: 0.0005 (1.7 ppm); Purity (HPLC): 100%.

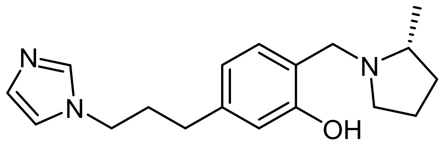

10

(*R*)-5-(3-(1*H*-imidazol-1-yl)propyl)-2-((2-methylpyrrolidin-1-yl)methyl)phenol (10): To a round-bottom flask equipped with a magnetic stir bar was added paraformaldehyde (11 mg, 0.37 mmol), (*R*)-2-methylpyrrolidine (0.04 mL, 0.37 mmol), toluene (5 mL), and ethanol (1 mL). The resulting solution was stirred at 60 °C for 1 hour. Then, 3-(3-(1*H*-imidazol-1-yl)propyl)phenol (50 mg, 0.25 mmol) was added in one portion and stirred at 120 °C for 16 hours. Upon completion, the reaction mixture was concentrated *in vacuo* and the residue was purified via silica gel chromatography (9:1 DCM:MeOH; 1% NEt_3), resulting in the title compound as a yellow oil (28.0 mg, 0.09 mmol, 38%). ^1H NMR ($CDCl_3$, 400MHz) δ_H: 7.43 (s, 1H), 7.04 (s, 1H), 6.90 - 6.84 (m, 2H), 6.59 (d, J = 1.6 Hz, 1H), 6.52 (dd, J = 7.6, 1.7 Hz, 1H), 4.19 (d, J = 13.9 Hz, 1H), 3.90 (t, J = 7.1 Hz, 2H), 3.30 (d, J = 13.9 Hz, 1H), 3.06 - 2.98 (m, 1H), 2.57 - 2.47 (m, 3H), 2.22 (q, J = 9.8 Hz, 1H), 2.12 - 1.97 (m, 3H), 1.82 - 1.66 (m, 2H), 1.54 - 1.43 (m, 1H), 1.19 (d, J = 6.1 Hz, 3H); ^{13}C NMR ($CDCl_3$, 100MHz) δ_C: 158.1, 140.8, 137.2, 129.5, 128.0, 120.8, 118.9, 118.8, 115.7, 60.0, 56.6, 54.0, 46.2, 32.8, 32.25, 32.21, 21.8, 18.9; MS-ESI Calculated: 300.2076, Experimental: 300.2070 $(M+H)^+$, 150.6071 (2+), Difference: 0.0006 (2.0 ppm); Purity (HPLC): 95%.

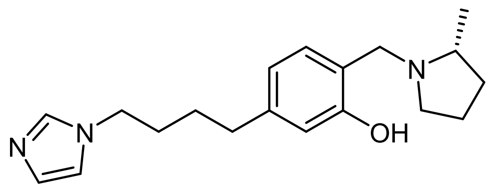

11

(*R*)-5-(4-(1*H*-imidazol-1-yl)butyl)-2-((2-methylpyrrolidin-1-yl)methyl)phenol (11): To a round-bottom flask was added paraformaldehyde (8.3 mg, 0.28 mmol), (*R*)-2-methylpyrrolidine (0.03 mL, 0.28 mmol), toluene (5 mL), and ethanol (1 mL). The reaction mixture was then heated to 60 ^{o}C and stirred for 1 hour. The reaction mixture was then cooled to room temperature upon which 3-(4-(1*H*-imidazol-1-yl)butyl)phenol (40 mg, 0.18 mmol) was added in one portion. The reaction was then set to stir at 110 ^{o}C for 16 hours. Upon completion, the solution was concentrated *in vacuo,* and the residue was purified via silica gel chromatography (9:1 DCM:MeOH; 1% NEt_3), resulting in the title compound as a yellow oil (33.0 mg, 0.10 mmol, 57%). ^{1}H NMR ($CDCl_3$, 400MHz) δ_H: 7.41 (s, 1H), 7.01 (s, 1H), 6.87 - 6.83 (m, 2H), 6.57 (d, *J* = 1.6 Hz, 1H), 6.53 - 6.49 (m, 1H), 4.18 (d, *J* = 13.8 Hz, 1H), 3.89 (t, *J* = 7.1 Hz, 2H), 3.29 (d, *J* = 13.9 Hz, 1H), 3.05 - 2.97 (m, 1H), 2.58 - 2.47 (m, 3H), 2.21 (q, *J* = 9.8 Hz, 1H), 2.07 - 1.97 (m, 1H), 1.84 - 1.65 (m, 4H), 1.63 - 1.54 (m, 2H), 1.54 - 1.42 (m, 1H), 1.19 (d, *J* = 6.1 Hz, 3H); ^{13}C NMR ($CDCl_3$, 100MHz) δ_C: 157.9, 142.0, 137.1, 129.4, 127.8, 120.4, 118.9, 118.8, 115.8, 60.0, 56.6, 54.0, 46.9, 35.0, 32.9, 30.6, 28.1, 21.8, 18.9; MS-ESI Calculated: 314.2232, Experimental: 314.2227 $(M+H)^+$, 157.6149 (2+), Difference: 0.0005 (1.6 ppm); Purity (HPLC): 100%.

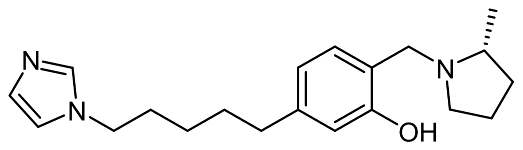

12

(*R*)-5-(5-(1*H*-imidazol-1-yl)pentyl)-2-((2-methylpyrrolidin-1-yl)methyl)phenol (12): To a round-bottom flask was added paraformaldehyde (20 mg, 0.65 mmol), (*R*)-2-methylpyrrolidine (0.07 mL, 0.65 mmol), toluene (5 mL), and ethanol (1 mL). The reaction mixture was then heated to 60 °C and stirred for 1 hour. The reaction mixture was then cooled to room temperature upon which 3-(5-(1*H*-imidazol-1-yl)pentyl)phenol (100 mg, 0.43 mmol) was added in one portion. The reaction was then set to stir at 110 °C for 16 hours. Upon completion, the solution was concentrated *in vacuo,* and the residue was purified via silica gel chromatography (9:1 DCM:MeOH; 1% NEt_3), resulting in the title compound as a yellow oil (53.0 mg, 0.16 mmol, 37%). ^{1}H NMR ($CDCl_3$, 400MHz) δ_H: 7.40 (s, 1H), 7.01 (s, 1H), 6.87 - 6.82 (m, 2H), 6.58 (d, J = 1.5 Hz, 1H), 6.54 - 6.50 (m, 1H), 4.18 (d, J = 13.8 Hz, 1H), 3.86 (t, J = 7.2 Hz, 2H), 3.28 (d, J = 13.9 Hz, 1H), 3.05 - 2.97 (m, 1H), 2.57 - 2.45 (m, 3H), 2.20 (q, J = 9.8 Hz, 1H), 2.06 - 1.95 (m, 1H), 1.82 - 1.64 (m, 4H), 1.64 - 1.54 (m, 2H), 1.53 - 1.42 (m, 1H), 1.35 - 1.25 (m, 2H), 1.18 (d, J = 6.1 Hz, 3H); ^{13}C NMR ($CDCl_3$, 100MHz) δ_C: 157.8, 142.6, 137.0, 129.3, 127.6, 120.1, 118.8, 118.7, 115.7, 59.9, 56.6, 53.9, 46.8, 35.3, 32.8, 31.0, 30.6, 26.1, 21.7, 18.8; MS-ESI Calculated: 328.2389, Experimental: 328.2388 $(M+H)^+$, 164.6228 (2+), Difference: 0.0001 (0.3 ppm); Purity (HPLC): 100%.

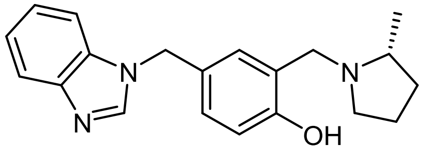

13

(*R*)-4-((1*H*-benzo[d]imidazol-1-yl)methyl)-2-((2-methylpyrrolidin-1-yl)methyl)phenol (13): To a pressure flask equipped with a magnetic stir bar was added paraformaldehyde (40.1 mg, 1.34 mmol), (*R*)-2-methylpyrrolidine (0.13 mL, 1.34 mmol), toluene (5 mL) and ethanol (1 mL). The mixture was then stirred at 60 °C for 1 hour, upon which the solution was cooled to room temperature and 4-((1*H*-benzo[*d*]imidazol-1-yl)methyl)phenol (100 mg, 0.57 mmol) was added. The reaction mixture was then stirred at 120 °C for 16 hours. Upon completion, the solution was then concentrated *in vacuo* and the residue was then purified via silica gel chromatography (12 g ReadySep Column, 100% dichloromethane ramp to 95:5 DCM:MeOH) across 7 minutes. The product was eluted in 95:5 DCM:MeOH, resulting in the title compound as a white solid (71.0 mg, 0.22 mmol, 49%). ^{1}H NMR ($CDCl_3$, 400 MHz) δ_H: 7.88 (s, 1H), 7.83 - 7.77 (m, 1H), 7.34 - 7.20 (m, 3H), 7.03 (dd, J = 8.2, 2.0 Hz, 1H), 6.78 (d, J = 2.0 Hz, 1H), 6.76 (d, J = 8.2 Hz, 1H), 5.19 (s, 2H), 4.14 (d, J = 14.0 Hz, 1H), 3.25 (d, J = 14.1 Hz, 1H), 3.03 - 2.95 (m, 1H), 2.57 - 2.47 (m, 1H), 2.19 (q, J = 9.7 Hz, 1H), 2.06 - 1.95 (m, 1H), 1.83 - 1.65 (m, 2H), 1.54 - 1.42 (m, 1H), 1.17 (d, J = 6.1 Hz, 3H); ^{13}C NMR ($CDCl_3$, 100 MHz) δ_C: 158.1, 144.0, 143.1, 134.0, 127.8, 127.0, 125.4, 123.3, 122.9, 122.1, 120.3, 116.3, 110.1, 60.0, 56.6, 53.9, 48.6, 32.7, 21.8, 18.8; MS-ESI Calculated: 322.1919, Experimental: 322.1920 $(M+H)^+$, Difference: 0.0001 (0.3 ppm); MS-ESI Calculated (para-QM formation): 204.1388, Experimental: 204.1385 $(C_{13}H_{18}NO)^+$, Difference: 0.0003 (0.9 ppm); Purity (HPLC): 98%.

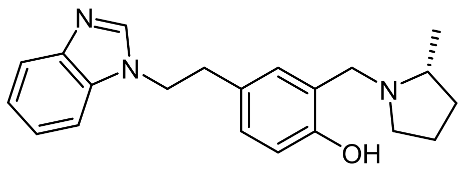

14

(*R*)-4-(2-(1*H*-benzo[*d*]imidazol-1-yl)ethyl)-2-((2-methylpyrrolidin-1-yl)methyl)phenol (14): To a pressure tube equipped with a magnetic stir bar was added paraformaldehyde (38 mg, 1.26 mmol), (*R*)-2-methylpyrrolidine (0.13 mL, 1.26 mmol), toluene (5 mL), and ethanol (1 mL). The tube was then sealed, and the reaction was stirred at 60 ℃ for 2 hours. The reaction mixture was then cooled to room temperature and 4-(2-(1*H*-benzo[*d*]imidazol-1-yl)ethyl)phenol (100 mg, 0.42 mmol) was added. The tube was then sealed and stirred at 110 ℃ for 18 hours. The reaction mixture was then concentrated *in vacuo* and purified by silica gel chromatography (12 g ReadySep Column, 99% dichloromethane with 1% triethylamine). The product was eluted in 99% dichloromethane with 1% triethylamine, resulting in the title compound as a clear oil (74 mg, 0.22 mmol, 52%). ^{1}H NMR ($CDCl_3$, 400 MHz) δ_H: 7.81 (m, 1H), 7.62 (s, 1H), 7.34 - 7.22 (m, 3H), 6.88 (dd, *J* = 8.2, 2.0 Hz, 1H), 6.72 (d, *J* = 8.2 Hz, 1H), 6.43 (d, *J* = 2.1 Hz, 1H), 4.39 - 4.27 (m, 2H), 4.10 (d, *J* = 13.9 Hz, 1H), 3.13 (d, *J* = 13.9 Hz, 1H), 3.01 (t, *J* = 6.9 Hz, 2H), 2.97 - 2.89 (m, 1H), 2.54 - 2.44 (m, 1H), 2.12 (q, *J* = 9.8 Hz, 1H), 2.06 - 1.95 (m, 1H), 1.83 - 1.65 (m, 2H), 1.54 - 1.42 (m, 1H), 1.17 (d, *J* = 6.1 Hz, 3H); ^{13}C NMR ($CDCl_3$, 100 MHz) δ_C: 157.0, 143.9, 143.1, 133.7, 128.5, 128.2, 127.7, 123.1, 122.8, 122.0, 120.4, 116.3, 109.6, 60.0, 56.8, 53.9, 47.1, 35.7, 32.9, 21.8, 18.9; MS-ESI Calculated: 336.2076, Experimental: 336.2077 $(M+H)^+$, Difference: 0.0001 (0.3 ppm); Purity (HPLC): 100%.

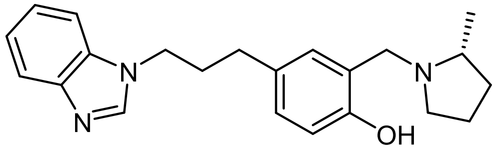

15

***(R)*-4-(3-(1*H*-benzo[*d*]imidazol-1-yl)propyl)-2-((2-methylpyrrolidin-1-yl)methyl)phenol (15):** To a pressure tube equipped with a magnetic stir bar was added paraformaldehyde (36 mg, 1.19 mmol), (*R*)-2-methylpyrrolidine (0.12 mL, 1.19 mmol), toluene (5 mL), and ethanol (1 mL). The tube was then sealed and the reaction was stirred at 60 ℃ for 2 hours. The reaction mixture was then cooled to room temperature and 4-(3-(1*H*-benzo[*d*]imidazol-1-yl)propyl)phenol (100 mg, 0.40 mmol) was added. The tube was then sealed and stirred at 110 ℃ for 18 hours. The reaction mixture was then concentrated *in vacuo* and purified by silica gel chromatography (24 g ReadySep Column, 99% dichloromethane, 1% triethylamine ramp to 95:5 DCM:MeOH with 1% triethylamine). The product was eluted in 98:2 DCM:MeOH with 1% triethylamine, resulting in the title compound as a clear oil (49 mg, 0.14 mmol, 35%). ^{1}H NMR ($CDCl_3$, 400 MHz) δ_H: 7.86 (s, 1H), 7.84 - 7.79 (m, 1H), 7.38 - 7.25 (m, 3H), 6.95 (dd, *J* = 8.2, 2.0 Hz, 1H), 6.77 - 6.72 (m, 2H), 4.22 (d, *J* = 13.9 Hz, 1H), 4.15 (t, *J* = 7.0 Hz, 2H), 3.29 (d, *J* = 13.9 Hz, 1H), 3.08 - 3.01 (m, 1H), 2.61 - 2.50 (m, 3H), 2.29 - 2.14 (m, 3H), 2.10 - 1.99 (m, 1H), 1.86 - 1.68 (m, 2H), 1.57 - 1.46 (m, 1H), 1.22 (d, *J* = 6.1 Hz, 3H); ^{13}C NMR ($CDCl_3$, 100 MHz) δ_C: 156.3, 143.8, 142.9, 133.7, 130.2, 128.1, 127.8, 122.8, 122.1, 120.4, 116.0, 109.7, 60.0, 56.9, 54.0, 50.5, 44.2, 32.8, 31.8, 31.1, 21.8, 18.9; MS-ESI Calculated: 350.2232, Experimental: 350.2235 $(M+H)^+$, Difference: 0.0003 (0.9 ppm); Purity (HPLC): 100%.

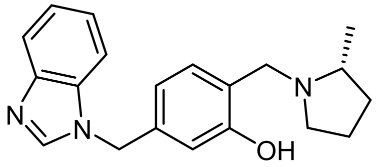

16

(*R*)-5-((1*H*-benzo[*d*]imidazol-1-yl)methyl)-2-((2-methylpyrrolidin-1-yl)methyl)phenol (16): To a pressure tube equipped with a magnetic stir bar was added paraformaldehyde (40 mg, 1.34 mmol), (*R*)-2-methylpyrrolidine (0.14 mL, 1.34 mmol), toluene (5 mL), and ethanol (1 mL). The tube was then sealed, and the reaction was stirred at 60 °C for 2 hours. The reaction mixture was then cooled to room temperature and 3-((1*H*-benzo[*d*]imidazol-1-yl)methyl)phenol (100 mg, 0.45 mmol) was added. The tube was then sealed and stirred at 110 °C for 18 hours. The reaction mixture was then concentrated *in vacuo* and purified by silica gel chromatography (12 g ReadySep Column, 99% dichloromethane with 1% triethylamine). The product was eluted in 99% dichloromethane with 1% triethylamine, resulting in the title compound as a clear oil (15 mg, 0.05 mmol, 10%). ^{1}H NMR ($CDCl_3$, 400 MHz) δ_H: 7.85 (s, 1H), 7.76 - 7.70 (m, 1H), 7.27 - 7.22 (m, 1H), 7.21 - 7.13 (m, 2H), 6.83 (d, *J* = 7.6 Hz, 1H), 6.60 (d, *J* = 1.7 Hz, 1H), 6.46 (dd, *J* = 7.6, 1.7 Hz, 1H), 5.18 (s, 2H), 4.12 (d, *J* = 14.0 Hz, 1H), 3.25 (d, *J* = 14.1 Hz, 1H), 2.98 - 2.89 (m, 1H), 2.53 - 2.41 (m, 1H), 2.15 (q, *J* = 9.8 Hz, 1H), 2.01 - 1.90 (m, 1H), 1.77 - 1.58 (m, 2H), 1.48 - 1.36 (m, 1H), 1.12 (d, *J* = 6.1 Hz, 3H); ^{13}C NMR ($CDCl_3$, 100 MHz) δ_C: 158.6, 144.0, 143.3, 136.0, 134.1, 128.4, 123.0, 122.8, 122.2, 120.4, 117.5, 114.9, 110.2, 60.1, 56.6, 54.0, 48.7, 32.8, 21.8, 18.8; MS-ESI Calculated: 322.1919, Experimental: 322.1921 $(M+H)^+$, Difference: 0.0002 (0.6 ppm); Purity (HPLC): 100%.

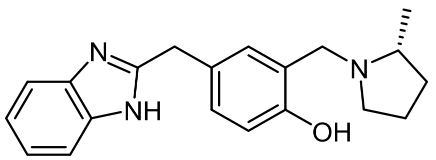

17

(*R*)-4-((1*H*-benzo[*d*]imidazol-2-yl)methyl)-2-((2-methylpyrrolidin-1-yl)methyl)phenol (17): To a pressure tube equipped with a magnetic stir bar was added paraformaldehyde (40 mg, 1.34 mmol), (*R*)-2-methylpyrrolidine (0.13 mL, 1.34 mmol), toluene (5 mL), and ethanol (1 mL). The tube was then sealed and the reaction was stirred at 60 ℃ for 2 hours. The reaction mixture was then cooled to room temperature, and 4-((1*H*-benzo[*d*]imidazol-2-yl)methyl)phenol (100 mg, 0.45 mmol) was added. The tube was then sealed and stirred at 110 ℃ for 18 hours. The reaction mixture was then concentrated *in vacuo* and purified by silica gel chromatography (12 g ReadySep Column, 99% dichloromethane with 1% triethylamine, and then ramp to 8:2 DCM:MeOH with 1% triethylamine). The product was eluted in 9:1 DCM:MeOH with 1% triethylamine, resulting in the title compound as a clear oil (22 mg, 0.07 mmol, 15%). ^{1}H NMR ($CDCl_3$, 400 MHz) δ_H: 7.55 - 7.46 (m, 2H), 7.22 - 7.14 (m, 2H), 7.05 - 6.95 (m, 2H), 6.72 (d, *J* = 8.1 Hz, 1H), 4.12 (s, 2H), 4.08 (d, *J* = 13.9 Hz, 1H), 3.30 (d, *J* = 13.8 Hz, 1H), 3.07 - 2.98 (m, 1H), 2.68 - 2.54 (m, 1H), 2.28 (q, *J* = 9.2 Hz, 1H), 2.09 - 1.97 (m, 1H), 1.86 - 1.65 (m, 2H), 1.59 - 1.46 (m, 1H), 1.20 (d, *J* = 6.1 Hz, 3H); ^{13}C NMR ($CDCl_3$, 100 MHz) δ_C: 157.0, 154.3, 138.5, 129.37, 129.32, 126.6, 122.3, 116.3, 115.0, 114.9, 60.6, 55.9, 53.8, 35.1, 32.6, 21.7, 18.5; MS-ESI Calculated: 322.1919, Experimental: 322.1922 $(M+H)^+$, Difference: 0.0003 (0.9 ppm); Purity (HPLC): 100%.

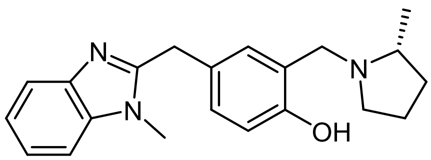

18

(*R*)-4-((1-methyl-1*H*-benzo[d]imidazol-2-yl)methyl)-2-((2-methylpyrrolidin-1-yl)methyl)phenol (18): To a pressure tube equipped with a magnetic stir bar was added paraformaldehyde (38 mg, 1.26 mmol), (*R*)-2-methylpyrrolidine (0.13 mL, 1.26 mmol), toluene (5 mL), and ethanol (1 mL). The tube was then sealed, and the reaction was stirred at 60 °C for 2 hours. The reaction mixture was then cooled to room temperature, and 4-((1-methyl-1*H*-benzo[*d*]imidazol-2-yl)methyl)phenol (100 mg, 0.42 mmol) was added. The tube was then sealed and stirred at 110 °C for 18 hours. The reaction mixture was then concentrated *in vacuo* and purified by silica gel chromatography (24 g ReadySep Column, 100% hexanes ramp to 100% ethyl acetate). The product was eluted in 100% ethyl acetate, resulting in the title compound as a clear oil (47 mg, 0.14 mmol, 33%). ^{1}H NMR ($CDCl_3$, 400 MHz) δ_H: 7.78 - 7.73 (m, 1H), 7.28 - 7.21 (m, 3H), 7.04 - 6.99 (m, 1H), 6.82 (d, *J* = 1.8 Hz, 1H), 6.72 (d, *J* = 8.2 Hz, 1H), 4.21 - 4.18 (m, 2H), 4.14 (d, *J* = 14.0 Hz, 1H), 3.57 (s, 3H), 3.25 (d, *J* = 14.0 Hz, 1H), 3.03 - 2.95 (m, 1H), 2.56 - 2.46 (m, 1H), 2.19 (q, *J* = 9.8 Hz, 1H), 2.06 - 1.95 (m, 1H), 1.83 - 1.63 (m, 2H), 1.53 - 1.42 (m, 1H), 1.17 (d, *J* = 6.1 Hz, 3H); ^{13}C NMR ($CDCl_3$, 100 MHz) δ_C: 156.9, 153.9, 142.5, 136.1, 128.4, 127.8, 126.1, 123.2, 122.2, 121.8, 119.4, 116.1, 109.0, 60.0, 56.7, 53.9, 33.7, 32.8, 30.1, 21.8, 18.8; MS-ESI Calculated: 336.2076, Experimental: 336.2078 $(M+H)^+$, Difference: 0.0002 (0.6 ppm); Purity (HPLC): 96%.

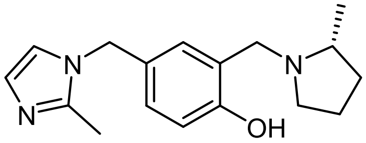

19

(*R*)-4-((2-methyl-1*H*-imidazol-1-yl)methyl)-2-((2-methylpyrrolidin-1-yl)methyl)phenol (19): To a pressure flask equipped with a magnetic stir bar was added paraformaldehyde (47.8 mg, 1.59 mmol), *(R)*-2-methylpyrrolidine (0.16 mL, 1.59 mmol), toluene (5 mL) and ethanol (1 mL). The mixture was then stirred at 60 ℃ for 1 hour, upon which the solution was cooled to room temperature, and 4-((2-methyl-1*H*-imidazol-1-yl)methyl)phenol (100 mg, 0.53 mmol) was added. The reaction mixture was then stirred at 120 ℃ for 16 hours. Upon completion, the solution was then concentrated *in vacuo* and the residue was then purified via silica gel chromatography (12 g ReadySep Column, 100% dichloromethane ramp to 9:1 DCM:MeOH) across 10 minutes. The product was eluted in 9:1 DCM:MeOH, resulting in the title compound as a tan oil (78.0 mg, 0.27 mmol, 51%). ^{1}H NMR ($CDCl_3$, 400 MHz) δ_H: 6.92 - 6.87 (m, 2H), 6.79 (d, *J* = 1.3 Hz, 1H), 6.74 (d, *J* = 8.2 Hz, 1H), 6.64 (d, *J* = 2.1 Hz, 1H), 4.90 (s, 2H), 4.17 (d, *J* = 14.0 Hz, 1H), 3.28 (d, *J* = 14.1 Hz, 1H), 3.04 - 2.96 (m, 1H), 2.59 - 2.48 (m, 1H), 2.33 (s, 3H), 2.21 (q, *J* = 9.8 Hz, 1H), 2.08 - 1.97 (m, 1H), 1.85 - 1.67 (m, 2H), 1.55 - 1.44 (m, 1H), 1.19 (d, *J* = 6.1 Hz, 3H); ^{13}C NMR ($CDCl_3$, 100 MHz) δ_C: 157.9, 144.8, 127.3, 127.1, 126.5, 126.4, 123.3, 119.8, 116.3, 60.1, 56.8, 54.0, 49.5, 32.8, 21.8, 18.9, 13.3; MS-ESI Calculated: 286.1919, Experimental: 289.1916 $(M+H)^+$, Difference: 0.0003 (0.9 ppm); MS-ESI Calculated (para-QM formation): 204.1388, Experimental: 204.1383 $(C_{13}H_{18}NO)^+$, Difference: 0.0005 (1.5 ppm); Purity (HPLC): 99%.

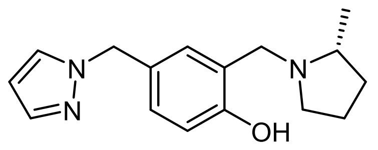

20

(*R*)-4-((1*H*-pyrazol-1-yl)methyl)-2-((2-methylpyrrolidin-1-yl)methyl)phenol (20): To a pressure tube equipped with a magnetic stir bar was added paraformaldehyde (26 mg, 0.86 mmol), (*R*)-2-methylpyrrolidine (0.09 mL, 0.86 mmol), toluene (5 mL), and ethanol (1 mL). The tube was then sealed, and the reaction was stirred at 60 °C for 2 hours. The reaction mixture was then cooled to room temperature and 4-((1*H*-pyrazol-1-yl)methyl)phenol (100 mg, 0.57 mmol) was added. The tube was then sealed and stirred at 110 °C for 18 hours. The reaction mixture was then concentrated *in vacuo* and purified by silica gel chromatography (12 g ReadySep Column, 99% dichloromethane 1% triethylamine, ramp to 95:5 DCM:MeOH with 1% triethylamine) across 7 minutes. The product was eluted in 95:5 DCM:MeOH with 1% triethylamine, resulting in the title compound as a clear oil (47 mg, 0.17 mmol, 30%). ^{1}H NMR ($CDCl_3$, 400 MHz) δ_H: 7.50 (d, *J* = 1.4 Hz, 1H), 7.31 (d, *J* = 2.1 Hz, 1H), 7.02 (dd, *J* = 8.2, 2.0 Hz, 1H), 6.86 (d, *J* = 1.9 Hz, 1H), 6.74 (d, *J* = 8.2 Hz, 1H), 6.22 (t, *J* = 2.1 Hz, 1H), 5.17 (s, 2H), 4.18 (d, *J* = 14.0 Hz, 1H), 3.29 (d, *J* = 14.0 Hz, 1H), 3.04 - 2.96 (m, 1H), 2.58 - 2.48 (m, 1H), 2.21 (q, *J* = 9.8 Hz, 1H), 2.08 - 1.96 (m, 1H), 1.84 - 1.65 (m, 2H), 1.54 - 1.43 (m, 1H), 1.18 (d, *J* = 6.1 Hz, 3H); ^{13}C NMR ($CDCl_3$, 100 MHz) δ_C: 157.9, 139.3, 128.8, 128.2, 127.6, 126.7, 123.0, 116.1, 105.7, 60.0, 56.8, 55.6, 53.9, 32.8, 21.8, 18.8; MS-ESI Calculated: 272.1763, Experimental: 272.1762 $(M+H)^+$, Difference: 0.0001 (0.3 ppm); Purity (HPLC): 100%.

Synthesis and Characterization of Intermediate Materials

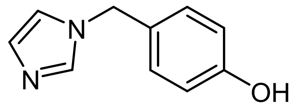

21

4-((1*H*-imidazol-1-yl)methyl)phenol (21): To a pressure flask equipped with a magnetic stir bar was added 4-(hydroxymethyl)phenol (5.00 g, 40.3 mmol) and imidazole (5.48 g, 80.5 mmol). The reaction was then stirred at 90 °C for 3 hours. The reaction was then allowed to cool to room temperature, and the solid residue was then filtered and washed using ethyl acetate (50 mL), resulting in the title compound as a tan solid (6.25 g, 35.9 mmol, 89%). ^{1}H NMR (DMSO, 400 MHz) δ_H: 9.47 (s, 1H), 7.68 (s, 1H), 7.13 - 7.07 (m, 3H), 6.86 (t, J = 1.0 Hz, 1H), 6.74 - 6.69 (m, 2H), 5.03 (s, 2H); ^{13}C NMR (DMSO, 100 MHz) δ_C: 156.9, 137.0, 129.0, 128.5, 127.9, 119.2, 115.2, 49.0.

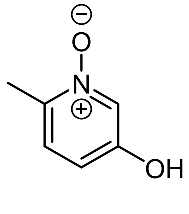

22

5-hydroxy-2-methylpyridine 1-oxide (22): To a round-bottom flask equipped with a magnetic stir bar was added 6-methylpyridin-3-ol (5.00 g, 45.8 mmol), mCPBA (13.5 g, 54.9 mmol), and

chloroform (50 mL). The reaction was stirred at room temperature for 16 hours. The reaction mixture was then concentrated *in vacuo* and the solid residue was recrystallized from ethanol and diethyl ether resulting in the title compound as a yellow solid (4.60 g, 36.8 mmol, 80%). Characterization data were consistent with what has been reported in the literature.[2]

Reference

(2) Shi, Q.; Lee, Y.-J.; Song, H.; Cheng, M.; Jew, S.; Park, H.; Jeong, B.-S. Electronically Modified Polymer-Supported Cinchona Phase-Transfer Catalysts for Asymmetric Synthesis of α-Alkyl-α-Amino Acid Derivatives. *Chem. Lett.* **2008**, *37* (4), 436–437. https://doi.org/10.1246/cl.2008.436.

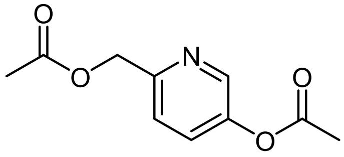

23

6-(acetoxymethyl)pyridin-3-yl acetate (23): To a round-bottom flask equipped with a magnetic stir bar was added 5-hydroxy-2-methylpyridine 1-oxide (4.60 g, 36.8 mmol) and acetic anhydride (34.7 mL, 367 mmol). The reaction mixture was then placed under nitrogen atmosphere and refluxed for 16 hours. Upon completion, the mixture was concentrated *in vacuo* and the residue was then taken into hexanes and triturated, filtering and removing the excess solids. The filtrate was then concentrated *in vacuo*, resulting in the title compound as a tan solid (5.82 g, 27.8 mmol, 76%). ^{1}H NMR ($CDCl_3$, 400 MHz) δ_H: 8.39 (d, J = 2.6 Hz, 1H), 7.48 (dd, J = 8.6, 2.3 Hz, 1H), 7.38 (d, J = 8.6 Hz, 1H), 5.20 (s, 2H), 2.33 - 2.31 (m, 3H), 2.15 - 2.12 (m, 3H); ^{13}C NMR ($CDCl_3$, 100 MHz) δ_C: 170.7, 168.9, 153.0, 146.7, 143.0, 130.0, 122.5, 66.3, 21.08, 21.00.

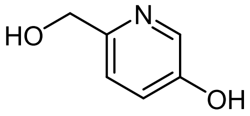

24

6-(hydroxymethyl)pyridin-3-ol (24): To a round-bottom flask equipped with a magnetic stir bar was added 6-(acetoxymethyl)pyridin-3-yl acetate (5.82 g, 27.8 mmol), lithium hydroxide monohydrate (2.33 g, 55.6 mmol), methanol (30 mL) and water (30 mL). The reaction mixture was then stirred at reflux for 1 hour. Upon completion, the reaction mixture was neutralized using hydrochloric acid to pH = 5. The aqueous mixture was then extracted using ethyl acetate (3 x 50 mL), and the organic layers were then combined, dried using sodium sulfate and concentrated *in vacuo*. The crude solid was then used in the next step without further purification.

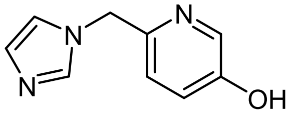

25

6-((1*H*-imidazol-1-yl)methyl)pyridin-3-ol (25): To a pressure flask equipped with a magnetic stir bar was added 6-(hydroxymethyl)pyridin-3-ol (500 mg, 4.00 mmol) and imidazole (1.09 g, 16.0 mmol). The reaction mixture was heated to 120 ℃ and stirred for 2 hours. Upon completion, the reaction was cooled to room temperature and diluted with ethyl acetate (30 mL). The organic layer was then washed with saturated potassium carbonate solution (3 x 30 mL), and the organic

layer was dried over sodium sulfate and concentrated *in vacuo*. The residue was then purified via silica gel chromatography (40 g ReadySep Column, 100% dichloromethane ramp to 8:2 DCM:MeOH) across 20 minutes. The product was eluted in 85:15 DCM:MeOH, resulting in the title compound as a tan solid (149 mg, 0.72 mmol, 18%). ^{1}H NMR ($CDCl_3$, 400 MHz) δ_H: 8.07 (dd, *J* = 2.8 Hz, 0.6 Hz, 1H), 7.69 (s, 1H), 7.16 - 7.11 (m, 2H), 7.09 - 7.05 (m, 1H), 7.02 (s, 1H), 6.88 (s, 1H), 5.14 (s, 2H); ^{13}C NMR ($CDCl_3$, 100 MHz) δ_C: 153.0, 146.9, 137.45, 137.40, 128.4, 122.8, 122.5, 119.5, 50.7.

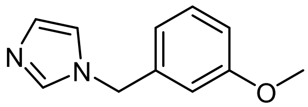

26

1-(3-methoxybenzyl)-1*H*-imidazole (26): To a round-bottom equipped with a magnetic stir bar was added 3-methoxybenzyl chloride (2.78 mL, 19.2 mmol), imidazole (1.30 g, 19.2 mmol), cesium carbonate (6.87 g, 21.1 mmol) and acetonitrile (40 mL). The solution was then stirred at 70 °C for 16 hours. Upon completion, the mixture was diluted with ethyl acetate (50 mL) and washed with saturated potassium carbonate solution (3 x 30 mL). The organic layer was then dried over sodium sulfate and concentrated *in vacuo*. The residue was then purified via silica gel chromatography (40 g ReadySep Column, 100% dichloromethane ramp to 9:1 DCM:MeOH) across 14 minutes. The product was eluted in 94:6 DCM:MeOH, resulting in the title compound as a tan solid (2.43 g, 12.9 mmol, 67%). ^{1}H NMR ($CDCl_3$, 400 MHz) δ_H: 7.48 (s, 1H), 7.21 (t, *J* = 7.9 Hz, 1H), 7.03 (t, *J* = 1.1 Hz, 1H), 6.85 (t, *J* = 1.2 Hz, 1H), 6.82 - 6.78 (m, 1H), 6.71 - 6.67 (m, 1H), 6.63 - 6.60 (m, 1H), 5.02 (s, 2H), 3.71 (s, 3H); ^{13}C NMR ($CDCl_3$, 100 MHz) δ_C: 160.0, 137.7, 137.3, 129.9, 129.6, 119.4, 119.2, 113.4, 112.9, 55.1, 50.6.

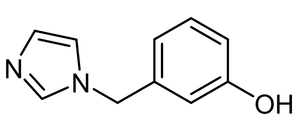

27

3-((1*H*-imidazol-1-yl)methyl)phenol (27): To a pressure flask equipped with a magnetic stir bar was added 1-(3-methoxybenzyl)-1*H*-imidazole (2.43 g, 12.9 mmol) and concentrated hydrobromic acid (15 mL). The solution was then stirred at 120 °C for 1 hour. Upon completion, the mixture was neutralized using saturated potassium carbonate solution (50 mL). The solution was then extracted with ethyl acetate (3 x 30 mL), and the organic layer was washed with brine (30 mL). The organic layer was then dried over sodium sulfate and concentrated *in vacuo*. The residue was then purified via silica gel chromatography (40 g ReadySep Column, 100% dichloromethane ramp to 9:1 DCM:MeOH) across 12 minutes. The product was eluted in 91:9 DCM:MeOH, resulting in the title compound as a tan solid (982 mg, 5.64 mmol, 44%). ^{1}H NMR (DMSO, 400 MHz) δ_H: 7.71 (s, 1H), 7.16 - 7.09 (m, 2H), 6.89 (t, J = 1.0 Hz, 1H), 6.68 - 6.61 (m, 2H), 6.58 - 6.55 (m, 1H), 5.09 (s, 2H); ^{13}C NMR (DMSO, 100 MHz) δ_C: 157.7, 139.1, 137.4, 129.6, 128.5, 119.6, 117.6, 114.6, 114.0, 49.3.

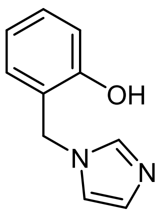

28

2-((1*H*-imidazol-1-yl)methyl)phenol (28): To a round-bottom flask equipped with a magnetic stir bar was added 2-hydroxybenzyl alcohol (1.00 g, 8.06 mmol), and imidazole (822 mg, 12.1 mmol). The mixture was then heated to 90 ℃, upon which a homogeneous melt formed. The reaction was stirred at 90 ℃ for 1 hour and was then cooled to room temperature. The solid formed was dissolved in ethyl acetate (30 mL), then washed with water (3 x 30 mL) and brine (30 mL). The organic layer was then dried over sodium sulfate and concentrated *in vacuo*. The crude residue was carried forward without further purification.

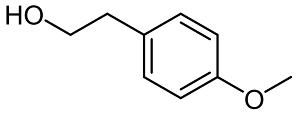

29

2-(4-methoxyphenyl)ethan-1-ol (29): To a dry round-bottom flask under a nitrogen environment equipped with a magnetic stir bar was added 4-methoxyphenylacetic acid (5.0 g, 30.1 mmol), boron trifluoride ethereate (6.1 mL, 49.3 mmol), and tetrahydrofuran (30 mL). The mixture was set to stir vigorously at room temperature and sodium borohydride (1.72 g, 45.4 mmol) was added portion wise and was subsequently stirred at room temperature for 1 hour. The reaction was then quenched using cold water (30 mL) and the aqueous solution was extracted using ethyl acetate (3 x 30 mL). The organic layers were then combined, dried over sodium sulfate, and concentrated *in vacuo* resulting in the title compound as a clear oil (3.45 g, 22.7 mmol, 75%). ^{1}H NMR ($CDCl_3$, 400 MHz) δ_H: 7.16 - 7.10 (m, 2H), 6.88 - 6.82 (m, 2H), 3.81 -

3.74 (m, 5H), 2.78 (t, J = 6.7 Hz, 2H), 2.06 (s, 1H); ^{13}C NMR ($CDCl_3$, 100 MHz) δ_C: 158.2, 130.5, 129.9, 113.9, 63.7, 55.2, 38.2.

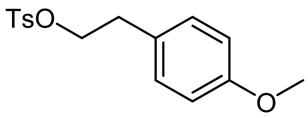

30

4-methoxyphenethyl 4-methylbenzenesulfonate (30): To a round-bottom flask equipped with a magnetic stir bar was added 2-(4-methoxyphenyl)ethan-1-ol (1.50 g, 9.86 mmol), 4-toluenesulfonyl chloride (3.76 g, 19.7 mmol), triethylamine (3.43 mL, 24.6 mmol), and DCM (20 mL). The reaction mixture was then stirred at room temperature for 16 hours. Upon completion, the solution was washed with water (3 x 30 mL) and brine (30 mL). The organic layer was then dried over sodium sulfate, filtered, and concentrated *in vacuo*. The residue was then purified via silica gel chromatography (40 g ReadySep Column, 100% hexanes ramp to 8:2 Hexanes:EtOAc) across 14 minutes. The product was eluted in 85:15 Hexanes:EtOAc, resulting in the title compound as a yellow oil (2.02 g, 6.59 mmol, 67%). ^{1}H NMR ($CDCl_3$, 400 MHz) δ_H: 7.71 - 7.66 (m, 2H), 7.30 - 7.25 (m, 2H), 7.04 - 6.99 (m, 2H), 6.80 - 6.75 (m, 2H), 4.16 (t, J = 7.1 Hz, 2H), 3.77 (s, 3H), 2.88 (t, J = 7.1 Hz, 2H), 2.43 (s, 3H); ^{13}C NMR ($CDCl_3$, 100 MHz) δ_C: 158.6, 144.7, 133.0, 129.9, 129.8, 128.2, 127.9, 114.0, 70.9, 55.3, 34.5, 21.7.

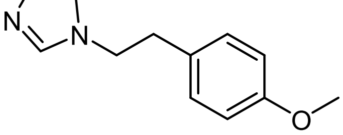

31

1-(4-methoxyphenethyl)-1*H*-imidazole (31): To a round-bottom equipped with a magnetic stir bar was added 4-methoxyphenethyl 4-methylbenzenesulfonate (2.00 g, 6.53 mmol), imidazole (0.44 g, 6.53 mmol), cesium carbonate (1.89 g, 9.79 mmol) and acetonitrile (10 mL). The solution was then stirred at 70 °C for 16 hours. Upon completion, the mixture was diluted with ethyl acetate (50 mL) and washed with saturated potassium carbonate solution (3 x 30 mL). The organic layer was then dried over sodium sulfate and concentrated *in vacuo*. The residue was then purified via silica gel chromatography (40 g ReadySep Column, 100% dichloromethane ramp to 9:1 DCM:MeOH) across 8 minutes. The product was eluted in 92:8 DCM:MeOH, resulting in the title compound as a tan solid (467 mg, 2.31 mmol, 35%). ^{1}H NMR ($CDCl_3$, 400 MHz) δ_H: 7.27 (s, 1H), 7.01 (s, 1H), 6.97 - 6.91 (m, 2H), 6.83 - 6.77 (m, 3H), 4.10 (t, J = 7.0 Hz, 2H), 3.76 (s, 3H), 2.96 (t, J = 7.0 Hz, 2H); ^{13}C NMR ($CDCl_3$, 100 MHz) δ_C: 158.6, 137.1, 129.6, 129.5, 129.4, 118.8, 114.1, 55.2, 48.7, 37.0.

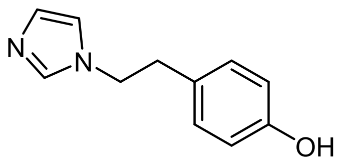

32

4-(2-(1*H*-imidazol-1-yl)ethyl)phenol (32): To a pressure flask equipped with a magnetic stir bar was added 1-(4-methoxyphenethyl)-1*H*-imidazole (467 mg, 2.31 mmol) and concentrated hydrobromic acid (5 mL). The solution was then stirred at 120 °C for 1 hour. Upon completion,

the mixture was neutralized using saturated potassium carbonate solution (10 mL) resulting in a brown precipitate. The precipitate was then filtered and washed with water, resulting in the title compound as a tan solid (288 mg, 1.53 mmol, 66%). ^{1}H NMR (DMSO, 400 MHz) δ_H: 9.24 (s, 1H), 7.47 (s, 1H), 7.11 (s, 1H), 6.97 - 6.91 (m, 2H), 6.83 (s, 1H), 6.67 - 6.62 (m, 2H), 4.11 (t, J = 7.3 Hz, 2H), 2.88 (t, J = 7.3 Hz, 2H); ^{13}C NMR (DMSO, 100 MHz) δ_C: 155.8, 137.1, 129.5, 128.18, 128.14, 119.1, 115.0, 47.5, 36.0.

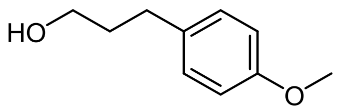

33

3-(4-methoxyphenyl)propan-1-ol (33): To a dry round-bottom flask under a nitrogen environment equipped with a magnetic stir bar was added 3-(4-methoxyphenyl)propanoic acid (3.00 g, 16.6 mmol), boron trifluoride ethereate (3.39 mL, 27.4 mmol), and tetrahydrofuran (16 mL). The mixture was stirred vigorously at room temperature, and then sodium borohydride (944 mg, 24.9 mmol) was added portion wise and was subsequently stirred at room temperature for 1 hour. The reaction was then quenched using cold water (30 mL), and the aqueous solution was extracted using ethyl acetate (3 x 30 mL). The organic layers were then combined, dried over sodium sulfate, and concentrated *in vacuo* resulting in the title compound as a clear oil (2.59 g, 15.6 mmol, 94%). ^{1}H NMR ($CDCl_3$, 400 MHz) δ_H: 7.14 - 7.09 (m, 2H), 6.86 - 6.81 (m, 2H), 3.78 (s, 3H), 3.66 (t, J = 6.4 Hz, 2H), 2.65 (t, J = 7.6 Hz, 2H), 1.90 - 1.81 (m, 2H); ^{13}C NMR ($CDCl_3$, 100 MHz) δ_C: 157.8, 133.9, 129.4, 113.9, 62.3, 55.3, 34.5, 31.2.

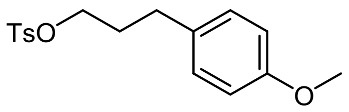

34

3-(4-methoxyphenyl)propyl 4-methylbenzenesulfonate (34): To a round-bottom flask equipped with a magnetic stir bar was added 3-(4-methoxyphenyl)propan-1-ol (2.50 g, 15.0 mmol), 4-toluenesulfonyl chloride (5.73 g, 30.1 mmol), triethylamine (2.31 mL, 16.5 mmol), and DCM (150 mL). The reaction mixture was then stirred at room temperature for 16 hours. Upon completion, the solution was washed with water (3 x 30 mL) and brine (30 mL). The organic layer was then dried over sodium sulfate, filtered, and concentrated *in vacuo*. The residue was then purified via silica gel chromatography (80 g ReadySep Column, 100% hexanes ramp to 1:1 Hexanes:EtOAc) across 12 minutes. The product was eluted in 7:3 Hexanes:EtOAc, resulting in the title compound as a clear oil (3.67 g, 11.4 mmol, 76%). ^{1}H NMR ($CDCl_3$, 400 MHz) δ_H: 7.78 (d, J = 8.3 Hz, 2H), 7.34 (d, J = 8.3 Hz, 2H), 7.00 - 6.95 (m, 2H), 6.80 - 6.74 (m, 2H), 4.01 (t, J = 6.2 Hz, 2H), 3.77 (s, 3H), 2.58 (t, J = 7.5 Hz, 2H), 1.96 - 1.87 (m, 2H); ^{13}C NMR ($CDCl_3$, 100 MHz) δ_C: 158.0, 144.8, 133.2, 132.4, 129.9, 129.4, 128.0, 113.9, 69.7, 55.3, 30.7, 30.6, 21.7.

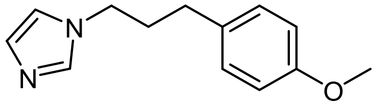

35

1-(3-(4-methoxyphenyl)propyl)-1*H*-imidazole (35): To a round-bottom equipped with a magnetic stir bar was added 3-(4-methoxyphenyl)propyl 4-methylbenzenesulfonate (1.50 g, 4.68 mmol), imidazole (0.35 g, 5.15 mmol), cesium carbonate (1.68 g, 5.15 mmol),

tetrabutylammonium iodide (86 mg, 0.23 mmol), and acetonitrile (10 mL). The solution was then stirred at 70 ℃ for 1 hour. Upon completion, the mixture was diluted with ethyl acetate (50 mL) and washed with saturated potassium carbonate solution (3 x 30 mL). The organic layer was then dried over sodium sulfate and concentrated *in vacuo*. The residue was then purified via silica gel chromatography (40 g ReadySep Column, 100% dichloromethane ramp to 9:1 DCM:MeOH) across 10 minutes. The product was eluted in 94:6 DCM:MeOH, resulting in the title compound as a white solid (512 mg, 2.37 mmol, 51%). ^{1}H NMR (DMSO, 400 MHz) δ_H: 7.61 (s, 1H), 7.17 (s, 1H), 7.12 - 7.06 (m, 2H), 6.89 (s, 1H), 6.86 - 6.81 (m, 2H), 3.93 (t, *J* = 7.1 Hz, 2H), 3.70 (s, 3H), 2.48 - 2.41 (m, 2H), 2.02 - 1.92 (m, 2H); ^{13}C NMR (DMSO, 100 MHz) δ_C: 157.5, 137.2, 132.7, 129.1, 128.3, 119.2, 113.7, 54.9, 45.5, 32.3, 31.1

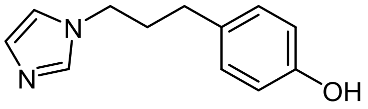

36

4-(3-(1*H*-imidazol-1-yl)propyl)phenol (36): To a round-bottom flask equipped with a magnetic stir bar and reflux condenser was added 1-(3-(4-methoxyphenyl)propyl)-1*H*-imidazole (512 mg, 2.37 mmol), and concentrated hydrobromic acid (5 mL). The reaction was then placed under a nitrogen atmosphere and stirred at reflux for 1 hour. Upon completion, the reaction mixture was cooled to room temperature and 1M sodium hydroxide solution was added until pH = 7 was reached. This resulted in a white solid precipitating from solution, and the white solid was then collected via filtration as the title compound (240 mg, 1.19 mmol, 50%). ^{1}H NMR (DMSO, 400 MHz) δ_H: 7.60 (s, 1H), 7.17 (s, 1H), 6.98 - 6.92 (m, 2H), 6.88 (s, 1H), 6.69 - 6.63 (m, 2H), 3.92

(t, J = 7.1 Hz, 2H), 2.39 (t, J = 8.2 Hz, 2H), 2.00 - 1.89 (m, 2H); ^{13}C NMR (DMSO, 100 MHz) δ_C: 155.6, 137.2, 130.7, 129.0, 128.3, 119.2, 115.1, 45.5, 32.4, 31.1.

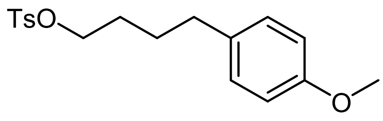

37

4-(4-methoxyphenyl)butyl 4-methylbenzenesulfonate (37): To a round-bottom flask equipped with a magnetic stir bar was added 4-(4-methoxyphenyl)butan-1-ol (250 mg, 1.39 mmol), tosyl chloride (528 mg, 2.77 mmol), triethylamine (0.39 mL, 2.77 mmol), and dichloromethane (15 mL). The reaction mixture was then stirred at room temperature for 16 hours. Upon completion, the organic layer was washed with water (3 x 30 mL) and brine (30 mL). The organic layer was then dried over sodium sulfate and concentrated *in vacuo*. The residue was then purified via silica gel chromatography (8:2 Hexanes:EtOAc), resulting in the title compound as a colorless oil (270 mg, 0.81 mmol, 58%). ^{1}H NMR ($CDCl_3$, 400MHz) δ_H: 7.80 - 7.75 (m, 2H), 7.35 - 7.30 (m, 2H), 7.04 - 6.99 (m, 2H), 6.83 - 6.77 (m, 2H), 4.02 (t, J = 6.1 Hz, 2H), 3.78 (s, 3H), 2.50 (t, J = 7.2 Hz, 2H), 2.44 (s, 3H), 1.70 - 1.53 (m, 4H); ^{13}C NMR ($CDCl_3$, 100MHz) δ_C: 157.9, 144.7, 133.7, 133.3, 129.9, 129.3, 127.9, 113.9, 70.5, 55.3, 34.2, 28.4, 27.4, 21.7.

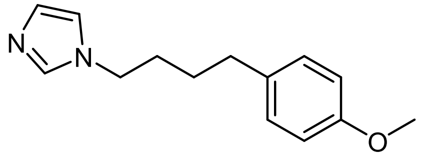

38

1-(4-(4-methoxyphenyl)butyl)-1*H*-imidazole (38): To a round-bottom flask equipped with a magnetic stir bar was added 4-(4-methoxyphenyl)butyl 4-methylbenzenesulfonate (250 mg, 0.75 mmol), cesium carbonate (487 mg, 1.50 mmol), imidazole (101 mg, 1.50 mmol), TBAI (13 mg, 0.04 mmol), and acetonitrile (4 mL). The reaction mixture was then stirred at 80 ^{o}C for 1 hour. Upon completion, the reaction mixture was filtered to remove salts, concentrated *in vacuo*, and purified via silica gel chromatography (DCM:MeOH), resulting in the title compound as a colorless oil (125 mg, 0.54 mmol, 73%). ^{1}H NMR ($CDCl_3$, 400MHz) δ_H: 7.44 (s, 1H), 7.07 - 7.02 (m, 3H), 6.87 (s, 1H), 6.84 - 6.79 (m, 2H), 3.91 (t, *J* = 7.1 Hz, 2H), 3.78 (s, 3H), 2.56 (t, *J* = 7.5 Hz, 2H), 1.82 - 1.73 (m, 2H), 1.63 - 1.53 (m, 2H); ^{13}C NMR ($CDCl_3$, 100MHz) δ_C: 158.0, 137.1, 133.6, 129.5, 129.3, 118.9, 113.9, 55.3, 47.0, 34.4, 30.6, 28.5.

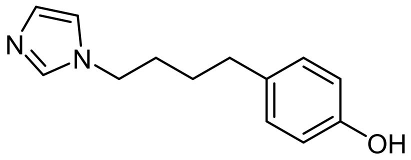

39

4-(4-(1*H*-imidazol-1-yl)butyl)phenol (39): To a round-bottom flask equipped with a magnetic stir bar was added 1-(4-(4-methoxyphenyl)butyl)-1*H*-imidazole (120 mg, 0.52 mmol), and hydrobromic acid (2 mL). The reaction was then stirred at reflux for 1 hour. Upon completion, the reaction mixture was cooled to room temperature and neutralized to pH 7, upon which ethyl acetate (50 mL) was added. The organic layer was then removed, and the reaction mixture was made slightly basic (pH 9), extracted with ethyl acetate (50 mL), and then made slightly acidic (pH 5) followed by extraction with ethyl acetate. The organic layers were then combined and washed with brine (50 mL), subsequently dried over sodium sulfate, and concentrated *in vacuo*,

resulting in the title compound as a colorless oil (107 mg, 0.49 mmol, 95%). ^{1}H NMR ($CDCl_3$, 400MHz) δ_H: 7.61 (s, 1H), 7.08 (s, 1H), 6.96 - 6.92 (m, 2H), 6.87 (s, 1H), 6.81 - 6.75 (m, 2H), 3.91 (t, J = 7.1 Hz, 2H), 2.53 (t, J = 7.4 Hz, 2H), 1.82 - 1.73 (m, 2H), 1.59 - 1.50 (m, 2H); ^{13}C NMR ($CDCl_3$, 100MHz) δ_C: 155.3, 136.5, 132.4, 129.3, 127.5, 119.2, 115.7, 47.5, 34.4, 30.2, 28.4.

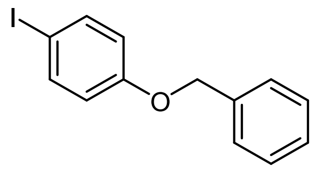

40

1-(benzyloxy)-4-iodobenzene (40): To a round-bottom flask equipped with a magnetic stir bar was added 4-iodophenol (2.00 g, 9.09 mmol), potassium carbonate (3.77 g, 27.2 mmol), benzyl bromide (1.09 mL, 9.18 mmol), and acetone (18 mL). The mixture was then stirred at reflux for 16 hours, upon which the reaction was cooled to room temperature. The reaction mixture was then diluted in ethyl acetate (50 mL) and washed using 6M sodium hydroxide (3 x 30 mL), followed by a brine wash (30 mL). The organic layer was then collected, dried over sodium sulfate, and concentrated *in vacuo*, resulting in the title compound as a white solid (2.47 g, 7.96 mmol, 88%). ^{1}H NMR ($CDCl_3$, 400 MHz) δ_H: 7.59 - 7.54 (m, 2H), 7.44 - 7.31 (m, 5H), 6.79 - 6.73 (m, 2H), 5.03 (s, 2H); ^{13}C NMR ($CDCl_3$, 100 MHz) δ_C: 158.7, 138.3, 136.6, 128.7, 128.2, 127.5, 117.4, 83.1, 70.1.

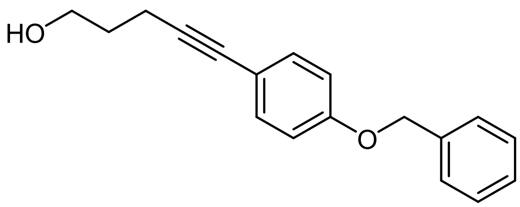

41

5-(4-(benzyloxy)phenyl)pent-4-yn-1-ol (41): To a round-bottom flask equipped with a magnetic stir bar under a nitrogen atmosphere was added 1-(benzyloxy)-4-iodobenzene (3.00 g, 9.67 mmol), 4-pentyn-1-ol (1.08 mL, 11.6 mmol), tetrakis(triphenylphosphine)palladium(0) (558 mg, 5 mol%), copper iodide (92 mg, 5 mol%), triethylamine (40 mL), and dichloromethane (20 mL). The reaction mixture was then stirred at 40 ^{o}C for 16 hours. Upon completion, the reaction mixture was cooled to room temperature and concentrated *in vacuo*. The residue was then diluted with ethyl acetate (50 mL) and washed with water (3 x 30 mL) and brine (30 mL). The organic layer was then dried over sodium sulfate and concentrated *in vacuo*. The residue was then purified via silica gel chromatography (2:8 Hexanes:EtOAc), resulting in the title compound as an orange oil (1.92 g, 7.21 mmol, 75%). ^{1}H NMR ($CDCl_3$, 400MHz) δ_H: 7.44 - 7.29 (m, 7H), 6.91 - 6.86 (m, 2H), 5.05 (s, 2H), 3.82 (t, J = 6.1 Hz, 2H), 2.52 (t, J = 6.9 Hz, 2H), 1.89 - 1.80 (m, 2H); ^{13}C NMR ($CDCl_3$, 100MHz) δ_C: 158.4, 136.8, 133.0, 128.7, 128.1, 127.5, 116.2, 114.9, 87.9, 81.0, 70.1, 62.0, 31.5, 16.1.

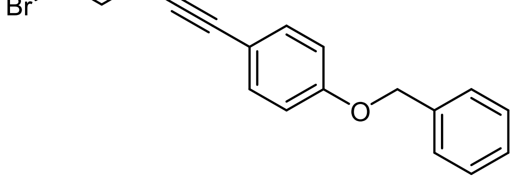

42

1-(benzyloxy)-4-(5-bromopent-1-yn-1-yl)benzene (42): To a round-bottom flask equipped with a magnetic stir bar was added 5-(4-(benzyloxy)phenyl)pent-4-yn-1-ol (1.00 g, 3.96 mmol), carbon tetrabromide (1.64 g, 4.95 mmol), triphenylphosphine (1.30 g, 4.95 mmol), and dichloromethane (20 mL). The reaction mixture was then stirred at room temperature for 16 hours. Upon completion, the reaction mixture was washed with water (30 mL) and brine (30 mL). The organic layer was then dried over sodium sulfate and concentrated *in vacuo*. The residue was then purified via silica gel chromatography (9:1 Hexanes:EtOAc), resulting in the title compound as a yellow oil (860 mg, 2.73 mmol, 69%). ^{1}H NMR ($CDCl_3$, 400MHz) δ_H: 7.44 - 7.32 (m, 7H), 6.92 - 6.88 (m, 2H), 5.06 (s, 2H), 3.51 (t, J = 7.4 Hz, 2H), 2.96 (t, J = 7.4 Hz, 2H); ^{13}C NMR ($CDCl_3$, 100MHz) δ_C: 158.7, 136.7, 133.1, 128.7, 128.1, 127.5, 115.6, 114.9, 85.2, 82.3, 70.1, 29.8, 24.0.

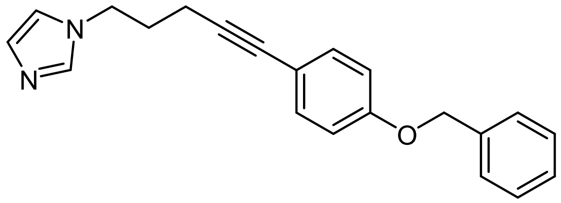

43

1-(5-(4-(benzyloxy)phenyl)pent-4-yn-1-yl)-1*H*-imidazole (43): To a round-bottom flask equipped with a magnetic stir bar was added 1-(benzyloxy)-4-(5-bromopent-1-yn-1-yl)benzene (500 mg, 1.52 mmol), imidazole (206 mg, 3.04 mmol), cesium carbonate (989 mg, 3.04 mmol), TBAI (28 mg, 0.07 mmol), and acetonitrile (8 mL). The reaction mixture was then stirred at 80 °C for 1 hour. Upon completion, the solution was filtered to remove solids, and the filtrate

was concentrated *in vacuo*. The residue was then purified via silica gel chromatography (9:1 DCM:MeOH), resulting in the title compound as a colorless oil (410 mg, 1.30 mmol, 85%).

^{1}H NMR ($CDCl_3$, 400MHz) δ_H: 7.51 (s, 1H), 7.45 - 7.29 (m, 7H), 7.07 (s, 1H), 6.94 (s, 1H), 6.93 - 6.87 (m, 2H), 5.05 (s, 2H), 4.13 (t, J = 6.8 Hz, 2H), 2.38 (t, J = 6.7 Hz, 2H), 2.06 - 1.97 (m, 2H); ^{13}C NMR ($CDCl_3$, 100MHz) δ_C: 158.6, 137.4, 136.7, 133.0, 129.6, 128.7, 128.1, 127.5, 118.9, 115.8, 114.9, 86.1, 82.1, 70.1, 45.5, 29.9, 16.5.

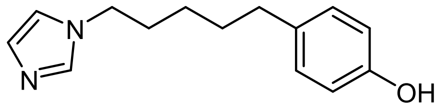

44

4-(5-(1*H*-imidazol-1-yl)pentyl)phenol (44): To a round-bottom flask equipped with a magnetic stir bar was added 1-(5-(4-(benzyloxy)phenyl)pent-4-yn-1-yl)-1*H*-imidazole (300 mg, 0.95 mmol), palladium hydroxide (30 mg, 10 wt%), methanol (5 mL), and ethyl acetate (5 mL). The reaction mixture was then placed under a hydrogen atmosphere (25 psi) and heated to 50 ^{o}C for 16 hours while stirring. Upon completion, the reaction mixture was filtered over celite and concentrated *in vacuo*. The residue was then purified via silica gel chromatography (DCM:MeOH), resulting in the title compound as a white solid (156 mg, 0.68 mmol, 71%).

^{1}H NMR (DMSO, 400MHz) δ_H: 9.12 (br s, 1H), 7.61 (s, 1H), 7.14 (s, 1H), 6.98 - 6.92 (m, 2H), 6.88 (s, 1H), 6.68 - 6.62 (m, 2H), 3.92 (t, J = 7.1 Hz, 2H), 2.43 (t, J = 7.6 Hz, 2H), 1.75 - 1.66 (m, 2H), 1.56 - 1.46 (m, 2H), 1.25 - 1.14 (m, 2H); ^{13}C NMR (DMSO, 100MHz) δ_C: 155.1, 137.0, 132.0, 129.0, 128.0, 119.2, 114.9, 45.8, 34.1, 30.6, 30.3, 25.4.

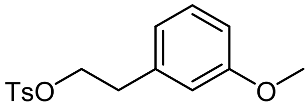

45

3-methoxyphenethyl 4-methylbenzenesulfonate (45): To a round-bottom flask equipped with a magnetic stir bar was added 2-(3-methoxyphenyl)ethan-1-ol (500 mg, 3.29 mmol), tosyl chloride (1.25 g, 6.57 mmol), triethylamine (0.91 mL, 6.57 mmol), and dichloromethane (6 mL). The reaction mixture was then stirred at room temperature for 16 hours. Upon completion, the reaction mixture was diluted with dichloromethane (30 mL) and subsequently extracted with water (3 x 30 mL) and brine (50 mL). The organic layer was then dried over sodium sulfate and concentrated *in vacuo*. The residue was then purified via silica gel chromatography (Hexanes:EtOAc), resulting in the title compound as a colorless oil (780 mg, 2.55 mmol, 77%). ^{1}H NMR ($CDCl_3$, 400MHz) δ_H: 7.71 - 7.66 (m, 2H), 7.30 - 7.25 (m, 2H), 7.16 (t, J = 7.8 Hz, 1H), 6.78 - 6.73 (m, 1H), 6.71 - 6.67 (m, 1H), 6.64 - 6.61 (m, 1H), 4.20 (t, J = 7.1 Hz, 2H), 3.75 (s, 3H), 2.92 (t, J = 7.0 Hz, 2H), 2.42 (s, 3H); ^{13}C NMR ($CDCl_3$, 100MHz) δ_C: 159.7, 144.7, 137.8, 132.9, 129.8, 129.6, 127.8, 121.2, 114.5, 112.3, 70.6, 55.1, 35.4, 21.6.

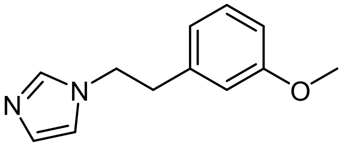

46

1-(3-methoxyphenethyl)-1*H*-imidazole (46): To a round-bottom flask equipped with a magnetic stir bar was added 3-methoxyphenethyl 4-methylbenzenesulfonate (500 mg, 1.63

mmol), imidazole (222 mg, 3.26 mmol), cesium carbonate (1.06 g, 3.26 mmol), TBAI (30 mg, 0.08 mmol), and acetonitrile (8 mL). The reaction mixture was then stirred at 80 °C for 1 hour. Upon completion, the reaction mixture was filtered, and the filter cake was washed with acetonitrile. The filtrate was then concentrated *in vacuo*, and the residue was purified via silica gel chromatography (DCM:MeOH), resulting in the title compound as a yellow oil (250 mg, 1.24 mmol, 76%). ^{1}H NMR ($CDCl_3$, 400MHz) δ_H: 7.31 (s, 1H), 7.19 (t, J = 7.8 Hz, 1H), 7.02 (s, 1H), 6.83 (s, 1H), 6.79 - 6.75 (m, 1H), 6.67 - 6.62 (m, 1H), 6.57 - 6.53 (m, 1H), 4.15 (t, J = 7.1 Hz, 2H), 3.74 (s, 3H), 3.00 (t, J = 7.1 Hz, 2H); ^{13}C NMR ($CDCl_3$, 100MHz) δ_C: 159.9, 139.0, 137.2, 129.8, 129.5, 120.9, 118.8, 114.3, 112.4, 55.2, 48.5, 37.9.

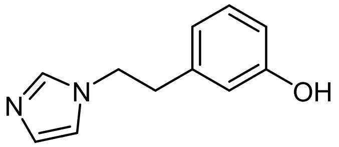

47

3-(2-(1*H*-imidazol-1-yl)ethyl)phenol (47): To a round-bottom flask equipped with a magnetic stir bar was added 1-(3-methoxyphenethyl)-1*H*-imidazole (250 mg, 1.24 mmol) and hydrobromic acid (2.5 mL). The reaction mixture was then stirred at reflux for 1 hour. Upon completion, the solution was neutralized to pH 7, diluted with water (30 mL), and then extracted with ethyl acetate (3 x 30 mL). The organic layer was then dried over sodium sulfate and concentrated *in vacuo*, resulting in the title compound as a white solid (198 mg, 1.05 mmol, 85%). ^{1}H NMR (DMSO, 400MHz) δ_H: 7.54 (s, 1H), 7.15 (s, 1H), 7.05 (t, J = 7.9 Hz, 1H), 6.86 (s, 1H), 6.63 - 6.52 (m, 3H), 4.16 (t, J = 7.3 Hz, 2H), 2.92 (t, J = 7.3 Hz, 2H); ^{13}C NMR (DMSO, 100MHz) δ_C: 157.3, 139.5, 129.2, 119.1, 115.6, 113.3, 47.2, 36.7.

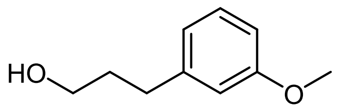

48

3-(3-methoxyphenyl)propan-1-ol (48): To a round-bottom flask equipped with a magnetic stir bar at 0 °C was added 3-(3-methoxyphenyl)propanoic acid (1.00 g, 5.55 mmol), boron trifluoride ethereate (1.12 mL, 9.10 mmol), and THF (5 mL). The solution was then stirred under nitrogen for 10 minutes, upon which sodium borohydride (317 mg, 8.38 mmol) was added portion-wise, resulting in vigorous bubbling. The reaction mixture was then warmed to room temperature and stirred for an additional hour. Upon completion, the reaction mixture was diluted in ethyl acetate (50 mL) and extracted with water (3 x 30 mL) and then brine (30 mL). The organic layer was then dried over sodium sulfate and concentrated *in vacuo*, resulting in the title compound as a colorless oil (909 mg, 5.47 mmol, 98%). ^{1}H NMR ($CDCl_3$, 400MHz) δ_H: 7.23 - 7.17 (m, 1H), 6.82 - 6.71 (m, 3H), 3.79 (s, 3H), 3.66 (t, J = 6.4 Hz, 2H), 2.68 (t, J = 7.7 Hz, 2H), 1.93 - 1.83 (m, 2H); ^{13}C NMR ($CDCl_3$, 100MHz) δ_C: 159.7, 143.5, 129.4, 120.9, 114.3, 111.2, 62.3, 55.2, 34.1, 32.2.

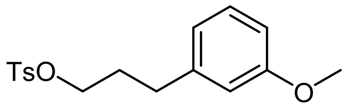

49

3-(3-methoxyphenyl)propyl 4-methylbenzenesulfonate (49): To a round-bottom flask equipped with a magnetic stir bar was added 3-(3-methoxyphenyl)propan-1-ol (900 mg, 5.41

mmol), tosyl chloride (2.06 g, 10.8 mmol), triethylamine (0.83 mL, 5.96 mmol) and dichloromethane (50 mL). The reaction mixture was then stirred at room temperature for 16 hours. Upon completion, the solution was extracted with water (3 x 30 mL) and brine (30 mL). The organic layer was then dried over sodium sulfate and concentrated *in vacuo*. The residue was then purified via silica gel chromatography (Hexanes:EtOAc), resulting in the title compound as a colorless oil (867 mg, 2.71 mmol, 50%). ^{1}H NMR ($CDCl_3$, 400MHz) δ_H: 7.78 (d, J = 8.3 Hz, 2H), 7.33 (d, J = 8.0 Hz, 2H), 7.15 (t, J = 7.8 Hz, 1H), 6.74 - 6.70 (m, 1H), 6.68 - 6.62 (m, 2H), 4.03 (t, J = 6.2 Hz, 2H), 3.77 (s, 3H), 2.62 (t, J = 7.5 Hz, 2H), 2.45 (s, 3H), 1.99 - 1.90 (m, 2H); ^{13}C NMR ($CDCl_3$, 100MHz) δ_C: 159.8, 144.8, 142.0, 133.2, 129.9, 129.5, 127.9, 120.8, 114.2, 111.5, 69.7, 55.2, 31.6, 30.4, 21.7.

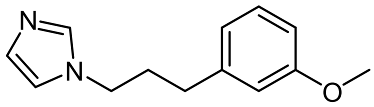

50

1-(3-(3-methoxyphenyl)propyl)-1*H*-imidazole (50): To a round-bottom flask equipped with a magnetic stir bar was added 3-(3-methoxyphenyl)propyl 4-methylbenzenesulfonate (700 mg, 2.18 mmol), imidazole (297 mg, 4.37 mmol), cesium carbonate (1.42 g, 4.37 mmol), TBAI (89 mg, 0.11 mmol), and acetonitrile (10 mL). The reaction mixture was then stirred at 80 ^{o}C for 1 hour. Upon completion, the reaction mixture was filtered, and the filter cake was washed with acetonitrile. The filtrate was then concentrated *in vacuo* and the residue was purified via silica gel chromatography (DCM:MeOH), resulting in the title compound as a yellow oil (190 mg, 0.88 mmol, 40%). ^{1}H NMR ($CDCl_3$, 400MHz) δ_H: 7.45 (s, 1H), 7.21 (t, J = 7.8 Hz, 1H), 7.07 (s, 1H),

6.90 (s, 1H), 6.78 - 6.71 (m, 2H), 6.69 (t, J = 1.9 Hz, 1H), 3.92 (t, J = 7.0 Hz, 2H), 3.79 (s, 3H), 2.58 (t, J = 7.5 Hz, 2H), 2.16 - 2.06 (m, 2H); ^{13}C NMR ($CDCl_3$, 100MHz) δ_C: 159.9, 141.9, 137.2, 129.7, 129.6, 120.8, 118.8, 114.3, 111.5, 55.2, 46.2, 32.5, 32.2.

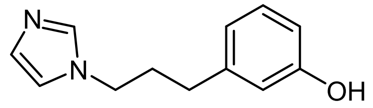

51

3-(3-(1*H*-imidazol-1-yl)propyl)phenol (51): To a round-bottom flask equipped with a magnetic stir bar was added 1-(3-(3-methoxyphenyl)propyl)-1*H*-imidazole (170 mg, 0.78 mmol), and hydrobromic acid (2 mL). The reaction was then stirred at reflux for 1 hour. Upon completion, the reaction mixture was cooled to room temperature and neutralized to pH 7, upon which ethyl acetate (50 mL) was added. The organic layer was then removed, and the remaining aqueous solution was made slightly basic (pH 9), extracted with ethyl acetate (50 mL), and then made slightly acidic (pH 5) followed by extraction with ethyl acetate. The organic layers were then combined and washed with brine (50 mL), subsequently dried over sodium sulfate, and concentrated *in vacuo*, resulting in the title compound as a colorless oil (136 mg, 0.67 mmol, 86%). ^{1}H NMR ($CDCl_3$, 400MHz) δ_H: 7.53 (s, 1H), 7.13 (t, J = 7.8 Hz, 1H), 7.08 (s, 1H), 6.91 (s, 1H), 6.77 - 6.71 (m, 1H), 6.69 - 6.61 (m, 2H), 3.91 (t, J = 6.9 Hz, 2H), 2.52 (t, J = 7.3 Hz, 2H), 2.14 - 2.04 (m, 2H); ^{13}C NMR ($CDCl_3$, 100MHz) δ_C: 157.7, 141.6, 129.8, 119.5, 115.7, 113.9, 46.4, 32.3, 31.9.

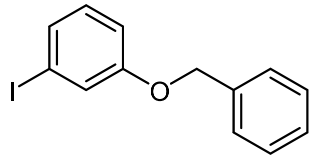

52

1-(benzyloxy)-3-iodobenzene (52): To a round-bottom flask equipped with a magnetic stir bar was added 3-iodophenol (8.00 g, 36.4 mmol), benzyl bromide (4.36 mL, 36.7 mmol), potassium carbonate (15.1 g, 109 mmol), and acetone (72 mL). The reaction mixture was then stirred at reflux for 16 hours. Upon completion, the reaction mixture was cooled to room temperature and concentrated *in vacuo*. The residue was then diluted with ethyl acetate (100 mL) and washed with water (50 mL), followed by 6M sodium hydroxide solution (3 x 50 mL) and brine (30 mL). The organic layer was then dried over sodium sulfate and concentrated *in vacuo*, resulting in the title compound as a white solid (11.3 g, 36.4 mmol, quantitative). ^{1}H NMR ($CDCl_3$, 400MHz) δ_H: 7.48 - 7.32 (m, 7H), 7.03 (t, J = 7.6 Hz, 1H), 6.99 - 6.95 (m, 1H), 5.04 (s, 2H); ^{13}C NMR ($CDCl_3$, 100MHz) δ_C: 159.3, 136.4, 130.8, 130.1, 128.7, 128.2, 127.5, 124.1, 114.5, 94.5, 70.1.

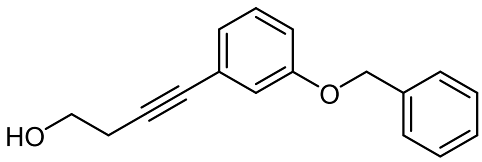

53

4-(3-(benzyloxy)phenyl)but-3-yn-1-ol (53): To a round-bottom flask equipped with a magnetic stir bar under a nitrogen atmosphere was added 1-(benzyloxy)-3-iodobenzene (2.00 g, 6.45

mmol), 3-butyn-1-ol (0.59 mL, 7.74 mmol), tetrakis(triphenylphosphine)palladium(0) (372 mg, 5 mol%), copper iodide (61 mg, 5 mol%), triethylamine (32 mL), and dichloromethane (32 mL). The reaction mixture was then stirred at 30 °C for 16 hours. Upon completion, the reaction mixture was cooled to room temperature and concentrated *in vacuo*. The residue was then diluted with ethyl acetate (50 mL), washed with water (3 x 30 mL) and brine (30 mL). The organic layer was then dried over sodium sulfate and concentrated *in vacuo*. The residue was then purified via silica gel chromatography (2:8 Hexanes:EtOAc), resulting in the title compound as a dark brown oil (1.19 g, 4.72 mmol, 73%). ^{1}H NMR ($CDCl_3$, 400MHz) δ_H: 7.46 - 7.31 (m, 5H), 7.23 - 7.18 (m, 1H), 7.08 - 7.02 (m, 2H), 6.96 - 6.91 (m, 1H), 5.04 (s, 2H), 3.85 - 3.77 (m, 2H), 2.68 (t, *J* = 6.3 Hz, 2H); ^{13}C NMR ($CDCl_3$, 100MHz) δ_C: 158.5, 136.8, 129.4, 128.6, 128.0, 127.5, 124.5, 124.4, 117.6, 115.4, 86.5, 82.3, 70.0, 61.1, 23.8.

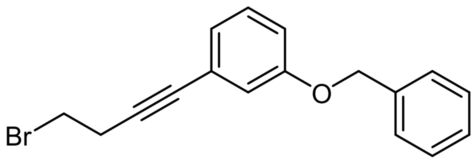

54

1-(benzyloxy)-3-(4-bromobut-1-yn-1-yl)benzene (54): To a round-bottom flask equipped with a magnetic stir bar was added 4-(3-(benzyloxy)phenyl)but-3-yn-1-ol (500 mg, 1.98 mmol), carbon tetrabromide (985 mg, 2.97 mmol), triphenylphosphine (779 mg, 2.97 mmol), and dichloromethane (10 mL). The reaction mixture was then stirred at room temperature for 16 hours. Upon completion, the reaction mixture was washed with water (30 mL) and brine (30 mL). The organic layer was then dried over sodium sulfate and concentrated *in vacuo*. The residue was then purified via silica gel chromatography (9:1 Hexanes:EtOAc), resulting in the

title compound as a yellow oil (310 mg, 0.98 mmol, 50%). ^{1}H NMR ($CDCl_3$, 400MHz) δ_H: 7.45 - 7.30 (m, 5H), 7.23 - 7.18 (m, 1H), 7.06 - 7.01 (m, 2H), 6.96 - 6.91 (m, 1H), 5.05 (s, 2H), 3.52 (t, J = 7.3 Hz, 2H), 2.97 (t, J = 7.3 Hz, 2H); ^{13}C NMR ($CDCl_3$, 100MHz) δ_C: 158.6, 136.8, 129.5, 128.7, 128.1, 127.6, 124.6, 124.2, 117.7, 115.6, 86.6, 82.4, 70.1, 29.6, 24.0.

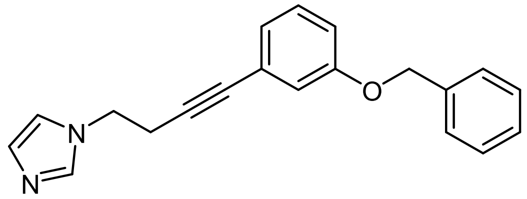

55

1-(4-(3-(benzyloxy)phenyl)but-3-yn-1-yl)-1*H*-imidazole (55): To a round-bottom flask equipped with a magnetic stir bar was added 1-(benzyloxy)-3-(4-bromobut-1-yn-1-yl)benzene (300 mg, 0.95 mmol), imidazole (129 mg, 1.90 mmol), cesium carbonate (620 mg, 1.90 mmol), TBAI (17 mg, 0.05 mmol), and acetonitrile (5 mL). The reaction mixture was then stirred at 60 ^{o}C for 1 hour. Upon completion, the reaction mixture was filtered, and the filter cake was washed with acetonitrile. The filtrate was then concentrated *in vacuo* and the residue was purified via silica gel chromatography (DCM:MeOH), resulting in the title compound as a tan oil (64 mg, 0.21 mmol, 22%). ^{1}H NMR ($CDCl_3$, 400MHz) δ_H: 7.57 (s, 1H), 7.45 - 7.29 (m, 5H), 7.22 - 7.16 (m, 1H), 7.09 (s, 1H), 7.04 - 6.96 (m, 3H), 6.94 - 6.90 (m, 1H), 5.03 (s, 2H), 4.13 (t, J = 6.7 Hz, 2H), 2.81 (t, J = 6.7 Hz, 2H); ^{13}C NMR ($CDCl_3$, 100MHz) δ_C: 158.5, 136.7, 129.6, 129.4, 128.6, 128.0, 127.5, 127.4, 124.4, 123.9, 119.0, 117.5, 115.5, 85.2, 83.2, 70.0, 45.8, 22.6.

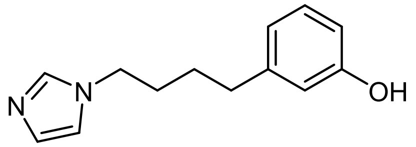

56

3-(4-(1*H*-imidazol-1-yl)butyl)phenol (56): To a round-bottom flask equipped with a magnetic stir bar was added 1-(4-(3-(benzyloxy)phenyl)but-3-yn-1-yl)-1*H*-imidazole (64 mg, 0.21 mmol), palladium hydroxide (6.4 mg, 10 wt%), methanol (2 mL), and ethyl acetate (2 mL). The reaction mixture was then placed under hydrogen atmosphere (20 psi) and stirred at 50 °C for 16 hours. Upon completion, the solution was filtered over celite and concentrated *in vacuo*, resulting in the title compound as a tan oil (43 mg, 0.20 mmol, 94%). ^{1}H NMR (CD_3OD, 400MHz) δ_H: 7.60 (s, 1H), 7.14 - 7.01 (m, 2H), 6.96 (s, 1H), 6.64 - 6.55 (m, 3H), 3.98 (t, *J* = 7.0 Hz, 2H), 2.53 (t, *J* = 7.5 Hz, 2H), 1.82 - 1.71 (m, 2H), 1.59 - 1.49 (m, 2H); ^{13}C NMR (CD_3OD, 100MHz) δ_C: 158.4, 144.7, 130.3, 120.6, 116.2, 113.8, 47.8, 36.1, 31.5, 29.2.

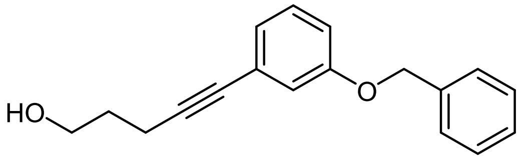

57

5-(3-(benzyloxy)phenyl)pent-4-yn-1-ol (57): To a round-bottom flask equipped with a magnetic stir bar under a nitrogen atmosphere was added 1-(benzyloxy)-3-iodobenzene (2.00 g, 6.45 mmol), 4-pentyn-1-ol (0.72 mL, 7.74 mmol), tetrakis(triphenylphosphine)palladium(0) (372 mg, 5 mol%), copper iodide (61 mg, 5 mol%), triethylamine (32 mL), and dichloromethane (32 mL). The reaction mixture was then stirred at 30 °C for 16 hours. Upon completion, the reaction

mixture was cooled to room temperature and concentrated *in vacuo*. The residue was then diluted with ethyl acetate (50 mL), washed with water (3 x 30 mL) and brine (30 mL). The organic layer was then dried over sodium sulfate and concentrated *in vacuo*. The residue was then purified via silica gel chromatography (2:8 Hexanes:EtOAc), resulting in the title compound as an orange oil (690 mg, 2.59 mmol, 40%). ^{1}H NMR ($CDCl_3$, 400MHz) δ_H: 7.45 - 7.30 (m, 5H), 7.22 - 7.16 (m, 1H), 7.05 - 6.99 (m, 2H), 6.93 - 6.88 (m, 1H), 5.03 (s, 2H), 3.80 (t, J = 6.2 Hz, 2H), 2.53 (t, J = 6.9 Hz, 2H), 1.89 - 1.81 (m, 2H); ^{13}C NMR ($CDCl_3$, 100MHz) δ_C: 158.5, 136.8, 129.3, 128.6, 128.0, 127.5, 124.8, 124.4, 117.5, 115.1, 89.4, 81.0, 70.0, 61.7, 31.4, 16.0.

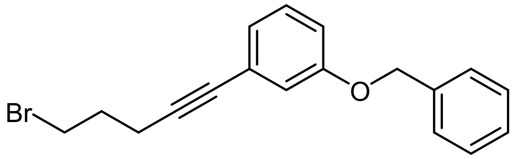

58

1-(benzyloxy)-3-(5-bromopent-1-yn-1-yl)benzene (58): To a round-bottom flask equipped with a magnetic stir bar was added 5-(3-(benzyloxy)phenyl)pent-4-yn-1-ol (500 mg, 1.88 mmol), carbon tetrabromide (933 mg, 2.82 mmol), triphenylphosphine (738 mg, 2.82 mmol), and dichloromethane (10 mL). The reaction mixture was then stirred at room temperature for 16 hours. Upon completion, the reaction mixture was washed with water (30 mL) and brine (30 mL). The organic layer was then dried over sodium sulfate and concentrated *in vacuo*. The residue was then purified via silica gel chromatography (9:1 Hexanes:EtOAc), resulting in the title compound as a yellow oil (384 mg, 1.17 mmol, 62%). ^{1}H NMR ($CDCl_3$, 400MHz) δ_H: 7.45 - 7.30 (m, 5H), 7.22 - 7.17 (m, 1H), 7.04 - 6.99 (m, 2H), 6.94 - 6.89 (m, 1H), 5.05 (s, 2H), 3.58 (t, J = 6.5 Hz, 2H), 2.60 (t, J = 6.8 Hz, 2H), 2.18 - 2.09 (m, 2H); ^{13}C NMR ($CDCl_3$, 100MHz) δ_C:

158.6, 136.8, 129.4, 128.7, 128.1, 127.6, 124.7, 124.5, 117.6, 115.2, 88.0, 81.6, 70.1, 32.5, 31.6, 18.2.

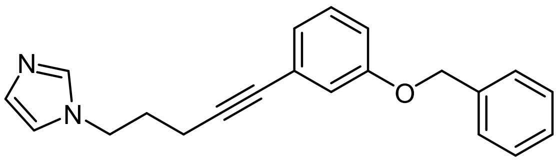

59

1-(5-(3-(benzyloxy)phenyl)pent-4-yn-1-yl)-1*H*-imidazole (59): To a round-bottom flask equipped with a magnetic stir bar was added 1-(benzyloxy)-3-(5-bromopent-1-yn-1-yl)benzene (350 mg, 1.06 mmol), imidazole (144 mg, 2.13 mmol), cesium carbonate (692 mg, 2.13 mmol), TBAI (19 mg, 0.05 mmol), and acetonitrile (5 mL). The reaction mixture was then stirred at 80 ^{o}C for 1 hour. Upon completion, the reaction mixture was filtered, and the filter cake was washed with acetonitrile. The filtrate was then concentrated *in vacuo* and the residue was purified via silica gel chromatography (DCM:MeOH) resulting in the title compound as a colorless oil (280 mg, 0.88 mmol, 83%). ^{1}H NMR ($CDCl_3$, 400MHz) δ_H: 7.52 (s, 1H), 7.45 - 7.29 (m, 5H), 7.24 - 7.18 (m, 1H), 7.08 (s, 1H), 7.04 - 7.00 (m, 2H), 6.98 - 6.90 (m, 2H), 5.05 (s, 2H), 4.13 (t, *J* = 6.8 Hz, 2H), 2.39 (t, *J* = 6.7 Hz, 2H), 2.07 - 1.98 (m, 2H); ^{13}C NMR ($CDCl_3$, 100MHz) δ_C: 158.6, 137.4, 136.8, 129.7, 129.5, 128.7, 128.1, 127.5, 124.4, 118.9, 117.7, 115.3, 87.5, 82.2, 70.1, 45.5, 29.8, 16.4.

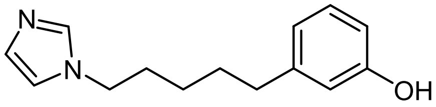

60

3-(5-(1*H*-imidazol-1-yl)pentyl)phenol (60): To a round-bottom flask equipped with a magnetic stir bar was added 1-(5-(3-(benzyloxy)phenyl)pent-4-yn-1-yl)-1*H*-imidazole (280 mg, 0.88 mmol), palladium hydroxide (28 mg, 10 wt%), methanol (10 mL), and ethyl acetate (10 mL). The reaction mixture was then placed under a hydrogen atmosphere (20 psi) and stirred at 50 °C for 16 hours. Upon completion, the reaction mixture was filtered over celite and concentrated *in vacuo*, resulting in the title compound as a white solid (186 mg, 0.81 mmol, 91%). ^{1}H NMR (DMSO, 400MHz) δ_H: 9.22 (s, 1H), 7.58 (s, 1H), 7.13 (s, 1H), 7.06 - 7.00 (m, 1H), 6.86 (s, 1H), 6.60 - 6.52 (m, 3H), 3.92 (t, J = 7.1 Hz, 2H), 2.44 (t, J = 7.6 Hz, 2H), 1.76 - 1.65 (m, 2H), 1.58 - 1.47 (m, 2H), 1.26 - 1.15 (m, 2H); ^{13}C NMR (DMSO, 100MHz) δ_C: 157.2, 143.4, 137.1, 129.0, 128.2, 119.1, 118.8, 115.1, 112.5, 45.8, 35.0, 30.4, 30.2, 25.5.

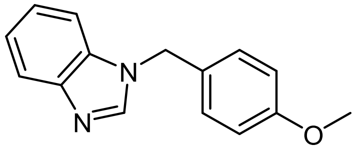

61

1-(4-methoxybenzyl)-1*H*-benzo[*d*]imidazole (61): To a round-bottom equipped with a magnetic stir bar was added 4-methoxybenzyl chloride (0.86 mL, 6.39 mmol), benzimidazole (754 mg, 6.39 mmol), cesium carbonate (2.29 g, 7.02 mmol) and acetonitrile (13 mL). The solution was then stirred at 70 °C for 16 hours. Upon completion, the mixture was diluted with ethyl acetate (50 mL) and washed with saturated potassium carbonate solution (3 x 30 mL). The organic layer was then dried over sodium sulfate and concentrated *in vacuo*. The residue was

then purified via silica gel chromatography (24 g ReadySep Column, 100% hexanes ramp to 100% ethyl acetate) across 15 minutes. The product was eluted in 25:75 Hexanes:EtOAc, resulting in the title compound as a white solid (920 mg, 3.86 mmol, 60%). ^{1}H NMR ($CDCl_3$, 400 MHz) δ_H: 7.90 (s, 1H), 7.83 - 7.79 (m, 1H), 7.32 - 7.21 (m, 3H), 7.15 - 7.10 (m, 2H), 6.88 - 6.83 (m, 2H), 5.27 (s, 2H), 3.77 (s, 3H); ^{13}C NMR ($CDCl_3$, 100 MHz) δ_C: 159.6, 144.1, 143.1, 134.0, 128.7, 127.4, 123.0, 122.2, 120.4, 114.4, 110.1, 55.4, 48.5.

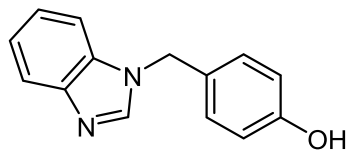

62

4-((1*H*-benzo[*d*]imidazol-1-yl)methyl)phenol (62): To a pressure flask equipped with a magnetic stir bar was added 1-(4-methoxybenzyl)-1*H*-benzo[*d*]imidazole (0.92 g, 3.86 mmol) and concentrated hydrobromic acid (5 mL). The reaction mixture was then stirred at 120 ℃ for 1 hour. Upon completion, the reaction was cooled to room temperature and saturated potassium carbonate (5 mL) was added resulting in a white precipitate. The precipitate was filtered and rinsed with water, resulting in the title compound as a crystalline white solid (0.55 g, 2.46 mmol, 64%). ^{1}H NMR (DMSO, 400 MHz) δ_H: 9.44 (s, 1H), 8.34 (s, 1H), 7.66 - 7.60 (m, 1H), 7.55 - 7.49 (m, 1H), 7.22 - 7.14 (m, 4H), 6.72 - 6.67 (m, 2H), 5.34 (s, 2H); ^{13}C NMR (DMSO, 100 MHz) δ_C: 156.9, 143.9, 143.5, 133.5, 128.9, 127.0, 122.2, 121.4, 119.3, 115.3, 110.7, 47.2.

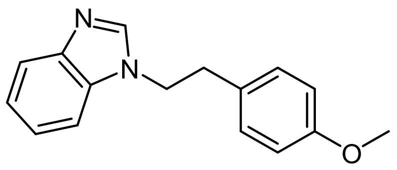

63

1-(4-methoxyphenethyl)-1*H*-benzo[*d*]imidazole (63): To a round-bottom equipped with a magnetic stir bar was added 4-methoxyphenethyl 4-methylbenzenesulfonate (1.07 g, 3.49 mmol), benzimidazole (0.45 g, 3.84 mmol), cesium carbonate (1.25 g, 3.84 mmol), tetrabutylammonium iodide (64 mg, 0.17 mmol), and acetonitrile (10 mL). The solution was then stirred at 70 ℃ for 1 hour. Upon completion, the mixture was diluted with ethyl acetate (50 mL) and washed with saturated potassium carbonate solution (3 x 30 mL). The organic layer was then dried over sodium sulfate and concentrated *in vacuo*. The residue was then purified via silica gel chromatography (40 g ReadySep Column, 100% dichloromethane ramp to 8:2 DCM:MeOH) across 14 minutes. The product was eluted in 92:8 DCM:MeOH, resulting in the title compound as a tan solid (559 mg, 2.22 mmol, 63%). ^{1}H NMR (DMSO, 400 MHz) δ_H: 8.01 (s, 1H), 7.64 - 7.59 (m, 2H), 7.26 - 7.15 (m, 2H), 7.09 - 7.04 (m, 2H), 6.83 - 6.78 (m, 2H), 4.43 (t, *J* = 7.2 Hz, 2H), 3.69 (s, 3H), 3.04 (t, *J* = 7.2 Hz, 2H); ^{13}C NMR (DMSO, 100 MHz) δ_C: 157.8, 143.9, 143.3, 133.6, 129.9, 129.7, 122.1, 121.3, 119.3, 113.7, 110.4, 54.9, 45.6, 34.4.

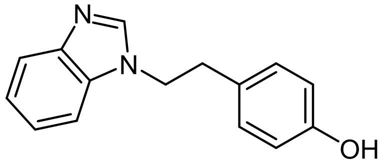

64

4-(2-(1*H*-benzo[*d*]imidazol-1-yl)ethyl)phenol (64): To a round-bottom flask equipped with a magnetic stir bar and reflux condenser was added 1-(4-methoxyphenethyl)-1*H*-benzo[*d*]imidazole (550 mg, 2.18 mmol), and concentrated hydrobromic acid (5 mL). The reaction was then placed under a nitrogen atmosphere and stirred at reflux for 1 hour. Upon completion, the reaction mixture was cooled to room temperature, and 1M sodium hydroxide solution was added until pH = 7 was reached. This resulted in a white solid precipitating, which was then collected via filtration as the title compound (519 mg, 2.18 mmol, quant.). ^{1}H NMR ($CDCl_3$, 400 MHz) δ_H: 9.45 (s, 1H), 8.04 - 7.98 (m, 1H), 7.88 - 7.82 (m, 1H), 7.64 - 7.57 (m, 2H), 6.97 - 6.91 (m, 2H), 6.66 - 6.60 (m, 2H), 4.68 (t, J = 7.2 Hz, 2H), 3.10 (t, J = 7.2 Hz, 2H); ^{13}C NMR ($CDCl_3$, 100 MHz) δ_C: 156.1, 141.4, 130.94, 130.90, 129.7, 126.9, 126.3, 125.9, 115.3, 114.9, 113.3, 47.8, 33.7.

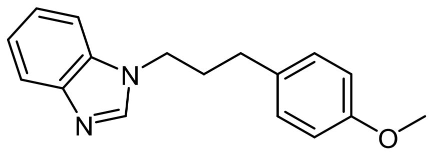

65

1-(3-(4-methoxyphenyl)propyl)-1*H*-benzo[*d*]imidazole (65): To a round-bottom equipped with a magnetic stir bar was added 3-(4-methoxyphenyl)propyl 4-methylbenzenesulfonate (1.50 g, 4.68 mmol), benzimidazole (0.61 g, 5.15 mmol), cesium carbonate (1.68 g, 5.15 mmol), tetrabutylammonium iodide (86 mg, 0.23 mmol), and acetonitrile (10 mL). The solution was then stirred at 70 °C for 1 hour. Upon completion, the mixture was diluted with ethyl acetate (50 mL) and washed with saturated potassium carbonate solution (3 x 30 mL). The organic layer was then

dried over sodium sulfate and concentrated *in vacuo*. The residue was then purified via silica gel chromatography (40 g ReadySep Column, 100% dichloromethane ramp to 8:2 DCM:MeOH) across 14 minutes. The product was eluted in 94:6 DCM:MeOH, resulting in the title compound as a tan solid (760 mg, 2.85 mmol, 61%). ^{1}H NMR (DMSO, 400 MHz) δ_H: 8.23 (s, 1H), 7.68 - 7.64 (m, 1H), 7.58 - 7.54 (m, 1H), 7.28 - 7.18 (m, 2H), 7.13 - 7.07 (m, 2H), 6.86 - 6.81 (m, 2H), 4.25 (t, *J* = 7.1 Hz, 2H), 3.71 (s, 3H), 2.55 - 2.48 (m, 2H), 2.13 - 2.03 (m, 2H); ^{13}C NMR (DMSO, 100 MHz) δ_C: 157.5, 143.9, 143.4, 133.7, 132.7, 129.1, 122.2, 121.3, 119.4, 113.7, 110.3, 54.9, 43.7, 31.29, 31.22.

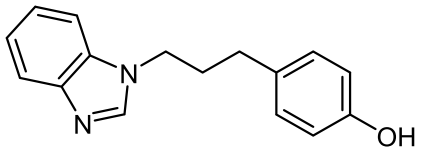

66

4-(3-(1*H*-benzo[*d*]imidazol-1-yl)propyl)phenol (66): To a round-bottom flask equipped with a magnetic stir bar and reflux condenser was added 1-(3-(4-methoxyphenyl)propyl)-1*H*-benzo[*d*]imidazole (761 mg, 2.86 mmol), and concentrated hydrobromic acid (7 mL). The reaction was then placed under a nitrogen atmosphere and stirred at reflux for 1 hour. Upon completion, the reaction mixture was cooled to room temperature and 1M sodium hydroxide solution was added until pH = 7 was reached. This resulted in a white solid precipitating from solution, and the precipitate was then collected via filtration as the title compound (555 mg, 2.20 mmol, 77%). ^{1}H NMR (DMSO, 400 MHz) δ_H: 8.93 (s, 1H), 7.80 - 7.72 (m, 2H), 7.46 - 7.36 (m, 2H), 7.00 - 6.94 (m, 2H), 6.69 - 6.63 (m, 2H), 4.36 (t, *J* = 7.2 Hz, 2H), 2.55 - 2.46 (m, 2H), 2.16

- 2.05 (m, 2H); ^{13}C NMR (DMSO, 100 MHz) δ_C: 155.5, 142.7, 137.4, 132.4, 130.6, 129.0, 124.0, 123.7, 117.2, 115.1, 111.7, 44.9, 31.2, 30.8.

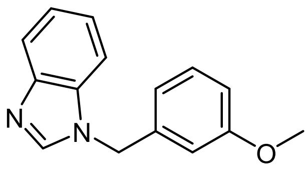

67

1-(3-methoxybenzyl)-1*H*-benzo[*d*]imidazole (67): To a round-bottom equipped with a magnetic stir bar was added 3-methoxybenzyl chloride (0.92 mL, 6.39 mmol), benzimidazole (829 mg, 7.02 mmol), cesium carbonate (2.29 g, 7.02 mmol), tetrabutylammonium iodide (117 mg, 0.32 mmol), and acetonitrile (12 mL). The solution was then stirred at 70 ℃ for 1 hour. Upon completion, the mixture was diluted with ethyl acetate (50 mL) and washed with saturated potassium carbonate solution (3 x 30 mL). The organic layer was then dried over sodium sulfate and concentrated *in vacuo*. The residue was then purified via silica gel chromatography (40 g ReadySep Column, 100% dichloromethane). The product was eluted in 100% DCM, resulting in the title compound as a tan solid (1.35 g, 5.67 mmol, 89%). ^{1}H NMR ($CDCl_3$, 400 MHz) δ_H: 7.96 (s, 1H), 7.83 (d, *J* = 7.2 Hz, 1H), 7.34 - 7.21 (m, 4H), 6.86 - 6.81 (m, 1H), 6.78 - 6.74 (m, 1H), 6.72 - 6.69 (m, 1H), 5.32 (s, 2H), 3.74 (s, 3H); ^{13}C NMR ($CDCl_3$, 100 MHz) δ_C: 160.2, 144.2, 143.4, 143.3, 137.1, 130.2, 123.2, 122.3, 120.5, 119.4, 113.5, 113.0, 110.1, 55.3, 48.9.

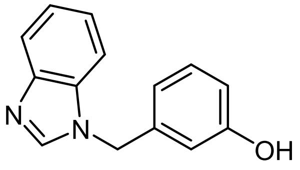

68

3-((1*H*-benzo[*d*]imidazol-1-yl)methyl)phenol (68): To a round-bottom flask equipped with a magnetic stir bar and reflux condenser was added 1-(3-methoxybenzyl)-1*H*-benzo[*d*]imidazole (500 mg, 2.10 mmol), and concentrated hydrobromic acid (5 mL). The reaction was then placed under a nitrogen atmosphere and stirred at reflux for 1 hour. Upon completion, the reaction mixture was cooled to room temperature, and 1M sodium hydroxide solution was added until pH = 7 was reached. This resulted in a white solid precipitating from solution, and the precipitate was then collected via filtration as the title compound (380 mg, 1.69 mmol, 81%). ^{1}H NMR (DMSO, 400 MHz) δ_H: 9.49 (s, 1H), 8.89 (s, 1H), 7.77 - 7.70 (m, 1H), 7.66 - 7.59 (m, 1H), 7.37 - 7.30 (m, 2H), 7.17 - 7.10 (m, 1H), 6.79 - 6.75 (m, 1H), 6.71 - 6.65 (m, 2H), 5.51 (s, 2H); ^{13}C NMR (DMSO, 100 MHz) δ_C: 157.6, 143.4, 139.1, 137.4, 132.7, 129.8, 123.7, 123.3, 118.0, 117.9, 114.9, 114.1, 111.7, 48.2.

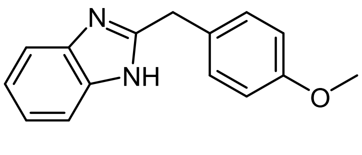

69

2-(4-methoxybenzyl)-1*H*-benzo[*d*]imidazole (69): To a round-bottom flask equipped with a magnetic stir bar was added phenylenediamine (1.00 g, 9.25 mmol), 4-methoxyphenylacetic acid (3.84 g, 23.1 mmol), and 5M hydrochloric acid solution (20 mL). The reaction was then stirred at

reflux for 16 hours. Upon completion, the reaction was placed in an ice bath and cooled to 0 °C. 6M sodium hydroxide solution was then added until pH = 8, wherein a tan precipitate formed in the solution. The tan solid was collected by filtration and washed with water (2 x 30 mL), resulting in the title compound (1.09 g, 4.57 mmol, 49%). ^{1}H NMR (DMSO, 400 MHz) δ_H: 12.25 (s, 1H), 7.58 - 7.33 (m, 2H), 7.28 - 7.21 (m, 2H), 7.13 - 7.06 (m, 2H), 6.90 - 6.84 (m, 2H), 4.09 (s, 2H), 3.70 (s, 3H); ^{13}C NMR (DMSO, 100 MHz) δ_C: 157.9, 153.9, 129.7, 129.5, 121.2, 121.1, 113.8, 55.0, 34.0.

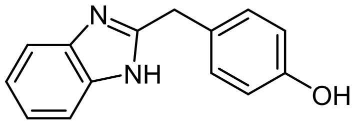

70

4-((1*H*-benzo[*d*]imidazol-2-yl)methyl)phenol (70): To a round-bottom flask equipped with a magnetic stir bar and reflux condenser was added 2-(4-methoxybenzyl)-1*H*-benzo[*d*]imidazole (500 mg, 2.10 mmol), and concentrated hydrobromic acid (5 mL). The reaction was then placed under a nitrogen atmosphere and stirred at reflux for 1 hour. Upon completion, the reaction mixture was cooled to room temperature, and 1M sodium hydroxide solution was added until pH = 7 was reached. This resulted in a tan solid precipitating, which was then collected via filtration as the title compound (463 mg, 2.06 mmol, 98%). ^{1}H NMR (DMSO, 400 MHz) δ_H: 9.51 (s, 1H), 7.80 - 7.72 (m, 2H), 7.56 - 7.49 (m, 2H), 7.26 - 7.19 (m, 2H), 6.82 - 6.75 (m, 2H), 4.40 (s, 2H); ^{13}C NMR (DMSO, 100 MHz) δ_C: 157.0, 153.5, 130.8, 130.3, 125.6, 123.4, 115.7, 113.8, 31.3.

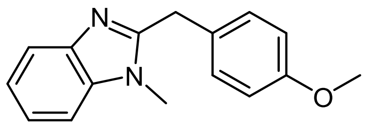

71

2-(4-methoxybenzyl)-1-methyl-1*H*-benzo[*d*]imidazole (71): To a round-bottom flask equipped with a magnetic stir bar was added 2-(4-methoxybenzyl)-1*H*-benzo[*d*]imidazole (500 mg, 2.10 mmol), and anhydrous THF (10 mL). The solution was then placed in an ice bath and cooled to 0 °C, upon which sodium hydride (92 mg, 2.31 mmol) was added. The reaction was then stirred and warmed to room temperature across 10 minutes, upon which methyl iodide (0.14 mL, 2.31 mmol) was added. The reaction was then set to stir at room temperature for 16 hours. Upon completion, the reaction was quenched with water (10 mL) and then diluted in ethyl acetate (30 mL). The organic layer was then washed with water (3 x 30 mL) and then brine (30 mL). The organic layer was then dried over sodium sulfate and concentrated *in vacuo*. The residue was then purified via silica gel chromatography (24 g ReadySep Column, 100% dichloromethane ramp to 95:5 DCM:MeOH) across 6 minutes. The product was eluted in 95:5 DCM:MeOH, resulting in the title compound as a brown oil (447 mg, 1.77 mmol, 84%). ^{1}H NMR ($CDCl_3$, 400 MHz) δ_H: 7.79 - 7.72 (m, 1H), 7.27 - 7.22 (m, 3H), 7.17 - 7.12 (m, 2H), 6.85 - 6.80 (m, 2H), 4.26 (s, 2H), 3.76 (s, 3H), 3.58 (s, 3H); ^{13}C NMR ($CDCl_3$, 100 MHz) δ_C: 158.6, 153.7, 142.6, 136.2, 129.5, 128.2, 122.3, 121.9, 119.5, 114.3, 109.0, 55.3, 33.7, 30.1.

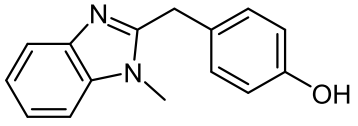

72

4-((1-methyl-1*H*-benzo[*d*]imidazol-2-yl)methyl)phenol (72): To a round-bottom flask equipped with a magnetic stir bar and reflux condenser was added 2-(4-methoxybenzyl)-1-methyl-1*H*-benzo[*d*]imidazole (447 mg, 1.77 mmol), and concentrated hydrobromic acid (5 mL). The reaction was then placed under a nitrogen atmosphere and stirred at reflux for 1 hour. Upon completion, the reaction mixture was cooled to room temperature, and 1M sodium hydroxide solution was added until pH = 7 was reached. This resulted in a tan solid precipitating from solution, and the precipitate was then collected via filtration as the title compound (422 mg, 1.77 mmol, quant.). ^{1}H NMR (DMSO, 400 MHz) δ_H: 9.54 (s, 1H), 7.97 - 7.91 (m, 1H), 7.80 - 7.73 (m, 1H), 7.62 - 7.53 (m, 2H), 7.24 - 7.19 (m, 2H), 6.83 - 6.76 (m, 2H), 4.50 (s, 2H), 3.98 (s, 3H); ^{13}C NMR (DMSO, 100 MHz) δ_C: 157.0, 153.2, 132.5, 130.3, 129.9, 126.0, 125.5, 122.5, 115.8, 114.0, 112.7, 31.2, 30.1.

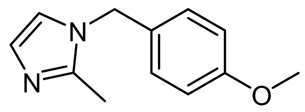

73

1-(4-methoxybenzyl)-2-methyl-1*H*-imidazole (73): To a round-bottom equipped with a magnetic stir bar was added 4-methoxybenzyl chloride (0.86 mL, 6.4 mmol), 2-methylimidazole (524 mg, 6.4 mmol), cesium carbonate (2.29 g, 7.0 mmol) and acetonitrile (13 mL). The solution was then stirred at 70 ℃ for 16 hours. Upon completion, the mixture was diluted with ethyl acetate (50 mL) and washed with saturated potassium carbonate solution (3 x 30 mL). The organic layer was then dried over sodium sulfate and concentrated *in vacuo*. The residue was

then purified via silica gel chromatography (40 g ReadySep Column, 100% dichloromethane ramp to 9:1 DCM:MeOH) across 9 minutes. The product was eluted in 92:8 DCM:MeOH, resulting in the title compound as a tan solid (677 mg, 3.3 mmol, 52%). ^{1}H NMR (DMSO, 400 MHz) δ_H: 7.12 - 7.08 (m, 2H), 7.07 (d, J = 1.3 Hz, 1H), 6.93 - 6.88 (m, 2H), 6.73 (d, J = 1.3 Hz, 1H), 5.03 (s, 2H), 3.72 (s, 3H), 2.22 (s, 3H); ^{13}C NMR (DMSO, 100 MHz) δ_C: 158.6, 143.6, 129.3, 128.4, 126.3, 120.0, 114.0, 55.0, 48.0, 12.7.

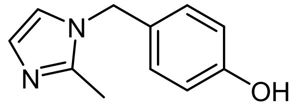

74

4-((2-methyl-1*H*-imidazol-1-yl)methyl)phenol (74): To a pressure flask equipped with a magnetic stir bar was added 1-(4-methoxybenzyl)-2-methyl-1*H*-imidazole (600 mg, 2.97 mmol) and concentrated hydrobromic acid (6 mL). The solution was then stirred at 120 ℃ for 1 hour. Upon completion, the mixture was neutralized using saturated potassium carbonate solution (20 mL), resulting in a brown precipitate. The precipitate was then filtered and washed with water, resulting in the title compound as a tan solid (406 mg, 2.16 mmol, 73%). ^{1}H NMR (DMSO, 400 MHz) δ_H: 9.50 (s, 1H), 7.05 (d, J = 1.3 Hz, 1H), 7.02 - 6.95 (m, 2H), 6.75 - 6.69 (m, 3H), 4.97 (s, 2H), 2.22 (s, 3H); ^{13}C NMR (DMSO, 100 MHz) δ_C: 156.7, 143.6, 128.5, 127.5, 126.1, 120.0, 115.3, 48.2, 12.7.

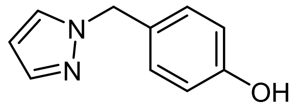

75

4-((1*H*-pyrazol-1-yl)methyl)phenol (75): To a pressure flask equipped with a magnetic stir bar was added 4-hydroxybenzyl alcohol (1.00 g, 8.06 mmol) and pyrazole (2.74 g, 40.3 mmol). The reaction mixture was heated to 110 ℃ and stirred for 16 hours. Upon completion, ethyl acetate (20 mL) was added directly to the hot flask and subsequently extracted with water (3 x 30 mL), and brine (30 mL). The organic layer was then dried over sodium sulfate and concentrated *in vacuo*. The residue was then purified via silica gel chromatography (40 g ReadySep Column, 100% dichloromethane ramp to 9:1 DCM:MeOH) across 10 minutes. The product was eluted in 93:7 DCM:MeOH, and the appropriate fractions were concentrated *in vacuo*. A white solid formed which contained excess pyrazole, which upon addition to water and filtration, resulting in the title compound as a powdery white solid (0.71 g, 4.08 mmol, 51%). ^{1}H NMR (DMSO, 400 MHz) δ_H: 9.40 (s, 1H), 7.73 - 7.71 (m, 1H), 7.42 - 7.40 (m, 1H), 7.09 - 7.04 (m, 2H), 6.73 - 6.67 (m, 2H), 6.22 (t, *J* = 2.0 Hz, 1H), 5.17 (s, 2H); ^{13}C NMR (DMSO, 100 MHz) δ_C: 156.8, 138.6, 129.5, 129.0, 127.8, 115.1, 105.2, 54.3.

Associated NMR and HPLC Spectra

^{1}H NMR ($CDCl_3$, 400 MHz) of 1a

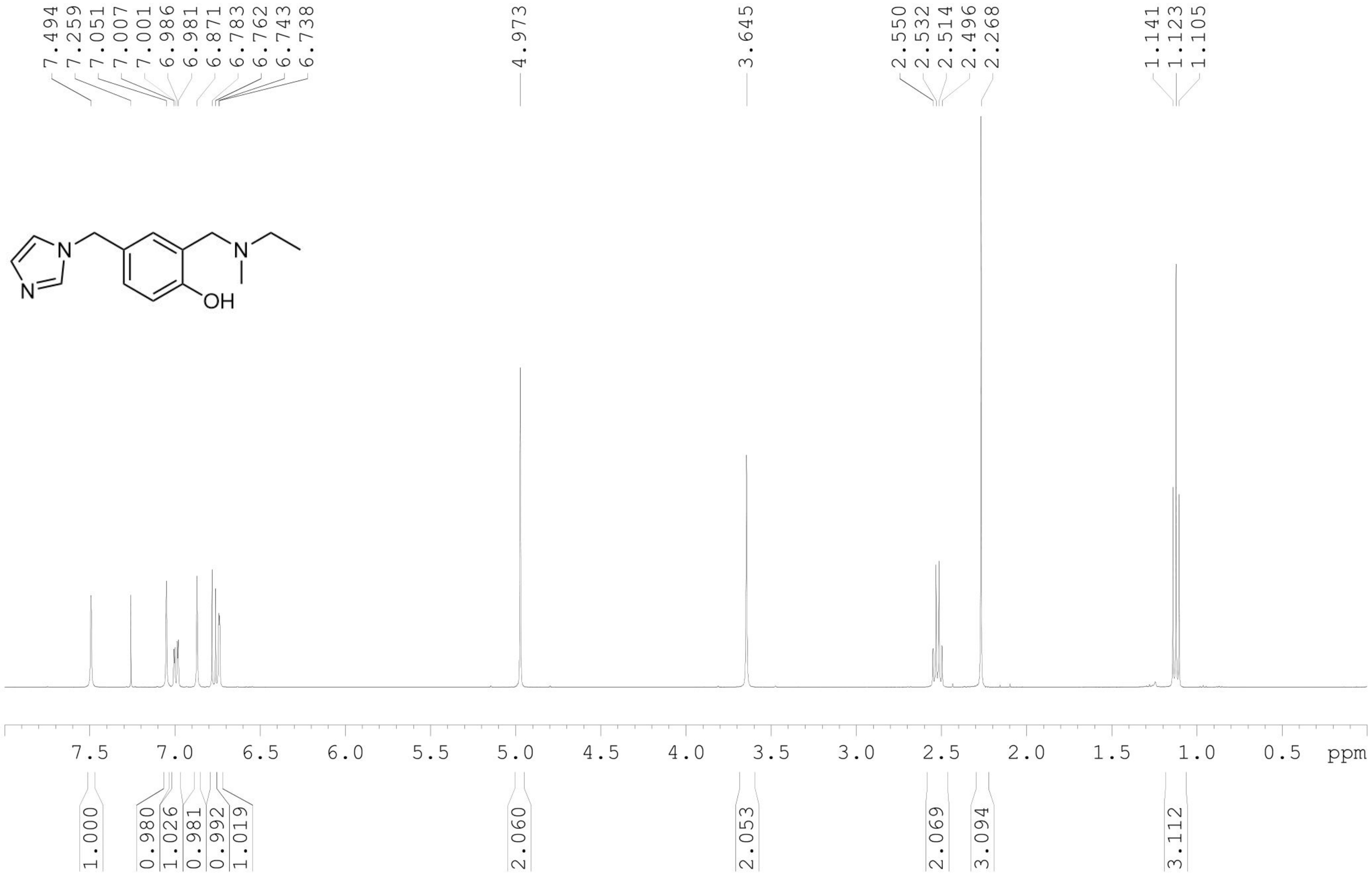

^{13}C NMR ($CDCl_3$, 100 MHz) of 1a

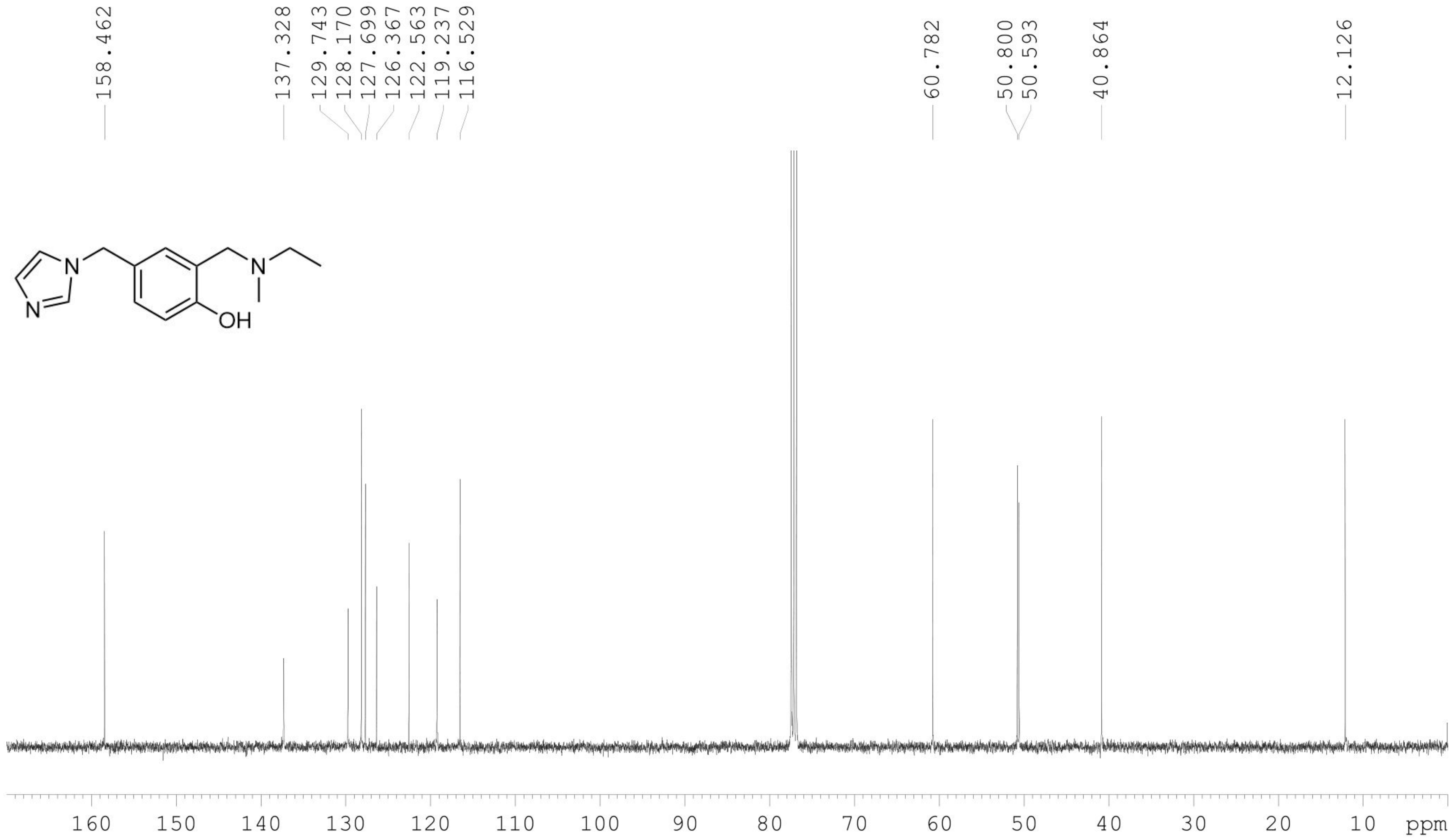

HPLC Trace (254 and 280 nm) of 1a

Waters 2.1 x100 mm C18 UPLC Column

Water/MeOH + 0.1% Triethylamine

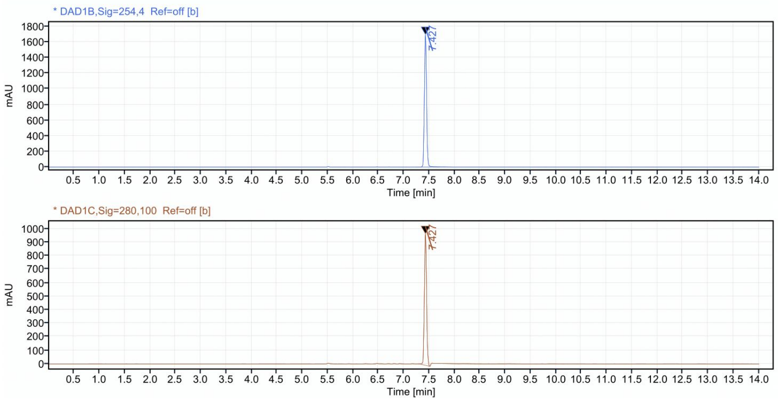

Signal: * DAD1B,Sig=254,4 Ref=off [b]

RT [min]	Peak Area	Type	Width [min]	Area	Height	Area%	Peak Area Percent
7.427	5132.723	BB	0.46	5132.72	1692.59	100.00	100.00
			Sum	5132.72			

Signal: * DAD1C,Sig=280,100 Ref=off [b]

RT [min]	Peak Area	Type	Width [min]	Area	Height	Area%	Peak Area Percent
7.427	2968.525	BB	0.21	2968.52	970.76	100.00	100.00
			Sum	2968.52			

^{1}H NMR ($CDCl_3$, 400 MHz) of 1b

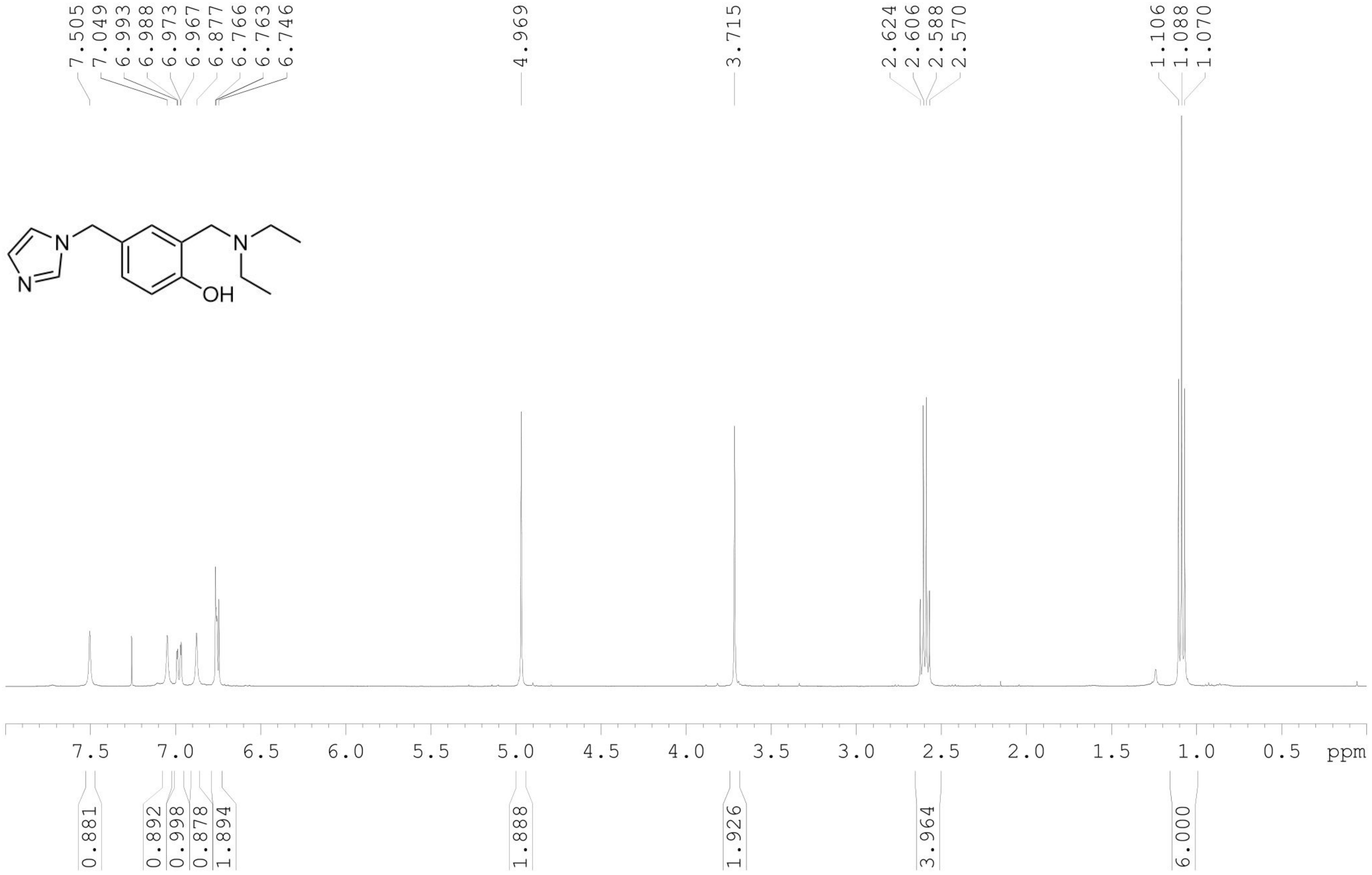

^{13}C NMR ($CDCl_3$, 100 MHz) of 1b

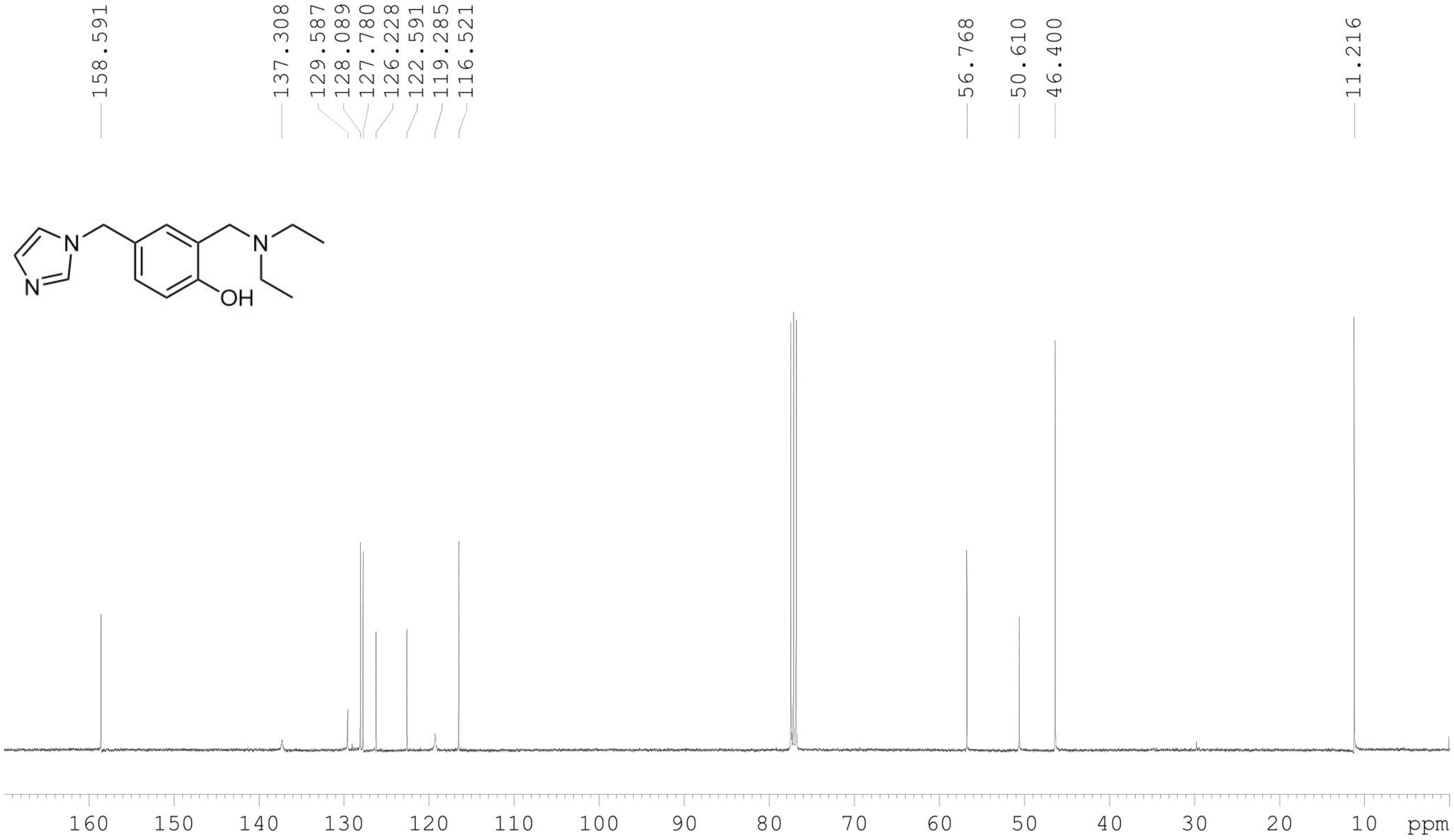

HPLC Trace (254 and 280 nm) of 1b

Waters 2.1 x100 mm C18 UPLC Column

Water/MeOH + 0.1% Triethylamine

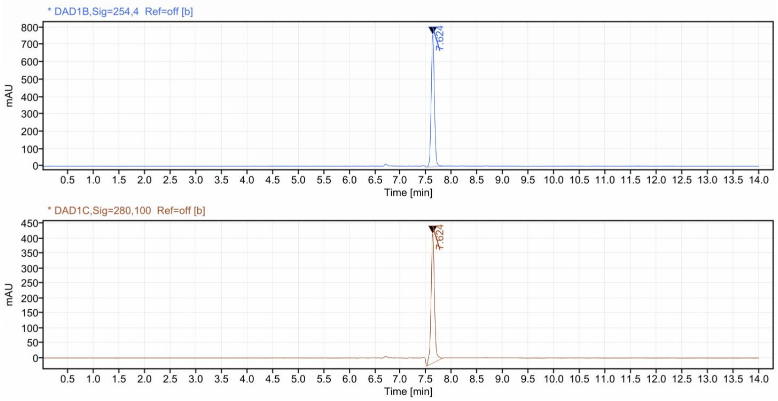

Signal: * DAD1B,Sig=254,4 Ref=off [b]

RT [min]	Peak Area	Type	Width [min]	Area	Height	Area%	Peak Area Percent
7.624	3276.530	BB	0.33	3276.53	760.80	100.00	100.00
			Sum	3276.53			

Signal: * DAD1C,Sig=280,100 Ref=off [b]

RT [min]	Peak Area	Type	Width [min]	Area	Height	Area%	Peak Area Percent
7.624	2005.726	BB	0.30	2005.73	432.83	100.00	100.00
			Sum	2005.73			

^{1}H NMR ($CDCl_3$, 400 MHz) of 1c

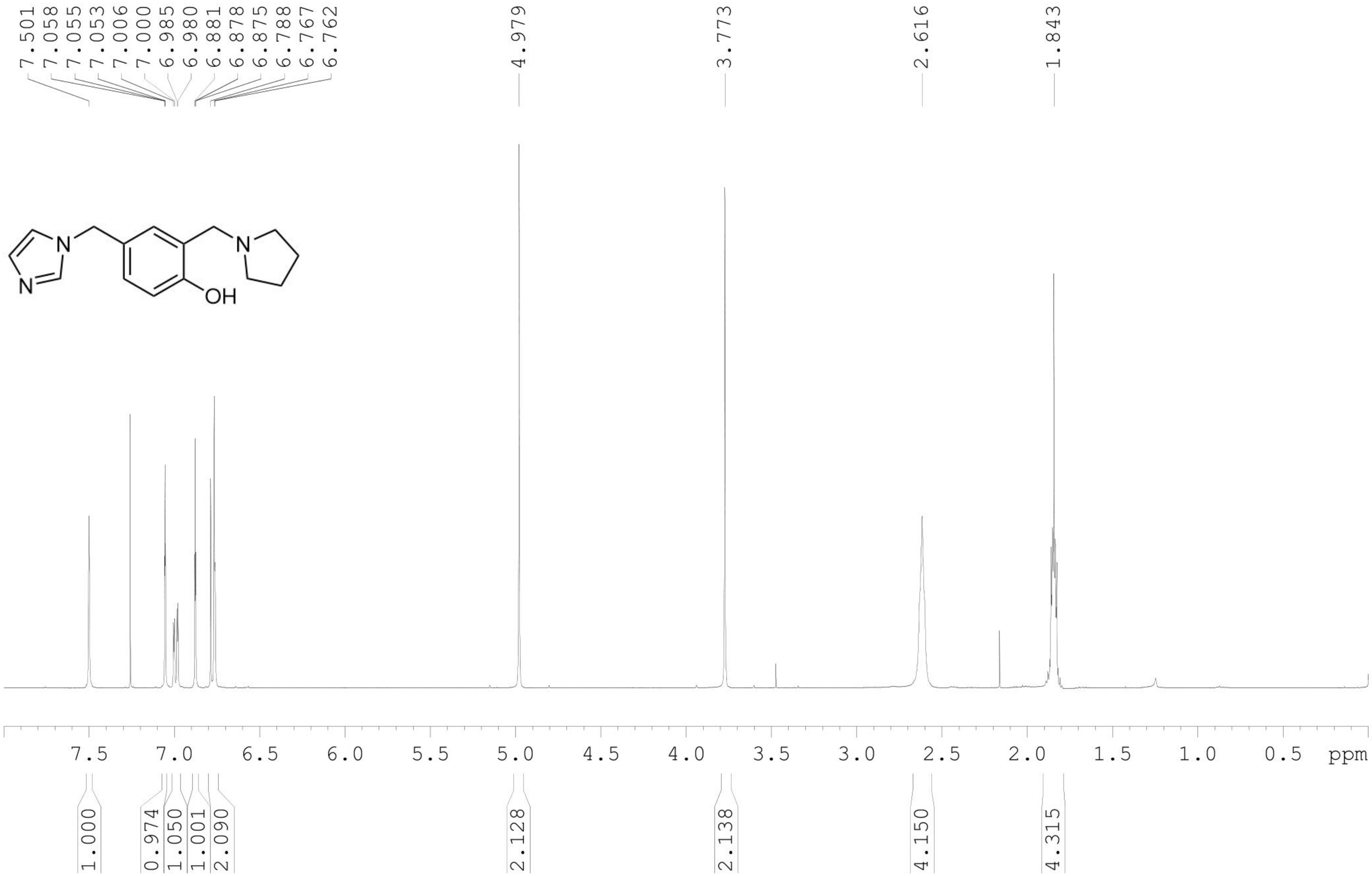

^{13}C NMR ($CDCl_3$, 100 MHz) of 1c

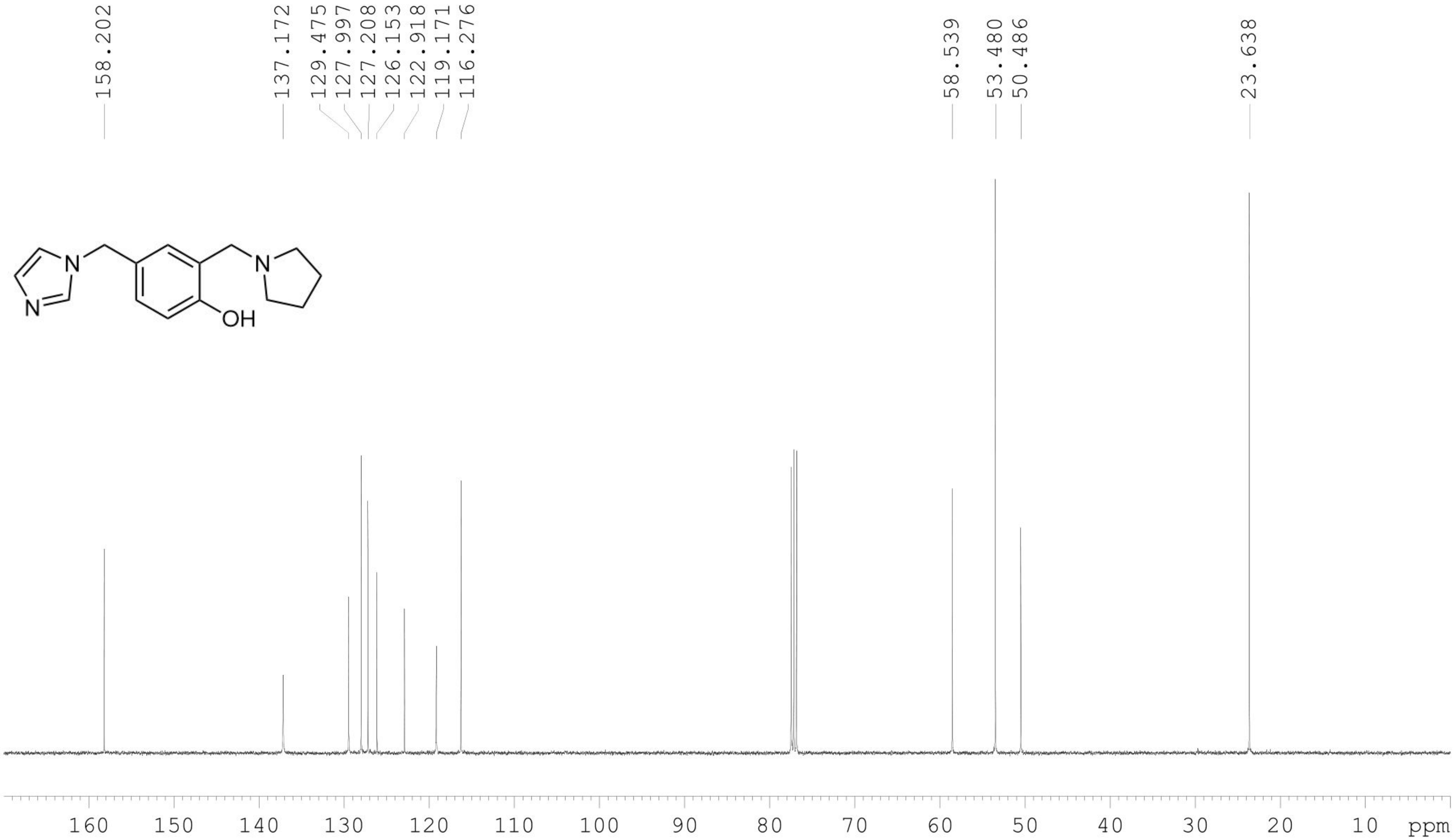

HPLC Trace (254 and 280 nm) of 1c

Waters 2.1 x100 mm C18 UPLC Column

Water/MeOH + 0.1% Triethylamine

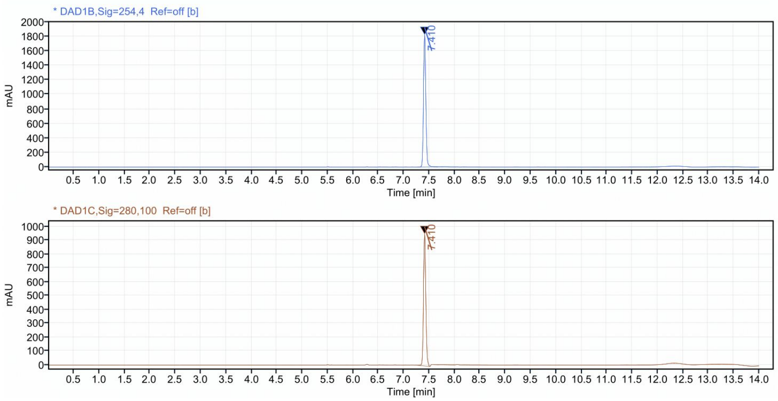

Signal: * DAD1B,Sig=254,4 Ref=off [b]

RT [min]	Peak Area	Type	Width [min]	Area	Height	Area%	Peak Area Percent
7.410	5818.016	BB	0.44	5818.02	1823.98	100.00	100.00
			Sum	5818.02			

Signal: * DAD1C,Sig=280,100 Ref=off [b]

RT [min]	Peak Area	Type	Width [min]	Area	Height	Area%	Peak Area Percent
7.410	3043.658	BB	0.28	3043.66	954.04	100.00	100.00
			Sum	3043.66			

^{1}H NMR ($CDCl_3$, 400 MHz) of 1d

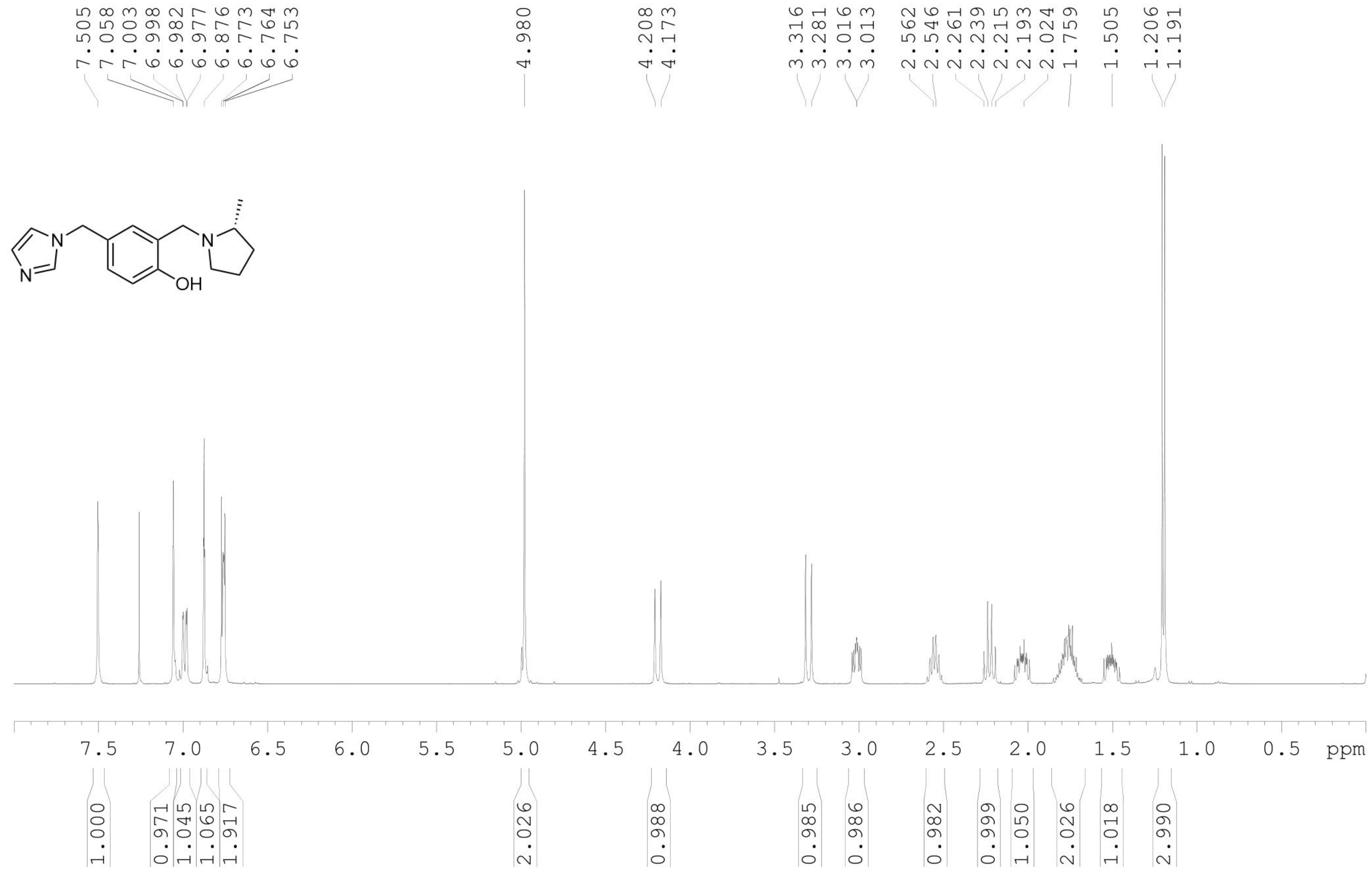

^{13}C NMR ($CDCl_3$, 100 MHz) of 1d

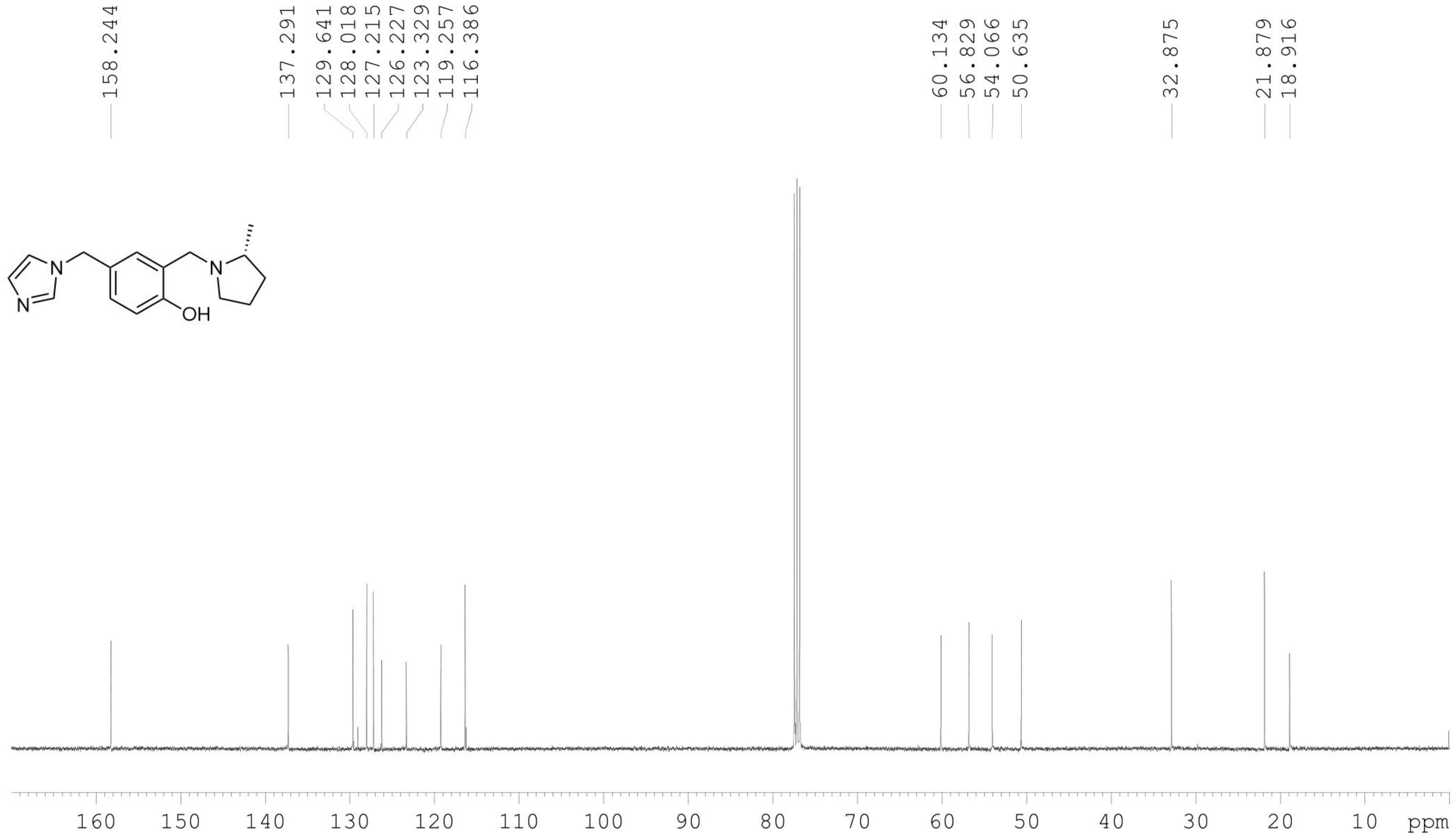

HPLC Trace (254 and 280 nm) of 1d

Waters 2.1 x100 mm C18 UPLC Column

Water/MeOH + 0.1% Triethylamine

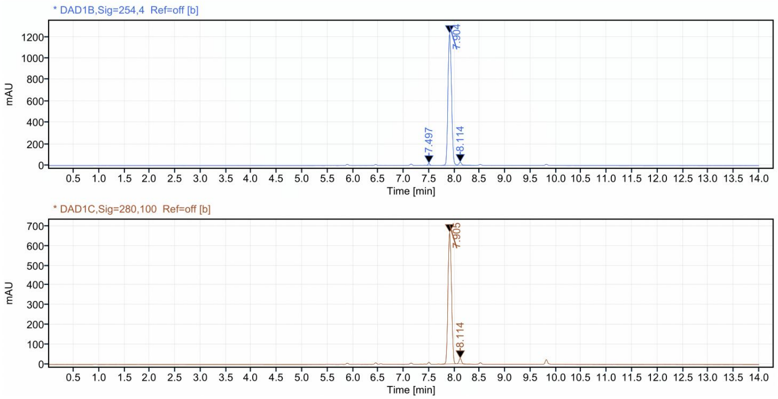

Signal: * DAD1B,Sig=254,4 Ref=off [b]

RT [min]	Peak Area	Type	Width [min]	Area	Height	Area%	Peak Area Percent
7.497	46.583	BB	0.11	46.58	18.16	0.76	0.76
7.904	5922.198	BV	0.33	5922.20	1231.27	97.11	97.11
8.114	129.668	VB	0.36	129.67	29.96	2.13	2.13
			Sum	6098.45			

Signal: * DAD1C,Sig=280,100 Ref=off [b]

RT [min]	Peak Area	Type	Width [min]	Area	Height	Area%	Peak Area Percent
7.905	3169.112	BB	0.29	3169.11	665.47	97.11	97.11
8.114	94.365	BB	0.18	94.37	27.51	2.89	2.89
			Sum	3263.48			

^{1}H NMR ($CDCl_3$, 400 MHz) of 1e

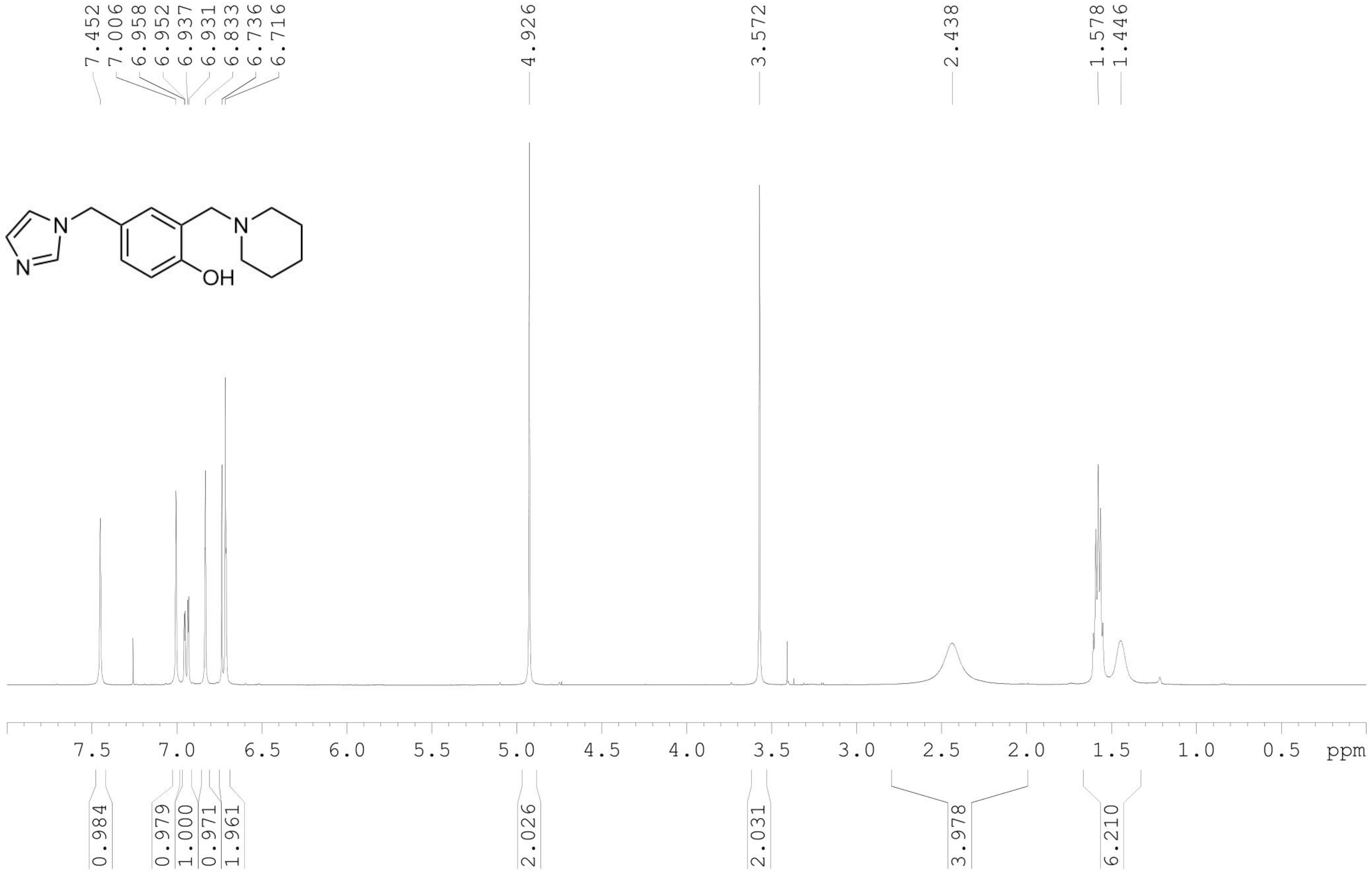

^{13}C NMR ($CDCl_3$, 100 MHz) of 1e

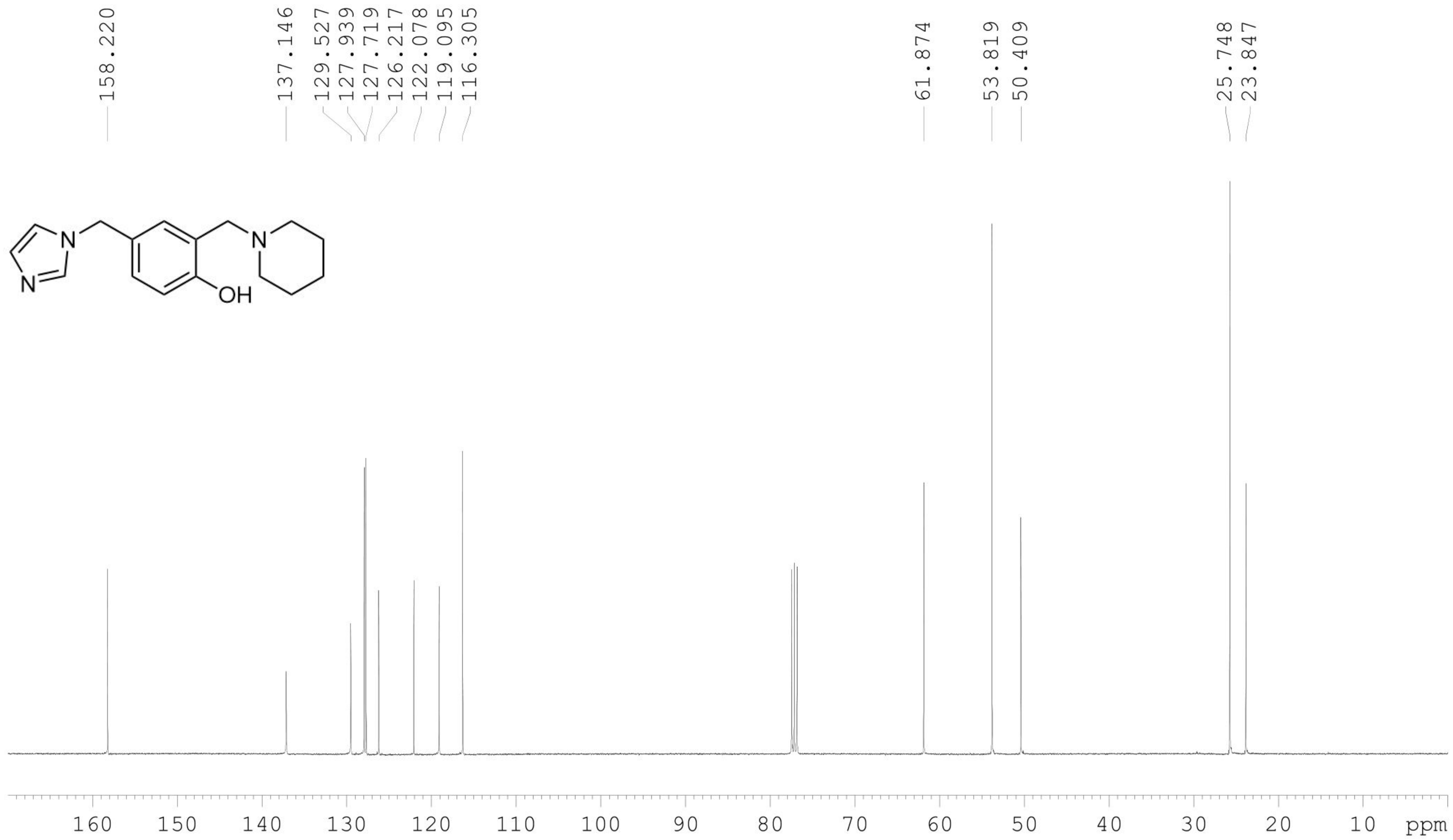

HPLC Trace (254 and 280 nm) of 1e

Waters 2.1 x100 mm C18 UPLC Column

Water/MeOH + 0.1% Triethylamine

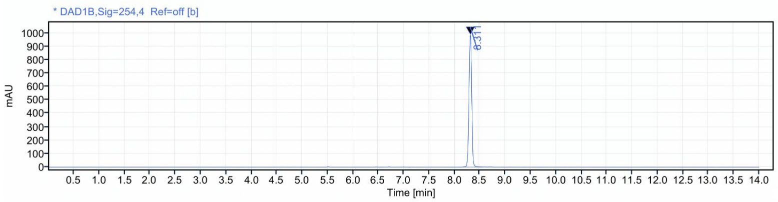

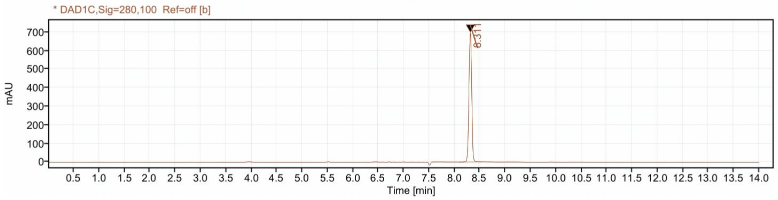

Signal: * DAD1B,Sig=254,4 Ref=off [b]

RT [min]	Peak Area	Type	Width [min]	Area	Height	Area%	Peak Area Percent
8.311	3693.186	BB	0.47	3693.19	986.77	100.00	100.00
			Sum	3693.19			

Signal: * DAD1C,Sig=280,100 Ref=off [b]

RT [min]	Peak Area	Type	Width [min]	Area	Height	Area%	Peak Area Percent
8.311	2623.694	BB	0.44	2623.69	697.48	100.00	100.00
			Sum	2623.69			

^{1}H NMR ($CDCl_3$, 400 MHz) of 2

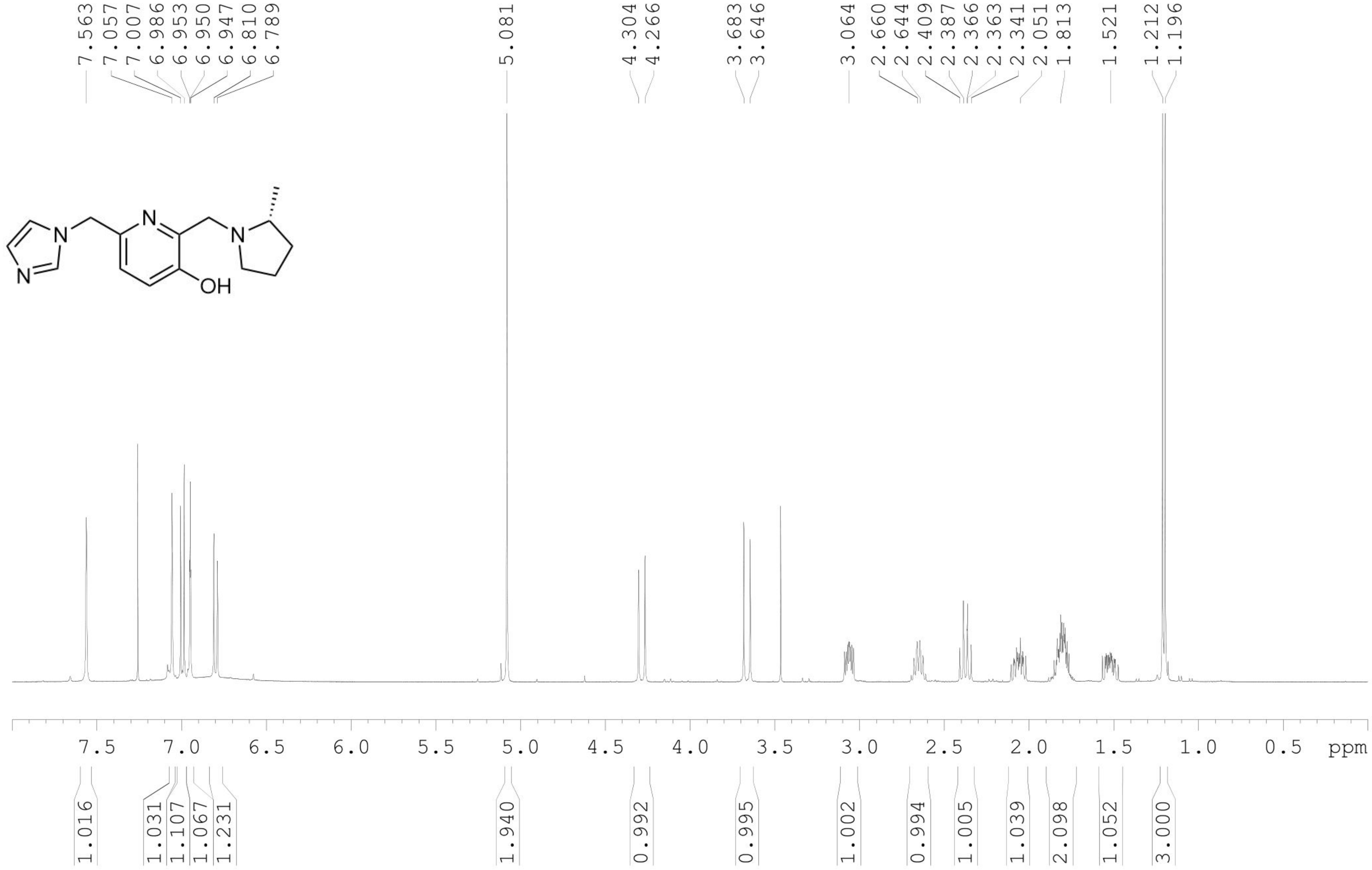

^{13}C NMR ($CDCl_3$, 100 MHz) of 2

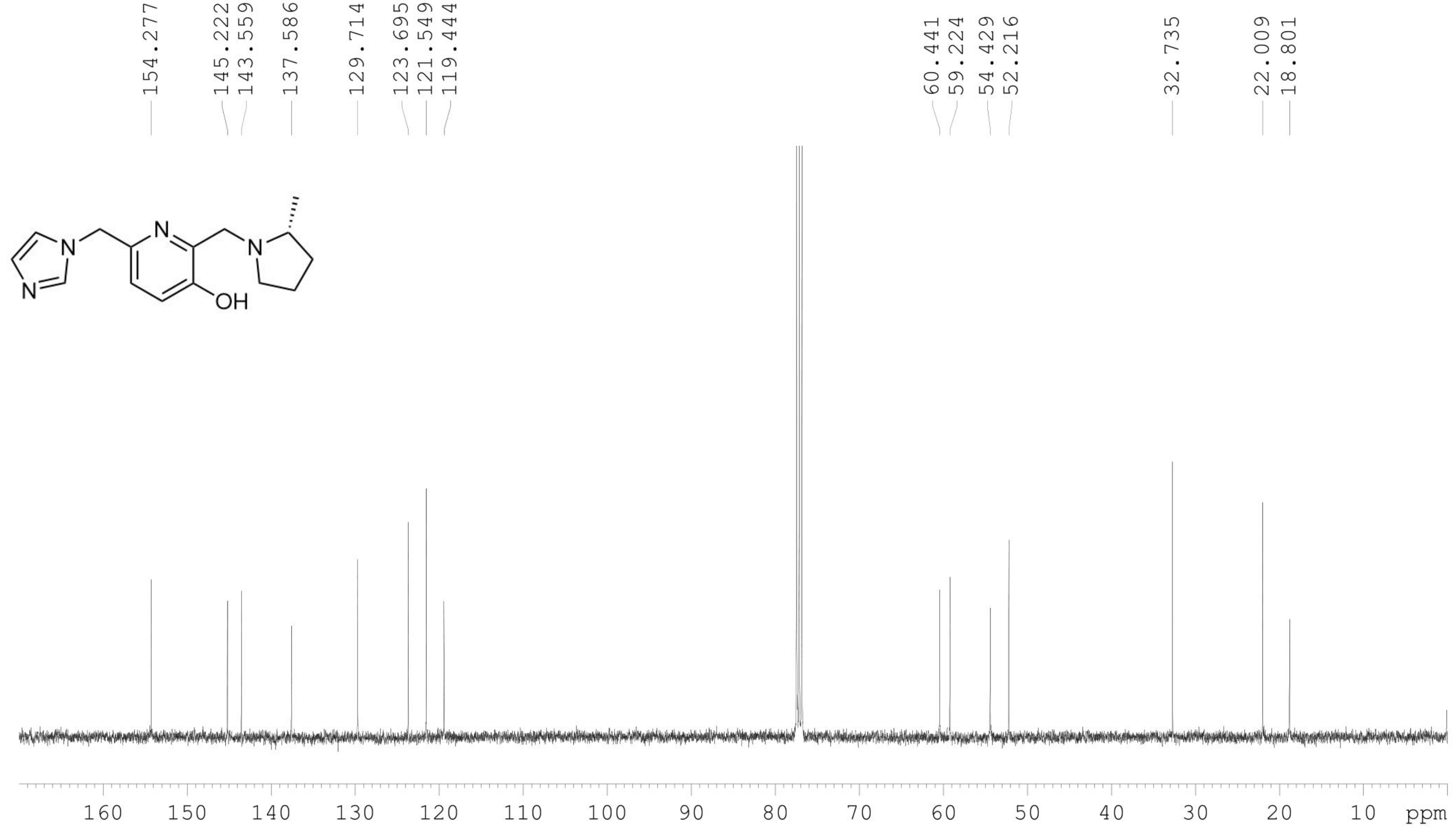

HPLC Trace (254 and 280 nm) of 2

Waters 2.1 x100 mm C18 UPLC Column

Water/MeOH + 0.1% Triethylamine

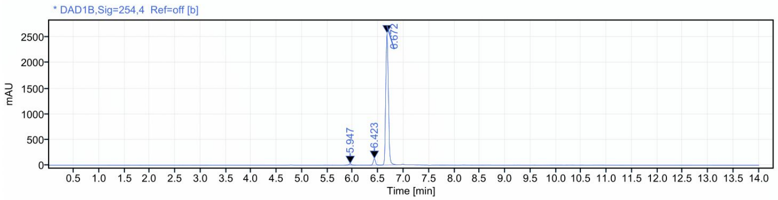

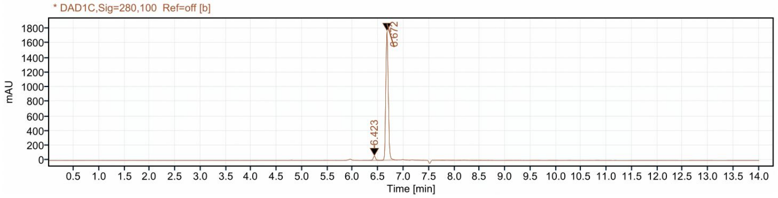

Signal: * DAD1B,Sig=254,4 Ref=off [b]

RT [min]	Peak Area	Type	Width [min]	Area	Height	Area%	Peak Area Percent
5.947	94.578	BB	0.21	94.58	25.05	0.96	0.96
6.423	396.418	BB	0.21	396.42	125.43	4.03	4.03
6.672	9356.018	BB	0.37	9356.02	2582.79	95.01	95.01
			Sum	9847.01			

Signal: * DAD1C,Sig=280,100 Ref=off [b]

RT [min]	Peak Area	Type	Width [min]	Area	Height	Area%	Peak Area Percent
6.423	186.812	BB	0.22	186.81	58.57	3.01	3.01
6.672	6018.010	BB	0.32	6018.01	1762.19	96.99	96.99
			Sum	6204.82			

^{1}H NMR ($CDCl_3$, 400 MHz) of 3

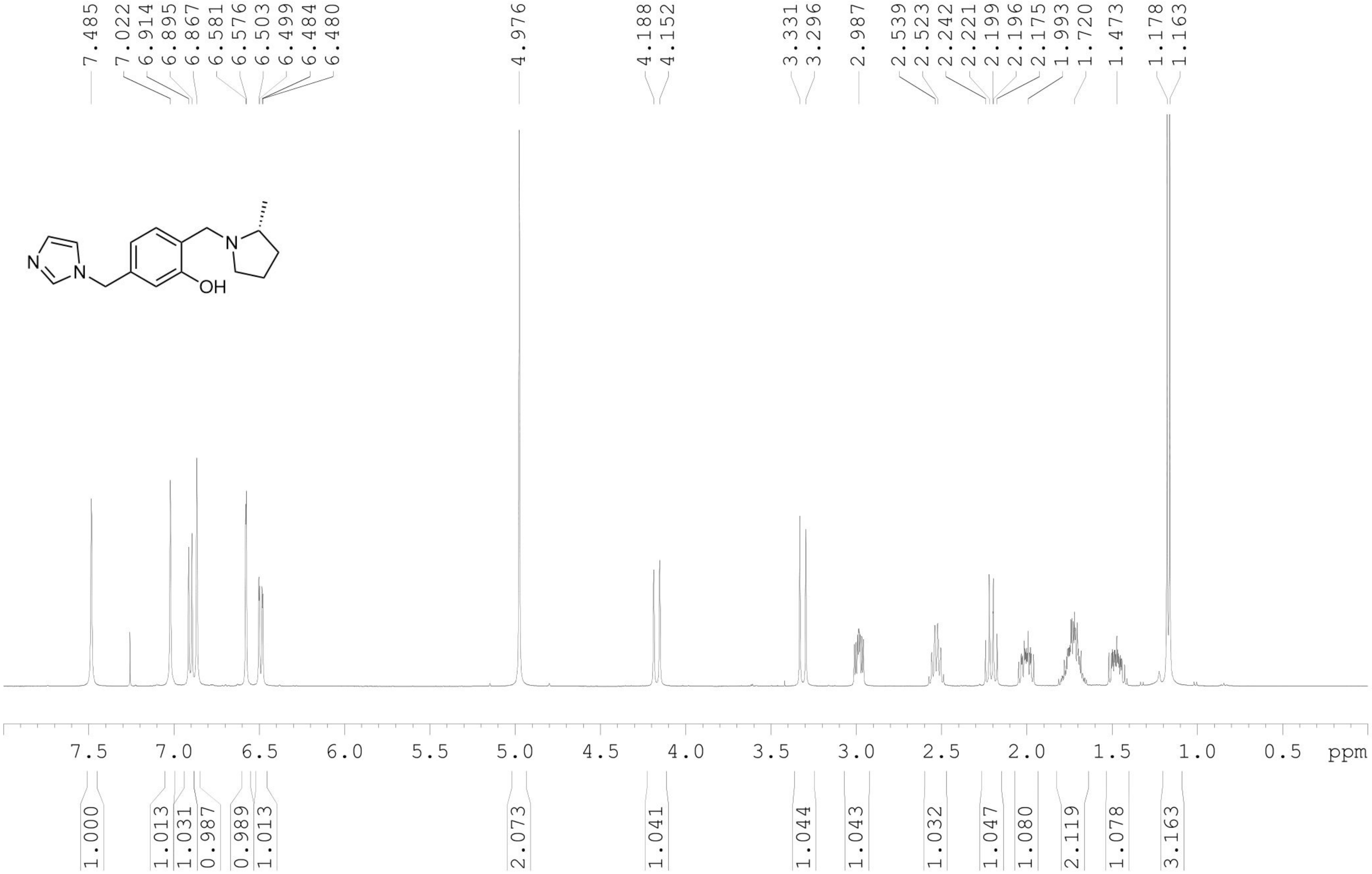

^{13}C NMR ($CDCl_3$, 100 MHz) of 3

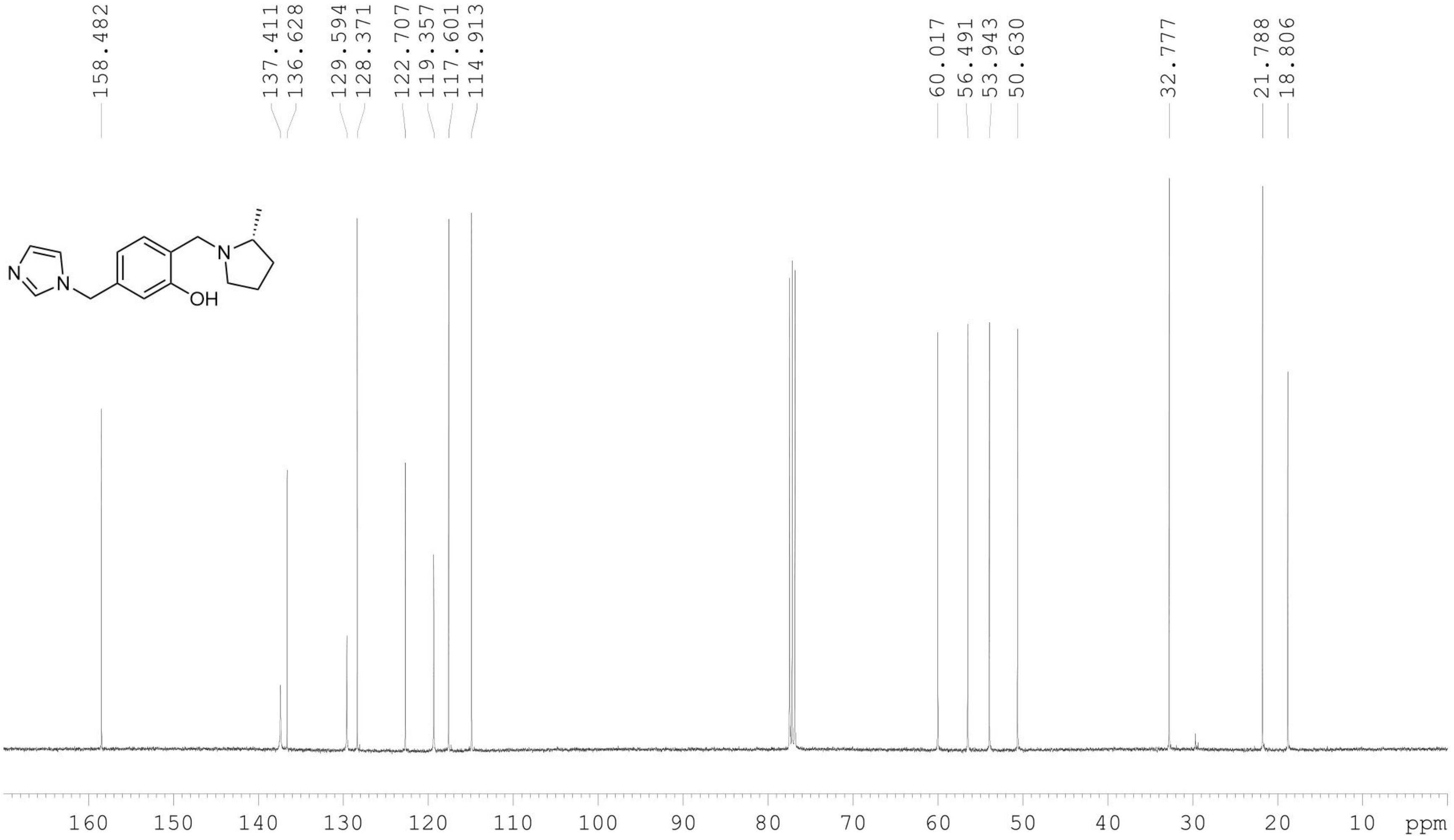

HPLC Trace (254 and 280 nm) of 3

Waters 2.1 x100 mm C18 UPLC Column

Water/MeOH + 0.1% Triethylamine

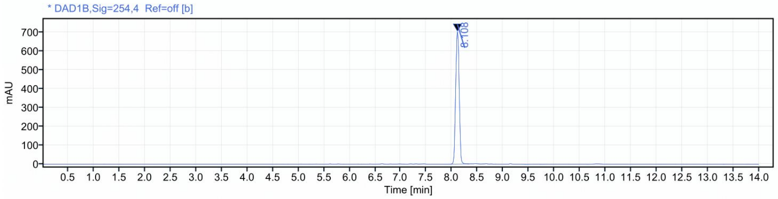

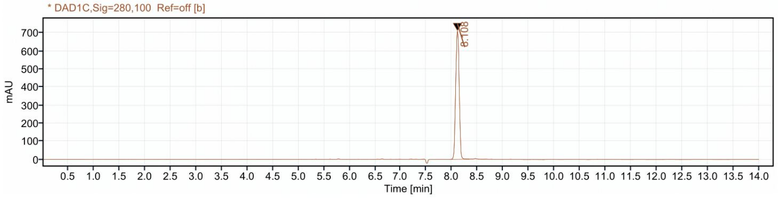

Signal: * DAD1B,Sig=254,4 Ref=off [b]

RT [min]	Peak Area	Type	Width [min]	Area	Height	Area%	Peak Area Percent
8.108	3403.953	BB	0.39	3403.95	705.47	100.00	100.00
			Sum	3403.95			

Signal: * DAD1C,Sig=280,100 Ref=off [b]

RT [min]	Peak Area	Type	Width [min]	Area	Height	Area%	Peak Area Percent
8.108	3422.125	BB	0.39	3422.12	711.30	100.00	100.00
			Sum	3422.12			

^{1}H NMR ($CDCl_3$, 400 MHz) of 4

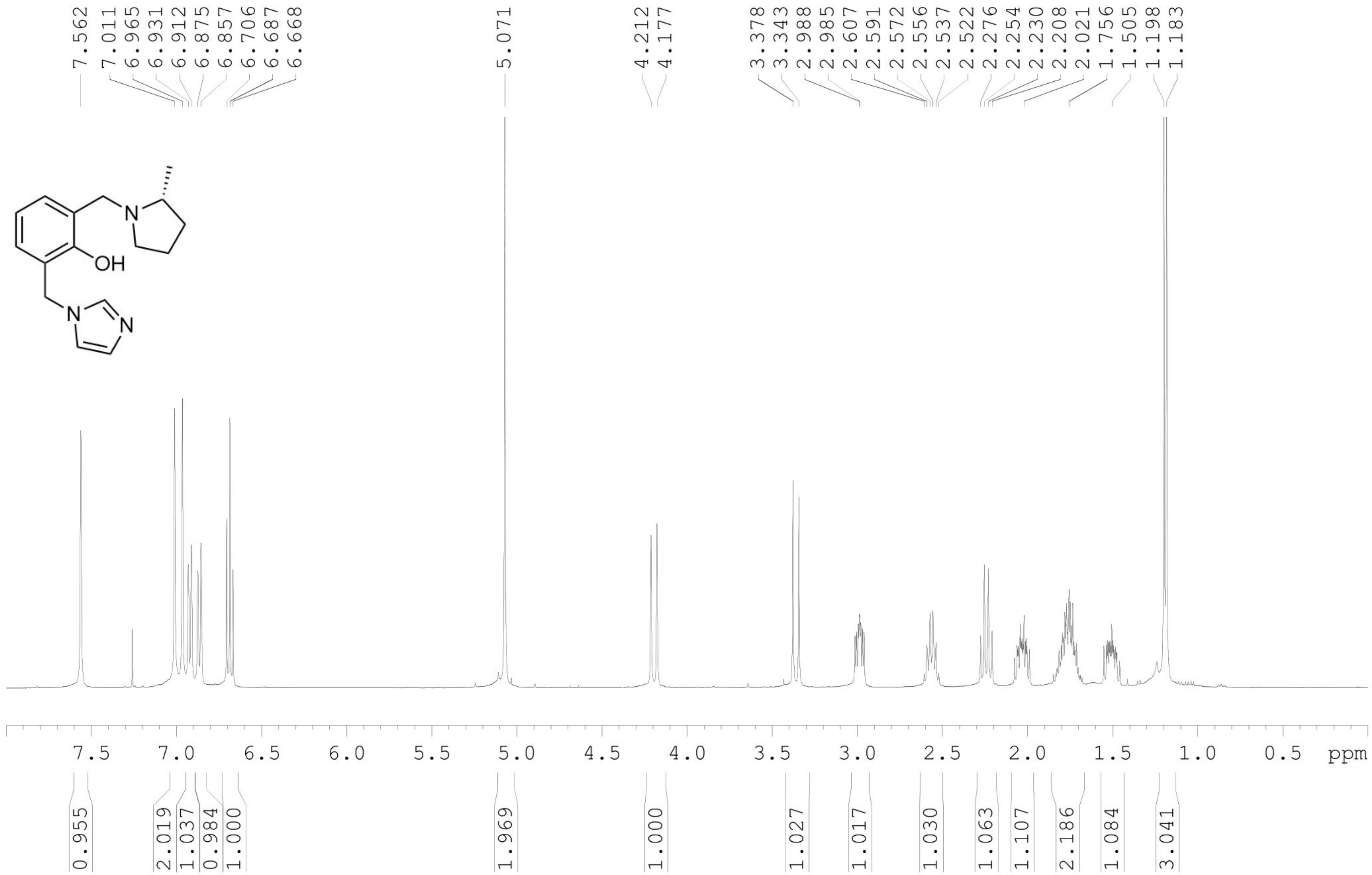

^{13}C NMR ($CDCl_3$, 100 MHz) of 4

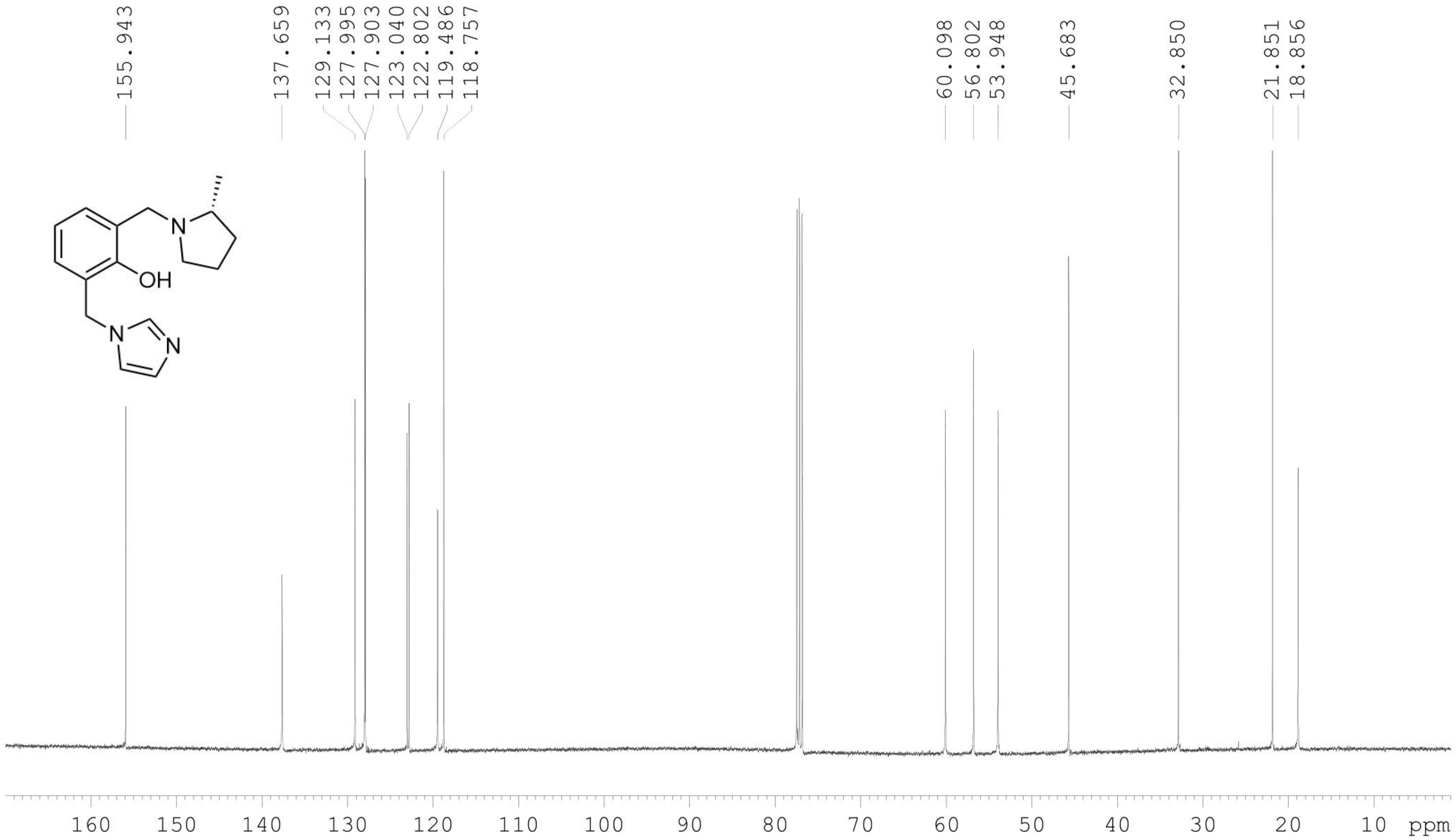

HPLC Trace (254 and 280 nm) of 4

Waters 2.1 x100 mm C18 UPLC Column

Water/MeOH + 0.1% Triethylamine

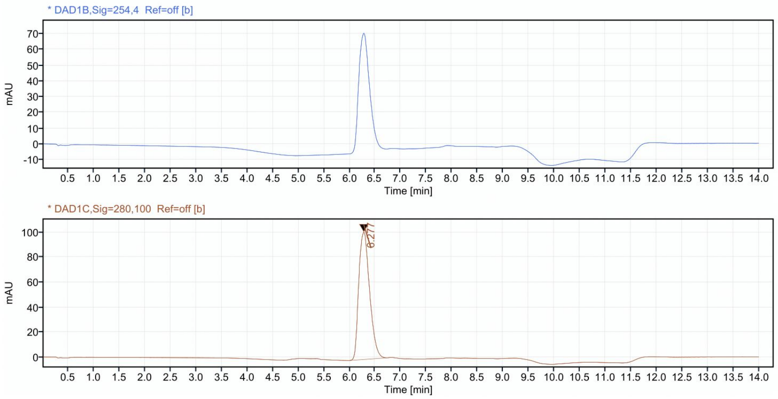

Signal: * DAD1C,Sig=280,100 Ref=off [b]

RT [min]	Peak Area	Type	Width [min]	Area	Height	Area%	Peak Area Percent
6.277	1503.170	BB	0.72	1503.17	102.05	100.00	100.00
			Sum	1503.17			

^{1}H NMR ($CDCl_3$, 400 MHz) of 5

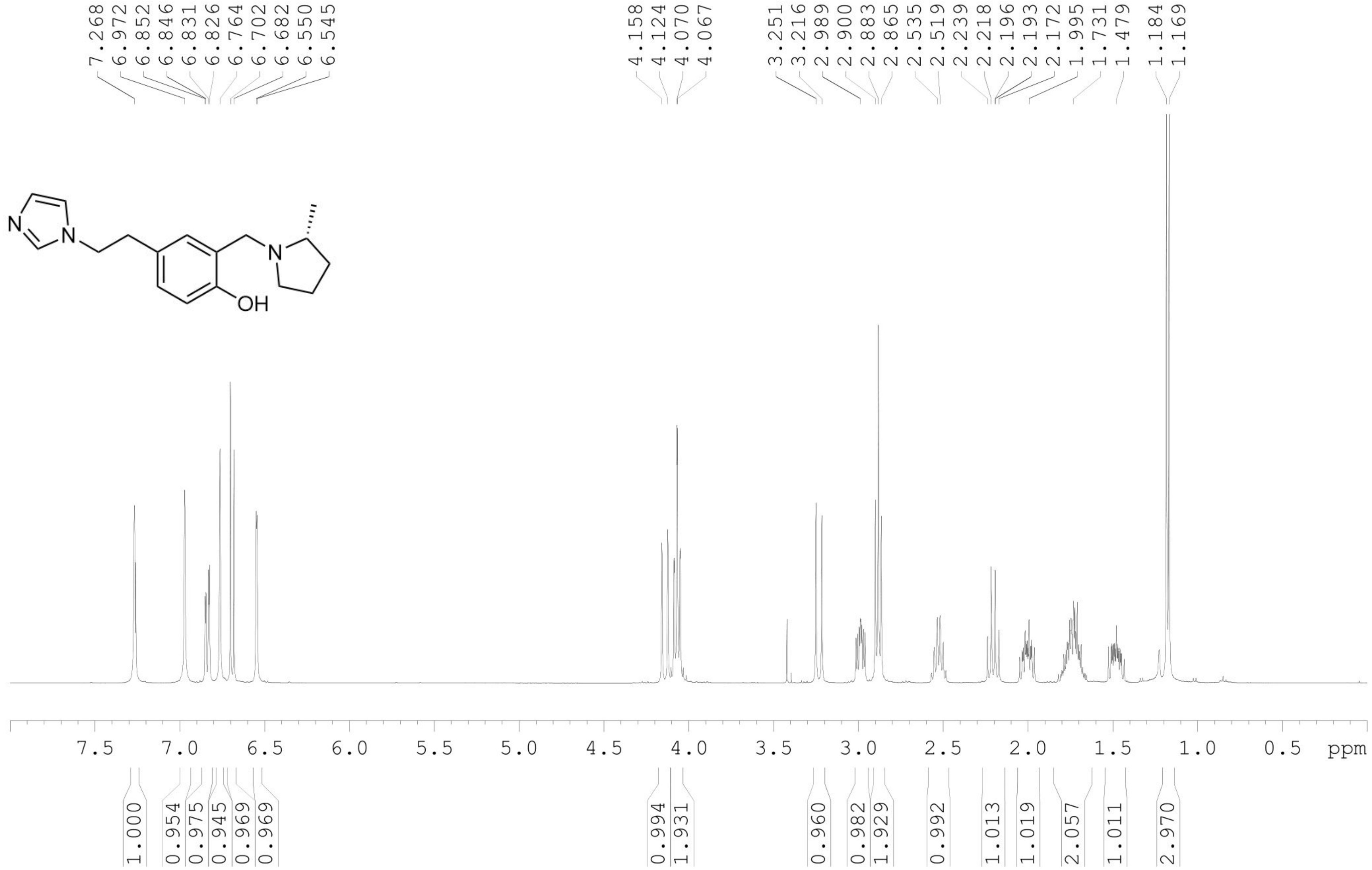

^{13}C NMR ($CDCl_3$, 100 MHz) of 5

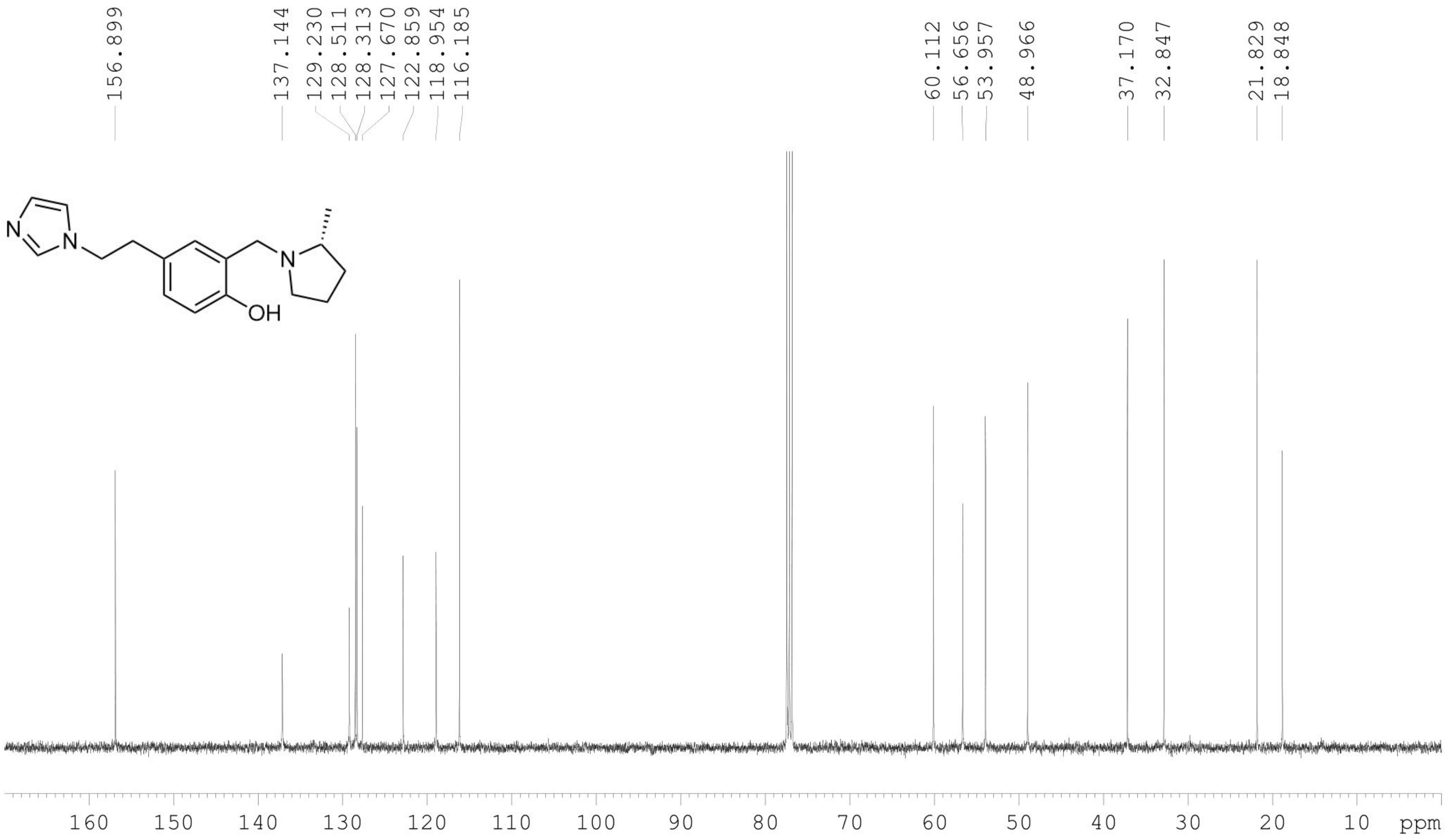

HPLC Trace (254 and 280 nm) of 5

Waters 2.1 x100 mm C18 UPLC Column

Water/MeOH + 0.1% Triethylamine

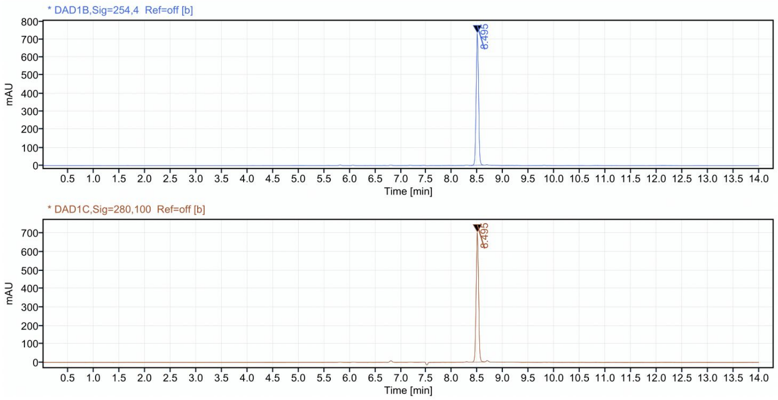

Signal: * DAD1B,Sig=254,4 Ref=off [b]

RT [min]	Peak Area	Type	Width [min]	Area	Height	Area%	Peak Area Percent
8.495	2406.844	BB	0.26	2406.84	732.72	100.00	100.00
			Sum	2406.84			

Signal: * DAD1C,Sig=280,100 Ref=off [b]

RT [min]	Peak Area	Type	Width [min]	Area	Height	Area%	Peak Area Percent
8.495	2341.955	BB	0.25	2341.95	705.31	100.00	100.00
			Sum	2341.95			

^{1}H NMR ($CDCl_3$, 400 MHz) of 6

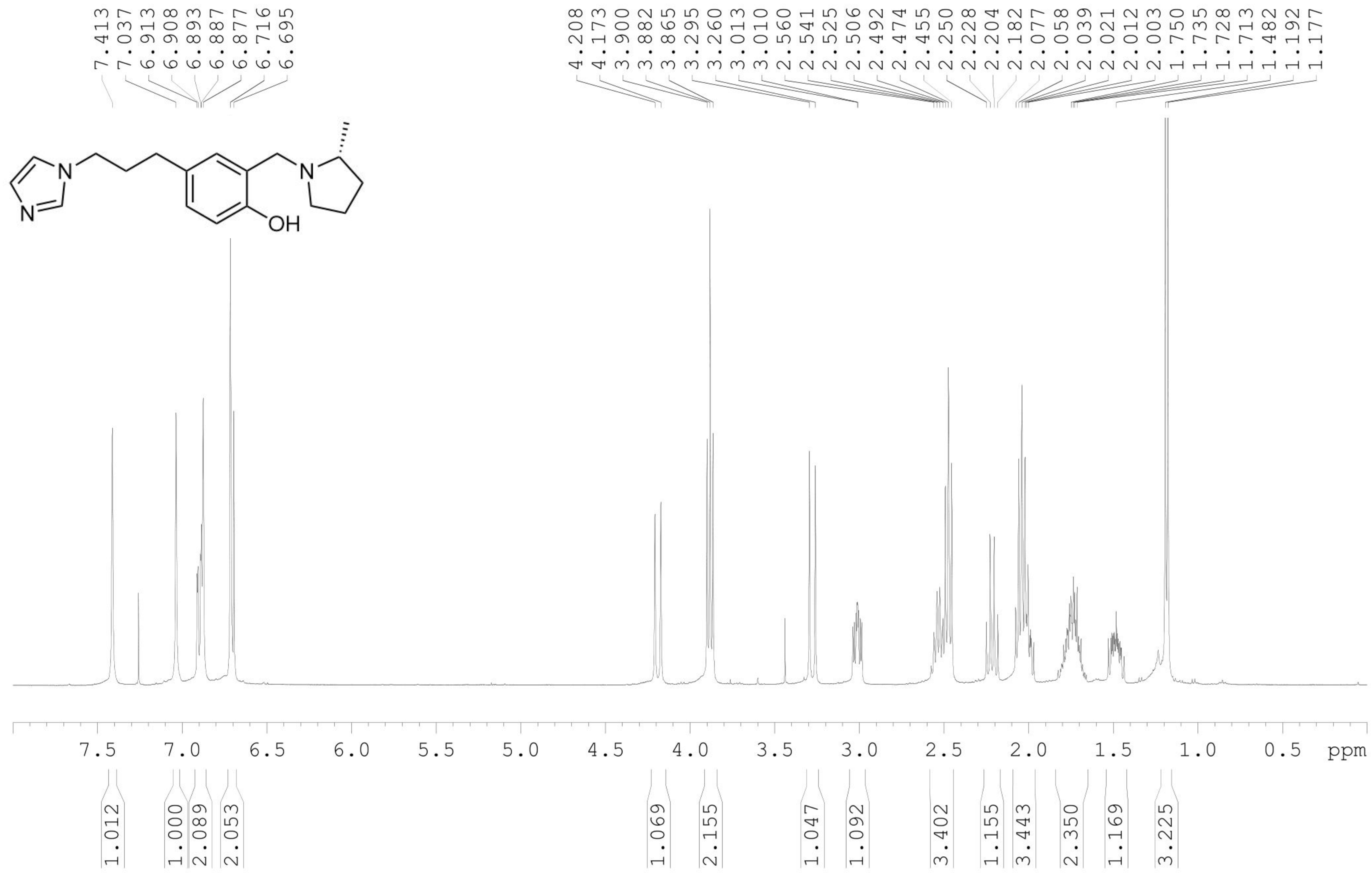

^{13}C NMR ($CDCl_3$, 100 MHz) of 6

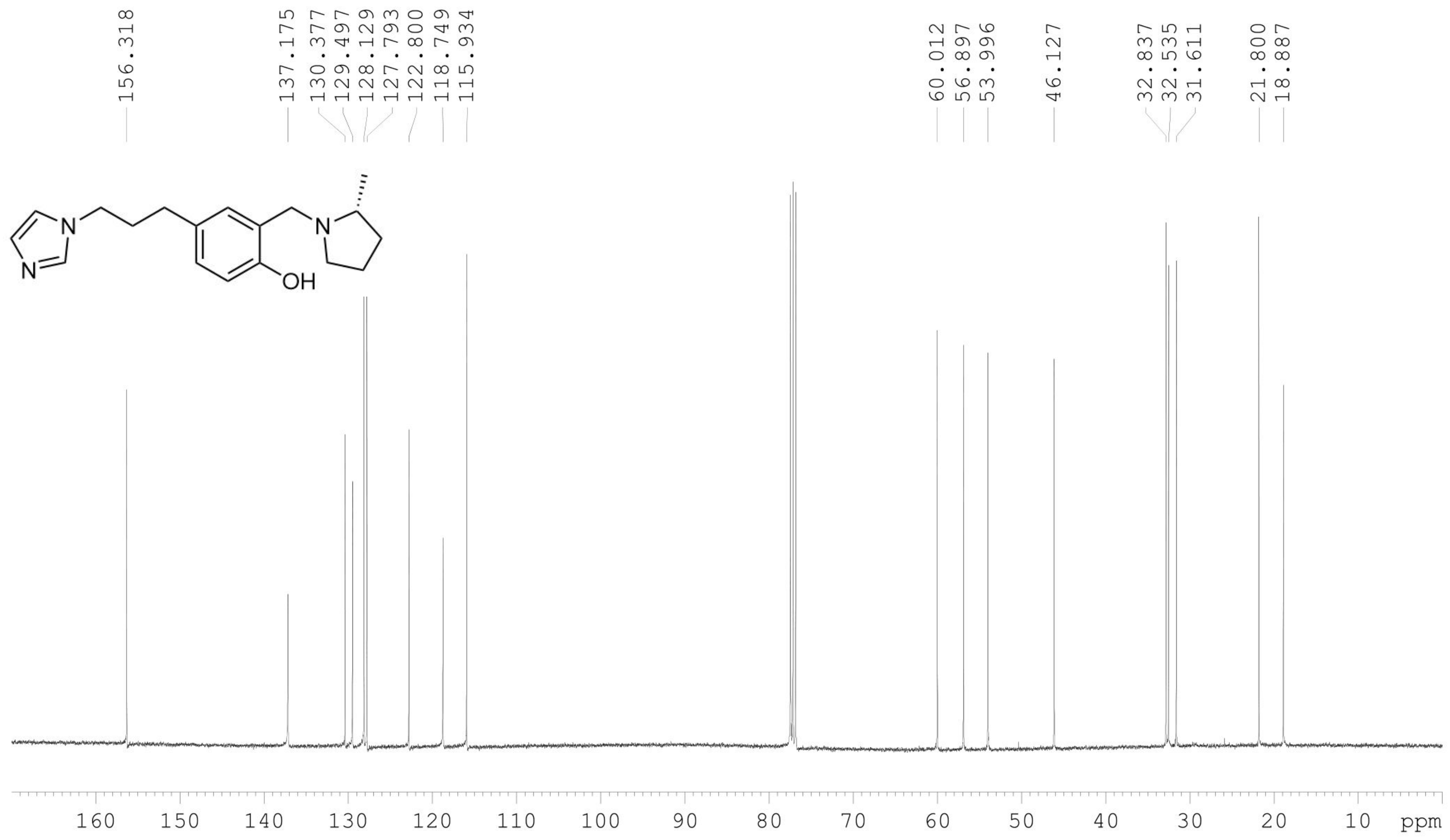

HPLC Trace (254 and 280 nm) of 6

Waters 2.1 x100 mm C18 UPLC Column

Water/MeOH + 0.1% Triethylamine

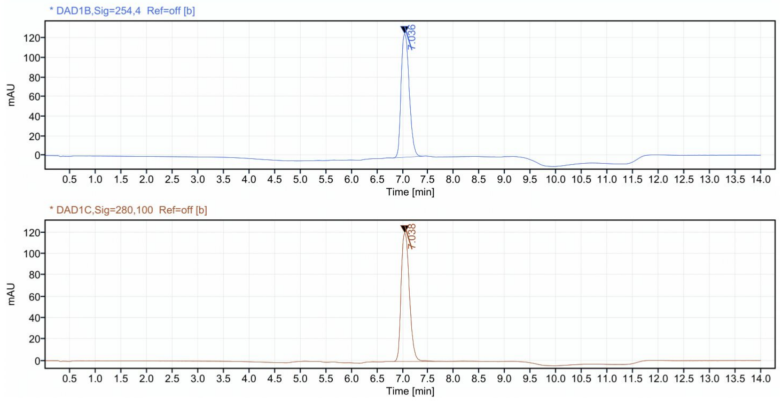

Signal: * DAD1B,Sig=254,4 Ref=off [b]

RT [min]	Peak Area	Type	Width [min]	Area	Height	Area%	Peak Area Percent
7.036	1390.497	BB	0.56	1390.50	125.74	100.00	100.00
			Sum	1390.50			

Signal: * DAD1C,Sig=280,100 Ref=off [b]

RT [min]	Peak Area	Type	Width [min]	Area	Height	Area%	Peak Area Percent
7.038	1350.644	BB	0.83	1350.64	119.66	100.00	100.00
			Sum	1350.64			

^{1}H NMR ($CDCl_3$, 400 MHz) of 7

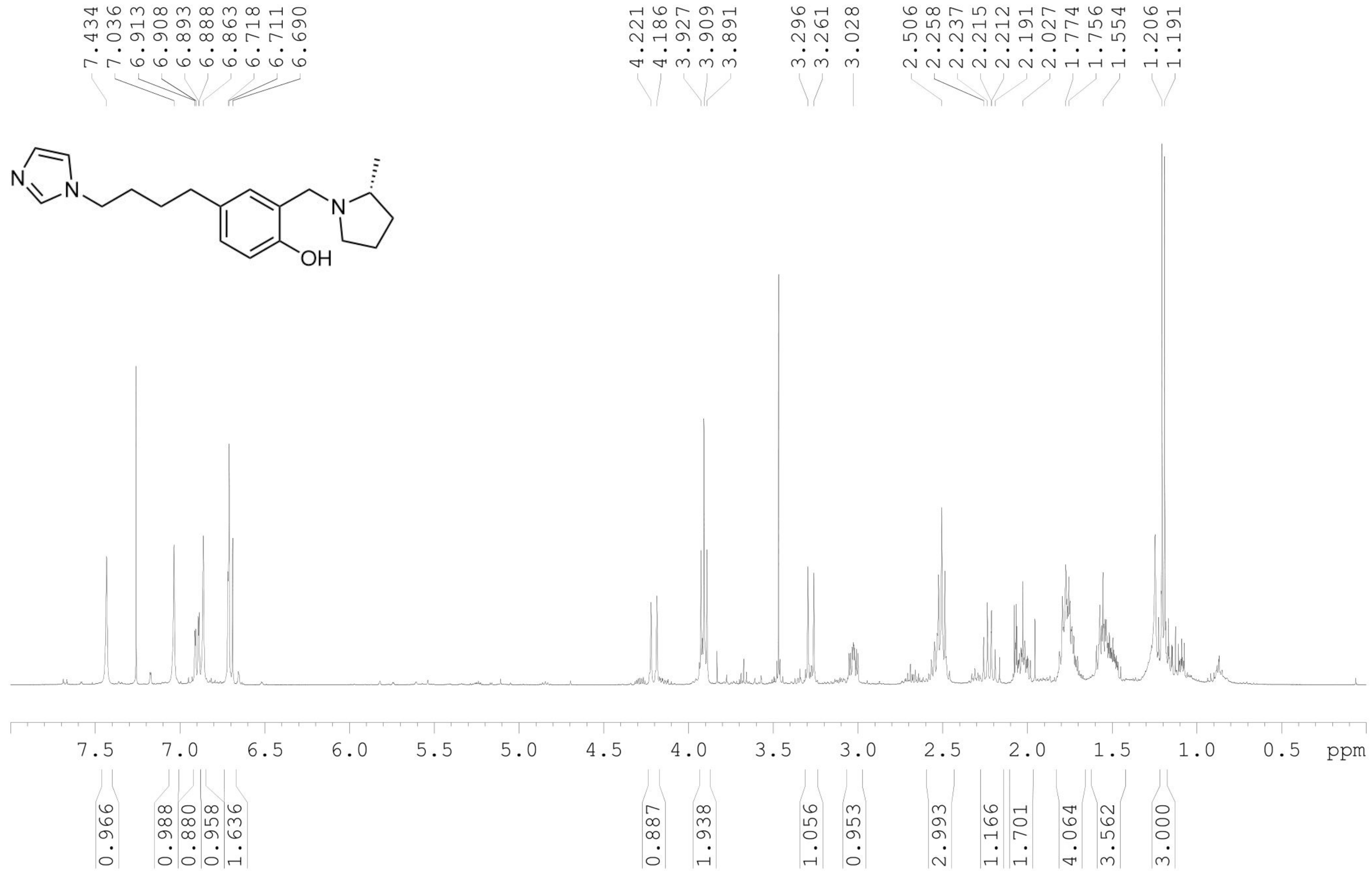

^{13}C NMR ($CDCl_3$, 100 MHz) of 7

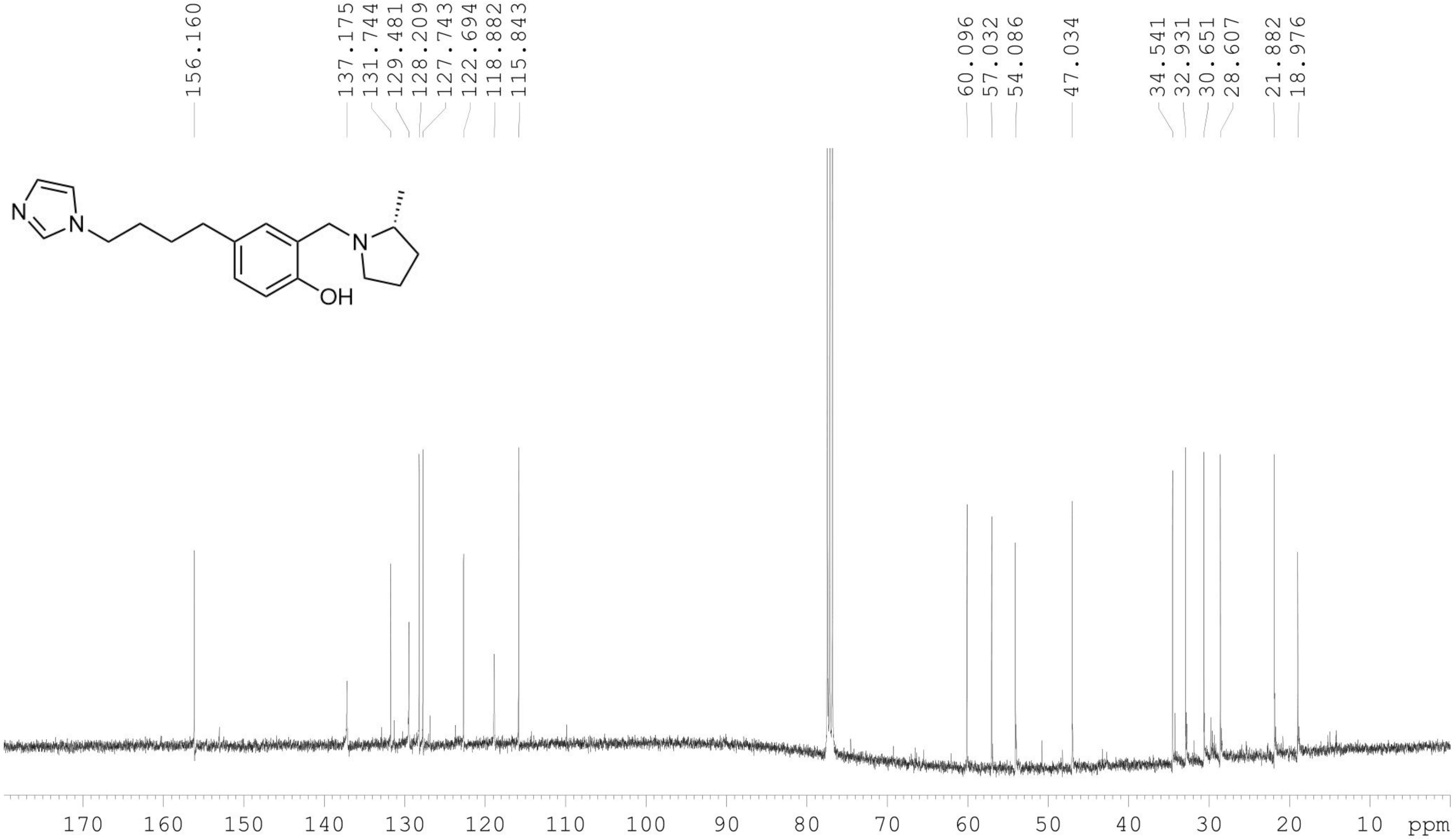

HPLC Trace (254 and 280 nm) of 7

Waters 2.1 x100 mm C18 UPLC Column

Water/MeOH + 0.1% Triethylamine

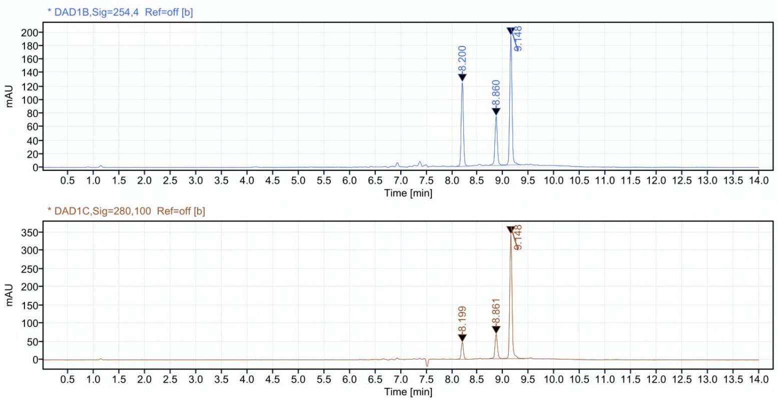

Signal: * DAD1B,Sig=254,4 Ref=off [b]

RT [min]	Peak Area	Type	Width [min]	Area	Height	Area%	Peak Area Percent
8.200	374.991	BB	0.27	374.99	124.97	31.08	31.08
8.860	236.571	BB	0.31	236.57	72.97	19.61	19.61
9.148	594.911	BB	0.37	594.91	192.07	49.31	49.31
			Sum	1206.47			

Signal: * DAD1C,Sig=280,100 Ref=off [b]

RT [min]	Peak Area	Type	Width [min]	Area	Height	Area%	Peak Area Percent
8.199	140.737	BB	0.27	140.74	46.54	9.77	9.77
8.861	227.604	BB	0.26	227.60	67.61	15.80	15.80
9.148	1072.608	BB	0.40	1072.61	340.90	74.44	74.44
			Sum	1440.95			

^{1}H NMR ($CDCl_3$, 400 MHz) of 8

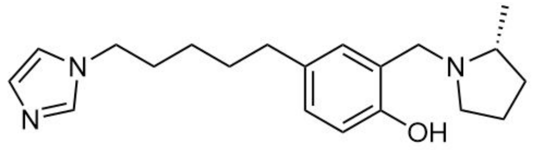

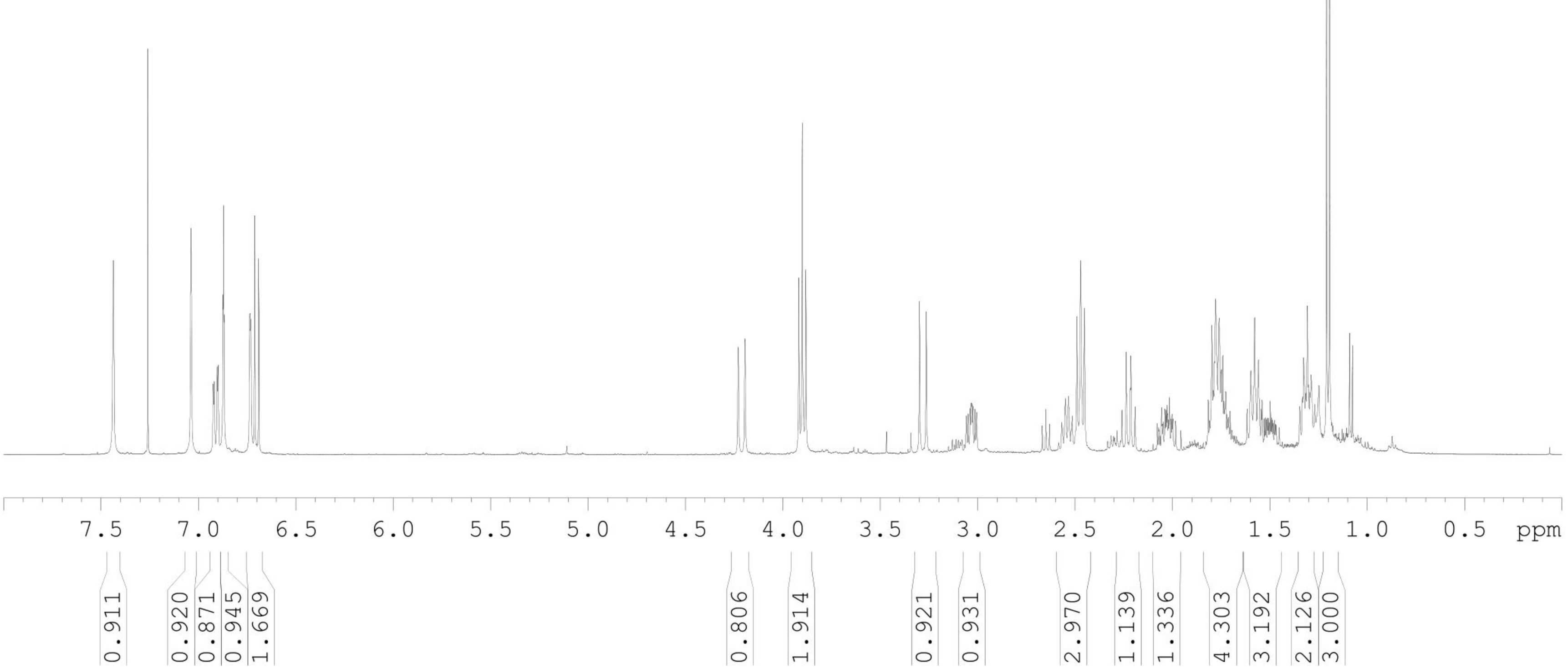

^{13}C NMR ($CDCl_3$, 100 MHz) of 8

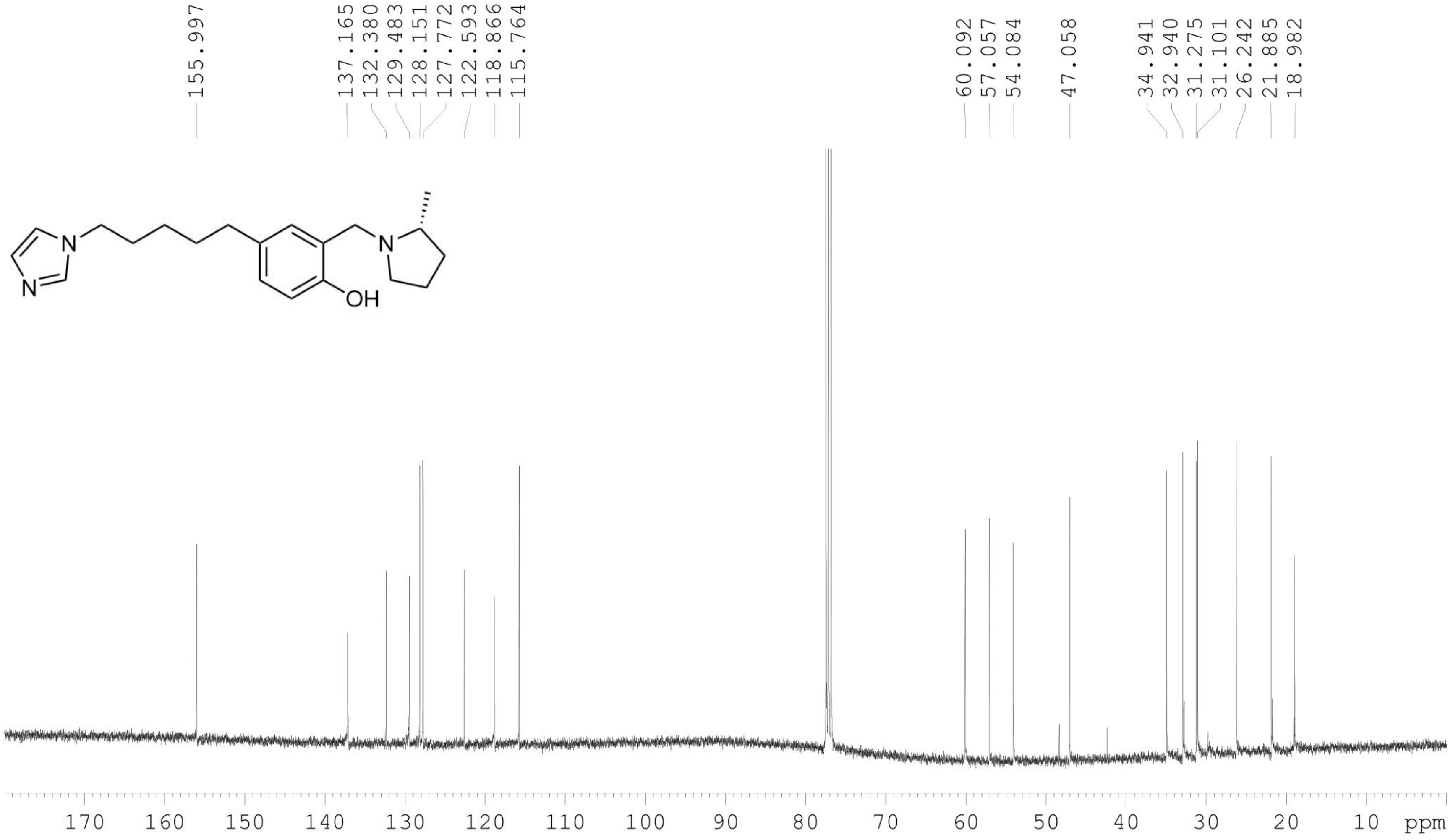

HPLC Trace (254 and 280 nm) of 8

Waters 2.1 x100 mm C18 UPLC Column

Water/MeOH + 0.1% Triethylamine

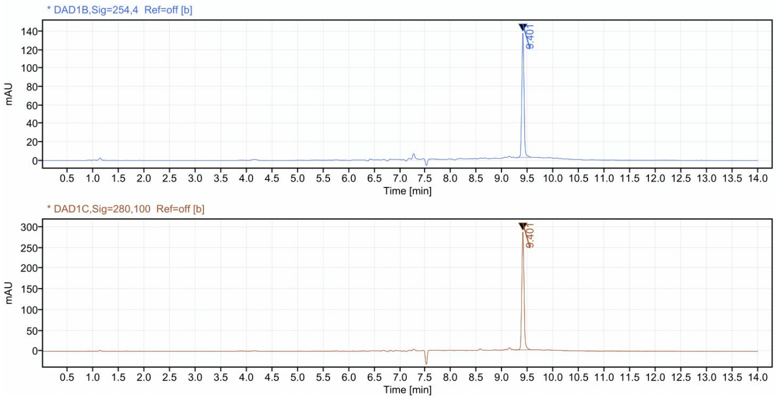

Signal: * DAD1B,Sig=254,4 Ref=off [b]

RT [min]	Peak Area	Type	Width [min]	Area	Height	Area%	Peak Area Percent
9.401	411.782	BB	0.27	411.78	136.89	100.00	100.00
			Sum	411.78			

Signal: * DAD1C,Sig=280,100 Ref=off [b]

RT [min]	Peak Area	Type	Width [min]	Area	Height	Area%	Peak Area Percent
9.401	877.013	BB	0.31	877.01	287.80	100.00	100.00
			Sum	877.01			

^{1}H NMR ($CDCl_3$, 400 MHz) of 9

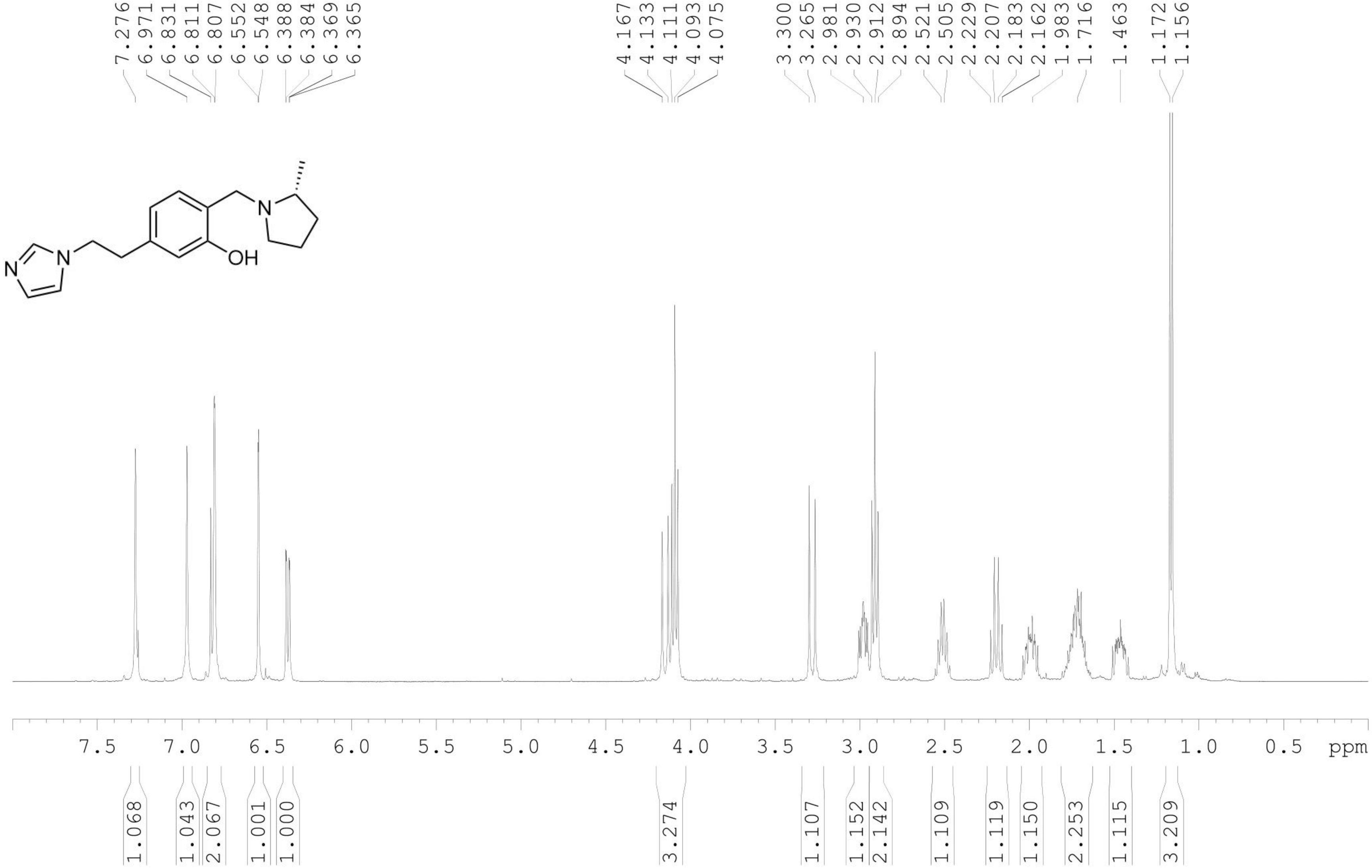

^{13}C NMR ($CDCl_3$, 100 MHz) of 9

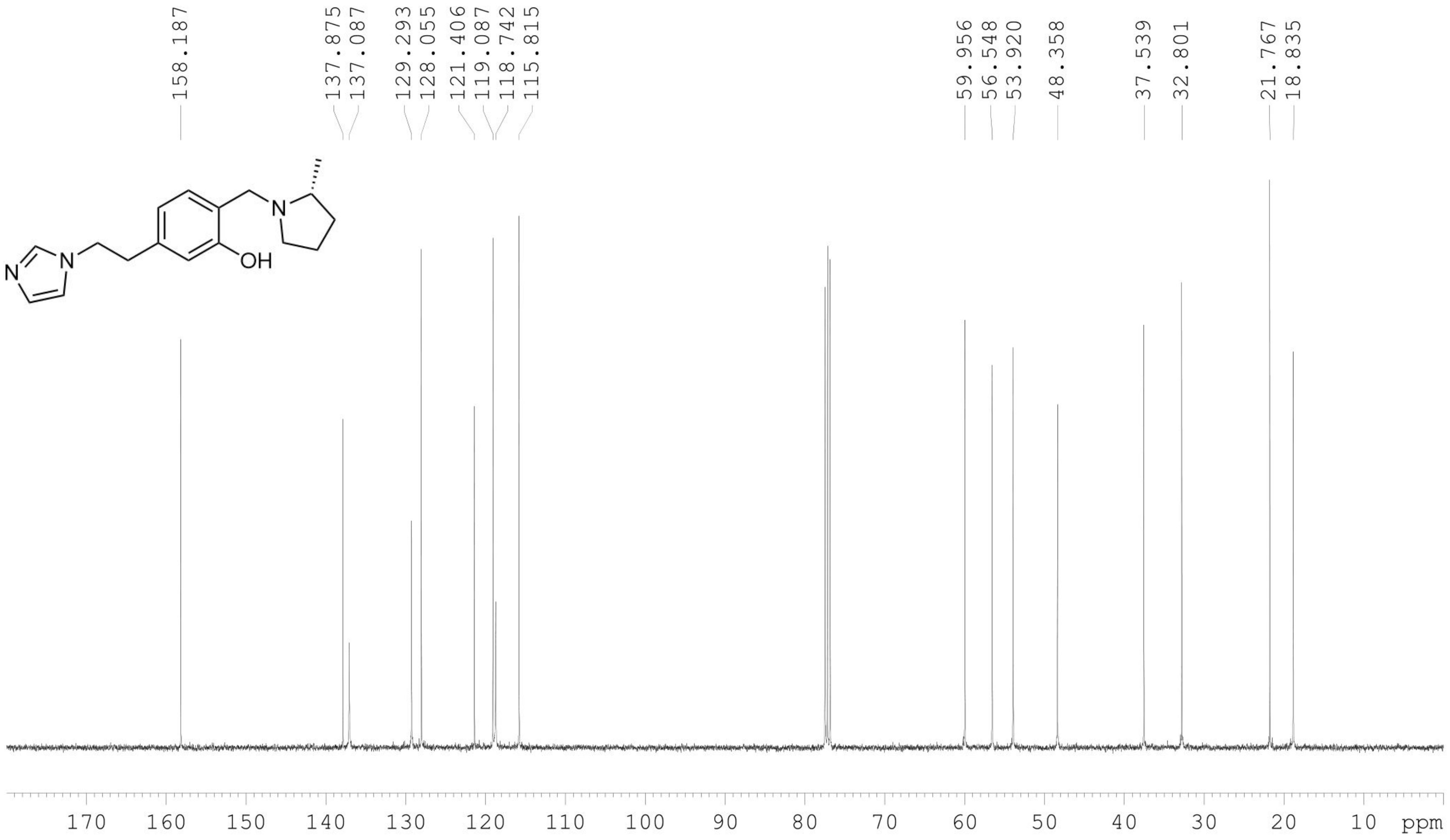

HPLC Trace (254 and 280 nm) of 9

Waters 2.1 x100 mm C18 UPLC Column

Water/MeOH + 0.1% Triethylamine

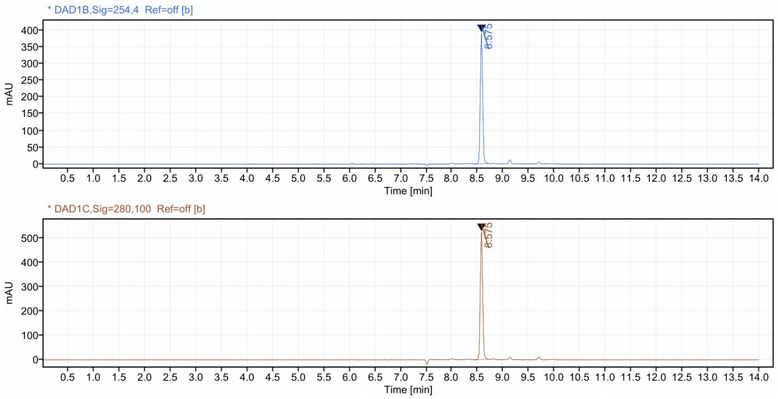

Signal: * DAD1B,Sig=254,4 Ref=off [b]

RT [min]	Peak Area	Type	Width [min]	Area	Height	Area%	Peak Area Percent
8.575	1281.410	BB	0.32	1281.41	391.43	100.00	100.00
			Sum	1281.41			

Signal: * DAD1C,Sig=280,100 Ref=off [b]

RT [min]	Peak Area	Type	Width [min]	Area	Height	Area%	Peak Area Percent
8.575	1735.581	BB	0.31	1735.58	528.69	100.00	100.00
			Sum	1735.58			

^{1}H NMR ($CDCl_3$, 400 MHz) of 10

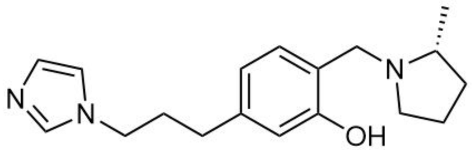

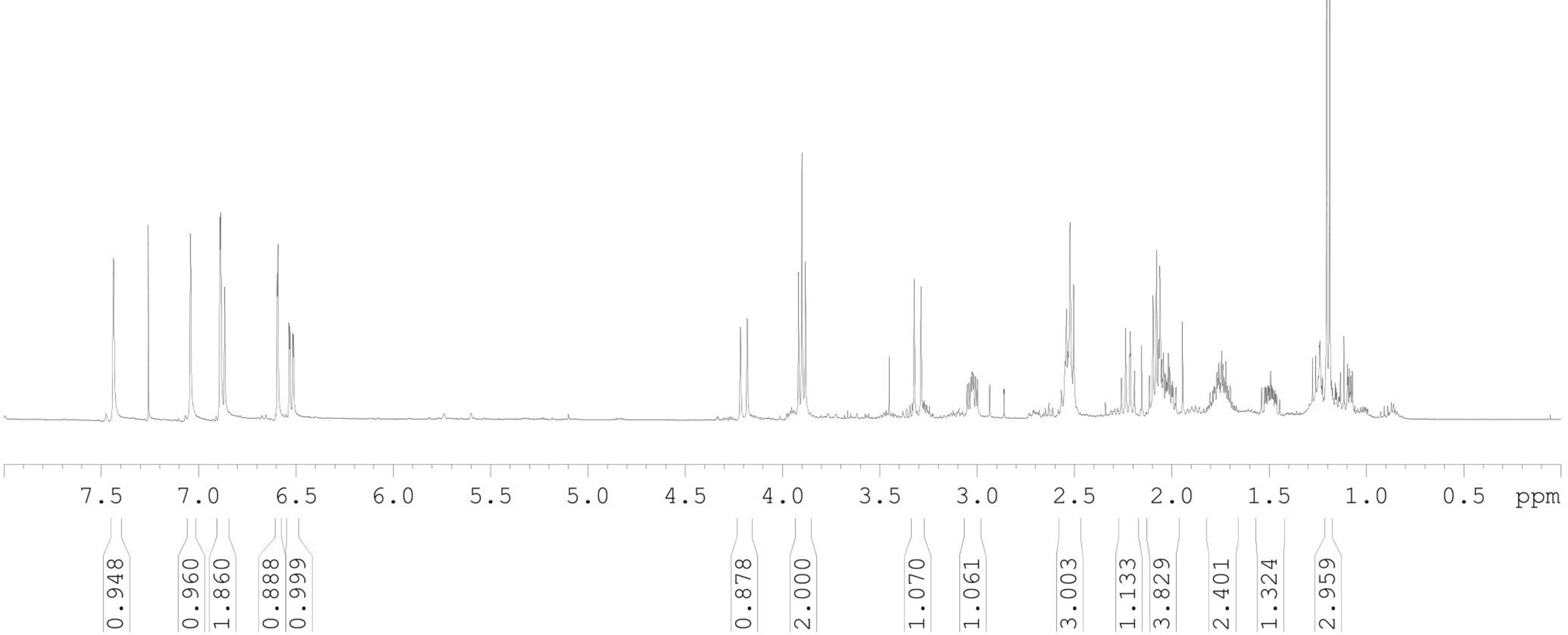

^{13}C NMR ($CDCl_3$, 100 MHz) of 10

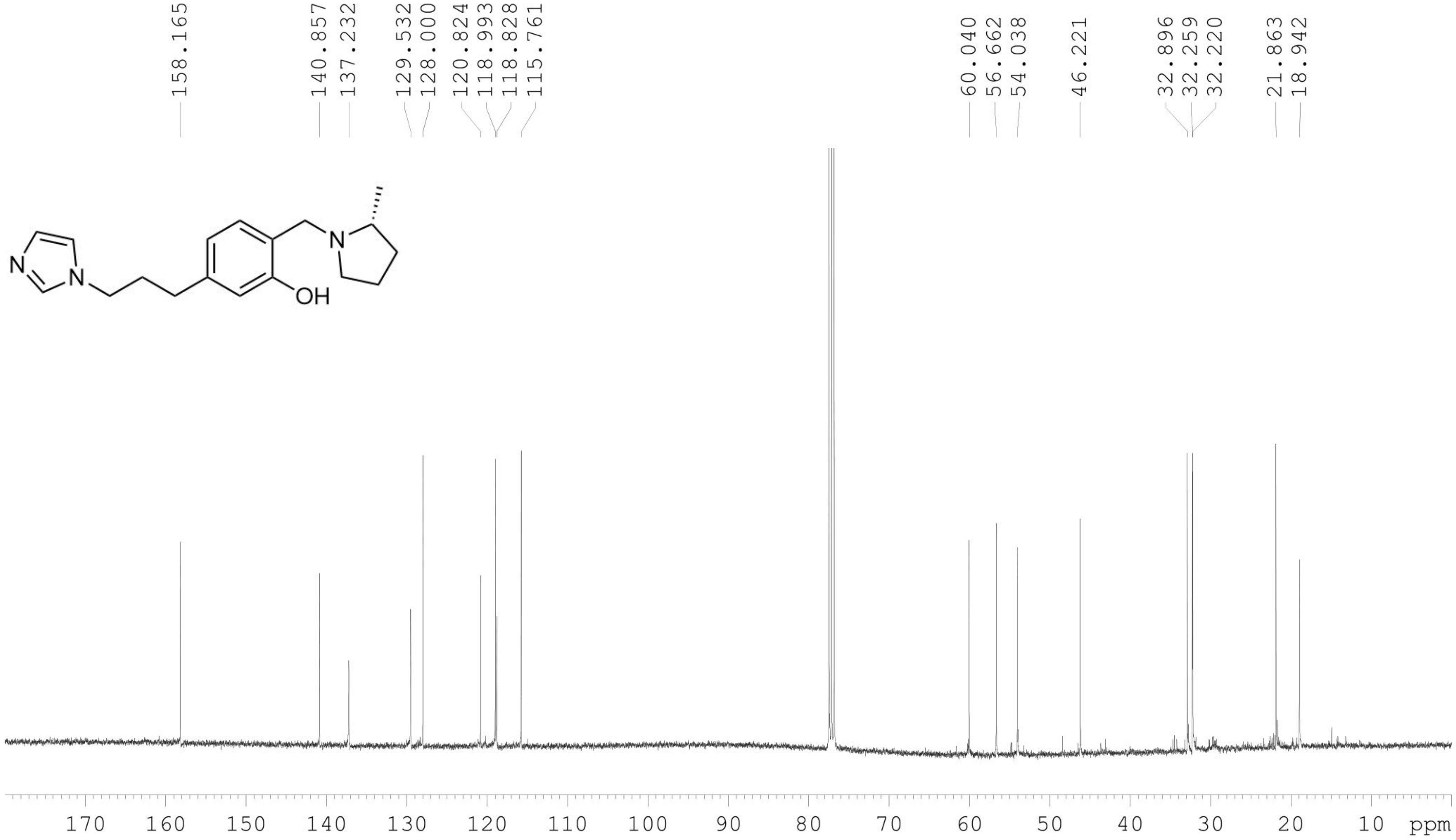

HPLC Trace (254 and 280 nm) of 10

Waters 2.1 x100 mm C18 UPLC Column

Water/MeOH + 0.1% Triethylamine

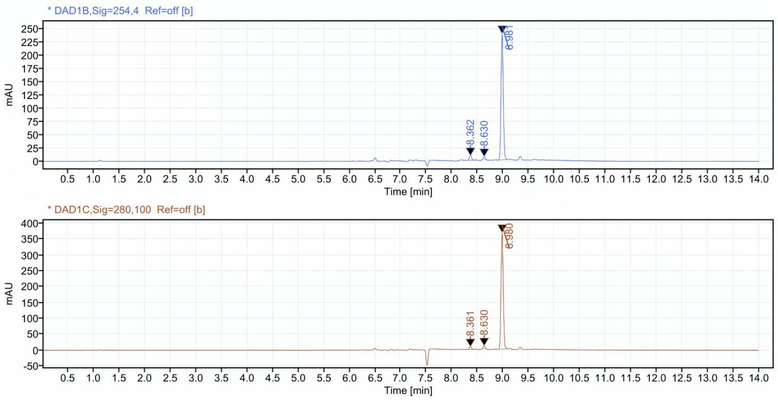

Signal: * DAD1B,Sig=254,4 Ref=off [b]

RT [min]	Peak Area	Type	Width [min]	Area	Height	Area%	Peak Area Percent
8.362	23.267	MM m	0.17	23.27	8.17	3.02	3.02
8.630	13.354	MM m	0.15	13.35	5.15	1.74	1.74
8.981	732.650	BB	0.19	732.65	238.99	95.24	95.24
			Sum	769.27			

Signal: * DAD1C,Sig=280,100 Ref=off [b]

RT [min]	Peak Area	Type	Width [min]	Area	Height	Area%	Peak Area Percent
8.361	24.874	MM m	0.14	24.87	7.96	2.12	2.12
8.630	21.793	MM m	0.08	21.79	8.06	1.85	1.85
8.980	1129.021	BB	0.20	1129.02	367.52	96.03	96.03
			Sum	1175.69			

^{1}H NMR ($CDCl_3$, 400 MHz) of 11

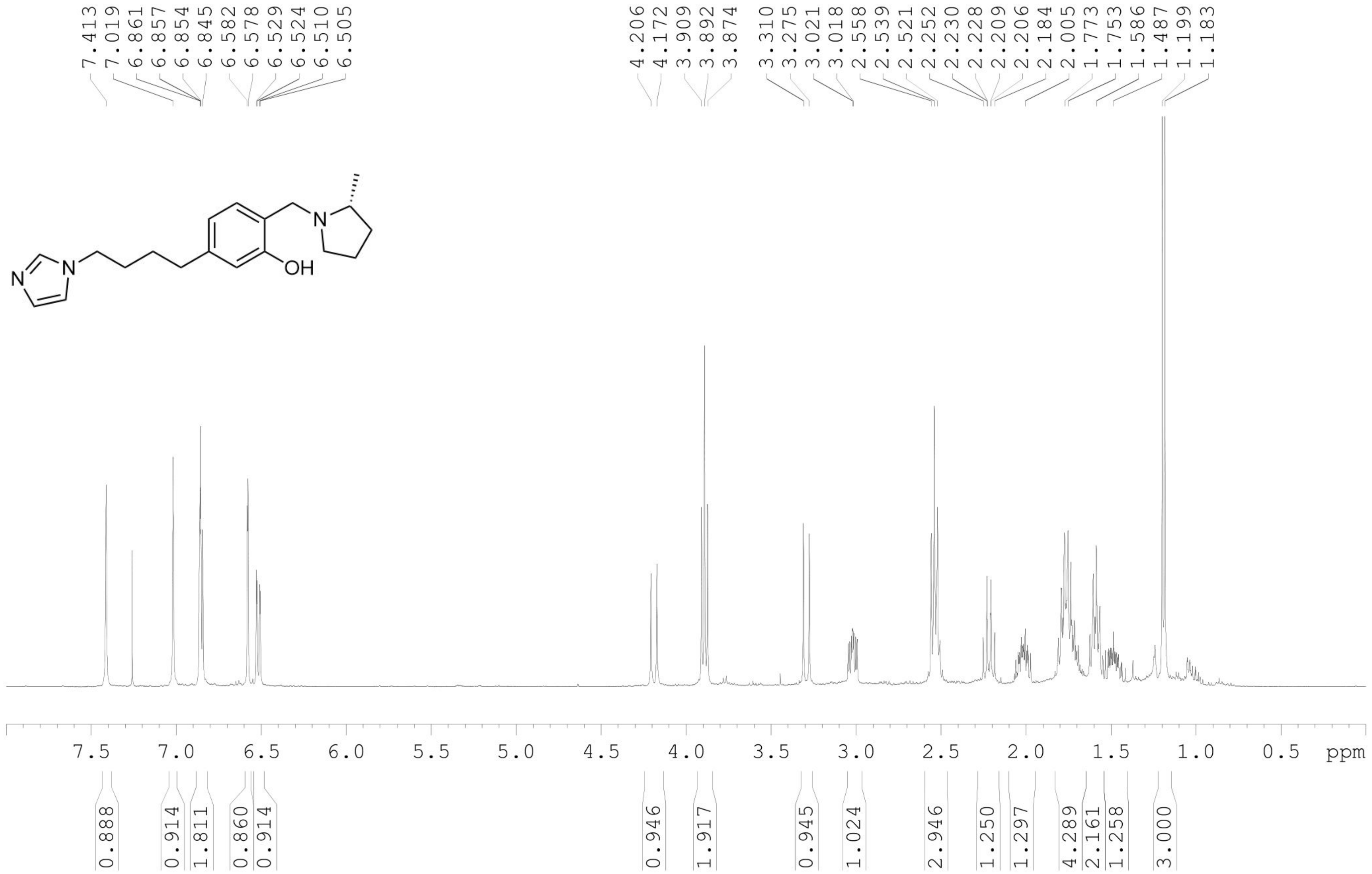

^{13}C NMR ($CDCl_3$, 100 MHz) of 11

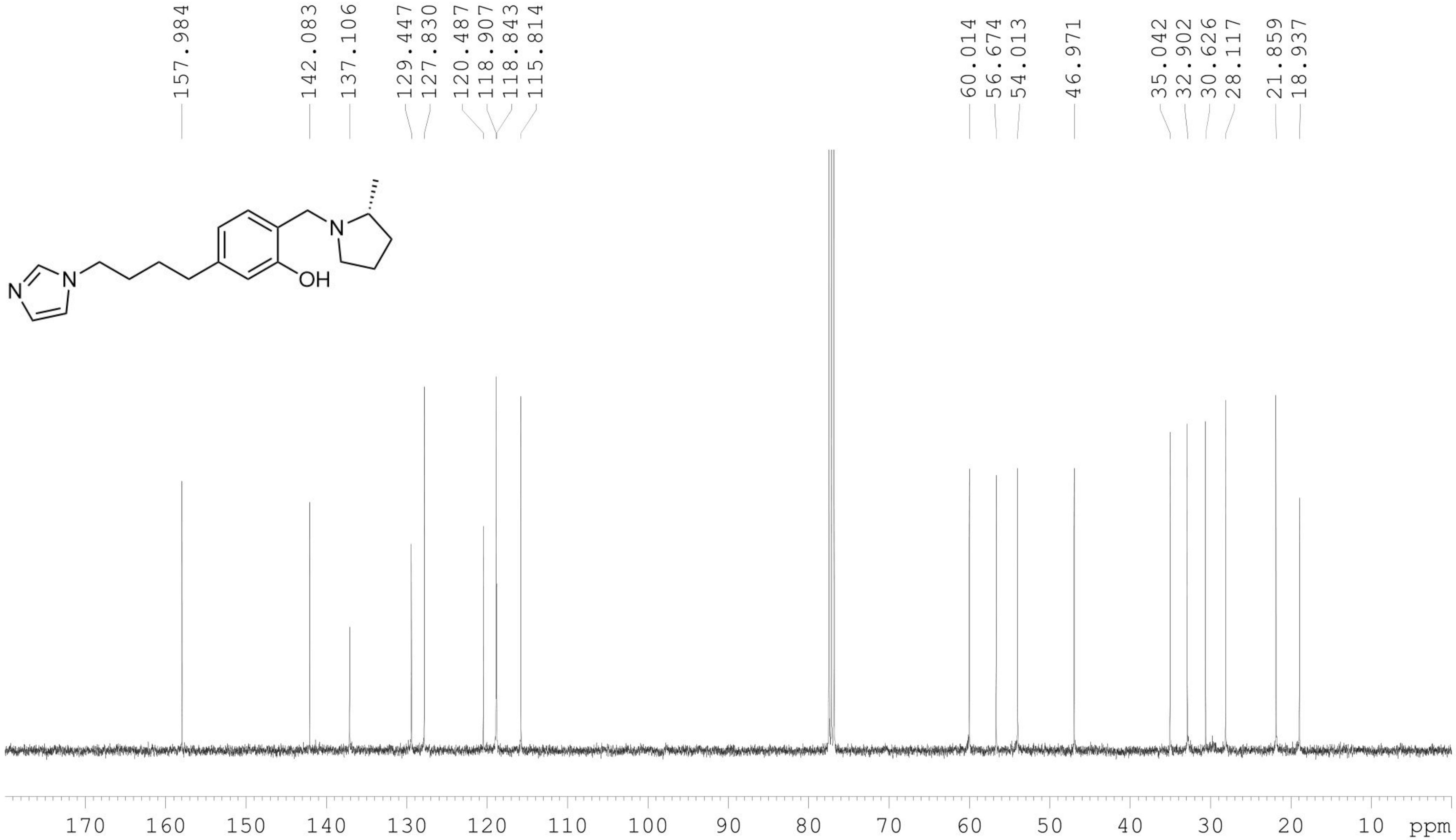

HPLC Trace (254 and 280 nm) of 11

Waters 2.1 x100 mm C18 UPLC Column

Water/MeOH + 0.1% Triethylamine

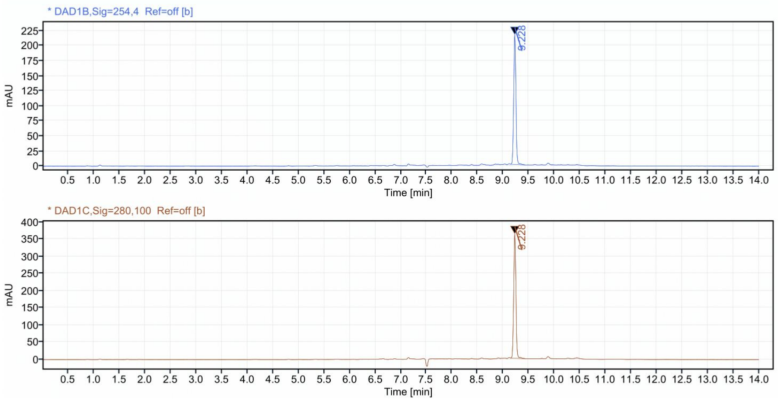

Signal: * DAD1B,Sig=254,4 Ref=off [b]

RT [min]	Peak Area	Type	Width [min]	Area	Height	Area%	Peak Area Percent
9.228	651.371	BB	0.29	651.37	215.25	100.00	100.00
			Sum	651.37			

Signal: * DAD1C,Sig=280,100 Ref=off [b]

RT [min]	Peak Area	Type	Width [min]	Area	Height	Area%	Peak Area Percent
9.228	1096.632	BB	0.30	1096.63	361.02	100.00	100.00
			Sum	1096.63			

^{1}H NMR ($CDCl_3$, 400 MHz) of 12

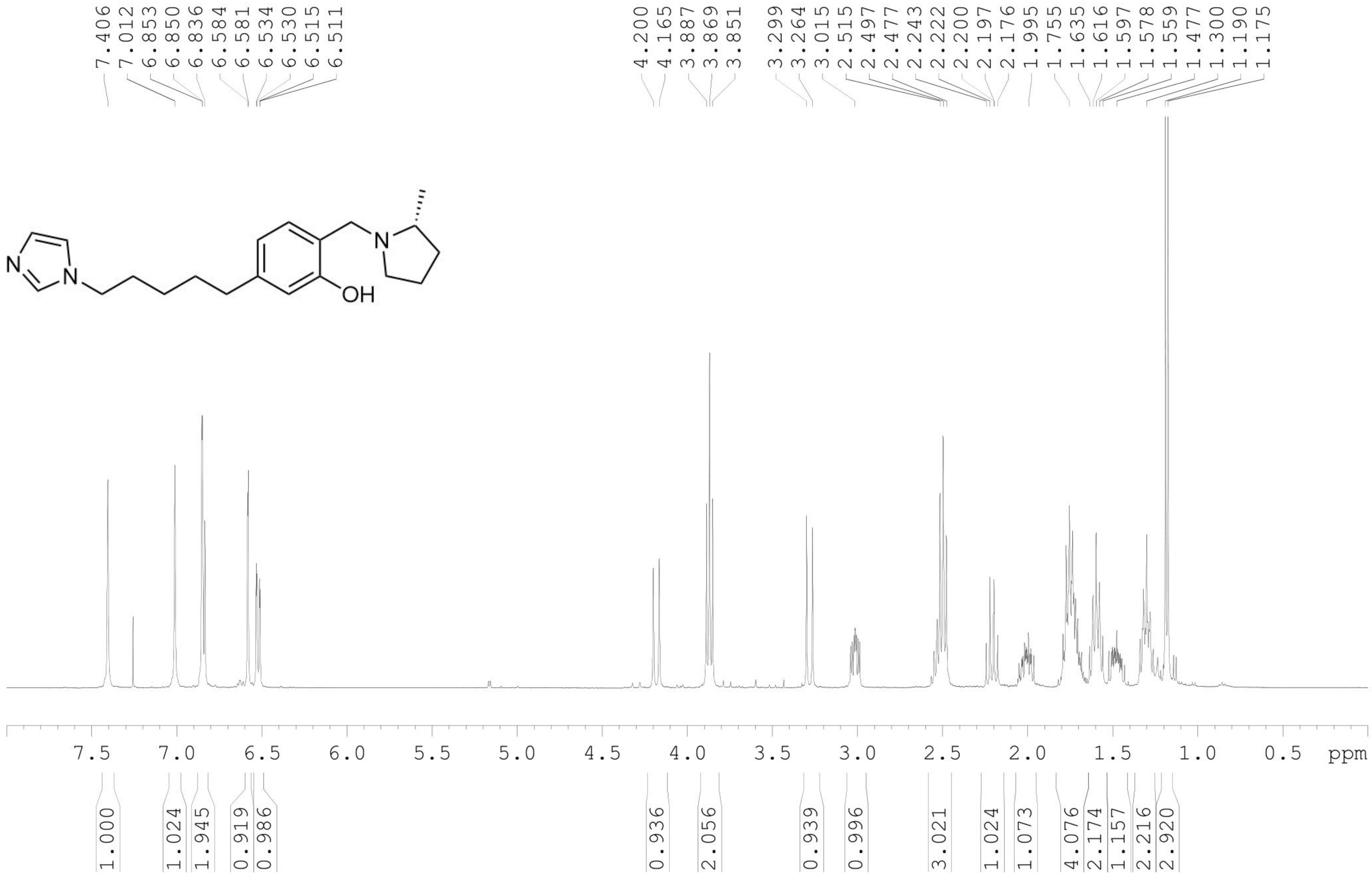

^{13}C NMR ($CDCl_3$, 100 MHz) of 12

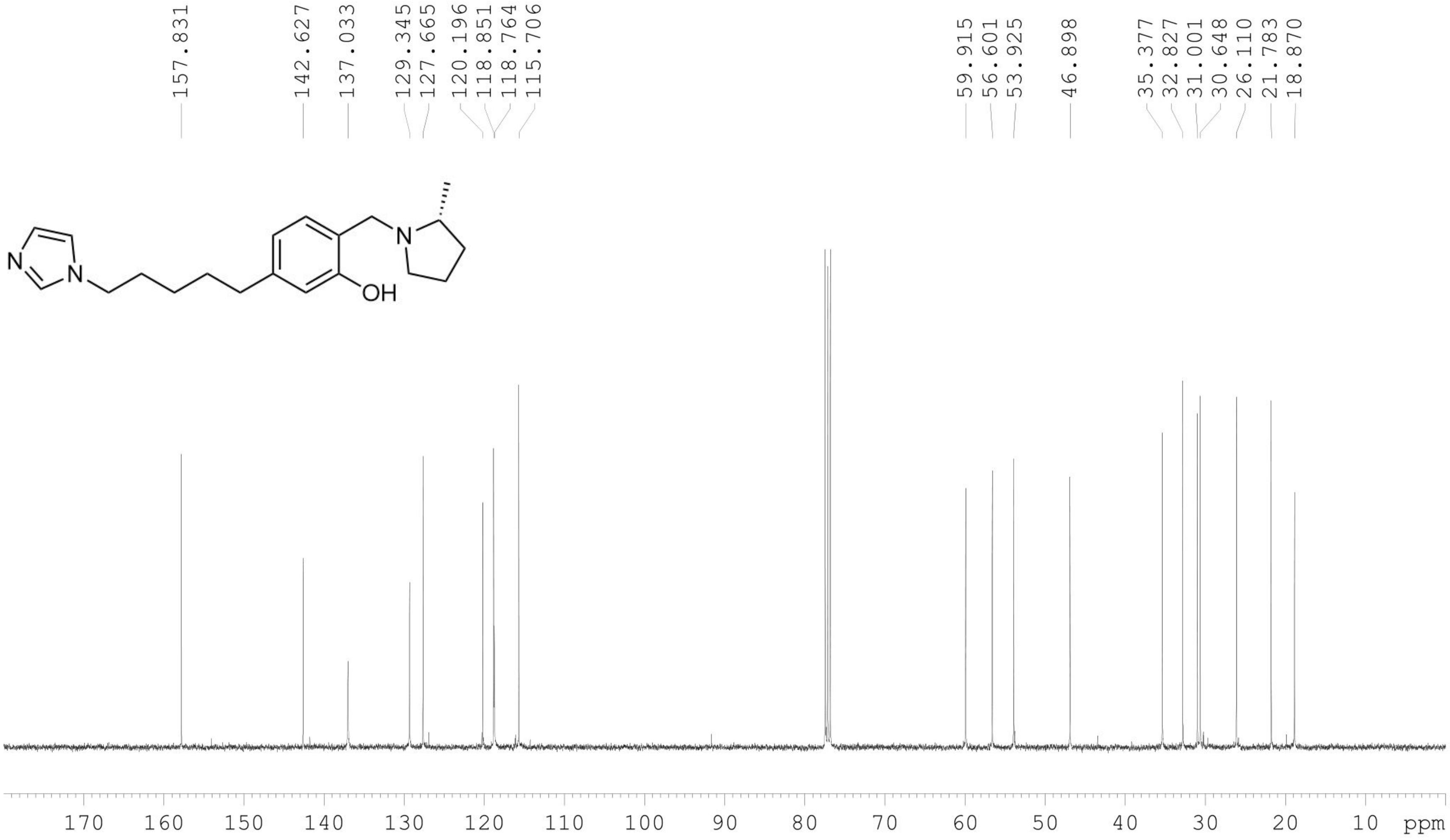

HPLC Trace (254 and 280 nm) of 12

Waters 2.1 x100 mm C18 UPLC Column

Water/MeOH + 0.1% Triethylamine

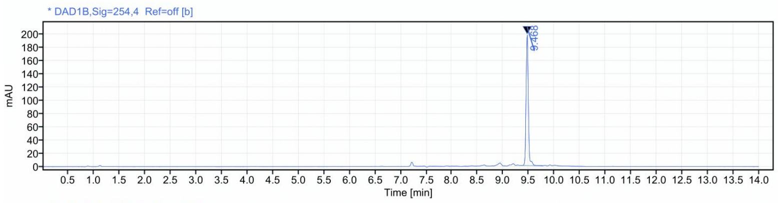

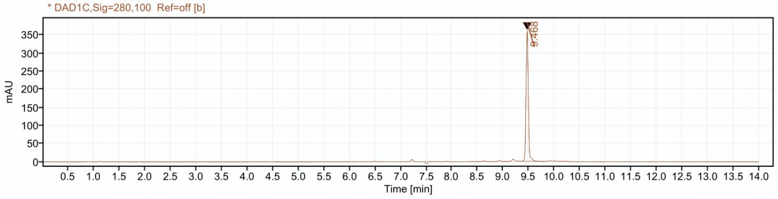

Signal: * DAD1B,Sig=254,4 Ref=off [b]

RT [min]	Peak Area	Type	Width [min]	Area	Height	Area%	Peak Area Percent
9.468	615.451	BB	0.44	615.45	198.49	100.00	100.00
			Sum	615.45			

Signal: * DAD1C,Sig=280,100 Ref=off [b]

RT [min]	Peak Area	Type	Width [min]	Area	Height	Area%	Peak Area Percent
9.468	1126.623	BB	0.45	1126.62	361.69	100.00	100.00
			Sum	1126.62			

^{1}H NMR ($CDCl_3$, 400 MHz) of 13

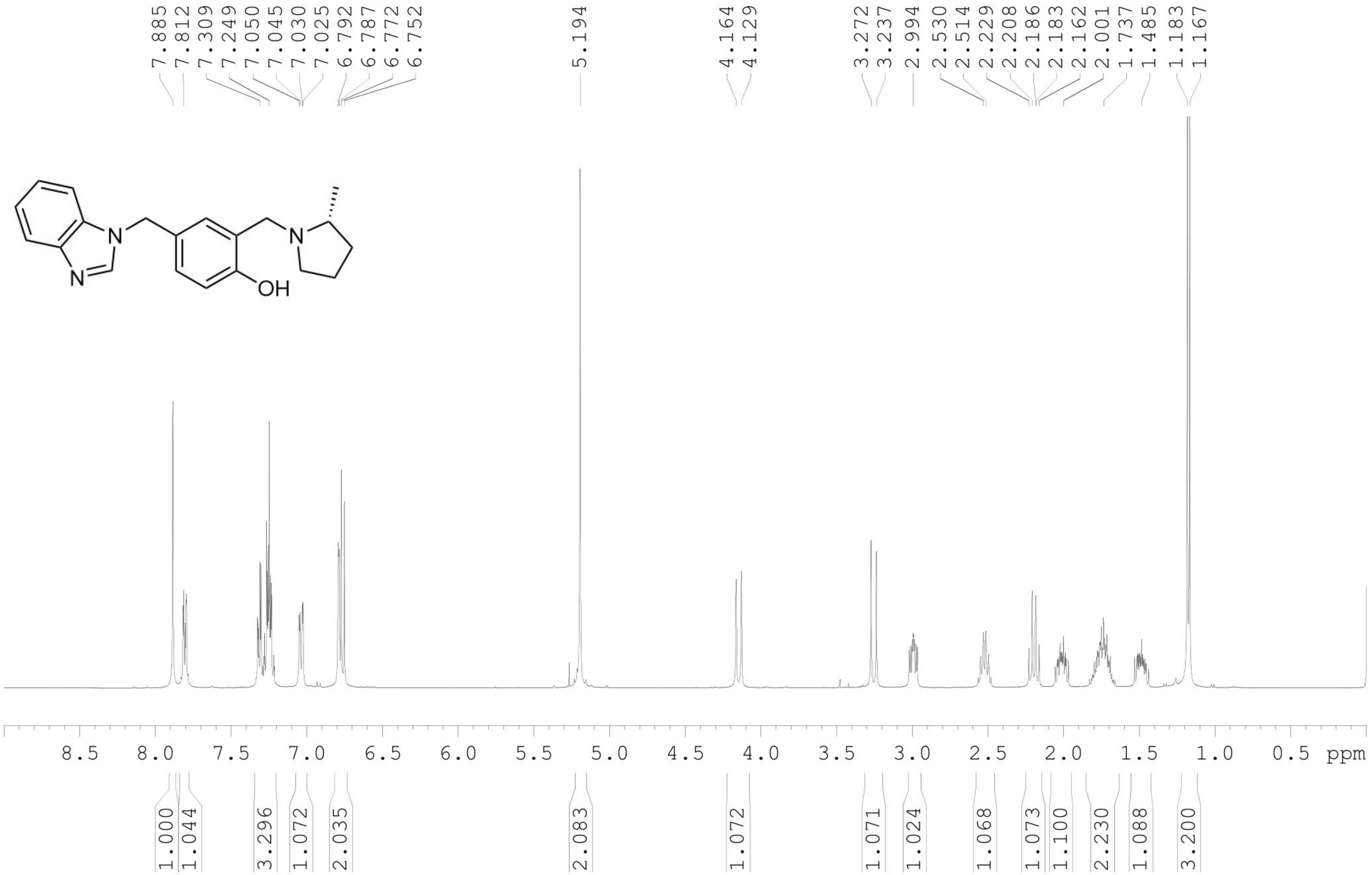

^{13}C NMR (CDCl$_3$, 100 MHz) of 13

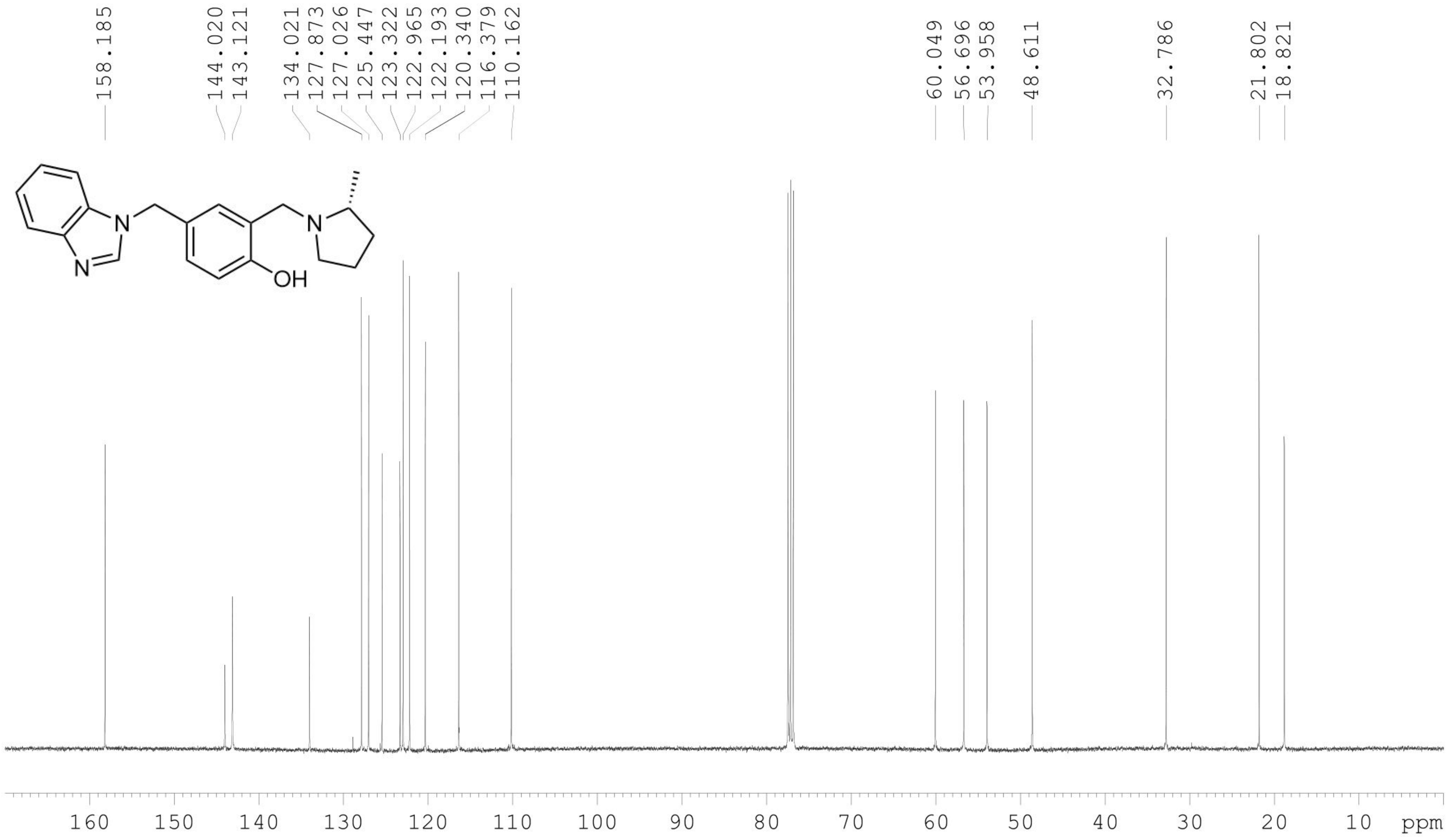

HPLC Trace (254 and 280 nm) of 13

Waters 2.1 x100 mm C18 UPLC Column

Water/MeOH + 0.1% Triethylamine

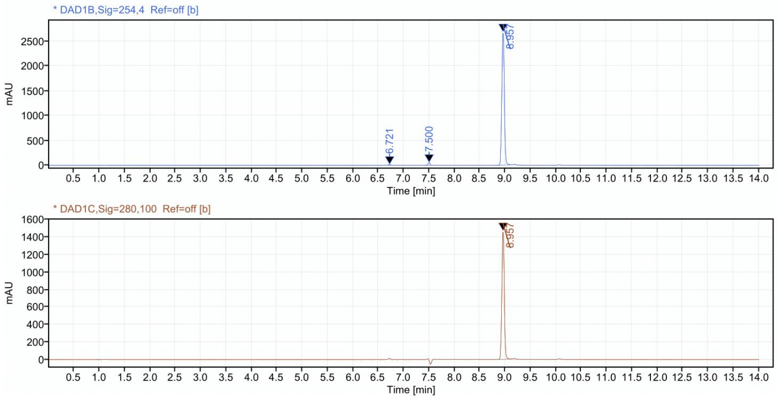

Signal: * DAD1B,Sig=254,4 Ref=off [b]

RT [min]	Peak Area	Type	Width [min]	Area	Height	Area%	Peak Area Percent
6.721	46.662	BB	0.10	46.66	17.24	0.50	0.50
7.500	166.231	BB	0.14	166.23	58.46	1.79	1.79
8.957	9083.571	BB	0.32	9083.57	2684.00	97.71	97.71
			Sum	9296.46			

Signal: * DAD1C,Sig=280,100 Ref=off [b]

RT [min]	Peak Area	Type	Width [min]	Area	Height	Area%	Peak Area Percent
8.957	4807.164	BB	0.32	4807.16	1474.59	100.00	100.00
			Sum	4807.16			

^{1}H NMR ($CDCl_3$, 400 MHz) of 14

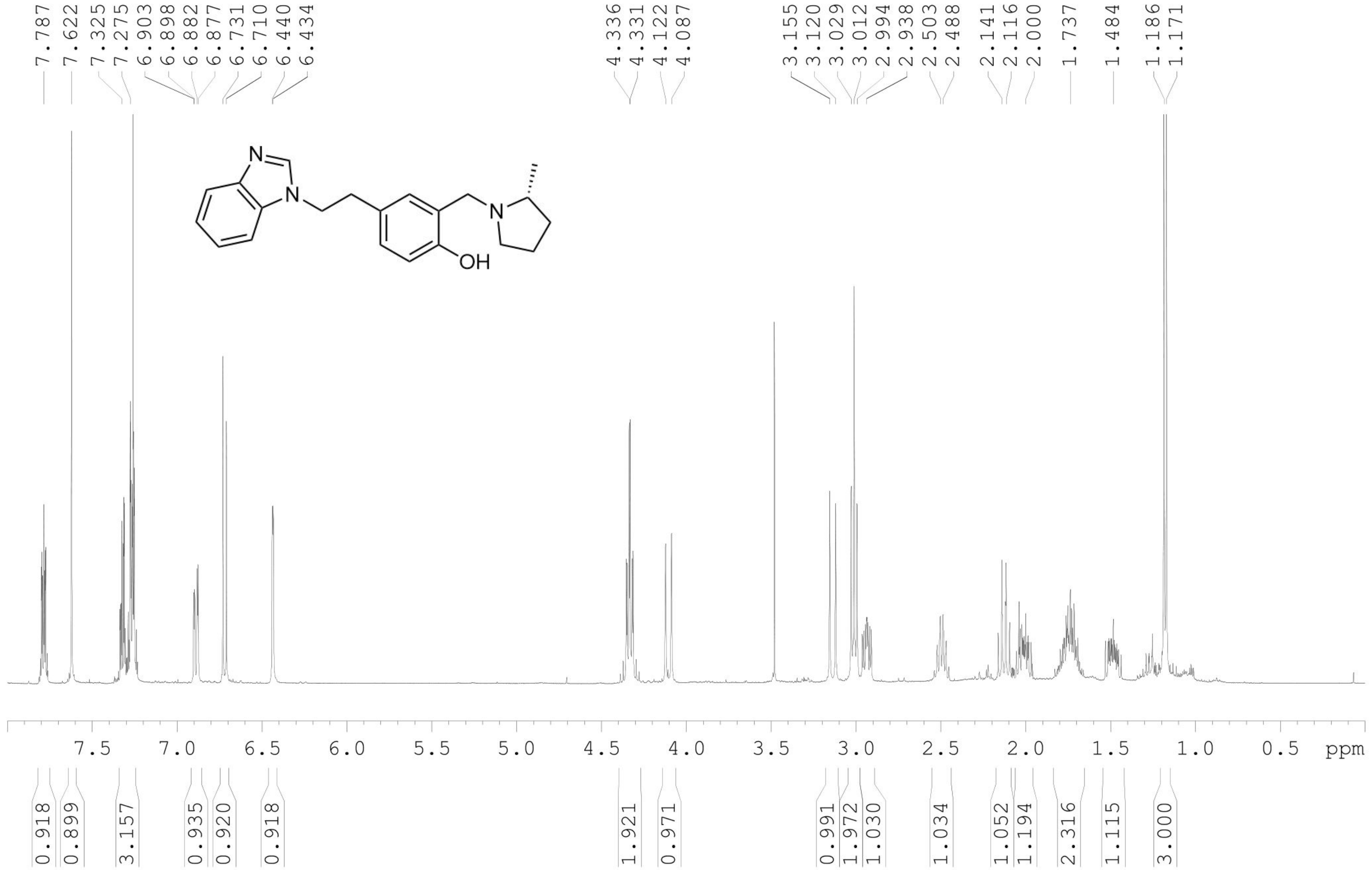

^{13}C NMR (CDCl$_3$, 100 MHz) of 14

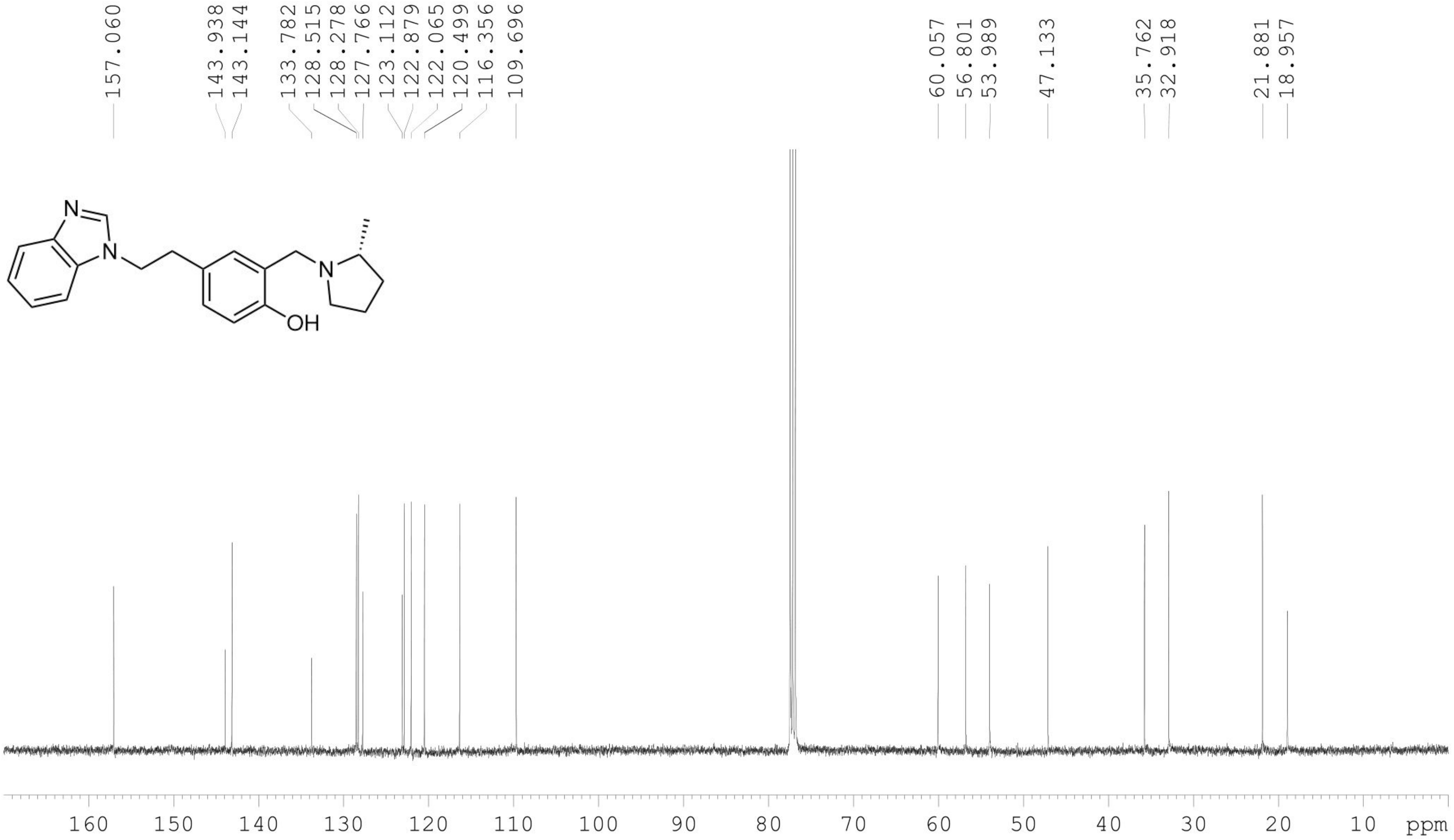

HPLC Trace (254 and 280 nm) of 14

Waters 2.1 x100 mm C18 UPLC Column

Water/MeOH + 0.1% Triethylamine

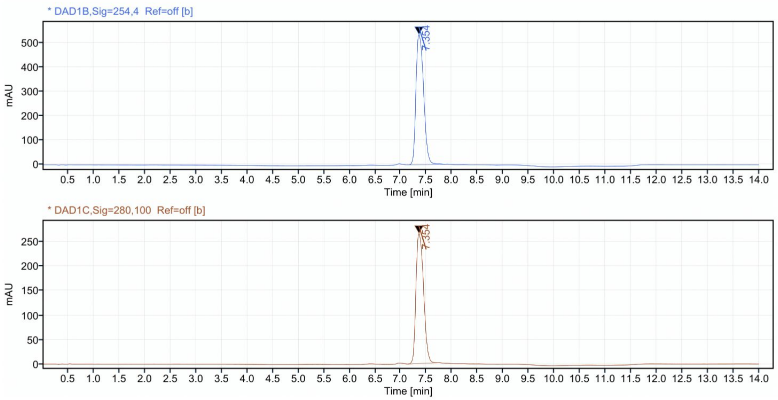

Signal: * DAD1B,Sig=254,4 Ref=off [b]

RT [min]	Peak Area	Type	Width [min]	Area	Height	Area%	Peak Area Percent
7.354	5552.631	BB	0.72	5552.63	533.67	100.00	100.00
			Sum	5552.63			

Signal: * DAD1C,Sig=280,100 Ref=off [b]

RT [min]	Peak Area	Type	Width [min]	Area	Height	Area%	Peak Area Percent
7.354	2748.794	BB	0.56	2748.79	266.62	100.00	100.00
			Sum	2748.79			

^{1}H NMR ($CDCl_3$, 400 MHz) of 15

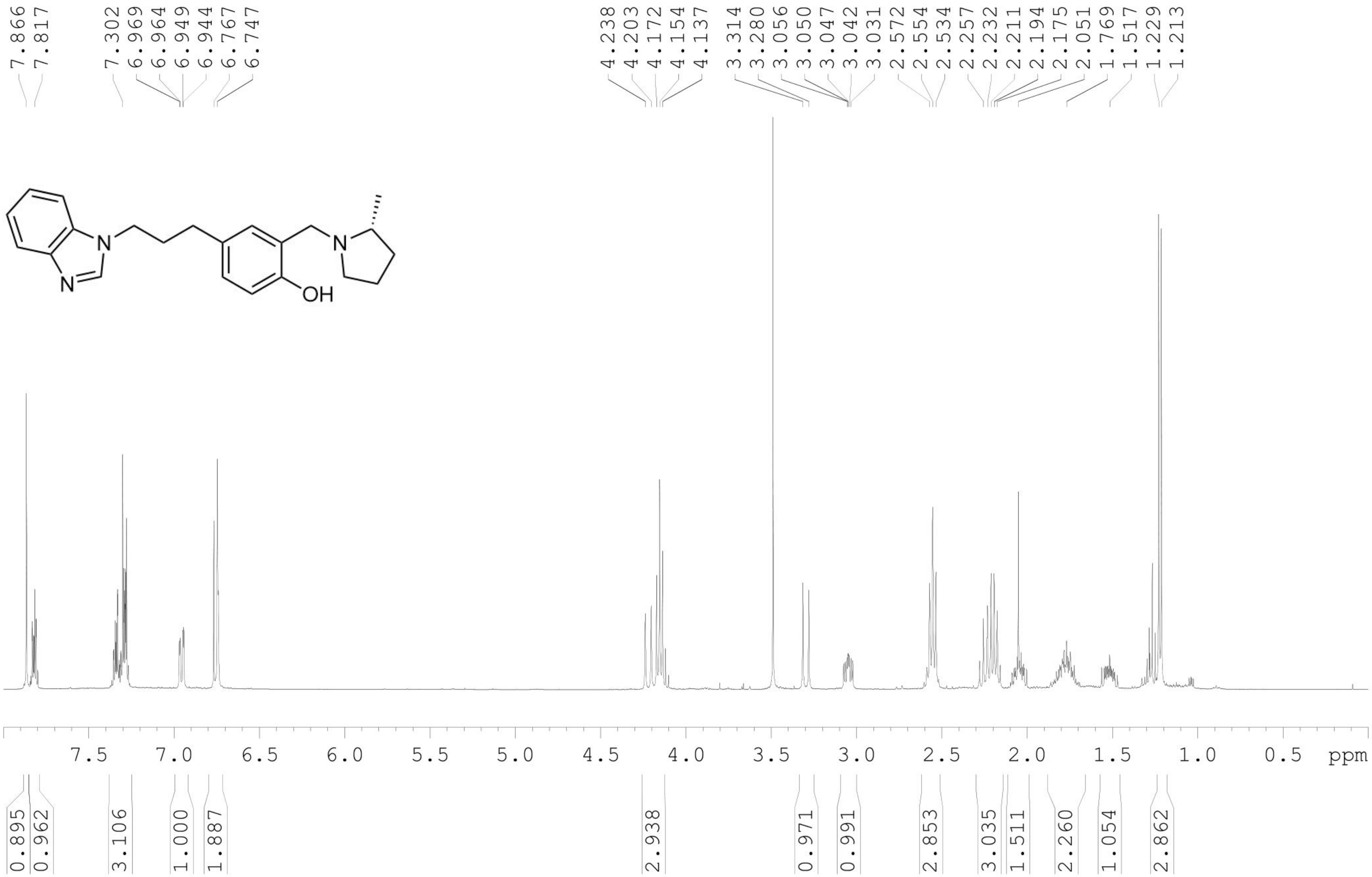

^{13}C NMR ($CDCl_3$, 100 MHz) of 15

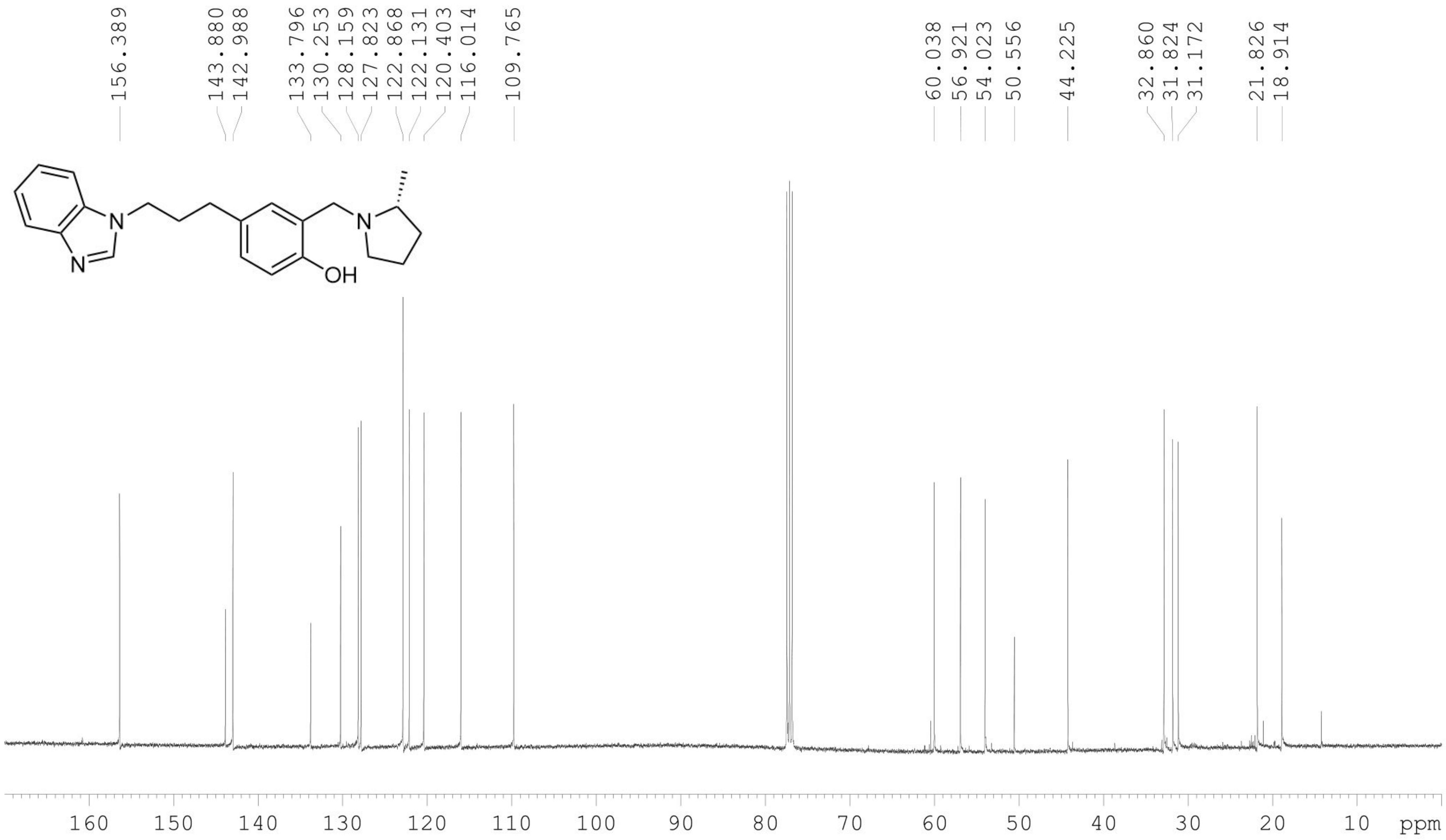

HPLC Trace (254 and 280 nm) of 15

Waters 2.1 x100 mm C18 UPLC Column

Water/MeOH + 0.1% Triethylamine

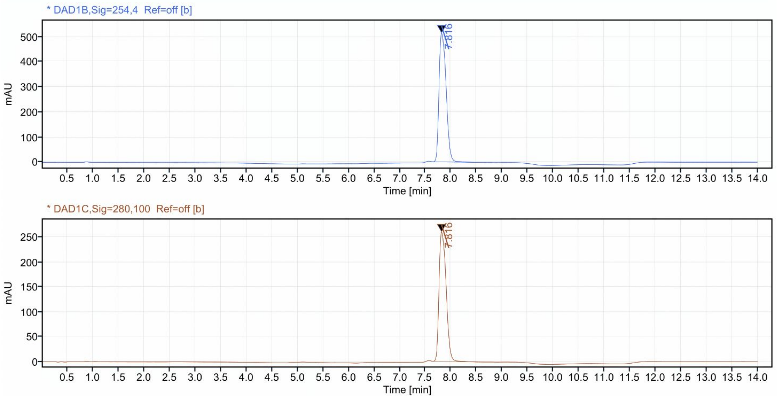

Signal: * DAD1B,Sig=254,4 Ref=off [b]

RT [min]	Peak Area	Type	Width [min]	Area	Height	Area%	Peak Area Percent
7.816	4943.499	BB	0.90	4943.50	512.24	100.00	100.00
			Sum	4943.50			

Signal: * DAD1C,Sig=280,100 Ref=off [b]

RT [min]	Peak Area	Type	Width [min]	Area	Height	Area%	Peak Area Percent
7.816	2512.721	BB	0.87	2512.72	259.96	100.00	100.00
			Sum	2512.72			

^{1}H NMR ($CDCl_3$, 400 MHz) of 16

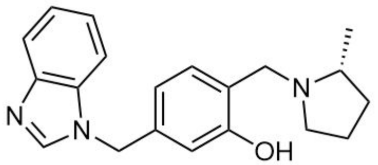

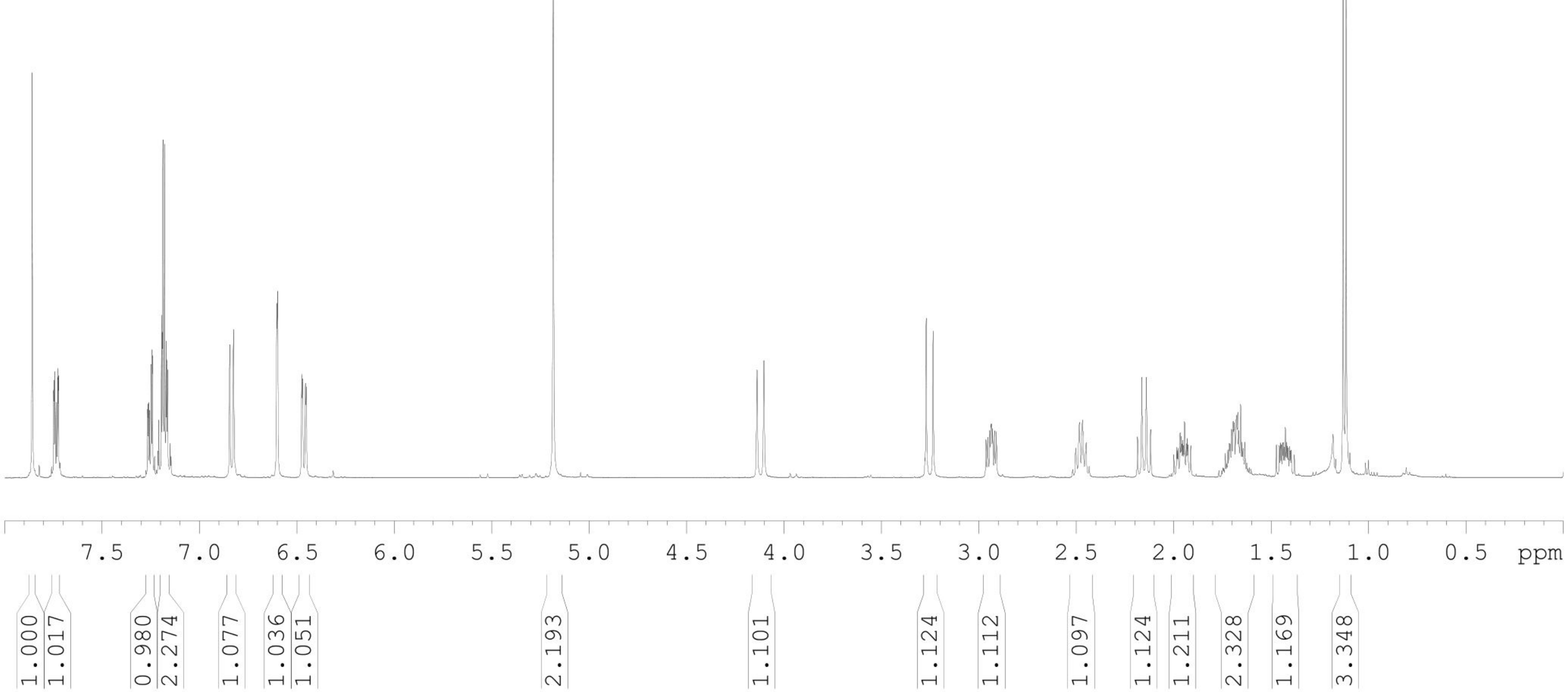

^{13}C NMR ($CDCl_3$, 100 MHz) of 16

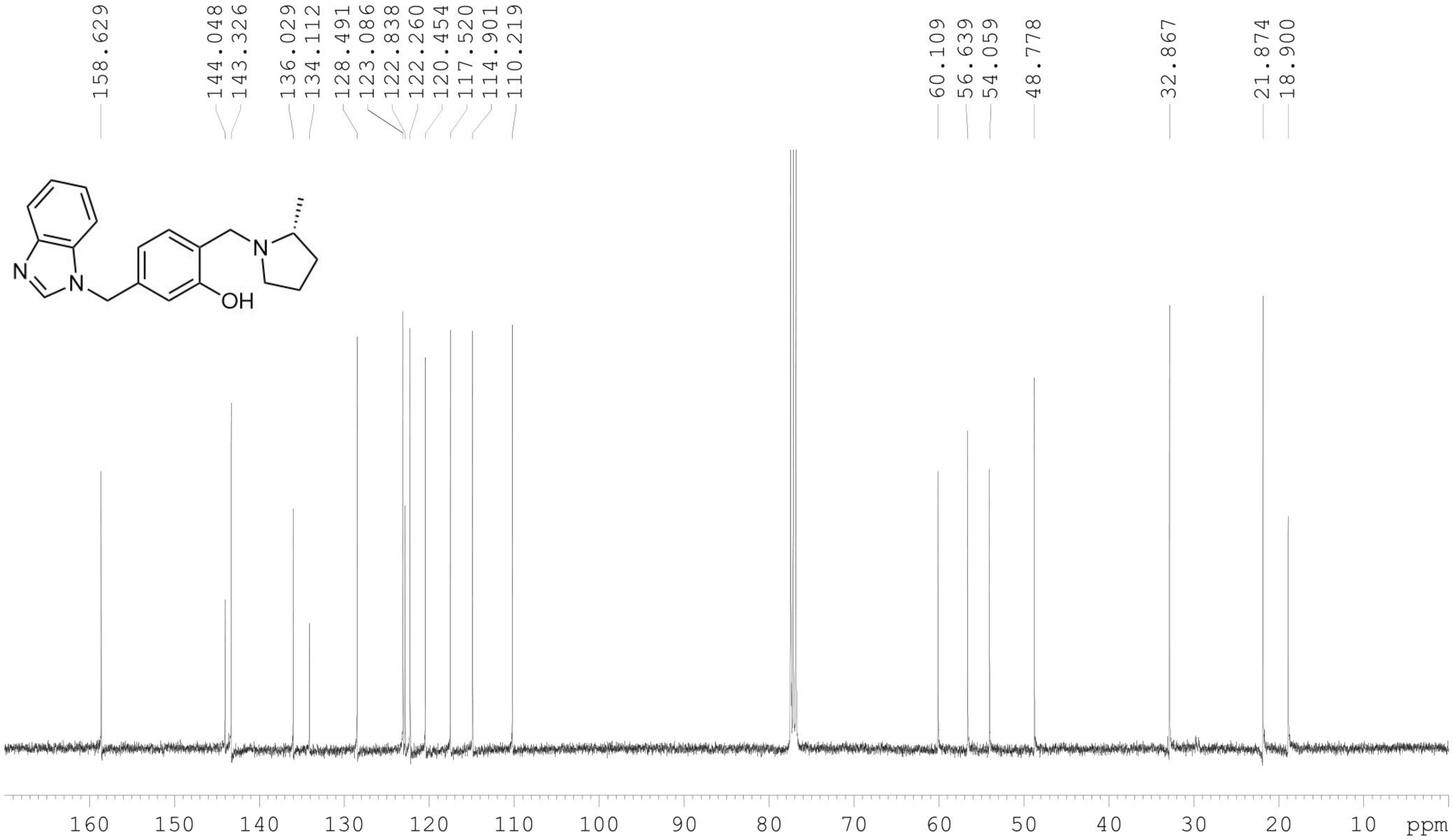

HPLC Trace (254 and 280 nm) of 16

Waters 2.1 x100 mm C18 UPLC Column

Water/MeOH + 0.1% Triethylamine

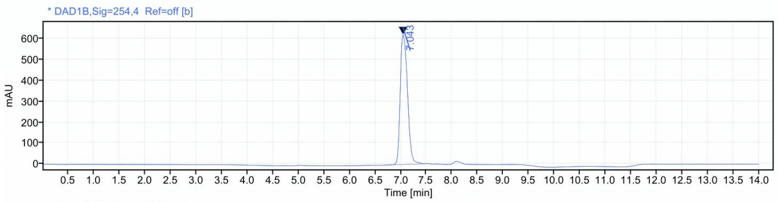

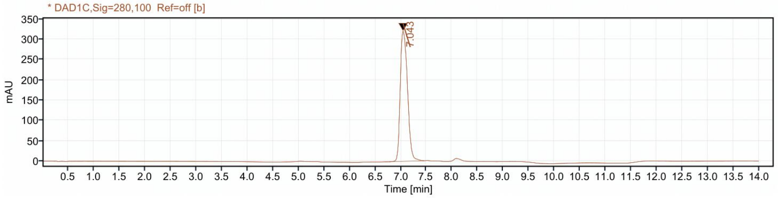

Signal: * DAD1B,Sig=254,4 Ref=off [b]

RT [min]	Peak Area	Type	Width [min]	Area	Height	Area%	Peak Area Percent
7.043	6107.296	BB	0.68	6107.30	618.02	100.00	100.00
			Sum	6107.30			

Signal: * DAD1C,Sig=280,100 Ref=off [b]

RT [min]	Peak Area	Type	Width [min]	Area	Height	Area%	Peak Area Percent
7.043	3173.579	BB	0.68	3173.58	321.50	100.00	100.00
			Sum	3173.58			

^{1}H NMR ($CDCl_3$, 400 MHz) of 17

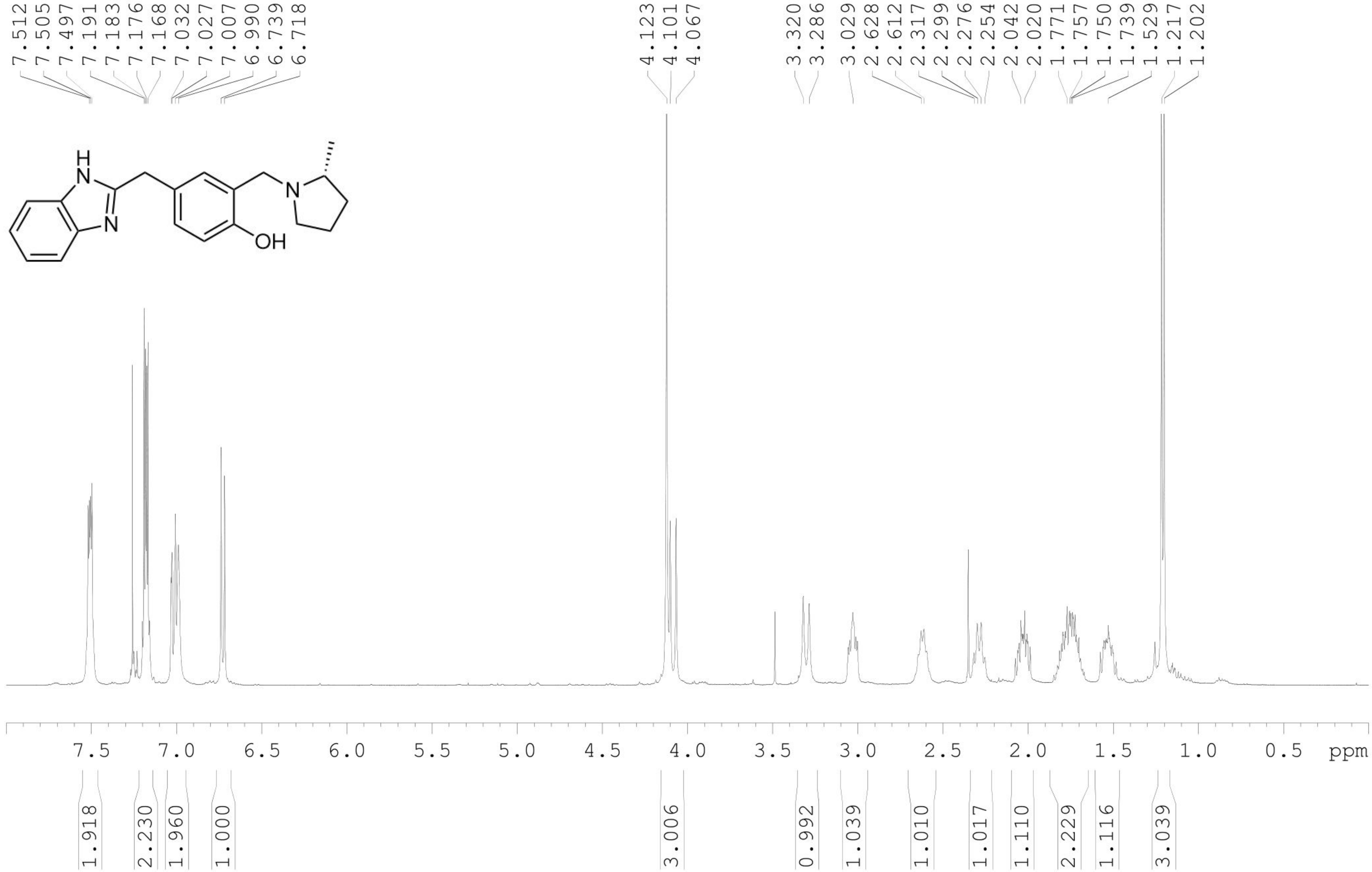

^{13}C NMR ($CDCl_3$, 100 MHz) of 17

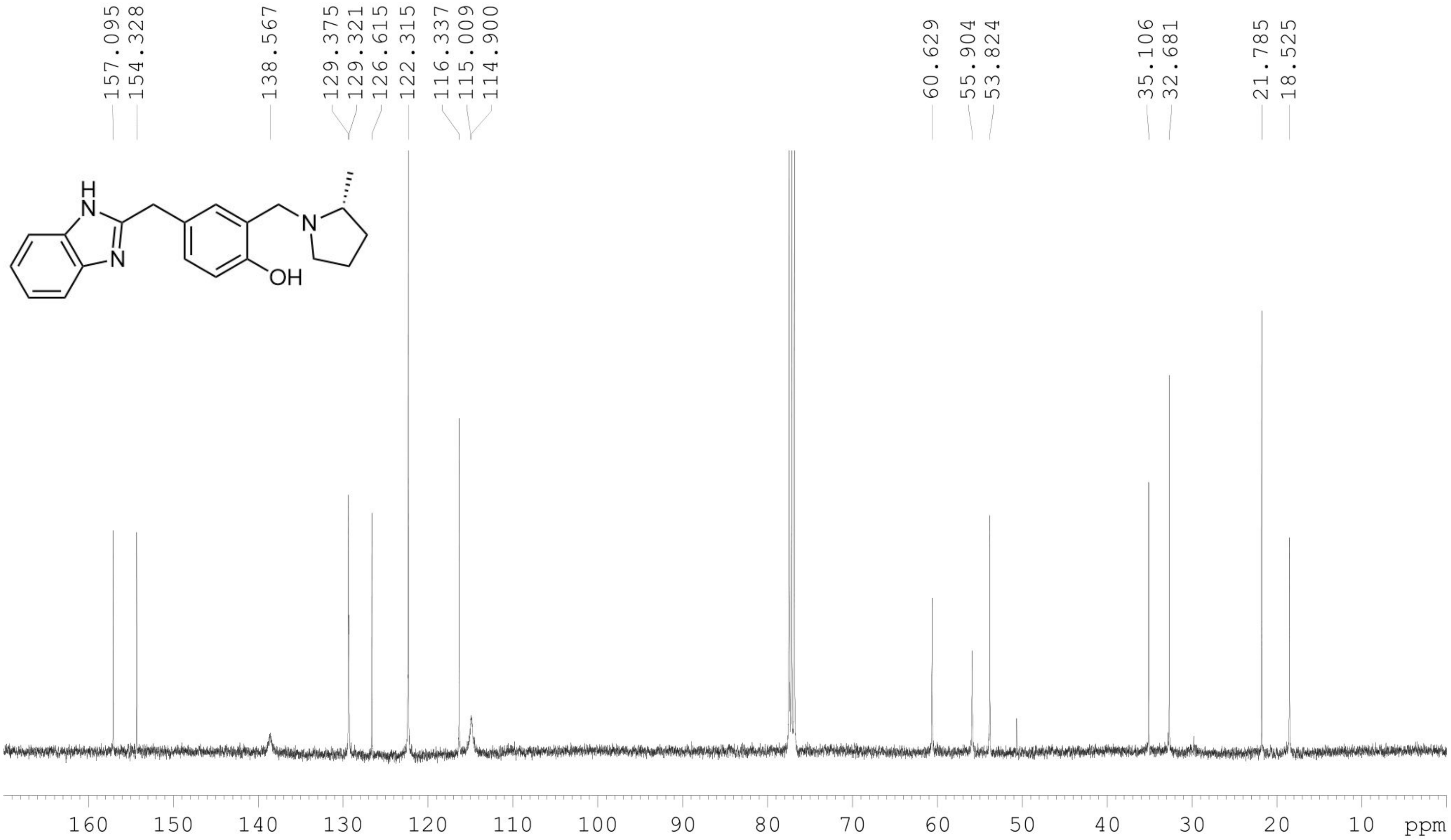

HPLC Trace (254 and 280 nm) of 17

Waters 2.1 x100 mm C18 UPLC Column

Water/MeOH + 0.1% Triethylamine

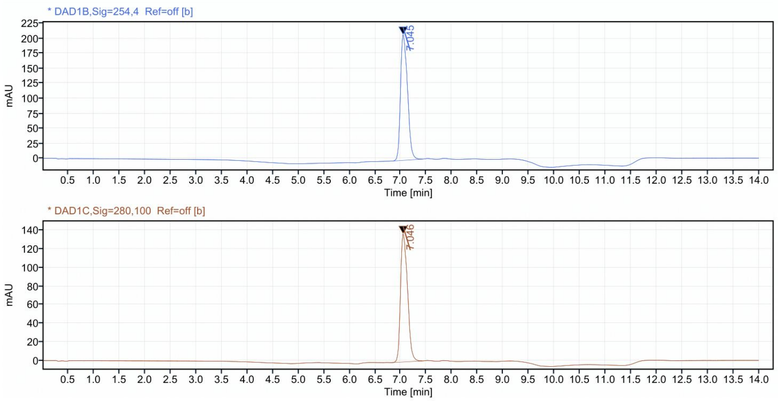

Signal: * DAD1B,Sig=254,4 Ref=off [b]

RT [min]	Peak Area	Type	Width [min]	Area	Height	Area%	Peak Area Percent
7.045	1979.098	BB	0.59	1979.10	209.45	100.00	100.00
			Sum	1979.10			

Signal: * DAD1C,Sig=280,100 Ref=off [b]

RT [min]	Peak Area	Type	Width [min]	Area	Height	Area%	Peak Area Percent
7.046	1305.181	BB	0.58	1305.18	137.30	100.00	100.00
			Sum	1305.18			

^{1}H NMR ($CDCl_3$, 400 MHz) of 18

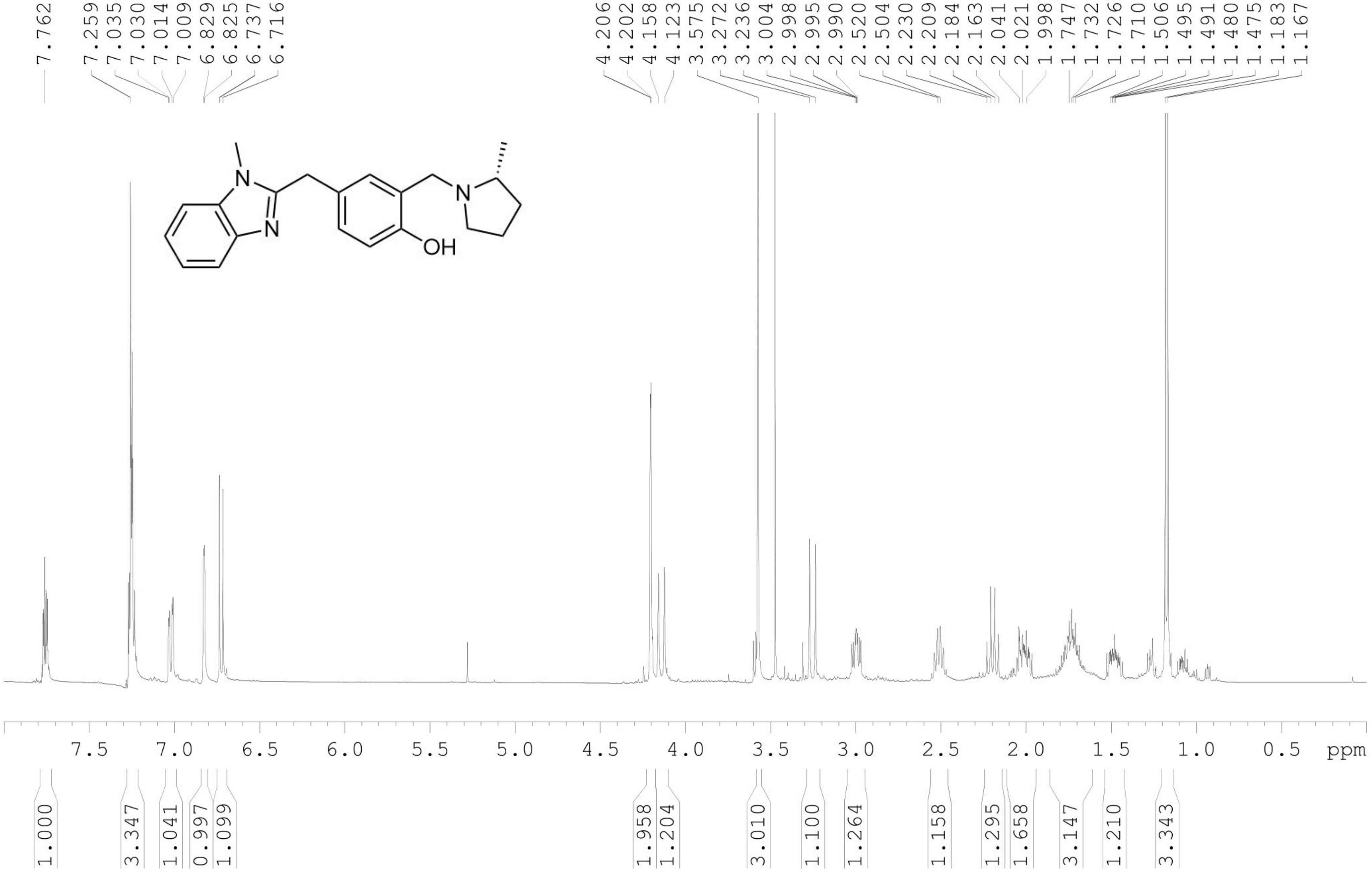

^{13}C NMR ($CDCl_3$, 100 MHz) of 18

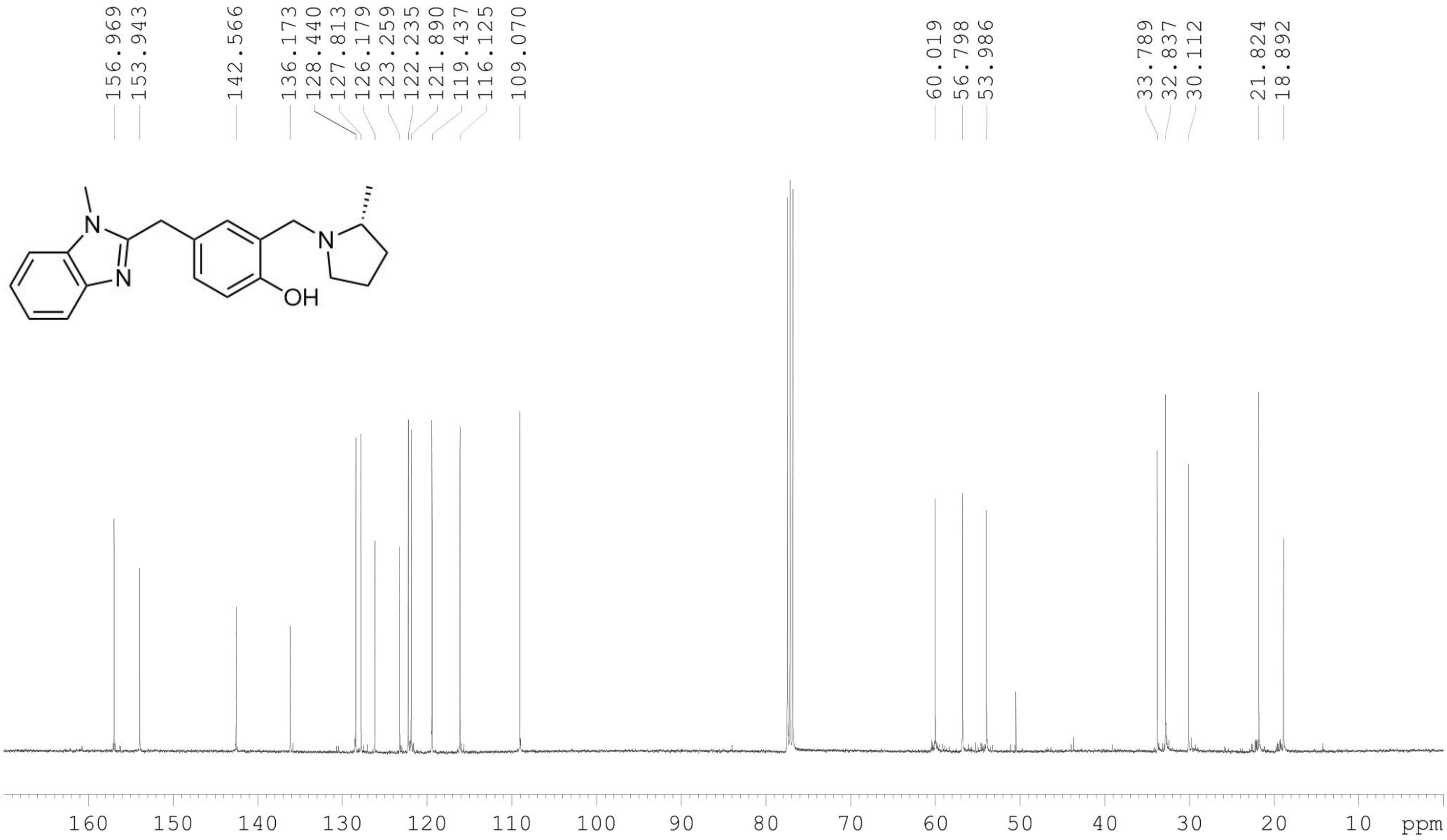

HPLC Trace (254 and 280 nm) of 18

Waters 2.1 x100 mm C18 UPLC Column

Water/MeOH + 0.1% Triethylamine

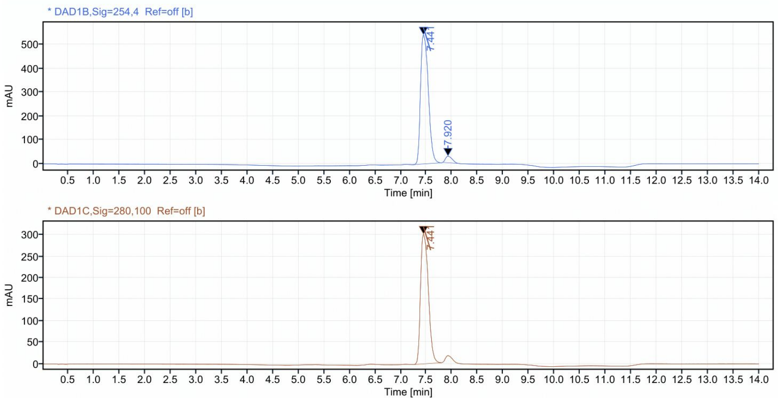

Signal: * DAD1B,Sig=254,4 Ref=off [b]

RT [min]	Peak Area	Type	Width [min]	Area	Height	Area%	Peak Area Percent
7.441	5724.796	BB	0.54	5724.80	540.31	95.85	95.85
7.920	248.112	MM m	0.31	248.11	27.30	4.15	4.15
			Sum	5972.91			

Signal: * DAD1C,Sig=280,100 Ref=off [b]

RT [min]	Peak Area	Type	Width [min]	Area	Height	Area%	Peak Area Percent
7.441	3205.904	BB	0.54	3205.90	301.75	100.00	100.00
			Sum	3205.90			

^{1}H NMR ($CDCl_3$, 400 MHz) of 19

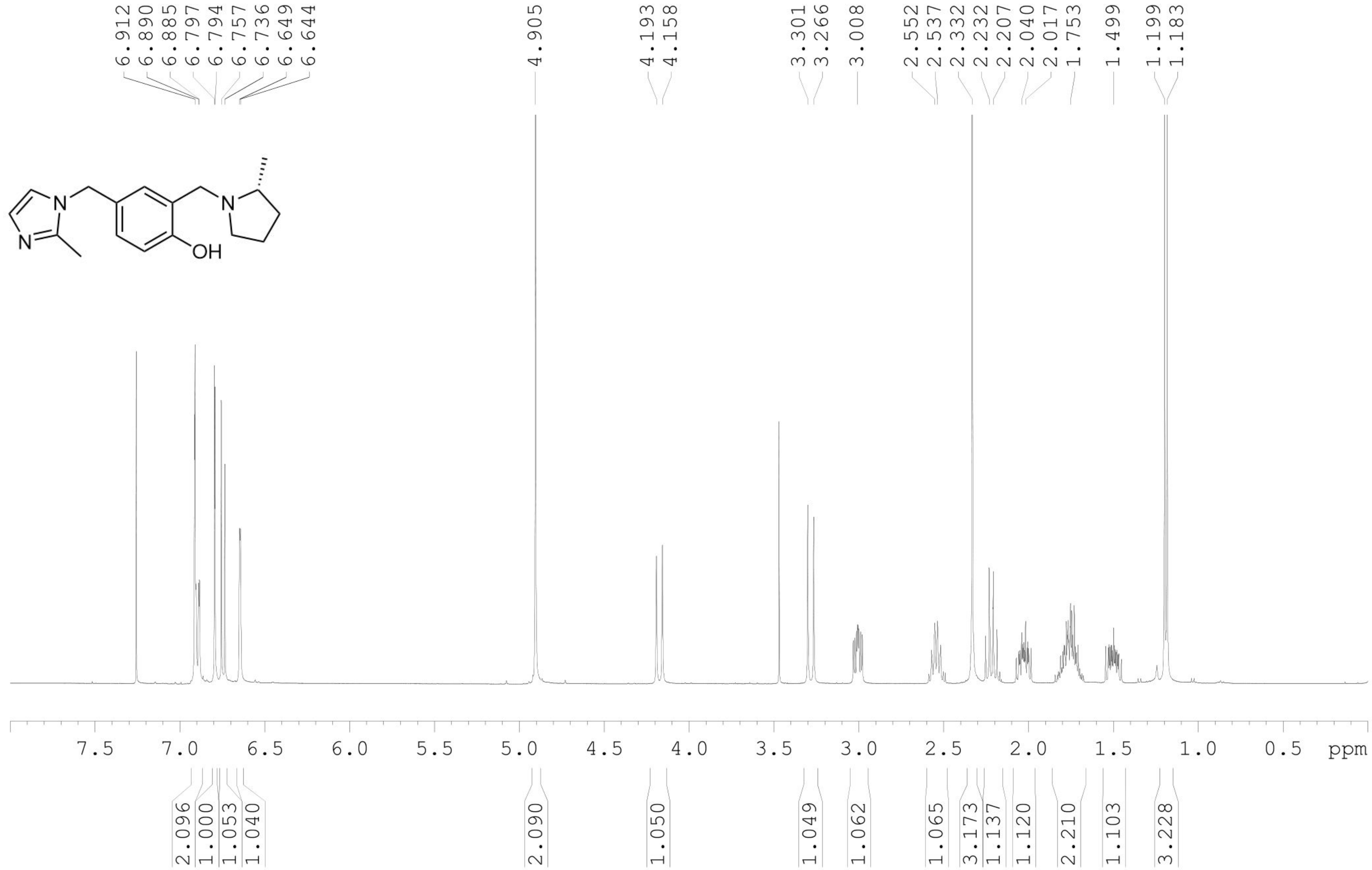

^{13}C NMR ($CDCl_3$, 100 MHz) of 19

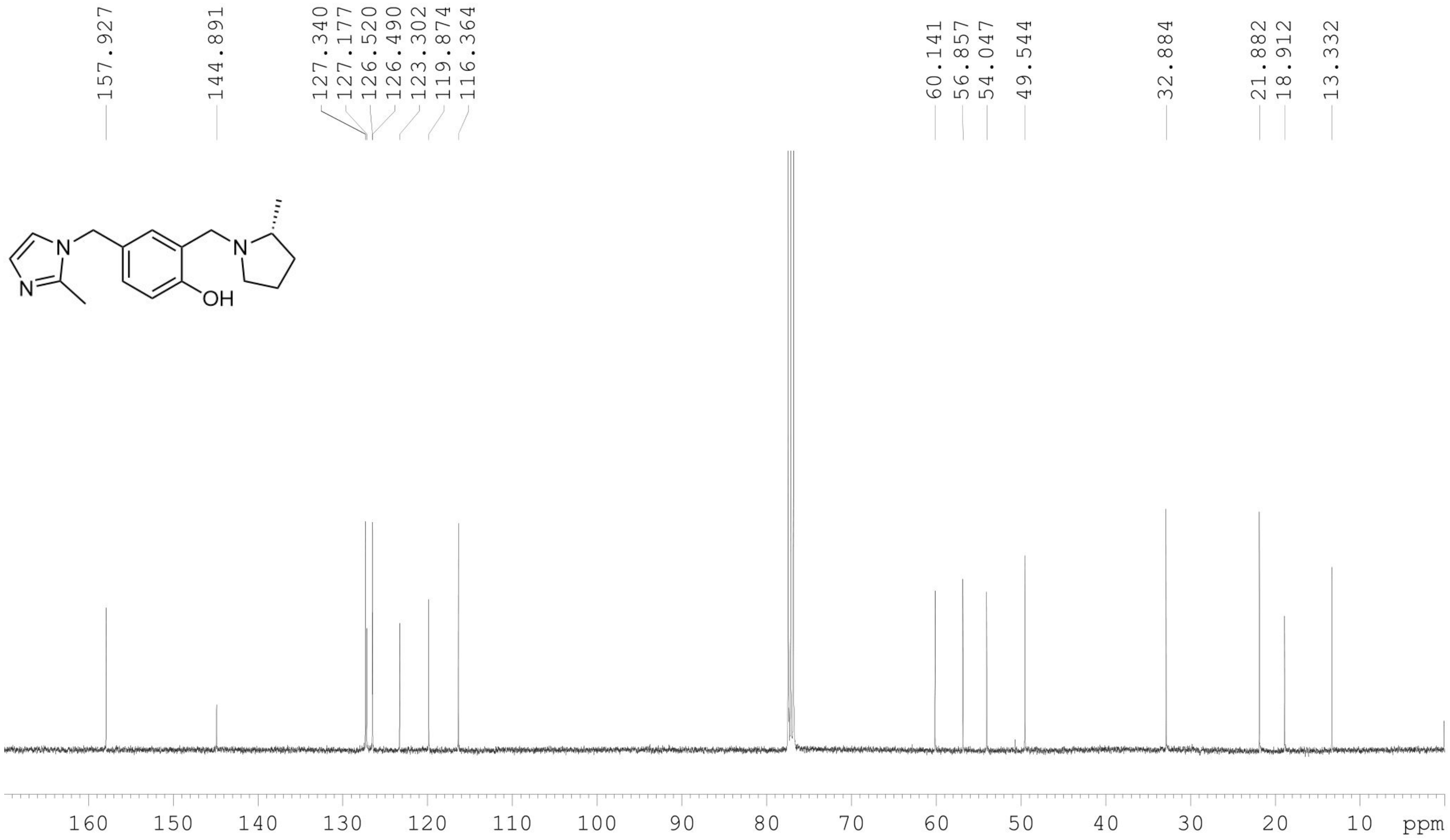

HPLC Trace (254 and 280 nm) of 19

Waters 2.1 x100 mm C18 UPLC Column

Water/MeOH + 0.1% Triethylamine

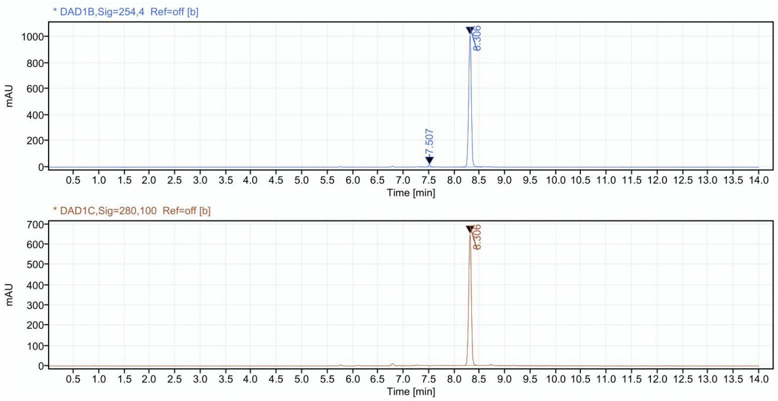

Signal: * DAD1B,Sig=254,4 Ref=off [b]

RT [min]	Peak Area	Type	Width [min]	Area	Height	Area%	Peak Area Percent
7.507	41.397	BB	0.13	41.40	16.47	1.11	1.11
8.306	3689.570	BB	0.41	3689.57	1012.37	98.89	98.89
			Sum	3730.97			

Signal: * DAD1C,Sig=280,100 Ref=off [b]

RT [min]	Peak Area	Type	Width [min]	Area	Height	Area%	Peak Area Percent
8.306	2382.434	BB	0.41	2382.43	651.98	100.00	100.00
			Sum	2382.43			

^{1}H NMR ($CDCl_3$, 400 MHz) of 20

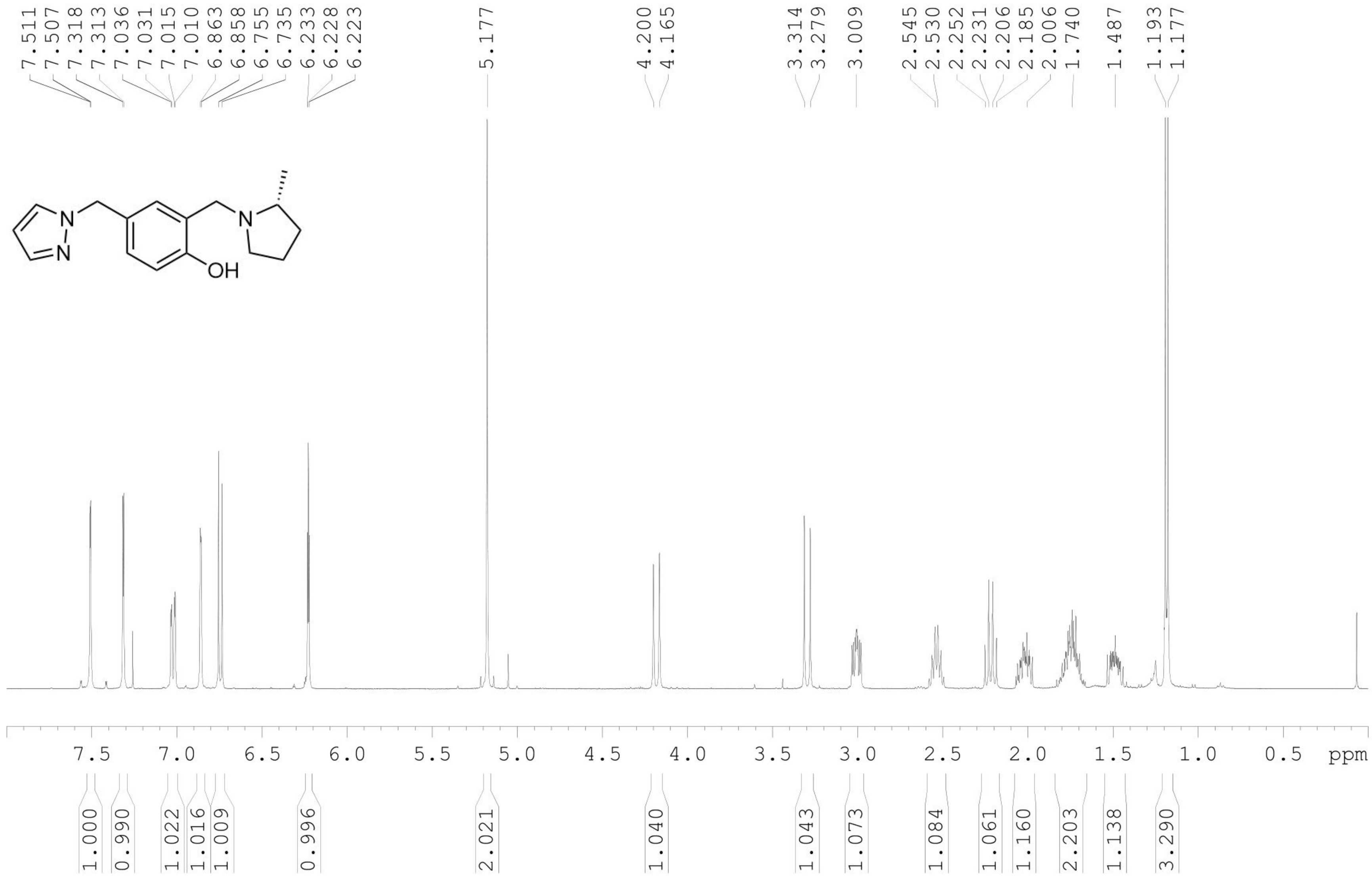

^{13}C NMR ($CDCl_3$, 100 MHz) of 20

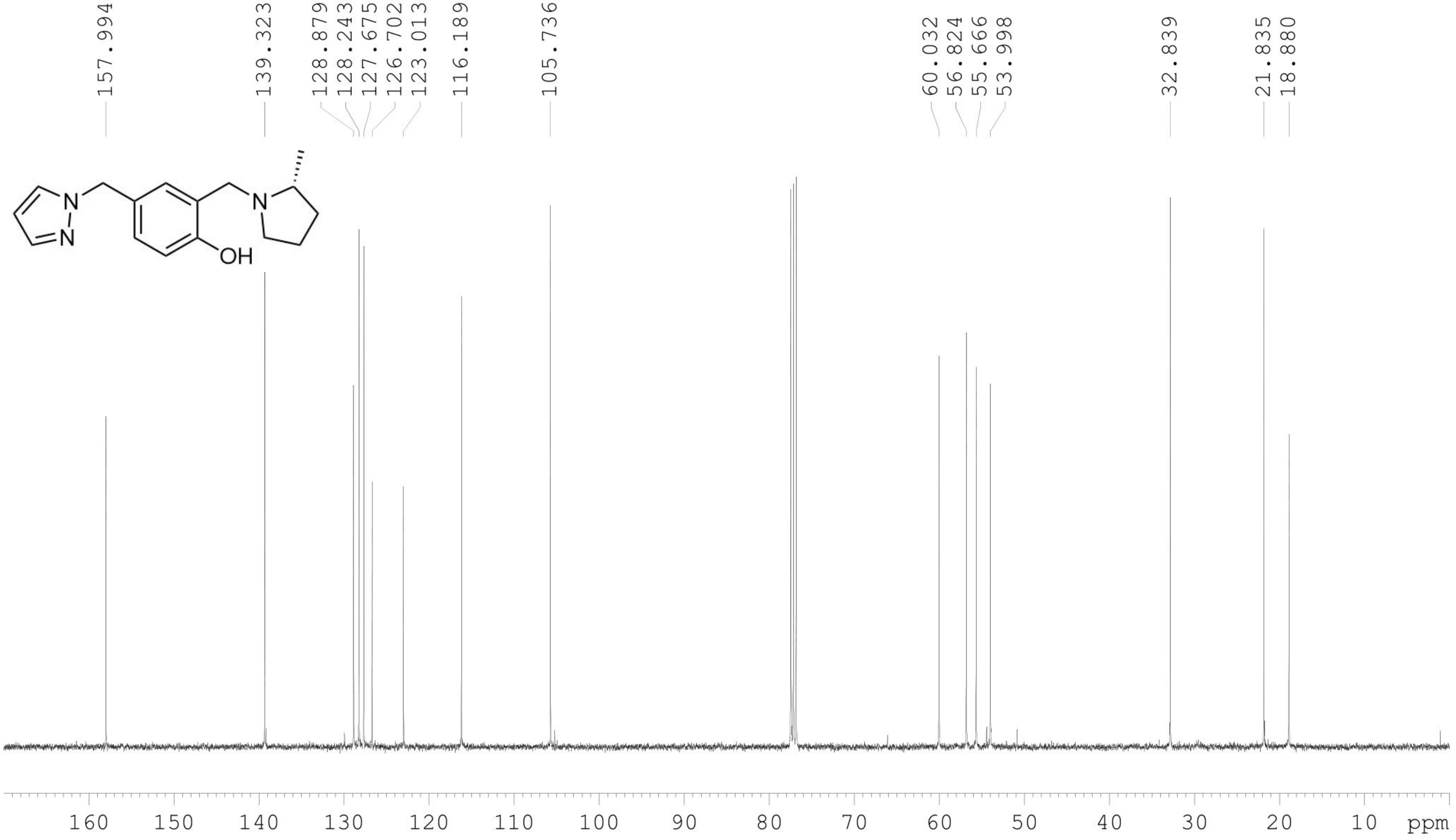

HPLC Trace (254 and 280 nm) of 20

Waters 2.1 x100 mm C18 UPLC Column

Water/MeOH + 0.1% Triethylamine

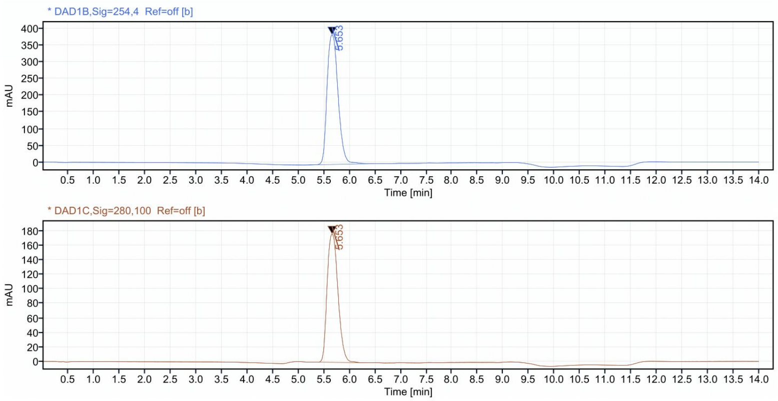

Signal: * DAD1B,Sig=254,4 Ref=off [b]

RT [min]	Peak Area	Type	Width [min]	Area	Height	Area%	Peak Area Percent
5.653	5849.371	BB	1.27	5849.37	390.18	100.00	100.00
			Sum	5849.37			

Signal: * DAD1C,Sig=280,100 Ref=off [b]

RT [min]	Peak Area	Type	Width [min]	Area	Height	Area%	Peak Area Percent
5.653	2609.453	BB	0.86	2609.45	176.92	100.00	100.00
			Sum	2609.45			

^{1}H NMR (DMSO, 400 MHz) of 21

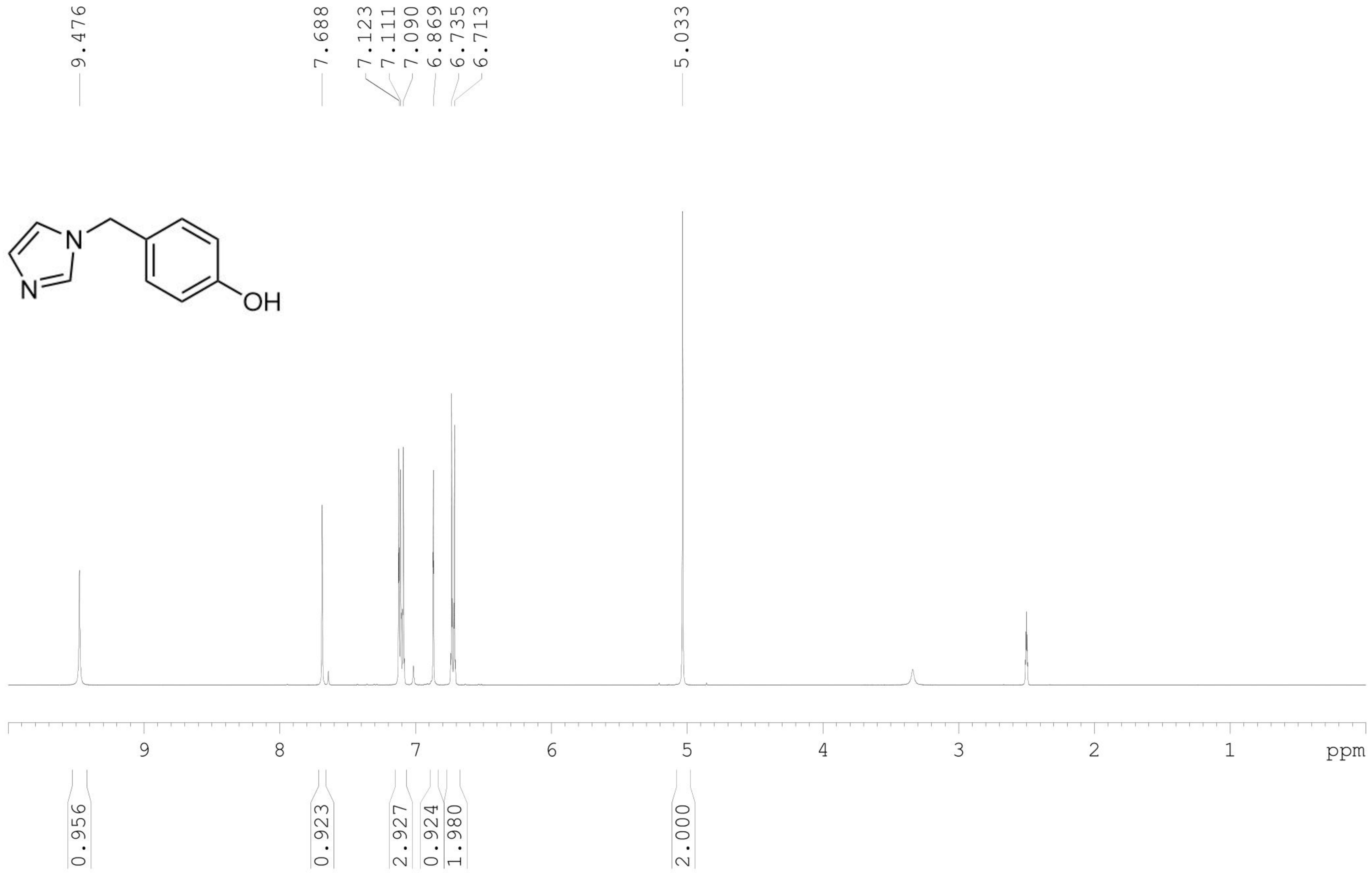

^{13}C NMR (DMSO, 100 MHz) of 21

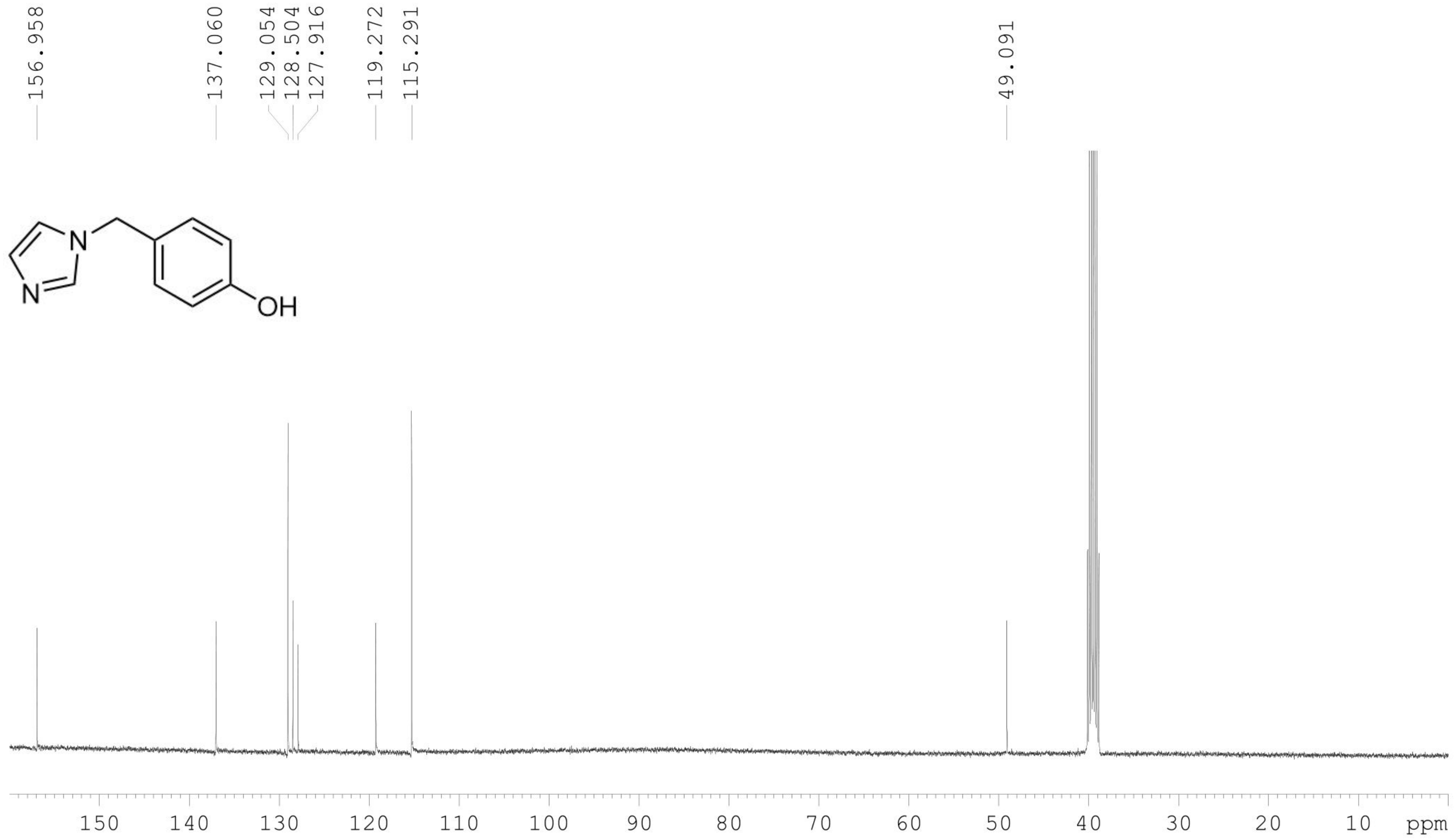

^{1}H NMR ($CDCl_3$, 400 MHz) of 23

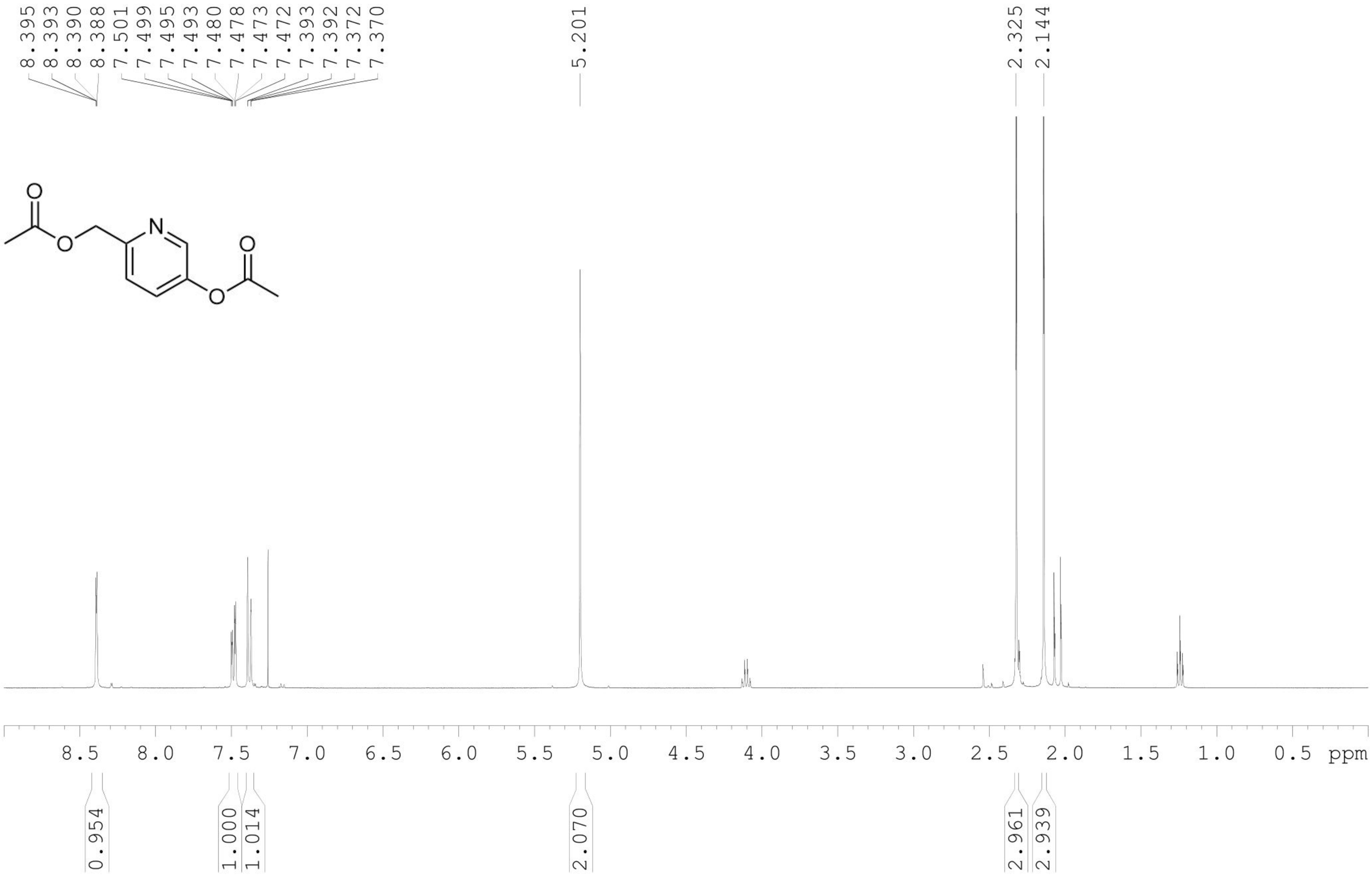

^{13}C NMR ($CDCl_3$, 100 MHz) of 23

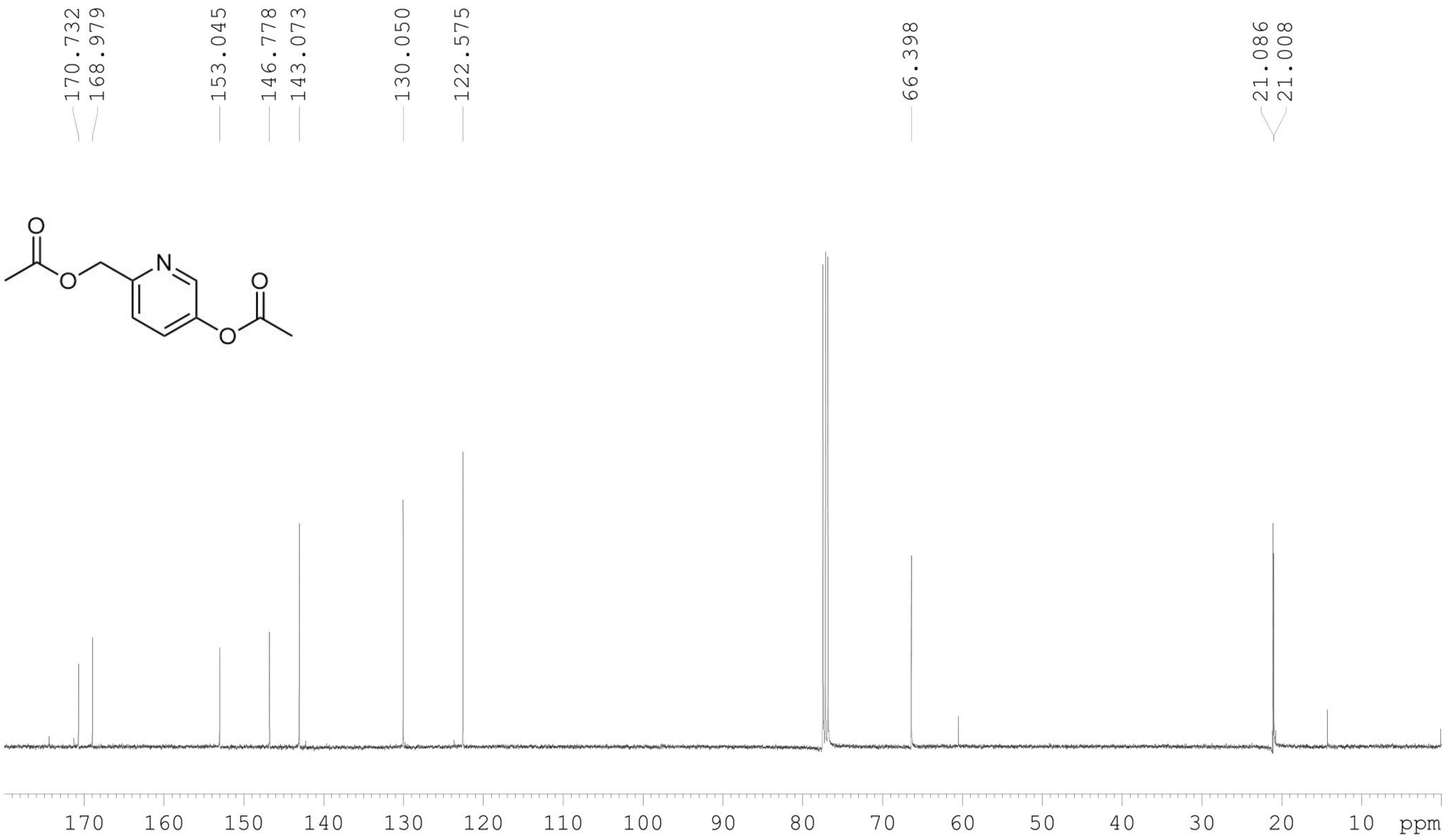

^{1}H NMR (DMSO, 400 MHz) of 25

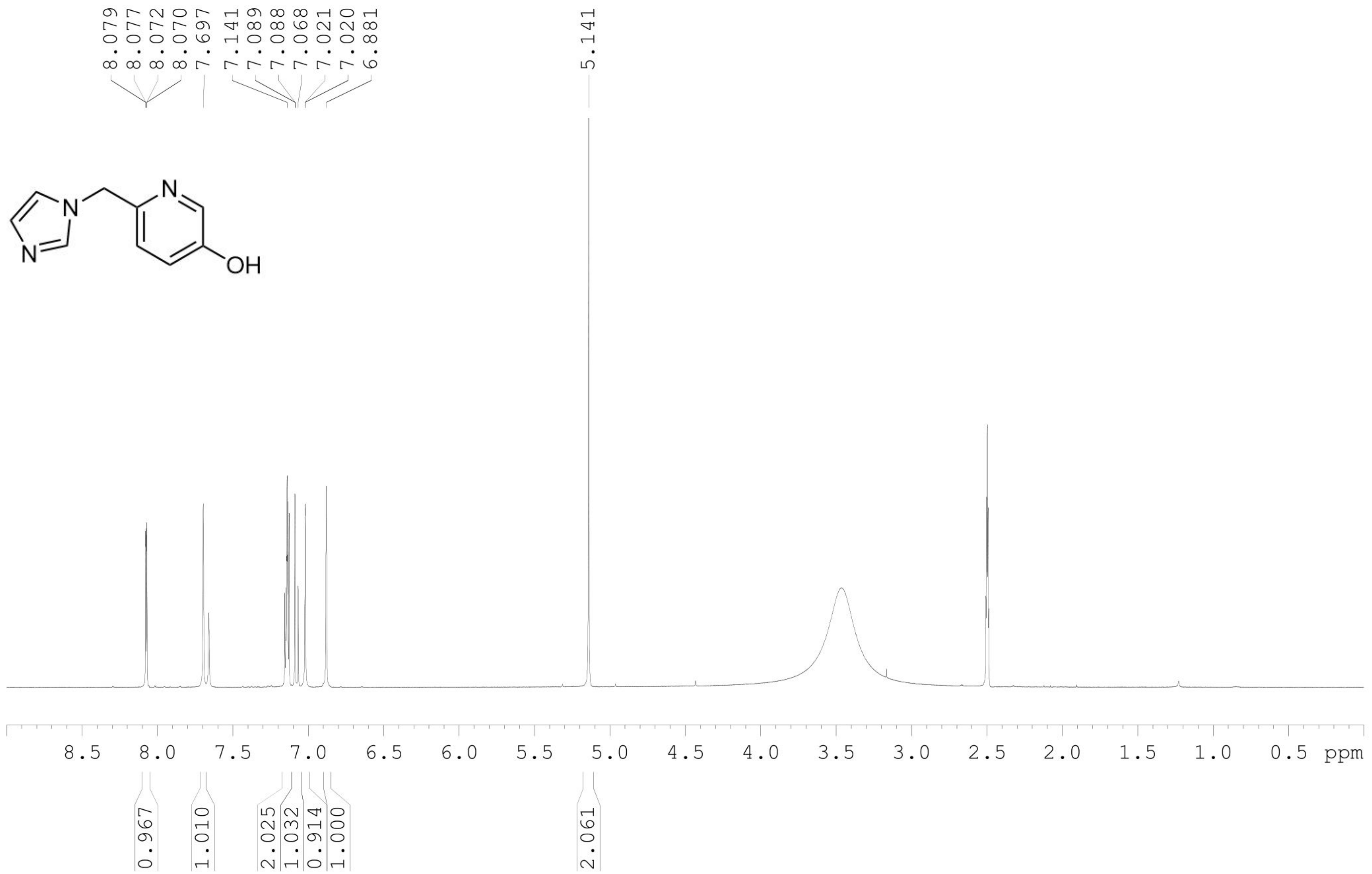

^{13}C NMR (DMSO, 100 MHz) of 25

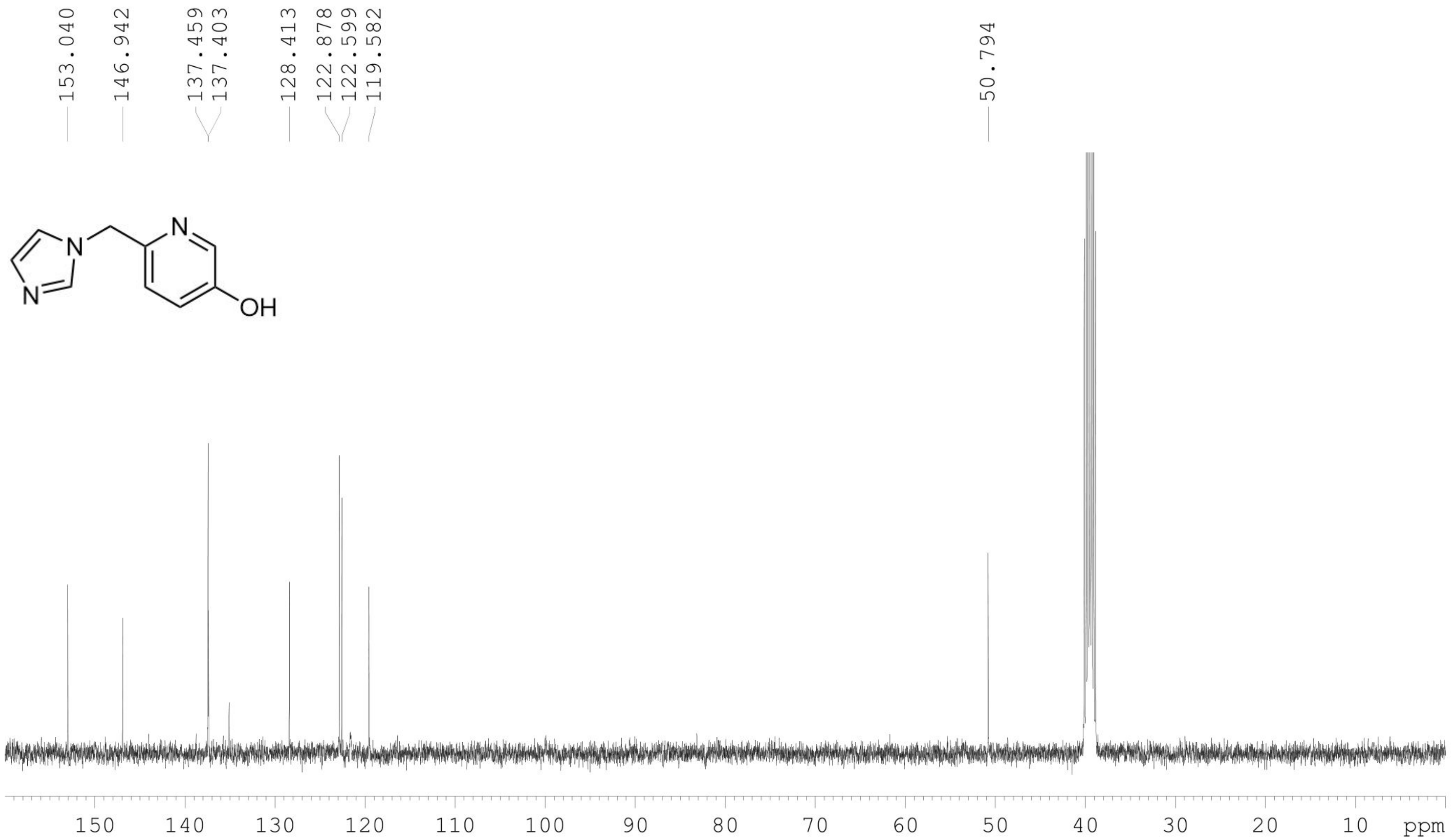

^{1}H NMR ($CDCl_3$, 400 MHz) of 26

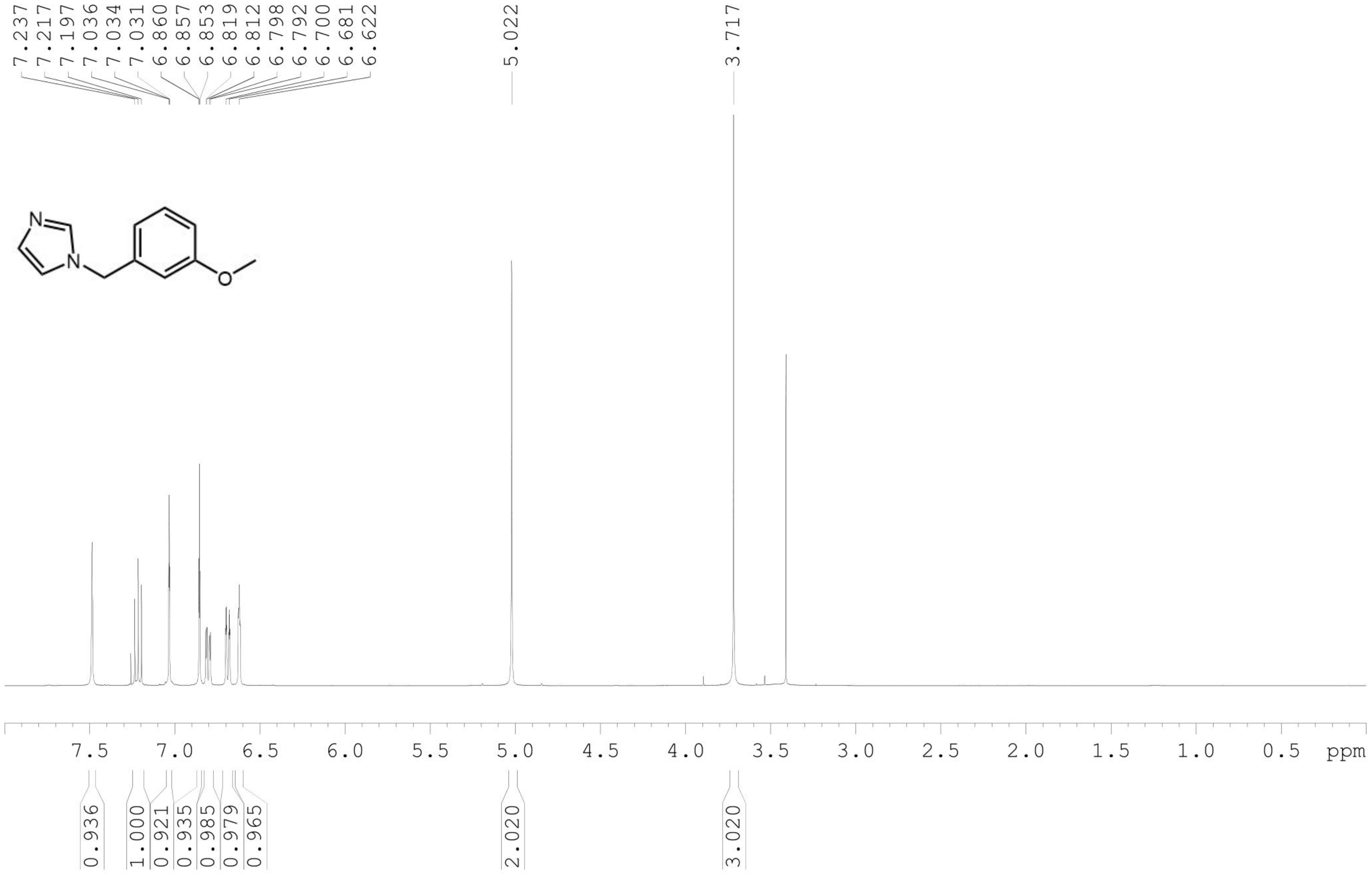

^{13}C NMR ($CDCl_3$, 100 MHz) of 26

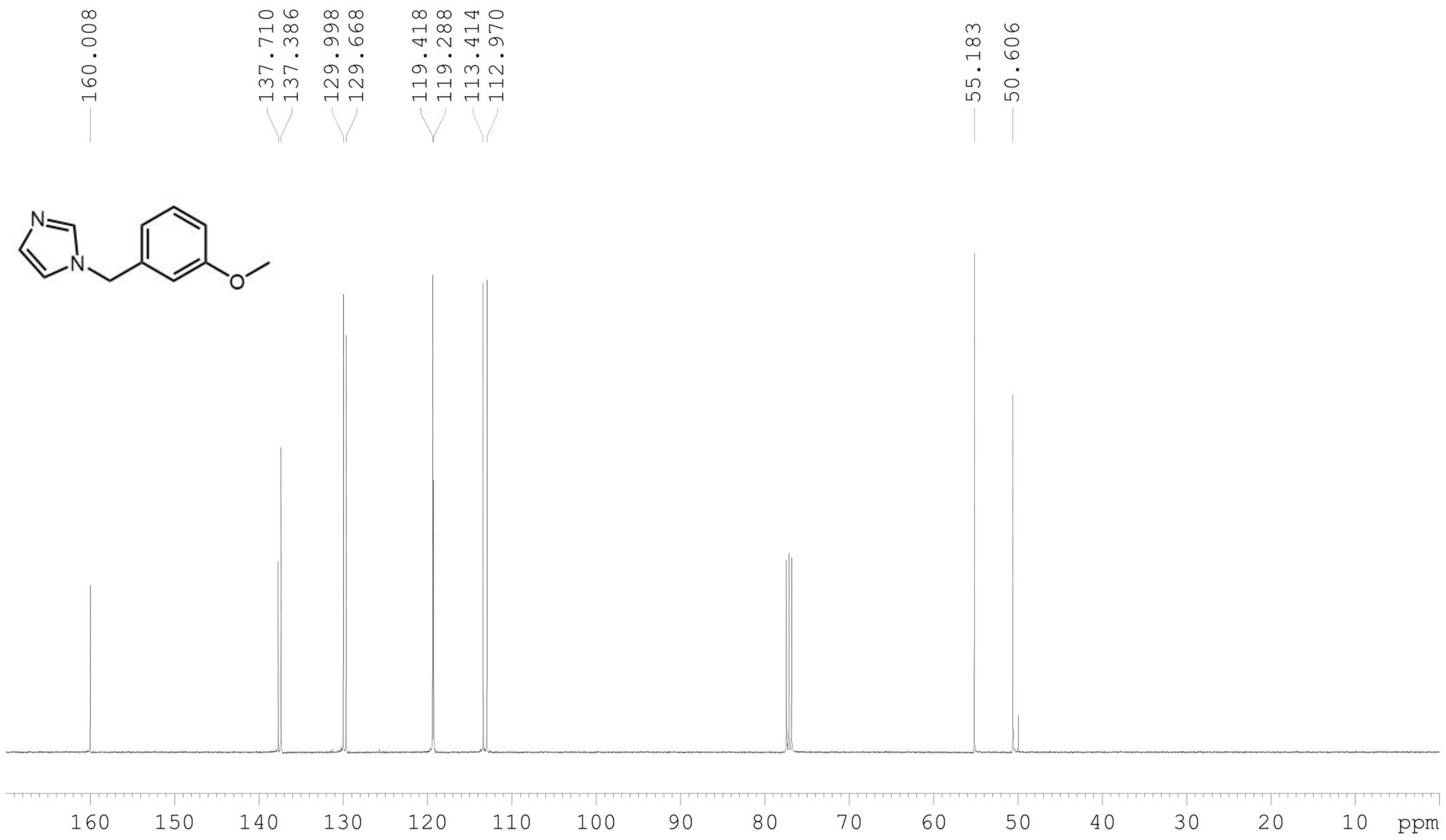

^{1}H NMR (DMSO, 400 MHz) of 27

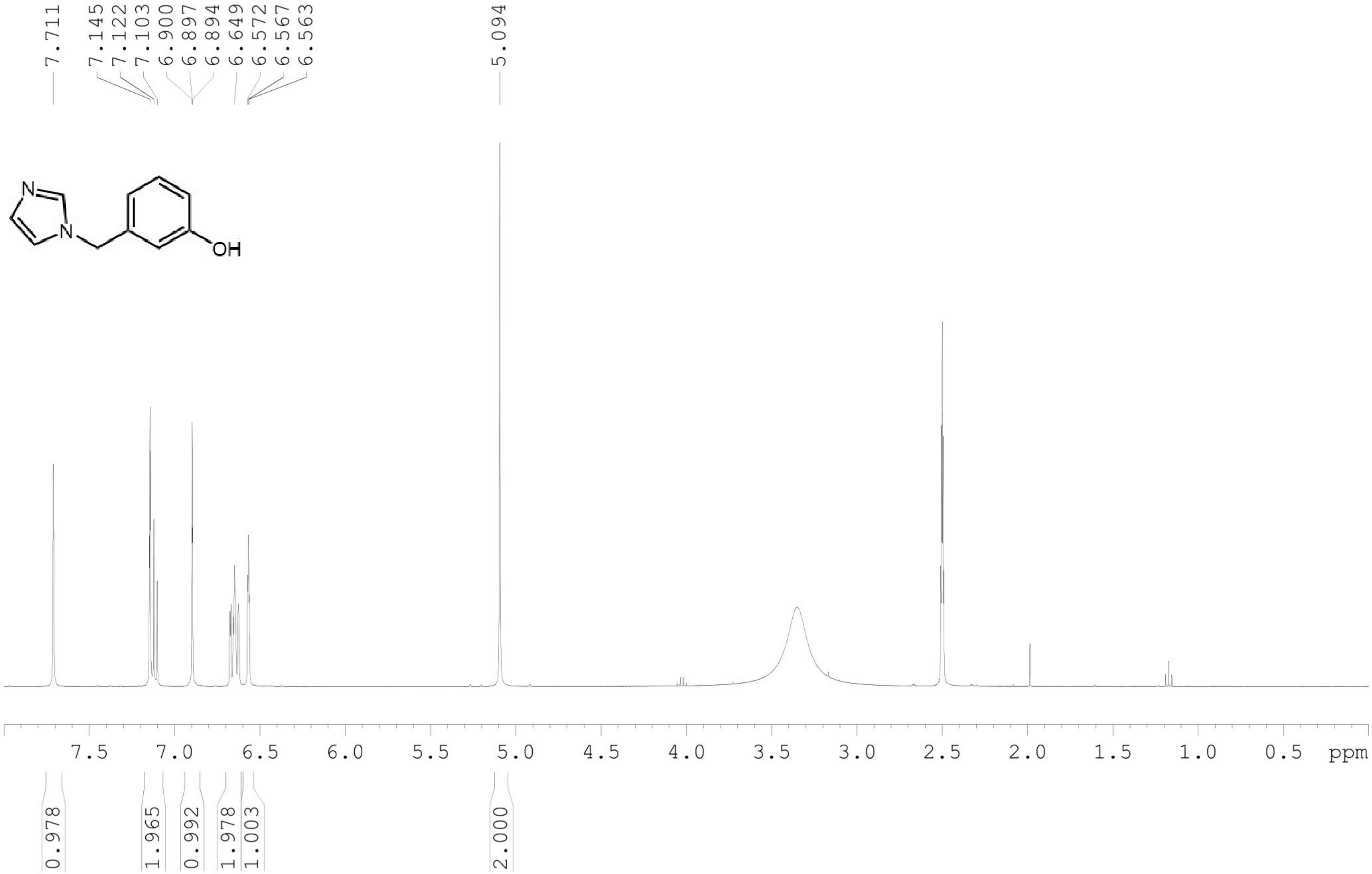

^{13}C NMR (DMSO, 100 MHz) of 27

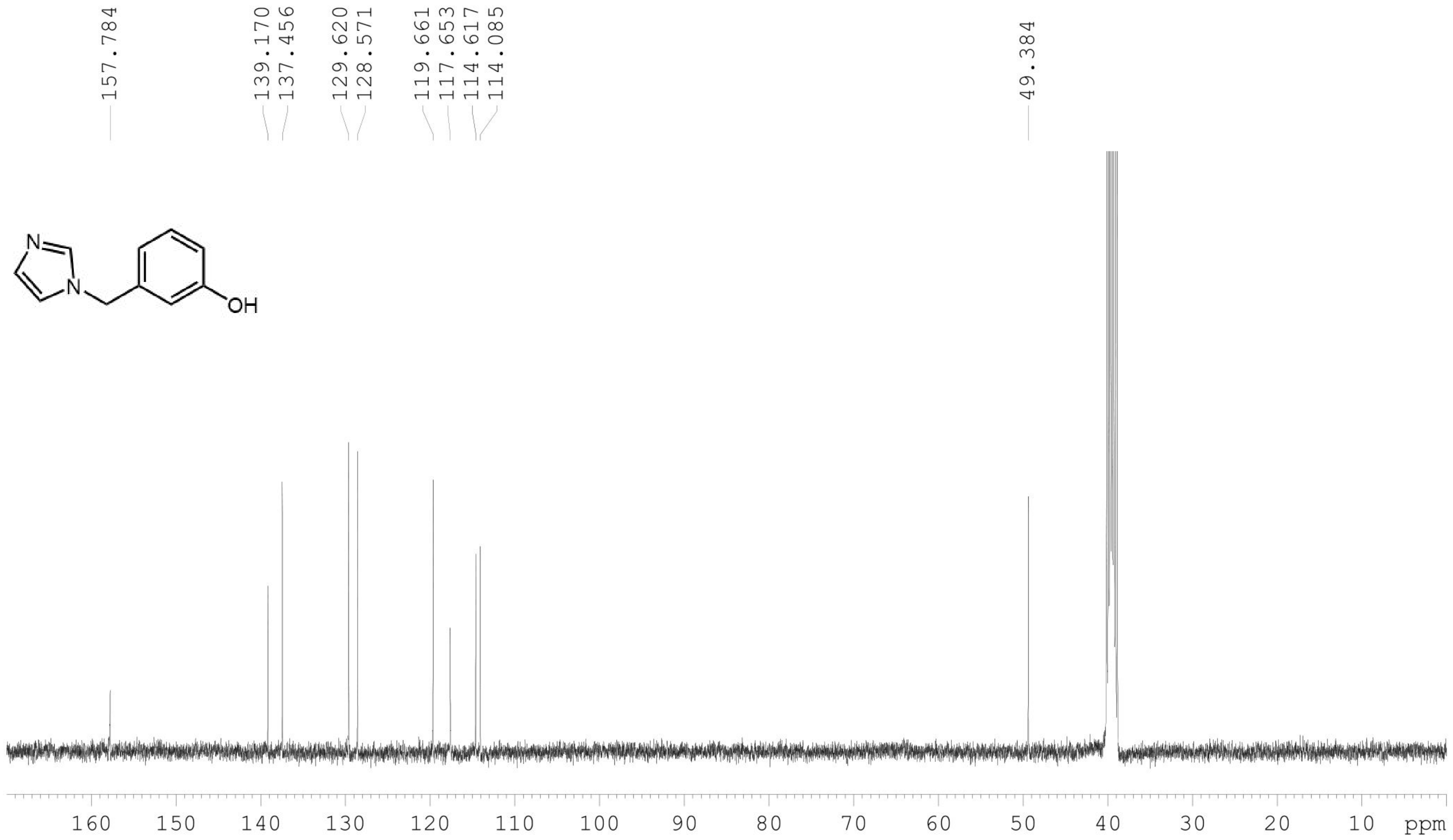

^{1}H NMR ($CDCl_3$, 400 MHz) of 29

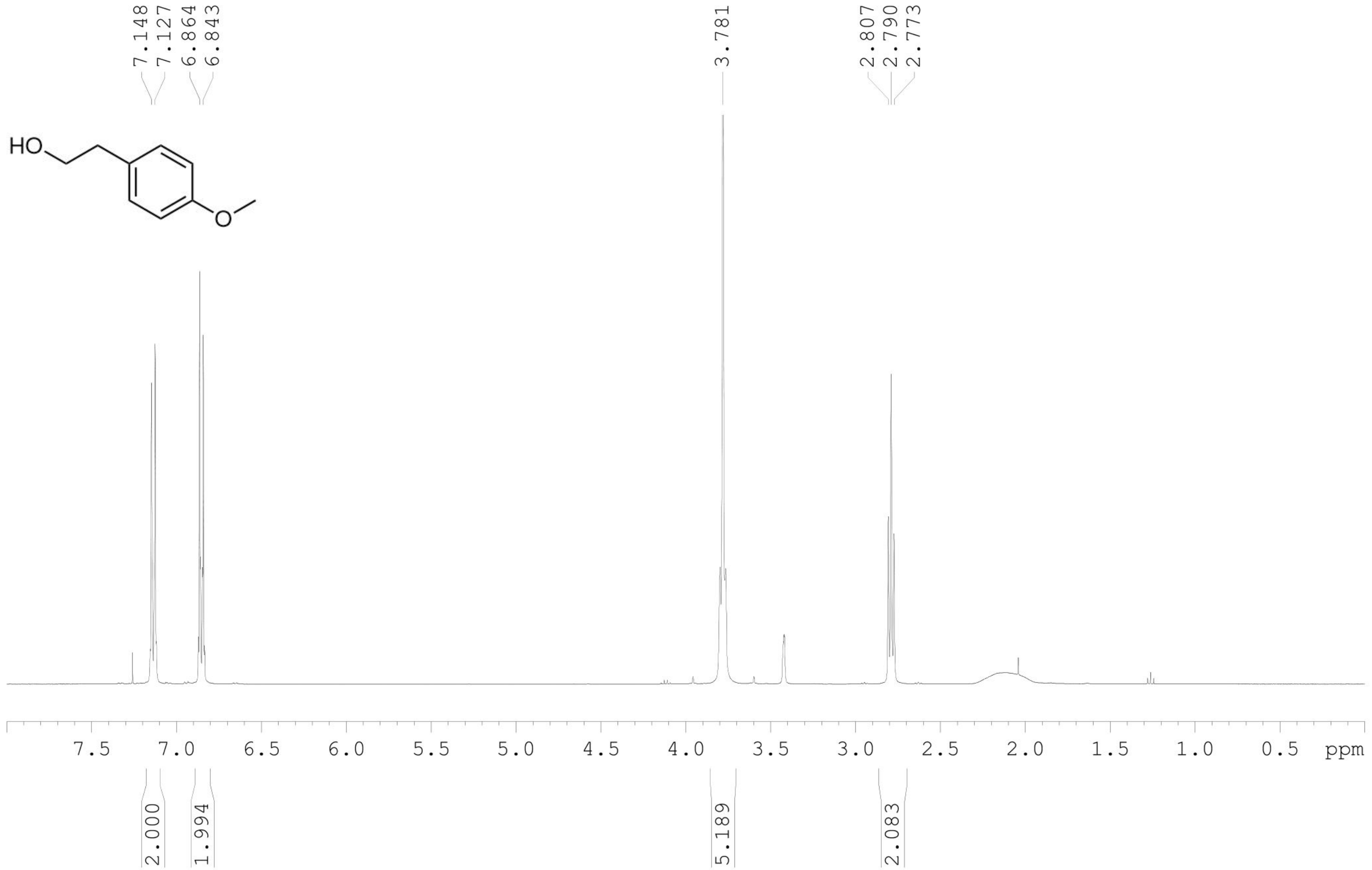

^{13}C NMR ($CDCl_3$, 100 MHz) of 29

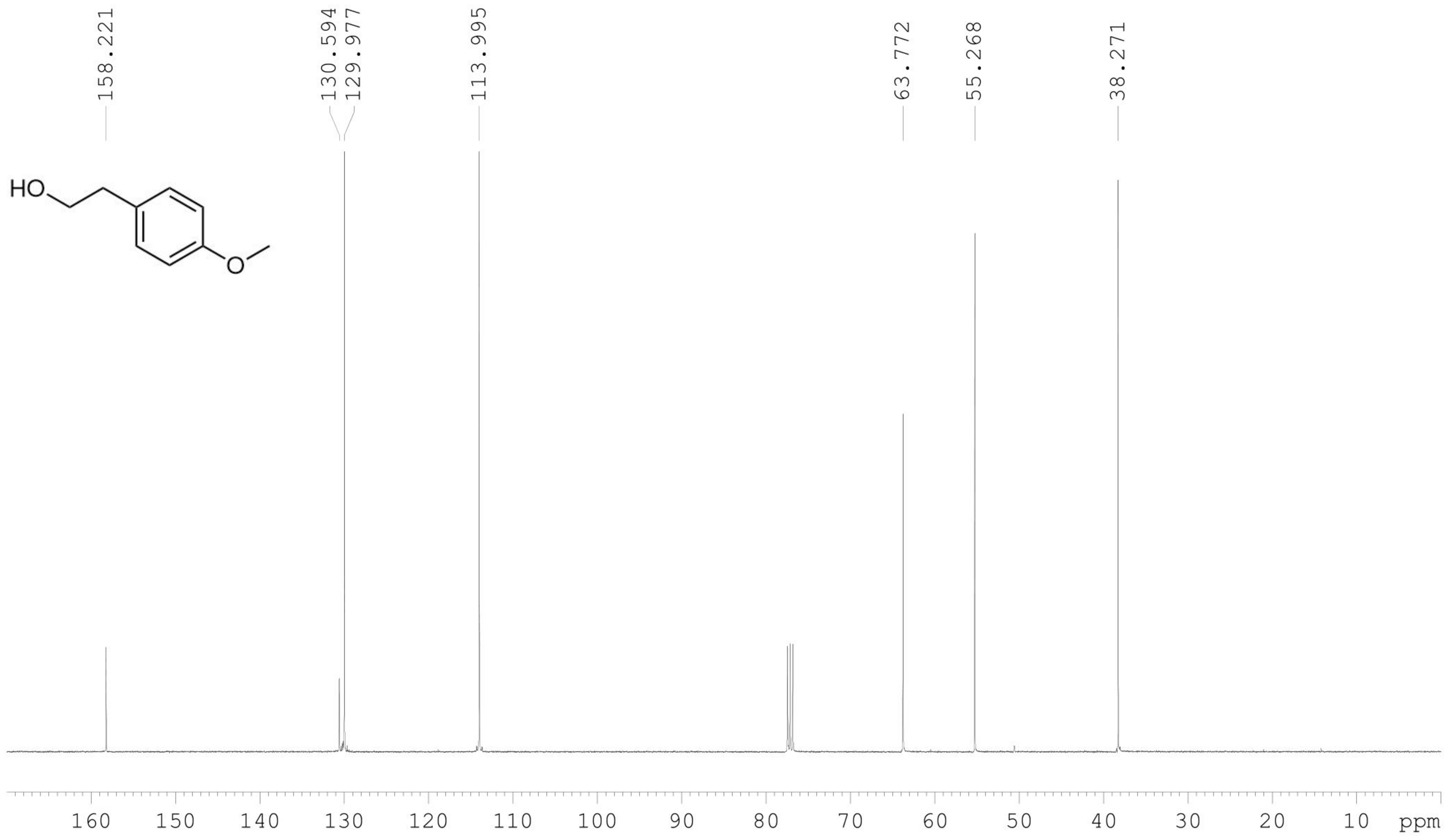

^{1}H NMR ($CDCl_3$, 400 MHz) of 30

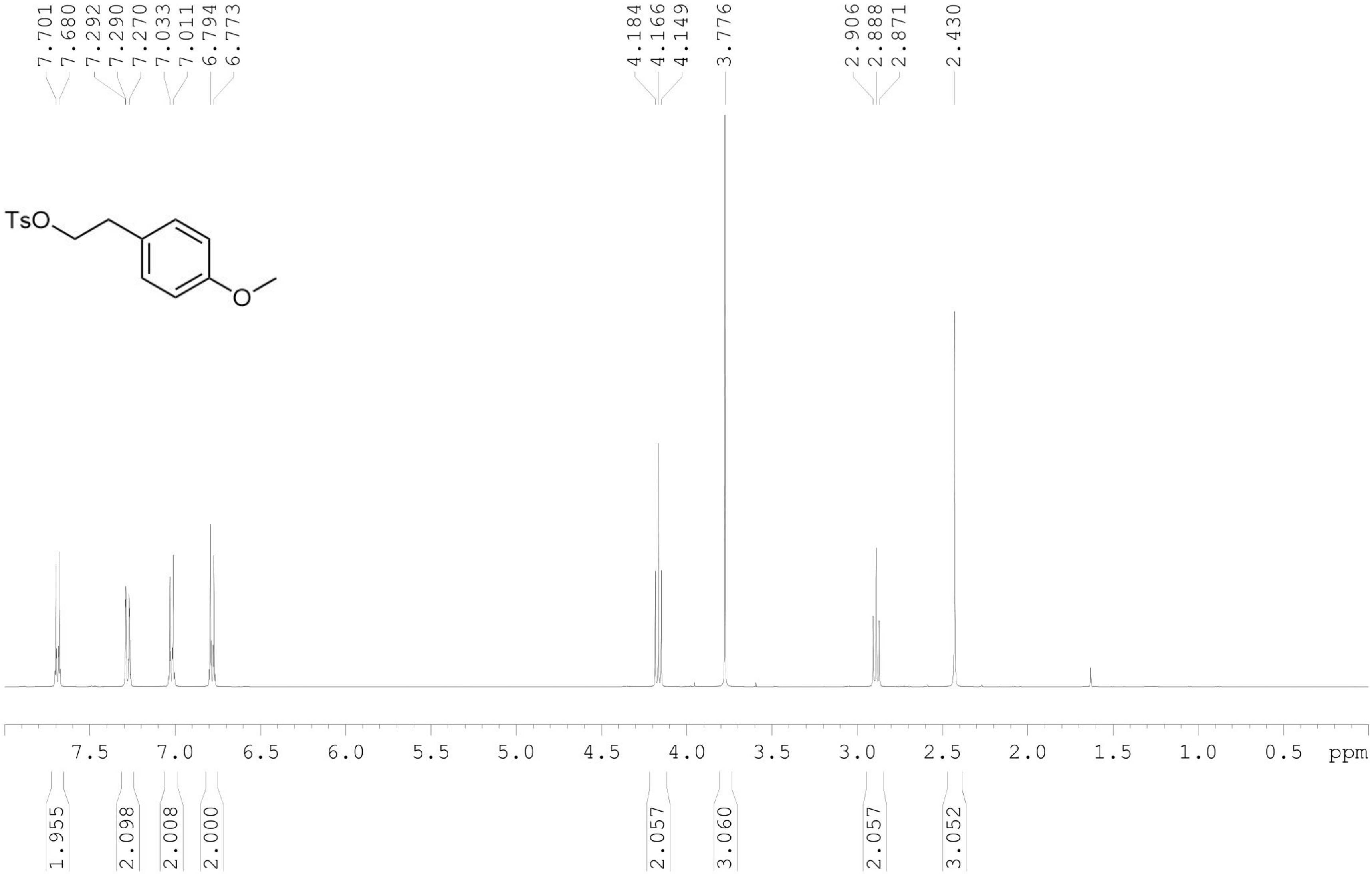

^{13}C NMR ($CDCl_3$, 100 MHz) of 30

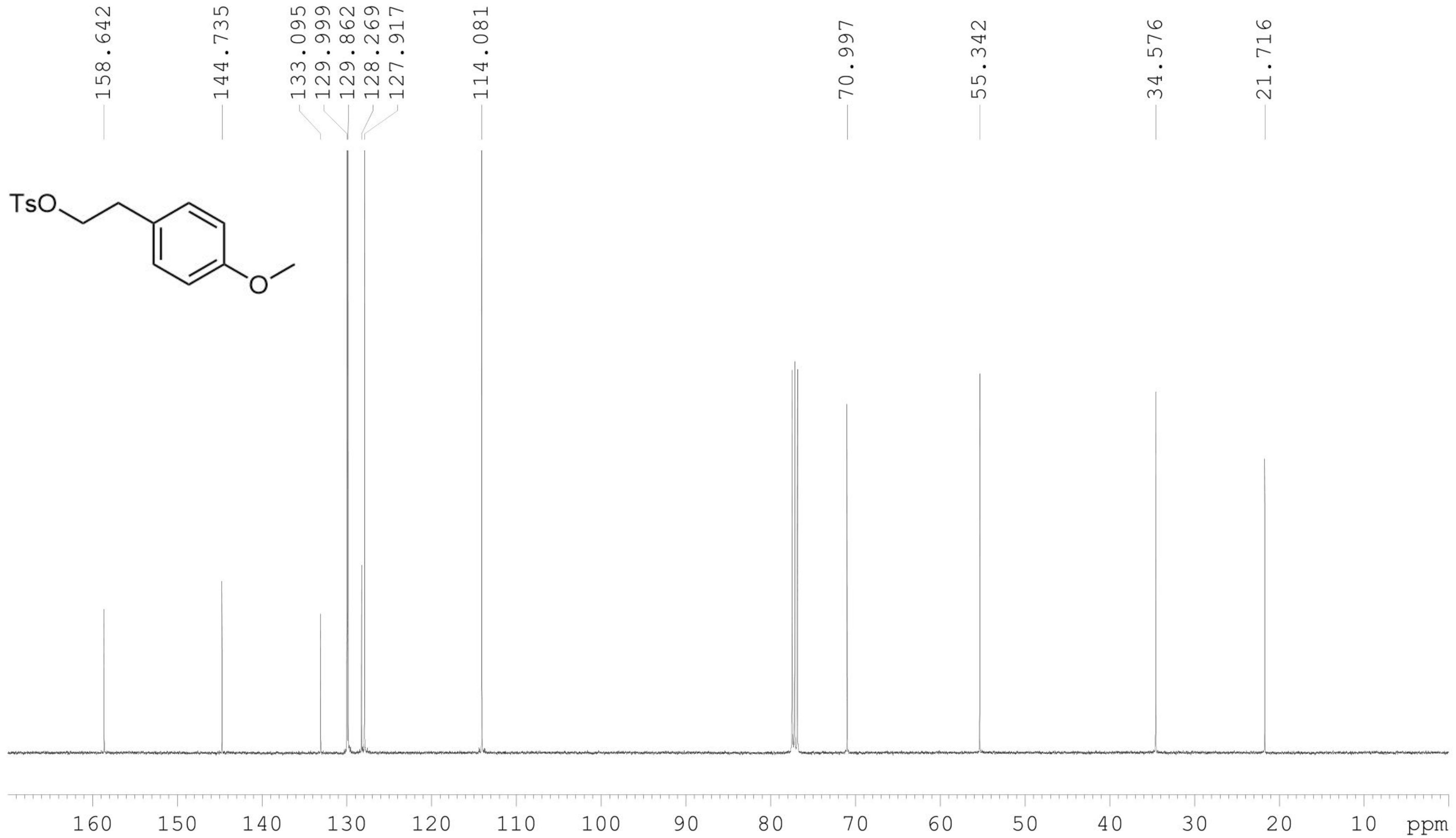

^{1}H NMR ($CDCl_3$, 400 MHz) of 31

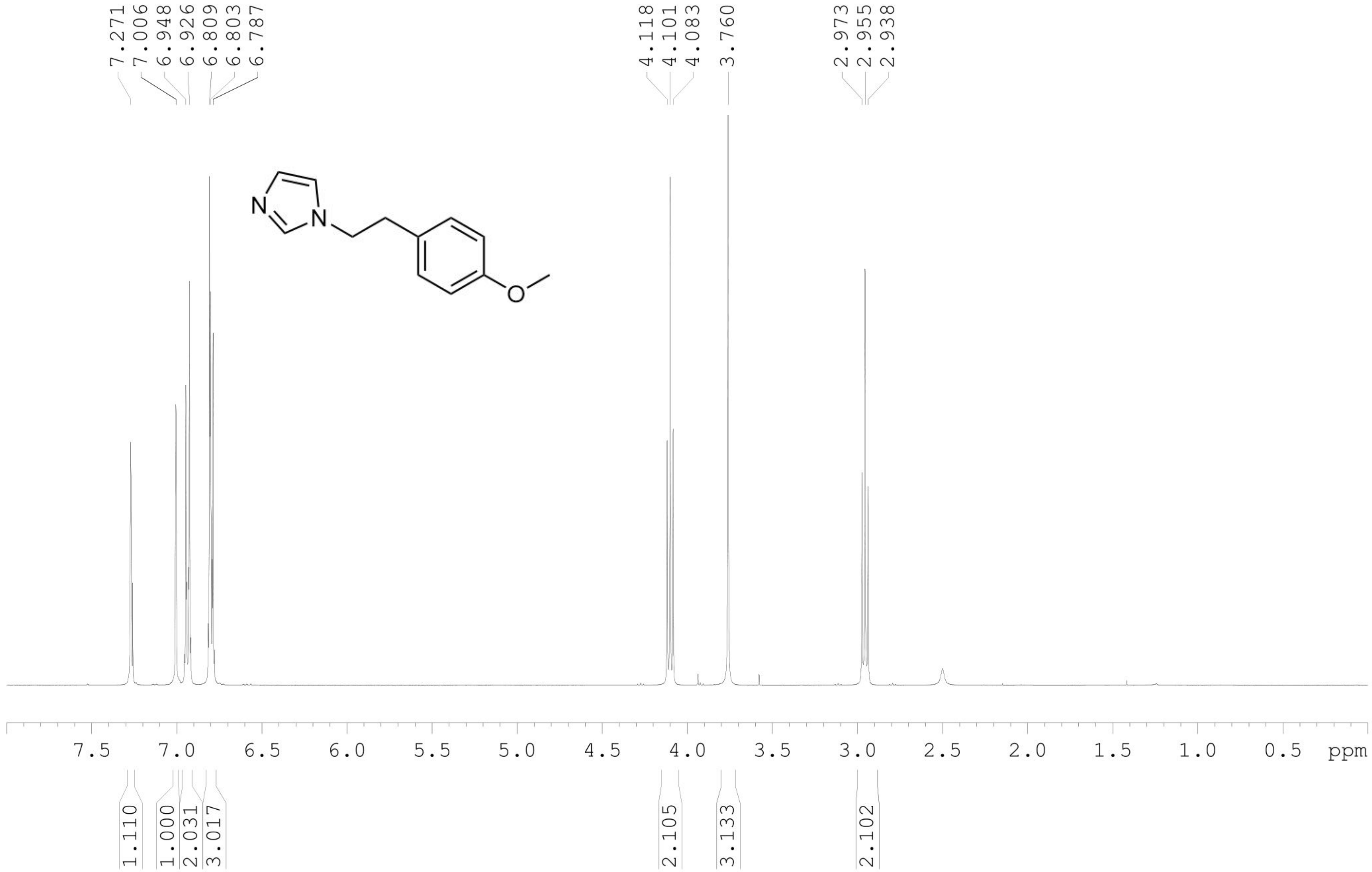

^{13}C NMR ($CDCl_3$, 100 MHz) of 31

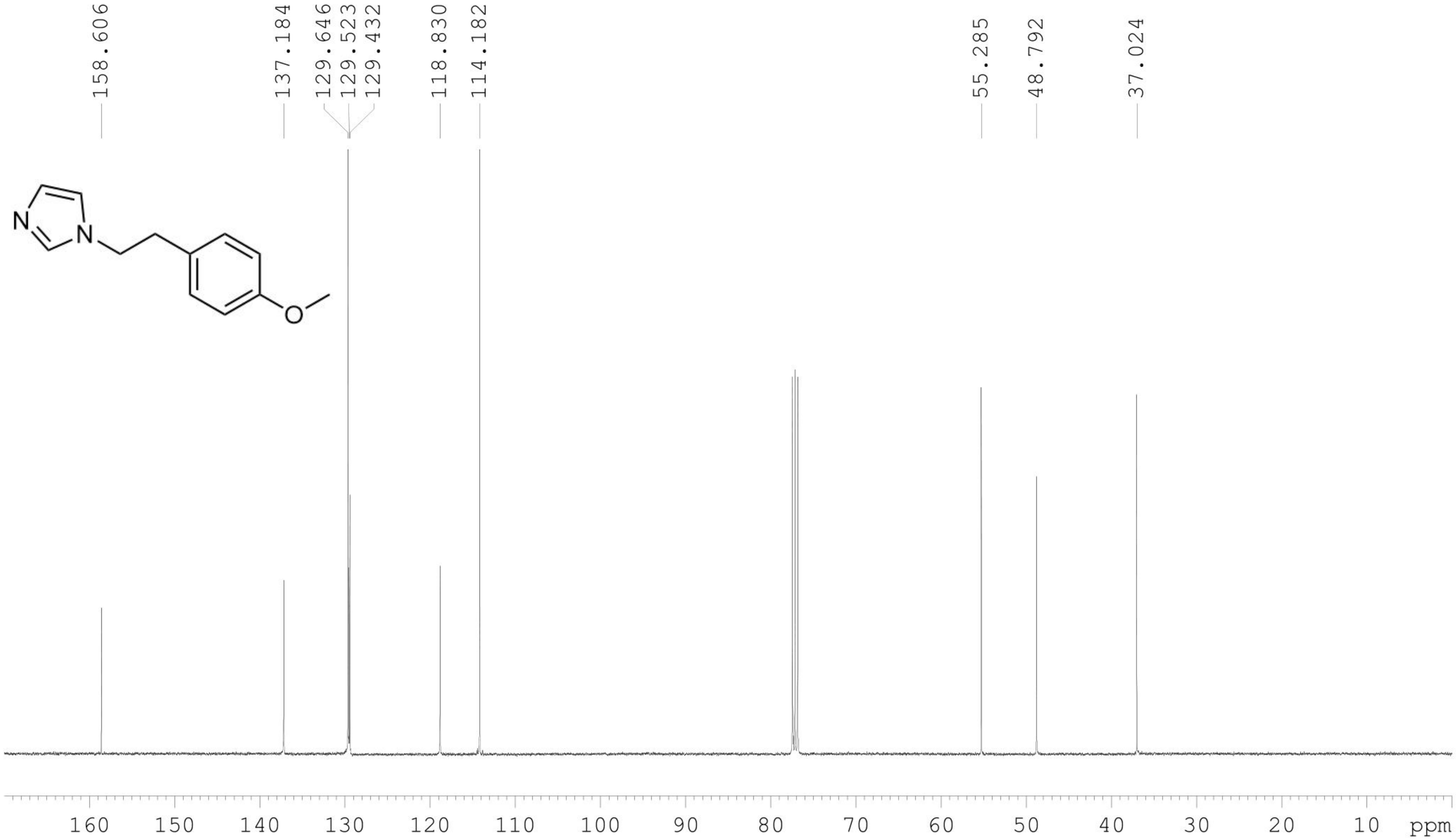

^{1}H NMR (DMSO, 400 MHz) of 32

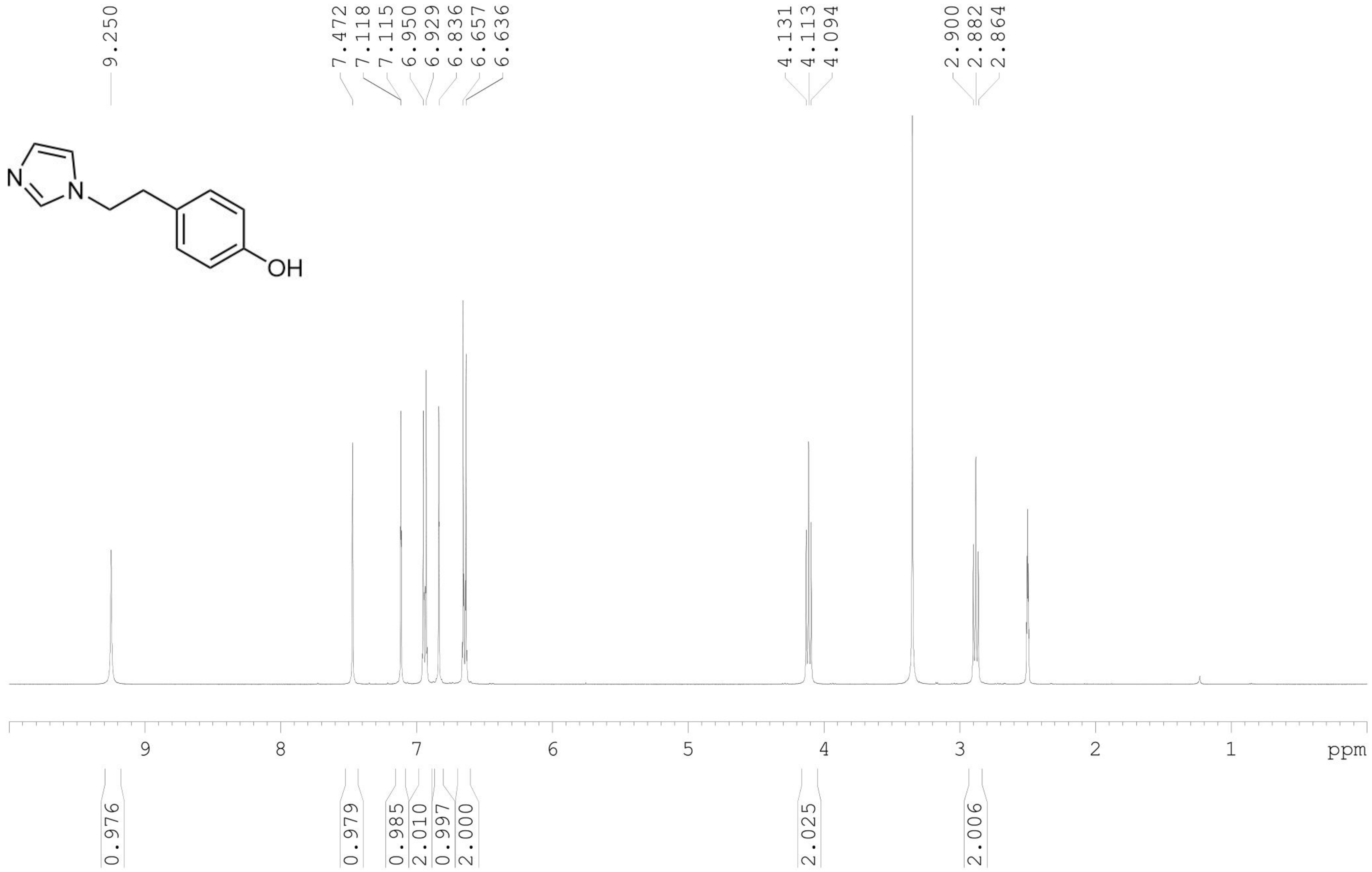

^{13}C NMR (DMSO, 100 MHz) of 32

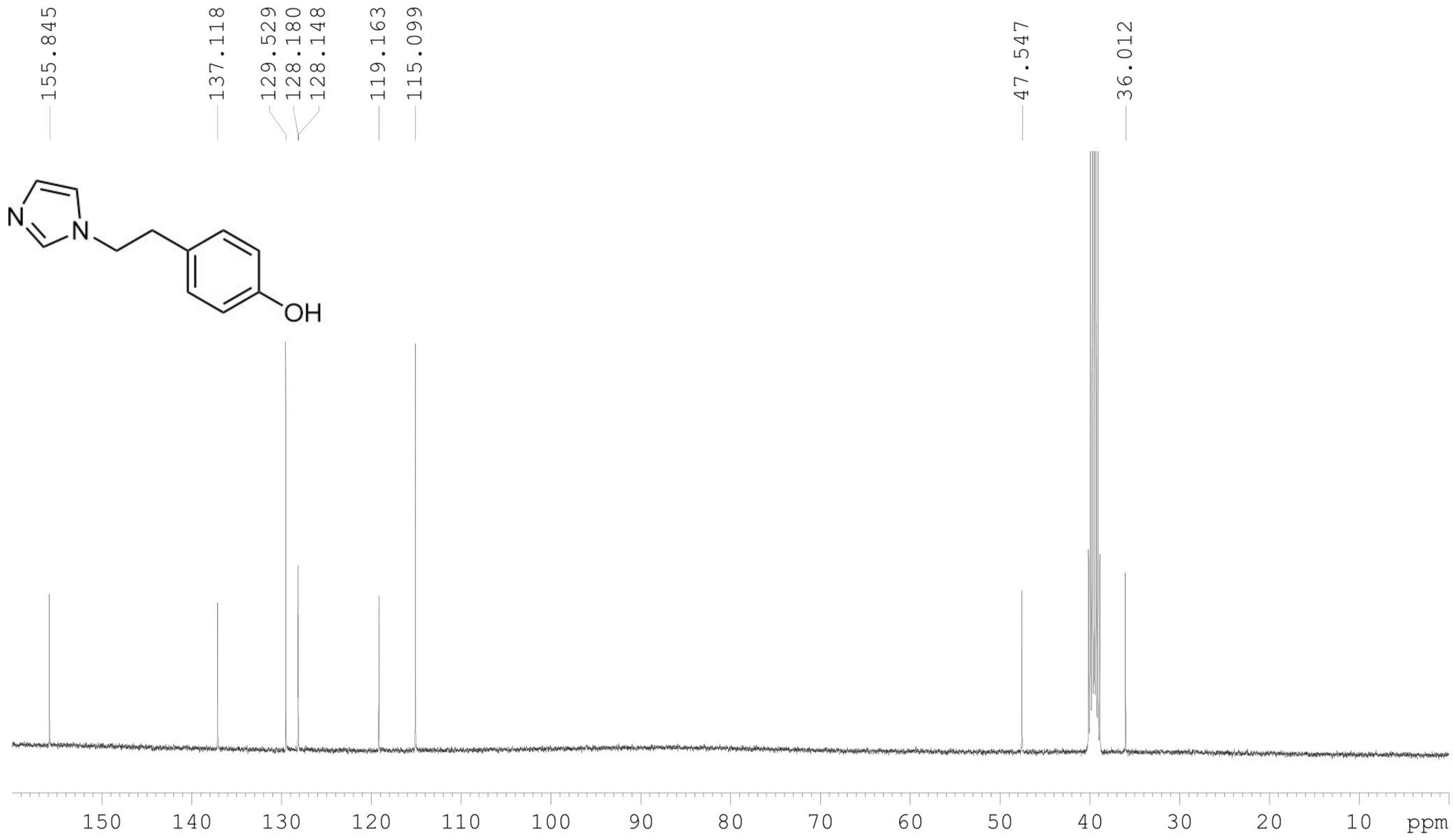

^{1}H NMR ($CDCl_3$, 400 MHz) of 33

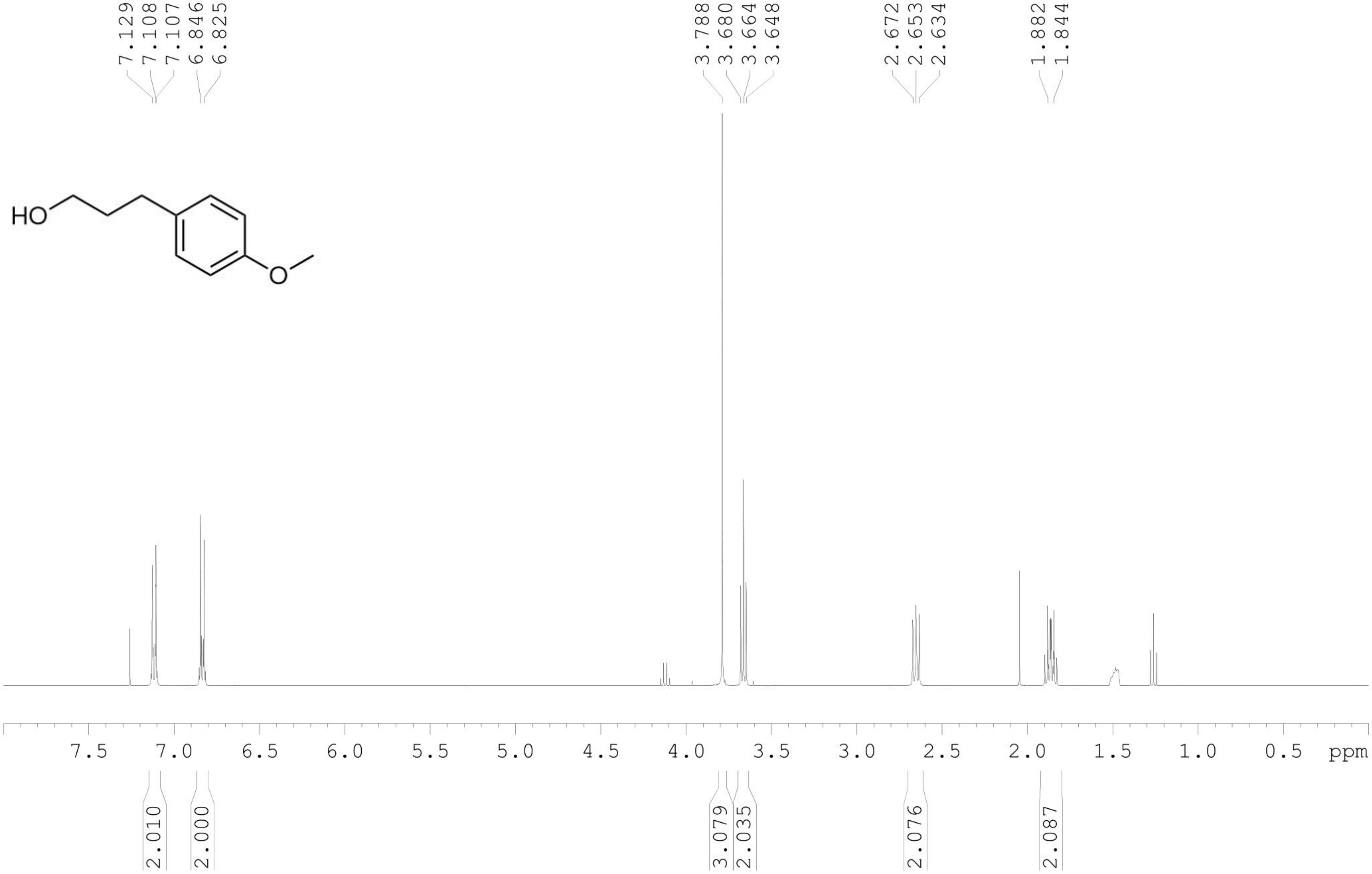

^{13}C NMR ($CDCl_3$, 100 MHz) of 33

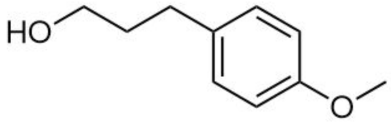

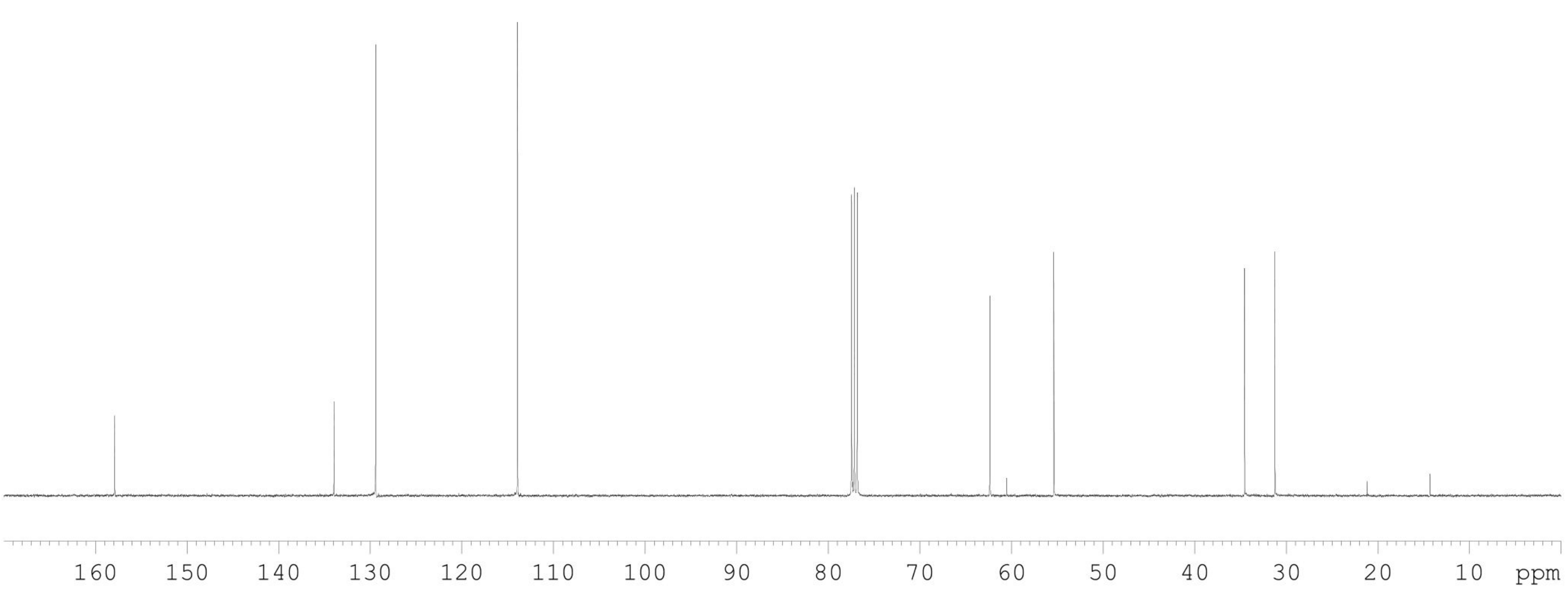

^{1}H NMR ($CDCl_3$, 400 MHz) of 34

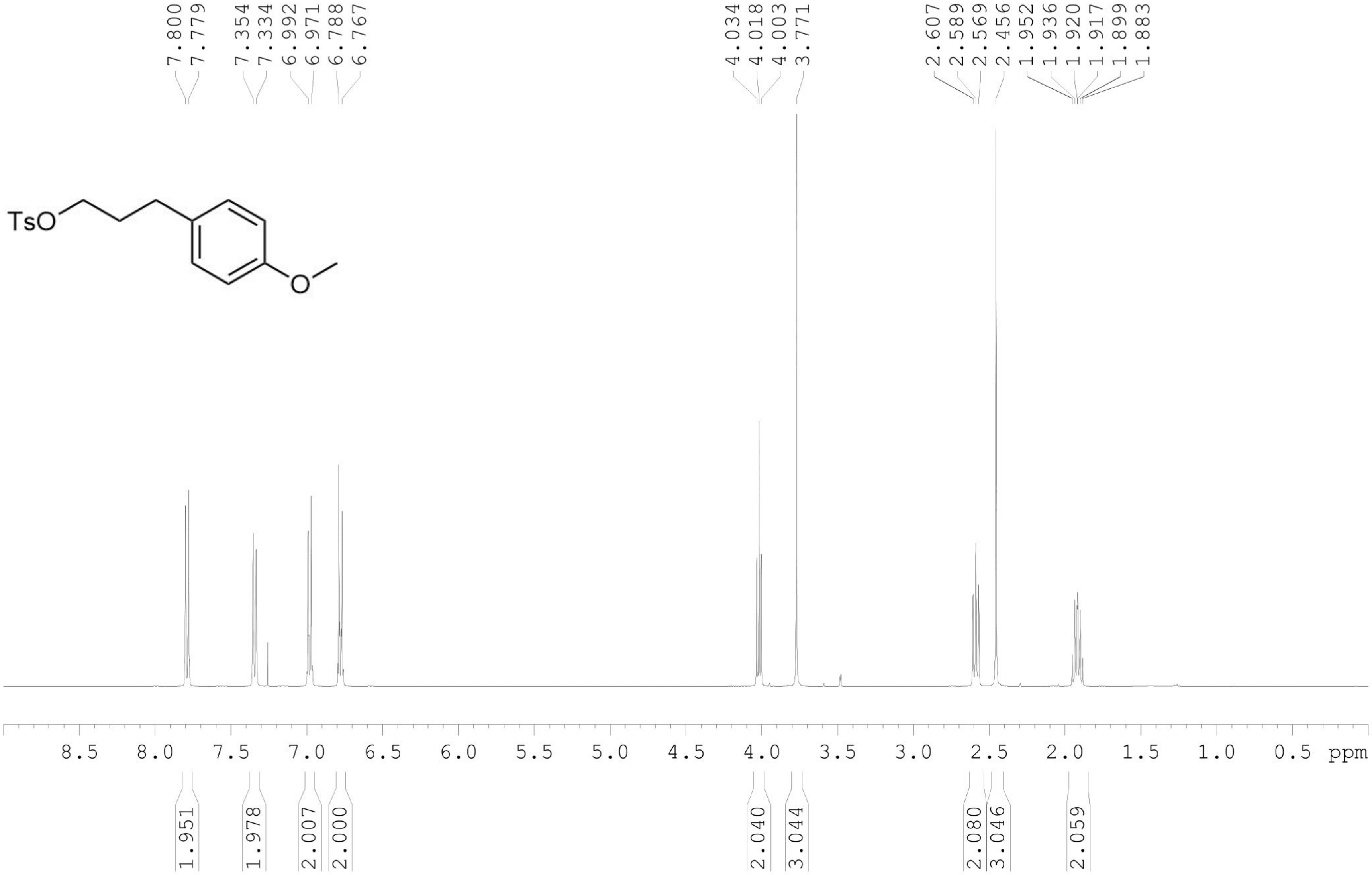

^{13}C NMR ($CDCl_3$, 100 MHz) of 34

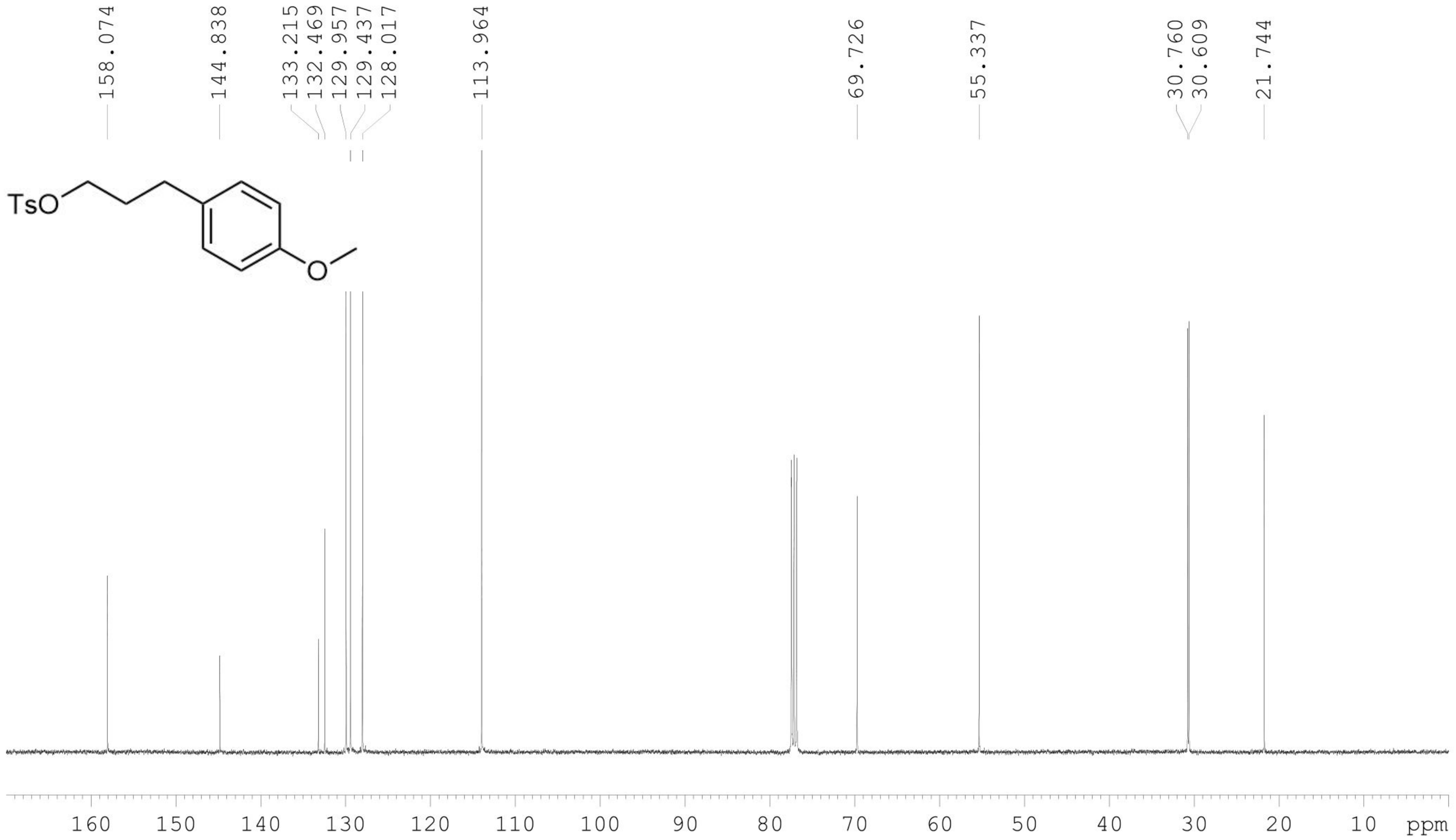

^{1}H NMR ($CDCl_3$, 400 MHz) of 35

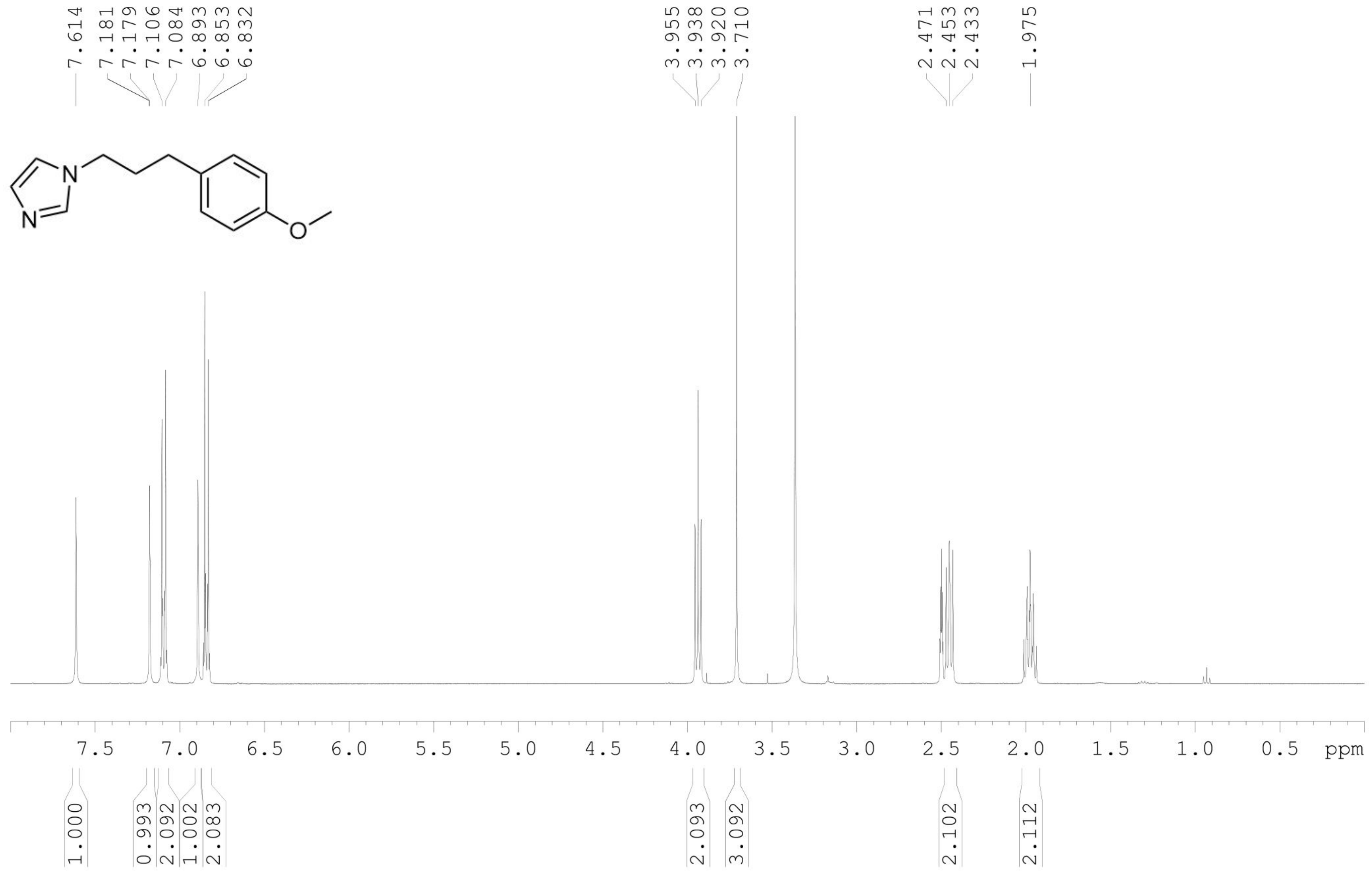

^{13}C NMR ($CDCl_3$, 100 MHz) of 35

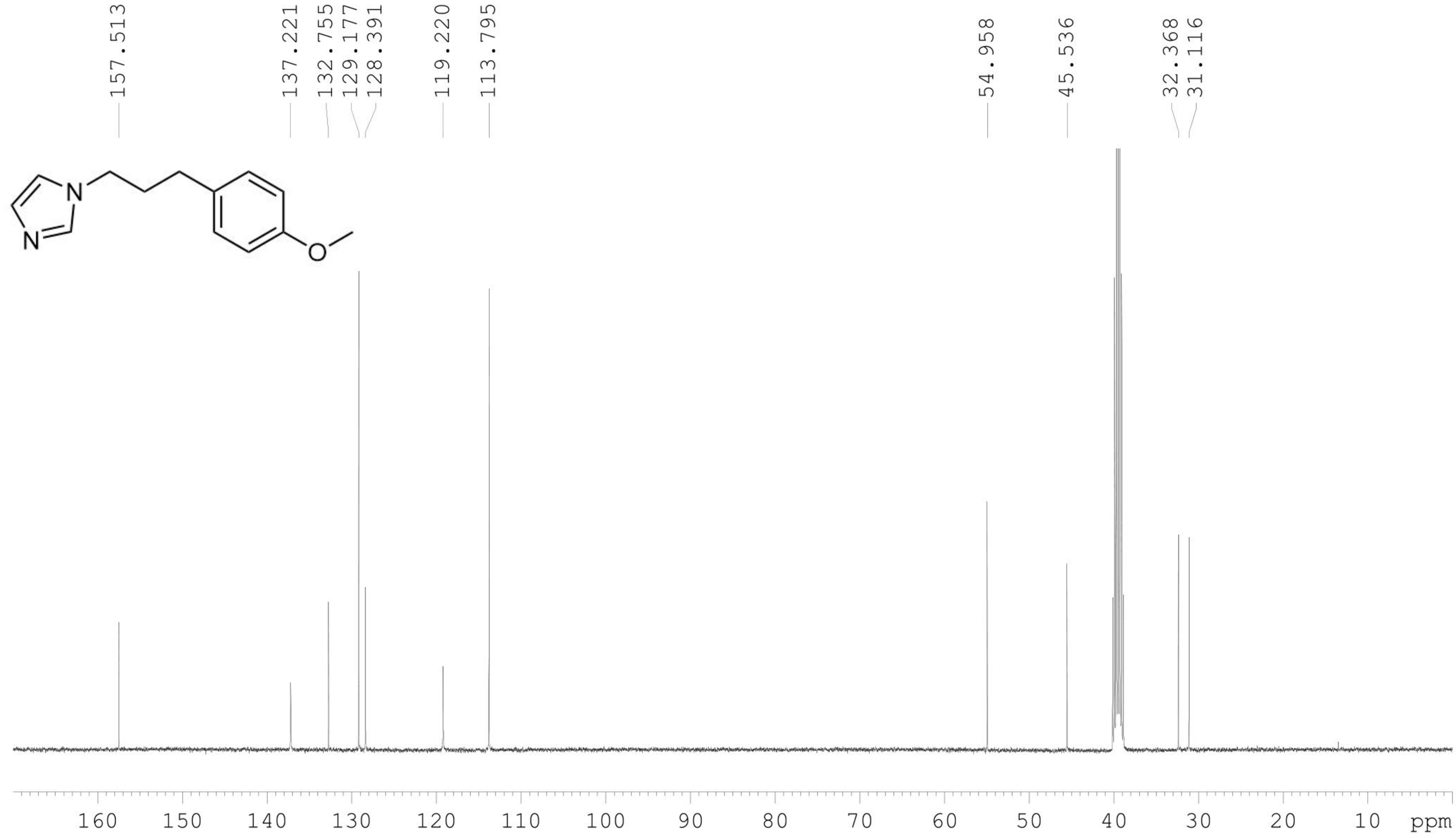

^{1}H NMR (DMSO, 400 MHz) of 36

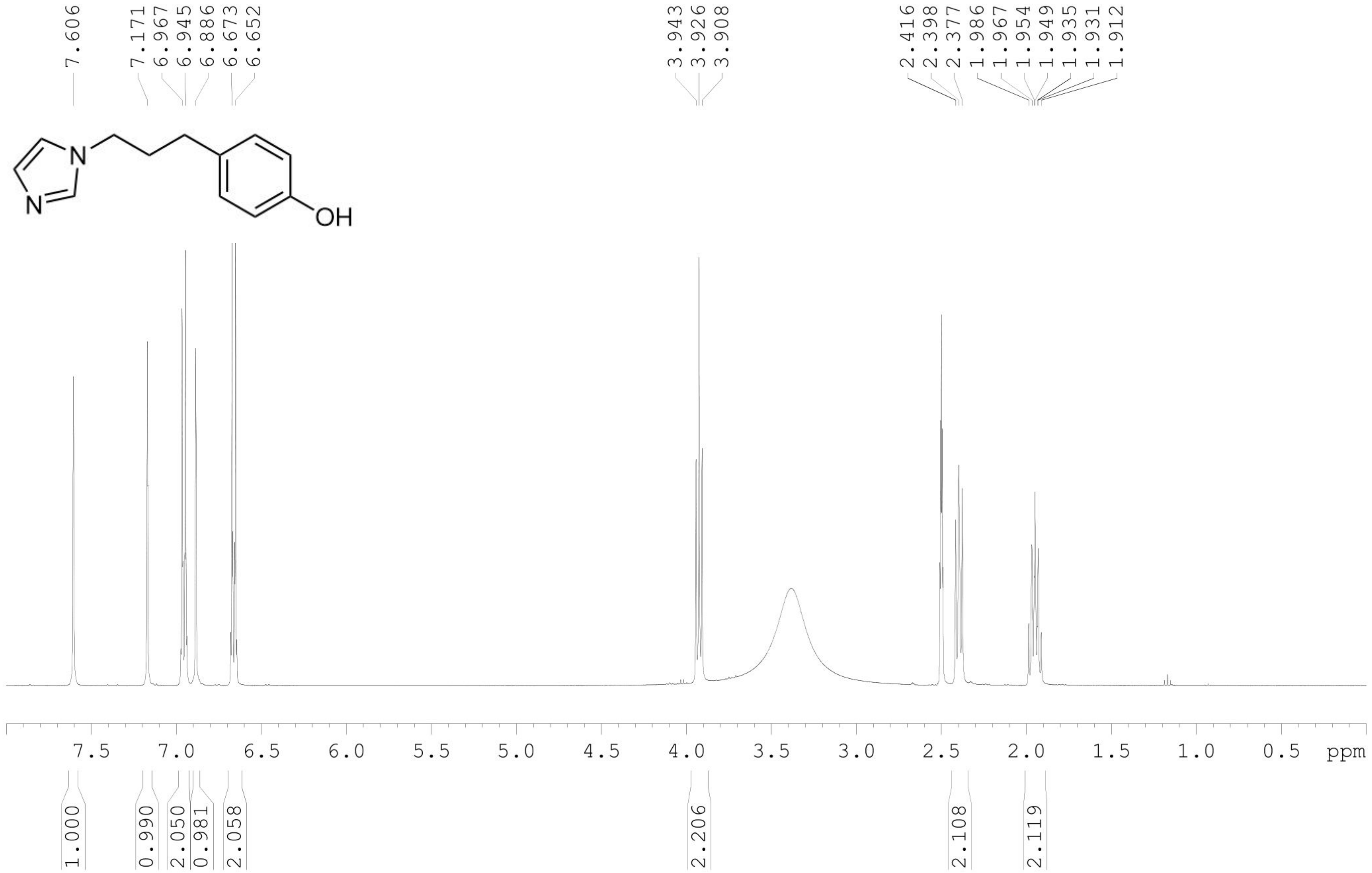

^{13}C NMR (DMSO, 100 MHz) of 36

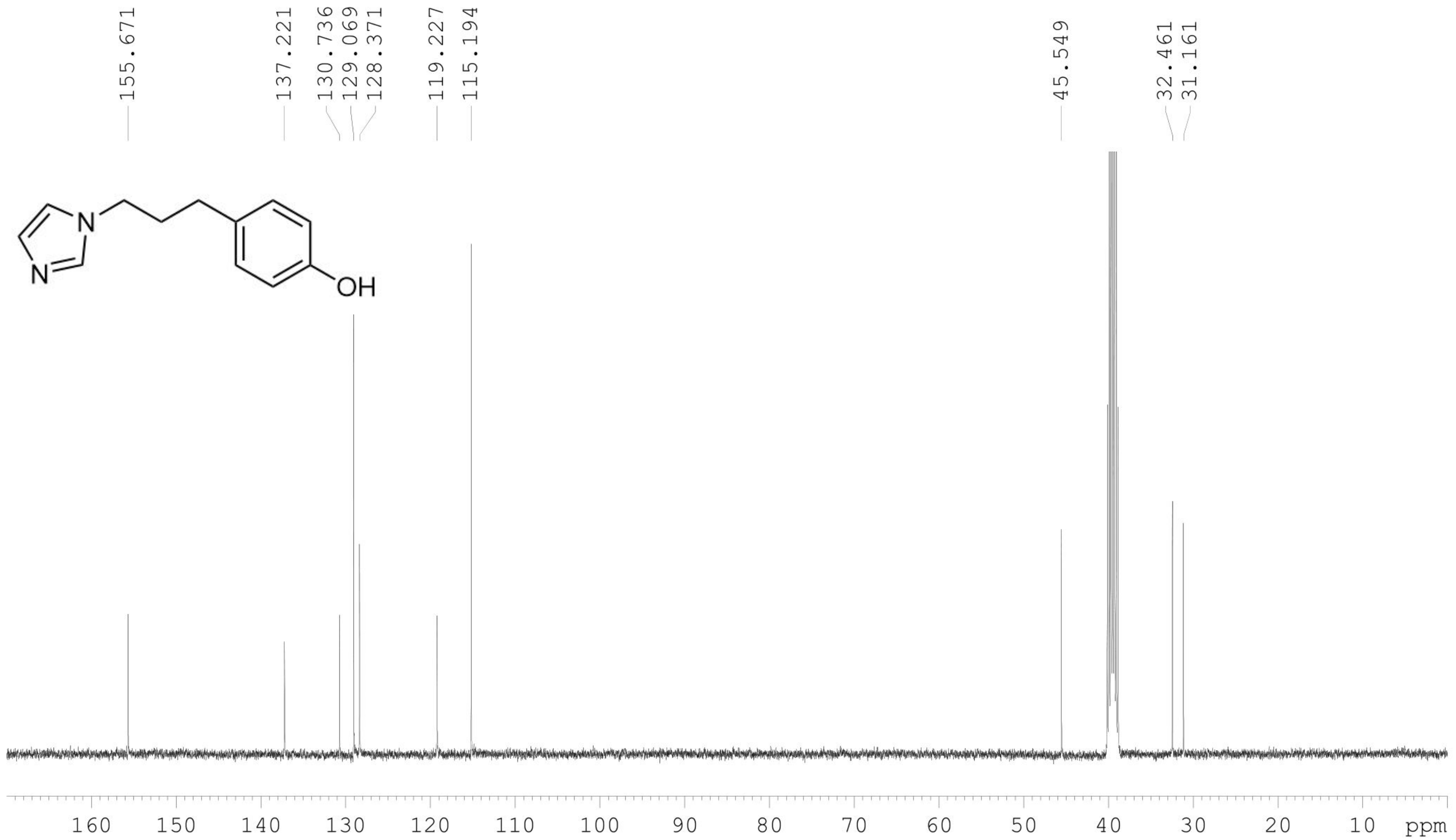

^{1}H NMR ($CDCl_3$, 400 MHz) of 37

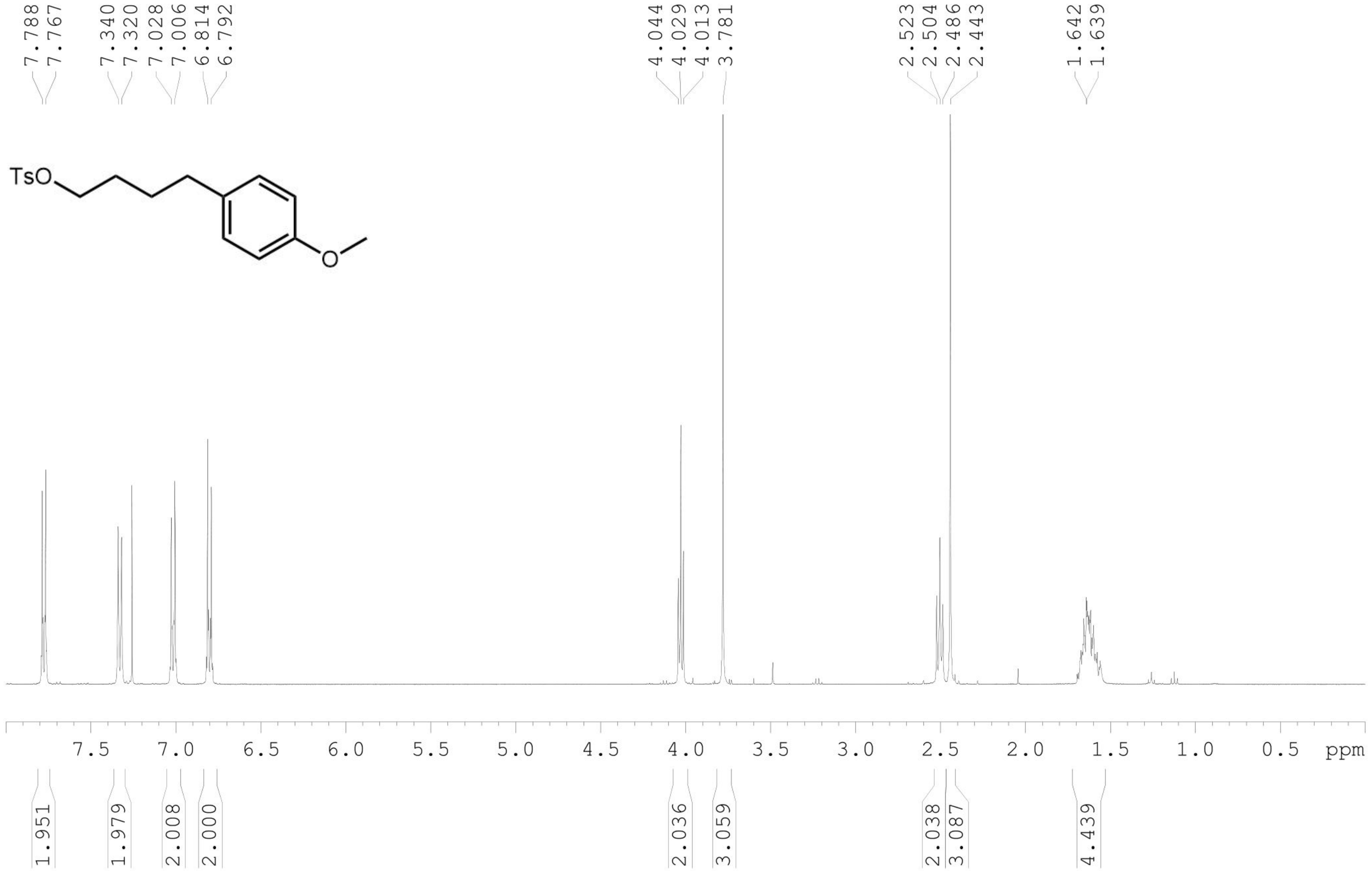

^{13}C NMR ($CDCl_3$, 100 MHz) of 37

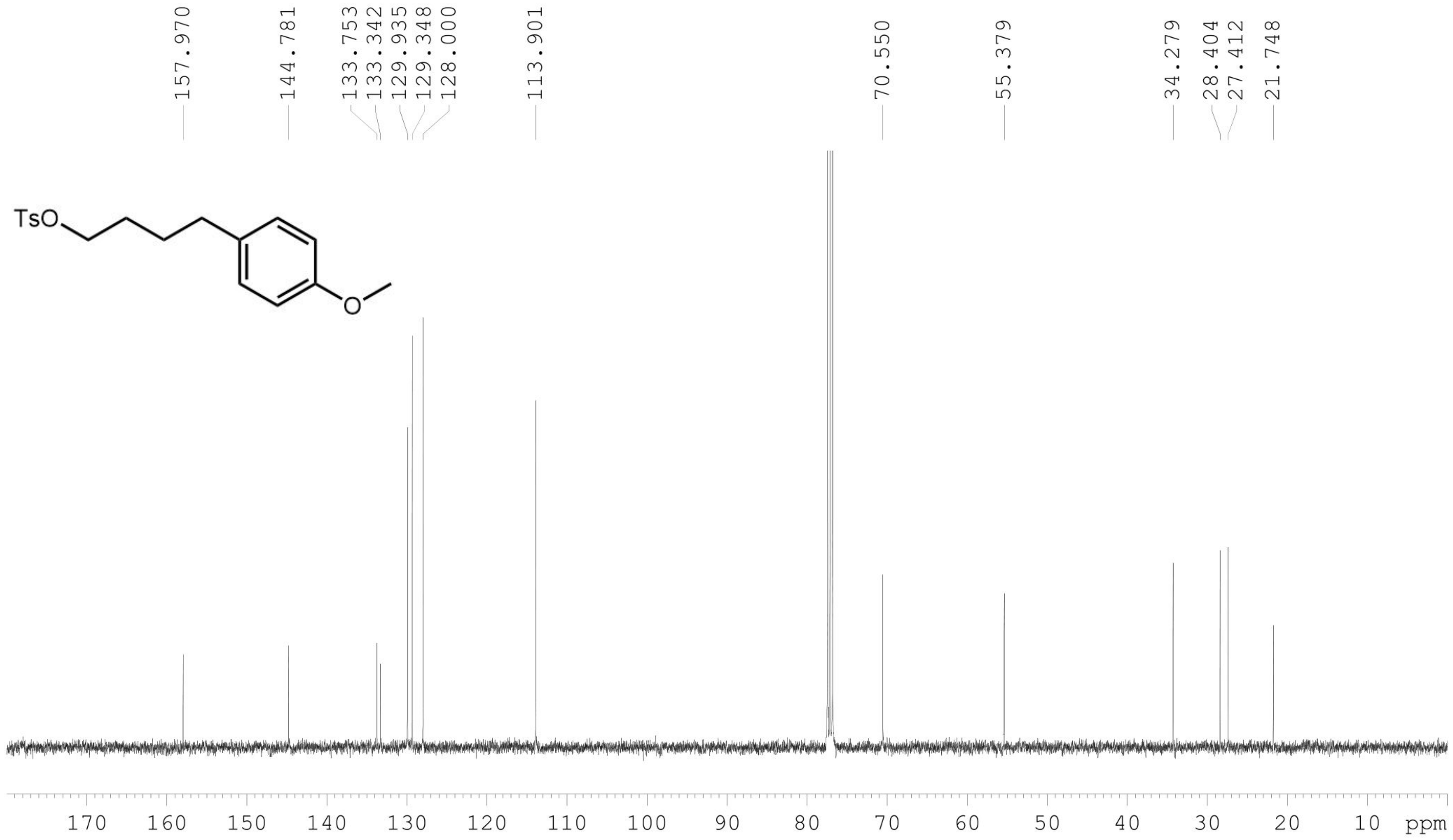

^{1}H NMR ($CDCl_3$, 400 MHz) of 38

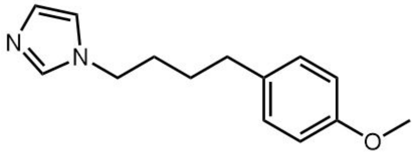

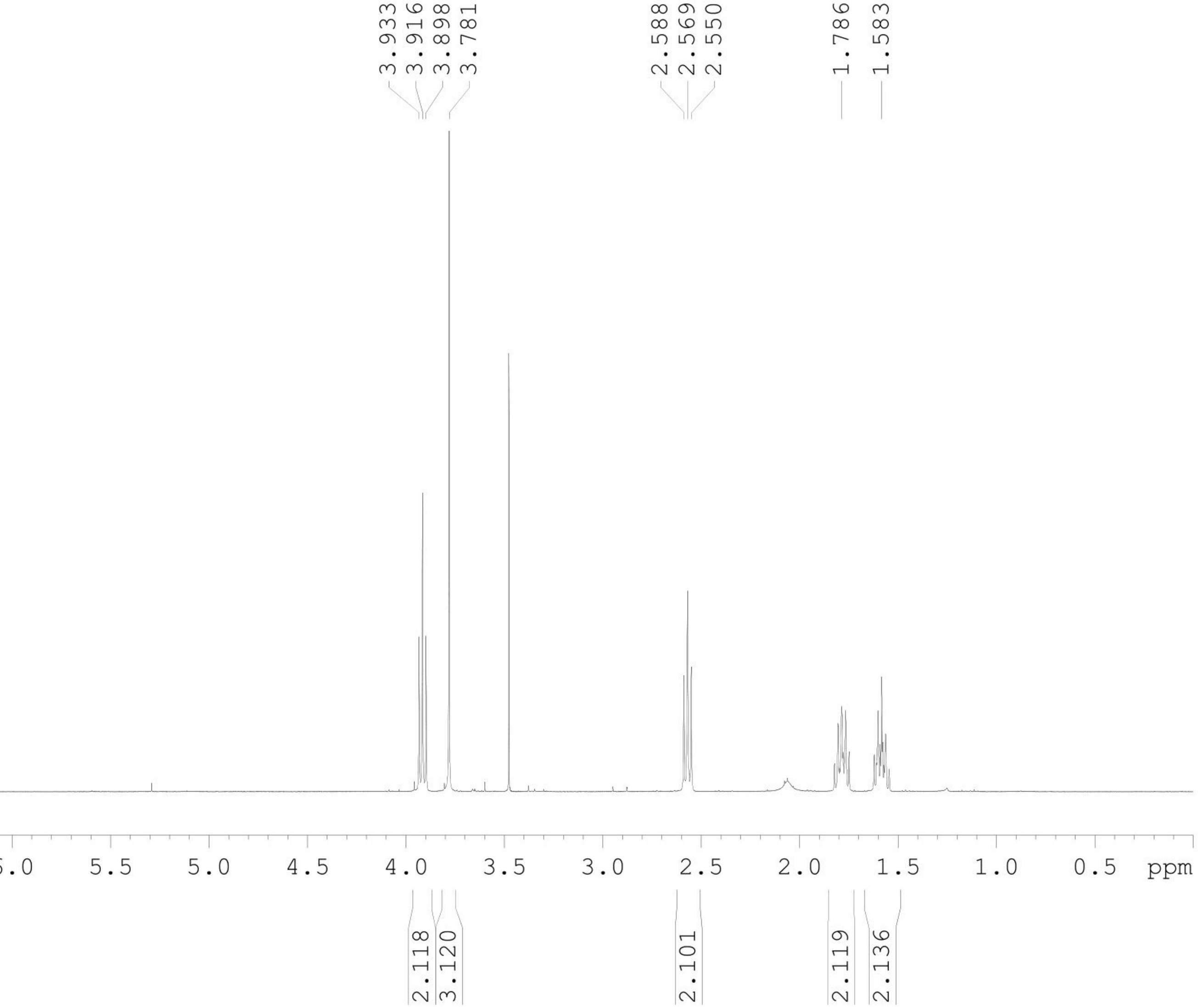

^{13}C NMR ($CDCl_3$, 100 MHz) of 38

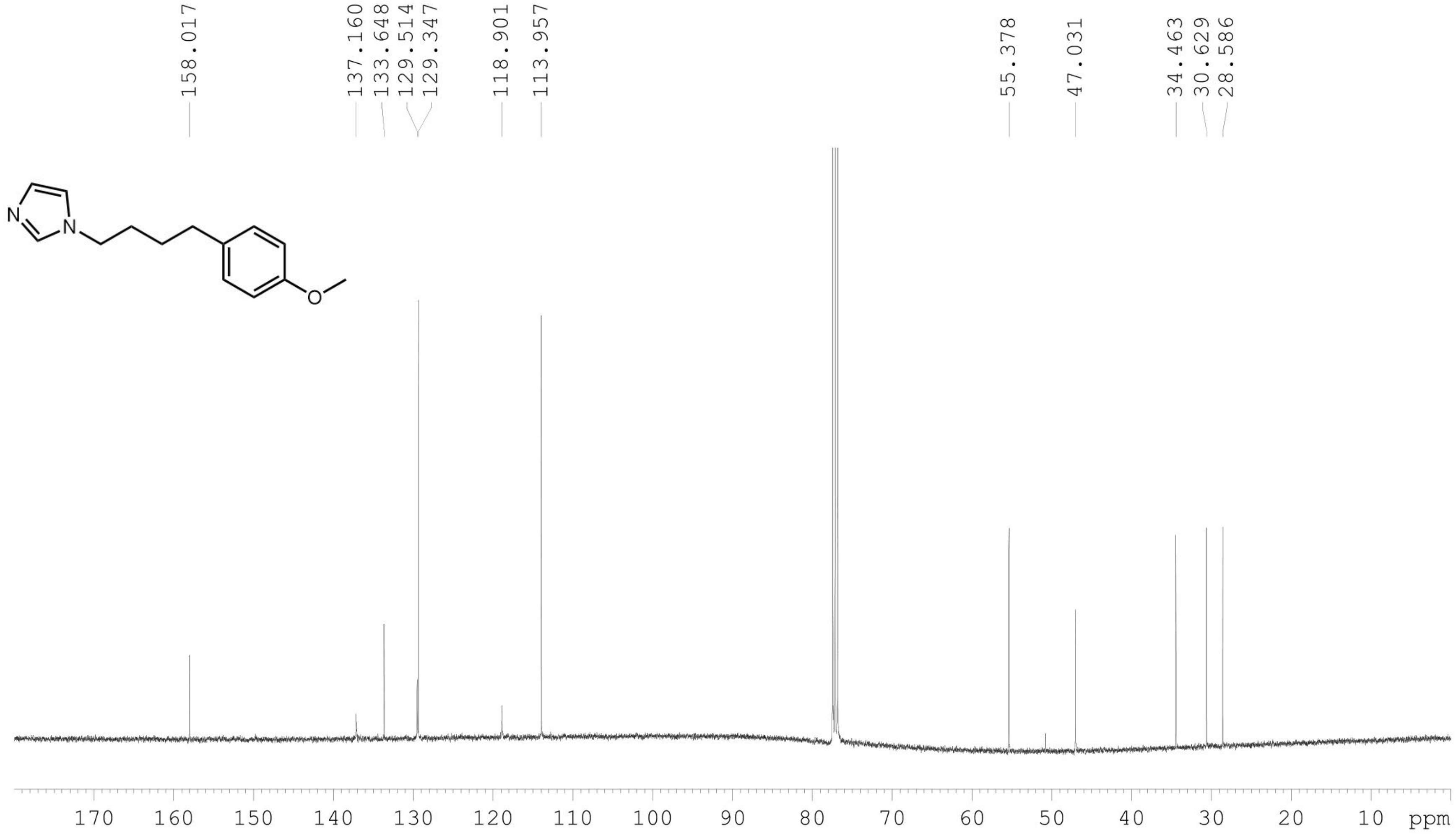

^{1}H NMR ($CDCl_3$, 400 MHz) of 39

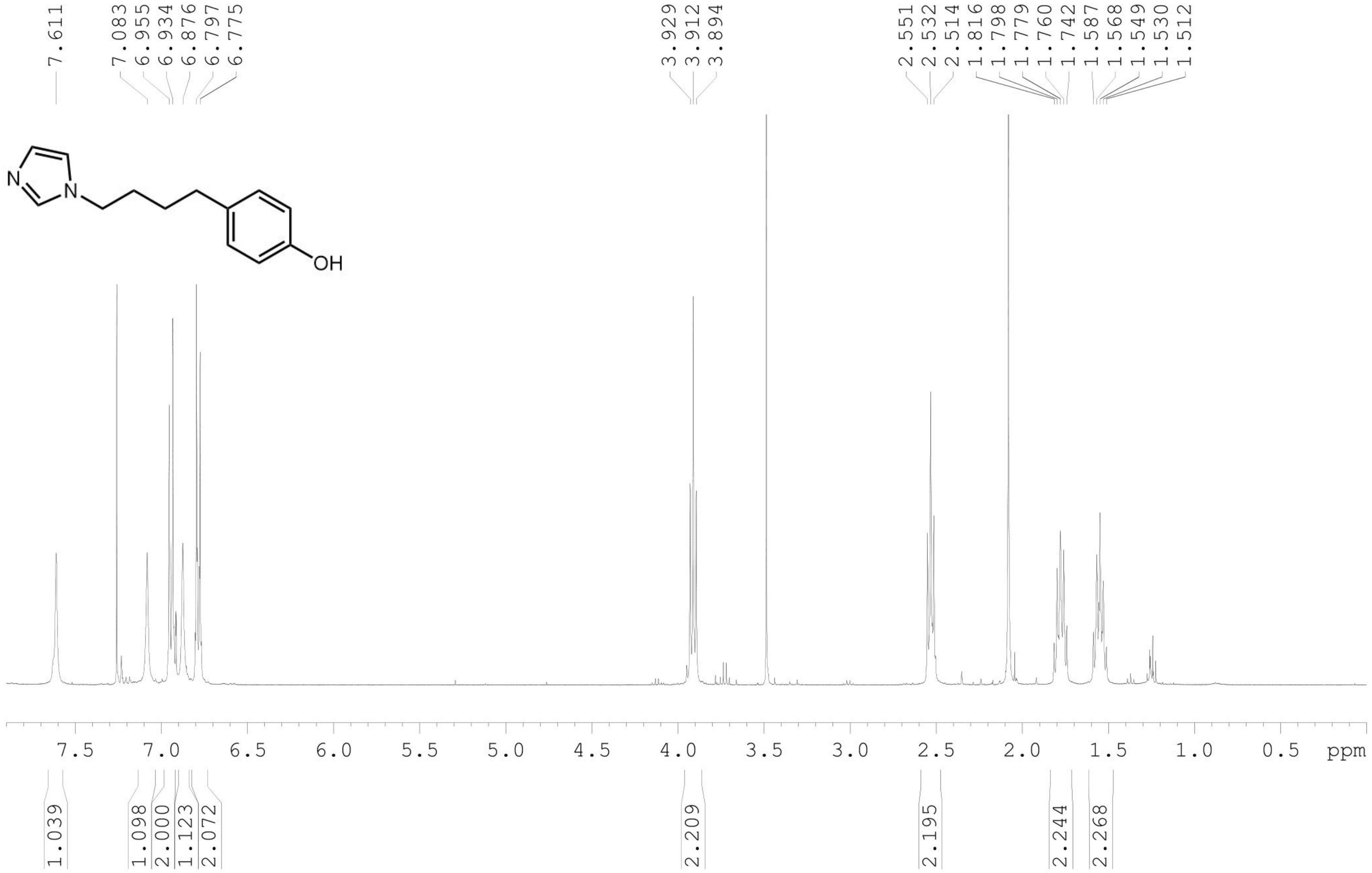

^{13}C NMR ($CDCl_3$, 100 MHz) of 39

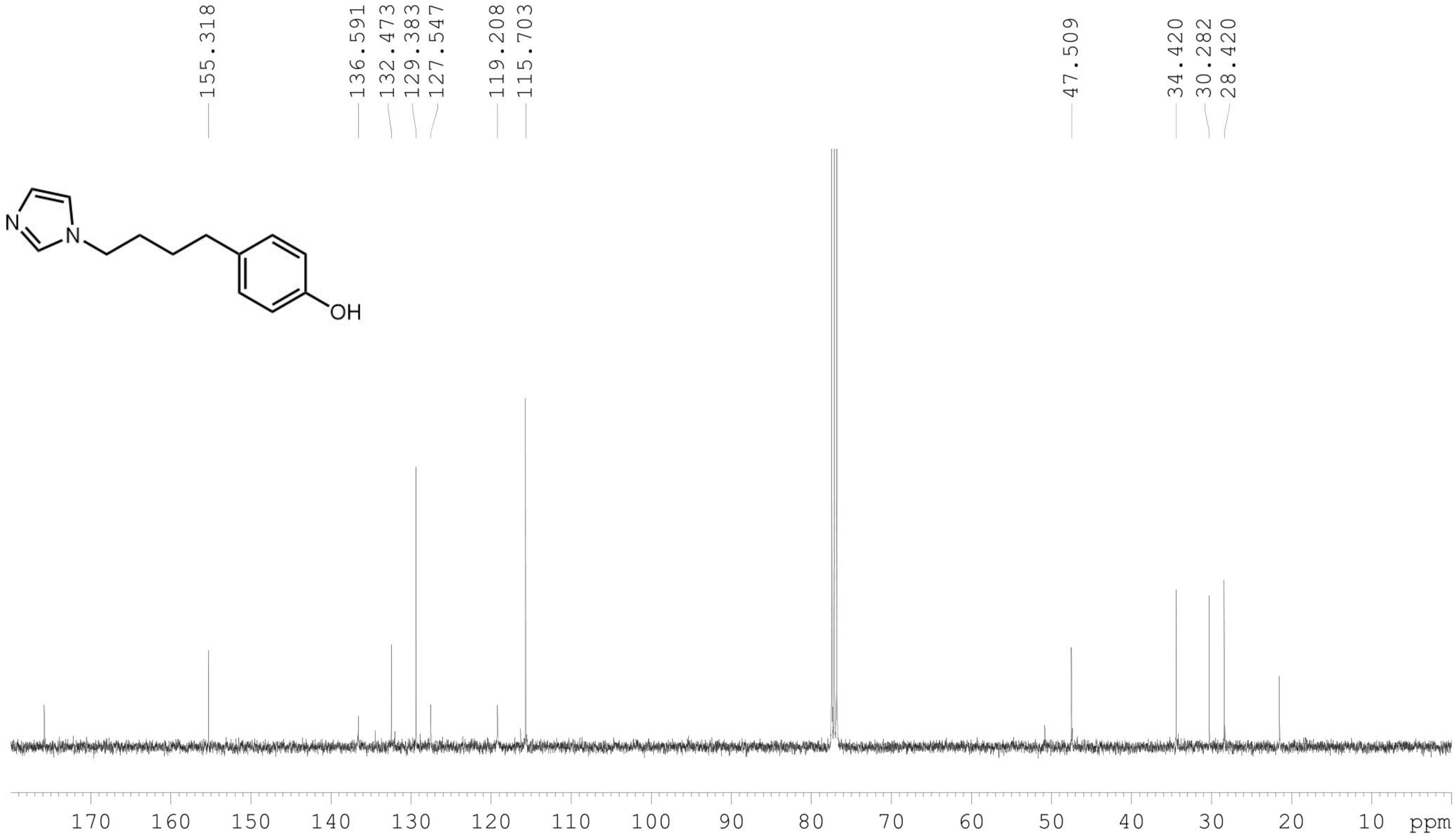

^{1}H NMR (DMSO, 400 MHz) of 40

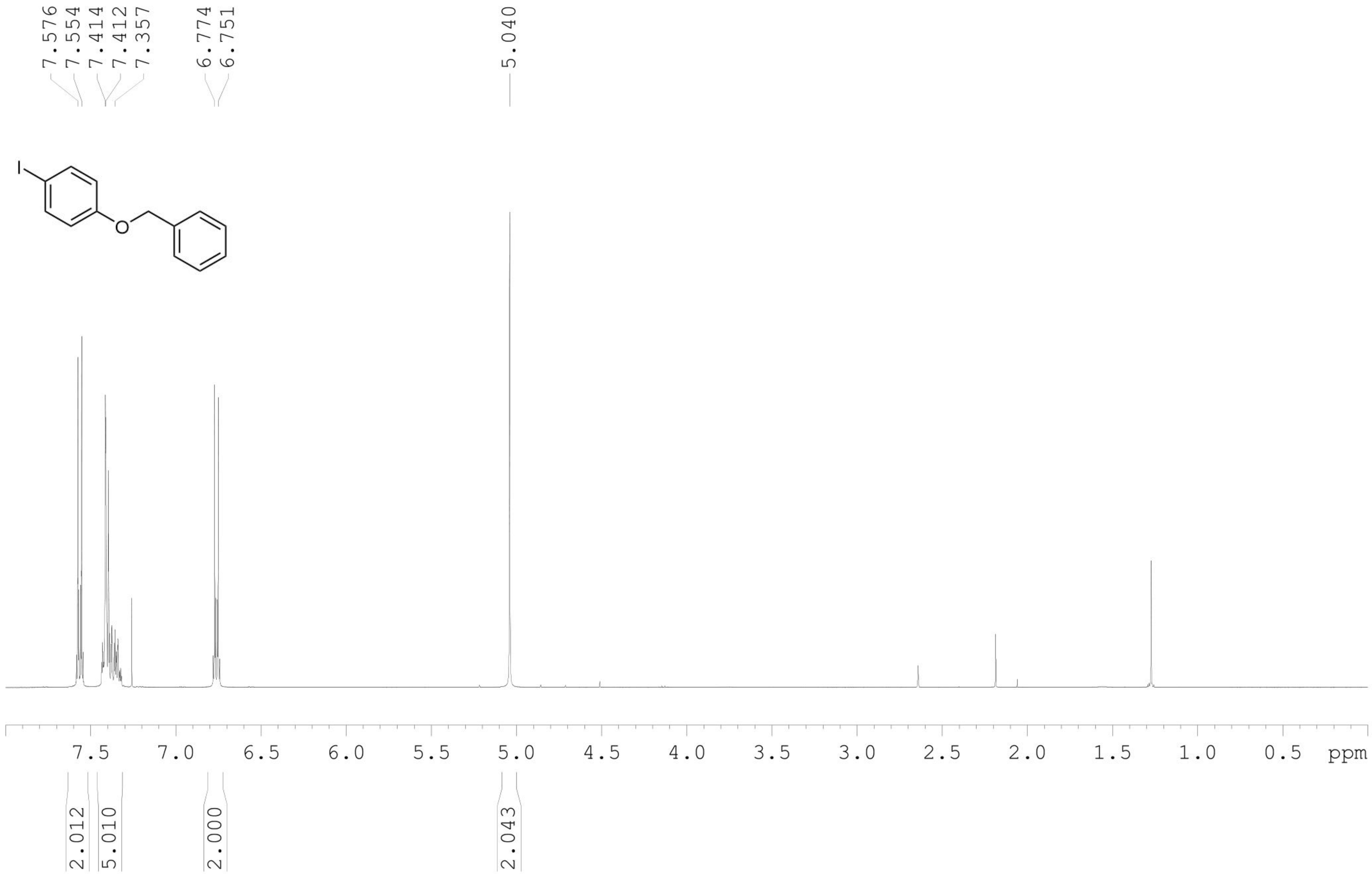

^{13}C NMR (DMSO, 100 MHz) of 40

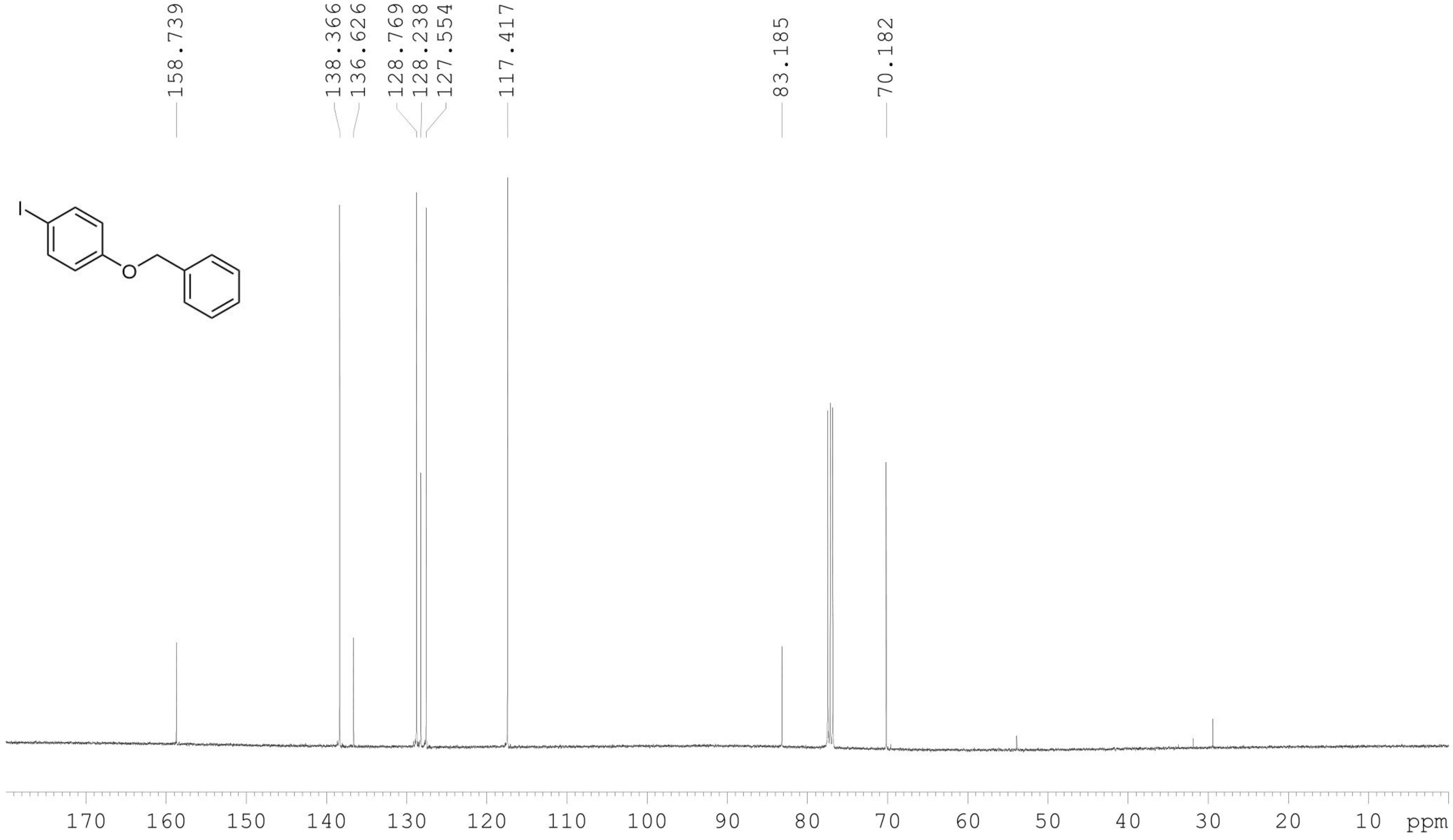

^{1}H NMR ($CDCl_3$, 400 MHz) of 41

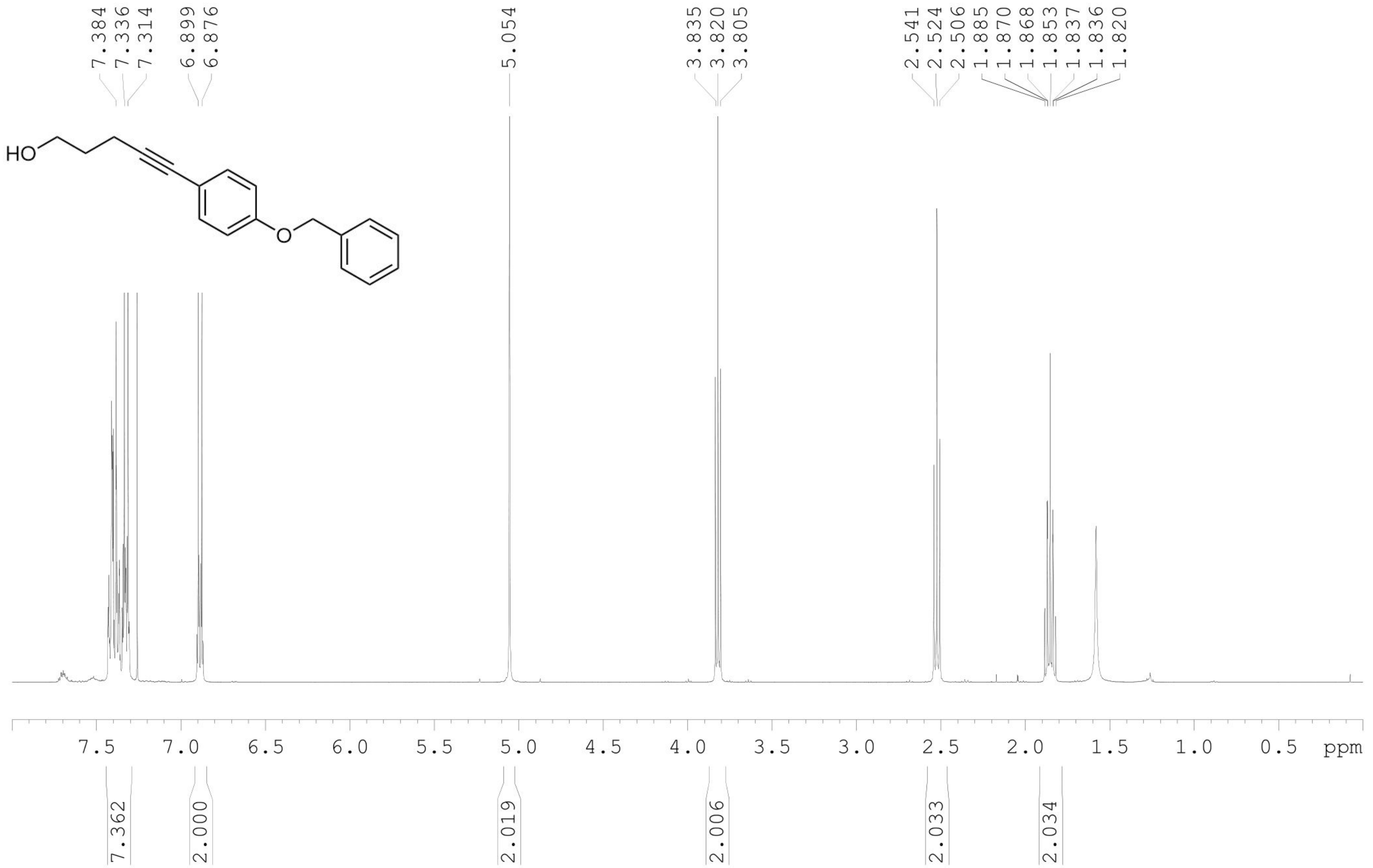

^{13}C NMR ($CDCl_3$, 100 MHz) of 41

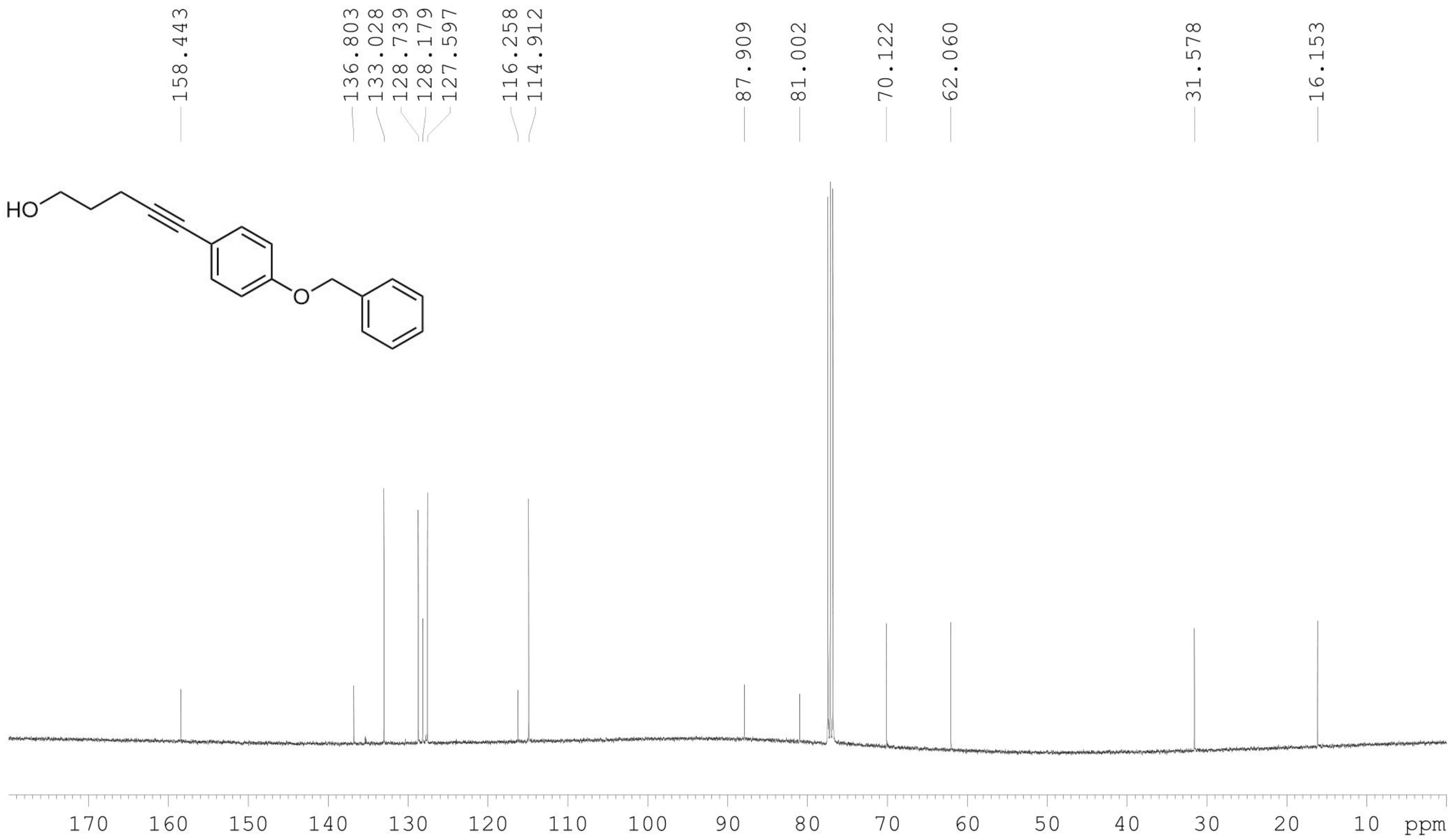

^{1}H NMR ($CDCl_3$, 400 MHz) of 42

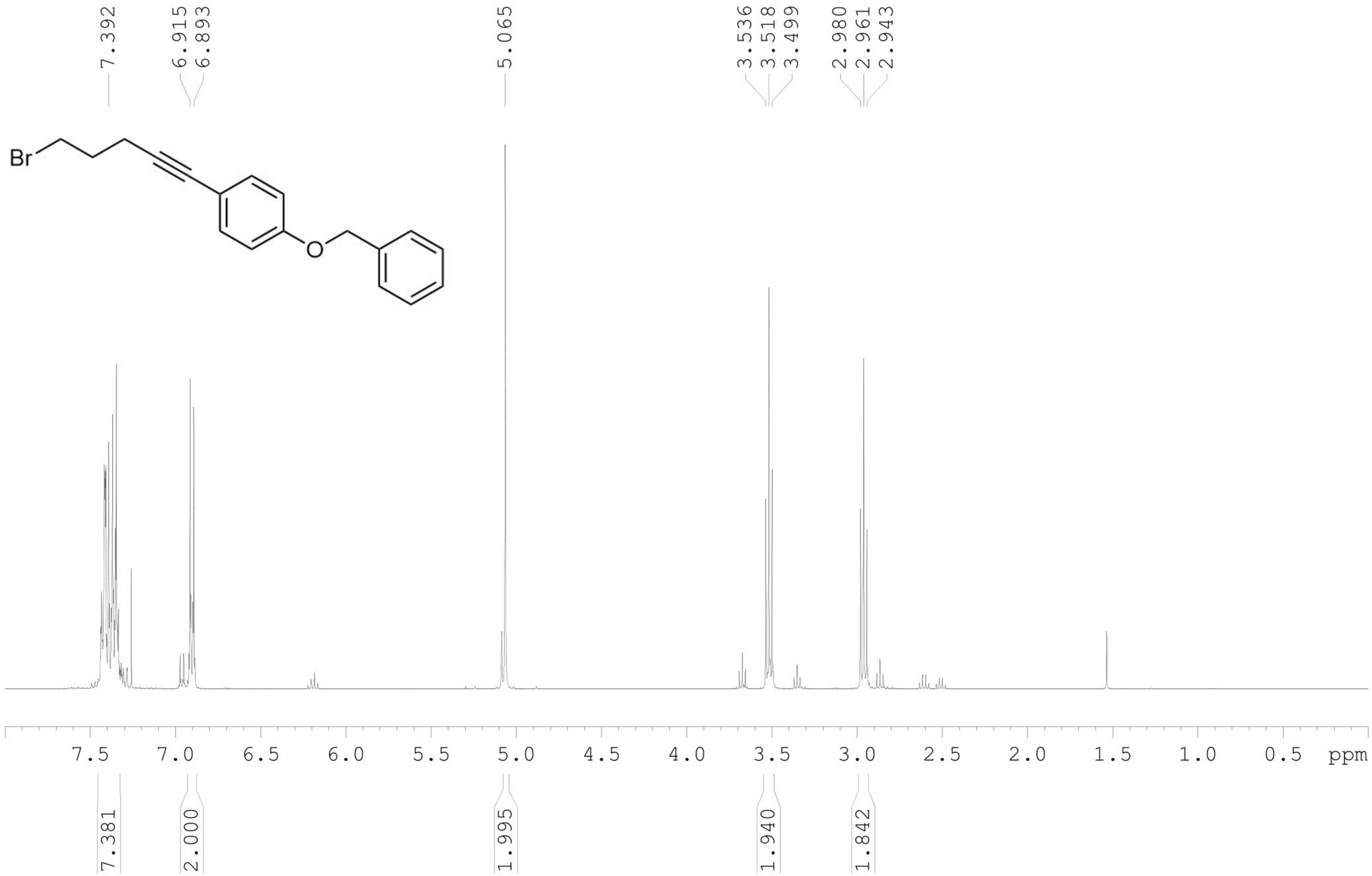

^{13}C NMR ($CDCl_3$, 100 MHz) of 42

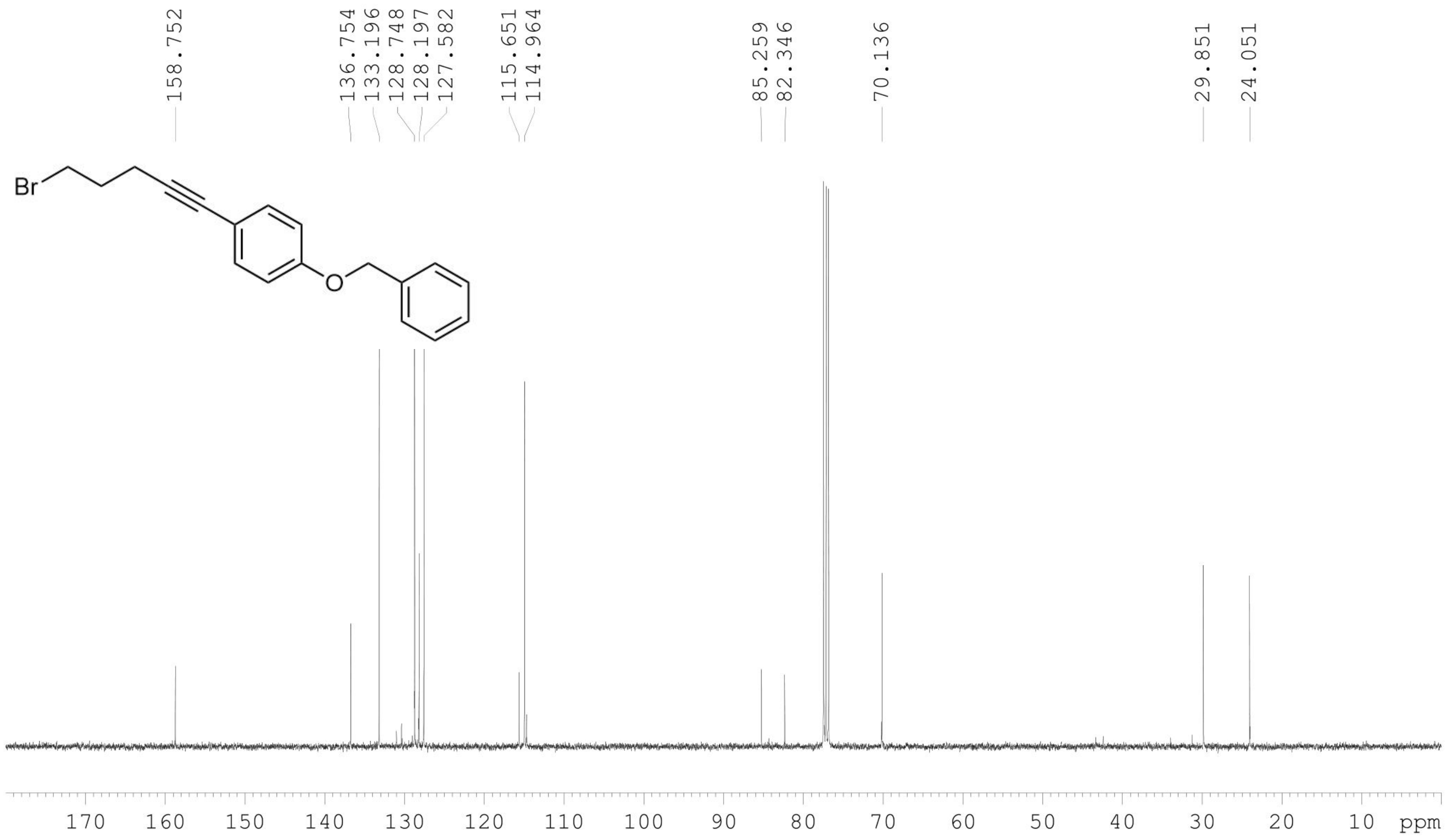

^{1}H NMR ($CDCl_3$, 400 MHz) of 43

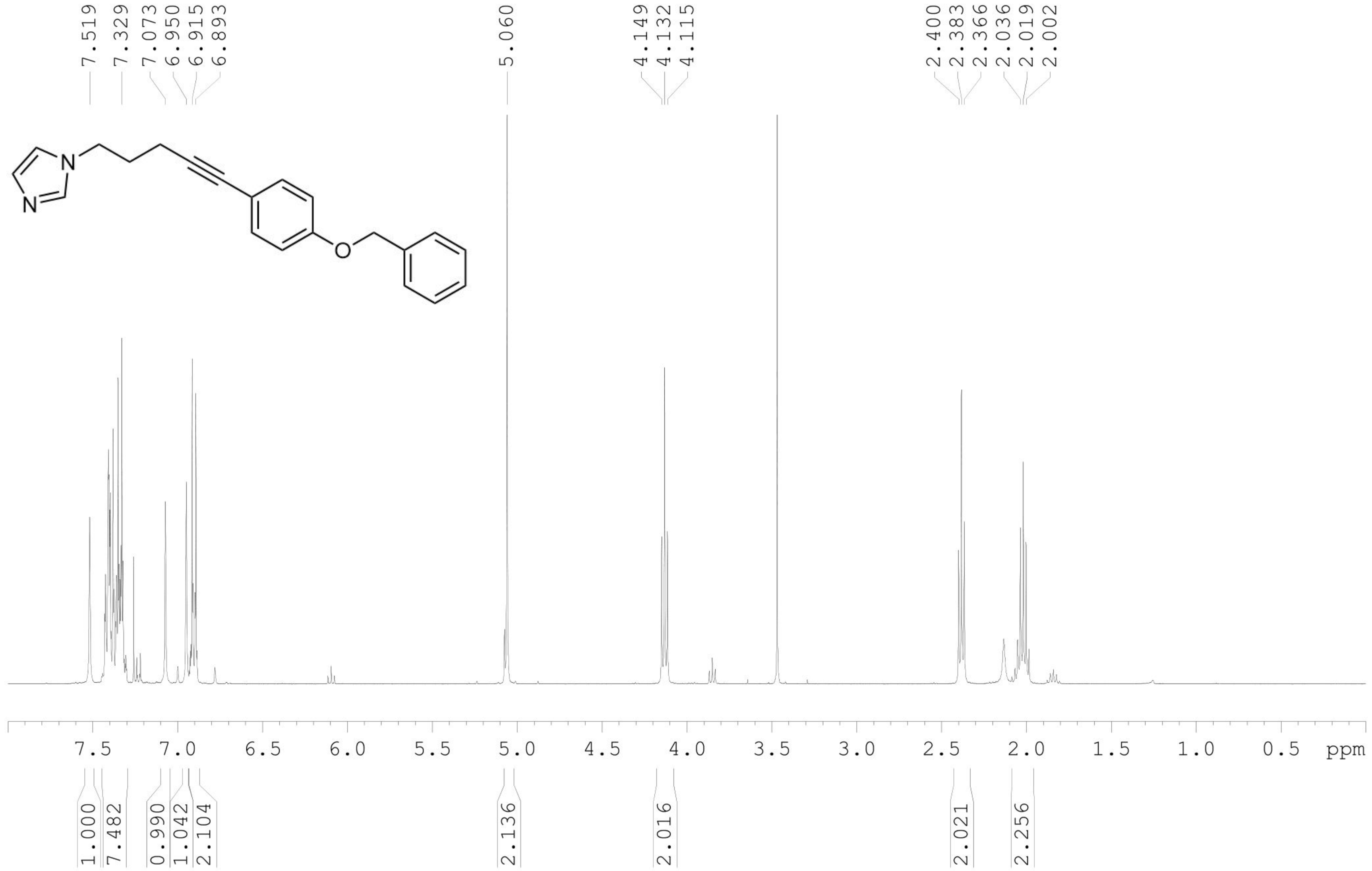

^{13}C NMR ($CDCl_3$, 100 MHz) of 43

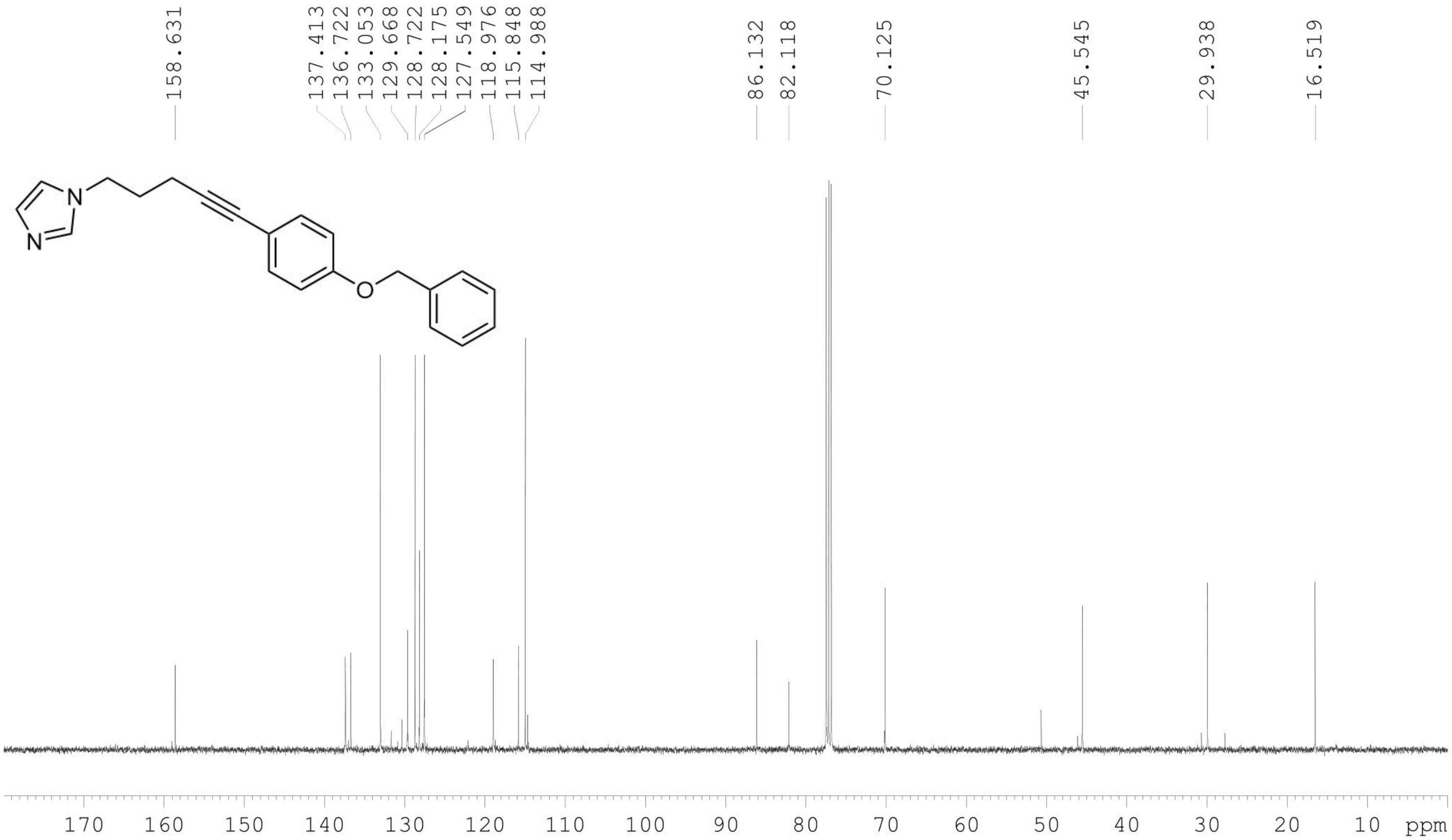

^{1}H NMR (DMSO, 400 MHz) of 44

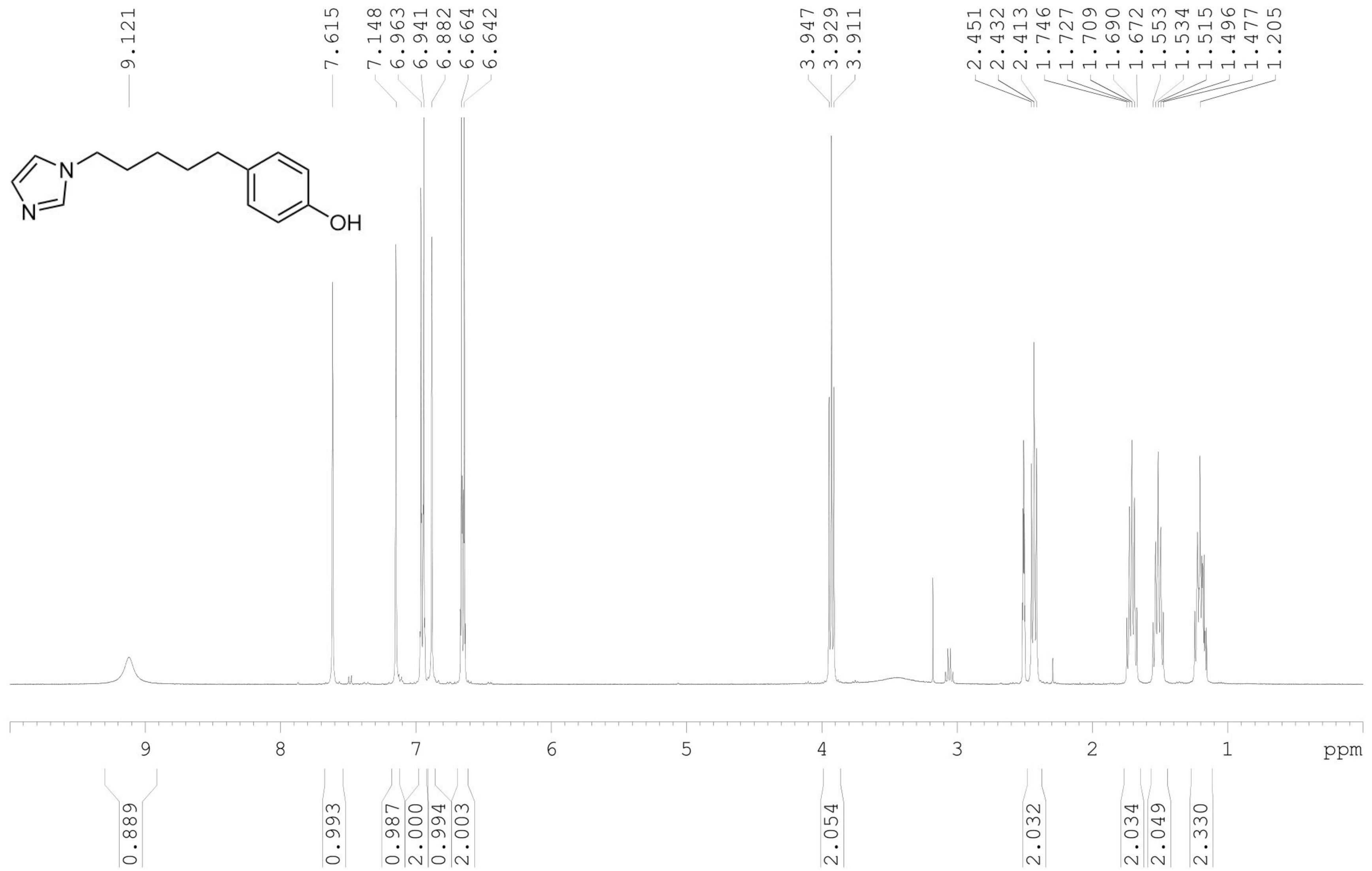

^{13}C NMR (DMSO, 100 MHz) of 44

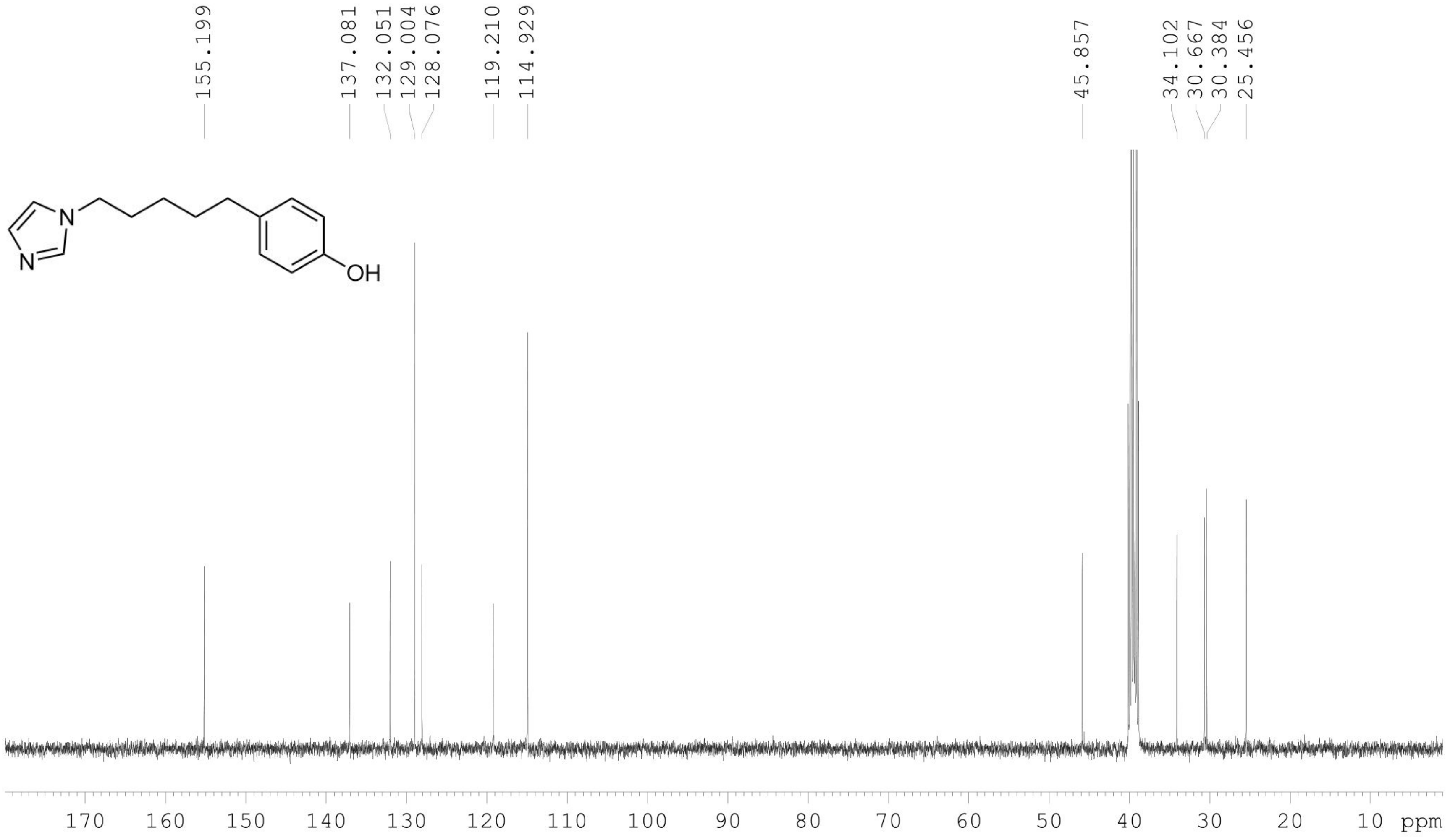

^{1}H NMR ($CDCl_3$, 400 MHz) of 45

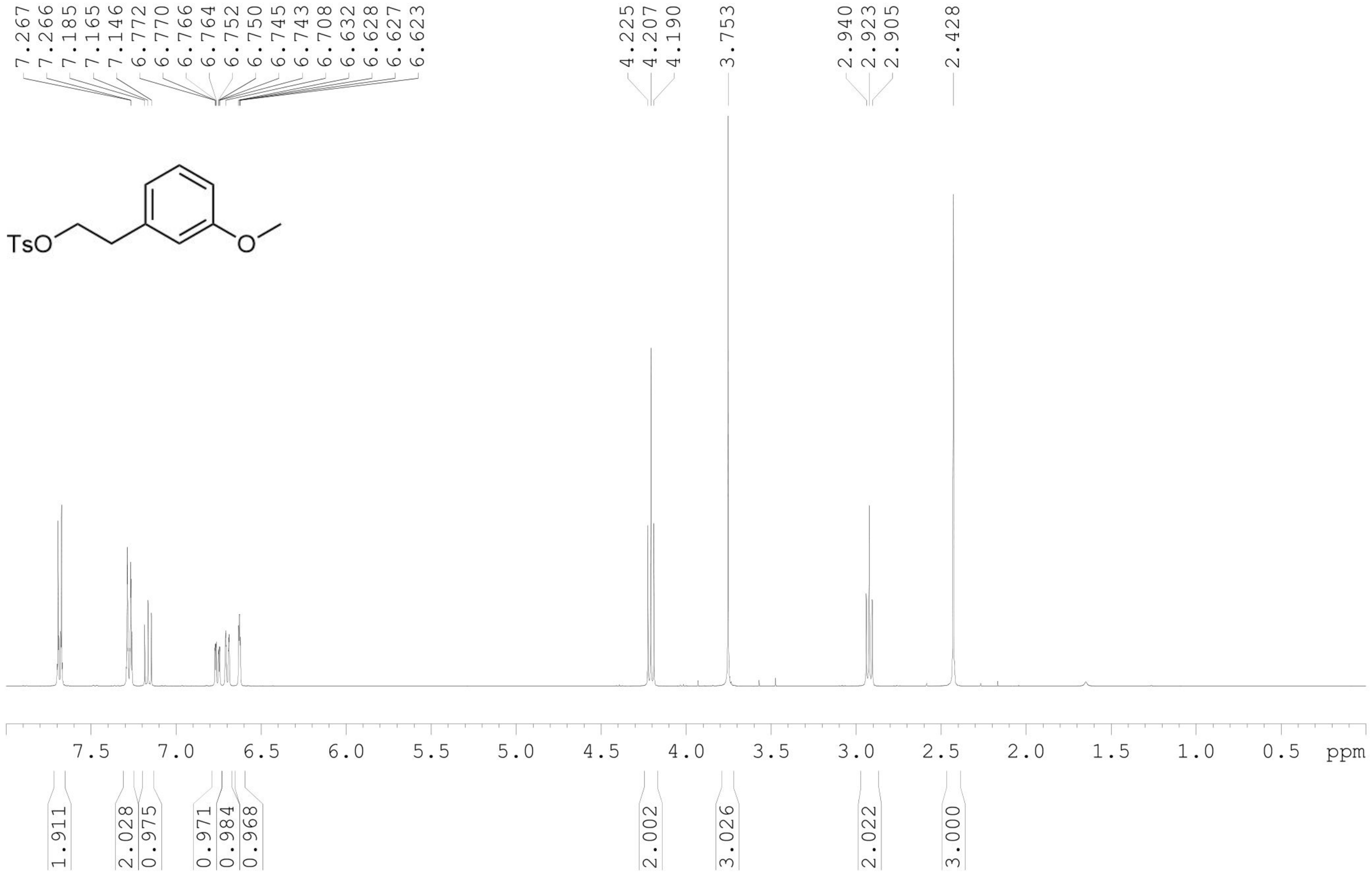

^{13}C NMR ($CDCl_3$, 100 MHz) of 45

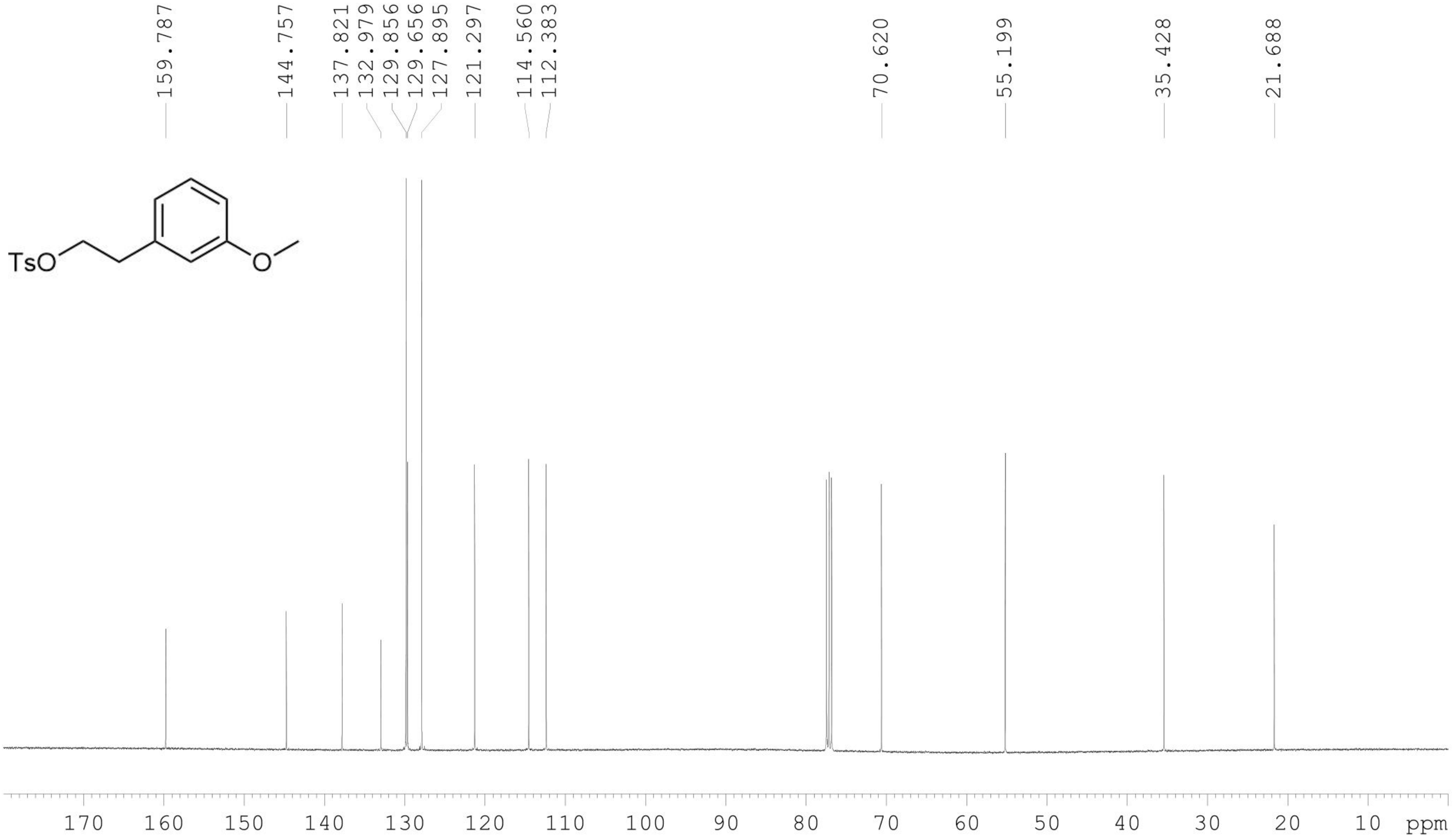

^{1}H NMR ($CDCl_3$, 400 MHz) of 46

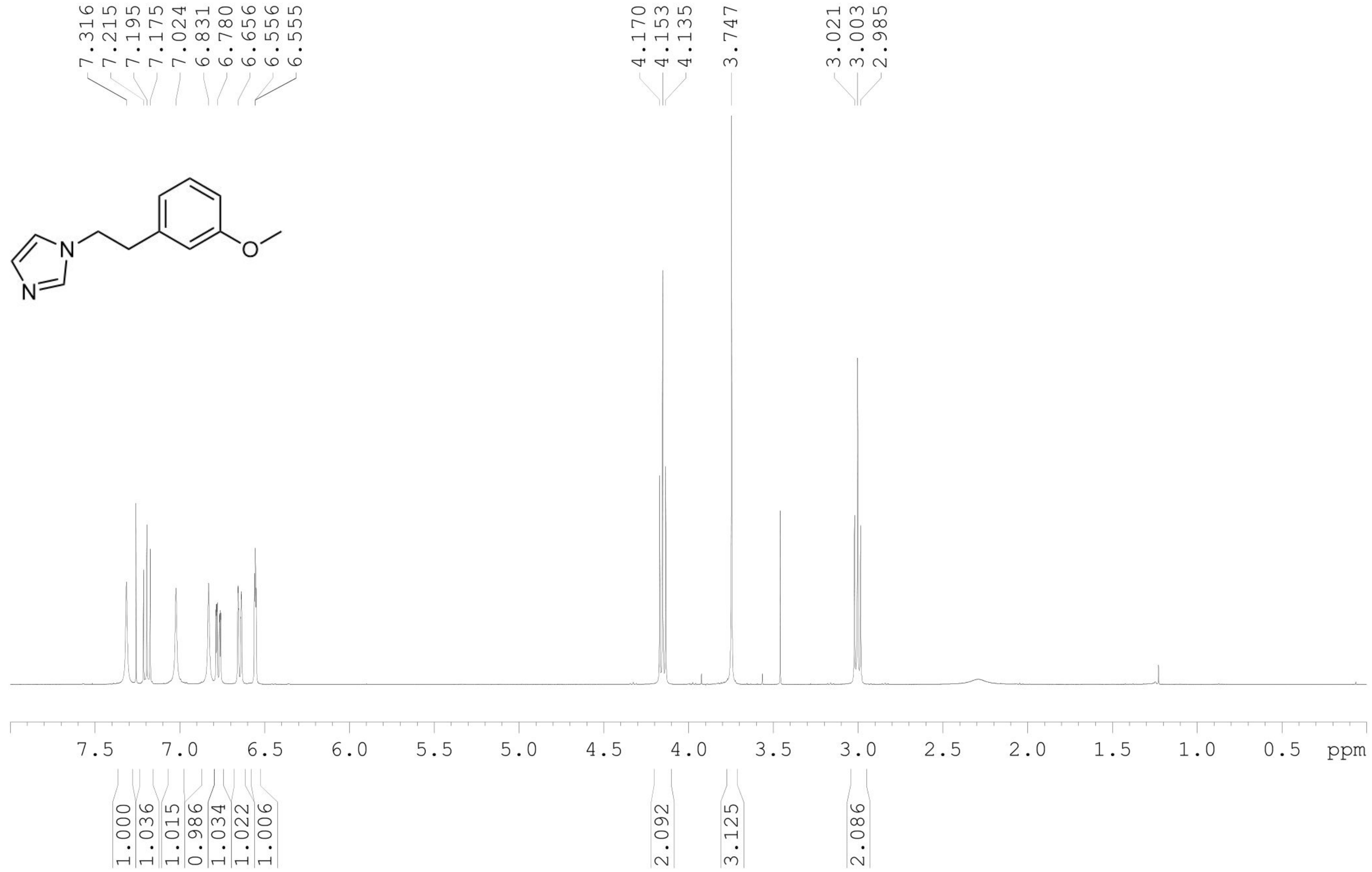

^{13}C NMR ($CDCl_3$, 100 MHz) of 46

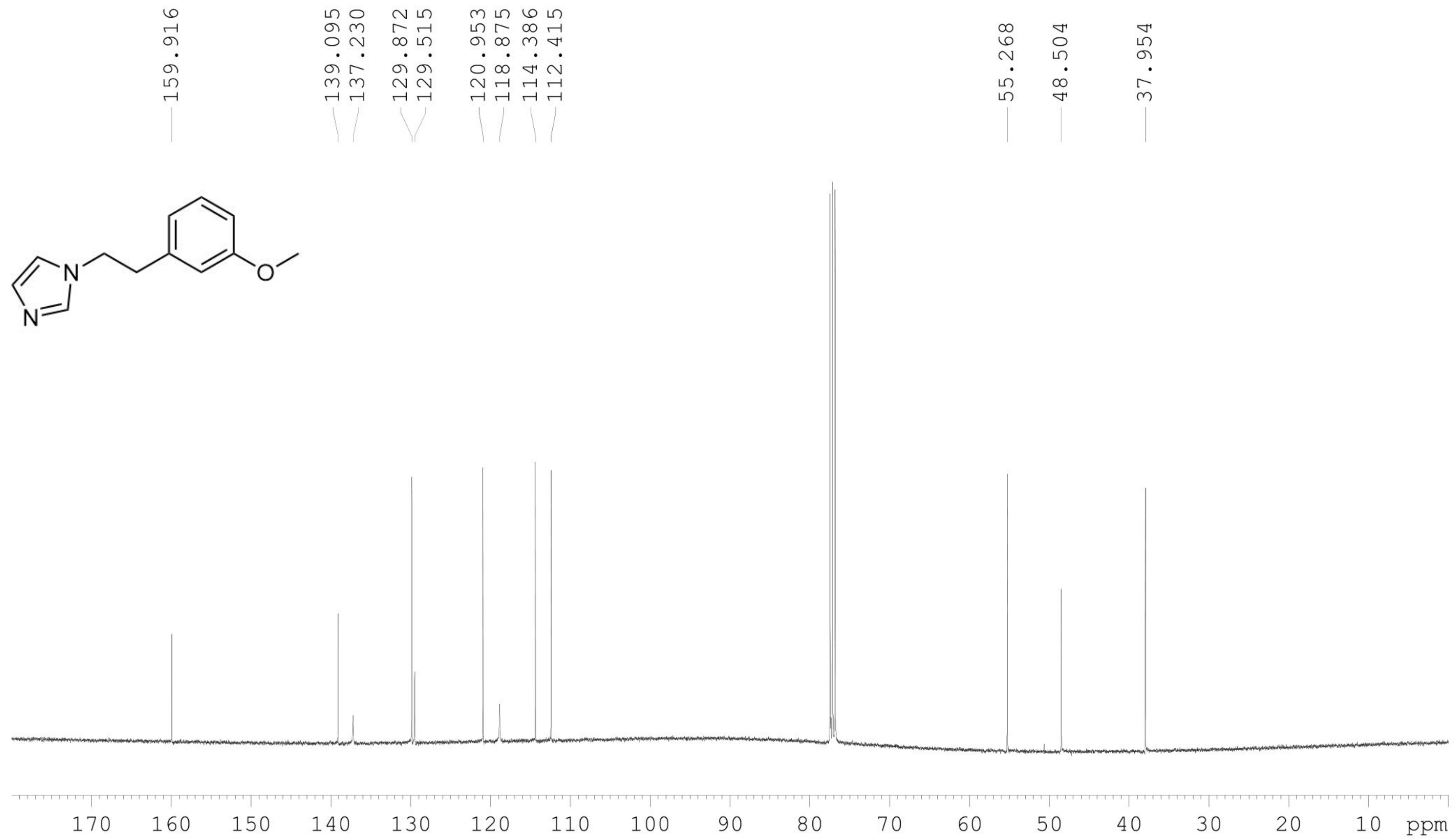

^{1}H NMR (DMSO, 400 MHz) of 47

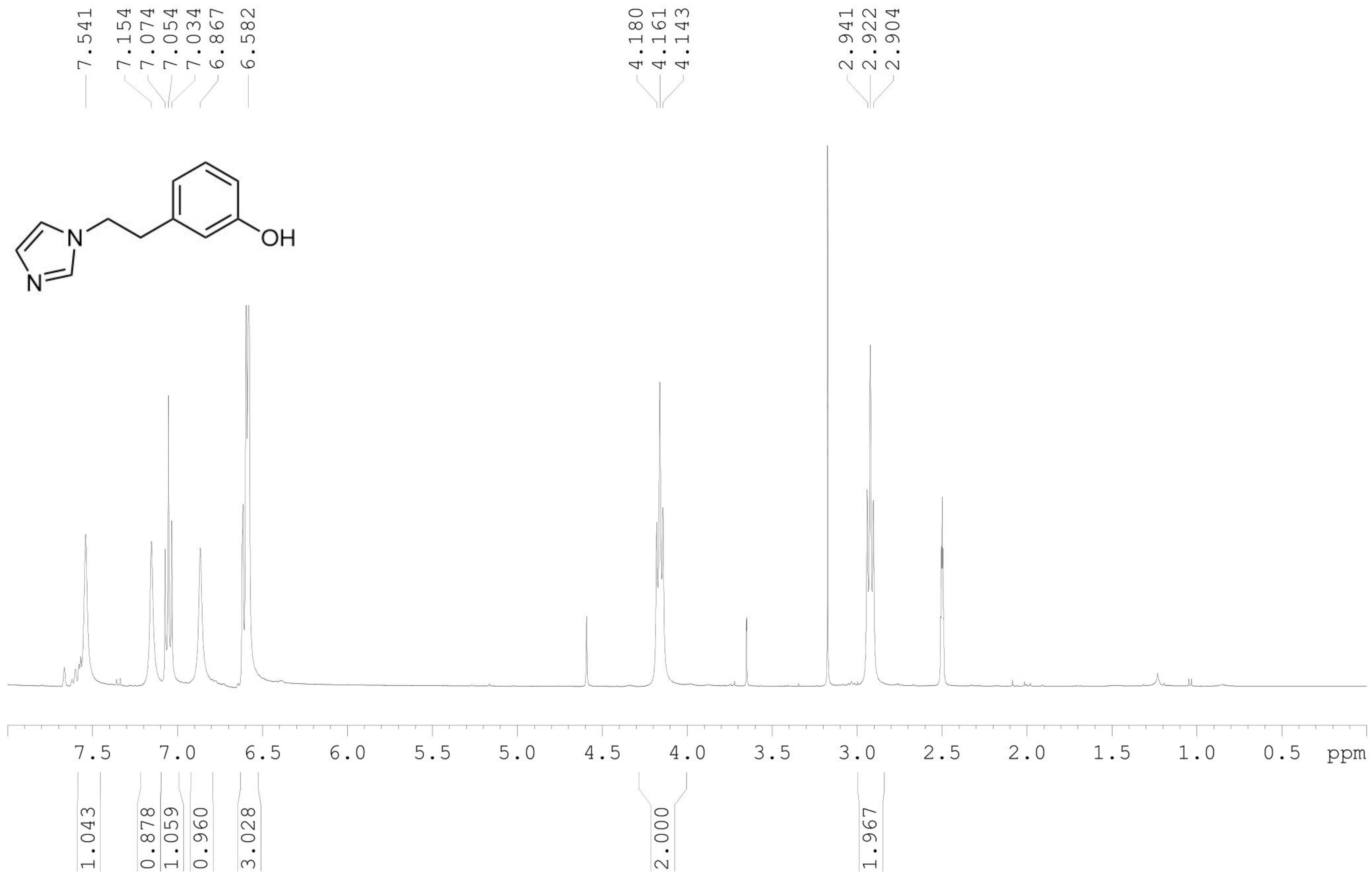

^{13}C NMR (DMSO, 100 MHz) of 47

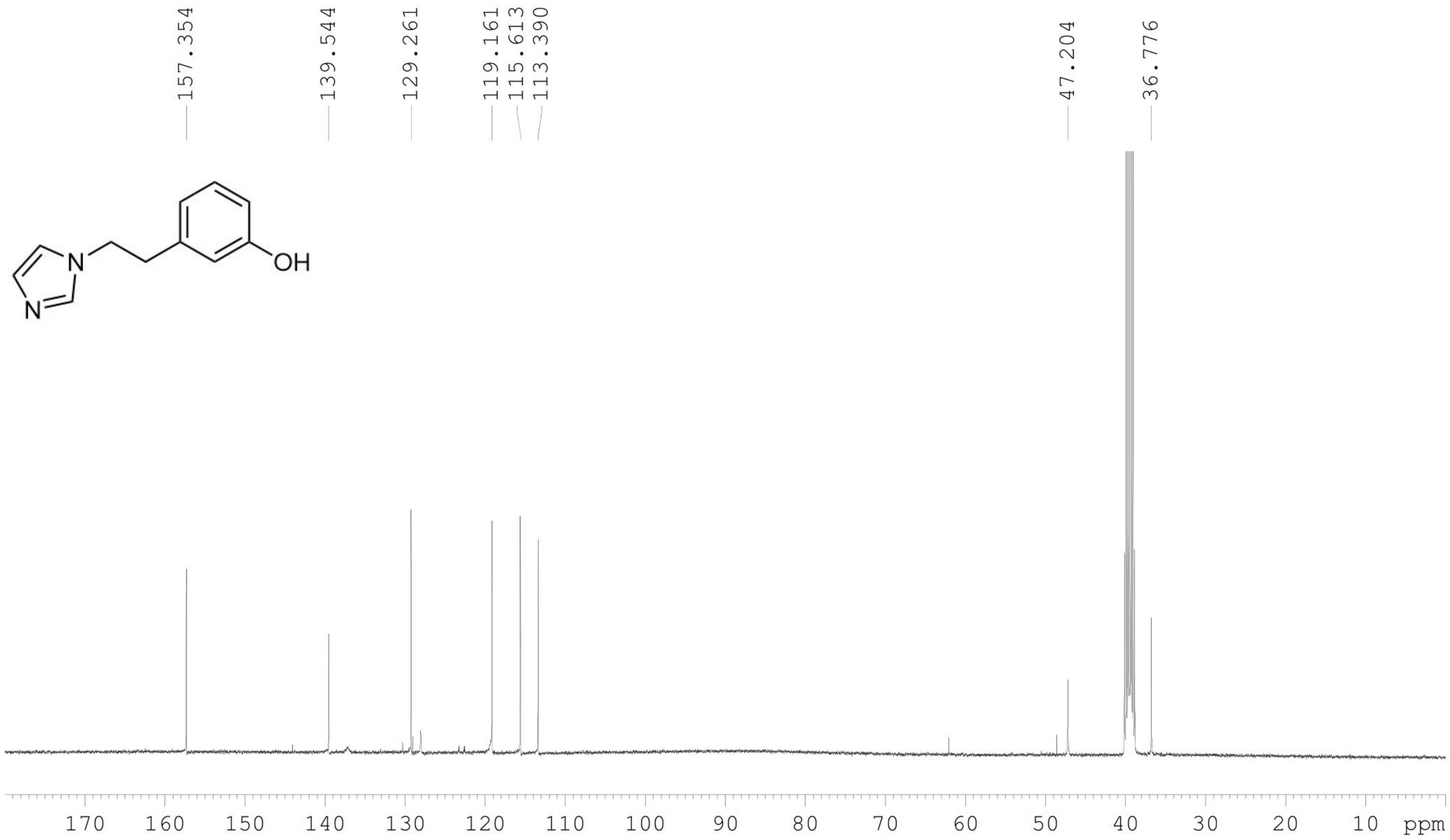

^{1}H NMR ($CDCl_3$, 400 MHz) of 48

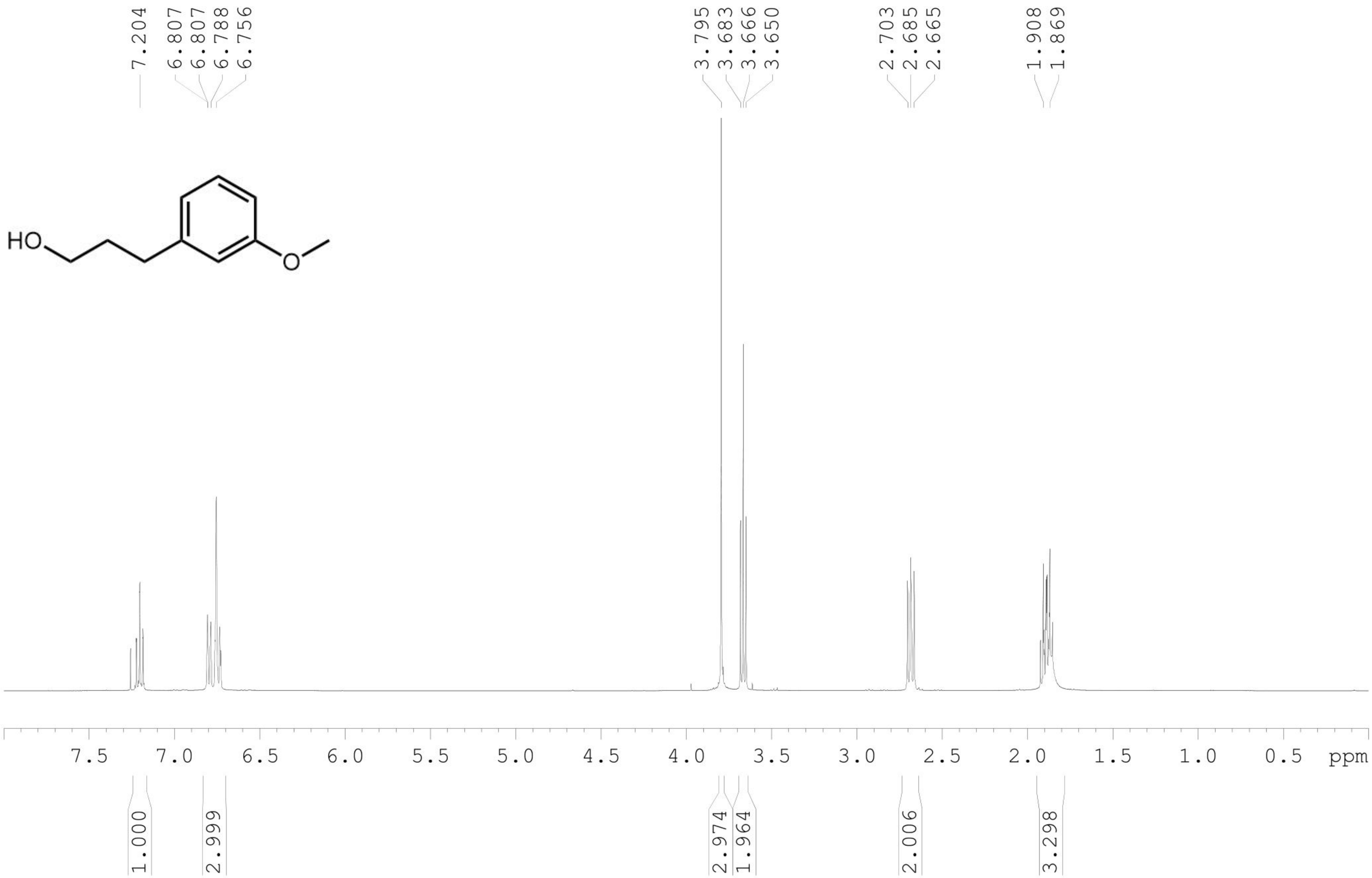

^{13}C NMR ($CDCl_3$, 100 MHz) of 48

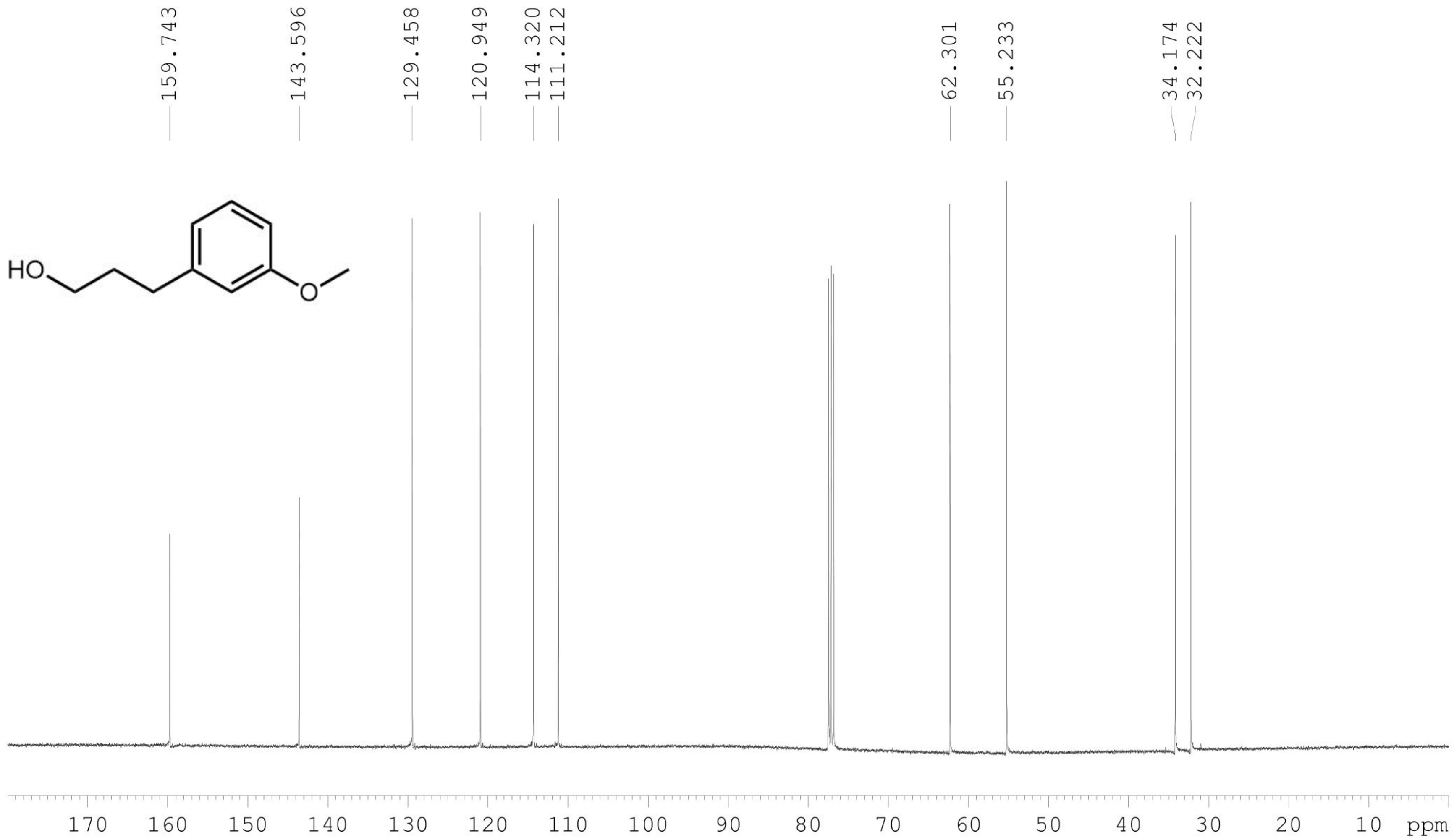

^{1}H NMR ($CDCl_3$, 400 MHz) of 49

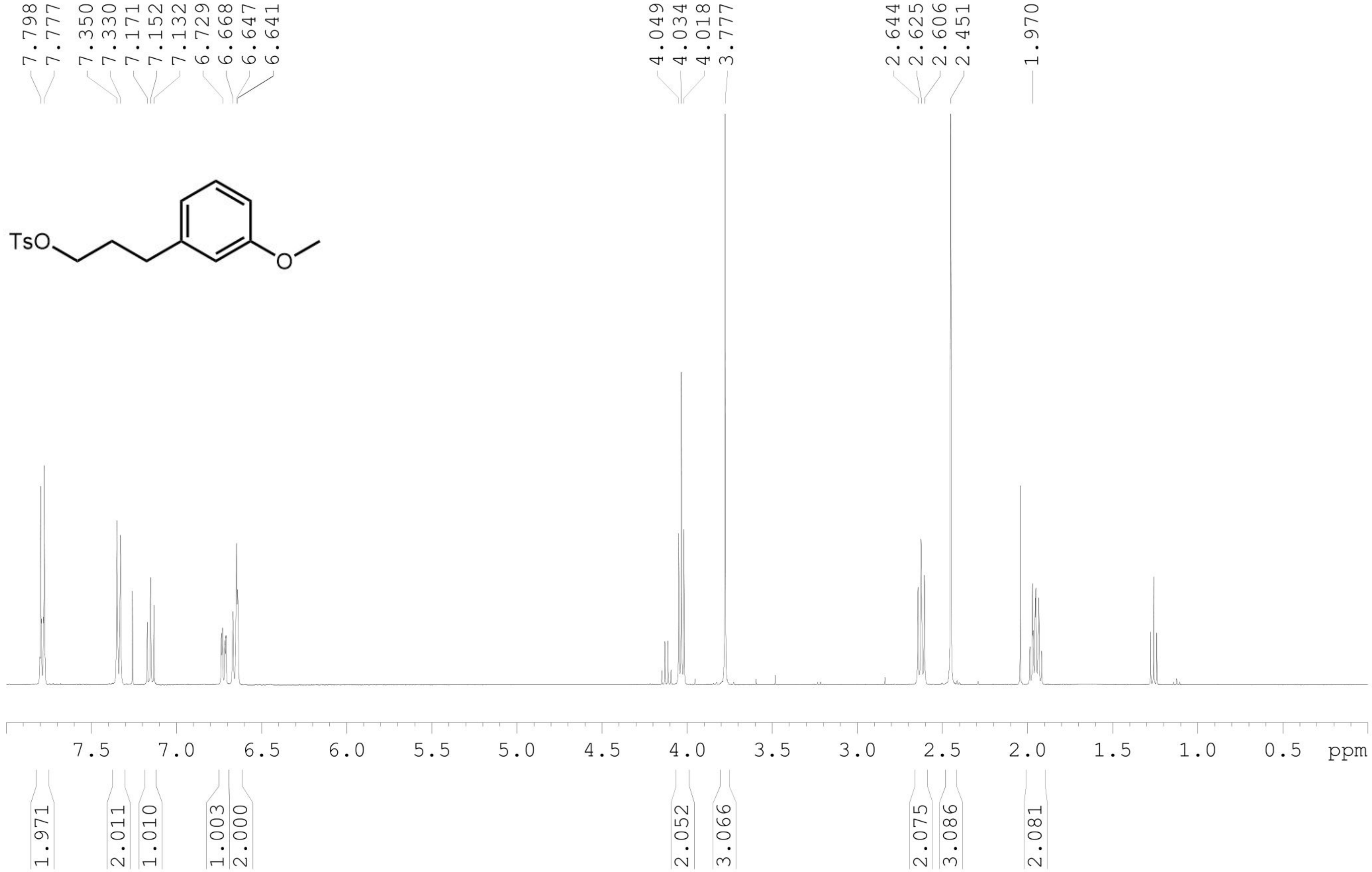

^{13}C NMR ($CDCl_3$, 100 MHz) of 49

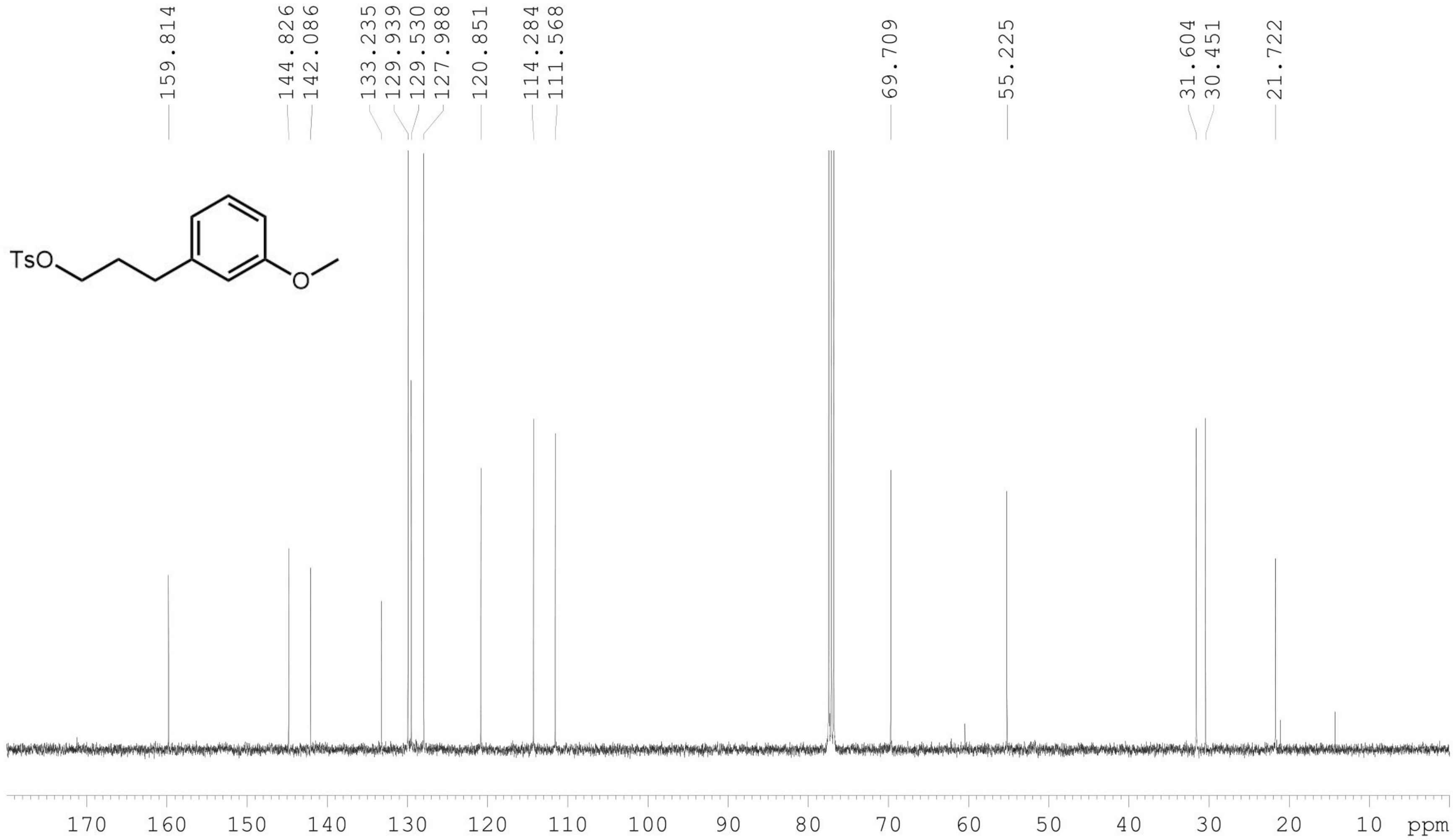

^{1}H NMR ($CDCl_3$, 400 MHz) of 50

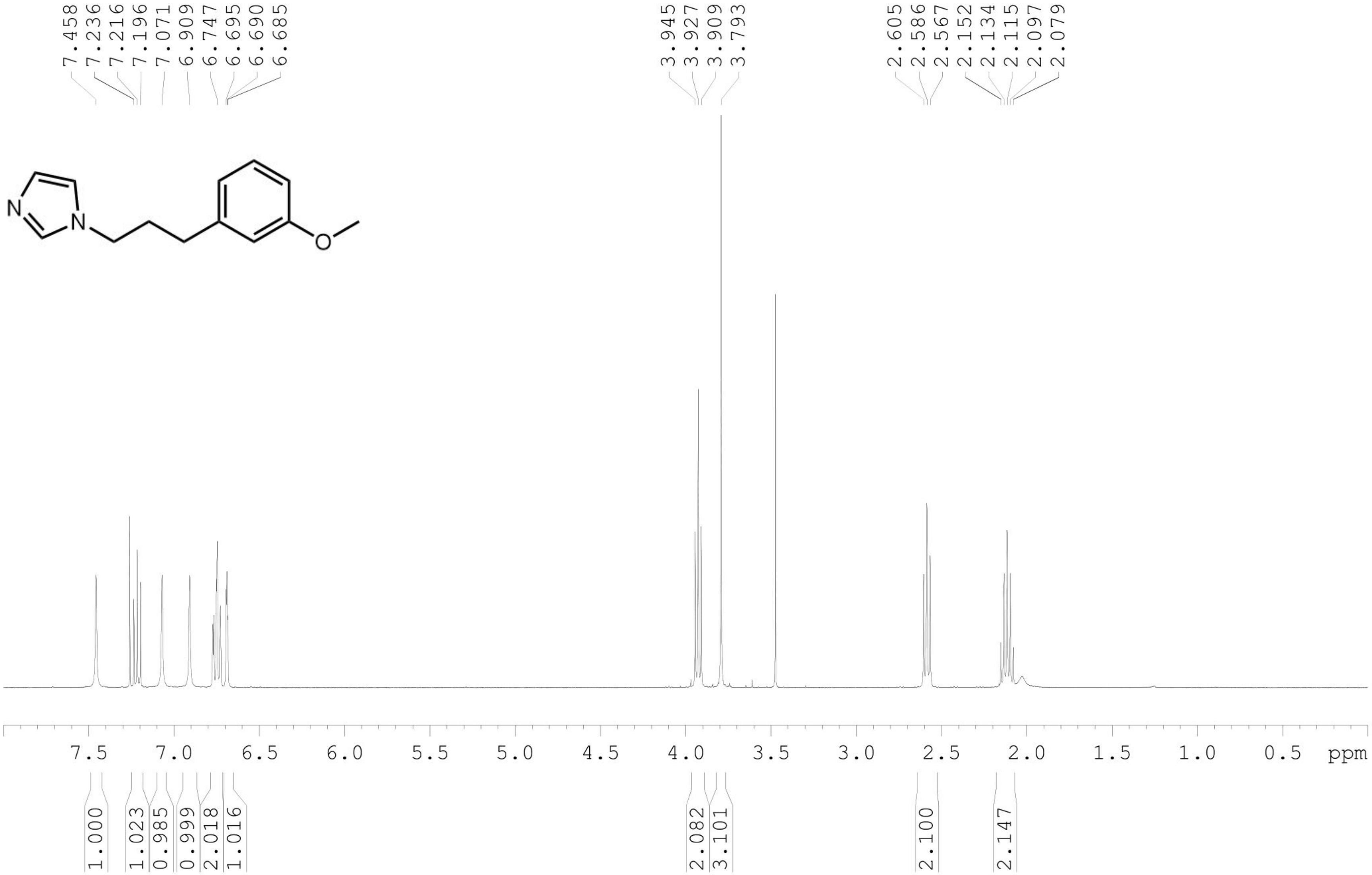

^{13}C NMR ($CDCl_3$, 100 MHz) of 50

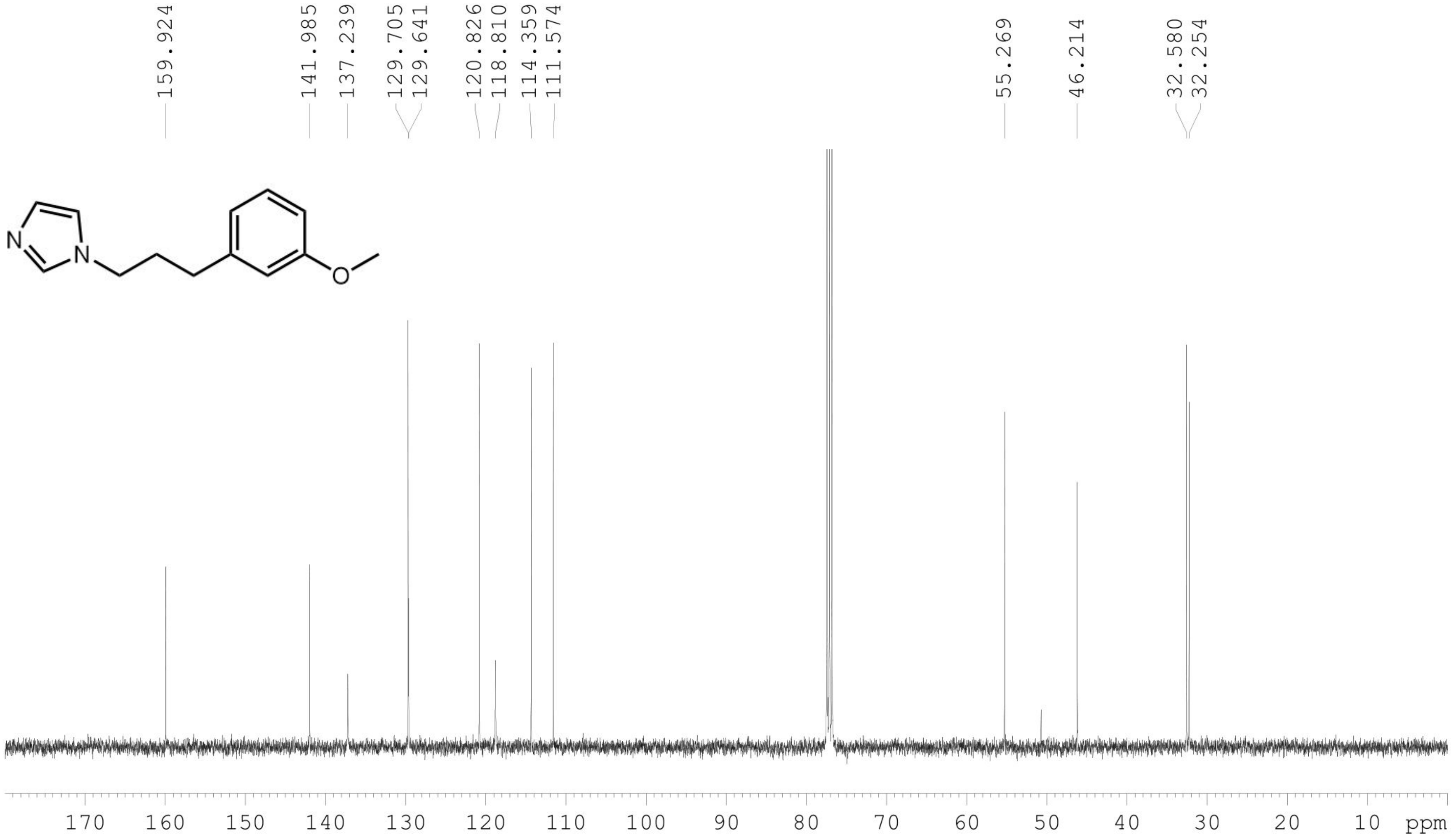

^{1}H NMR ($CDCl_3$, 400 MHz) of 51

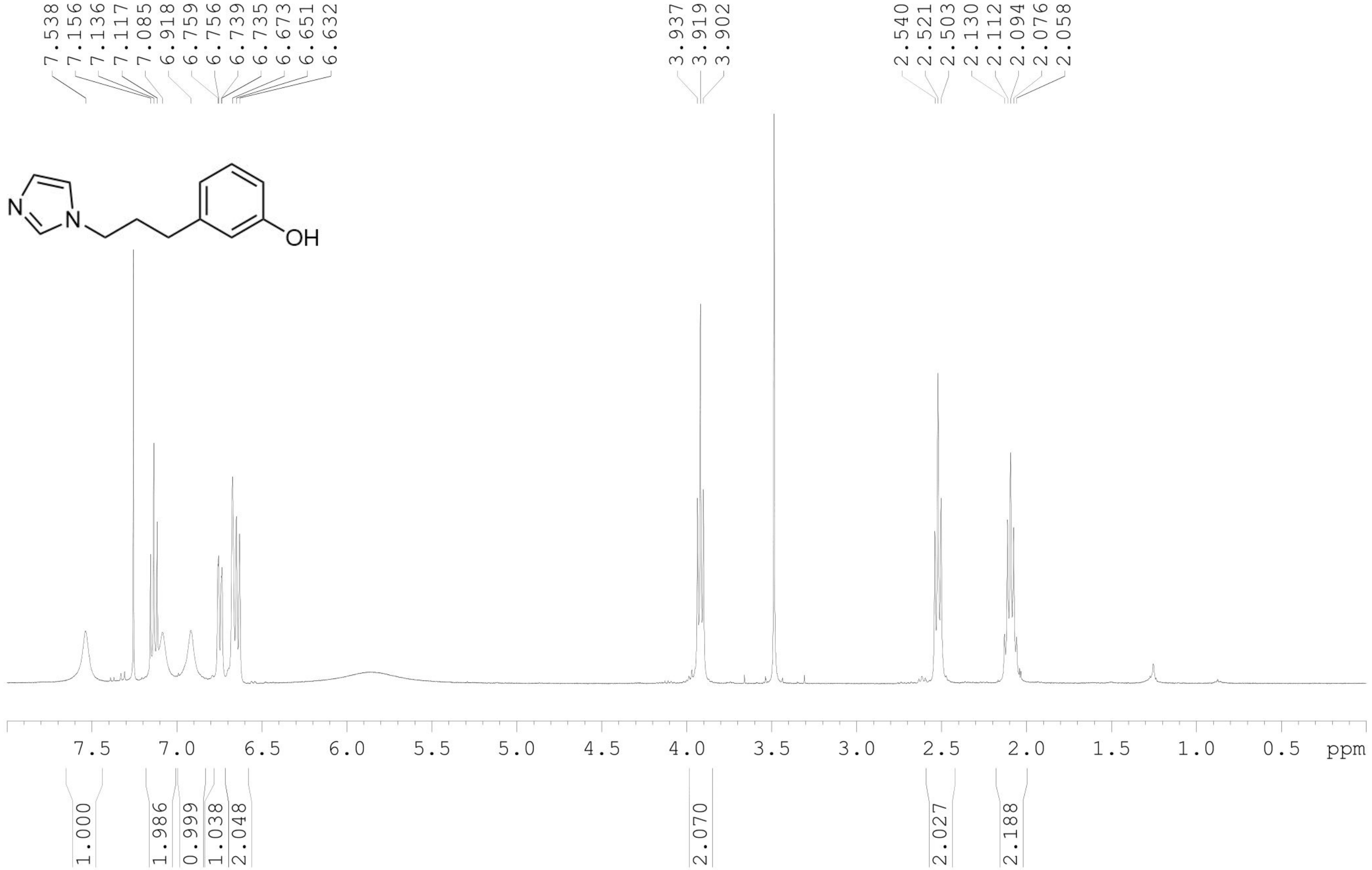

^{13}C NMR ($CDCl_3$, 100 MHz) of 51

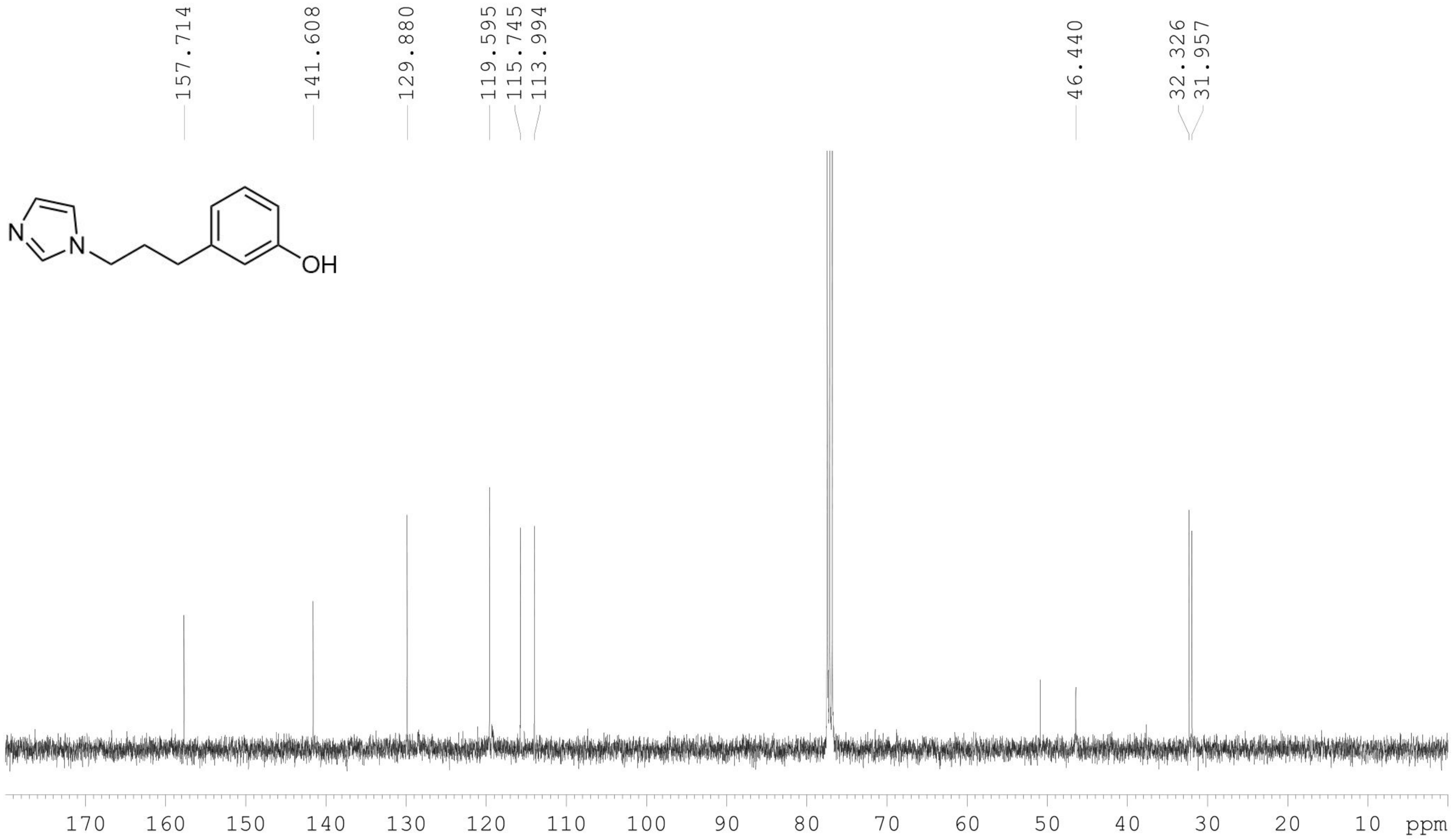

^{1}H NMR ($CDCl_3$, 400 MHz) of 52

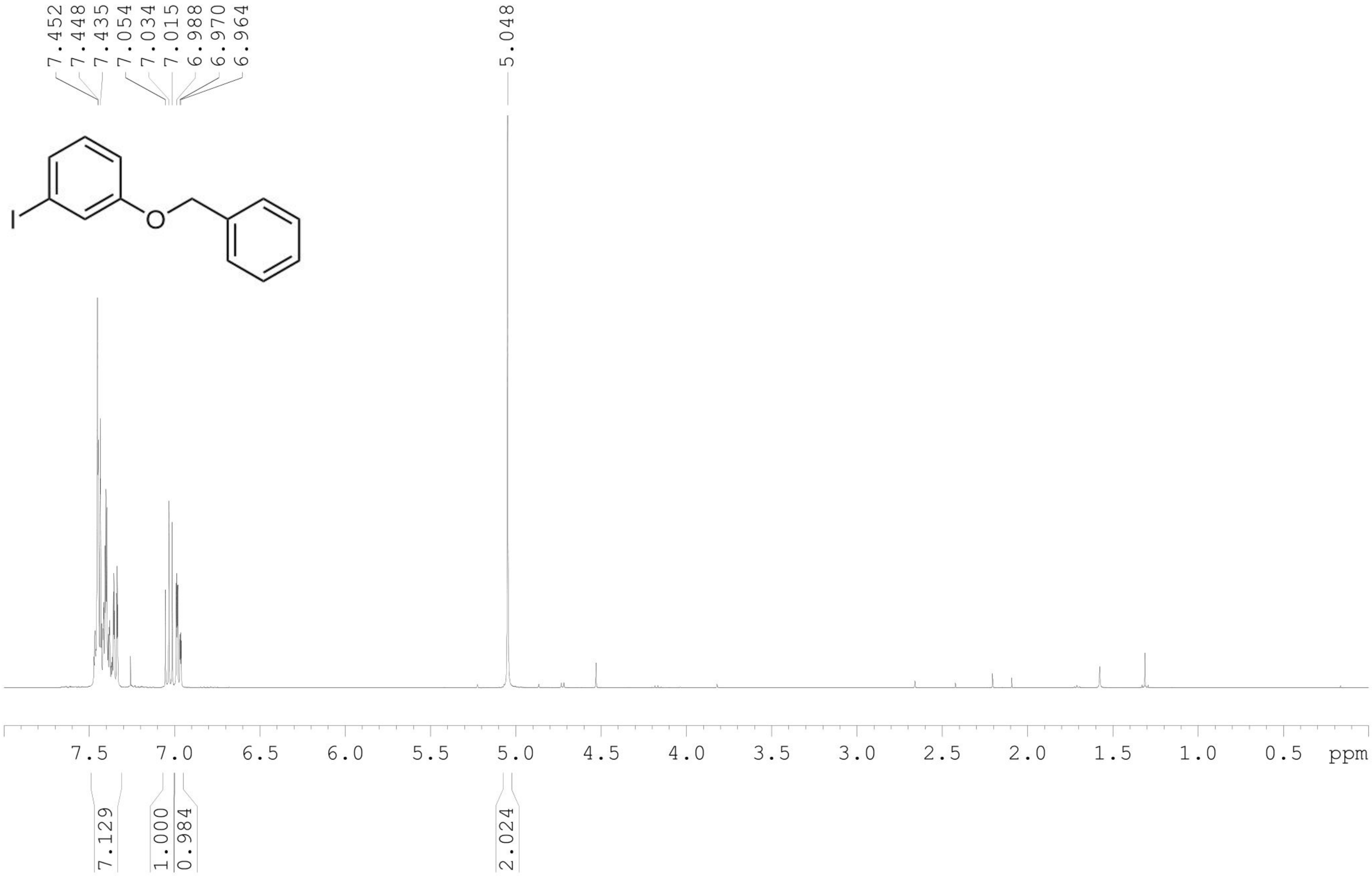

^{13}C NMR ($CDCl_3$, 100 MHz) of 52

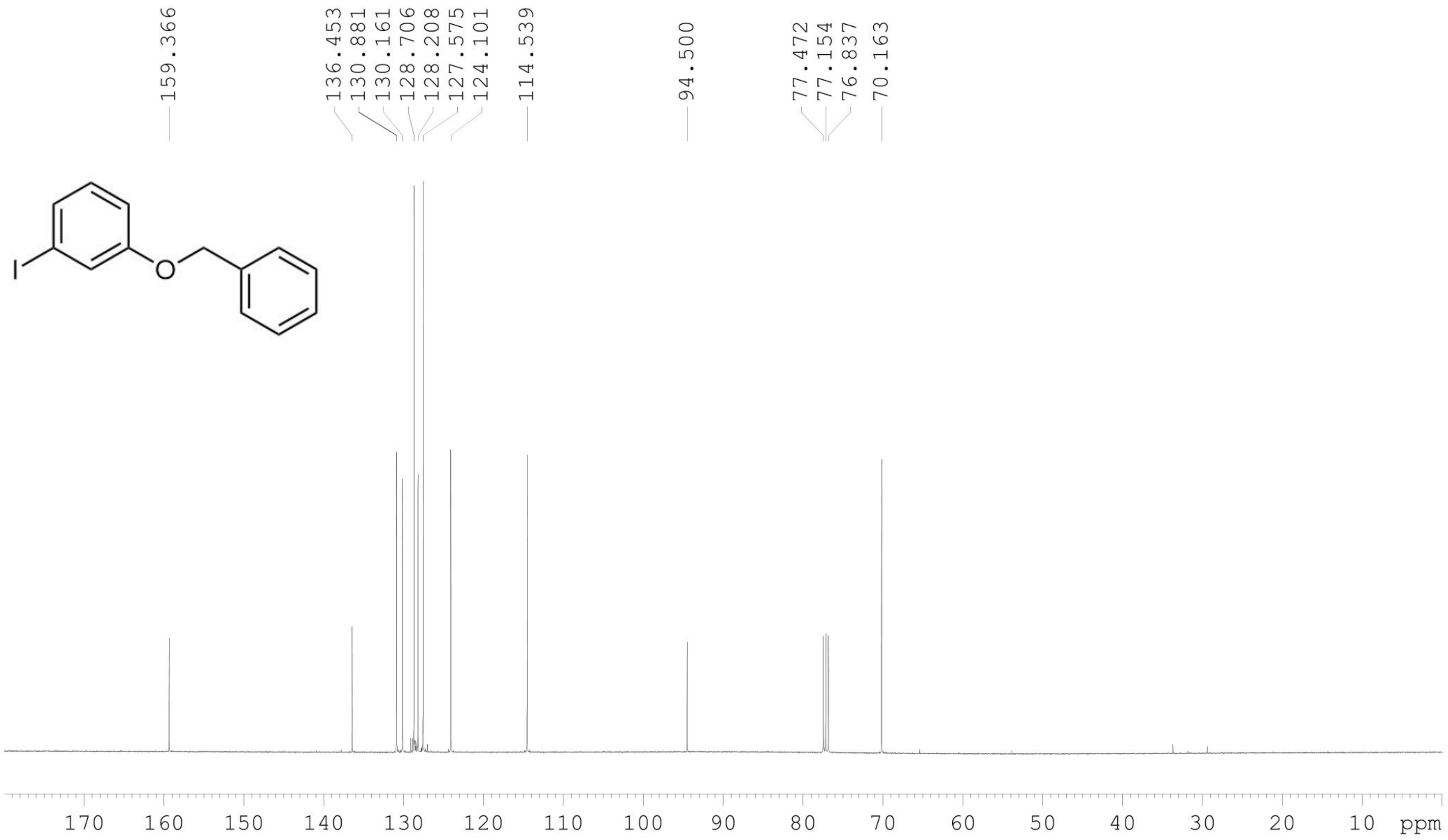

^{1}H NMR ($CDCl_3$, 400 MHz) of 53

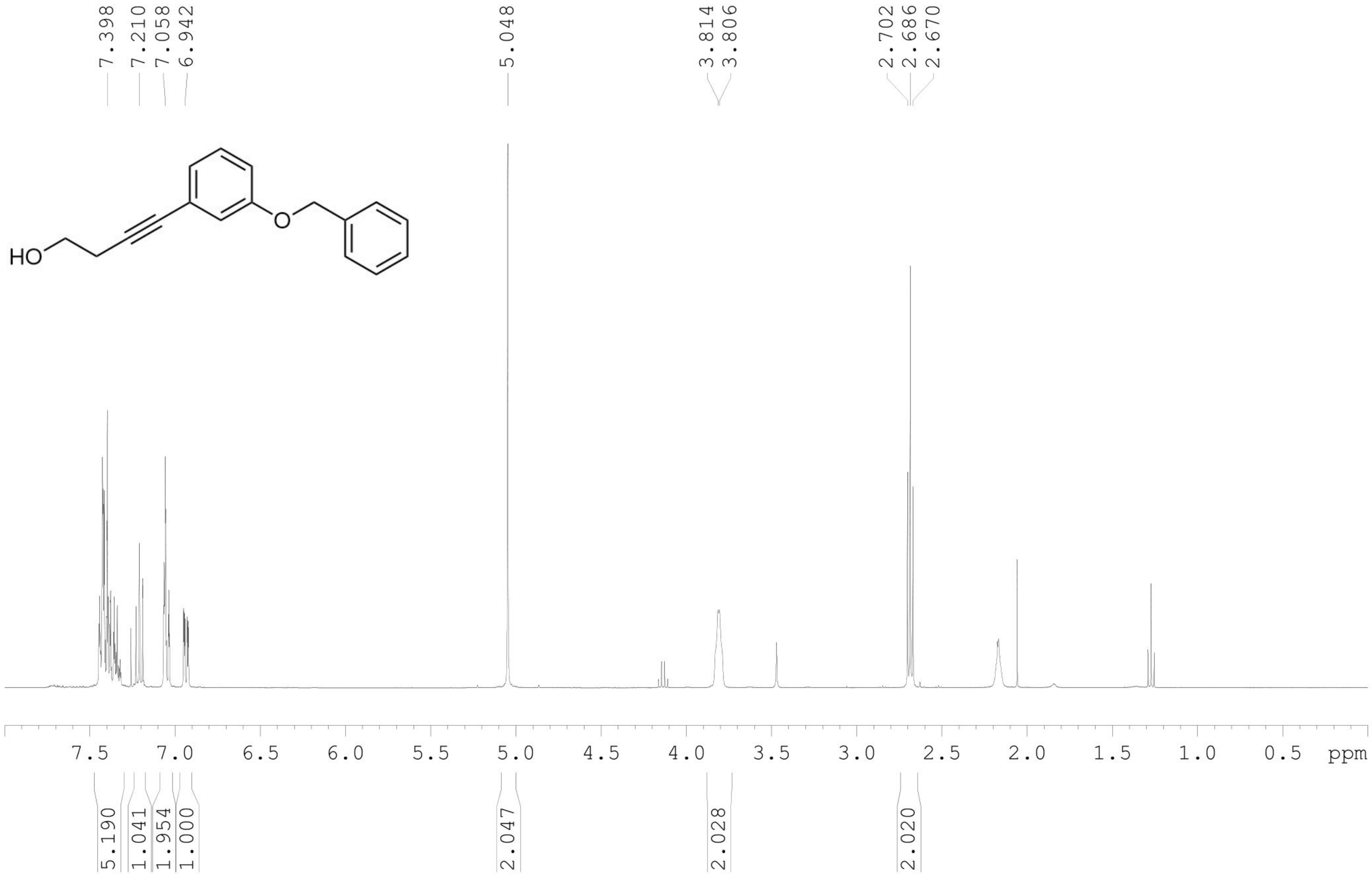

^{13}C NMR ($CDCl_3$, 100 MHz) of 53

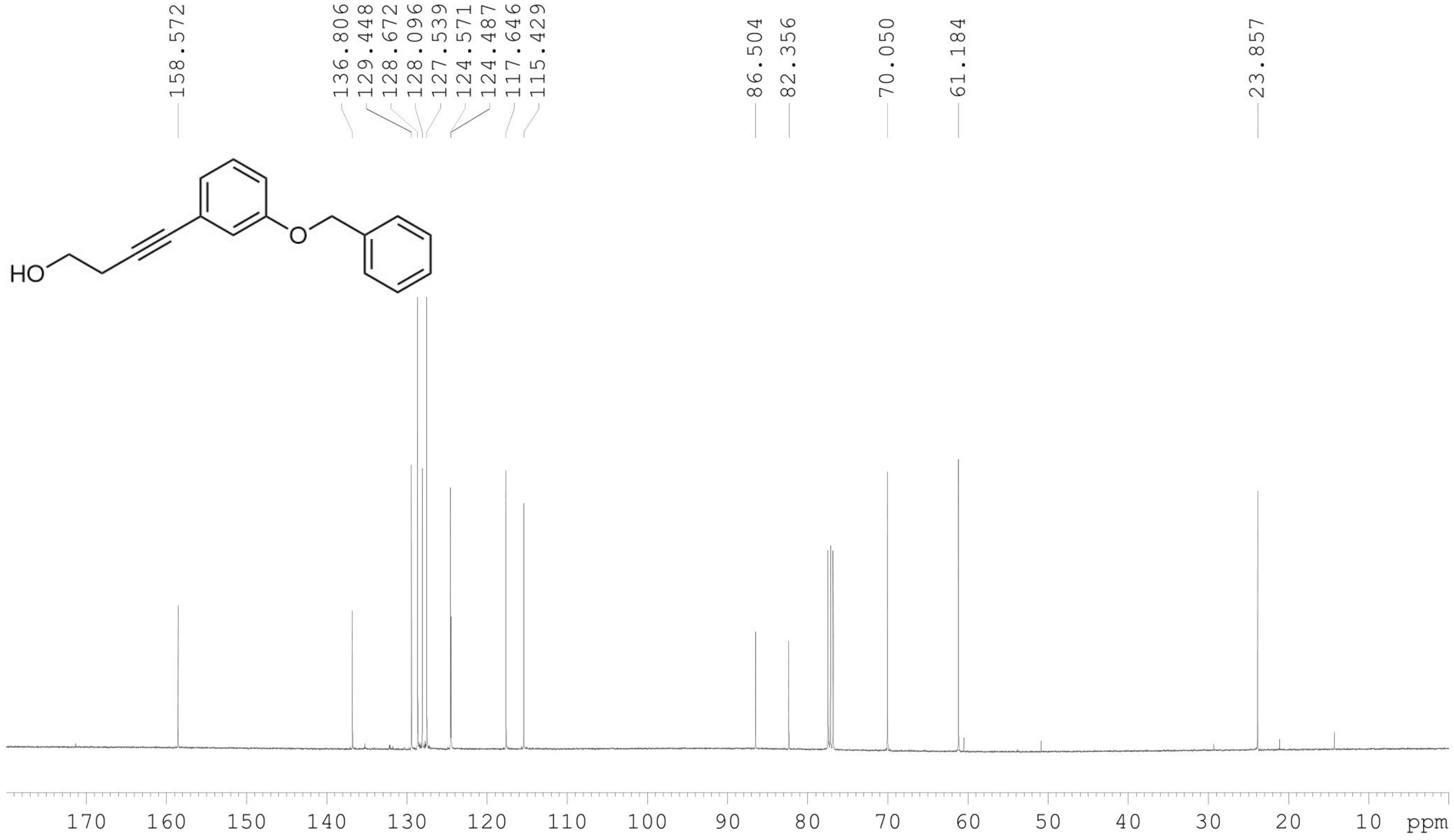

^{1}H NMR ($CDCl_3$, 400 MHz) of 54

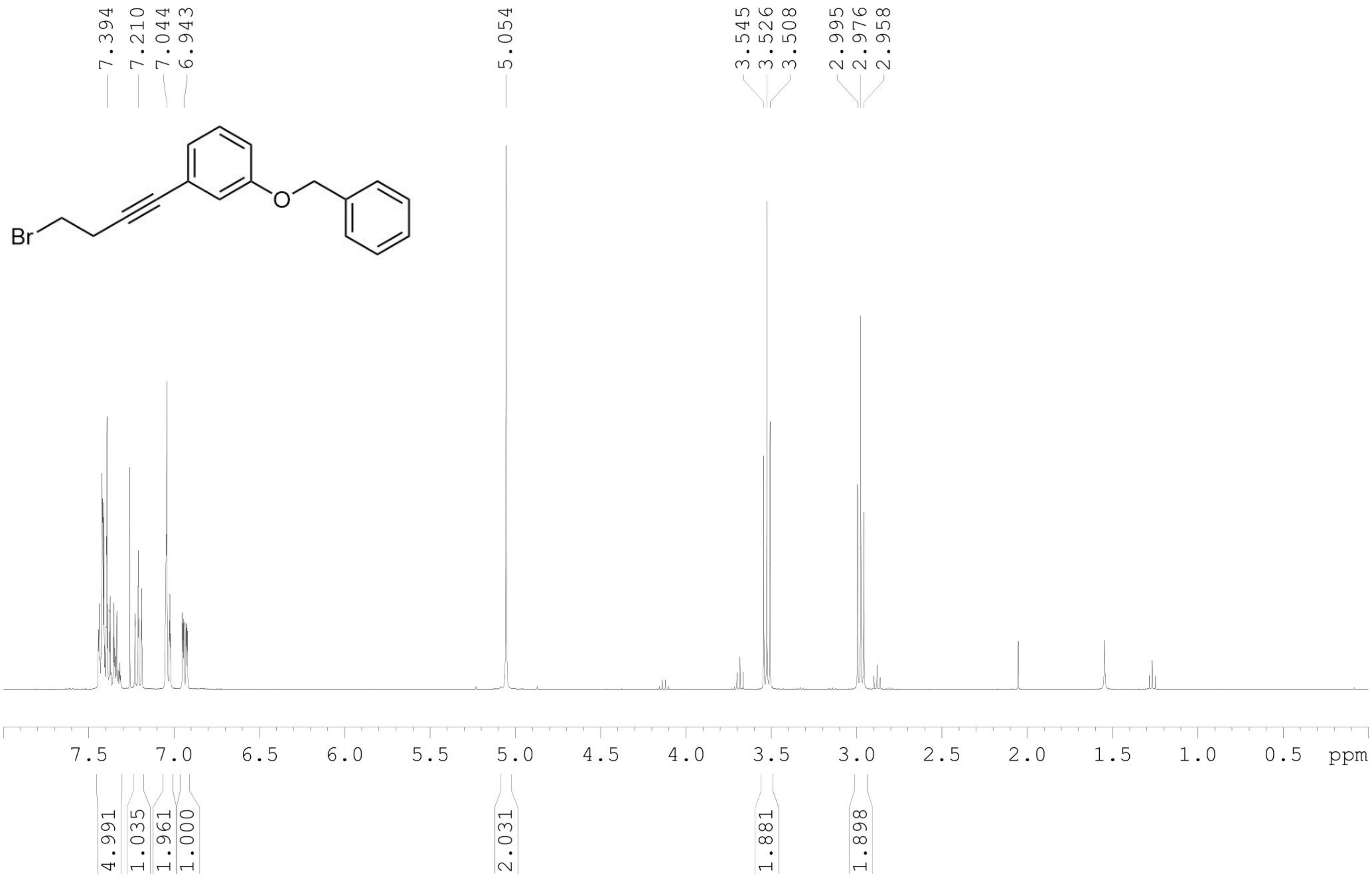

^{13}C NMR ($CDCl_3$, 100 MHz) of 54

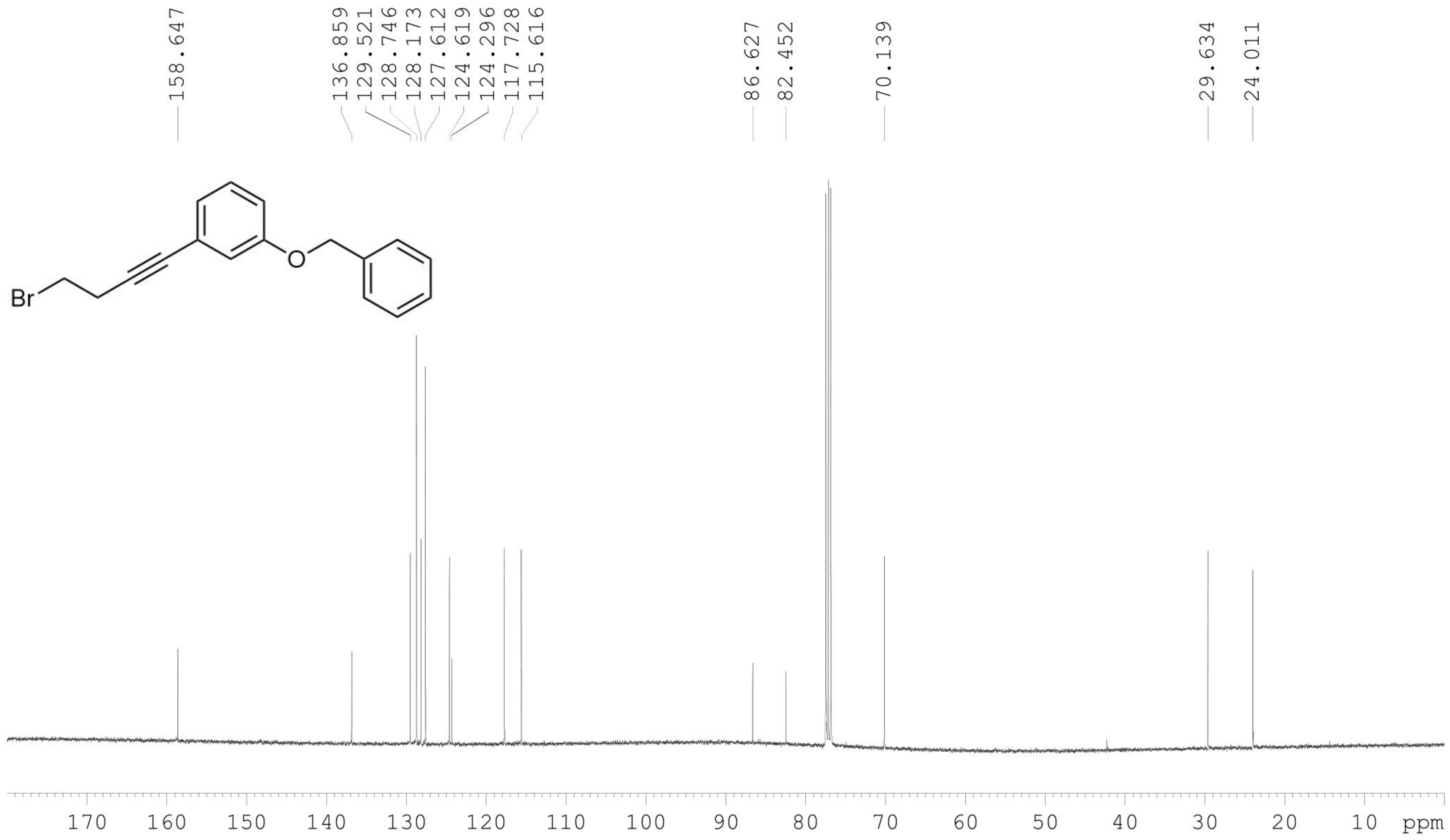

^{1}H NMR ($CDCl_3$, 400 MHz) of 55

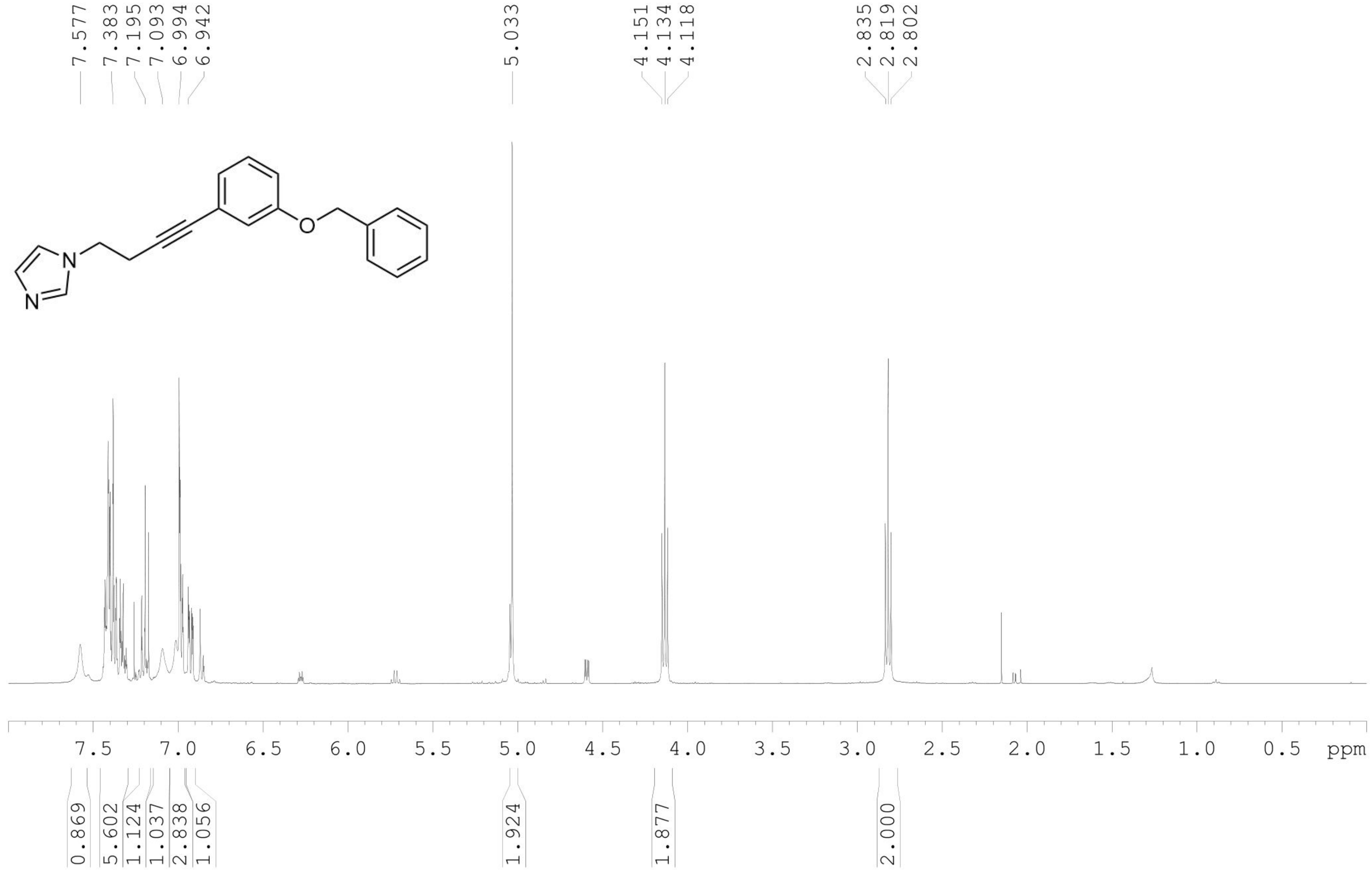

^{13}C NMR ($CDCl_3$, 100 MHz) of 55

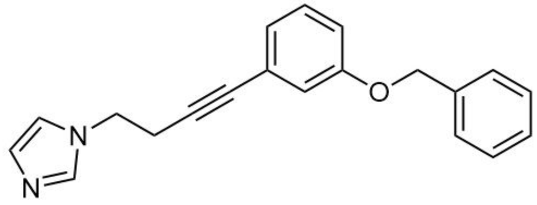

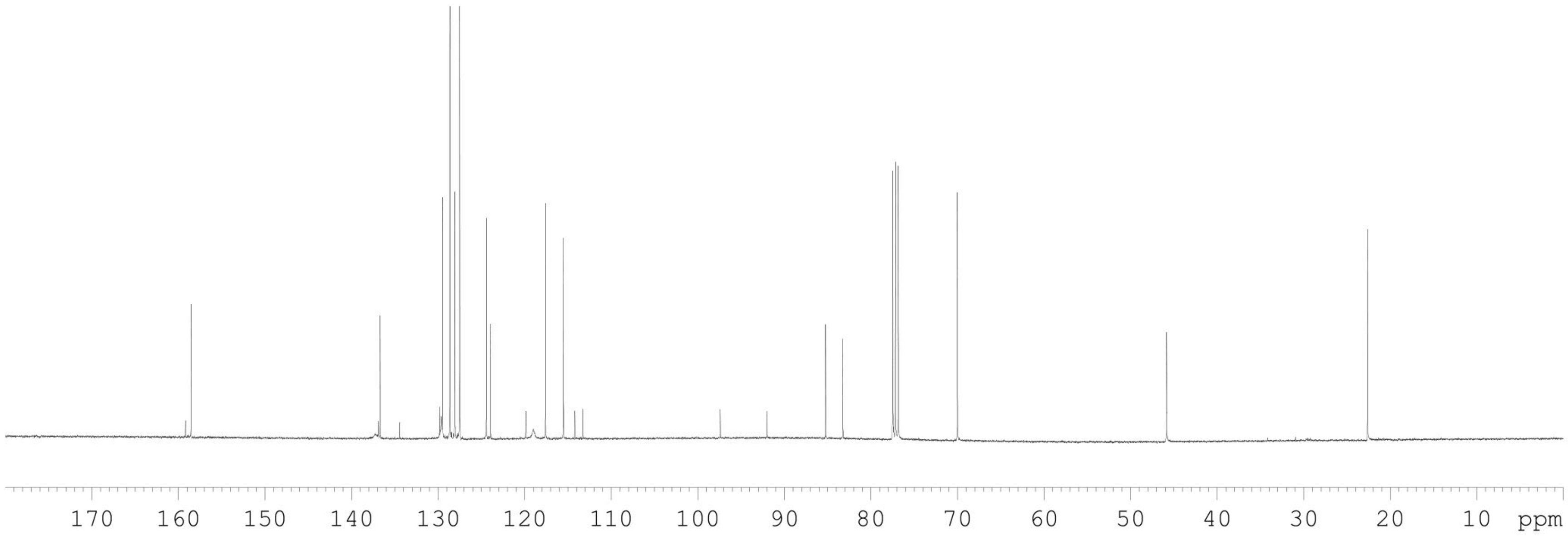

^{1}H NMR (DMSO, 400 MHz) of 56

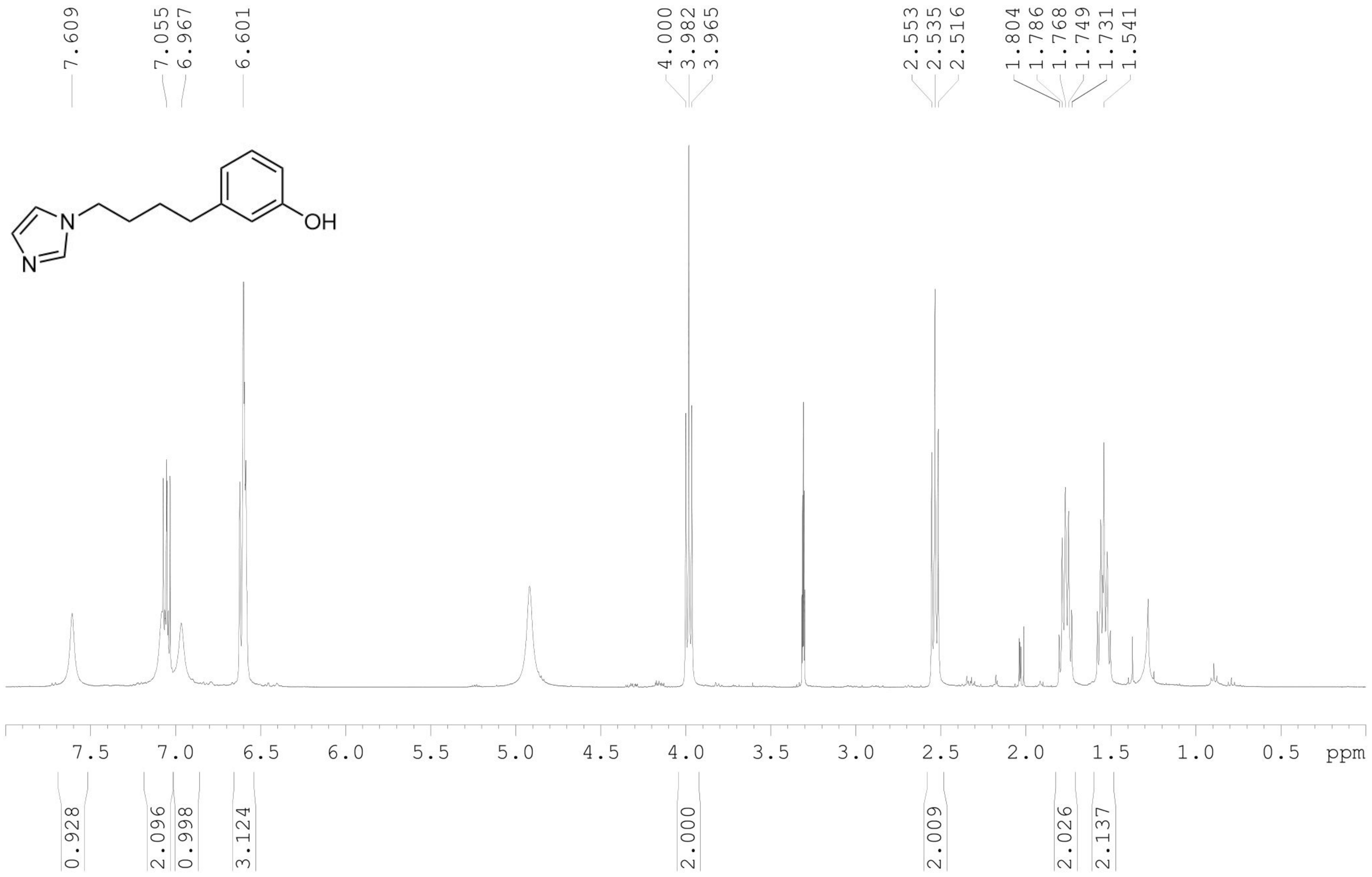

^{13}C NMR (DMSO, 100 MHz) of 56

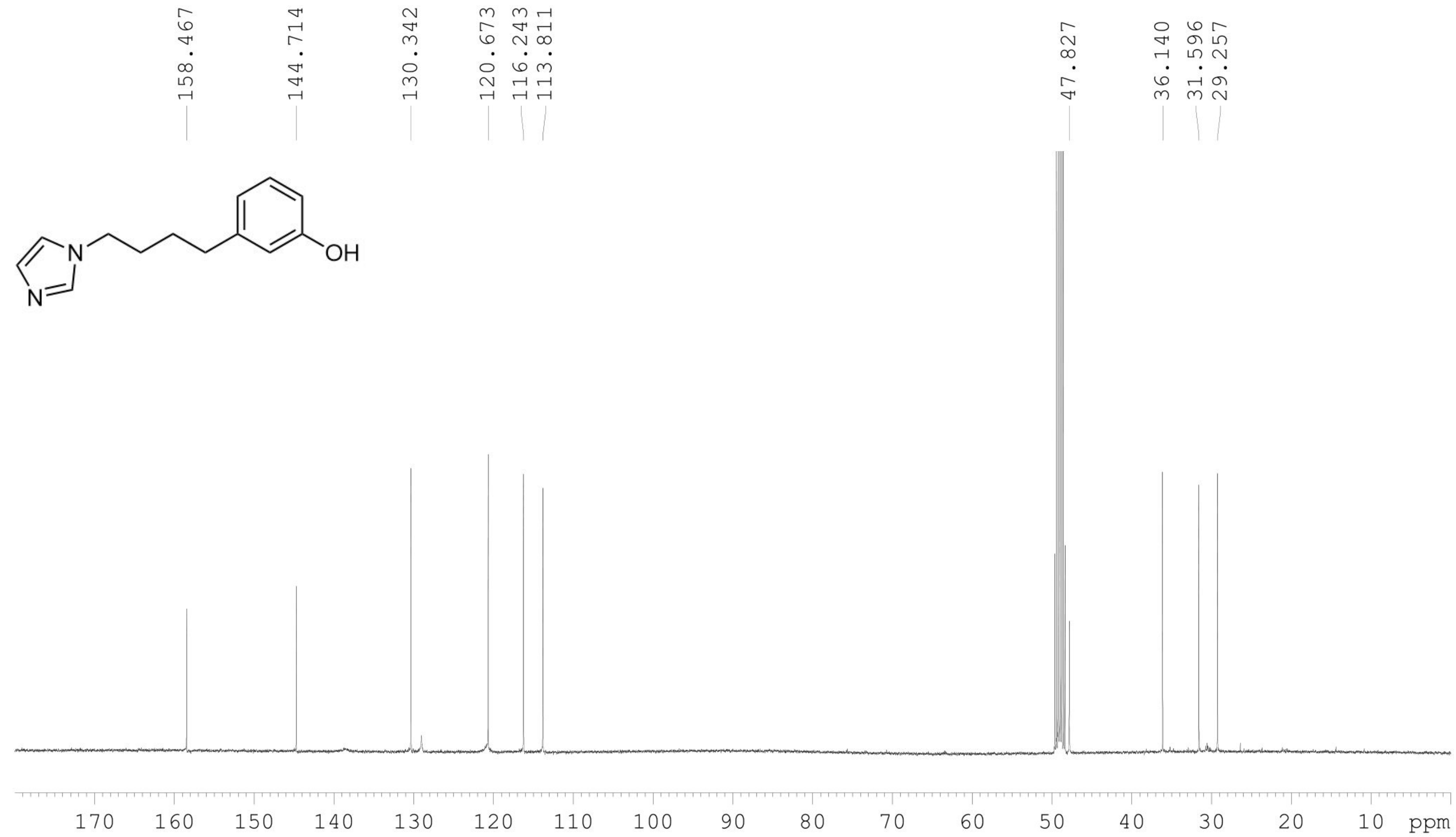

^{1}H NMR ($CDCl_3$, 400 MHz) of 57

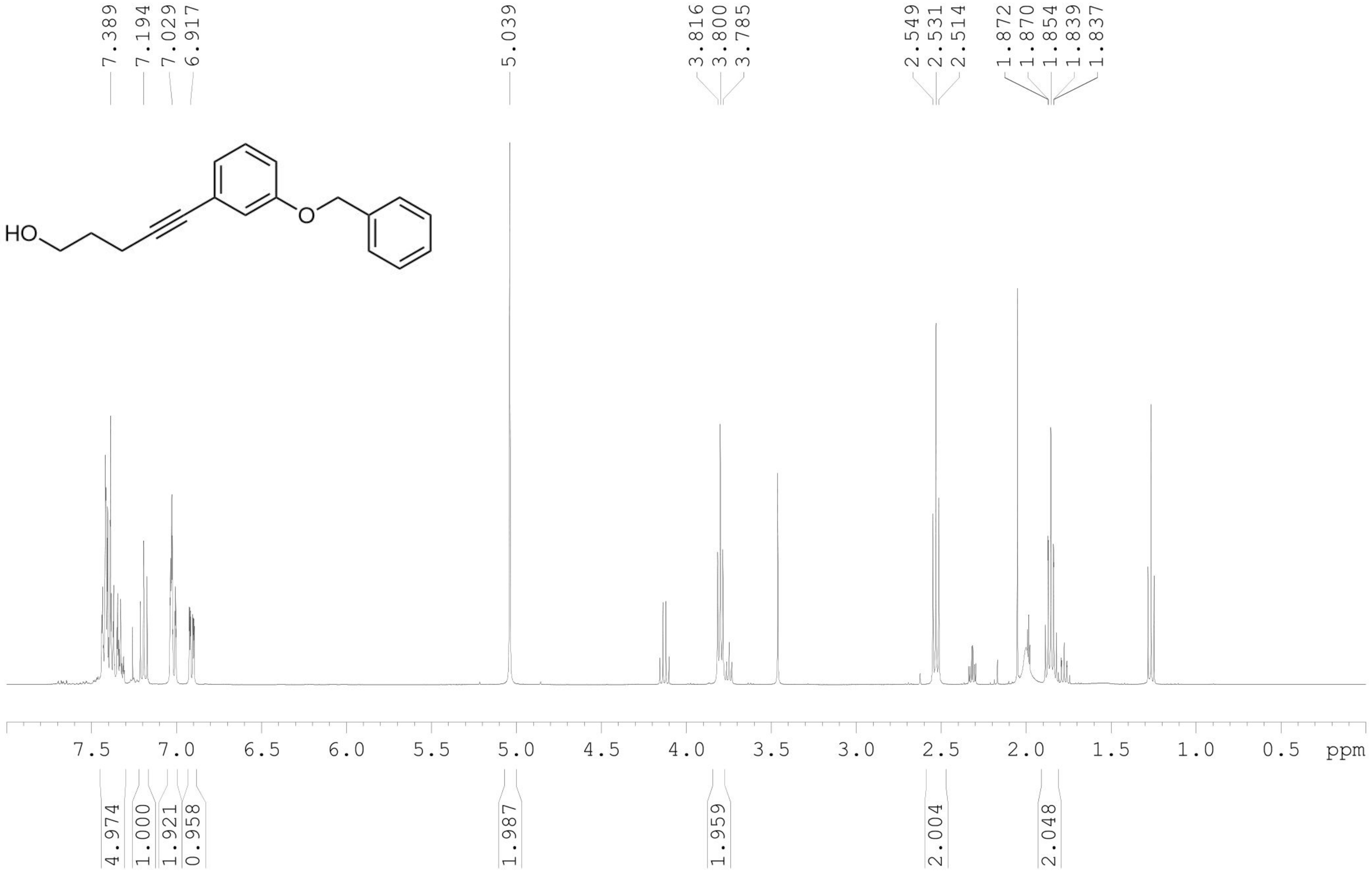

^{13}C NMR ($CDCl_3$, 100 MHz) of 57

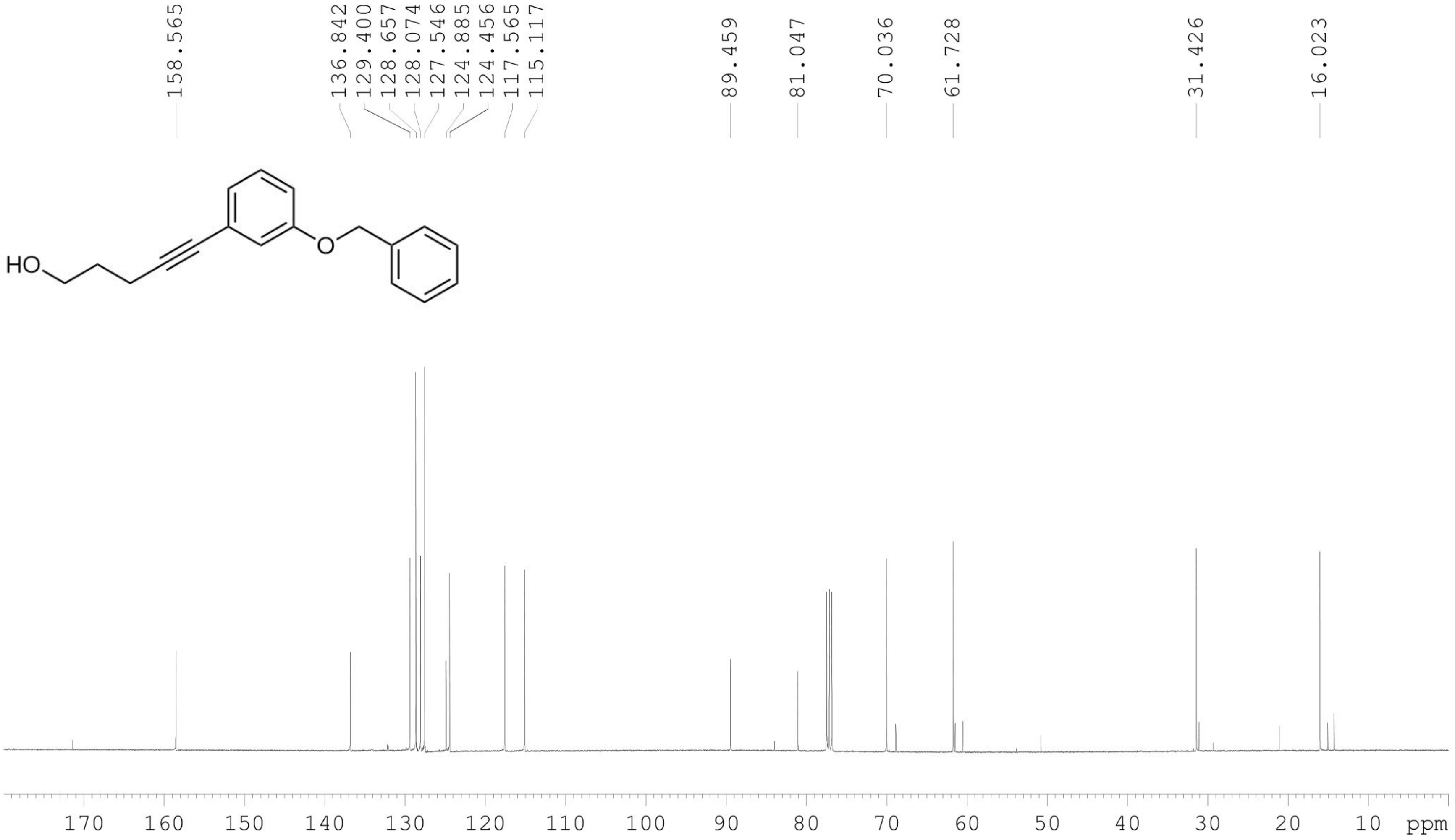

^{1}H NMR ($CDCl_3$, 400 MHz) of 58

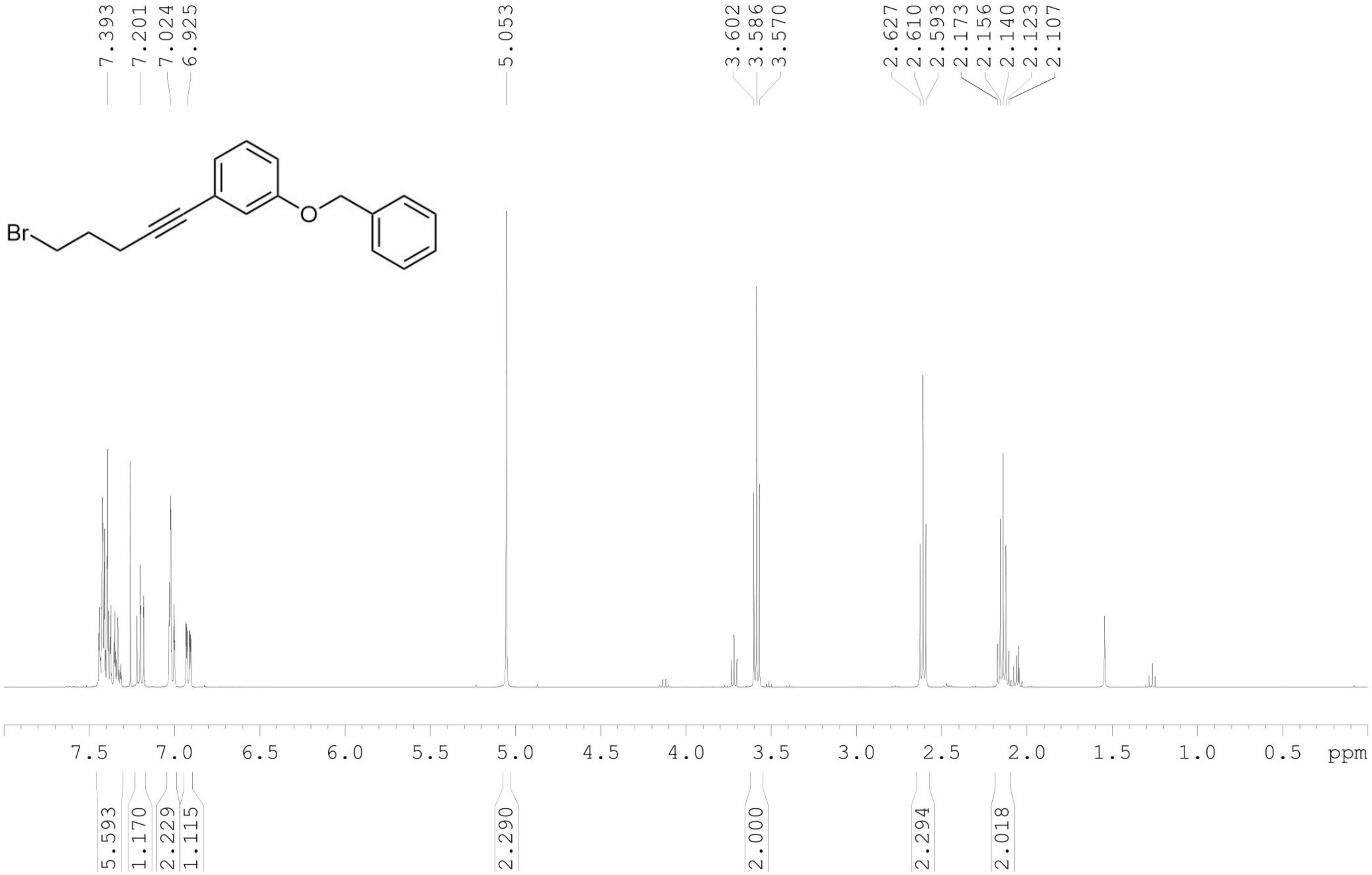

^{13}C NMR ($CDCl_3$, 100 MHz) of 58

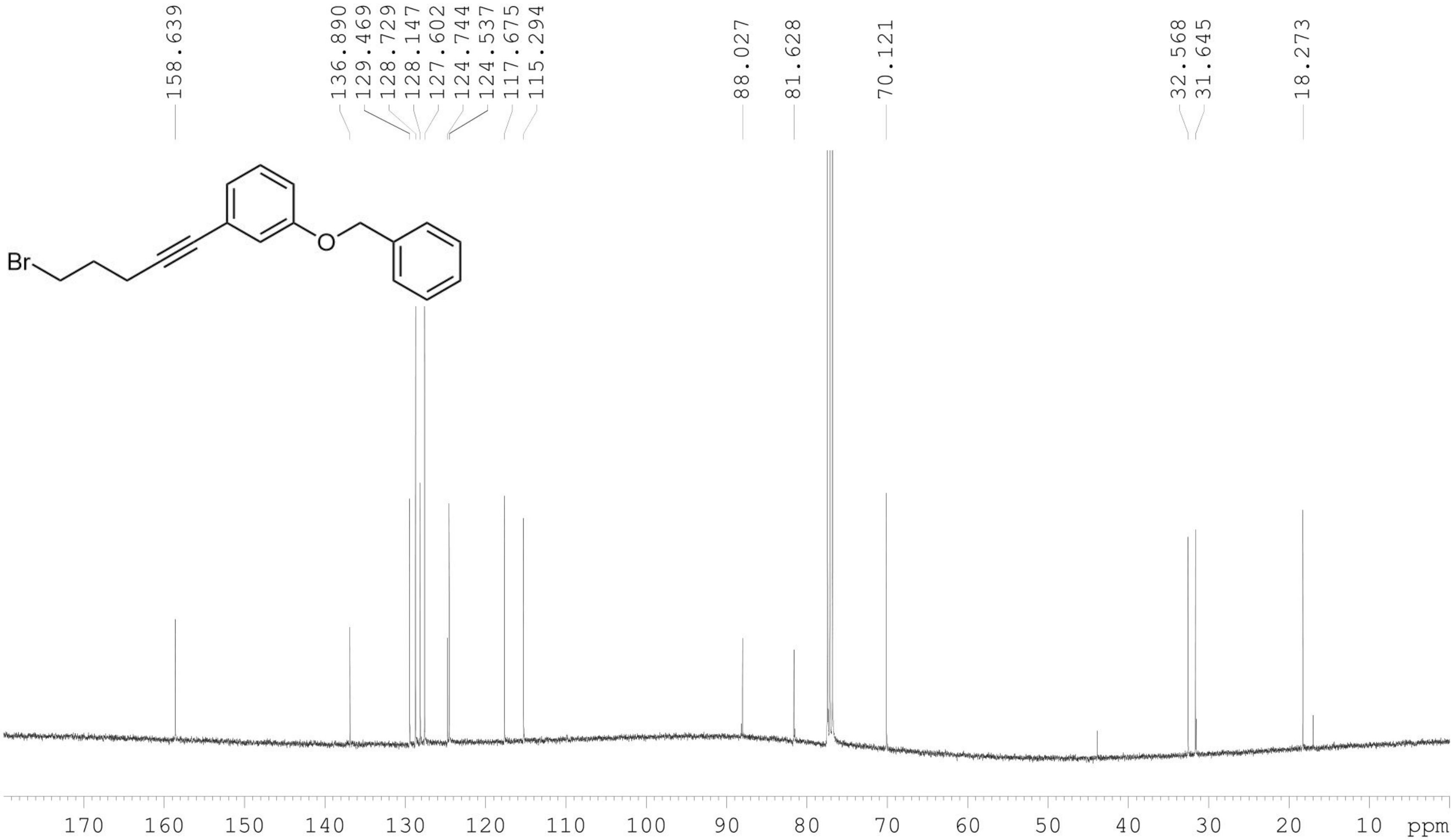

^{1}H NMR ($CDCl_3$, 400 MHz) of 59

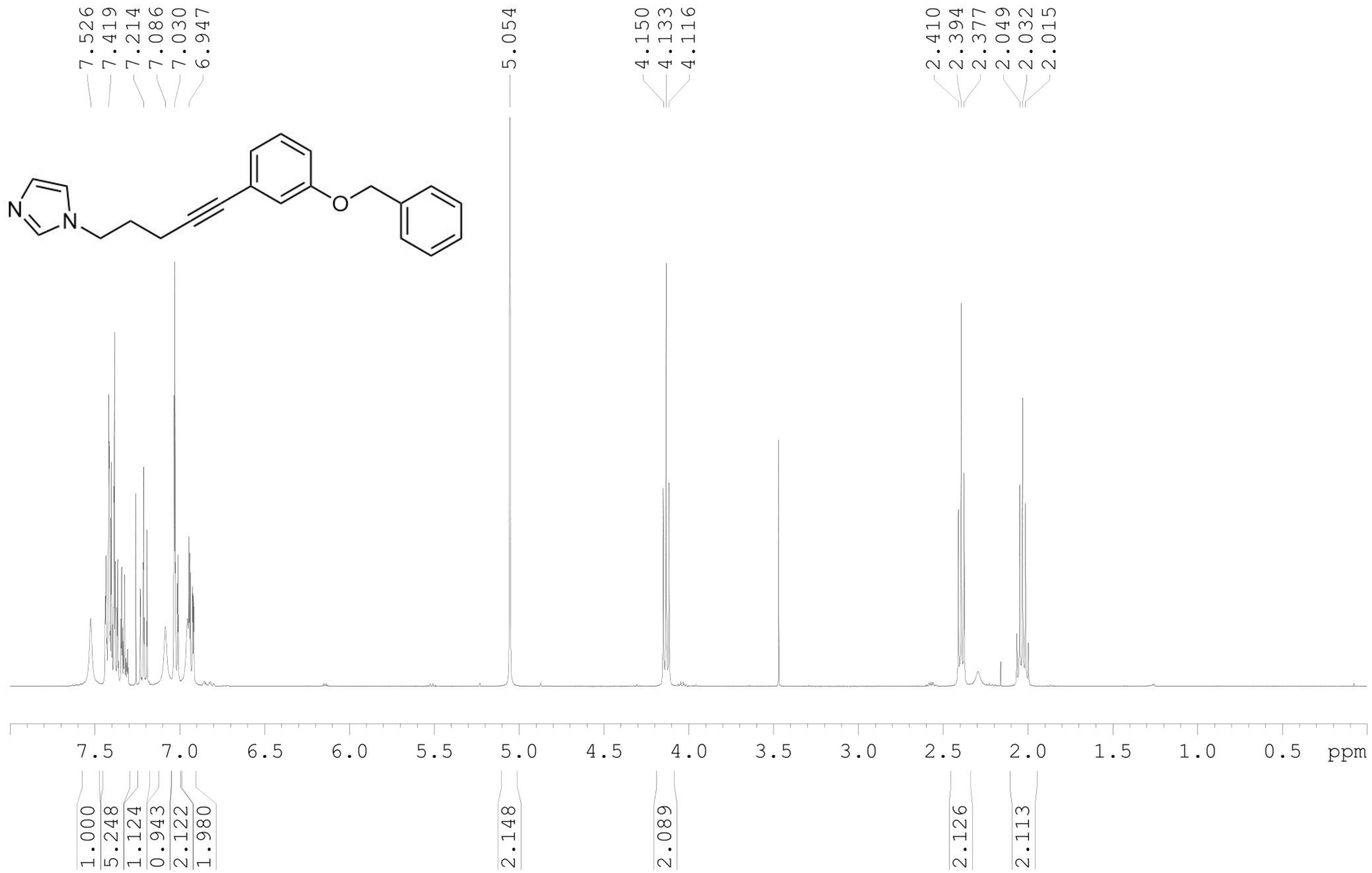

^{13}C NMR ($CDCl_3$, 100 MHz) of 59

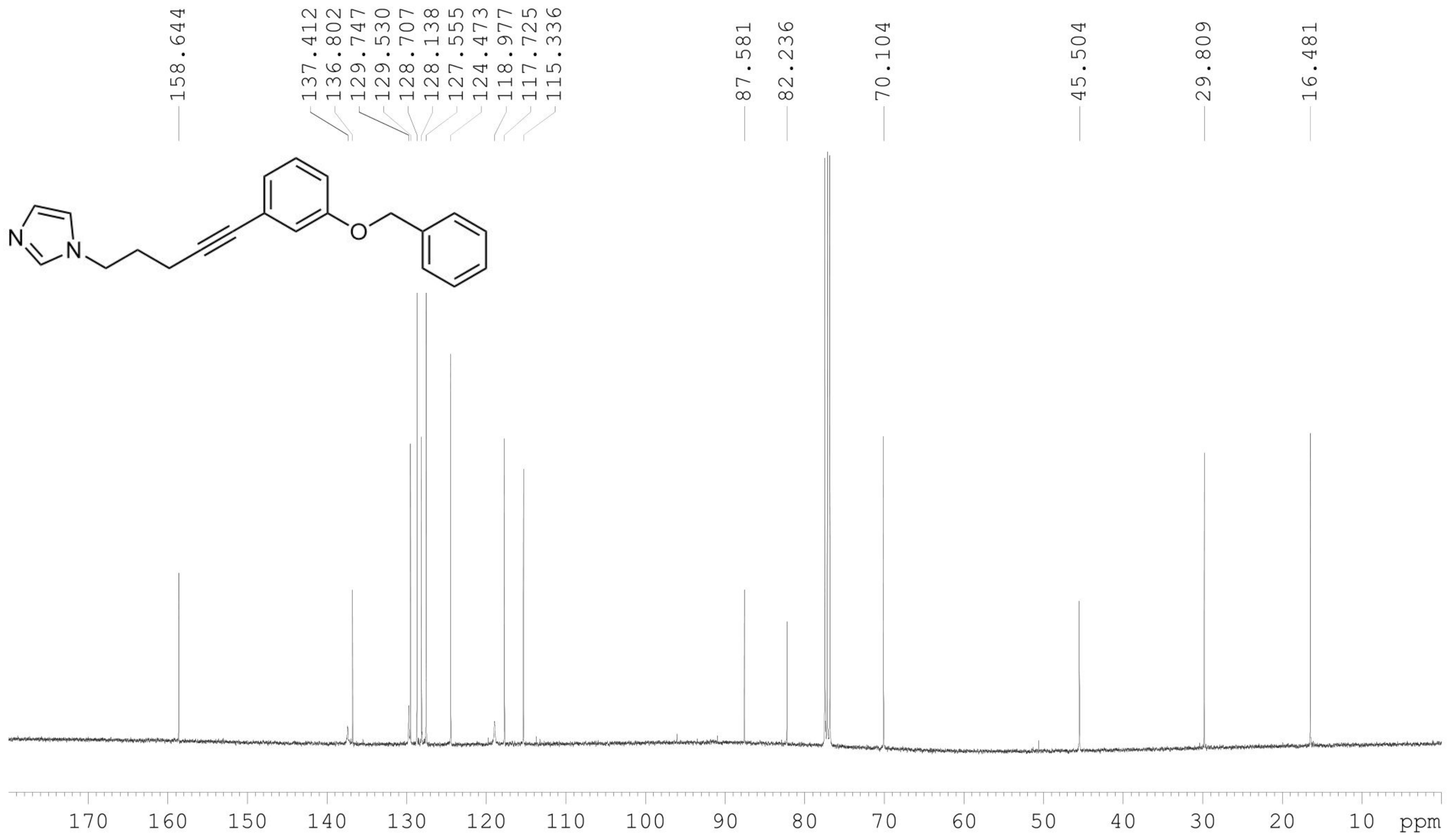

^{1}H NMR (DMSO, 400 MHz) of 60

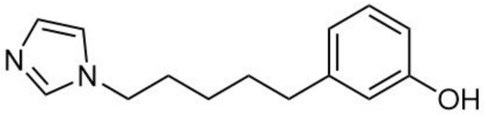

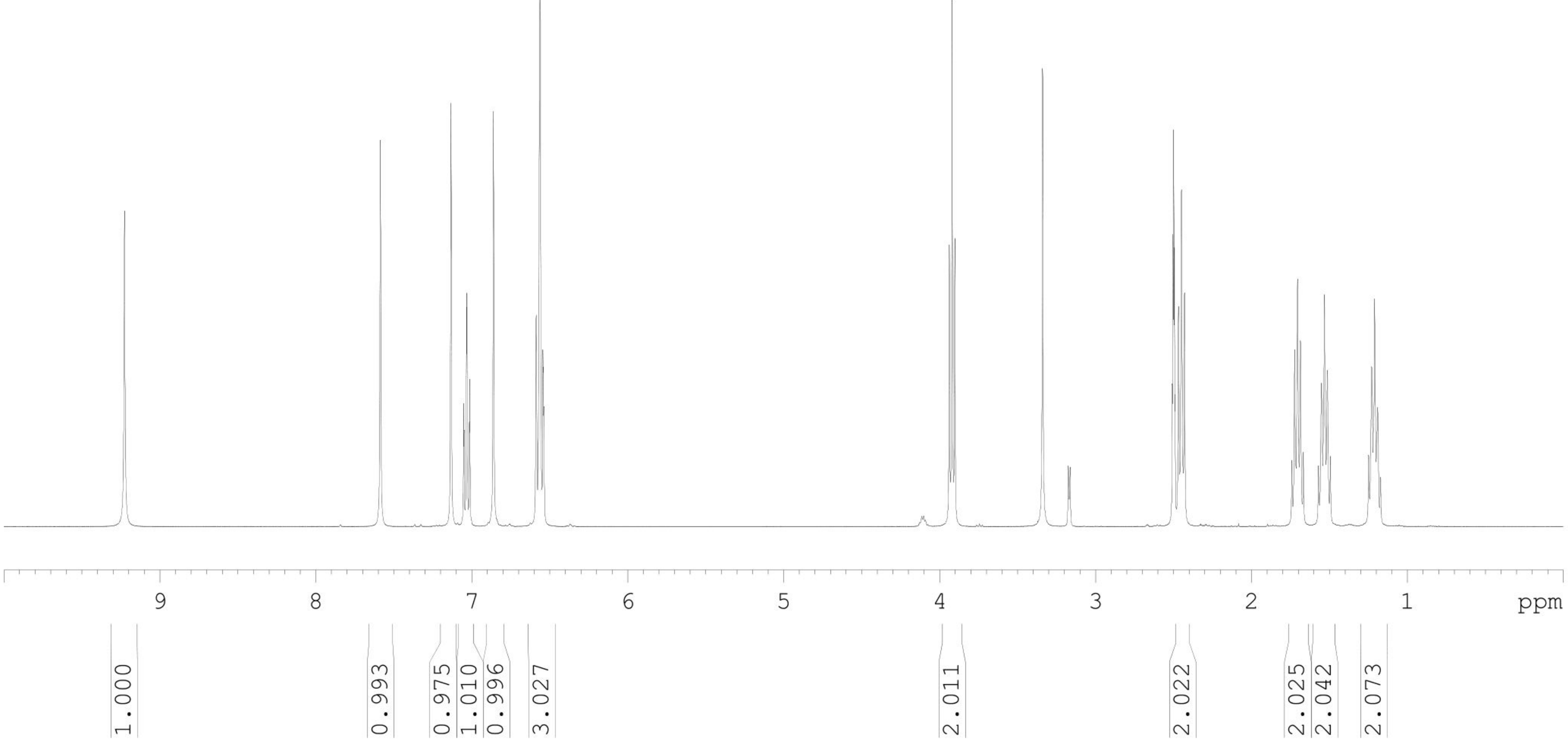

^{13}C NMR (DMSO, 100 MHz) of 60

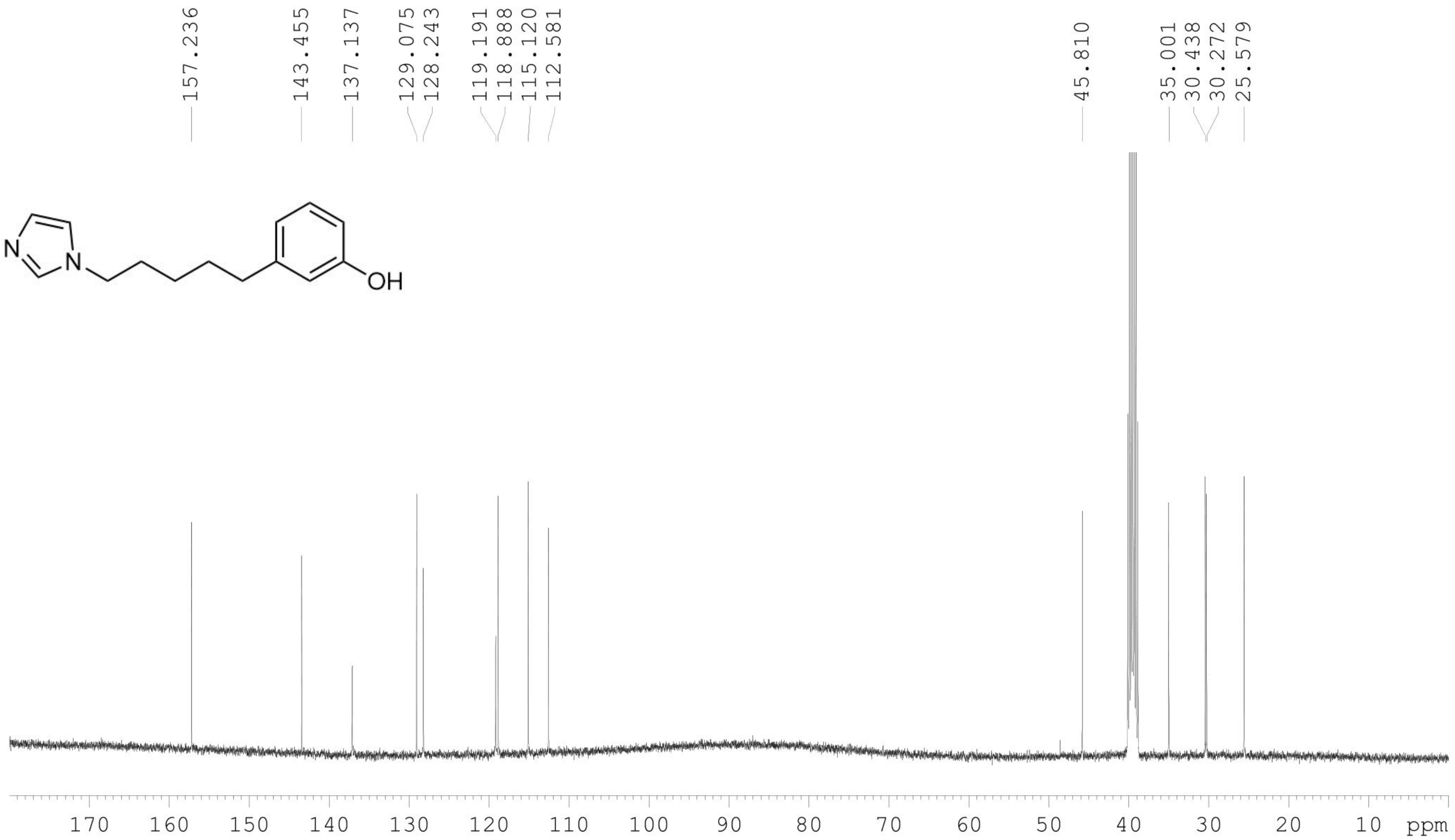

^{1}H NMR ($CDCl_3$, 400 MHz) of 61

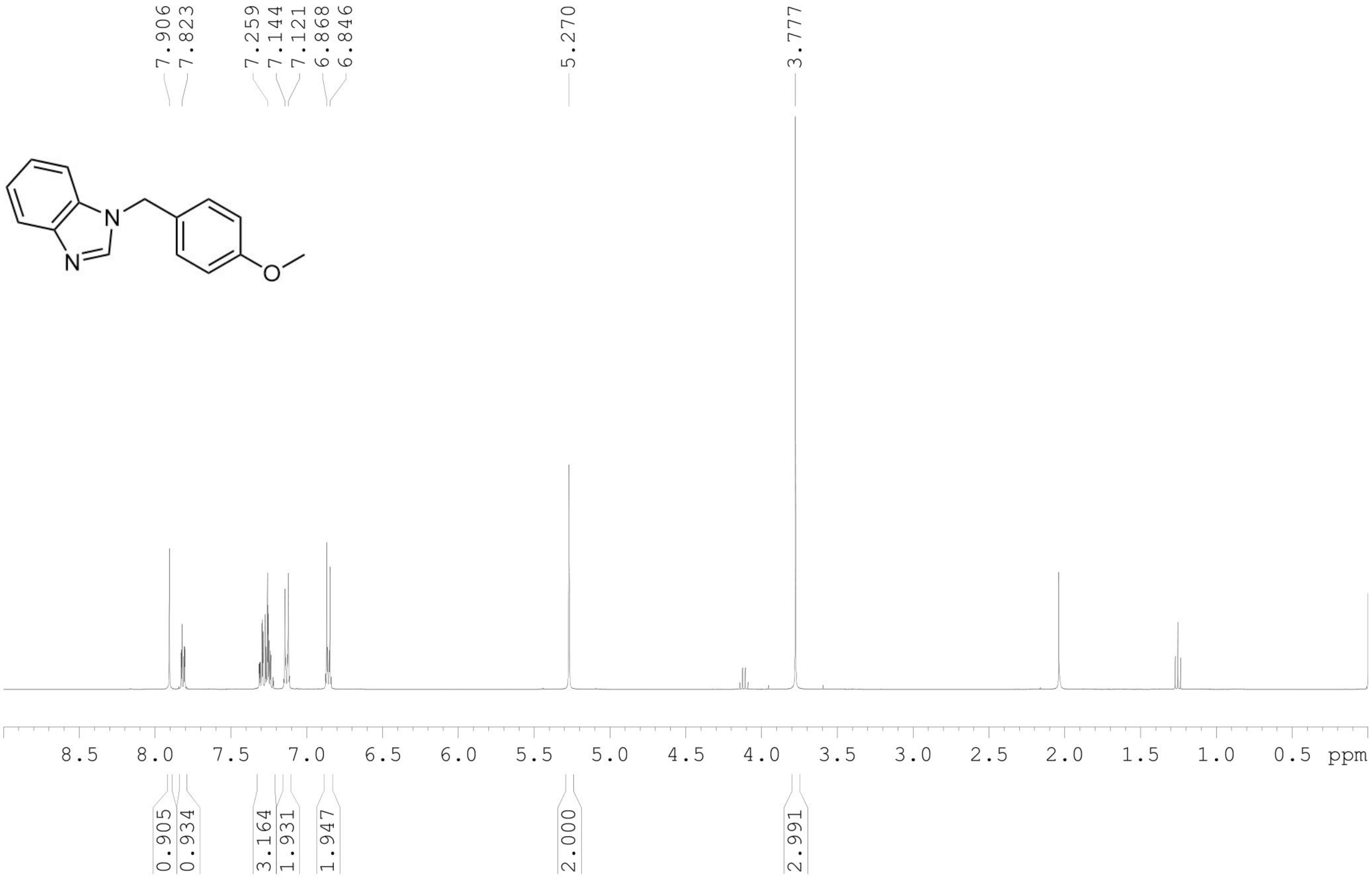

^{13}C NMR ($CDCl_3$, 100 MHz) of 61

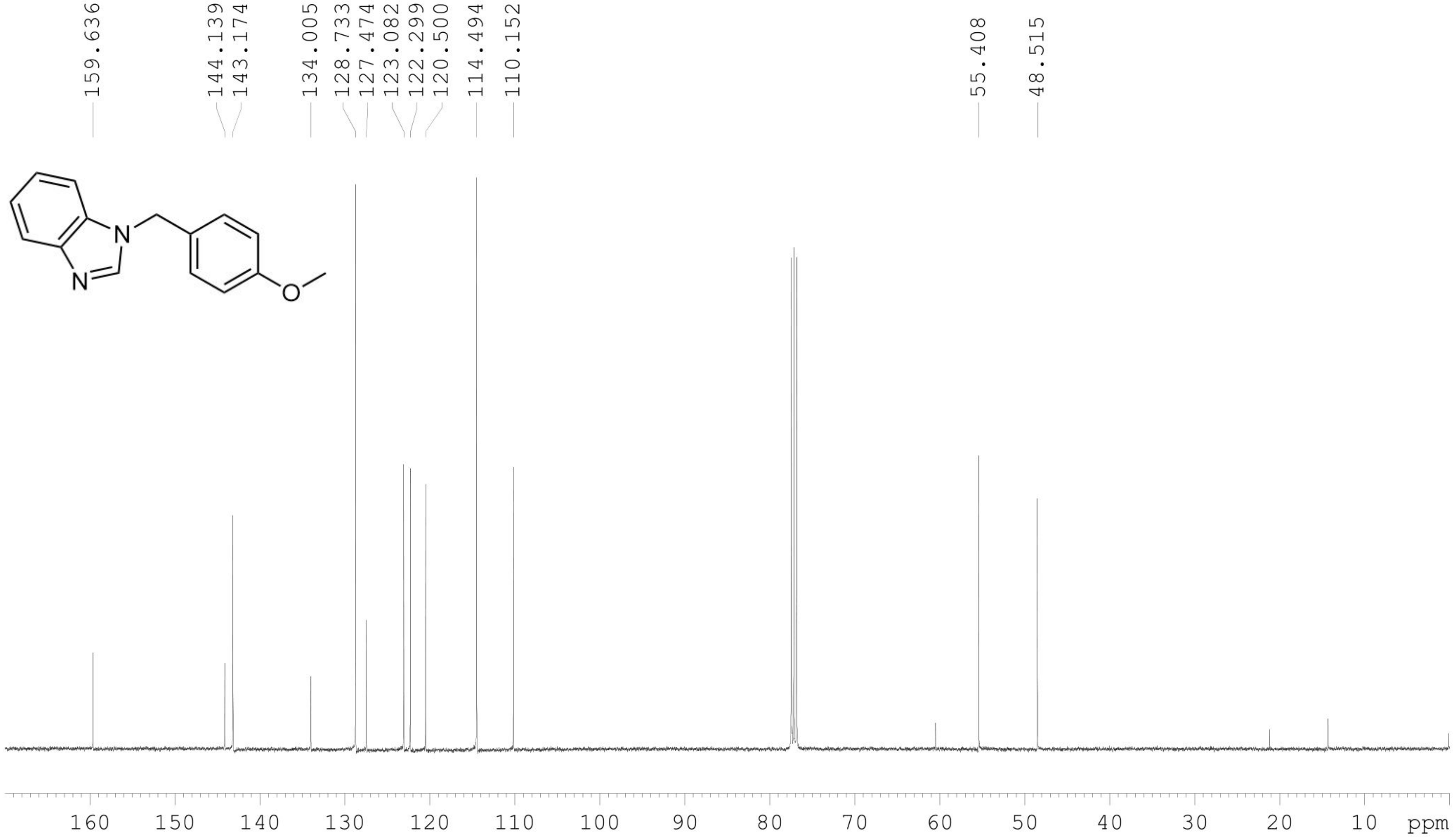

^{1}H NMR (DMSO, 400 MHz) of 62

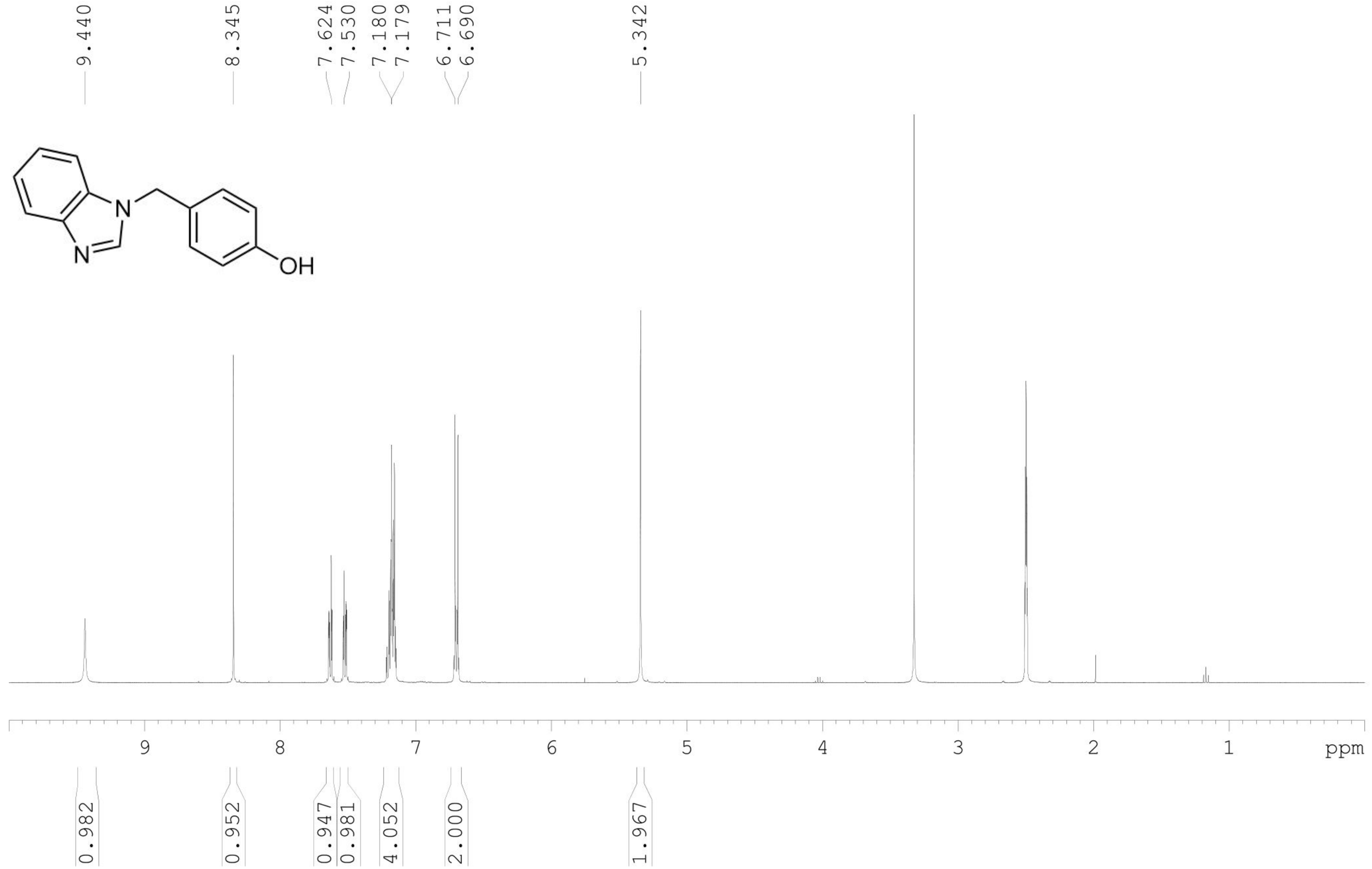

^{13}C NMR (DMSO, 100 MHz) of 62

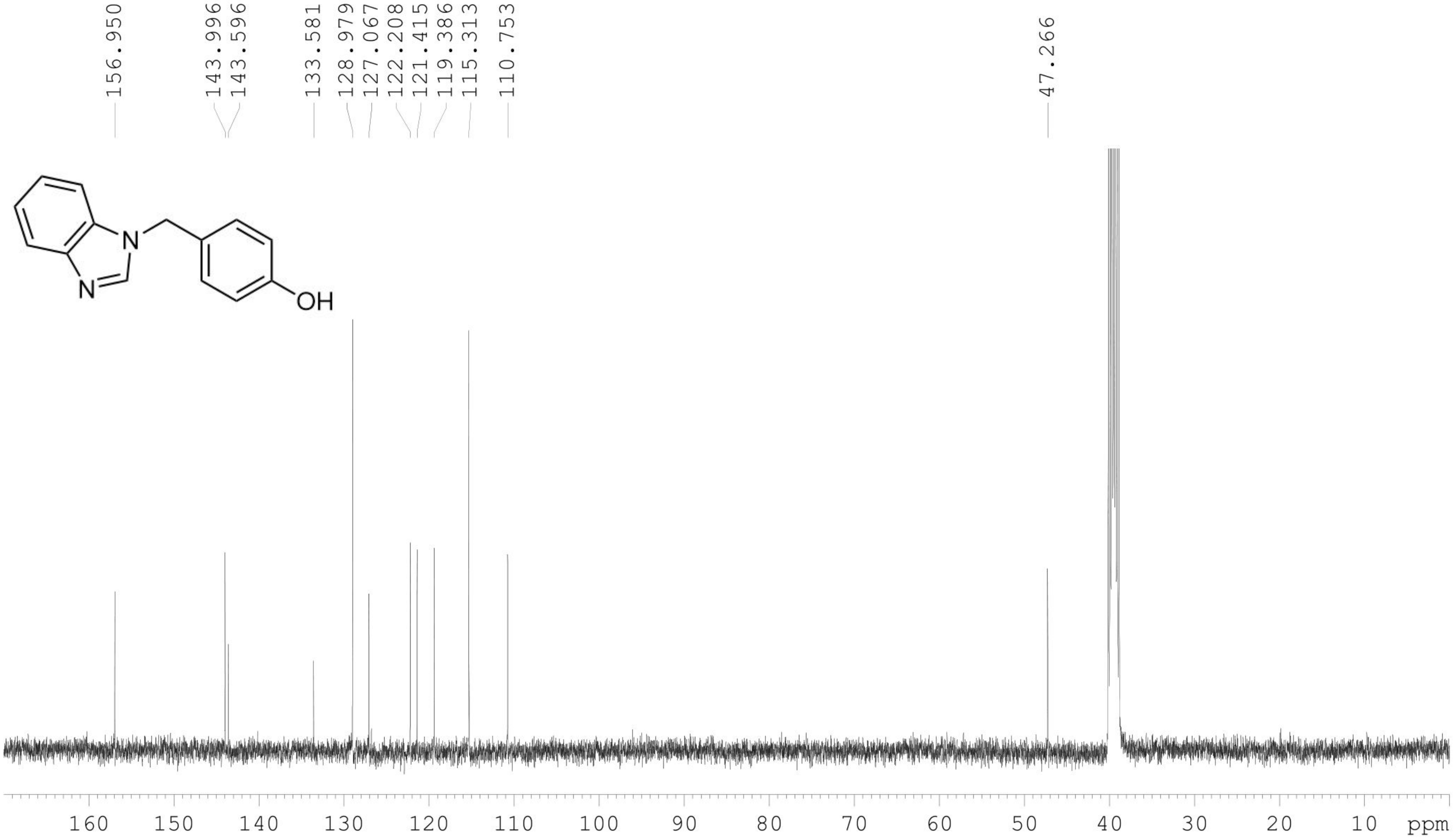

^{1}H NMR (DMSO, 400 MHz) of 63

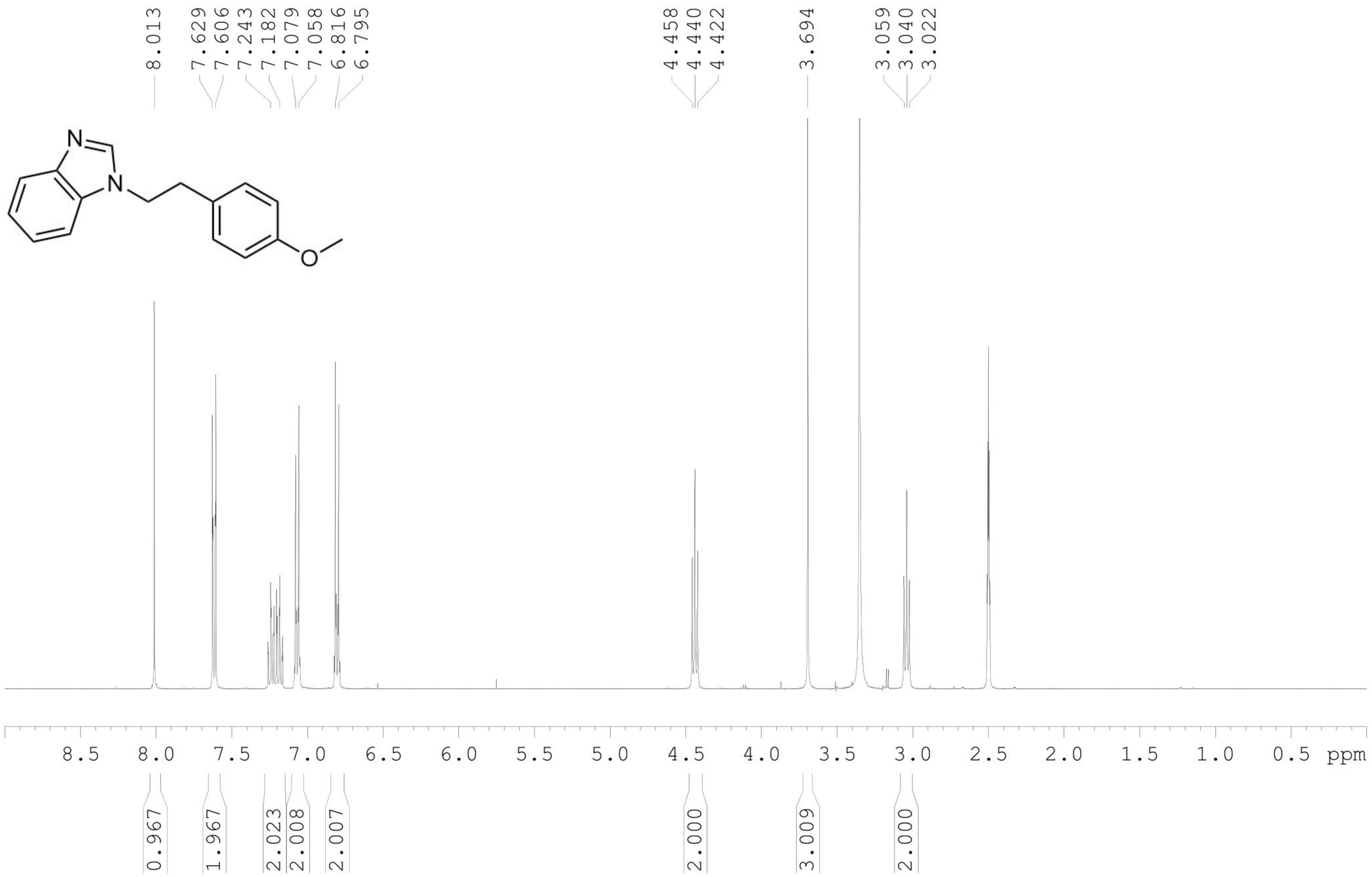

^{13}C NMR (DMSO, 100 MHz) of 63

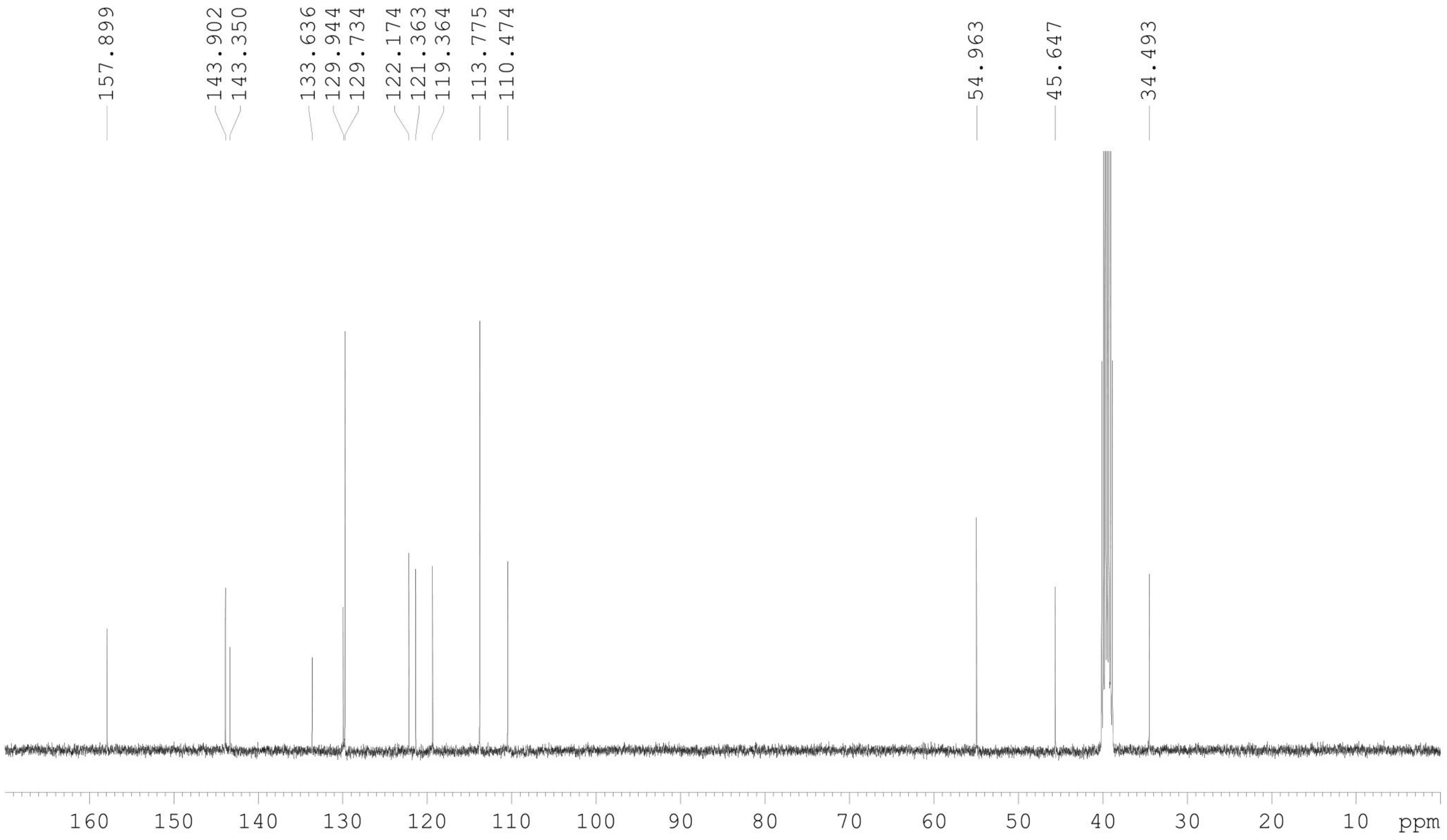

^{1}H NMR (DMSO, 400 MHz) of 64

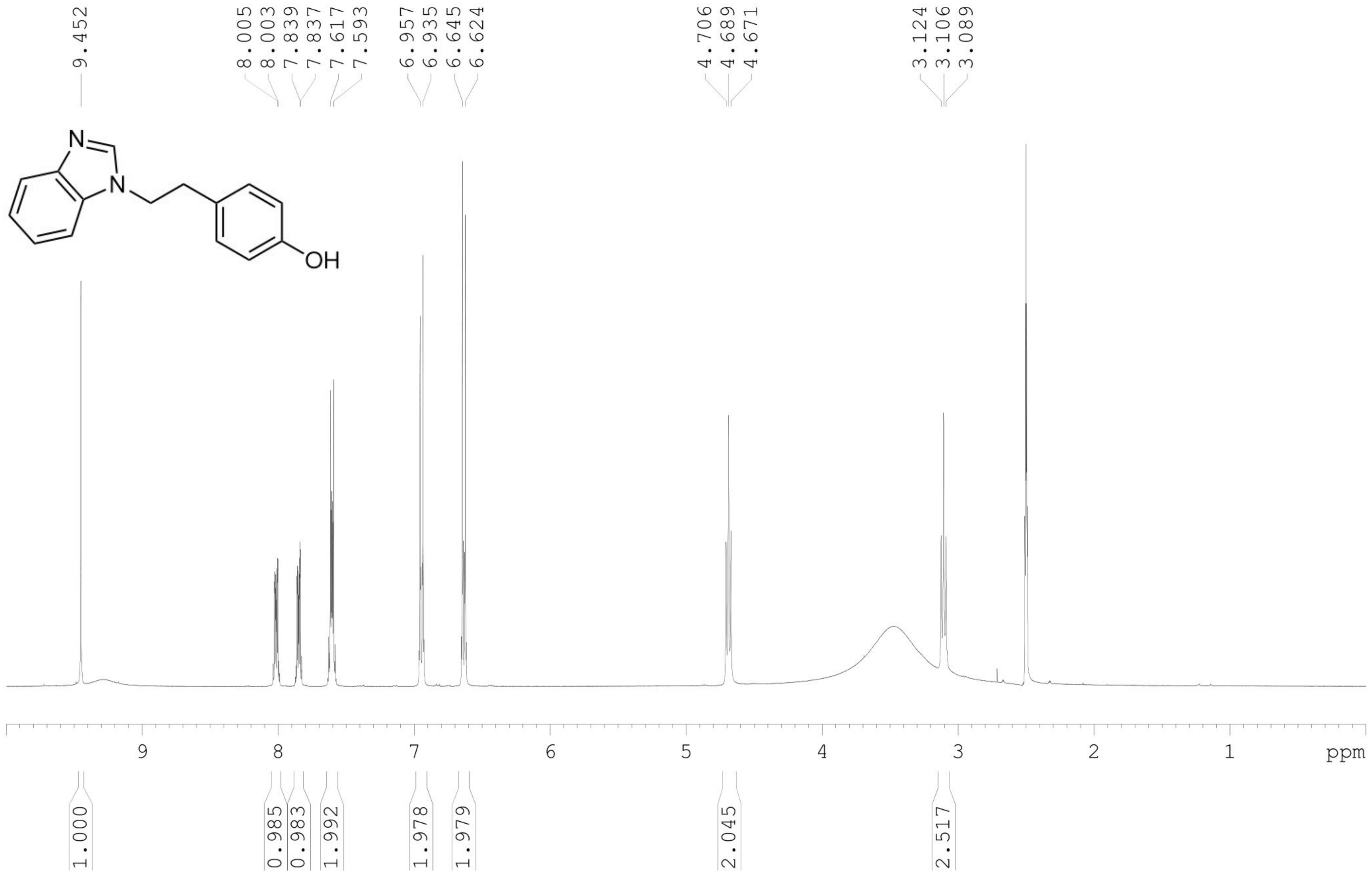

^{13}C NMR (DMSO, 100 MHz) of 64

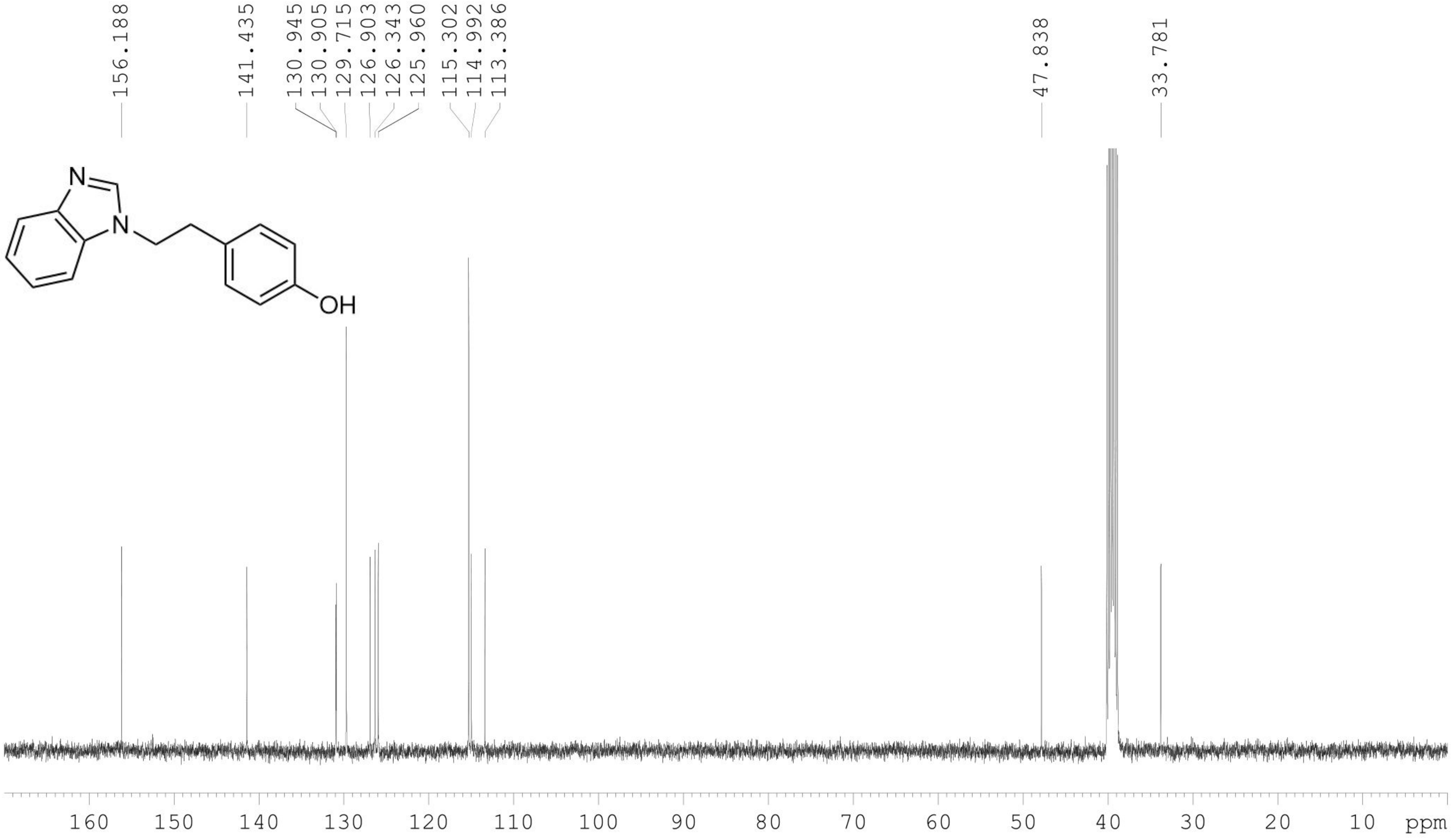

^{1}H NMR (DMSO, 400 MHz) of 65

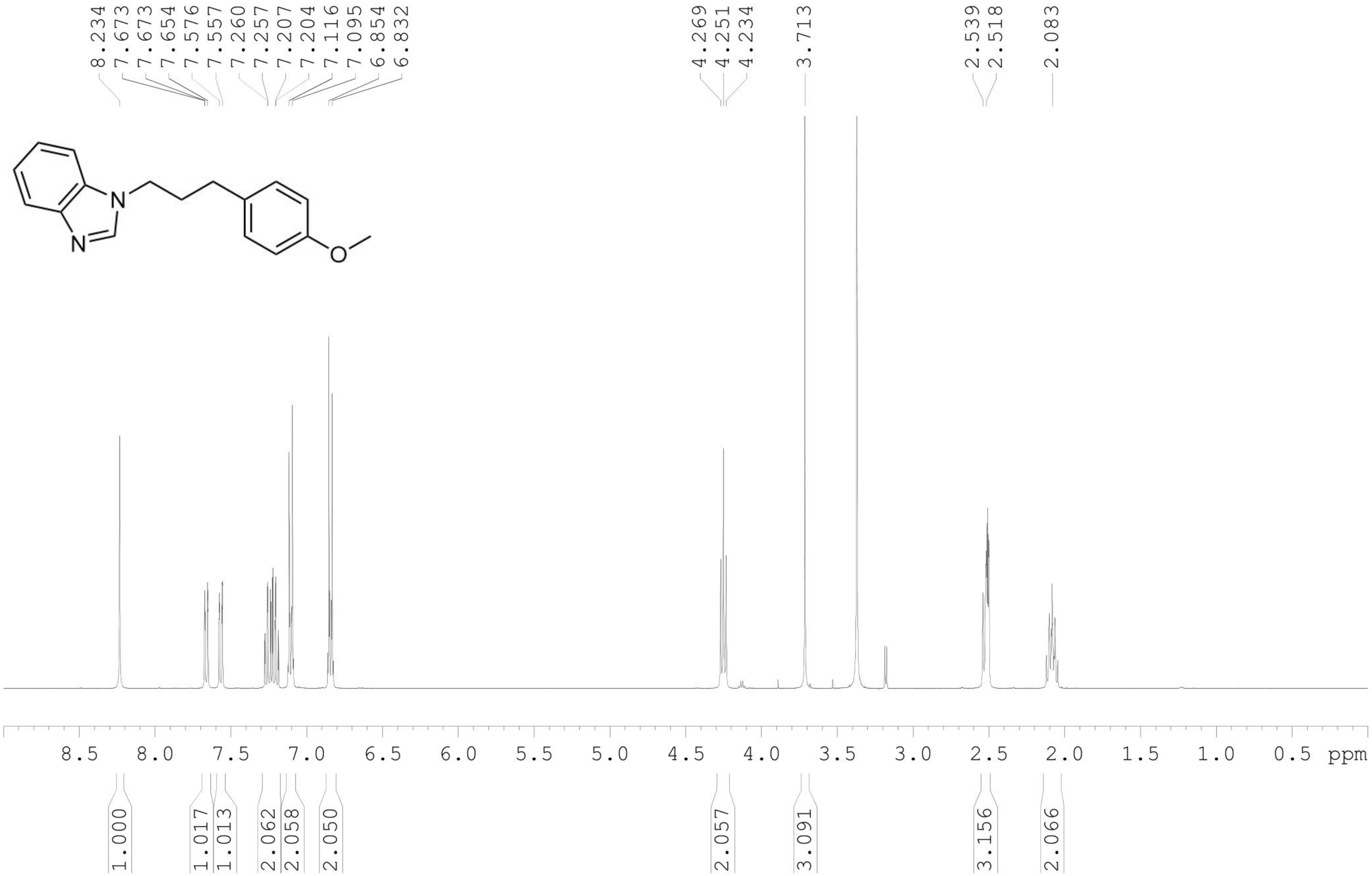

^{13}C NMR (DMSO, 100 MHz) of 65

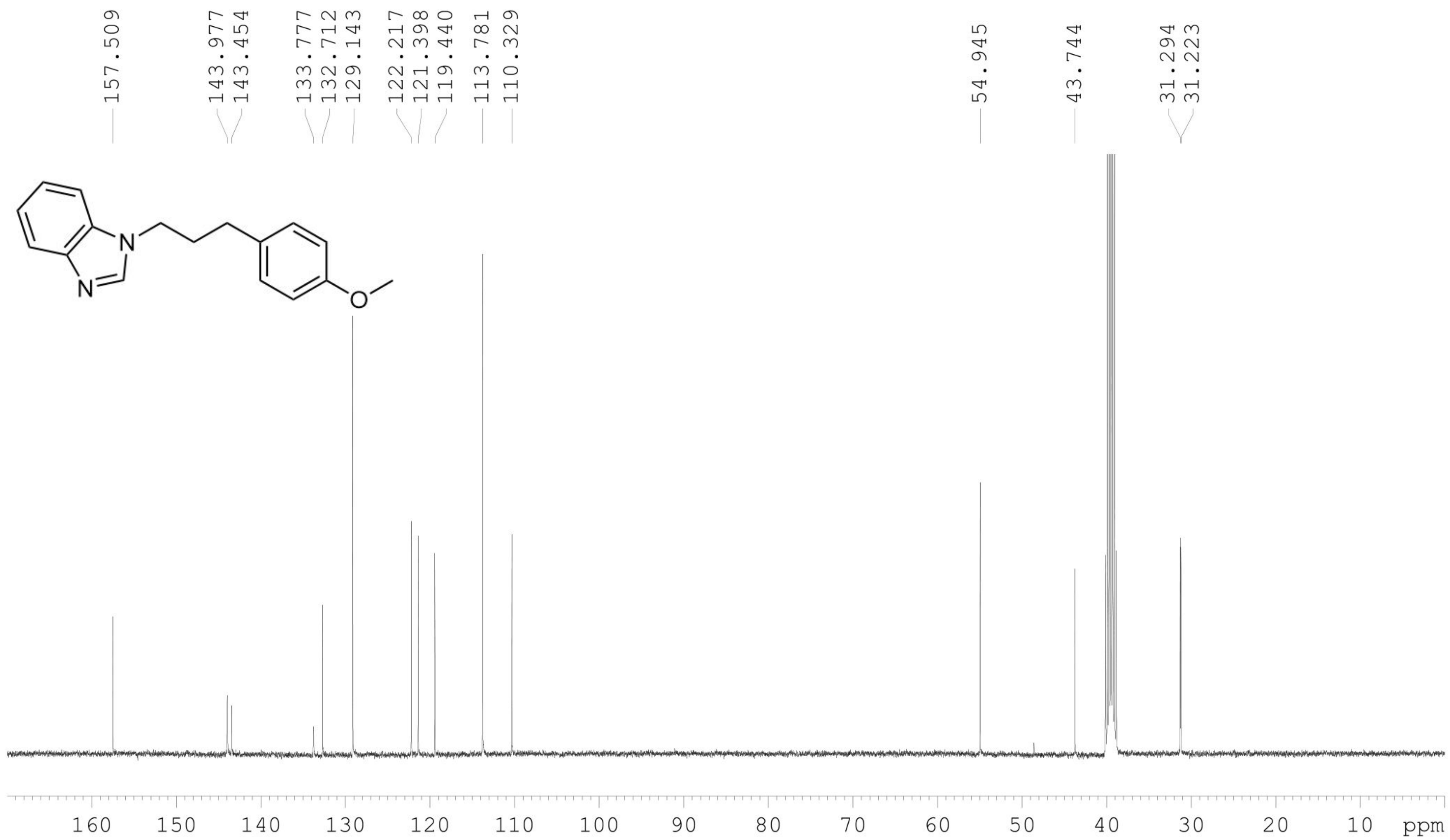

^{1}H NMR (DMSO, 400 MHz) of 66

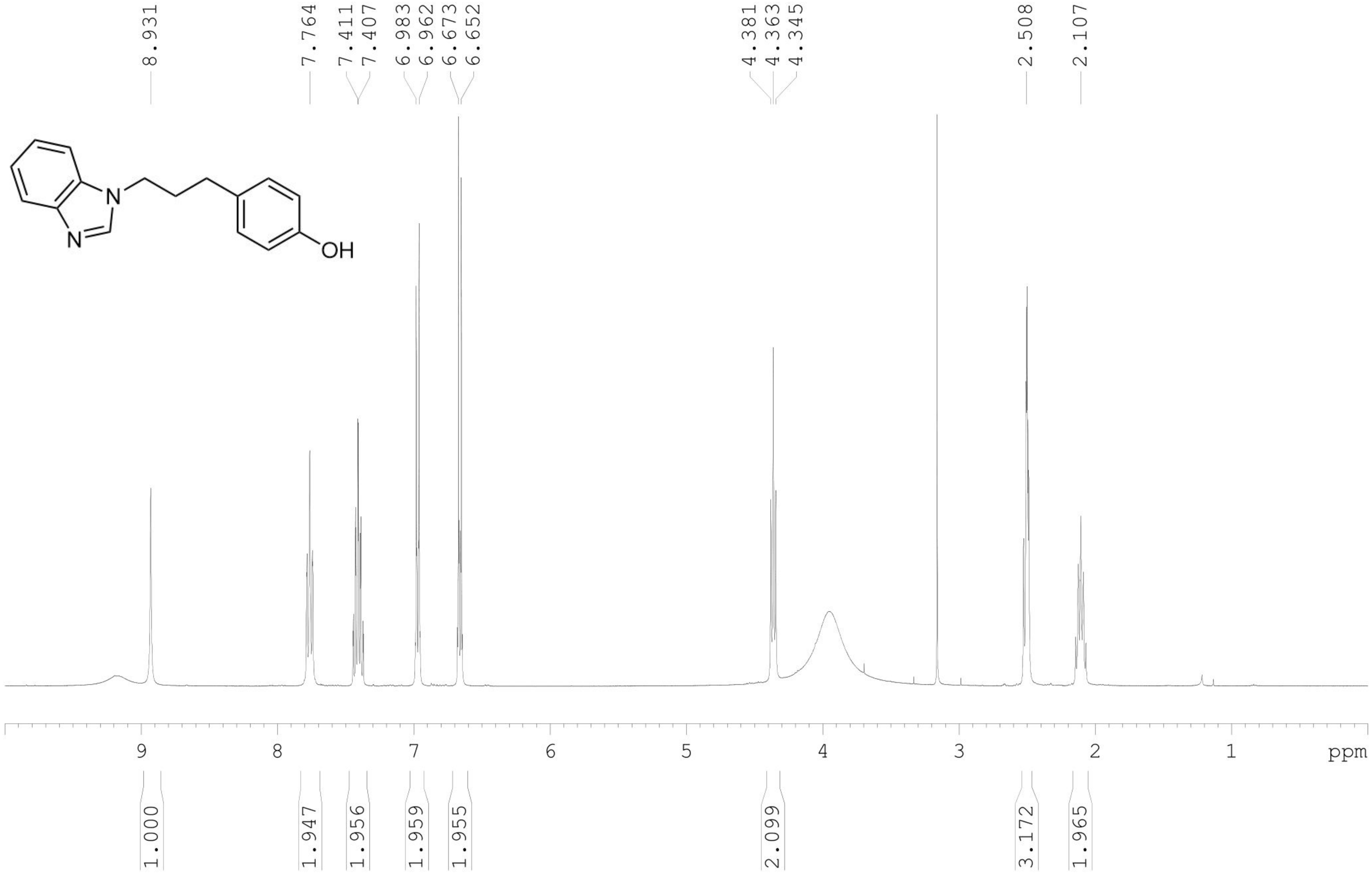

^{13}C NMR (DMSO, 100 MHz) of 66

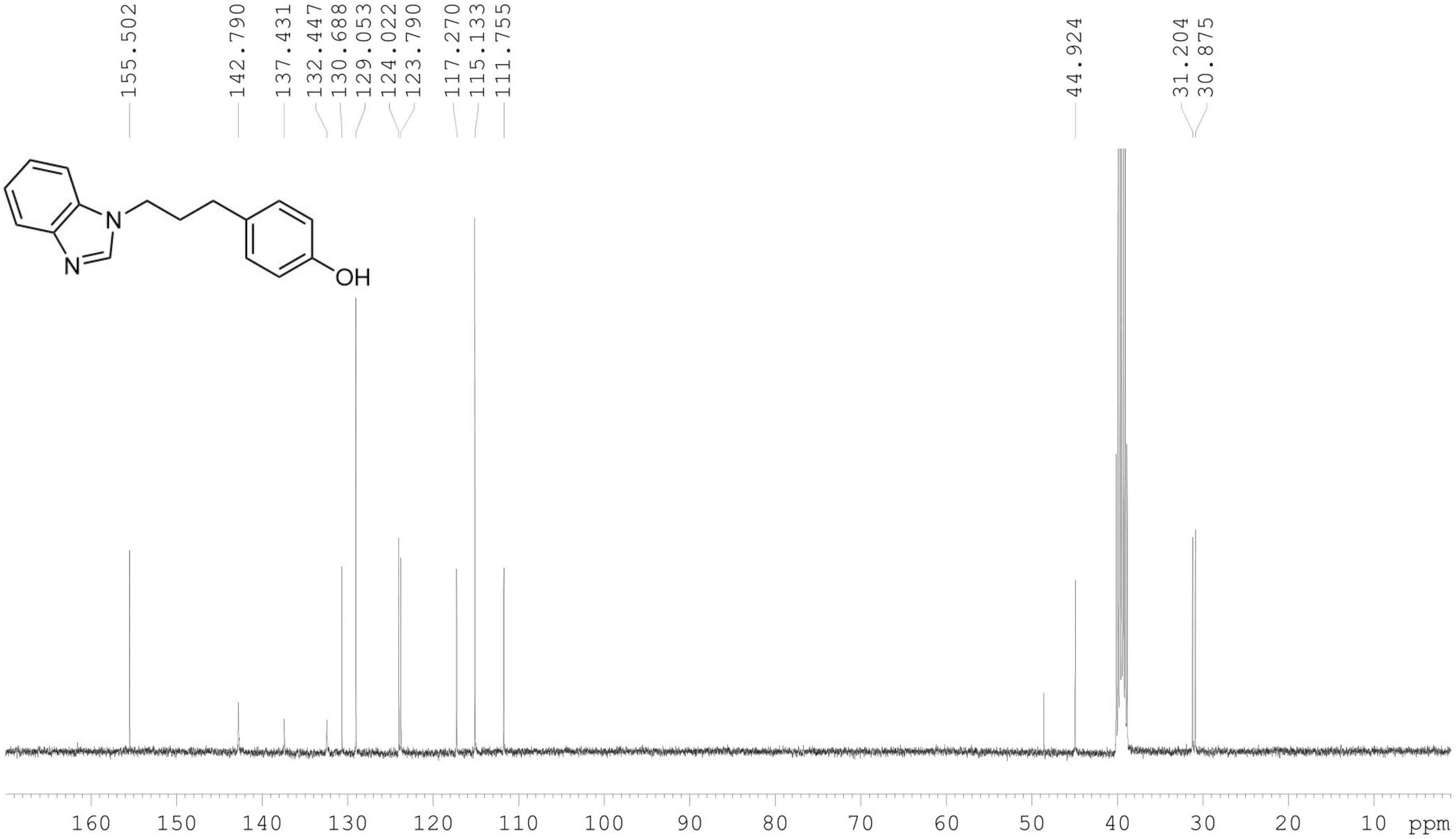

^{1}H NMR ($CDCl_3$, 400 MHz) of 67

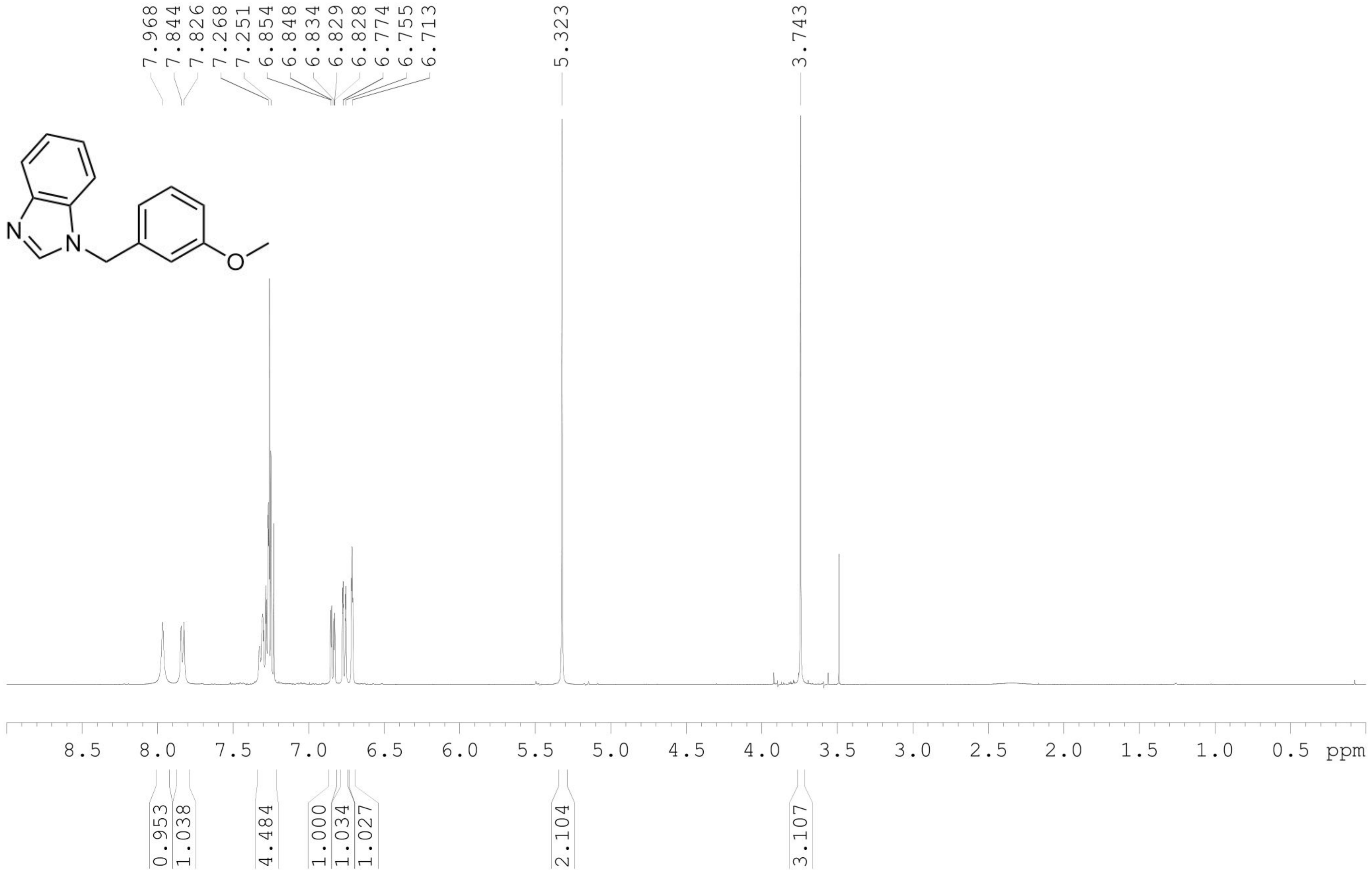

^{13}C NMR ($CDCl_3$, 100 MHz) of 67

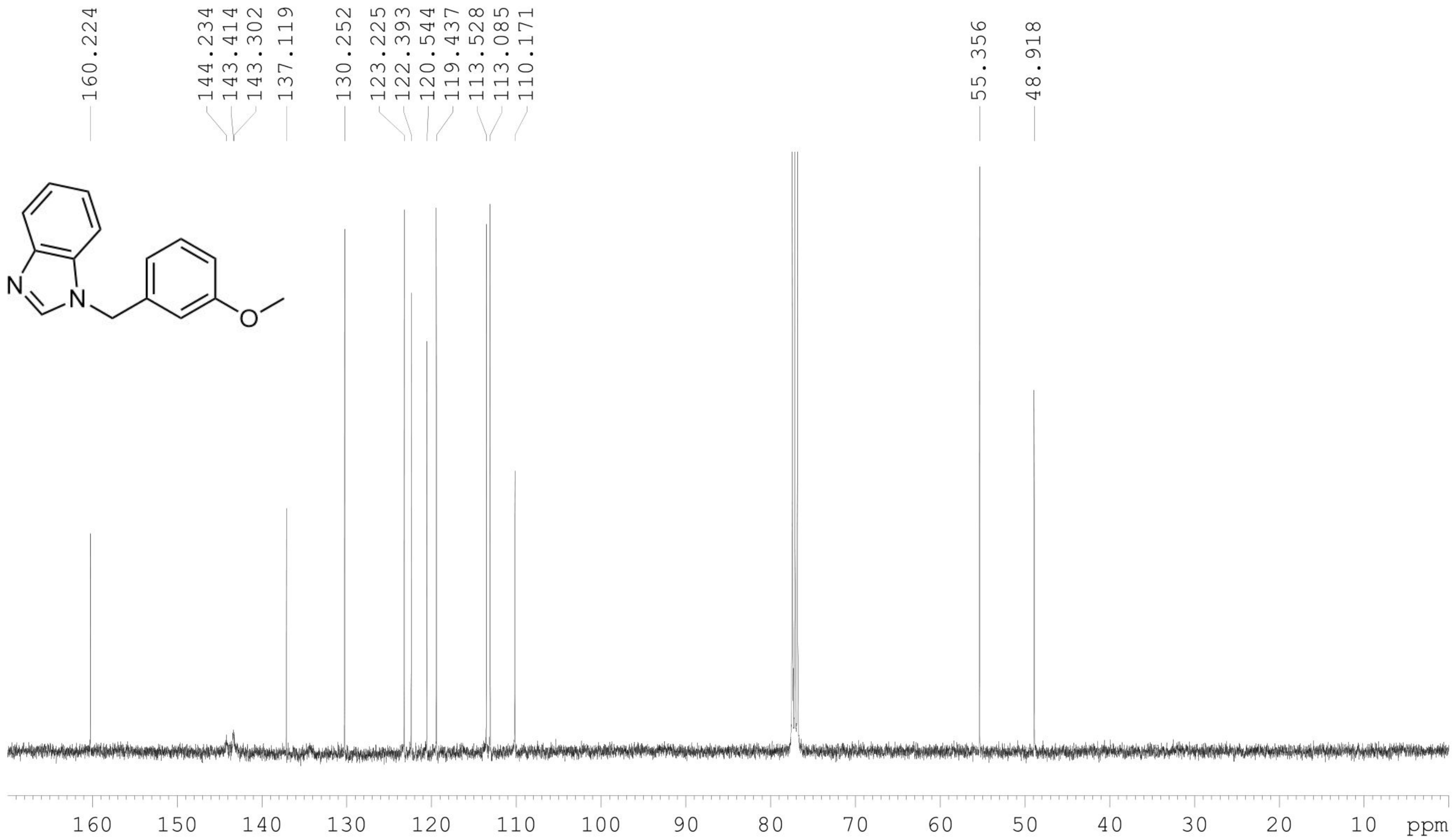

^{1}H NMR (DMSO, 400 MHz) of 68

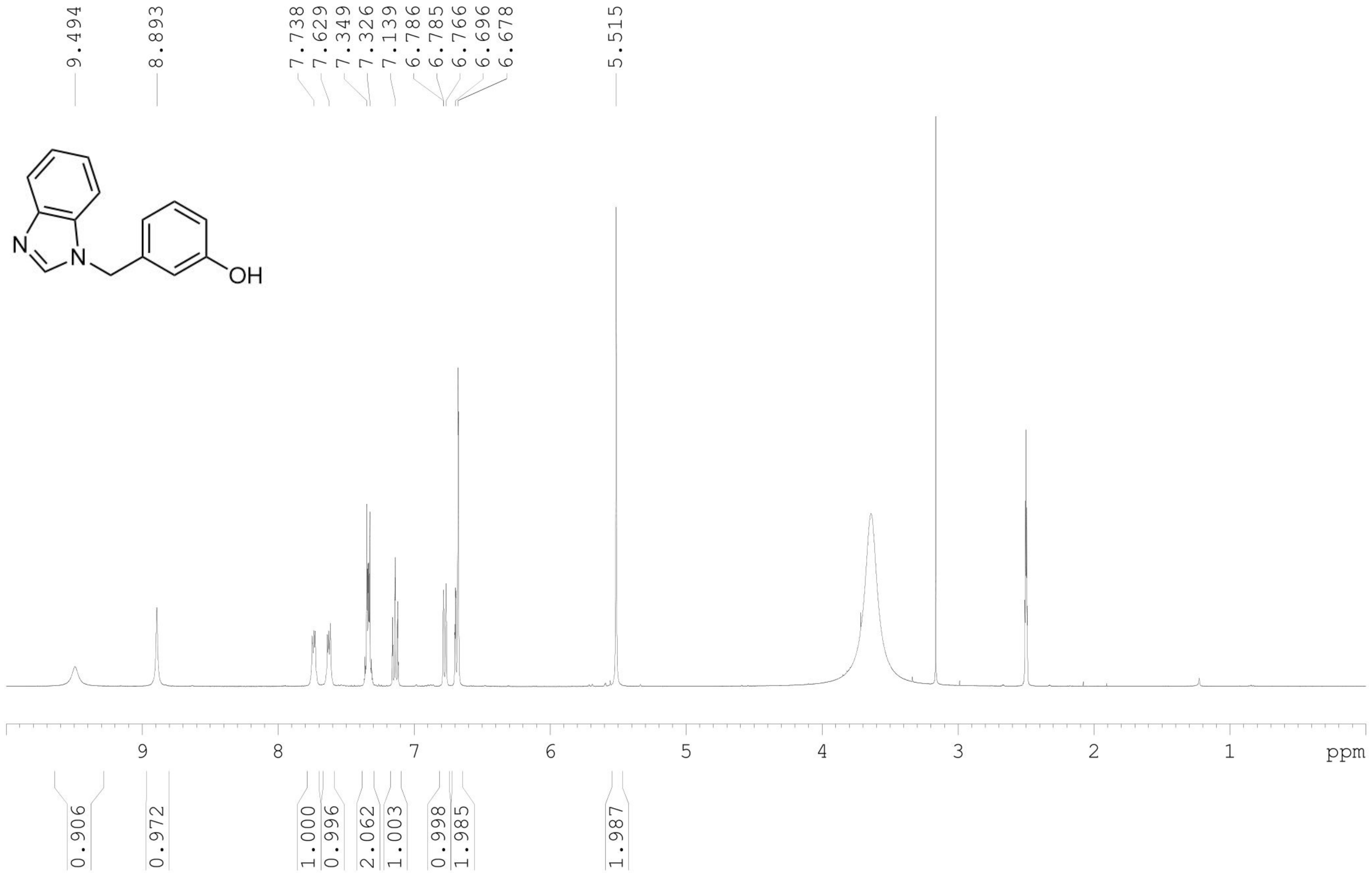

^{13}C NMR (DMSO, 100 MHz) of 68

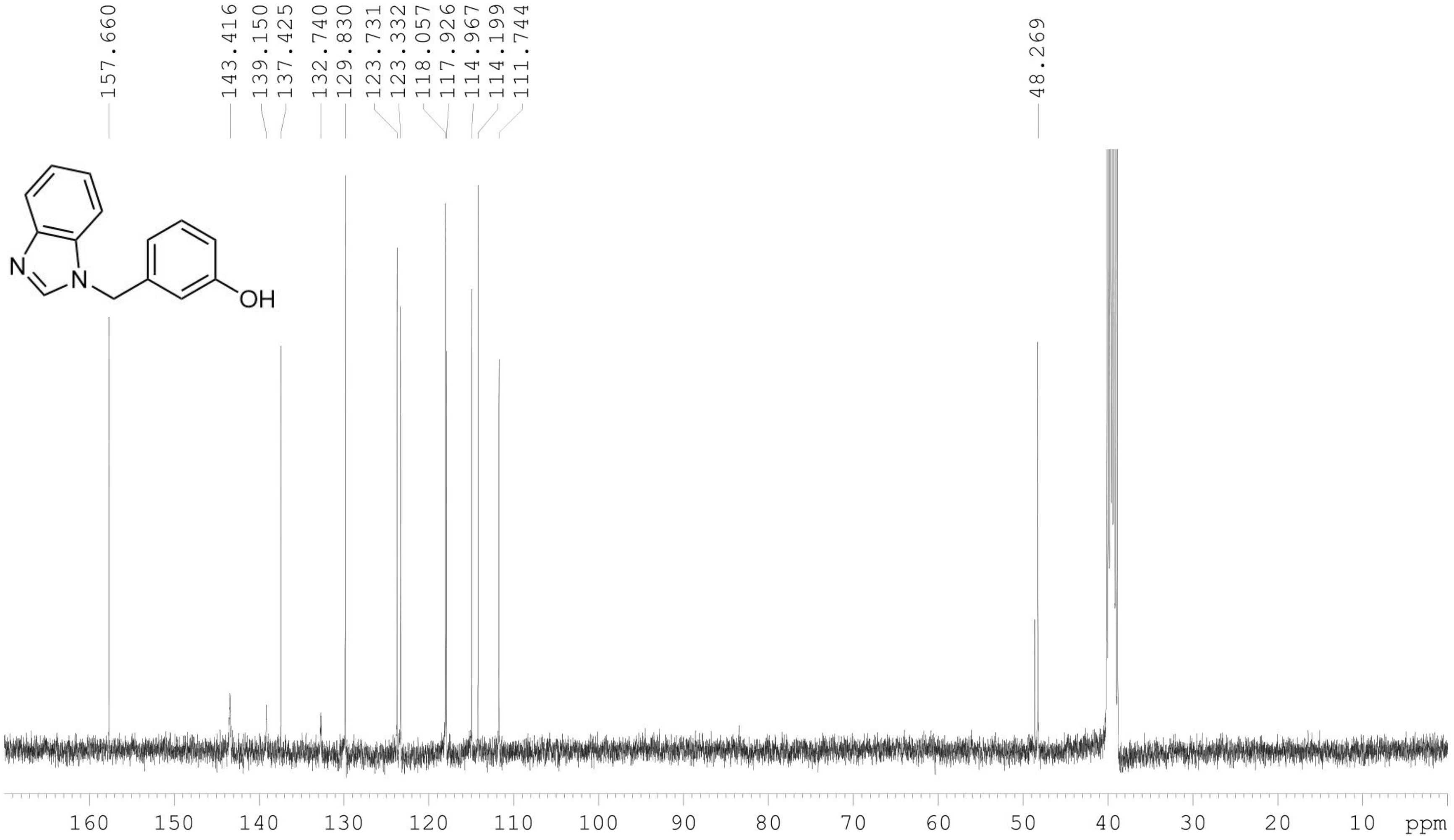

^{1}H NMR (DMSO, 400 MHz) of 69

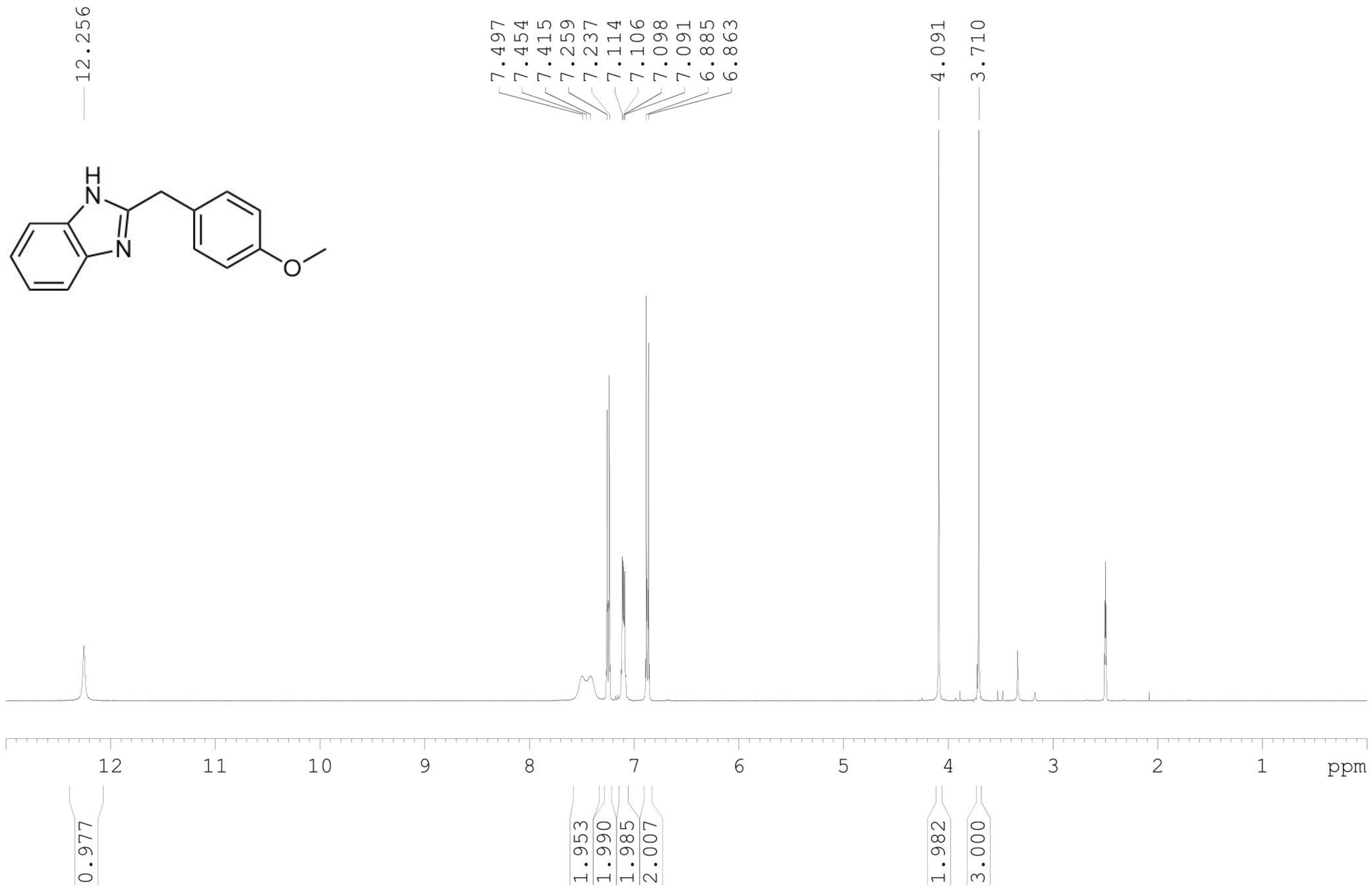

^{13}C NMR (DMSO, 100 MHz) of 69

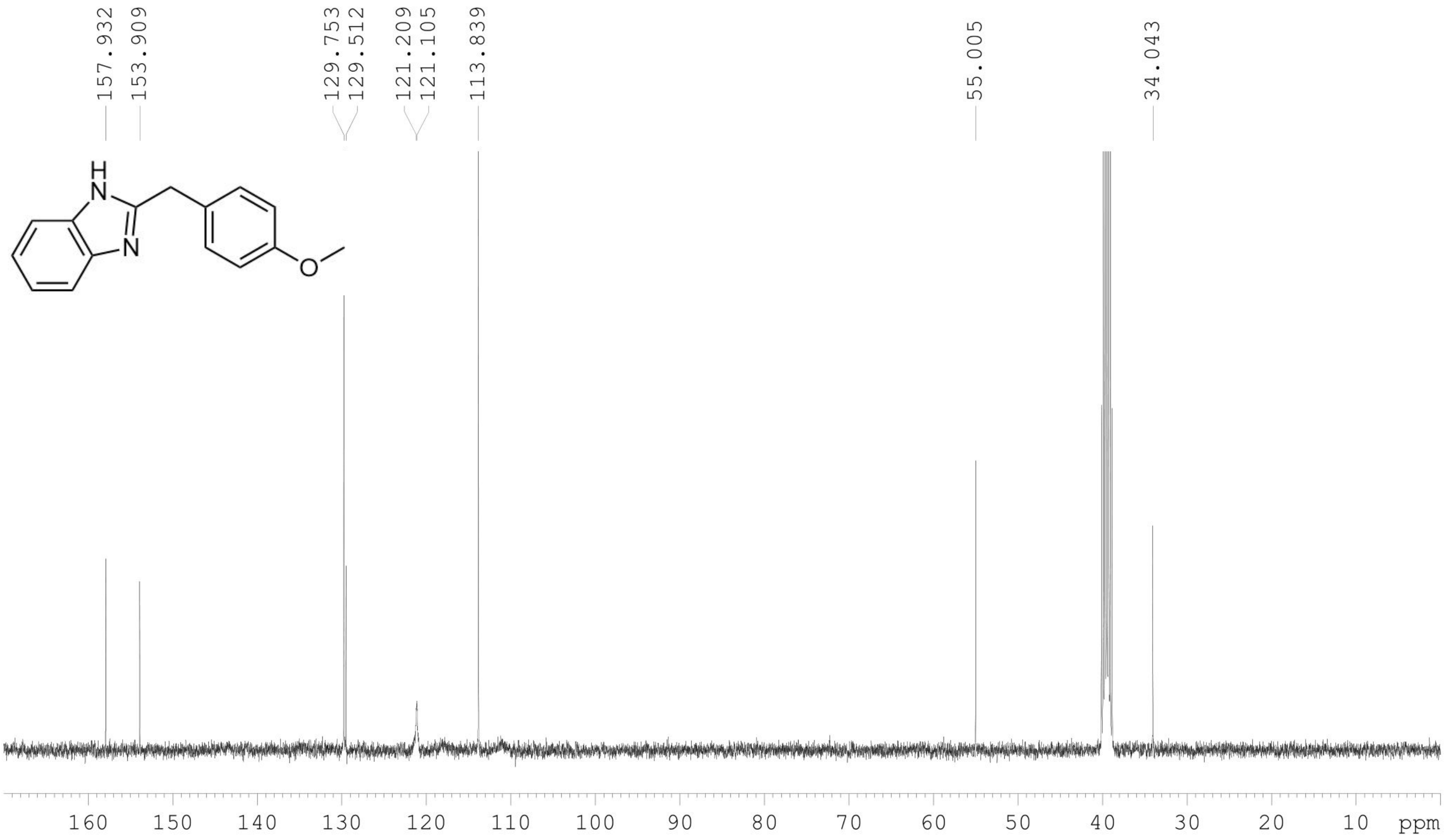

^{1}H NMR (DMSO, 400 MHz) of 70

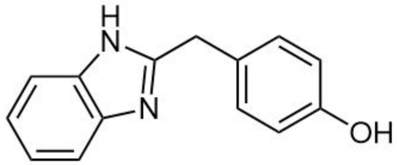

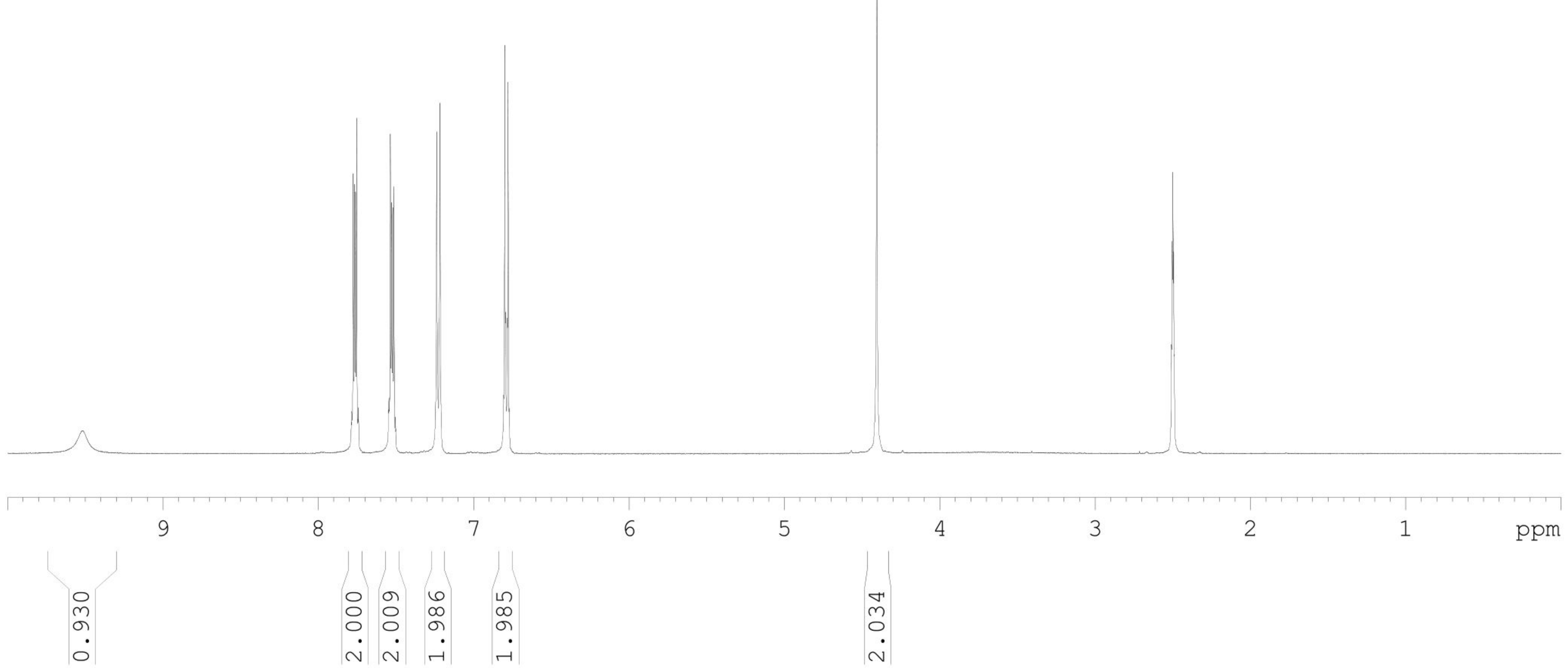

^{13}C NMR (DMSO, 100 MHz) of 70

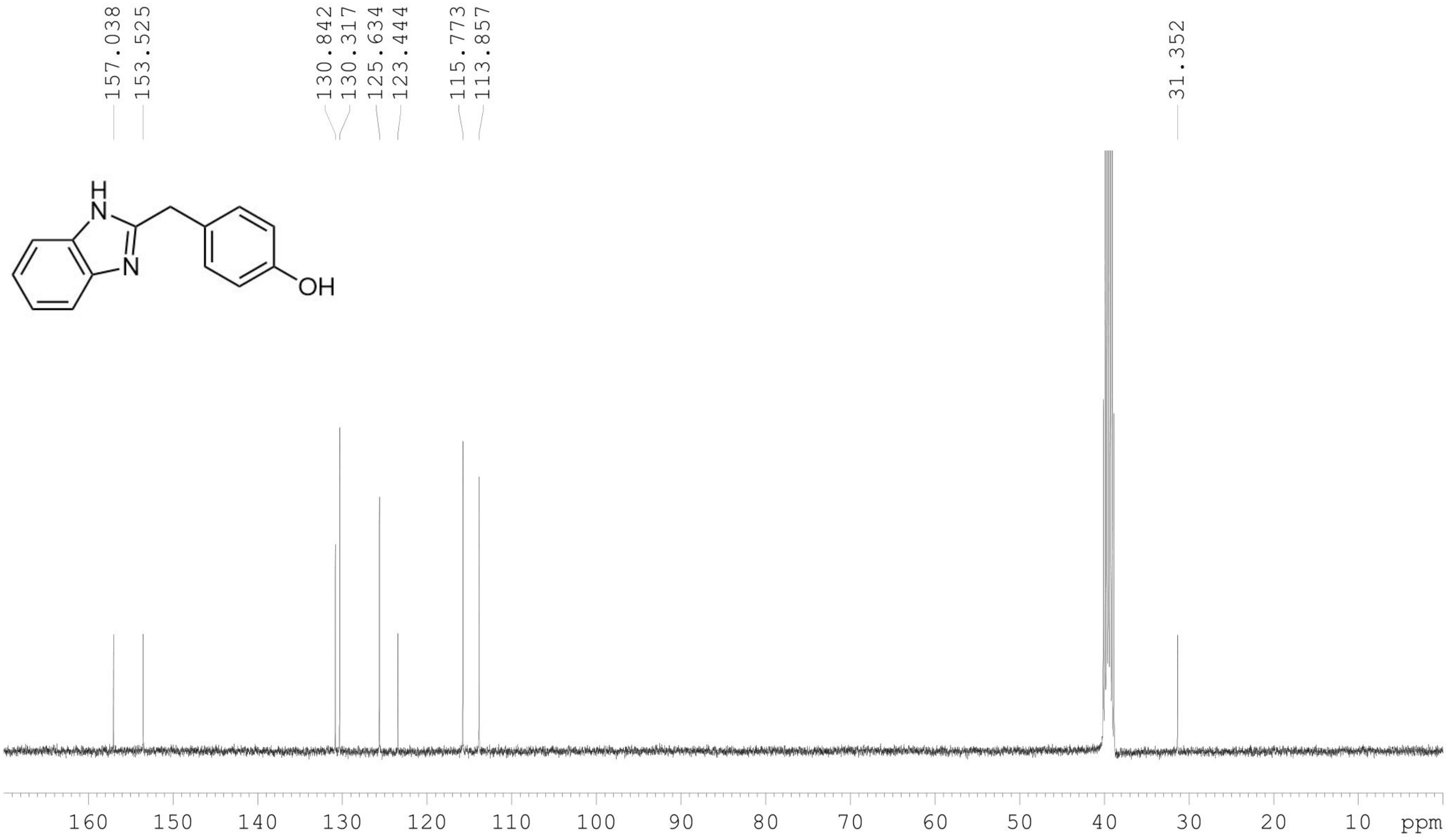

^{1}H NMR ($CDCl_3$, 400 MHz) of 71

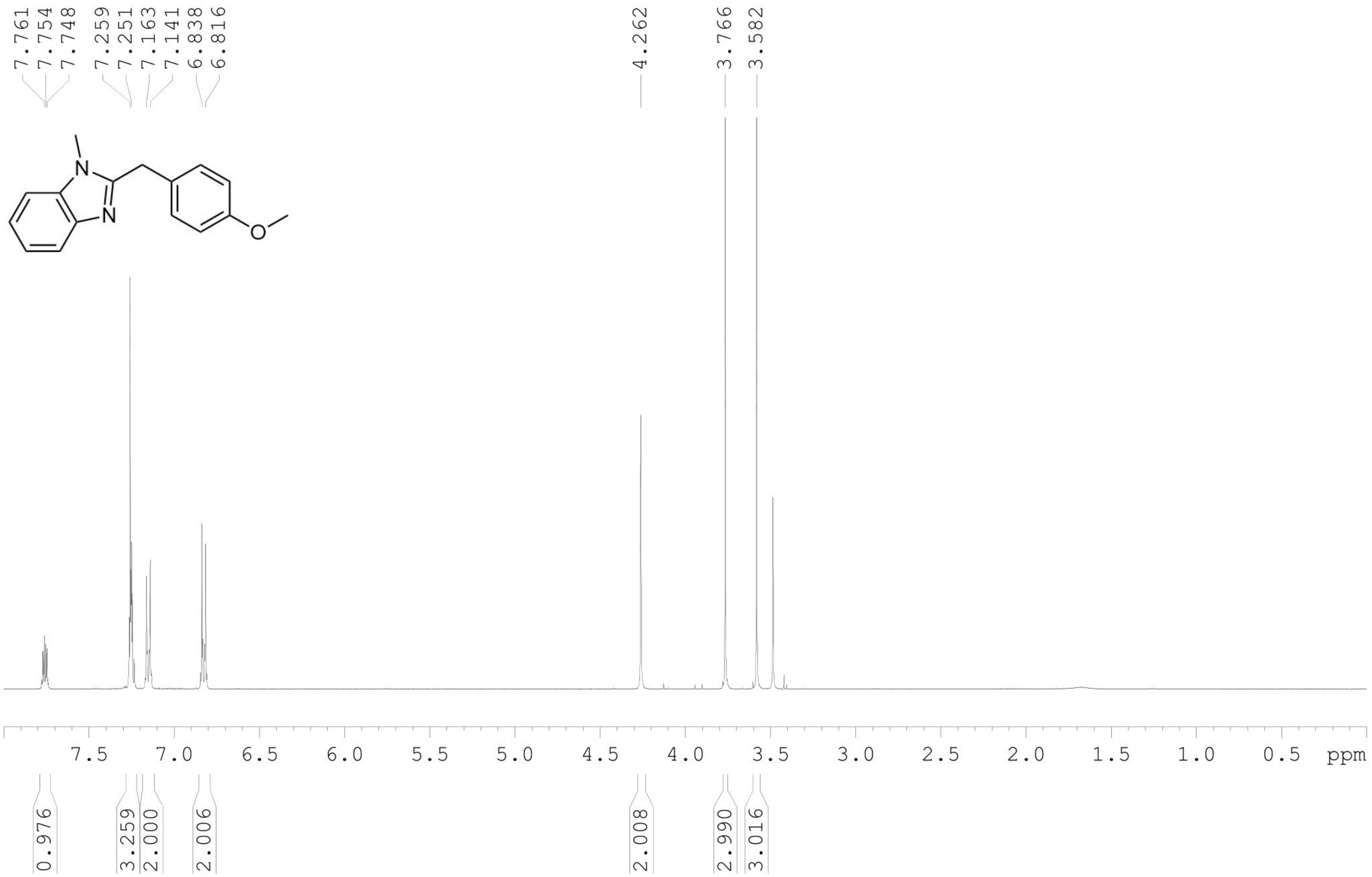

^{13}C NMR ($CDCl_3$, 100 MHz) of 71

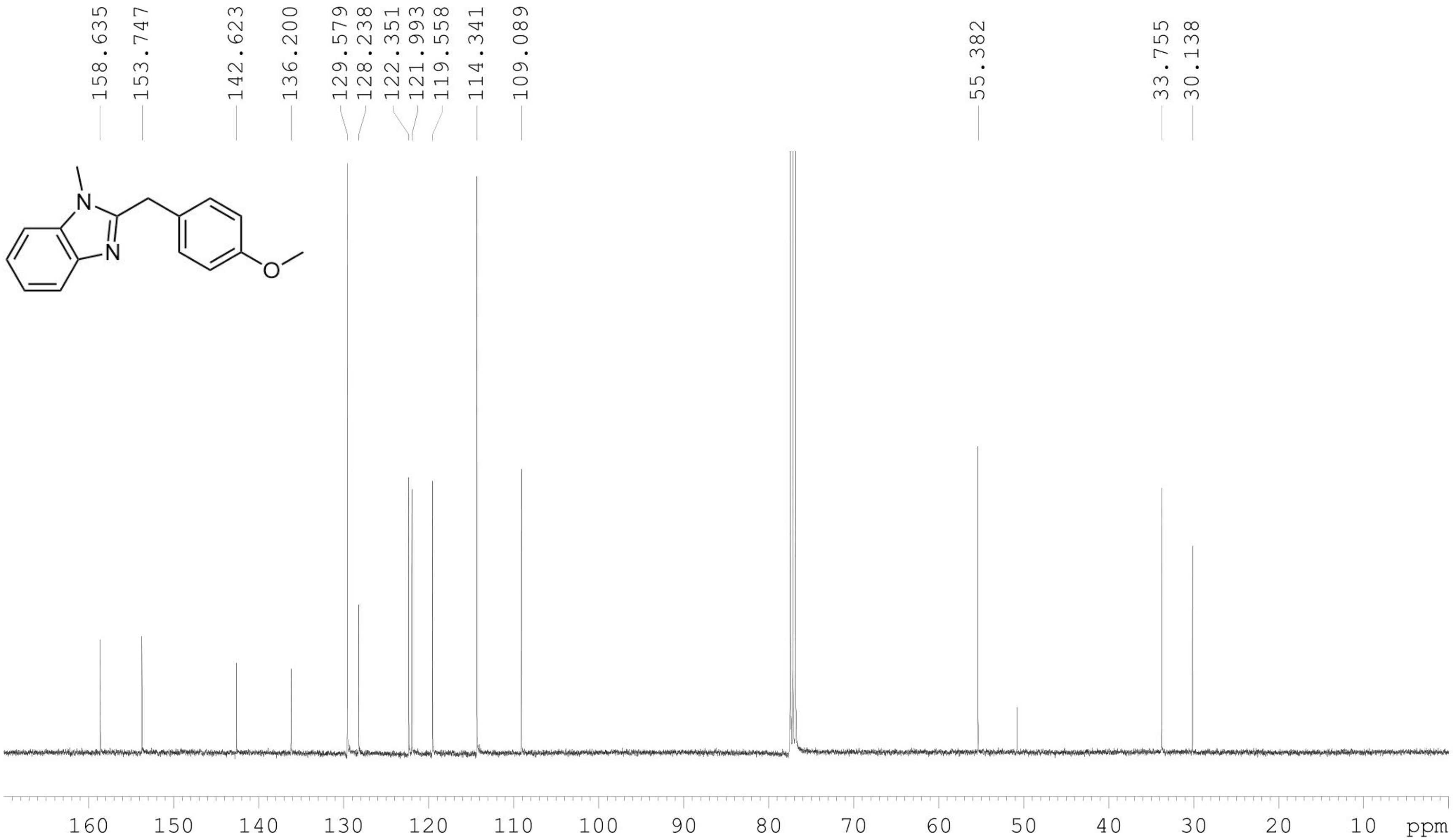

^{1}H NMR (DMSO, 400 MHz) of 72

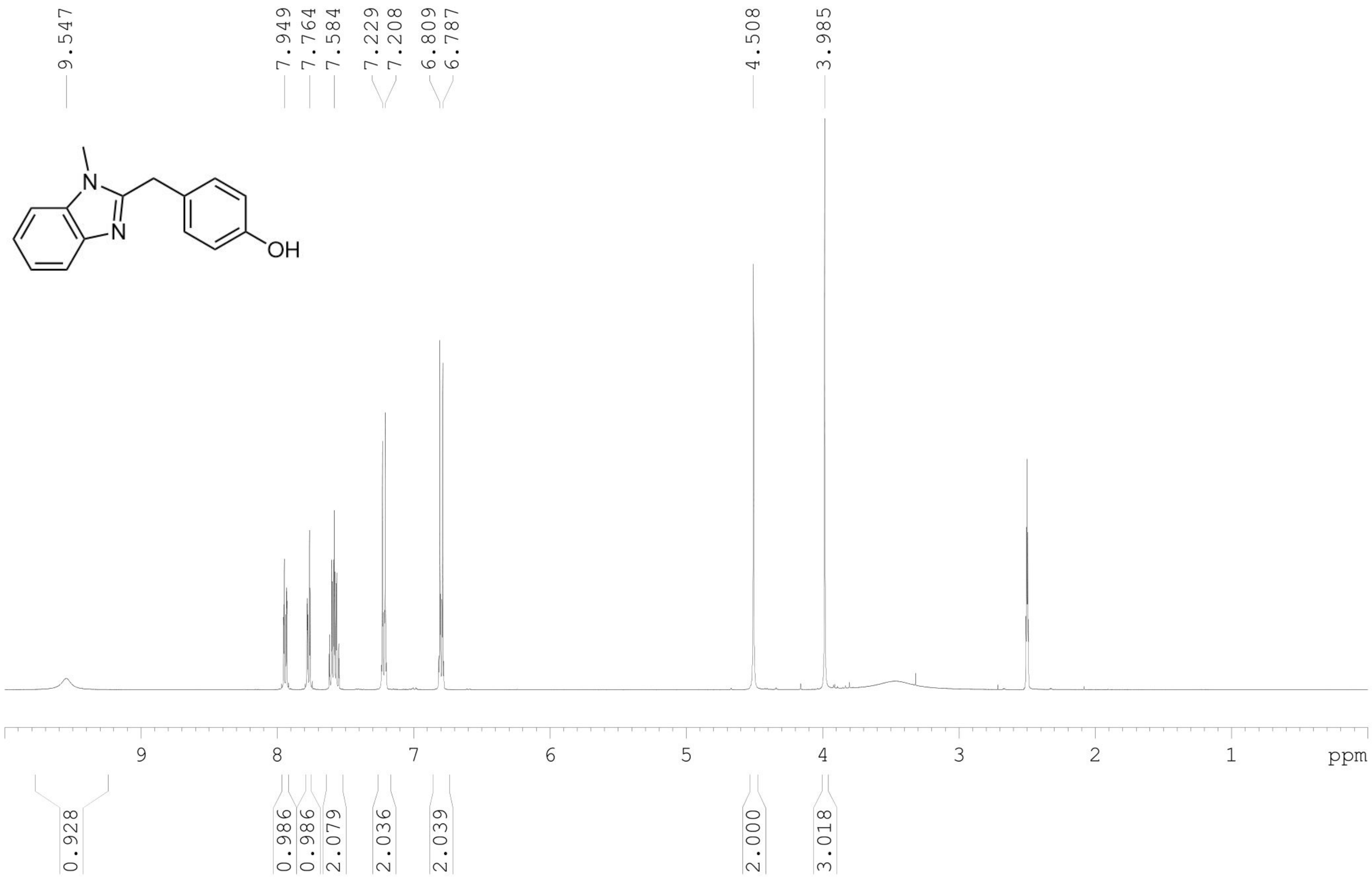

^{13}C NMR (DMSO, 100 MHz) of 72

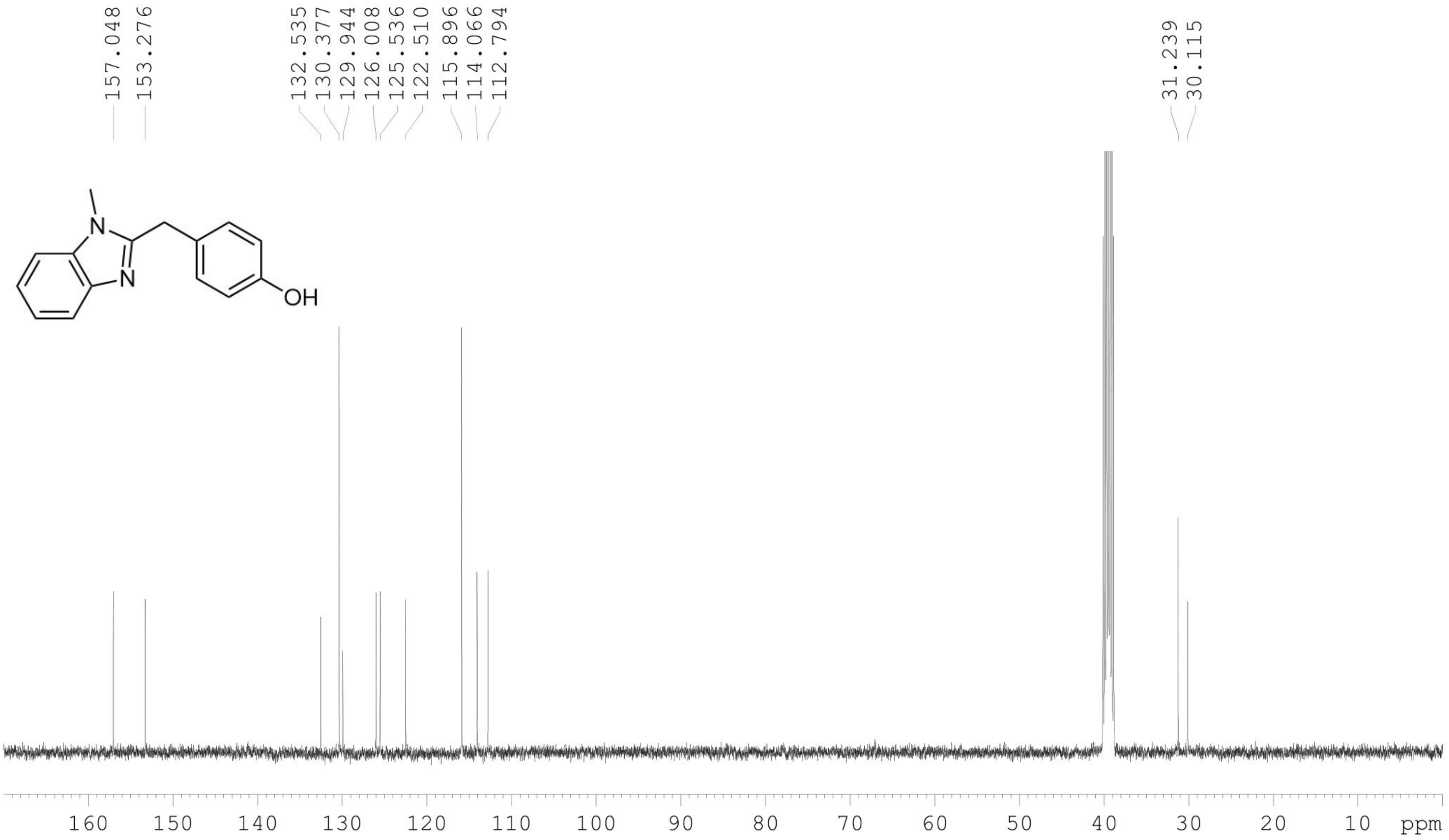

^{1}H NMR (DMSO, 400 MHz) of 73

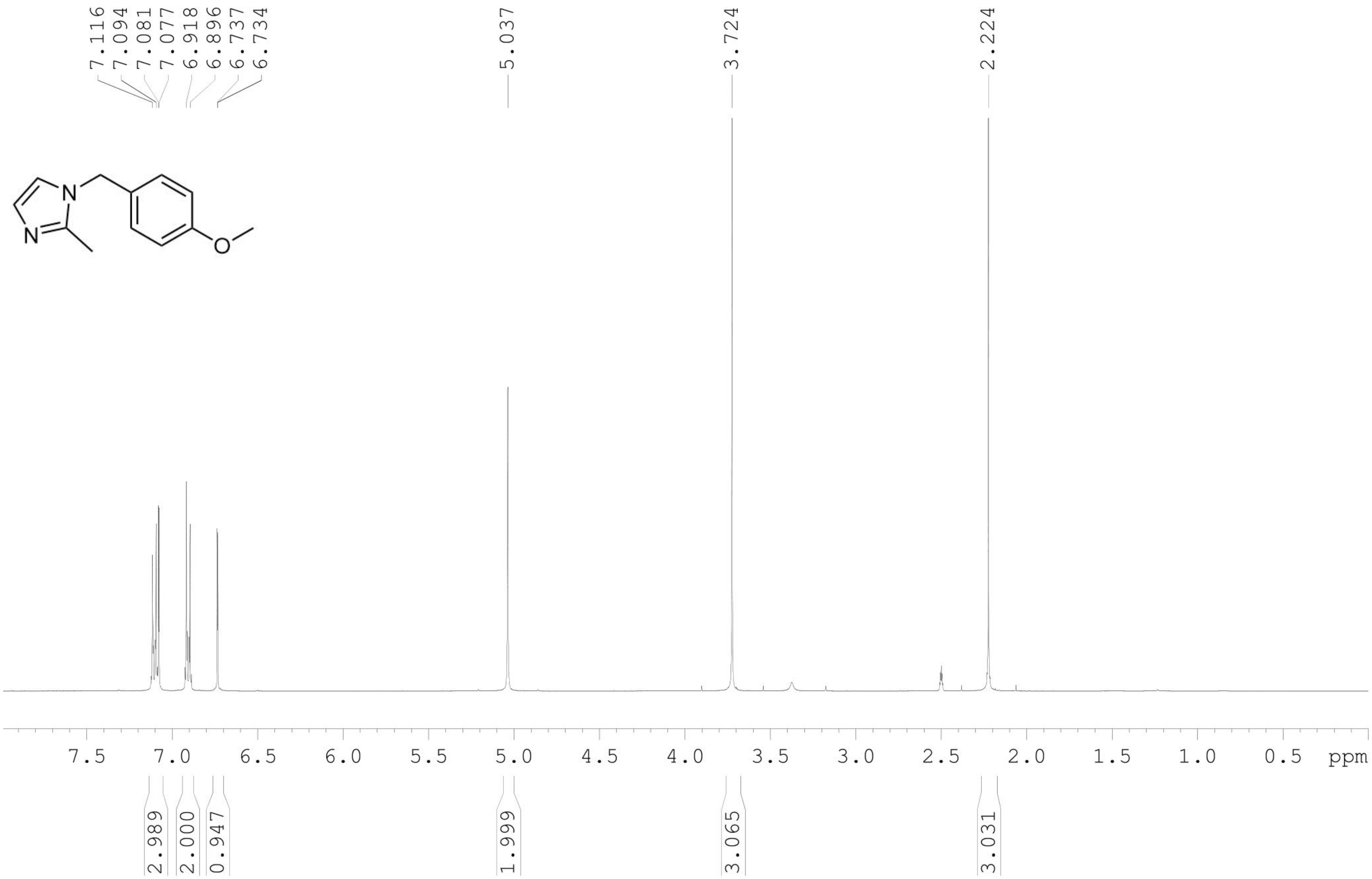

^{13}C NMR (DMSO, 100 MHz) of 73

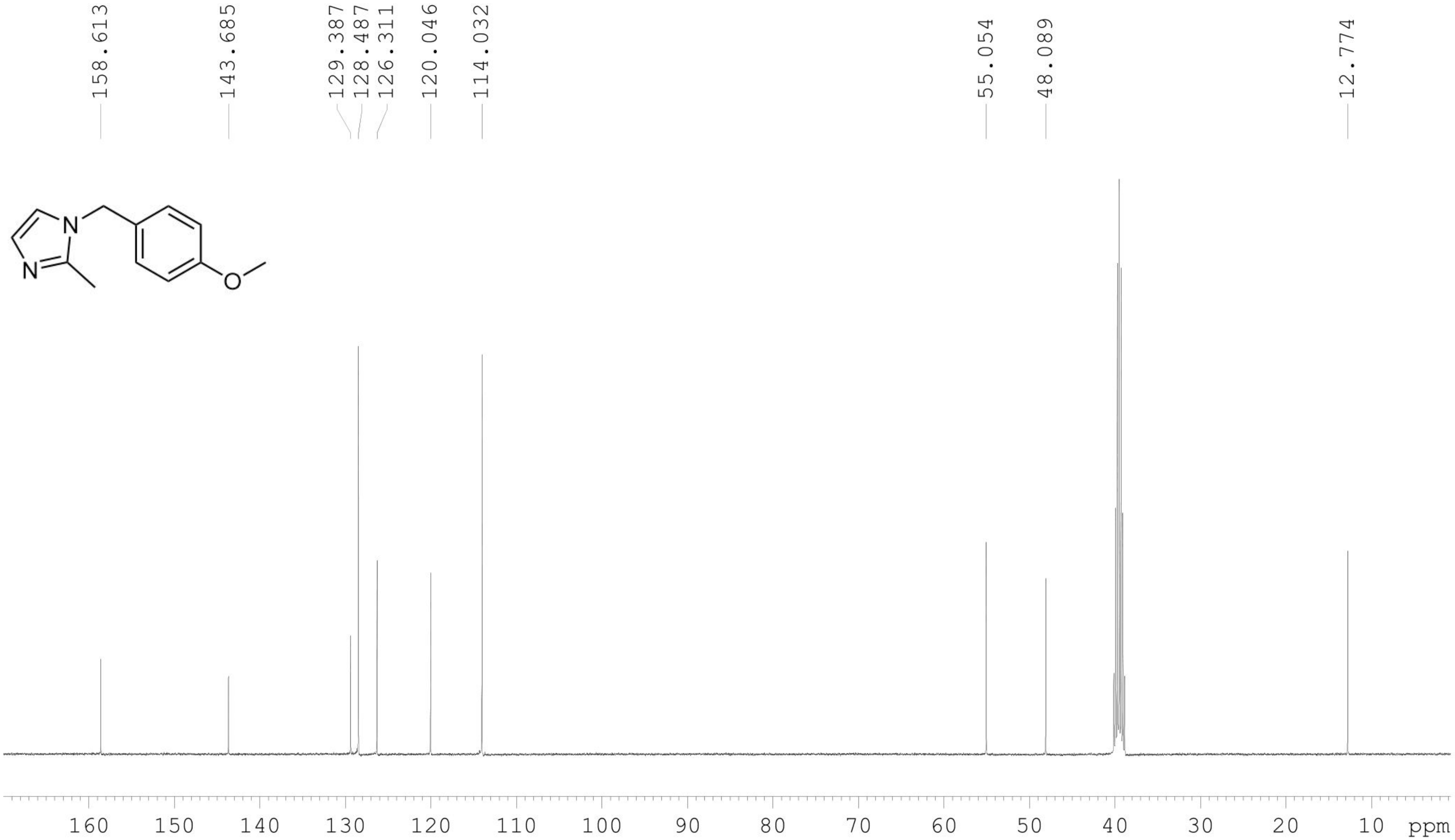

^{1}H NMR (DMSO, 400 MHz) of 74

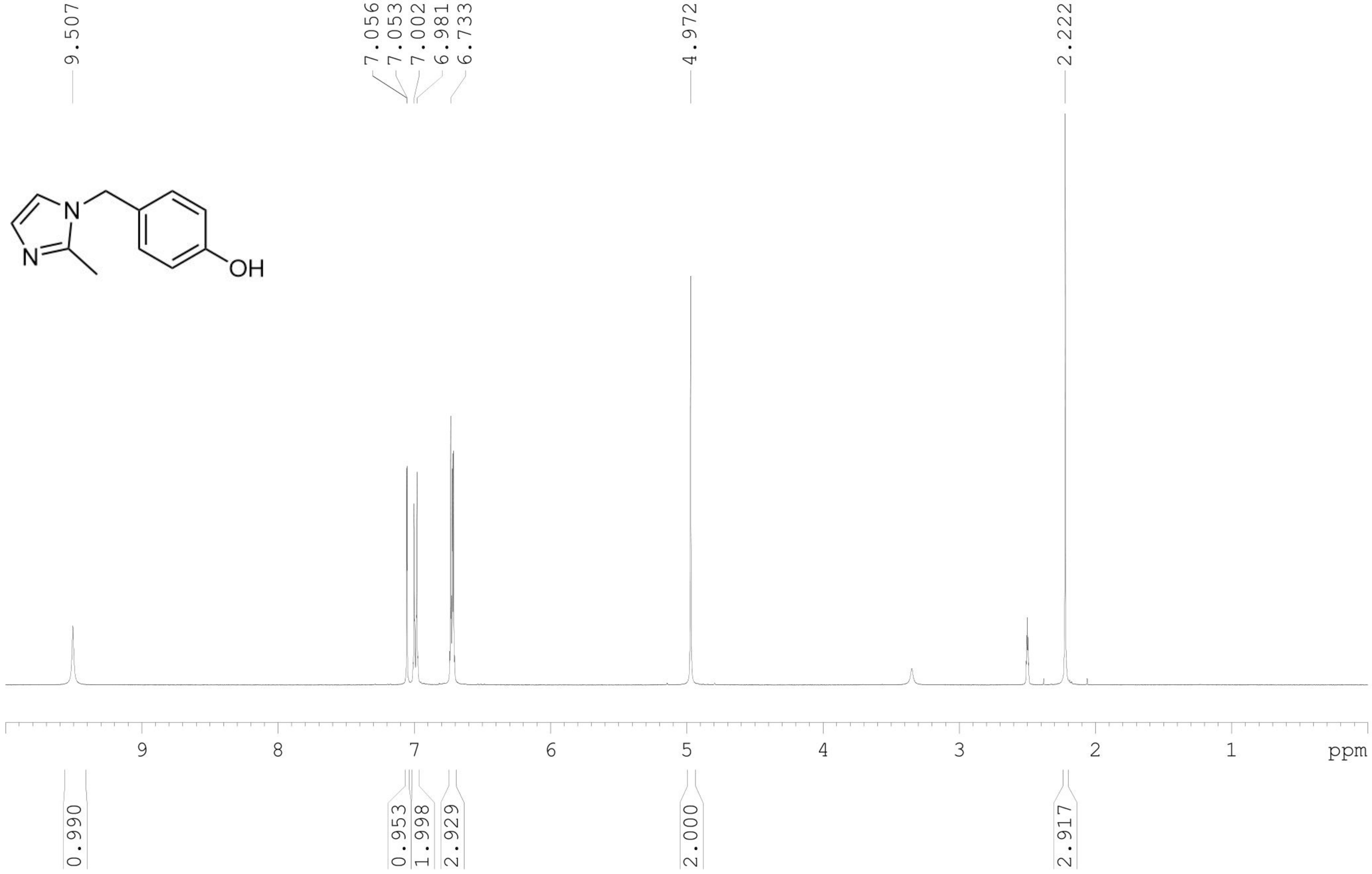

^{13}C NMR (DMSO, 100 MHz) of 74

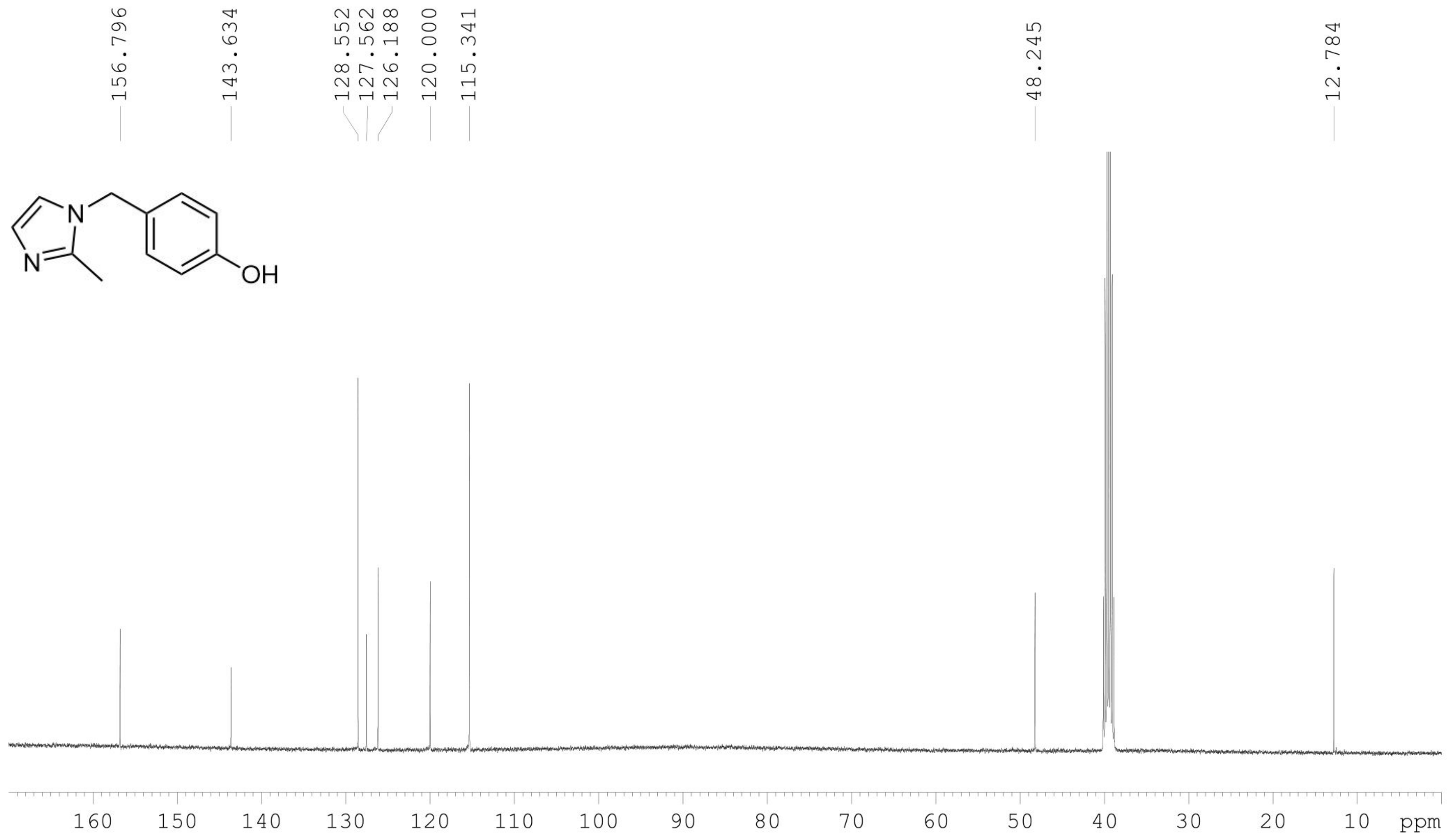

^{1}H NMR (DMSO, 400 MHz) of 75

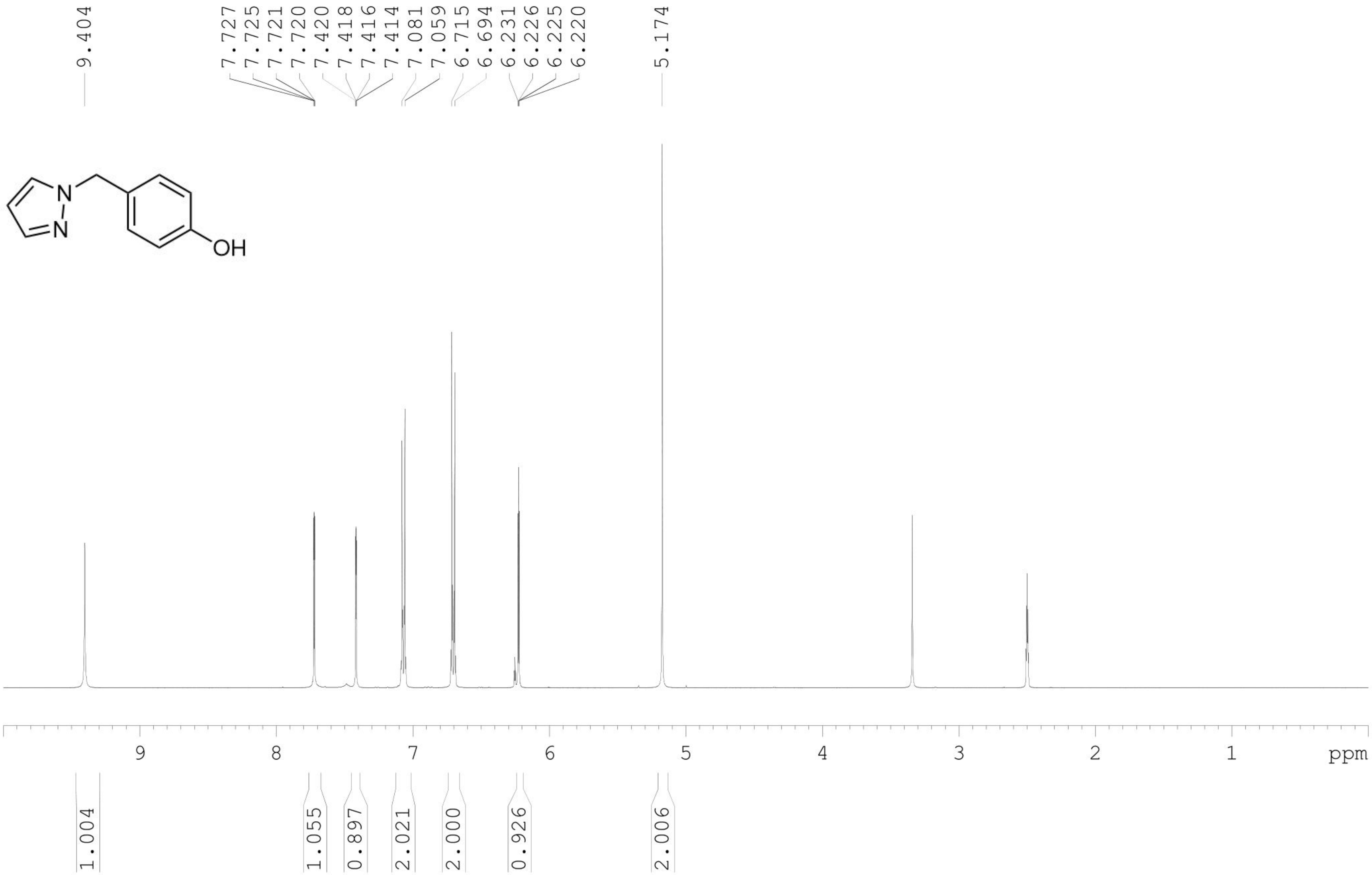

^{13}C NMR (DMSO, 100 MHz) of 75

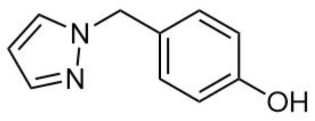

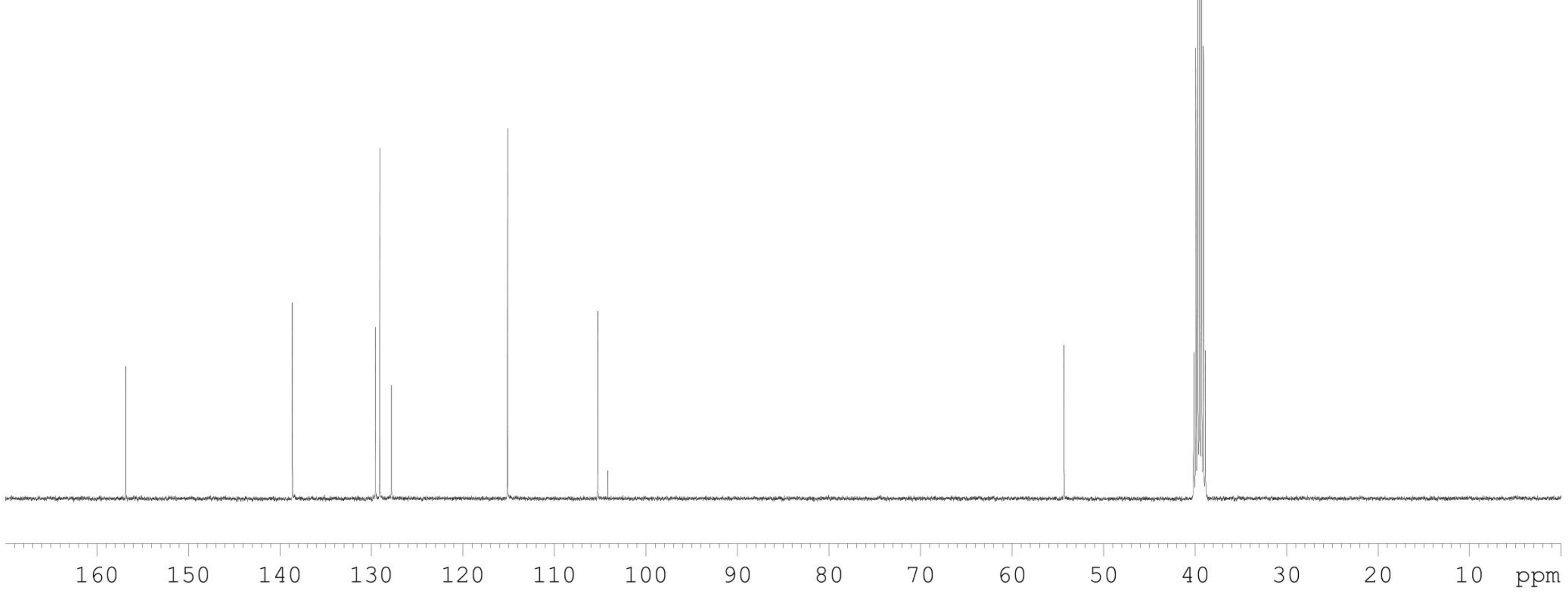

Supplement: Supplementary file 1 [file cn5c00631_si_001.pdf]
